# Enantioselective Synthesis of Axially Chiral Diaryl Ethers via Rhodium‐Catalyzed [2 + 2 + 2] Cycloaddition

**DOI:** 10.1002/anie.3486845

**Published:** 2026-06-03

**Authors:** Yu Sato, Julong Jiang, Kairi Yamashiro, Ken Tanaka

**Affiliations:** ^1^ Department of Chemical Science and Engineering Institute of Science Tokyo Tokyo Japan; ^2^ Institute for Chemical Reaction Design and Discovery (WPI‐ICReDD) Hokkaido University Sapporo Hokkaido Japan

**Keywords:** [2 + 2 + 2] cycloaddition, alkynyl ethers, asymmetric catalysis, axial chirality, diaryl ethers, rhodium

## Abstract

Axially chiral diaryl ethers are important structural motifs in biologically active molecules, functional materials, and chiral catalysts. However, their enantioselective synthesis remains challenging because rotation around the Ar–O bond often occurs with relatively low rotational barriers, and most existing methods rely on desymmetrization strategies that inherently limit the accessible substitution patterns. Here we report a rhodium‐catalyzed enantioselective [2 + 2 + 2] cycloaddition of triynes bearing alkynyl ether units that provides axially chiral diaryl ethers with high enantioselectivity. In this transformation, benzannulation simultaneously constructs the aromatic ring and restricts rotation about the Ar–O bond while introducing diverse substituents, thereby enabling efficient atroposelective synthesis. The reaction exhibits a broad substrate scope and affords the desired products in good yields and enantioselectivities. In suitably designed substrates, the benzannulation process generates multiple atropisomeric axes in a single step, providing complex stereochemical architectures with up to four stereogenic axes. DFT calculations indicate that steric and dispersion interactions during oxidative cyclization govern the stereochemical outcome, and a stepwise alkyne insertion, featuring shortened Rh–C bonds in the transition state, likely enables efficient stereoinduction within the chiral environment, accounting for the observed high enantioselectivity.

## Graphical abstract

An enantioselective rhodium‐catalyzed [2 + 2 + 2] cycloaddition enables the synthesis of axially chiral diaryl ethers. Carbonyl coordination ensures high reactivity and regioselectivity, affording products bearing up to four stereogenic axes with good enantio‐ and diastereoselectivity.

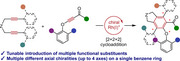

## 1 Introduction

**FIGURE 1:**
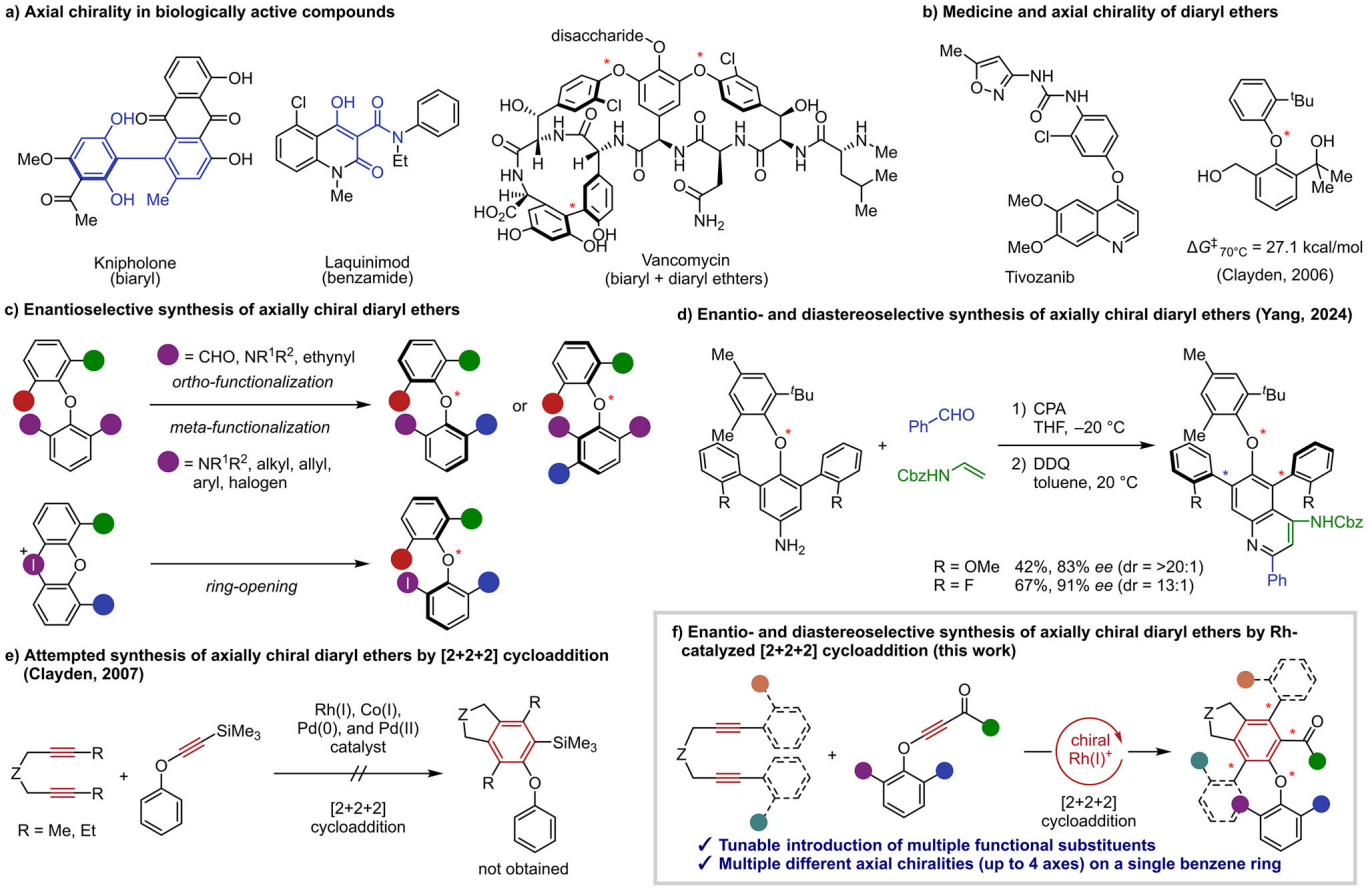
Axially chiral compounds, including diaryl ethers (a and b) and enantioselective synthesis of axially chiral diaryl ethers (c–f). CPA = chiral phosphoric acid. DDQ = 2,3‐dichloro‐5,6‐dicyano‐1,4‐benzoquinone. Cbz = benzyloxycarbonyl.

Axially chiral compounds constitute an important class of molecules widely employed as core frameworks in active pharmaceutical ingredients (Figure 1a, left), chiral catalysts, and chiral organic materials [1, 2, 3]. On the basis of the configurational stability of atropisomerism, they are classified according to the axial rotation barrier (ΔE^‡^_rot_) into Class 1 (< 20 kcal/mol), Class 2 (20–30 kcal/mol), and Class 3 (> 30 kcal/mol) [4]. Although research has traditionally focused on configurationally stable Class 3 atropisomers, such as biaryls [1, 2, 3], increasing attention has recently been directed toward more flexible Class 2 systems, including benzamides (Figure 1a, middle) [5] and diaryl ethers [6, 7, 8]. Moreover, certain molecules, exemplified by vancomycin (Figure 1a, right), exhibit outstanding biological activity arising from the combination of multiple highly functionalized, relatively low‐barrier axial chiral elements [9, 10, 11]. However, stereocontrol of low‐barrier axial chirality, particularly in systems containing multiple axes, remains challenging. General methods that enable the simultaneous construction and functionalization of multiple stereogenic axes are still limited [12, 13, 14, 15, 16, 17, 18, 19, 20, 21, 22].

Among the Class 2 atropisomers, diaryl ether scaffolds are found in bioactive molecules (Figure 1b, left) [23, 24, 25]. are particularly interesting because they can possess axial chirality within their structurally diverse frameworks. Since the 2006 study by Clayden and co‐workers (Figure 1b, right) [26], significant progress has been made in the synthesis and characterization of stereochemically stable examples. However, their inherent conformational flexibility makes stereocontrol particularly challenging. Although strategies such as dynamic kinetic resolution [27]. and enantioselective desymmetrization of prochiral precursors via substituent installation [28, 29, 30, 31, 32, 33, 34, 35, 36, 37, 38, 39, 40]. or ring‐opening [41] have been reported (Figure 1c); most existing approaches rely on desymmetrization processes, which inherently limit the accessible substitution patterns of axially chiral diaryl ethers. Furthermore, methods capable of simultaneously introducing and controlling multiple stereogenic axes remain exceedingly rare; to date, the only example achieving simultaneous control of three axes is the enantioselective desymmetrization reported by Yang and co‐workers (Figure 1d) [34].

On the other hand, the [2 + 2 + 2] cycloaddition [42, 43, 44] represents a powerful strategy for the rapid construction and diversification of aromatic rings and has been extended to the synthesis of various axially chiral frameworks through appropriate alkyne design [21, 22, 45, 46, 47, 48, 49, 50, 51, 52, 53, 54]. Cationic rhodium(I) catalysts operate under mild conditions [55, 56, 57]. and are well suited for the enantioselective construction of axially chiral structures with relatively low rotational barriers. In such benzannulation processes, the aromatic ring is formed while rotation around the newly generated bonds becomes restricted, which can give rise to multiple atropisomeric axes. Therefore, the simultaneous construction of several stereogenic axes is, in principle, feasible. However, Clayden and co‐workers reported that the [2 + 2 + 2] cycloaddition of an alkynyl ether bearing a terminal trimethylsilyl group failed to deliver the desired diaryl ether products (Figure 1e) [58]. In contrast, we previously demonstrated that alkynyl ethers bearing a terminal ester moiety exhibit high reactivity, affording homo‐[2 + 2 + 2] cycloaddition products in high yields with excellent regioselectivity [59, 60].

Here we report the enantioselective synthesis of axially chiral diaryl ethers via a rhodium‐catalyzed [2 + 2 + 2] cycloaddition of 1,6‐diynes with alkynyl ethers (Figure 1f). By employing alkynyl ethers bearing a coordinating carbonyl group at the terminus, the reaction proceeds with high reactivity and regioselectivity, enabling efficient transformation of substrates bearing bulky *ortho* substituents. In suitably designed substrates, the benzannulation process generates multiple atropisomeric axes, affording compounds bearing up to four stereogenic axes with good enantio‐ and diastereoselectivity. Mechanistic investigations, including DFT calculations, reveal a stepwise alkyne insertion pathway distinct from previously proposed concerted insertion processes and provide insight into the origin of the observed reactivity and enantioselectivity.

## 2 Results and Discussion

We investigated the optimization of the [2 + 2 + 2] cycloaddition between unsymmetrical 1,6‐diyne and alkynyl ether **2a** (Table 1). First, we examined the [2 + 2 + 2] cycloaddition of 1,6‐diyne **1a** with **2a** in the presence of 10 mol% of cationic rhodium(I)/bisphosphine catalysts. Screening of axially chiral biaryl bisphosphine ligands revealed that regioisomers **3** and **4** are obtained in comparable yields (entries 1–5); in contrast, no reaction occurs when substituents are added to the aryl groups on the phosphorus atoms (entries 6 and 7). For regioisomer **3**, the highest enantioselectivity was observed using Difluorphos (91% *ee*, entry 5). In contrast, for regioisomer **4**, the best result was achieved with H_8_‐BINAP (62% *ee*, entry 1), although the enantioselectivity was markedly lower than that observed for **3**. To suppress yield loss arising from homo‐[2 + 2 + 2] cycloaddition of **1a**, the stoichiometry of **2a** was varied (entries 8 and 9), revealing that using two equiv of **2a** improved the yields of **3a*/*4a** (entry 8). By examining the concentration (entries 10 and 11), the yield was further improved at low concentrations of 3 mM (entry 11). The introduction of a methoxy group at the *ortho* position of the alkynyl ether (**2b**) improved regioselectivity, providing **3ab** selectively; however, the conversion of diyne **1a** was markedly reduced, resulting in low product yield (entry 12). We anticipated that the coordination of the methoxy group may inhibit the reaction. Thus, the addition of coordinating solvents was screened (entries 13–19), revealing that using a CH_2_Cl_2_/EtOH (1:4) mixed solvent system afforded **3ab** in 89% yield and 99% *ee* (entry 15). Notably, conducting the reaction of **2a** under the same conditions did not markedly affect either the yield or enantioselectivity of **3aa** (entry 20).

**TABLE 1:** Screening of monoyne substituents and reaction conditions^a^.

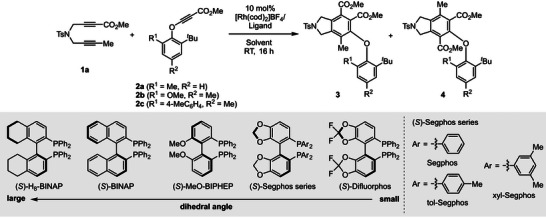					
Entry	2 (equiv)	Ligand	Solvent (mM)	3/% yield^b^ (% *ee*)	4/% yield^b^ (% *ee*)
1	**2a** (1.0)	(*S*)‐H_8_‐BINAP	CH_2_Cl_2_ (25)	14 (62, +)	20 (62, +)
2	**2a** (1.0)	(*S*)‐BINAP	CH_2_Cl_2_ (25)	27 (50, +)	23 (49, +)
3	**2a** (1.0)	(*S*)‐MeO‐BIPHEP	CH_2_Cl_2_ (25)	16 (78, +)	21 (22, +)
4	**2a** (1.0)	(*S*)‐Segphos	CH_2_Cl_2_ (25)	18 (84, +)	22 (11, +)
5	**2a** (1.0)	(*S*)‐Difluorphos	CH_2_Cl_2_ (25)	19 (91, +)	23 (4, –)
6	**2a** (1.0)	(*S*)‐tol‐Segphos	CH_2_Cl_2_ (25)	0	0
7	**2a** (1.0)	(*S*)‐xyl‐Segphos	CH_2_Cl_2_ (25)	0	0
8	**2a** (2.0)	(*S*)‐Difluorphos	CH_2_Cl_2_ (25)	29 (91, +)	27 (4, +)
9	**2a** (0.5)	(*S*)‐Difluorphos	CH_2_Cl_2_ (25)	24 (88, +)	32 (6, +)
10	**2a** (2.0)	(*S*)‐Difluorphos	CH_2_Cl_2_ (10)	31 (89, +)	34 (8, +)
**11**	**2a (2.0)**	**(*S*)‐Difluorphos**	**CH_2_Cl_2_ (3)**	**37 (90, +)**	**33 (10, +)**
12	**2b** (2.0)	(*S*)‐Difluorphos	CH_2_Cl_2_ (3)	12 (94, +)	4 (38, –)
13	**2b** (2.0)	(*S*)‐Difluorphos	CH_2_Cl_2_/EtOH = 1.5:1 (3)	56 (99, +)	2 (7, +)
14	**2b** (2.0)	(*S*)‐Difluorphos	CH_2_Cl_2_/EtOH = 1:1.5 (3)	80 (99, +)	8 (14, –)
**15**	**2b (2.0)**	**(*S*)‐Difluorphos**	**CH_2_Cl_2_/EtOH = 1:4 (3)**	**89 (99, +)**	**4 (4, –)**
16	**2b** (2.0)	(*S*)‐Difluorphos	CH_2_Cl_2_/*^i^* PrOH = 1:4 (3)	52 (94, +)	5 (8, –)
17	**2b** (2.0)	(*S*)‐Difluorphos	CH_2_Cl_2_/CF_3_CH_2_OH = 1:4 (3)	10 (96, +)	2 (5, +)
18	**2b** (2.0)	(*S*)‐Difluorphos	CH_2_Cl_2_/(CF_3_)_2_CHOH = 1:4 (3)	27 (95, +)	3 (18, –)
19	**2b** (2.0)	(*S*)‐Difluorphos	CH_2_Cl_2_/CH_3_CN = 1:4 (3)	4 (10, +)	12 (46, –)
20	**2a** (2.0)	(*S*)‐Difluorphos	CH_2_Cl_2_/EtOH = 1:4 (3)	41 (90, +)	30 (53, +)
21	**2c** (2.0)	(*S*)‐Difluorphos	CH_2_Cl_2_ (3)	34 (90, +)	35 (72, +)
22	**2c** (2.0)	(*S*)‐Difluorphos	CH_2_Cl_2_/EtOH = 1:4 (3)	14 (91, +)	8 (84, +)
23	**2c** (2.0)	(*S*)‐BINAP	CH_2_Cl_2_ (3)	14 (73, +)	18 (15, +)
**24**	**2c (2.0)**	**(*S*)‐BINAP**	**CH_2_Cl_2_/EtOH = 1:4 (3)**	**51 (89, +)**	**14 (36, +)**
25	**2c** (2.0)	(*S*)‐H_8_‐BINAP	CH_2_Cl_2_ (3)	6 (40, +)	9 (6, +)
26	**2c** (2.0)	(*S*)‐H_8_‐BINAP	CH_2_Cl_2_/EtOH = 1:4 (3)	51 (87, +)	10 (3, +)

^a^ **1** (0.025–0.10 mmol), **2** (0.025–0.10 mmol), [Rh(cod)_2_]BF_4_ and ligand (0.0025–0.010 mmol), and solvent (2.0–16.7 mL) were used.

^b^ Isolated yield. cod = 1,5‐cyclooctadiene.

*Note*: Bold values indicate the best reaction conditions.

For alkynyl ether **2c**, bearing a 4‐methylphenyl group at the *ortho* position, the use of Difluorphos afforded **3ac**/**4ac** in 34%/35% yields (entry 21), and a mixed solvent [CH_2_Cl_2_/EtOH (1:4)] system lowered the yields (entry 22). In contrast, the use of BINAP and H_8_‐BINAP afforded **3ac** selectively in 51% yields, along with **4ac** in 10%–14% yields (entries 24 and 26) in CH_2_Cl_2_/EtOH (1:4), but substantially low yields in CH_2_Cl_2_ (entries 23 and 25). BINAP was chosen as the ligand of choice due to its high enantioselectivity and low cost (entry 24).

**FIGURE 2:**
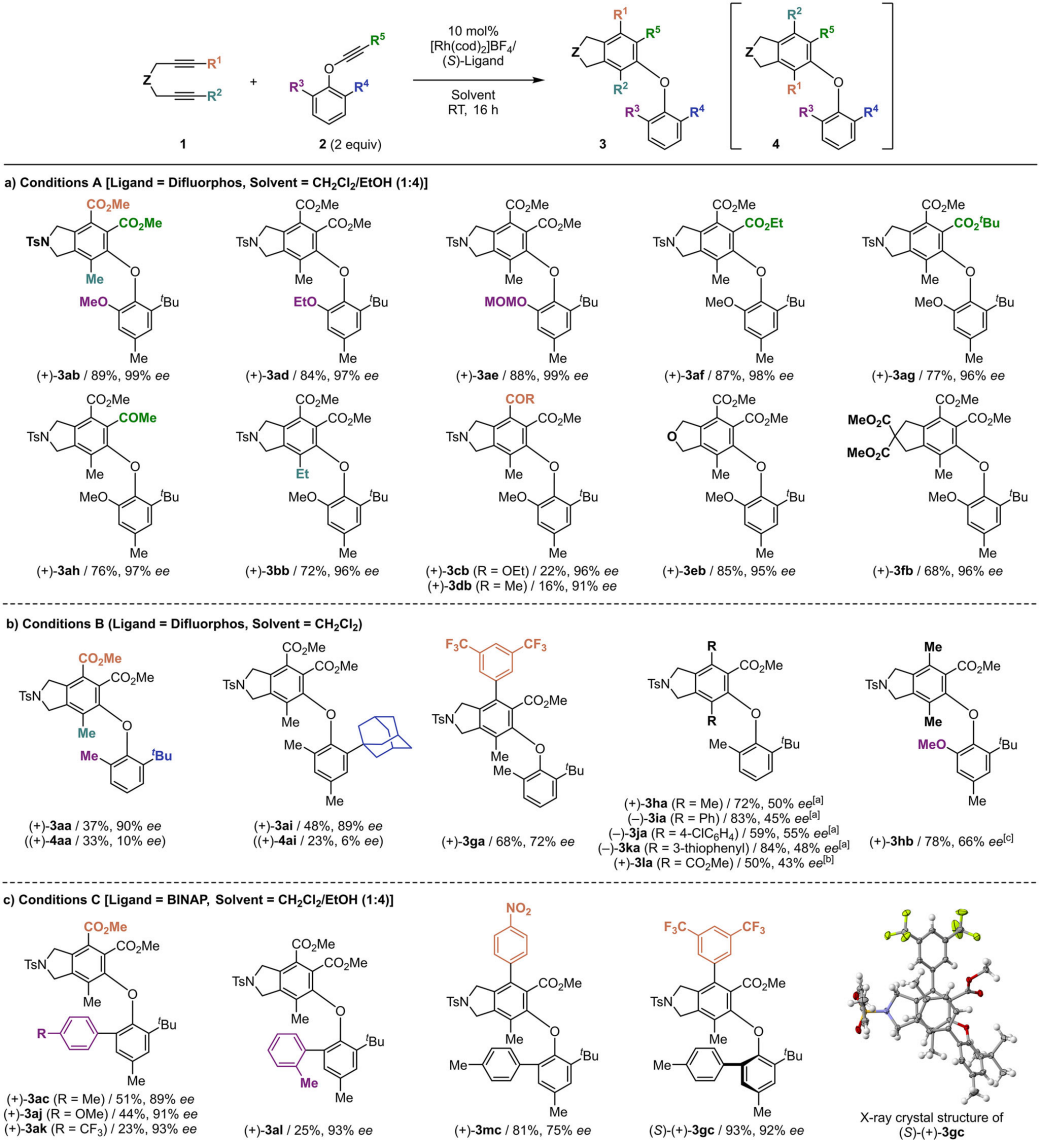
Enantioselective synthesis of axially chiral diaryl ethers (a–c). Cited yields are those of isolated products. Conditions **A**: **1** (0.05 mmol), **2** (0.10 mmol), [Rh(cod)_2_]BF_4_, (*S*)‐Difluorphos (0.005 mmol), and CH_2_Cl_2_/EtOH (1:4) (16.7 mL). Conditions **B**: **1** (0.05 mmol), **2** (0.10 mmol), [Rh(cod)_2_]BF_4_, (*S*)‐Difluorphos (0.005 mmol), and CH_2_Cl_2_ (16.7 mL). Conditions **C**: **1** (0.05 mmol), **2** (0.10 mmol), [Rh(cod)_2_]BF_4_, (*S*)‐BINAP (0.005 mmol), and CH_2_Cl_2_/EtOH (1:4) (16.7 mL). (a) **1** (0.10 mmol), **2** (0.10 mmol), [Rh(cod)_2_]BF_4_, (*S*)‐Segphos (0.010 mmol), and CH_2_Cl_2_ (2.0 mL) were used. (b) **1** (0.05 mmol), **2** (0.05 mmol), [Rh(cod)_2_]BF_4_, (*S*)‐Segphos (0.005 mmol), and CH_2_Cl_2_ (2.0 mL) were used. (c) Conditions **A**.

With the optimized reaction conditions in hand, we examined the substrate scope with respect to 1,6‐diynes **1** and alkynyl ethers **2** (Figure 2). For the alkynyl ethers, variation of the alkoxy substituent from methyl (**2b**) to ethyl (**2d**) or methoxymethyl (**2e**) had little effect on the reaction outcome, and the corresponding diaryl ethers **3ab**, **3ad**, and **3ae** were obtained in high yields (84%–89%) and excellent enantioselectivities (98%–99% *ee*). Changing the terminal substituent of the alkyne to ethyl (**2f**), *tert*‐butyl (**2g**), or an acetyl group (**2h**) led to a slight decrease in yield; however, the desired products **3af–3ah** were still obtained in good to high yields, ranging from 76% to 87%. The reaction was also applicable when the terminal alkyl group of the 1,6‐diyne was changed to an ethyl group (**3bb**). In contrast, the introduction of an ethyl ester or acetyl group at the carbonyl terminus of the diyne led to a substantial decrease in the product yield (**3cb** and **3db**). In these cases, the conversion of the corresponding diynes (**1c** and **1d**) was also diminished, which may be attributed to the reduced coordinating ability of the carbonyl group in these 1,6‐diynes. Ether (**1e**) and dimethyl malonate (**1f**)‐linked 1,6‐diynes were compatible, affording the desired products **3eb** and **3fb**, respectively, in good yields with high enantioselectivities.

For alkynyl ethers bearing an *ortho*‐methyl group, replacement of the *tert*‐butyl group (**1a**) with an adamantyl group (**1i**) led to a higher yield of the corresponding diaryl ether **3ai** than that of **3aa**. Incorporation of an electron‐deficient 3,5‐bis(trifluoromethyl)phenyl group into the 1,6‐diyne (**1g**) increased the product yield to 68% (**3ga**), although the enantioselectivity decreased to 71% *ee*. In contrast, symmetric 1,6‐diynes (**1h–1l**) reacted with **2a** to yield the corresponding diaryl ethers (**3ha–3la**) in moderate to high yields, while the enantioselectivities were moderate; a similar trend was observed with monoalkyne **2b**, producing **3hb**. This outcome is likely due to the absence of electronic and steric differentiation between substituents, rendering the transition state for the alkyne insertion step insufficiently biased. For 1,6‐diynes **1j** and **1k**, the product yields were moderate due to the incomplete conversion (61%) of **1j** and rapid homo‐[2 + 2 + 2] cycloaddition of **1k**.

For alkynyl ethers bearing *ortho*‐aryl substituents, alkynes bearing a methyl, methoxy, or trifluoromethyl group at the *para*‐position of the phenyl substituent all reacted with 1,6‐diyne **1a** to give the corresponding products (**3ac**, **3aj**, and **3ak**) with high enantioselectivity, albeit in varied yields. A 2‐methylphenyl group was also acceptable, but the yield of the product (**3al**) decreased. Notably, alkynes bearing sterically demanding *ortho*‐aryl substituents (**2** **k** and **2** **l**) gave substantially lower yields of the corresponding products (**3ak** and **3al**), likely due to increased steric hindrance at the *ortho* position, which impedes the insertion of the alkynyl ether. In contrast, alkynyl ether **2c** reacted smoothly with 1,6‐diynes bearing electron‐deficient 4‐nitrophenyl or 3,5‐bis(trifluoromethyl)phenyl groups, affording the corresponding products (**3mc** and **3gc**) in high yields. The absolute configuration of (+)‐**3gc** was determined to be the *S* configuration by x‐ray crystallographic analysis (Figure S10) [61].

**FIGURE 3:**
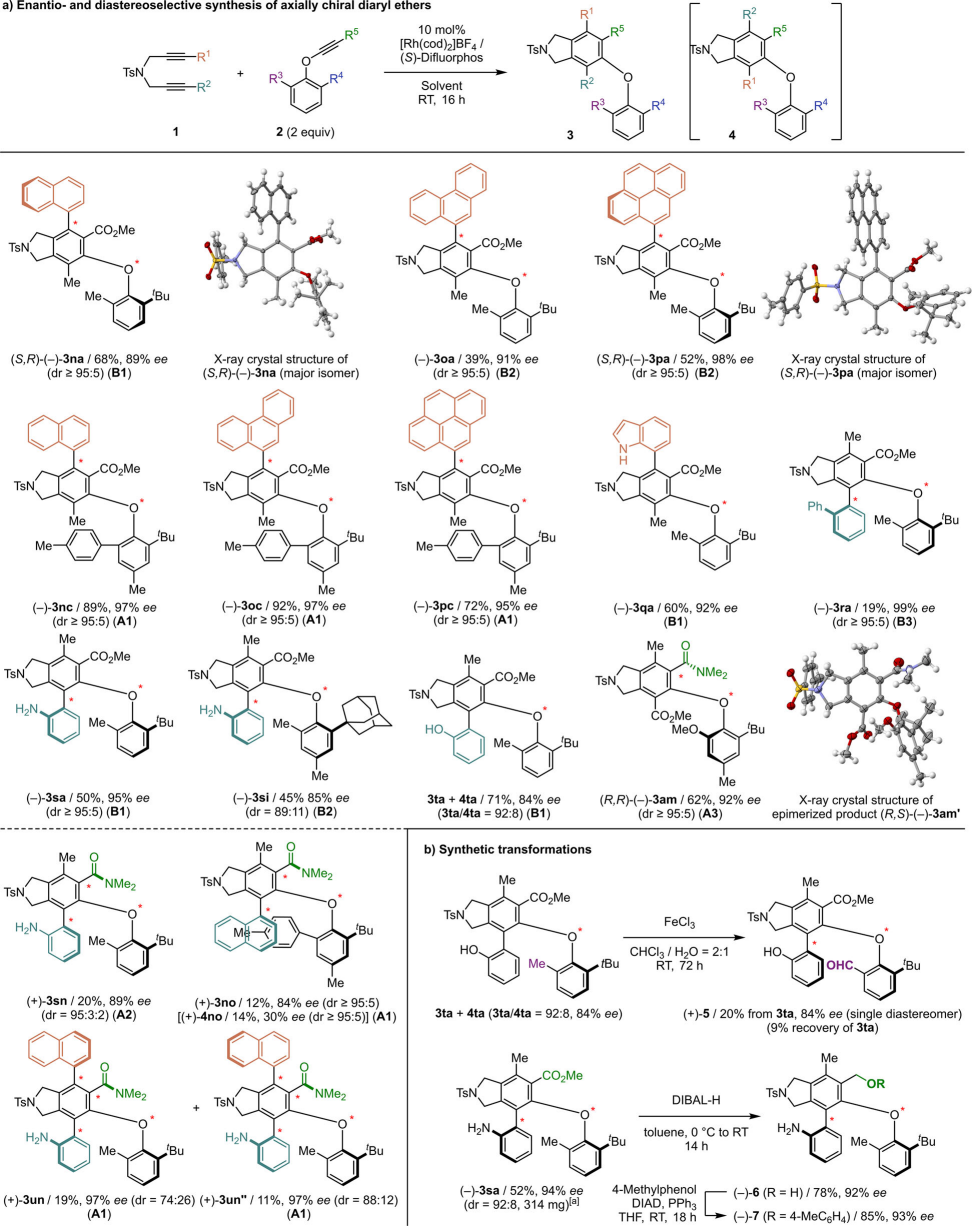
Enantio‐ and diastereoselective synthesis of axially chiral diaryl ethers. Cited yields are those of isolated products. Reaction conditions are shown in parentheses. Conditions **A1**: **1** (0.05 mmol), **2** (0.10 mmol), [Rh(cod)_2_]BF_4_, (*S*)‐Difluorphos (0.005 mmol), and CH_2_Cl_2_/EtOH (5:1) (1.2 mL). Conditions **A2**: **1** (0.05 mmol), **2** (0.10 mmol), [Rh(cod)_2_]BF_4_, (*S*)‐Difluorphos (0.005 mmol), and CH_2_Cl_2_/EtOH (5:1) (0.6 mL). Conditions **A3**: **1** (0.05 mmol), **2** (0.10 mmol), [Rh(cod)_2_]BF_4_, (*S*)‐Difluorphos (0.005 mmol), and CH_2_Cl_2_/EtOH (1:5) (16.7 mL). Conditions **B1**: **1** (0.10 mmol), **2** (0.20 mmol), [Rh(cod)_2_]BF_4_, (*S*)‐Difluorphos (0.010 mmol), and CH_2_Cl_2_ (1.0 mL). Conditions **B2**: **1** (0.050 mmol), **2** (0.10 mmol), [Rh(cod)_2_]BF_4_, (*S*)‐Difluorphos (0.005 mmol), and CH_2_Cl_2_ (1.0 mL). Conditions **B3**: **1** (0.050 mmol), **2** (0.10 mmol), [Rh(cod)_2_]BF_4_ (0.005 mmol), (*S*)‐Difluorphos (0.005 mmol), and CH_2_Cl_2_ (0.5 mL). (a) **1** (1.0 mmol), **2** (2.0 mmol), [Rh(cod)_2_]BF_4_, (*S*)‐Difluorphos (0.05 mmol), CH_2_Cl_2_ (10 mL), and 72 h were used. (b) Based on recovered starting material.

Next, we examined substrates designed to generate additional atropisomeric axes beyond the diaryl ether axis (Figure 3a). For each substrate, optimized solvents and concentrations were used with reaction conditions **A1–A3** in CH_2_Cl_2_/EtOH and **B1–B3** in CH_2_Cl_2_. 1,6‐Diyne **1l**, bearing a 1‐naphthyl group, reacted with **2a** in CH_2_Cl_2_ in the presence of a cationic rhodium(I)/Difluorphos catalyst to give product **3na**, incorporating both an axially chiral diaryl ether and an additional axially chiral biaryl unit at the *meta* position (R^1^), in good yield (68%) with high stereoselectivity (89% *ee*, dr ≥ 95:5). The absolute and relative configurations of (−)‐**3na** were determined to be the *S* and *R* configurations by x‐ray crystallographic analysis (Figure S11) [61]. Extension of the π‐system to 9‐phenanthrenyl and 4‐pyrenyl substituents likewise furnished the corresponding products **3oa** and **3pa** in moderate yields with high enantio‐ and diastereoselectivities. The absolute and relative configurations of (−)‐**3pa** were determined to be the *S* and *R* configurations by x‐ray crystallographic analysis (Figure S12) [61]. Furthermore, using alkynyl ether **2c**, bearing a 4‐methylphenyl group at the *ortho* position (R^3^), provided the corresponding diaryl ethers **3nc–3pc** in high yields (72%–92%) with excellent stereoselectivities (95%–97% *ee*, dr ≥ 95:5). 7‐Indolyl‐substituted diyne **1q** reacted with **2a** to afford **3qa** in good yield and enantioselectivity, although it was obtained as a mixture of diastereomers due to the relatively low rotational barrier of the biaryl axis.

Introduction of a 2‐phenylphenyl group at the terminus of the diyne (**1r**) afforded a single regioisomer, **3ra,** bearing an axially chiral biaryl unit at the *ortho* position (*R* ^2^) of the diaryl ether in high stereoselectivity (99% *ee*, dr ≥ 95:5), albeit in low yield. Similarly, diyne **1s**, which has a 2‐aminophenyl group, produced **3sa** and **3si** in moderate yields but high *ee* values. In contrast, product **3ta**, derived from a 2‐hydroxyphenyl‐substituted diyne **1t**, was obtained as a diastereomeric mixture due to the lower rotational barrier of the biaryl axis, although good yield and enantioselectivity were observed. Installation of a dimethylamide group at the terminus of the alkynyl ether enabled access to **3am**, bearing an axially chiral benzamide moiety at the *ortho* position (R^5^) of the diaryl ether, in good yield with high diastereoselectivity (≥ 95:5) and enantioselectivity (92% *ee*). During the crystallization study, (−)‐**3am** gradually underwent epimerization, with the amide moiety rotating to (−)‐**3am’**, and the absolute and relative configurations of (−)‐**3am’** were determined to be *R* and *S* configurations by x‐ray crystallographic analysis (Figure S13) [61].

Encouraged by these results, we next attempted the simultaneous introduction of three distinct axial chiral elements (diaryl ether, biaryl, and benzamide). The reaction of 2‐aminophenyl‐substituted diyne **1s** with dimethylamide‐substituted monoyne **2n** afforded **3sn**, possessing atropisomeric axes at the *ortho* positions (R^2^ and R^5^) of the diaryl ether, in 20% yield with high enantio‐ and diastereoselectivity. Similarly, 1‐naphthyl‐substituted diyne **1n** reacted with dimethylamide‐substituted monoyne **2o**, affording **3no** and **4no** in 12% and 14% yields with high diastereoselectivity (≥ 95:5). Although **3no** was obtained in a good *ee* value, that of **4no** was low. Finally, we examined the introduction of four atropisomeric axes on a single aromatic ring formed via the [2 + 2 + 2] cycloaddition. Products **3un** and **3sn''** were obtained in low yields but with high enantioselectivity and good diastereoselectivity. Although only limited examples were obtained, these results demonstrate the potential of this strategy for constructing complex atropisomeric architectures.

Subsequently, we investigated the derivatization of the axially chiral diaryl ethers (Figure 3b). A configurationally unstable diastereomer mixture of **3ta** underwent gradual oxidation of the R^3^ methyl group to the corresponding aldehyde **5** upon standing in CDCl_3_, affording benzaldehyde **5** diastereoselectively. Presumably via benzyl radical formation, treatment of **3ta** with FeCl_3_ in a CHCl_3_/H_2_O mixture at room temperature furnished **5** in 20% yield as a single diastereomer without racemization. The preparative‐scale reaction of **1s** (1.0 mmol) with **2a** (2.0 mmol) proceeded efficiently with the 5 mol% rhodium catalyst for 72 h, affording **3sa** in 52% yield (314 mg) with 94% *ee*. Subsequently, **3sa** was smoothly reduced with DIBAL‐H to convert the methyl ester into the corresponding benzyl alcohol **6** in good yield. The subsequent Mitsunobu reaction with 4‐methylphenol selectively functionalized the benzyl alcohol at the R^3^ position, not the aniline moiety, to give **7**, preserving the enantiomeric excess throughout this sequence of transformations.

**FIGURE 4:**
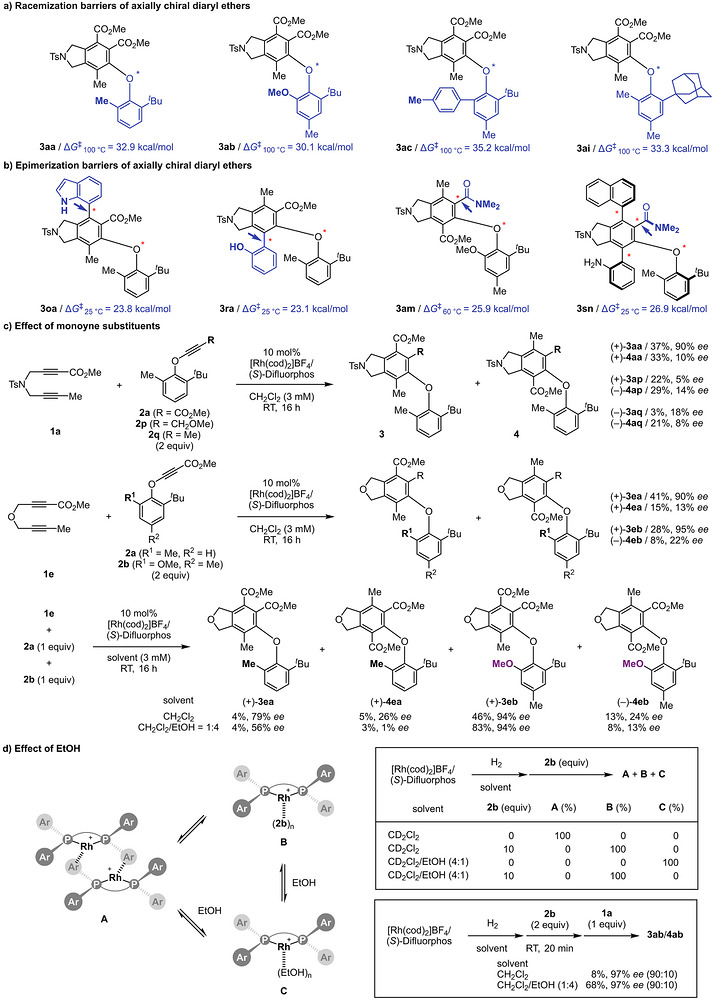
Configurational stability of axially chiral diaryl ethers (a and b) and experimental mechanistic studies (c and d). Cited yields are those of isolated products.

We evaluated the configurational stability of the axially chiral diaryl ethers by monitoring the change in *ee* values upon heating in toluene at 100°C, and the racemization barriers were determined from Arrhenius plots (Table S1, Figures S1–S4, and Figure 4a). Variation of one *ortho* substituent on the diaryl ether revealed a clear trend in the activation barriers: methoxy (**3ab**, 30.1 kcal/mol) < methyl (**3aa**, 32.9 kcal/mol) < 4‐methylphenyl (**3ac**, 35.2 kcal/mol). All values exceeded 30 kcal/mol, indicating that these compounds possess configurationally stable atropisomeric axes at ambient temperature. Replacement of the *tert*‐butyl group in **3aa** (32.9 kcal/mol) with an adamantyl group (**3ai**, 33.3 kcal/mol) resulted in no marked change in the rotational barrier.

Next, we investigated epimerization processes for molecules bearing multiple atropisomeric axes by monitoring the time‐dependent changes in their NMR spectra (Table S2, Figures S5–S8, and Figure S4b). The indole‐containing axially chiral biaryl derivative **3qa** underwent epimerization with a barrier of 23.8 kcal/mol. Among compounds featuring an axially chiral biaryl unit at the *ortho* position of the diaryl ether, **3sa** bearing a 2‐aminophenyl group exhibited almost no detectable epimerization at room temperature, whereas **3ta** bearing a 2‐hydroxyphenyl group epimerized with a relatively low barrier of 23.1 kcal/mol. The axially chiral benzamide **3am** gradually epimerized upon heating to 60°C, corresponding to a rotational barrier of 25.9 kcal/mol. Furthermore, **3un** gradually underwent epimerization of the atropisomeric benzamide at room temperature with a barrier of 26.9 kcal/mol. In contrast, no epimerization was observed for diastereomer **3un*''*** under the same conditions, indicating that the steric environment created by the adjacent axially chirality, the *tert*‐butyl group of the 2‐methyl‐6‐*tert*‐butylphenoxy moiety, and the m‐region of the 1‐naphthyl group more effectively suppresses rotation of the chiral axis.

To gain mechanistic insight, the effect of the terminal substituent R on the alkynyl ether **2** was examined (Figure 4c). Changing the R group from an ester (**2a**) to a methoxymethyl group (**2p**) resulted in a slight decrease in yield and, notably, a dramatic reduction in the *ee* value for compound **3ap**. When *R* was a methyl group (**2q**), the yield and enantioselectivity of **3aq** decreased substantially. These results indicate that the carbonyl group at the terminal position of the alkynyl ether **2** plays a dual role: it enhances reactivity through strong coordination to the rhodium center and conformational fixation of the substrate, and it modulates the electronic properties of the alkyne as an electron‐withdrawing substituent.

The substituent effect at the *ortho* position (R^3^) of the alkynyl ether **2** was further examined through the [2 + 2 + 2] cycloaddition with diyne **1e** under identical conditions. Although the methyl‐substituted alkyne (**2a**) afforded higher combined yields of the two regioisomers (**3ea**/**4ea**) than those (**3eb**/**4eb**) derived from the methoxy‐substituted alkyne (**2b**), both enantioselectivity and regioselectivity were improved for **3eb** bearing the methoxy group. In a competition experiment using equimolar amounts of alkynyl ethers **2a** and **2b** with diyne **1e**, the product derived from **2b** was preferentially formed in CH_2_Cl_2_, and the yield of **3eb** was markedly enhanced in a CH_2_Cl_2_/EtOH mixed solvent. These findings indicate that the *ortho*‐methoxy group contributes to both regio‐ and enantioselectivity, promoting the pathway leading to **3eb**.

Finally, to clarify the solvent effect observed with methoxy‐substituted alkynyl ether **2b**, the initially formed rhodium complexes were analyzed (Figure S9 and Figure 4d). In CH_2_Cl_2_, the cationic rhodium(I)/bisphosphine complex adopts an arene‐coordinated dimeric structure **A** [62, 63]. Upon addition of 10 equivalents of **2b**, the ^31^P NMR signal shifted upfield to 27.8 ppm, consistent with the formation of the alkynyl ether–coordinated complex **B**. In contrast, the addition of ethanol resulted in a shift to 52.5 ppm, indicating the formation of the highly active alcohol‐coordinated complex **C** [64]. When **2b** (10 equiv) was added in CH_2_Cl_2_/EtOH, only complex **B** was observed, and complex **C** was not detected.

Assuming that complex **B** represents the resting state, we examined its ability to catalyze reactions. In CH_2_Cl_2_, the reaction proceeded only sluggishly to give **3ab** in 8% yield. In CH_2_Cl_2_/EtOH, however, the yield increased to 68%, while enantio‐ and regioselectivities remained essentially unchanged. Simultaneous addition of diyne **1a** and alkynyl ether **2b** further increased the yield of **3ab** to 89% (Figure 2a), presumably by decreasing the proportion of complex **B**. Collectively, these results suggest that EtOH facilitates initiation of the catalytic cycle by generating a small amount of EtOH‐coordinated complex **C** from complex **B**.

**FIGURE 5:**
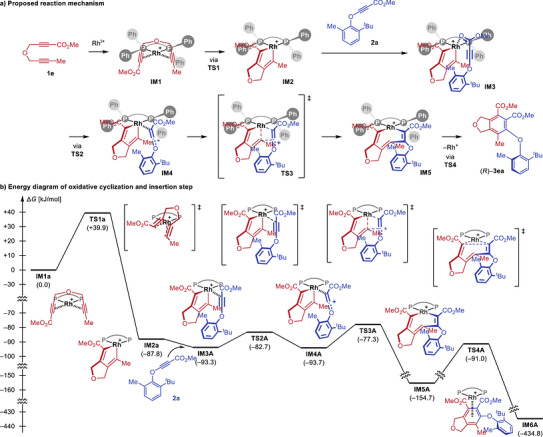
Proposed reaction mechanism (a) and DFT‐calculated energy diagram for the oxidative cyclization and insertion steps, calculated at the B3LYP‐D3/Def2‐TZVPPD/IEFPCM(DCM)//B3LYP‐D3/Def2‐SVP/IEFPCM(DCM) level of theory (b).

To elucidate the mechanism of enantioselective axial chirality control, DFT calculations and AFIR facilitated [65] conformational searches were performed using diyne **1e** and alkynyl ether **2a**. The mechanism of cationic Rh(I)‐catalyzed [2 + 2 + 2] cycloaddition is established in previous studies [50, 66, 67, 68, 69, 70]. Diyne **1e** first coordinates to rhodium (Figure 5a, **IM1**), followed by oxidative cyclization (**TS1**) to form a rhodacyclopentadiene (**IM2**). Alkyne insertion then proceeds stepwise, distinct from concerted mechanisms. Carbonyl coordination of alkynyl ether **2a** (**IM3**) and Rh–C bond formation (**TS2**) generate a cationic ketene intermediate (**IM4**), followed by C–C bond formation (**TS3**) to give a cycloheptatriene intermediate (**IM5**). Reductive elimination affords diaryl ether **3ea**. The computed free energy profile (Figure 5b) shows that all transition states (**TS1a**, **TS2A**, **TS3A**, and **TS4A**) are energetically accessible (10.6–63.7 kJ/mol). The stereochemistry of **3ea** is determined at C–C bond formation (**TS3**), whereas the conformation is set during oxidative cyclization (**TS1**). Accordingly, we performed detailed calculations by generating conformers of these transition states using the SC‐AFIR method at the GFN‐xTB level [71], followed by DFT optimization of the low‐energy structures.

**FIGURE 6:**
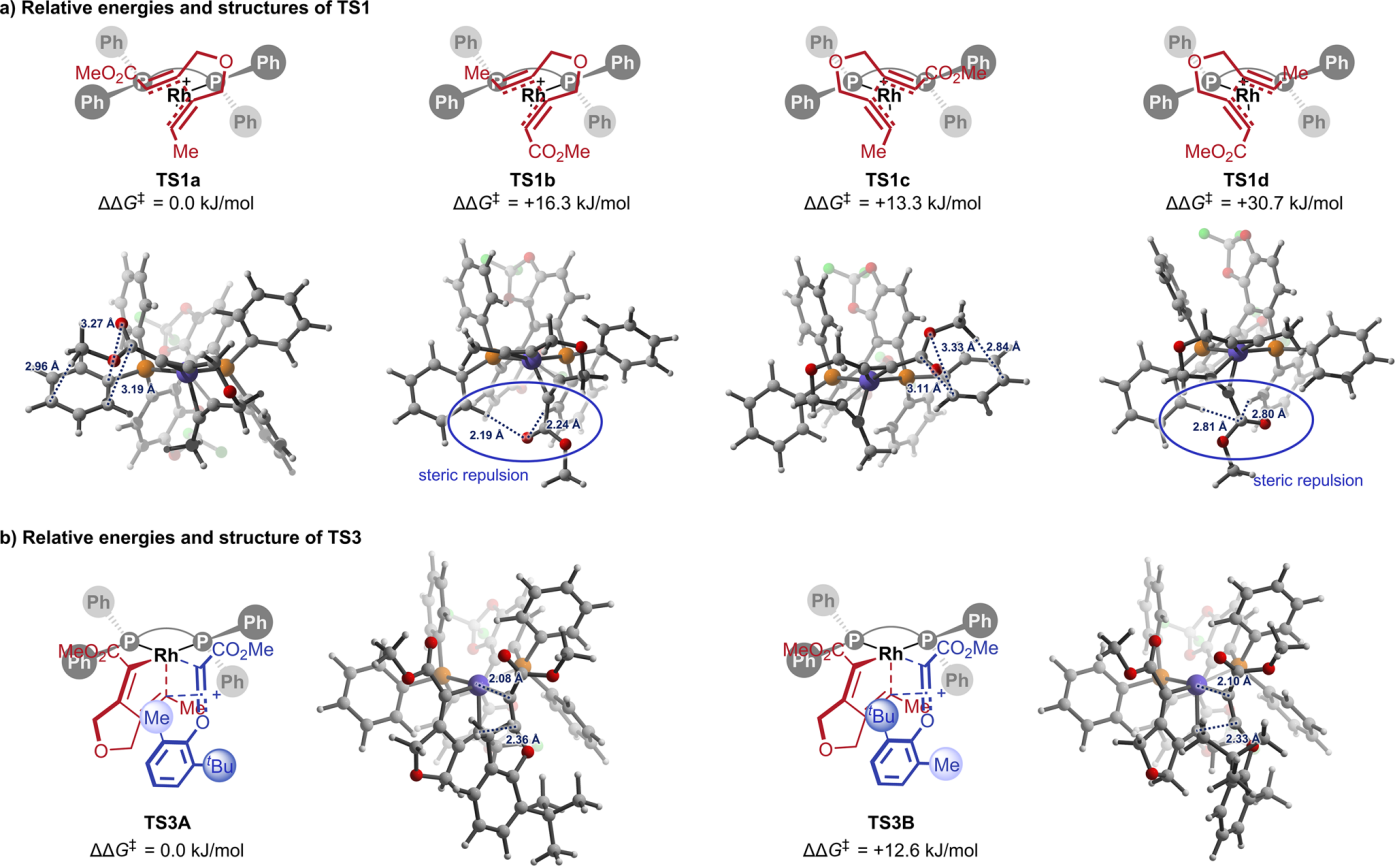
DFT‐calculated relative energies and structures of **TS1** (a) and the relative energies and structures of **TS3** (b).

For the oxidative cyclization step, four conformations (**IM2a–IM2d**, Figure S14) are conceivable, depending on the terminal substituent orientation of the diyne and the relative arrangement of the rhodacyclopentadiene and the bisphosphine ligand. Compared to the lowest‐energy transition state **TS1a**, **TS1b** (ΔΔ*G* ^‡^ = +16.3 kJ/mol), **TS1c** (ΔΔ*G* ^‡^ = +13.3 kJ/mol), and **TS1d** (ΔΔ*G* ^‡^ = +30.7 kJ/mol) are destabilized, indicating that the reaction proceeds selectively via **TS1a** (Figure 6a and Figure S15a). Distortion–interaction analysis [72], based on fragmentation into the diyne and the rhodium bisphosphine complex, revealed that the diyne distortion energy (Δ*E*_dis_diyne_) is smaller for the lower‐energy transition states **TS1a** and **TS1c** (Figure S15b). From structural inspections of **TS1b** and **TS1d**, with the terminal ester positioned along a pseudo‐axial direction, steric repulsion between the ester and phenyl groups on the ligand causes large distortion (Table S8). Dispersion interaction between the carbonyl group of the diyne ester and the phenyl groups on phosphorus stabilizes **TS1a** and **TS1c**; NCI and IGMH analyses [73, 74] confirm extensive non‐covalent interactions (Figure S15d), and energy decomposition analysis using the sobEDA program [75, 76] shows large dispersion contributions (Δ*E*_dc_) in both **TS1a** and **TS1c** (Figure S15c). In both **TS1a** and **TS1c**, the terminal ester approaches the phenyl group; however, the shorter interfragment distances in **TS1c** increase overlap and distortion, making **TS1a** more favorable. Interconversion barriers of **IM2a–IM2d** (Figure S16) exceed those of the subsequent insertion step (Figure 5b and Figure S17).

Previous studies describe the insertion step as a concerted process involving the formation of two bonds and the cleavage of one bond. However, the lowest‐energy pathway involves carbonyl coordination of **2a** to rhodium (**IM3**), followed by Rh–C bond formation (**IM4**) and C–C bond formation to give cycloheptatriene intermediate **IM5** (Figure 5b). Natural population analysis (NPA) charges indicate that the rhodium center is positively charged in **IM3**, whereas in **IM4**, the ether oxygen and the adjacent alkyne carbon bear positive charge, while the alkyne carbon coordinated to rhodium is negatively charged (Tables S9 and S10). Hirshfeld charge analysis also shows pronounced polarization of the alkyne carbons (Tables S9 and S10). These results are consistent with the polarized limiting structure of push‐pull alkynes.

Two transition states are conceivable for the insertion step, depending on the phenoxy conformation of **2a** that defines the axial chirality. **IM3A** (–93.3 kJ/mol) and **IM4A** (–93.7 kJ/mol) are nearly isoenergetic, with the intervening transition state **TS2A** (−82.7 kJ/mol) being low in energy (Figure 5b), indicating that interconversion between them is reversible. Similar behavior was found for the alternative conformer (Figure S17). In contrast, the insertion transition state **TS3** (–77.3 kJ/mol) is higher in energy, thereby determining the product stereochemistry (Figure 5b). **TS3B** is destabilized by ΔΔ*G* ^‡^ = +12.6 kJ/mol relative to **TS3A**, consistent with the experimentally observed 90% *ee* (Figure 6b). In **TS3**, the dihydrofuran moiety formed from the diyne approaches the *ortho* substituents (methyl and *tert*‐butyl groups) of **2a**. The transition state **TS3A**, in which the smaller methyl group contacts the furan moiety, gives a shorter Rh–C distance (2.08 Å) than **TS3B** (2.10 Å). Distortion–interaction analysis, separating the cyclopentadiene complex and **2a**, shows stronger stabilization in **TS3A** (Δ*E*_int_ = –227.4 kJ/mol) than **TS3B** (Δ*E*_int_ = –216.9 kJ/mol) (Figure S18b). Energy decomposition analysis using sobEDA [75] indicates that **TS3A** is more stabilized than **TS3b**, with more favorable electrostatic (ΔΔ*E*_els_ = –7.9 kJ/mol) and orbital (ΔΔ*E*_orb_ = –16.9 kJ/mol) interactions. However, **TS3A** shows a less favorable exchange‐repulsion term (ΔΔ*E*_xrep_ = +8.4 kJ/mol) due to increased Pauli repulsion from the shorter Rh–C distance, which is balanced by the enhanced electrostatic and orbital stabilization (Figure S18c).

Transition states in which the phenoxy group is oriented approximately perpendicular to the rhodacyclopentadiene plane were also identified as higher‐energy conformers (**TS3A‐V**: ΔΔ*G* ^‡^ = +21.2 kJ/mol; **TS3B‐V**: ΔΔ*G* ^‡^ = +18.7 kJ/mol) (Figure S18a). Although alkyne coordination transition states (**TS2**) are located for these conformers, no corresponding insertion transition states could be identified. In these structures, the steric proximity between the phenoxy group and the phenyl group on the ligand prevents relief of steric repulsion, resulting in a smaller energy difference between the diastereomeric transition states (Figure S18d). Energy decomposition analysis shows reduced interaction energy (Δ*E*_int_), and NCI / IGMH analysis reveals the diminished interaction region between the rhodacyclopentadiene moiety and the phenoxy ring in **TS3A‐V**/**TS3B‐V** (Figure S18c,d). The shorter Rh–C bonds in the stepwise insertion TSs (**TS3A**: 2.08 Å, **TS3B**: 2.10 Å) versus the concerted TSs (**TS3A‐V**: 2.16 Å, **TS3B‐V**: 2.14 Å) create a compact transition state, likely enabling efficient transfer of the complex's chiral environment to the forming axial chirality and thus accounting for the observed high enantioselectivity (Figure S18d).

Collectively, these results indicate that strong coordination of the carbonyl group and the polarized push–pull alkyne to the rhodium center fixes the substrate conformation. The steric repulsion with the ligand stabilizes a conformation wherein the rhodacyclopentadiene plane formed during oxidative cyclization positions the phenoxy substituents in close proximity. As a result, the steric information of the phenoxy substituents, distal from the catalytic center, is effectively recognized, enabling highly enantioselective synthesis of axially chiral diaryl ethers.

## 3 Conclusion

We have developed a rhodium‐catalyzed enantioselective [2 + 2 + 2] cycloaddition that enables efficient synthesis of axially chiral diaryl ethers. Benzannulation simultaneously constructs the aromatic ring and restricts rotation about the Ar–O bond, providing a general strategy for atroposelective synthesis. The introduction of a coordinating terminal carbonyl group proved crucial for high reactivity, regioselectivity, and stereocontrol, allowing access to products with bulky ortho substituents and, in suitably designed substrates, multiple atropisomeric axes with up to four stereogenic axes. DFT calculations reveal that a stepwise alkyne insertion, featuring shortened Rh–C bonds in the transition state, likely enables efficient transfer of the chiral environment to the forming axis, accounting for the observed high enantioselectivity. These findings provide insight into controlling flexible axial chiralities and highlight the potential of benzannulation‐based strategies for constructing structurally diverse and configurationally labile atropisomers.

## Author Contributions

**Yu Sato**: conceptualization, methodology, data curation, investigation, validation, formal analysis, visualization, writing – original draft, writing – review and editing. **Julong Jiang**: data curation, investigation, validation, formal analysis, supervision, visualization, resources, writing – original draft, writing – review and editing. **Kairi Yamashiro**: investigation, validation. **Ken Tanaka**: conceptualization, methodology, data curation, investigation, validation, formal analysis, supervision, funding acquisition, visualization, project administration, resources, writing – original draft, writing – review and editing.

## Conflicts of Interest

The authors declare no conflicts of interest.

## Supporting information




**Supporting File 1**: anie72956‐sup‐0001‐SuppMat.zip.

**Supporting File 2**: anie72956‐sup‐0002‐SuppMat.xyz.

## Data Availability

The data that supports the findings of this study are available in the supplementary material of this article.
