# Supplementary material for: Enantioselective Synthesis of Axially Chiral Diaryl Ethers via Rhodium‐Catalyzed [2 + 2 + 2] Cycloaddition
Source: Angew Chem Int Ed Engl. 2026 Jun 3;65(32):e3486845. doi: 10.1002/anie.3486845 (PMC13427224; doi:10.1002/anie.3486845)
Supplement: Supplementary file 1 — Supporting File 1: anie72956‐sup‐0001‐SuppMat.zip. [file ANIE-65-e3486845-s002.zip › anie72956-sup-0001-SuppMat/2026.5.30-revised-SI(Chiral-Diaryl-Ether).pdf]

Supporting Information

Table of Contents

1. General Information **S2**
1.1. General Experimental Information S2
1.2. General Analytical Information S2

2. Synthetic Experiments **S3**
2.1. Synthesis of Substrates S3
2.1.1. Synthesis of 1,6-Diynes S3
2.1.2. Synthesis of Alkynyl Ethers S9
2.2. Rh-Catalyzed Enantioselective Synthesis of Axially Chiral Diaryl Ethers S19
2.3. Rh-Catalyzed Enantio- and Diastereoselective Synthesis of Axially Chiral Diaryl Ethers S37
2.4. Synthetic Transformations S49
2.4.1. Oxidation S49
2.4.2. Reduction S49
2.4.3. Mitsunobu Reaction S50
2.5. Stability of Axial Chirality S51
2.5.1. Racemization S51
2.5.2. Epimerization S53
2.6. Experimental Mechanistic Studies S56
2.6.1. Effect of Terminal Substituent on Monoyne S56
2.6.2. Effect of *ortho*-Substituent on Monoyne S58
2.6.3. Competition Experiments S59
2.6.4. Observation of Rh Complexes in Various Solvents by NMR S60
2.6.5. Reaction via in-situ Generation of Rh-Monoyne Complex S61

3. Single-Crystal X-Ray Diffraction Analysis **S62**

4. Theoretical Calculations **S71**
4.1. Computational Methods S71
4.2. Computational Energies of All Optimized Structures S72
4.3. Computational Studies for Reaction Pathways S73

5. References **S79**

6. ^{1}H, ^{13}C, and ^{19}F NMR Spectra **S83**

7. Chiral HPLC Charts **S244–S300**

1. General Information

1.1. General Experimental Information

Anhydrous and degassed CH_2Cl_2 (No. 041-32345, Wako) was used as received. Other solvents used in this work were dried over molecular sieves 4Å or 3Å (Wako) before use.

$[Rh(cod)_2]BF_4$ was synthesized from $[RhCl(cod)]_2$ according to the published literature.[1] (*S*)-H_8-BINAP, (*S*)-Segphos, (*S*)-tol-Segphos, and (*S*)-xyl-Segphos were obtained from Takasago International Corporation. (*S*)-Difluorphos was obtained from Strem Catalog and Angene Chemical. All other reagents were purchased from TCI Chemicals, Wako Pure Chemical Industries, Sigma-Aldrich, and Kanto Chemicals and used as received.

Silica gel column chromatography was performed using silica gel [Silica Gel 60 N (spherical, neutral), Kanto Chemicals] and JIS (Japanese Industrial Standards) special grade solvents. Silica gel preparative thin layer chromatography (PTLC) was performed using silica gel (Wakogel® B-5F) and JIS special grade solvents.

All reactions were carried out under an atmosphere of argon (Ar) or nitrogen (N_2) in oven-dried glassware with magnetic stirring.

1.2. General Analytical Information

^{1}H, ^{13}C, ^{19}F, and ^{31}P NMR data were collected on Bruker AVANCE III HD 400 at ambient temperature. All ^{1}H NMR experiments are reported in δ units, parts per million (ppm), and were measured relative to the signal for residual chloroform (7.26 ppm). All ^{13}C NMR spectra are reported in ppm relative to deuterochloroform (77.16 ppm), and were obtained with ^{1}H decoupling. All ^{19}F NMR spectra are reported in ppm relative to benzotrifluoride (–64.0 ppm), and were obtained with ^{1}H decoupling. HRMS data were obtained on a Bruker micrOTOF Focus II or JEOL JMS-T2000GC. Melting points were determined on a Mettler MP30.

Chiral HPLC analyses were carried out on a Jasco LC-2000Plus Series system using Daicel CHIRALPAK® columns (internal diameter 4.6 mm, column length 250 mm, and particle size 3 or 5 μm). Optical rotation data were obtained on a Jasco P-2200 digital polarimeter with the sodium D line (589 nm) at ambient temperature.

2. Synthetic Experiments

2.1. Synthesis of Substrates

Substrates **1a**,[2] **S1b**,[3] **S1c**,[4] **1e**,[5] **1f**,[6] **1h**,[7] **1i**,[8] **1j**,[9] **S1k**,[10] **1s**,[11] **1t**,[11] **S2b**,[12] **S2c**,[13] **S4d**,[12] **S2i**,[14] **S2j**,[15] **S2k**,[13] and **S5l**,[16] were synthesized according to the literature.

2.1.1. Synthesis of 1,6-Diynes

Methyl 4-((4-methyl-*N*-(pent-2-yn-1-yl)phenyl)sulfonamido)but-2-ynoate (1b)

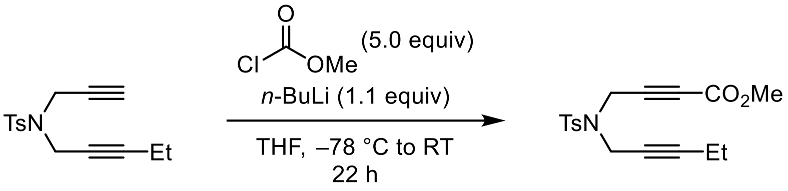

S1b **1b**

To a solution of 4-methyl-*N*-(pent-2-yn-1-yl)-*N*-(prop-2-yn-1-yl)benzenesulfonamide (**S1b**[3], 0.551 g, 2.00 mmol) in THF (15 mL) was added dropwise a 1.51 M solution of *n*-BuLi in hexane (1.46 mL, 2.20 mmol of *n*-BuLi) at −78 °C. After stirring at −78 °C for 0.5 h, methyl chloroformate (0.945 g, 10.0 mmol) was added, and the mixture was warmed to room temperature. The resulting mixture was stirred at room temperature for 22 h. The reaction was quenched by the addition of phosphate buffer solution [NaH_2PO_4 (1.36 g, 10.0 mmol) and (Na_2HPO_4 1.74 g, 10.0 mmol) in water 100 mL], and organic layer was immediately separated and dried over Na_2SO_4. The aqueous layer was extracted with Et_2O, and combined organic layer was dried over Na_2SO_4 and concentrated. The residue was purified on silica gel column chromatography (*n*-hexane/EtOAc = 3:1), which furnished **1b** (0.359 g, 1.08 mmol, 54% yield).

Yellow oil; ^{1}H NMR (400 MHz, $CDCl_3$) δ 7.72 (d, *J* = 8.3 Hz, 2H), 7.31 (d, *J* = 8.0 Hz, 2H), 4.28 (s, 2H), 4.10 (t, *J* = 2.1 Hz, 2H), 3.73 (s, 3H), 2.42 (s, 3H), 2.06–1.99 (m, 2H), 0.98 (t, *J* = 7.5 Hz, 3H); ^{13}C NMR (101 MHz, $CDCl_3$) δ 153.1, 144.1, 135.0, 129.6, 127.9, 88.5, 80.7, 76.8, 71.1, 52.7, 37.4, 36.2, 21.5, 13.4, 12.2; HRMS (FD) calcd for $C_{17}H_{19}NO_4S$ $[M]^+$ 333.1035, found 333.1016.

Ethyl 4-((*N*-(but-2-yn-1-yl)-4-methylphenyl)sulfonamido)but-2-ynoate (1c)

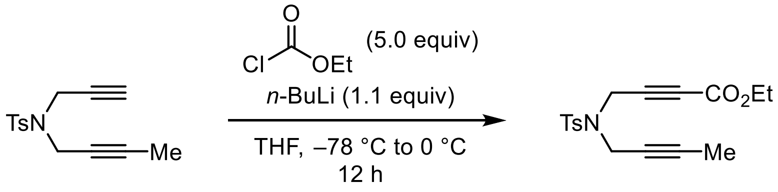

S1c **1c**

To a solution of *N*-(but-2-yn-1-yl)-4-methyl-*N*-(prop-2-yn-1-yl)benzenesulfonamide (**S1c**,[4] 0.522 g, 2.00 mmol) in THF (15 mL) was added dropwise a 1.51 M solution of *n*-BuLi in hexane (1.39 mL, 2.10 mmol of *n*-BuLi) at −78 °C. After stirring at −78 °C for 0.5 h, ethyl chloroformate (1.085 g, 10.0 mmol) was added, and the mixture was warmed to 0 °C. The resulting mixture was stirred at 0 °C for 12 h. The reaction was quenched by the addition of phosphate buffer solution [NaH_2PO_4 (1.36 g, 10.0 mmol) and Na_2HPO_4 (1.74 g, 10.00 mmol) in water 100 mL], and organic layer was immediately separated and dried over Na_2SO_4. The aqueous layer was extracted with Et_2O, and combined organic layer was dried over Na_2SO_4 and concentrated. The residue was purified by silica gel column chromatography (*n*-hexane/EtOAc = 4:1), which furnished **1c** (0.203 g, 0.609 mmol, 20% yield).

Yellow oil; ^{1}H NMR (400 MHz, $CDCl_3$) δ 7.72 (d, *J* = 8.3 Hz, 2H), 7.31 (d, *J* = 8.0 Hz, 2H), 4.28 (s, 2H), 4.18 (q, *J* = 7.1 Hz, 2H), 4.08 (q, *J* = 2.3 Hz, 2H), 2.42 (s, 3H), 1.66 (t, *J* = 2.4 Hz, 3H), 1.28 (t, *J* = 7.1 Hz, 3H) ^{13}C NMR (101 MHz, $CDCl_3$) δ 152.7, 144.1, 135.0, 129.6, 127.9, 82.7, 80.2, 77.2,

71.0, 62.1, 37.4, 36.2, 21.5, 14.0, 3.4; HRMS (FD) calcd for $C_{17}H_{19}NO_4S$ $[M+H]^+$ 334.1108, found 334.1116.

N-(But-2-yn-1-yl)-4-methyl-*N*-(4-oxopent-2-yn-1-yl)benzenesulfonamide (1d)

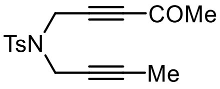

1d was prepared from *N*-(but-2-yn-1-yl)-4-methyl-*N*-(prop-2-yn-1-yl)benzenesulfonamide (**S1c,**[4] 0.521 g, 2.00 mmol) and acetyl chloride (0.785 g, 10.0 mmol) according to the procedure for **1c**. The final residue was purified by silica gel column chromatography (*n*-hexane/EtOAc = 4:1), which furnished **1d** (0.123 g, 0.406 mmol, 20% yield).

Brown oil; ^{1}H NMR (400 MHz, $CDCl_3$) δ 7.72 (d, *J* = 8.3 Hz, 2H), 7.32 (d, *J* = 8.0 Hz, 2H), 4.32 (s, 2H), 4.08 (q, *J* = 2.3 Hz, 2H), 2.42 (s, 3H), 2.18 (s, 3H), 1.67 (t, *J* = 2.4 Hz, 3H); ^{13}C NMR (101 MHz, $CDCl_3$) δ 183.4, 144.1, 135.1, 129.6, 128.0, 84.6, 84.3, 82.7, 71.0, 37.4, 36.3, 32.4, 21.5, 3.4; HRMS (FD) calcd for $C_{16}H_{17}NO_3S$ $[M+Na]^+$ 303.0929, found 303.0935.

N-(3-(3,5-Bis(trifluoromethyl)phenyl)prop-2-yn-1-yl)-*N*-(but-2-yn-1-yl)-4-methylbenzenesulfonamide (1g)

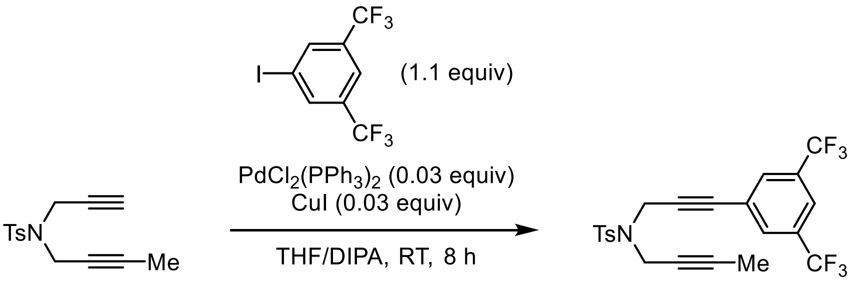

S1c **1g**

A solution of *N*-(but-2-yn-1-yl)-4-methyl-*N*-(prop-2-yn-1-yl)benzenesulfonamide (**S1c,**[4] 1.305 g, 5.00 mmol), 1-iodo-3,5-bis(trifluoromethyl)benzene (2.040 g, 6.00 mmol), $PdCl_2(PPh_3)_2$ (105.3 mg, 0.150 mmol), and CuI (28.6 mg, 0.150 mmol) in degassed THF/diisopropylamine(DIPA) (1:1, 50 mL) was stirred at room temperature for 14 h. The reaction mixture was filtered with Celite® and concentrated. The residue was purified by silica gel column chromatography (*n*-hexane/EtOAc = 95:5), which furnished **1g** (1.284 g, 2.71 mmol, 57% yield).

Pale brown solid; mp 109.0–110.3 °C; ^{1}H NMR (400 MHz, $CDCl_3$) δ 7.78 (s, 1H), 7.77 (d, *J* = 8.3 Hz, 2H), 7.58 (s, 2H), 7.29 (d, *J* = 8.0 Hz, 2H), 4.43 (s, 2H), 4.13 (q, *J* = 2.3 Hz, 2H), 2.35 (s, 3H), 1.71 (t, *J* = 2.4 Hz, 3H); ^{13}C NMR (101 MHz, $CDCl_3$) δ 144.0, 135.6, 131.90 (q, *J* = 33.7 Hz), 131.47 (dd, *J* = 1.0, 3.7 Hz), 129.5, 128.1, 124.6, 122.83 (q, *J* = 272.9 Hz), 121.89 (q, *J* = 3.8 Hz), 85.8, 82.5, 82.3, 71.4, 37.4, 36.9, 21.3, 3.5; HRMS (ESI) calcd for $C_{22}H_{17}F_6NO_2S$ $[M+Na]^+$ 496.0782, found 496.0770.

N-(But-2-yn-1-yl)-4-methyl-*N*-(3-(4-nitrophenyl)prop-2-yn-1-yl)benzenesulfonamide (1m)

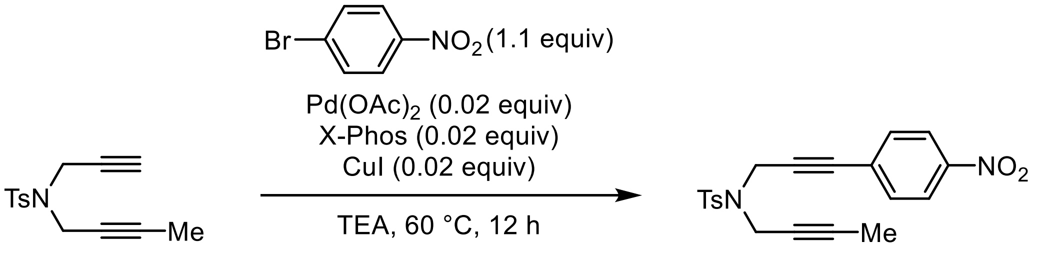

S1c **1m**

A solution of *N*-(but-2-yn-1-yl)-4-methyl-*N*-(prop-2-yn-1-yl)benzenesulfonamide (**S1c,**[4] 0.524 g, 2.00 mmol), 1-bromo-4-nitrobenzene (0.444 g, 2.20 mmol), $Pd(OAc)_2$ (9.0 mg, 0.040 mmol), X-Phos (38.1 mg, 0.080 mmol), and CuI (7.6 mg, 0.040 mmol) in degassed triethylamine (TEA) was stirred at 60 °C for 12 h. The reaction mixture was filtered with Celite® and concentrated. The residue was purified by silica gel column chromatography (*n*-hexane/CH_2Cl_2 = 1:4), which furnished **1m** (0.239 g, 0.624 mmol, 31% yield).

Pale brown solid; mp 94.3–96.0 °C; ^{1}H NMR (400 MHz, $CDCl_3$) δ 8.13 (d, *J* = 8.9 Hz, 2H), 7.76 (d, *J* = 8.3 Hz, 2H), 7.33 (d, *J* = 8.9 Hz, 2H), 7.28 (d, *J* = 8.0 Hz, 2H), 4.41 (s, 2H), 4.14 (q, *J* = 2.3 Hz, 2H), 2.38 (s, 3H), 1.69 (t, *J* = 2.4 Hz, 3H); ^{13}C NMR (101 MHz, $CDCl_3$) δ 147.2, 143.8, 135.5, 132.4, 129.5, 129.1, 128.0, 123.4, 87.6, 83.6, 82.3, 71.4, 37.3, 37.0, 21.5, 3.5; HRMS (FD) calcd for $C_{20}H_{18}N_2O_4S$ $[M]^+$ 382.0987, found 382.0992.

_N_-(But-2-yn-1-yl)-4-methyl-_N_-(3-(naphthalen-1-yl)prop-2-yn-1-yl)benzenesulfonamide (1n)

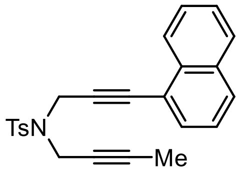

1n was prepared from *N*-(but-2-yn-1-yl)-4-methyl-*N*-(prop-2-yn-1-yl)benzenesulfonamide (**S1c,**[4] 0.134 g, 0.513 mmol) and 1-iodonaphthalene (0.280 g, 1.10 mmol) according to the procedure for **1g**. The final residue was purified by silica gel column chromatography (*n*-hexane/EtOAc = 4:1), which furnished **1n** (0.149 g, 0.385 mmol, 75% yield).

Pale brown solid; mp 64.1–66.1 °C; ^{1}H NMR (400 MHz, $CDCl_3$) δ 8.00–7.97 (m, 1H), 7.83–7.77 (m, 4H), 7.52–7.46 (m, 2H), 7.41 (dd, *J* = 1.3, 7.1 Hz, 1H), 7.36 (dd, *J* = 7.6, 7.6 Hz, 1H), 7.19 (d, *J* = 8.0 Hz, 2H), 4.55 (s, 2H), 4.22 (q, *J* = 2.3 Hz, 2H), 2.22 (s, 3H), 1.71 (t, *J* = 2.4 Hz, 3H); ^{13}C NMR (101 MHz, $CDCl_3$) δ 143.7, 135.5, 133.13, 133.06, 130.7, 129.5, 128.9, 128.2, 128.0, 126.8, 126.4, 126.0, 125.0, 120.0, 86.6, 83.8, 82.0, 71.7, 37.4, 37.2, 21.4, 3.5; HRMS (FD) calcd for $C_{24}H_{21}NO_2S$ $[M]^+$ 387.1293, found 387.1298.

_N_-(But-2-yn-1-yl)-4-methyl-_N_-(3-(phenanthren-9-yl)prop-2-yn-1-yl)benzenesulfonamide (1o)

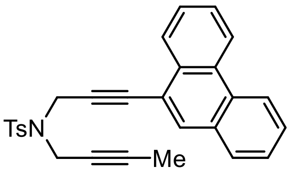

1o was prepared from *N*-(but-2-yn-1-yl)-4-methyl-*N*-(prop-2-yn-1-yl)benzenesulfonamide (**S1c,**[4] 0.262 g, 1.00 mmol) and 9-bromophenanthrene (0.283 g, 1.10 mmol) according to the procedure for **1m**. The final residue was purified by silica gel column chromatography (*n*-hexane/CH_2Cl_2 = 10:1), which furnished **1o** (0.209 g, 0.477 mmol, 48% yield).

Brown solid; mp 125.1–126.1 °C; ^{1}H NMR (400 MHz, $CDCl_3$) δ 8.65 (dd, *J* = 8.6, 8.6 Hz, 2H), 8.09 (dd, *J* = 1.1, 8.1 Hz, 1H), 7.80 (d, *J* = 8.3 Hz, 2H), 7.80 (d, *J* = 8.7 Hz, 1H), 7.72 (s, 1H), 7.70–7.64 (m, 2H), 7.61–7.58 (m, 2H), 7.20 (d, *J* = 8.1 Hz, 2H), 4.58 (s, 2H), 4.25 (q, *J* = 2.3 Hz, 2H), 2.19 (s, 3H), 1.73 (t, *J* = 2.4 Hz, 3H); ^{13}C NMR (101 MHz, $CDCl_3$) δ 143.7, 135.5, 132.3, 131.0, 130.9, 130.3, 130.0, 129.5, 128.4, 128.0, 127.6, 127.1, 127.03, 127.02, 126.7, 122.7, 122.6, 118.8, 86.3, 84.0, 82.1, 71.7, 37.5, 37.3, 21.4, 3.5; HRMS (FD) calcd for $C_{28}H_{23}NO_2S$ $[M]^+$ 437.1450, found 437.1439.

N-(But-2-yn-1-yl)-4-methyl-N-(3-(pyren-4-yl)prop-2-yn-1-yl)benzenesulfonamide (1p)

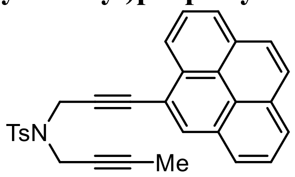

1p was prepared from N-(but-2-yn-1-yl)-4-methyl-N-(prop-2-yn-1-yl)benzenesulfonamide (**S1c,**[4] 0.261 g, 1.00 mmol) and 4-bromopyrene (0.309 g, 1.10 mmol) according to the procedure for **1m**. The final residue was purified by silica gel column chromatography (n-hexane/CH_2Cl_2 = 10:1), which furnished **1p** (0.282 g, 0.610 mmol, 61% yield).

White solid; mp 90.2–92.2 °C; ^{1}H NMR (400 MHz, $CDCl_3$) δ 8.23–8.19 (m, 3H), 8.10 (d, J = 2.8 Hz, 1H), 8.08 (d, J = 3.0 Hz, 1H), 8.05–8.01 (m, 3H), 7.85–7.81 (m, 3H), 7.20 (d, J = 8.0 Hz, 2H), 4.64 (s, 2H), 4.29 (q, J = 2.3 Hz, 2H), 2.17 (s, 3H), 1.74 (t, J = 2.4 Hz, 3H); ^{13}C NMR (101 MHz, $CDCl_3$) δ 143.7, 135.5, 132.0, 131.3, 131.2, 131.0, 129.7, 129.5, 128.4, 128.3, 128.0, 127.2, 126.3, 125.69, 125.66, 125.2, 124.3, 124.25, 124.19, 116.8, 87.4, 84.8, 82.1, 71.8, 37.6, 37.3, 21.4, 3.6; HRMS (FD) calcd for $C_{30}H_{23}NO_2S$ $[M]^+$ 461.1450, found 461.1421.

N-(3-(1H-Indol-7-yl)prop-2-yn-1-yl)-N-(but-2-yn-1-yl)-4-methylbenzenesulfonamide (1q)

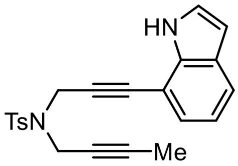

1q was prepared from N-(but-2-yn-1-yl)-4-methyl-N-(prop-2-yn-1-yl)benzenesulfonamide (**S1c,**[4] 0.261 g, 1.00 mmol) and 7-bromoindole (0.216 g, 1.10 mmol) according to the procedure for **1m**. The final residue was purified by silica gel column chromatography (n-hexane/EtOAc = 4:1), which furnished **1q** (0.165 g, 0.437 mmol, 44% yield).

Deep purple oil; ^{1}H NMR (400 MHz, $CDCl_3$) δ 8.61 (s, 1H), 7.78 (d, J = 8.3 Hz, 2H), 7.59 (d, J = 7.8 Hz, 1H), 7.21 (d, J = 8.0 Hz, 2H), 7.18 (dd, J = 2.8, 2.8 Hz, 1H), 7.08 (dd, J = 0.9, 7.3 Hz, 1H), 7.00 (dd, J = 7.6, 7.6 Hz, 1H), 6.52 (dd, J = 2.1, 3.2 Hz, 1H), 4.45 (s, 2H), 4.17 (q, J = 2.3 Hz, 2H), 2.28 (s, 3H), 1.66 (t, J = 2.4 Hz, 3H); ^{13}C NMR (101 MHz, $CDCl_3$) δ 144.1, 137.0, 135.3, 129.6, 128.1, 127.5, 125.0, 124.7, 121.7, 119.5, 105.2, 103.1, 86.2, 82.4, 82.2, 71.9, 37.5, 37.4, 21.5, 3.5; HRMS (FD) calcd for $C_{22}H_{20}N_2O_2S$ $[M]^+$ 376.1246, found 376.1246.

N-(3-([1,1'-Biphenyl]-2-yl)prop-2-yn-1-yl)-N-(but-2-yn-1-yl)-4-methylbenzenesulfonamide (1r)

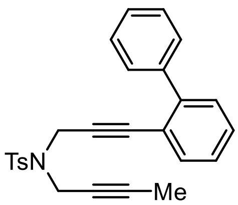

1r was prepared from N-(but-2-yn-1-yl)-4-methyl-N-(prop-2-yn-1-yl)benzenesulfonamide (**S1c,**[4] 0.522 g, 2.00 mmol) and 2-iodobiphenyl (0.616 g, 2.20 mmol) according to the procedure for **1g**. The final residue was purified by silica gel column chromatography (n-hexane/EtOAc = 4:1), which furnished **1r** (0.478 g, 1.17 mmol, 58% yield).

Brown oil; ^{1}H NMR (400 MHz, $CDCl_3$) δ 7.64 (d, J = 8.3 Hz, 2H), 7.44–7.23 (m, 9H), 7.18 (d, J = 8.0 Hz, 2H), 4.25 (s, 2H), 3.82 (q, J = 2.2 Hz, 2H), 2.30 (s, 3H), 1.65 (t, J = 2.4 Hz, 3H); ^{13}C NMR (101 MHz, $CDCl_3$) δ 143.9, 143.5, 140.3, 135.4, 133.1, 129.4, 129.3, 129.0, 128.6, 128.0, 127.9, 127.4, 126.9, 120.7, 85.3, 84.6, 81.7, 71.6, 37.1, 36.8, 21.4, 3.5; HRMS (FD) calcd for $C_{26}H_{23}NO_2S$ $[M]^+$ 413.1450, found 413.1424.

4-Methyl-*N*,*N*-bis(3-(thiophen-3-yl)prop-2-yn-1-yl)benzenesulfonamide (1k)

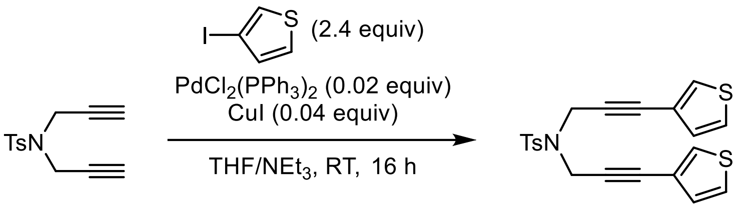

S1k **1k**

A solution of 4-methyl-*N*,*N*-di(prop-2-yn-1-yl)benzenesulfonamide (**S1k**,[10] 1.237 g, 5.00 mmol), 3-iodothiophene (2.520 g, 12.00 mmol), $PdCl_2(PPh_3)_2$ (140.4 mg, 0.020 mmol), and CuI (76.2 mg, 0.040 mmol) in degassed THF/NEt_3 (20:7, 27 mL) was stirred at room temperature for 16 h. The reaction mixture was filtered with Celite® and concentrated. The residue was purified by silica gel column chromatography (*n*-hexane/ EtOAc = 19:1), which furnished **1k** (0.451 g, 1.09 mmol, 22% yield).

Yellow solid; mp 94.3–95.6 °C; ^{1}H NMR (400 MHz, $CDCl_3$) δ 7.78 (d, *J* = 8.3 Hz, 2H), 7.27-7.22 (m, 4H), 7.22-7.18 (m, 2H), 6.89 (dd, *J* = 1.2, 5.0 Hz, 2H), 4.40 (s, 4H), 2.33 (s, 3H); ^{13}C NMR ($CDCl_3$, 100 MHz) δ 143.76, 135.50, 129.72, 129.52, 129.15, 128.00, 125.22, 121.28, 81.34, 80.92, 37.49, 21.45; HRMS (FD) calcd for $C_{21}H_{17}NO_2S_3$ $[M]^+$ 411.0421, found 411.0434.

Dimethyl 4,4'-(tosylazanediyl)bis(but-2-ynoate) (1l)

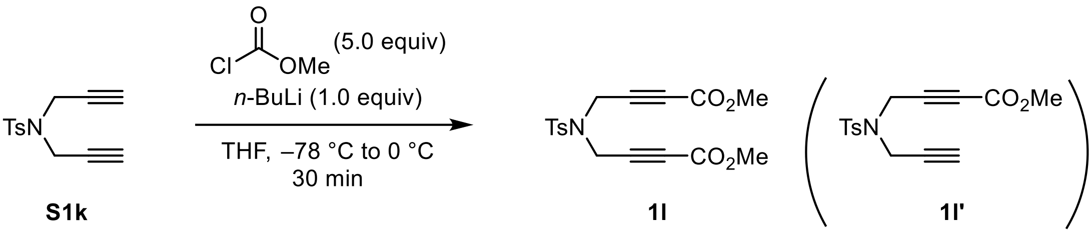

S1k **1l** **1l'**

To a solution of 4-methyl-*N*,*N*-di(prop-2-yn-1-yl)benzenesulfonamide (**S1k,**[10] 0.741 g, 3.00 mmol) in THF (20 mL) was added dropwise a 1.58 M solution of *n*-BuLi in hexane (1.90 mL, 3.00 mmol of *n*-BuLi) at −78 °C. After stirring at −78 °C for 0.5 h, methyl chloroformate (1.417 g, 15.0 mmol) was added, and the mixture was warmed to 0 °C. The resulting mixture was stirred at 0 °C for 30 min. The reaction was quenched by the addition of phosphate buffer solution [NaH_2PO_4 (1.36 g, 10.0 mmol) and Na_2HPO_4 (1.74 g, 10.0 mmol) in water 100 mL], and organic layer was immediately separated and dried over Na_2SO_4. The aqueous layer was extracted with Et_2O, and organic layer was combined, dried over Na_2SO_4, and concentrated. The residue was purified by silica gel column chromatography (*n*-hexane/ EtOAc = 4:1), which furnished **1l** (0.156 g, 0.430 mmol, 14% yield) and methyl 4-((4-methyl-*N*-(prop-2-yn-1-yl)phenyl)sulfonamido)but-2-ynoate (**1l’**, 0.424 g, 1.39 mmol, 57% yield).

White solid; mp 108.7–110.7 °C; ^{1}H NMR (400 MHz, $CDCl_3$) δ 7.71 (d, *J* = 8.3 Hz, 2H), 7.33 (d, *J* = 8.0 Hz, 2H), 4.28 (s, 4H), 3.73 (s, 6H), 2.43 (s, 3H); ^{13}C NMR (101 MHz, $CDCl_3$) δ 152.9, 144.8, 134.4, 130.0, 127.8, 79.5, 77.6, 52.8, 36.7, 21.6; HRMS (ESI) calcd for $C_{17}H_{17}NO_6S$ $[M+Na]^+$ 386.0674, found 386.0661.

N-(3-(2-Aminophenyl)prop-2-yn-1-yl)-4-methyl-N-(3-(naphthalen-1-yl)prop-2-yn-1-yl)benzenesulfonamide (1u)

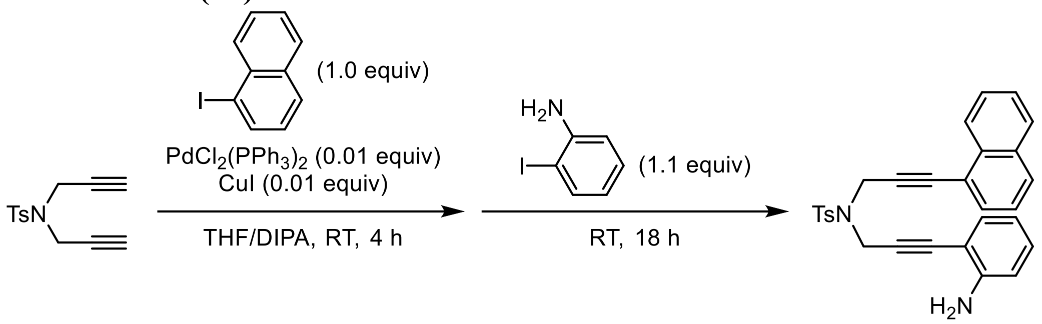

S1k **1u**

A solution of 4-methyl-N,N-di(prop-2-yn-1-yl)benzenesulfonamide (**S1k,**[10] 2.477 g, 10.00 mmol), 1-iodonaphthalene (2.541 g, 10.00 mmol), $PdCl_2(PPh_3)_2$ (70.2 mg, 0.100 mmol), and CuI (19.0 mg, 0.100 mmol) in degassed THF/diisopropylamine (DIPA) (1:1, 100 mL) was stirred at room temperature for 4 h. 2-Iodoaniline (2.409 g, 11.00 mmol) was added, and the resulting mixture was stirred at room temperature for 18 h. The reaction mixture was filtered with Celite® and concentrated. The residue was purified by silica gel column chromatography (*n*-hexane/ EtOAc = 4:1), which furnished **1u** (1.495 g, 3.22 mmol, 32% yield).

Brown oil; ^{1}H NMR (400 MHz, $CDCl_3$) δ 8.02 (dd, J = 1.3, 7.6 Hz, 1H), 7.83–7.79 (m, 4H), 7.51–7.42 (m, 3H), 7.36 (dd, J = 7.2, 8.1 Hz, 1H), 7.21 (d, J = 8.0 Hz, 2H), 7.11–7.07 (m, 2H), 6.63–6.59 (m, 2H), 4.62 (s, 2H), 4.56 (s, 2H), 4.08 (s, 2H), 2.22 (s, 3H); ^{13}C NMR (101 MHz, $CDCl_3$) δ 148.3, 144.1, 135.2, 133.15, 133.06, 132.4, 130.8, 130.0, 129.7, 129.1, 128.3, 127.9, 126.9, 126.5, 125.9, 125.0, 119.8, 117.6, 114.3, 106.7, 87.0, 86.5, 84.2, 82.9, 37.9, 37.8, 21.4; HRMS (FD) calcd for $C_{29}H_{24}N_2O_2S$ $[M]^+$ 464.1559, found 464.1568.

2.1.2. Synthesis of Alkynyl Ethers

Methyl 3-(2-(*tert*-butyl)-6-methylphenoxy)propiolate (2a)

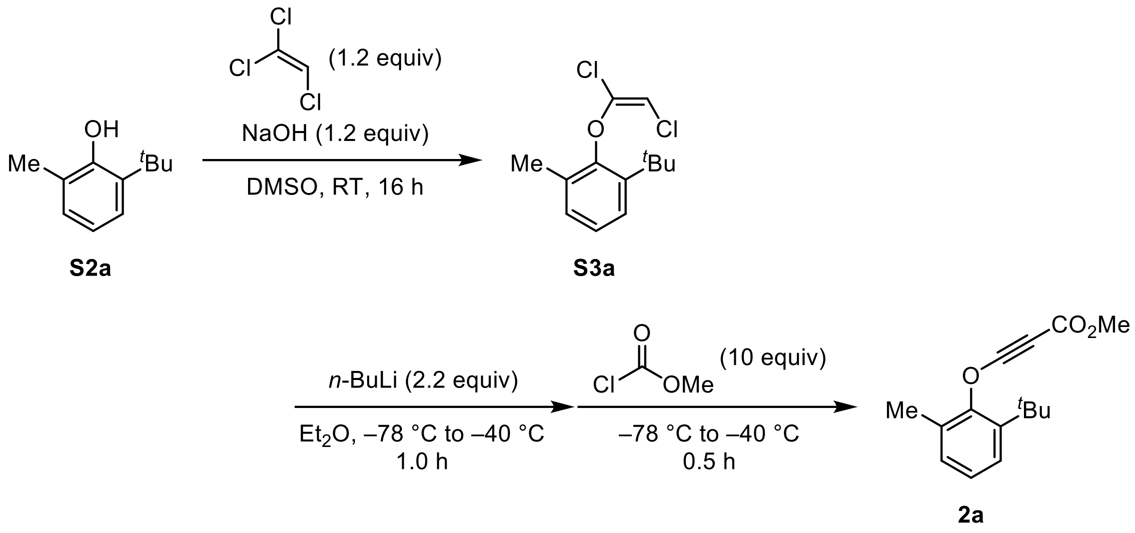

To a solution of 2-*tert*-butyl-6-methylphenol (**S2a**, 25.0 g, 0.152 mol) in DMSO (300 mL) was added crushed sodium hydroxide (7.3 g, 0.18 mol). After the resulting mixture was stirred at room temperature for 2 h, trichloroethylene (24.0 g, 0.180 mol) was added to the mixture. The resulting mixture was stirred at room temperature for 16 h. The reaction was quenched by the addition of water and extracted with Et_2O. The combined organic layer was washed with brine, dried over Na_2SO_4, and concentrated. The residue was purified by silica gel column chromatography (*n*-hexane), which furnished **S3a** (34.9 g, 0.135 mol, 89% yield).

To a solution of **S3a** (6.75 g, 26.1 mmol) in Et_2O (130 mL) was added dropwise a 1.58 M solution of *n*-BuLi in hexane (36.3 mL, 57.4 mmol of *n*-BuLi) at −78 °C. After stirring at −78 °C for 0.5 h, the mixture was warmed to −40 °C, and was stirred at −40 °C for 0.5 h. The resulting mixture was again cooled to −78 °C and methyl chloroformate (24.6 g, 0.261 mol) was added, and the mixture was warmed to −40 °C. The resulting mixture was stirred at −40 °C for 30 min. The reaction was quenched by the addition of phosphate buffer solution [NaH_2PO_4 (1.36 g, 10.00 mmol) and Na_2HPO_4 (1.74 g, 10.00 mmol) in water 100 mL], and organic layer was immediately separated and dried over Na_2SO_4. The aqueous layer was extracted with Et_2O, and organic layer was combined, dried over Na_2SO_4, and concentrated. The residue was purified by silica gel column chromatography (*n*-hexane/EtOAc = 10:1), which furnished **2a** (6.30 g, 25.6 mmol, 98% yield).

(*E*)-1-(*tert*-Butyl)-2-((1,2-dichlorovinyl)oxy)-3-methylbenzene (S3a)

Yellow oil; ^{1}H NMR (400 MHz, $CDCl_3$) δ 7.23 (dd, *J* = 2.0, 7.6 Hz, 1H), 7.08 (dd, *J* = 7.5, 7.5 Hz, 1H), 7.05 (dd, *J* = 1.8, 7.5 Hz, 1H), 5.56 (s, 1H), 2.24 (s, 3H), 1.39 (s, 9H); ^{13}C NMR (101 MHz, $CDCl_3$) δ 150.7, 142.3, 141.1, 132.1, 129.4, 125.6, 125.1, 94.1, 35.0, 30.7, 16.4; HRMS (FD) calcd for $C_{13}H_{16}Cl_2O$ $[M]^+$ 258.0578, found 258.0567.

Methyl 3-(2-(*tert*-butyl)-6-methylphenoxy)propiolate (2a)

Yellow oil; ^{1}H NMR (400 MHz, $CDCl_3$) δ 7.23–7.20 (m, 1H), 7.14–7.09 (m, 2H), 3.74 (s, 3H), 2.44 (s, 3H), 1.41 (s, 9H); ^{13}C NMR (101 MHz, $CDCl_3$) δ 155.0, 153.6, 140.7, 130.3, 130.2, 126.7, 125.5, 93.6, 52.4, 35.8, 35.0, 30.6, 16.3; HRMS (ESI) calcd for $C_{15}H_{18}O_3$ $[M+Na]^+$ 269.1154, found 269.1129.

(*E*)-1-(*tert*-Butyl)-2-((1,2-dichlorovinyl)oxy)-3-methoxy-5-methylbenzene (S3b)

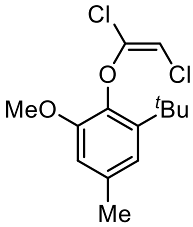

S3b was prepared from 2-(*tert*-butyl)-6-methoxy-4-methylphenol (**S2b**,[12] 2.01 g, 10.5 mmol) according to the procedure for **S3a**. The residue was purified by silica gel column chromatography (*n*-hexane/EtOAc = 100:1), which furnished **S3b** (2.60 g, 8.97 mmol, 87% yield).

Pale yellow oil; ^{1}H NMR (400 MHz, $CDCl_3$) δ 6.76 (d, *J* = 1.3 Hz, 1H), 6.64 (d, *J* = 1.6 Hz, 1H), 5.48 (s, 1H), 3.81 (s, 3H), 2.33 (s, 3H), 1.37 (s, 9H); ^{13}C NMR (101 MHz, $CDCl_3$) δ 152.1, 142.5, 142.1, 138.8, 135.3, 119.5, 111.5, 93.1, 56.2, 34.9, 30.4, 21.8; HRMS (FD) calcd for $C_{14}H_{18}Cl_2O_2$ $[M]^+$ 288.0684, found 288.0684.

Methyl 3-(2-(*tert*-butyl)-6-methoxy-4-methylphenoxy)propiolate (2b)

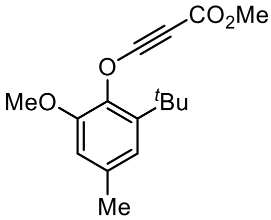

2b was prepared from **S3b** (0.871 g, 3.00 mmol) according to the procedure for **2a**. The residue was purified by silica gel column chromatography (*n*-hexane/EtOAc = 10:1), which furnished **2b** (0.542 g, 1.87 mmol, 62% yield).

Pale yellow oil; ^{1}H NMR (400 MHz, $CDCl_3$) δ 6.71–6.70 (m, 2H), 3.88 (s, 3H), 3.72 (s, 3H), 2.31 (s, 3H), 1.37 (s, 9H); ^{13}C NMR (101 MHz, $CDCl_3$) δ 155.3, 150.9, 142.0, 141.1, 136.6, 119.3, 112.2, 94.7, 56.4, 52.2, 34.9, 34.8, 30.3, 21.7; HRMS (ESI) calcd for $C_{16}H_{20}O_4$ $[M+Na]^+$ 299.1259, found 299.1255.

(*E*)-3-(*tert*-Butyl)-2-((1,2-dichlorovinyl)oxy)-4',5-dimethyl-1,1'-biphenyl (S3c)

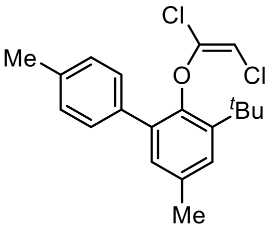

S3c was prepared from 3-(*tert*-butyl)-4',5-dimethyl-[1,1'-biphenyl]-2-ol (**S2c**,[13] 2.544 g, 10.00 mmol) according to the procedure for **S3a**. The residue was purified by silica gel column chromatography (*n*-hexane/EtOAc = 10:1), which furnished **S3c** (2.757 g, 7.89 mmol, 79% yield).

White solid; mp 99.3–100.3 °C; ^{1}H NMR (400 MHz, $CDCl_3$) δ 7.36 (d, *J* = 8.1 Hz, 2H), 7.17 (d, *J* = 7.9 Hz, 2H), 7.15 (d, *J* = 2.2 Hz, 1H), 7.00 (d, *J* = 1.6 Hz, 1H), 5.04 (s, 1H), 2.38 (s, 3H), 2.36 (s, 3H), 1.42 (s, 9H); ^{13}C NMR (101 MHz, $CDCl_3$) δ 146.9, 142.3, 140.2, 137.0, 135.8, 135.0, 134.6, 129.8, 129.4, 128.6, 126.9, 94.3, 35.0, 30.6, 21.3, 21.2; HRMS (FD) calcd for $C_{20}H_{22}Cl_2O$ $[M]^+$ 348.1048, found 348.1051.

Methyl 3-((3-(*tert*-butyl)-4',5-dimethyl-[1,1'-biphenyl]-2-yl)oxy)propiolate (2c)

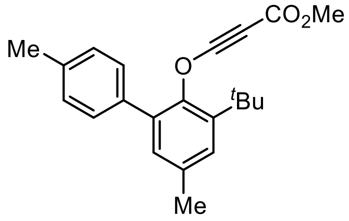

2c was prepared from **S3c** (1.045 g, 3.00 mmol) according to the procedure for **2a**. The residue was purified by silica gel column chromatography (*n*-hexane/EtOAc = 10:1), which furnished **2c** (0.867 g, 2.58 mmol, 86% yield).

White solid; mp 118.4–120.0 °C; ^{1}H NMR (400 MHz, $CDCl_3$) δ 7.33 (d, *J* = 8.1 Hz, 2H), 7.23 (d, *J* = 7.9 Hz, 2H), 7.13 (d, *J* = 2.0 Hz, 1H), 7.02 (d, *J* = 1.8 Hz, 1H), 3.57 (s, 3H), 2.39 (s, 3H), 2.35 (s, 3H), 1.42 (s, 9H); ^{13}C NMR (101 MHz, $CDCl_3$) δ 154.8, 150.3, 140.5, 137.5, 136.2, 134.5, 133.4, 130.5, 129.21, 129.18, 127.2, 94.1, 52.1, 36.0, 35.1, 30.6, 21.3, 21.1; HRMS (FD) calcd for $C_{22}H_{24}O_3$ $[M]^+$ 336.1725, found 336.1733.

Methyl 3-(2-(*tert*-butyl)-6-ethoxy-4-methylphenoxy)propiolate (2d)

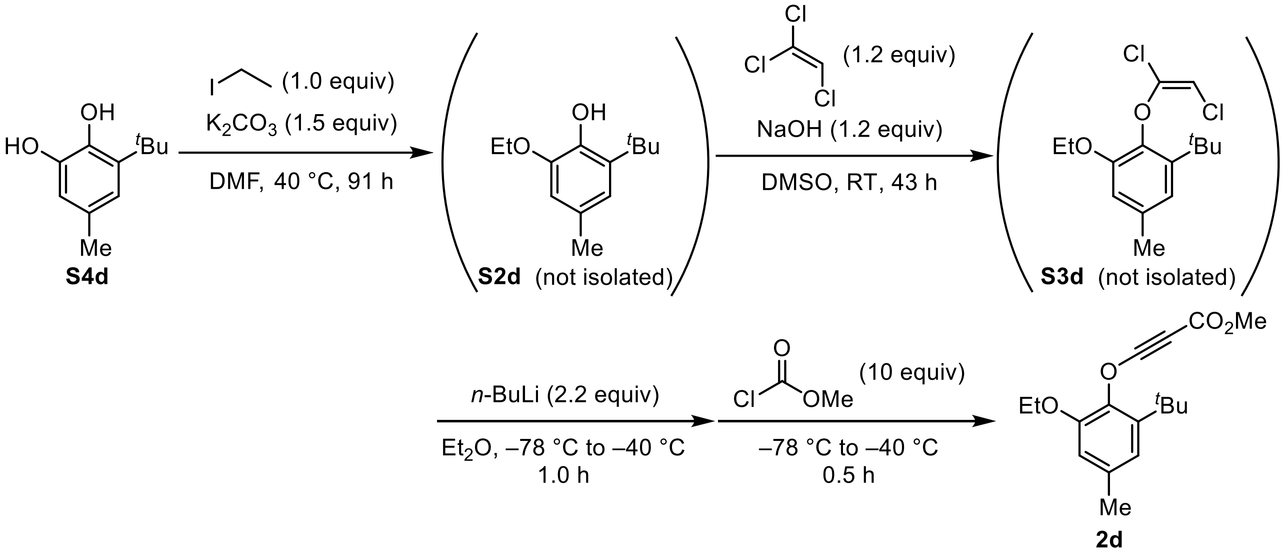

To a solution of 3-(*tert*-butyl)-5-methylbenzene-1,2-diol (**S4d**,[12] 0.902 g, 5.00 mmol) in DMF (140 mL) was added dropwise iodoethane (0.780 g, 5.00 mmol). The resulting mixture was stirred at 40 °C for 91 h. The reaction was quenched by the addition of water and extracted with CH_2Cl_2. The combined organic layer was washed with brine, dried over Na_2SO_4, and concentrated. The residue was passed through silica gel (*n*-hexane/EtOAc = 10:1) to give crude **S2d** (0.823 g, 3.95 mmol, ca. 79% yield), which was used in the next step without further purification.

S3d was prepared from crude **S2d** (0.823 g, 3.95 mmol) according to the procedure for **S3a**. The residue was passed through silica gel (*n*-hexane/EtOAc = 96:4), which furnished crude **S3d** (0.582 g, 1.92 mmol, ca. 49% yield).

2d was prepared from crude **S3d** (0.576 g, 1.90 mmol) according to the procedure for **2a**. The residue was purified by silica gel column chromatography (*n*-hexane/EtOAc = 10:1), which furnished **2d** (0.428 g, 1.47 mmol, ca. 78% yield, 30% yield from **S4d**).

Orange oil; ^{1}H NMR (400 MHz, $CDCl_3$) δ 6.68 (s, 2H), 4.11 (q, *J* = 7.0 Hz, 2H), 3.73 (s, 3H), 2.31 (s, 3H), 1.46 (t, *J* = 7.0 Hz, 3H), 1.37 (s, 9H); ^{13}C NMR (101 MHz, $CDCl_3$) δ 155.3, 150.1, 142.0, 141.0, 136.4, 119.1, 112.9, 94.9, 77.2, 65.0, 52.3, 34.9, 30.3, 21.7, 14.7; HRMS (FD) calcd for $C_{17}H_{22}O_4$ $[M]^+$ 290.1518, found 290.1509.

Methyl 3-(2-(*tert*-butyl)-6-(methoxymethoxy)-4-methylphenoxy)propiolate (2e)

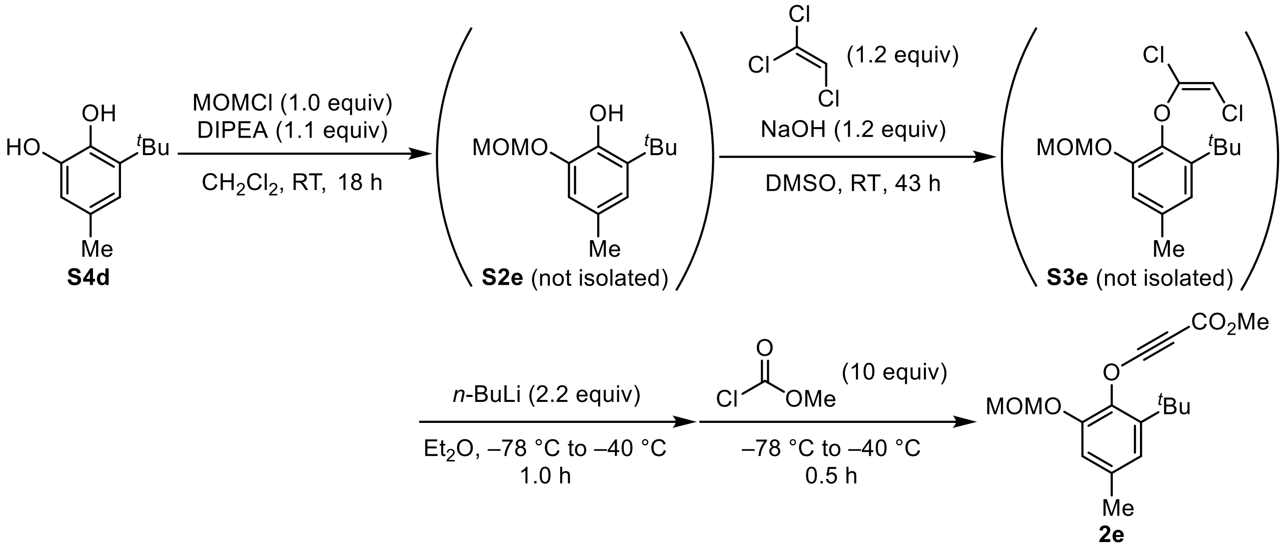

To a solution of 3-(*tert*-butyl)-5-methylbenzene-1,2-diol (**S4d**,[12] 0.902 g, 5.00 mmol) in CH_2Cl_2 (25 mL) was added *N,N*-diisopropylethylamine (DIPEA, 0.711 g, 5.50 mmol) and chloromethyl methyl ether (MOMCl, 0.403 g, 5.00 mmol) at 0 °C. The resulting mixture was stirred at room temperature for 18 h. The reaction was quenched by the addition of MeOH and water and extracted with CH_2Cl_2. The combined organic layer was washed with brine, dried over Na_2SO_4, and concentrated. The residue was passed through silica gel (*n*-hexane/EtOAc = 20:1) to give crude **S2e** (0.783 g, 3.49 mmol, ca. 70% yield), which was used in the next step without further purification.

S3e was prepared from crude **S2e** (0.777 g, 3.46 mmol) according to the procedure for **S3a**. The residue was passed through silica gel (*n*-hexane/EtOAc = 97:3), which furnished **S3e** (1.068 g, 3.35 mmol, ca. 97% yield).

2e was prepared from crude **S3e** (0.641 g, 2.00 mmol) according to the procedure for **2a**. The residue was purified by silica gel column chromatography (*n*-hexane/EtOAc = 10:1), which furnished **2e** (0.435 g, 1.42 mmol, ca. 71% yield, 48% yield from **S4d**).

Brown oil; ^1H NMR (400 MHz, $CDCl_3$) δ 6.93 (d, *J* = 1.4 Hz, 1H), 6.77 (d, *J* = 1.4 Hz, 1H), 5.24 (s, 2H), 3.70 (s, 3H), 3.53 (s, 3H), 2.30 (s, 3H), 1.38 (s, 9H); ^{13}C NMR (101 MHz, $CDCl_3$) δ 155.1, 148.4, 142.3, 141.1, 136.7, 120.4, 115.0, 94.9, 94.6, 56.4, 52.2, 35.0, 34.9, 30.3, 21.6; HRMS (ESI) calcd for $C_{17}H_{22}O_5$ $[M+Na]^+$ 329.1365, found 329.1371.

Ethyl 3-(2-(*tert*-butyl)-6-methoxy-4-methylphenoxy)propiolate (2f)

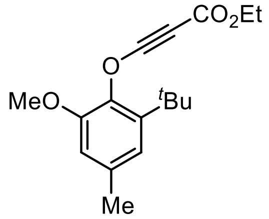

2f was prepared from **S3b** (0.287 g, 1.00 mmol) and ethyl chloroformate (1.085 g, 10.00 mmol) according to the procedure for **2a**. The residue was purified by silica gel column chromatography (*n*-hexane/EtOAc = 10:1), which furnished **2f** (0.176 g, 0.604 mmol, 61% yield).

Yellow oil; ^1H NMR (400 MHz, $CDCl_3$) δ 6.70 (d, *J* = 0.5 Hz, 1H), 6.70 (d, *J* = 0.4 Hz, 1H), 4.19 (q, *J* = 7.1 Hz, 2H), 3.90 (s, 3H), 2.32 (s, 3H), 1.37 (s, 9H), 1.28 (t, *J* = 7.1 Hz, 3H); ^{13}C NMR (101 MHz, $CDCl_3$) δ 154.9, 151.0, 142.0, 141.1, 136.5, 119.3, 112.2, 94.5, 61.4, 56.4, 35.1, 35.0, 30.4, 21.7, 14.1; HRMS (FD) calcd for $C_{17}H_{22}O_4$ $[M]^+$ 290.1518, found 290.1510.

***tert*-Butyl 3-(2-(*tert*-butyl)-6-methoxy-4-methylphenoxy)propiolate (2g)**

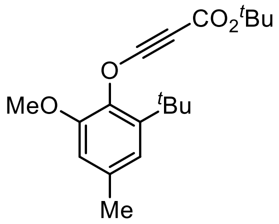

2f was prepared from **S3b** (0.288 g, 1.00 mmol) and di-*tert*-butyl dicarbonate (0.437 g, 2.00 mmol) according to the procedure for **2a**. The residue was purified by silica gel column chromatography (*n*-hexane/EtOAc = 10:1), which furnished **2g** (0.201 g, 0.631 mmol, 63% yield).

Yellow oil; ^{1}H NMR (400 MHz, $CDCl_3$) δ 6.70 (s, 1H), 6.69 (s, 1H), 3.90 (s, 3H), 2.32 (s, 3H), 1.48 (s, 9H), 1.37 (s, 9H) ^{13}C NMR (101 MHz, $CDCl_3$) δ 154.0, 151.1, 142.1, 141.1, 136.3, 119.3, 112.3, 92.5, 82.2, 56.5, 36.2, 35.0, 30.4, 28.1, 21.7; HRMS (FD) calcd for $C_{19}H_{26}O_4$ $[M]^+$ 318.1831, found 318.1829.

4-(2-(*tert*-Butyl)-6-methoxy-4-methylphenoxy)but-3-yn-2-one (2h)

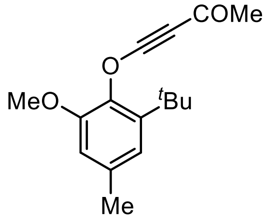

2h was prepared from **S3b** (0.288 g, 1.00 mmol) and acetyl chloride (0.785 g, 10.0 mmol) according to the procedure for **2a**. The residue was purified by silica gel column chromatography (*n*-hexane/EtOAc = 10:1), which furnished **2h** (0.095 g, 0.38 mmol, 38% yield).

Yellow oil; ^{1}H NMR (400 MHz, $CDCl_3$) δ 6.72 (s, 1H), 6.71 (s, 1H), 3.89 (s, 3H), 2.33 (s, 3H), 2.29 (s, 3H), 1.37 (s, 9H); ^{13}C NMR (101 MHz, $CDCl_3$) δ 184.7, 150.9, 142.3, 141.1, 136.6, 119.3, 112.1, 101.1, 56.3, 44.3, 35.0, 32.8, 30.3, 21.7; HRMS (FD) calcd for $C_{16}H_{20}O_3$ $[M]^+$ 260.1412, found 260.1410.

Methyl 3-(2-((3*r*,5*r*,7*r*)-adamantan-1-yl)-4,6-dimethylphenoxy)propiolate (2i)

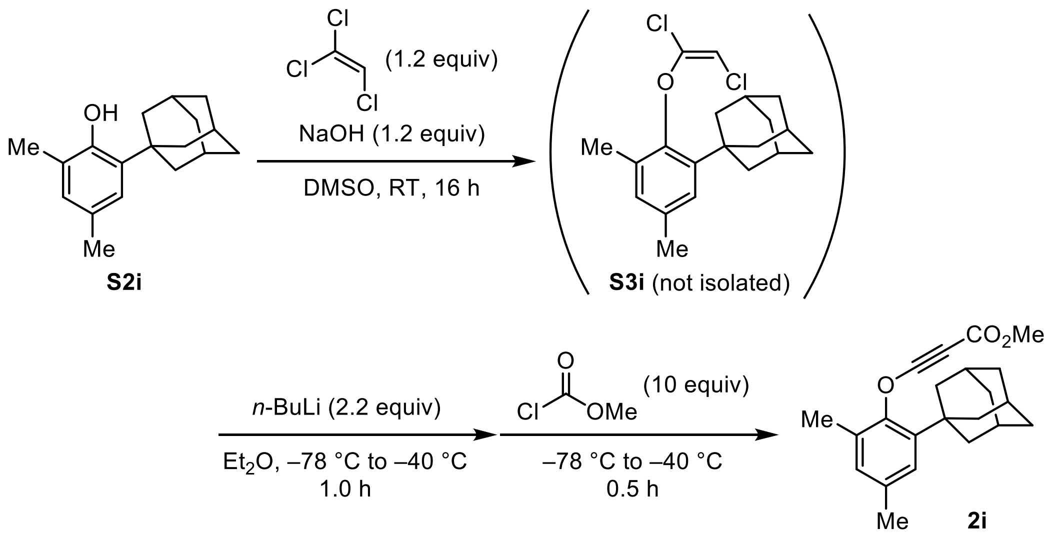

Crude **S3i** was prepared from 2-((3*r*,5*r*,7*r*)-adamantan-1-yl)-4,6-dimethylphenol (**S2i**,[14] 2.564 g, 10.00 mmol) according to the procedure for **S3a**. The residue was passed through silica gel (*n*-hexane),

which furnished crude **S3i** (1.627 g, 4.63 mmol, ca. 46% yield).

2i was prepared from crude **S3i** (1.627 g, 4.63 mmol) according to the procedure for **2a**. The residue was purified by silica gel column chromatography (*n*-hexane/EtOAc = 20:1), which furnished **2i** (1.435 g, 4.24 mmol, ca. 91% yield, 42% from **S2i**).

White solid; mp 111.4–112.7 °C; ^{1}H NMR (400 MHz, $CDCl_3$) δ 6.95 (d, *J* = 2.0 Hz, 1H), 6.89 (d, *J* = 1.5 Hz, 1H), 3.73 (s, 3H), 2.39 (s, 3H), 2.29 (s, 3H), 2.10 (s, 3H), 2.04 (d, *J* = 2.9 Hz, 6H), 1.78 (dd, *J* = 3.0, 3.0 Hz, 6H); ^{13}C NMR (101 MHz, $CDCl_3$) δ 155.1, 152.0, 140.4, 136.3, 130.5, 129.8, 126.1, 94.0, 52.4, 41.5, 37.2, 36.8, 36.0, 29.0, 21.1, 16.3; HRMS (ESI) calcd for $C_{22}H_{26}O_3$ $[M+Na]^+$ 361.1780, found 361.1765.

(*E*)-3-(*tert*-Butyl)-2-((1,2-dichlorovinyl)oxy)-4'-methoxy-5-methyl-1,1'-biphenyl (S3j)

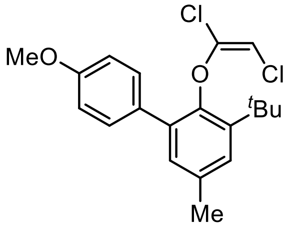

S3j was prepared from 3-(*tert*-butyl)-4'-methoxy-5-methyl-[1,1'-biphenyl]-2-ol (**S2j**,[15] 1.163 g, 4.30 mmol) according to the procedure for **S3a**. The residue was purified by silica gel column chromatography (*n*-hexane/EtOAc = 3:1), which furnished **S3j** (1.386 g, 3.80 mmol, 79% yield).

White solid; mp 111.7–113.4 °C; ^{1}H NMR (400 MHz, $CDCl_3$) δ 7.39 (d, *J* = 8.8 Hz, 2H), 7.14 (d, *J* = 1.8 Hz, 1H), 6.98 (d, *J* = 1.6 Hz, 1H), 6.90 (d, *J* = 8.8 Hz, 2H), 5.05 (s, 1H), 3.83 (s, 3H), 2.36 (s, 3H), 1.42 (s, 9H); ^{13}C NMR (101 MHz, $CDCl_3$) δ 159.0, 146.9, 142.3, 140.3, 135.5, 135.0, 130.7, 129.9, 129.8, 126.7, 113.4, 94.3, 55.3, 35.0, 30.7, 21.3; HRMS (FD) calcd for $C_{20}H_{22}Cl_2O_2$ $[M]^+$ 364.0997, found 364.1003.

Methyl 3-((3-(*tert*-butyl)-4'-methoxy-5-methyl-[1,1'-biphenyl]-2-yl)oxy)propiolate (2j)

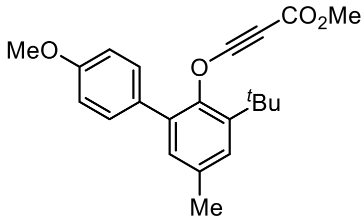

2j was prepared from **S3j** (0.728 g, 2.00 mmol) according to the procedure for **2a**. The residue was purified by silica gel column chromatography (*n*-hexane/EtOAc = 10:1), which furnished **2j** (0.516 g, 1.47 mmol, 74% yield).

Pale brown solid; mp 82.6–84.0 °C; ^{1}H NMR (400 MHz, $CDCl_3$) δ 7.37 (d, *J* = 8.8 Hz, 2H), 7.12 (d, *J* = 1.8 Hz, 1H), 7.02 (d, *J* = 1.6 Hz, 1H), 6.95 (d, *J* = 8.8 Hz, 2H), 3.84 (s, 3H), 3.57 (s, 3H), 2.35 (s, 3H), 1.42 (s, 9H); ^{13}C NMR (101 MHz, $CDCl_3$) δ 159.4, 154.8, 150.3, 140.5, 136.2, 134.2, 130.53, 130.52, 128.6, 127.1, 114.0, 94.1, 55.3, 52.1, 36.1, 35.1, 30.6, 21.1; HRMS (FD) calcd for $C_{22}H_{24}O_4$ $[M]^+$ 352.1675, found 352.1674.

(*E*)-3-(*tert*-Butyl)-2-((1,2-dichlorovinyl)oxy)-5-methyl-4'-(trifluoromethyl)-1,1'-biphenyl (S3k)

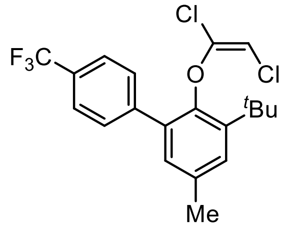

S3k was prepared from 3-(*tert*-butyl)-5-methyl-4'-(trifluoromethyl)-[1,1'-biphenyl]-2-ol (**S2k**,[13] 1.388 g, 4.50 mmol) according to the procedure for **S3a**. The residue was purified by silica gel column chromatography (*n*-hexane), which furnished **S3k** (1.688 g, 4.19 mmol, 94% yield).

White solid; mp 71.8–73.8 °C; ^{1}H NMR (400 MHz, $CDCl_3$) δ 7.62 (d, *J* = 8.3 Hz, 2H), 7.58 (d, *J* = 8.3 Hz, 2H), 7.23 (d, *J* = 1.8 Hz, 1H), 6.99 (d, *J* = 1.6 Hz, 1H), 5.03 (s, 1H), 2.38 (s, 3H), 1.43 (s, 9H); ^{13}C NMR (101 MHz, $CDCl_3$) δ 146.8, 142.7, 141.2 (q, *J* = 1.3 Hz), 139.9, 135.4, 134.5, 129.9, 129.6, 129.5 (q, *J* = 32.4 Hz), 128.0, 124.8 (q, *J* = 3.8 Hz), 124.3 (q, *J* = 272.0 Hz), 94.9, 35.1, 30.6, 21.2, ^{19}F NMR (377 MHz, $CDCl_3$) δ −62.4; HRMS (FD) calcd for $C_{20}H_{19}Cl_2F_3O$ $[M]^+$ 402.0765, found 402.0760.

Methyl 3-((3-(*tert*-butyl)-5-methyl-4'-(trifluoromethyl)-[1,1'-biphenyl]-2-yl)oxy)propiolate (2k)

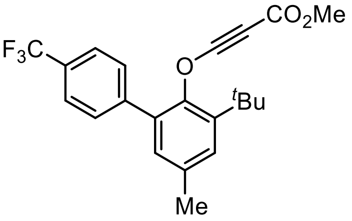

2k was prepared from **S3k** (0.806 g, 2.00 mmol) according to the procedure for **2a**. The residue was purified by silica gel column chromatography (*n*-hexane/EtOAc = 10:1), which furnished **2k** (0.711 g, 1.82 mmol, 91% yield).

White solid; mp 91.0–93.0 °C; ^{1}H NMR (400 MHz, $CDCl_3$) δ 7.69 (d, *J* = 8.1 Hz, 2H), 7.58 (d, *J* = 8.0 Hz, 2H), 7.22 (d, *J* = 1.8 Hz, 1H), 7.04 (d, *J* = 1.6 Hz, 1H), 3.55 (s, 3H), 2.37 (s, 3H), 1.43 (s, 9H); ^{13}C NMR (101 MHz, $CDCl_3$) δ 154.4, 150.1, 140.9, 140.2 (q, *J* = 1.3 Hz), 136.7, 133.0, 130.2, 129.9 (q, *J* = 32.5 Hz), 129.8, 128.4, 125.4 (q, *J* = 3.8 Hz), 124.2 (q, *J* = 272.1 Hz), 93.3, 52.1, 36.4, 35.2, 30.5, 21.1; ^{19}F NMR (377 MHz, $CDCl_3$) δ −62.6; HRMS (FD) calcd for $C_{22}H_{21}F_3O_3$ $[M]^+$ 390.1443, found 390.1436.

3-(*tert*-Butyl)-2',5-dimethyl-[1,1'-biphenyl]-2-ol (S2l)

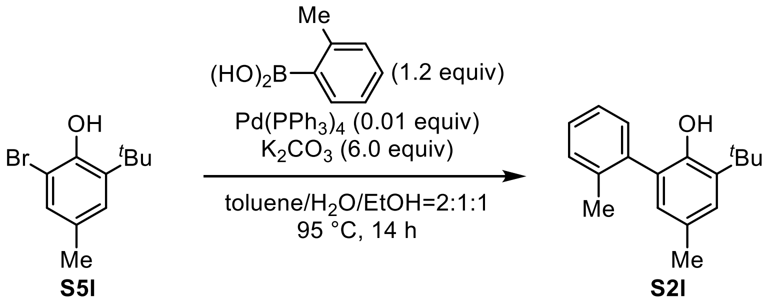

A solution of 2-bromo-6-(*tert*-butyl)-4-methylphenol (**S5l**,[16] 1.177 g, 4.84 mmol), 2-methylphenylboronic acid (0.816 g, 6.00 mmol), $Pd(PPh_3)_4$ (58.0 mg, 0.050 mmol), and K_2CO_3

(4.146 g, 30.00 mmol) in degassed toluene/H_2O/EtOH (2:1:1, 100 mL) was stirred at 95 °C for 14 h. The reaction was quenched by the addition of aqueous 2M HCl and extracted with EtOAc. The combined organic layer was washed with brine, dried over Na_2SO_4, and concentrated. The residue was purified by silica gel column chromatography (*n*-hexane/EtOAc = 10:1) to give crude **S2l** (0.738 g, 2.90 mmol, 60% yield).

Colorless oil; ^{1}H NMR (400 MHz, $CDCl_3$) δ 7.32–7.24 (m, 4H), 7.09 (d, *J* = 2.0 Hz, 1H), 6.78 (d, *J* = 1.7 Hz, 1H), 4.76 (s, 1H), 2.30 (s, 3H), 2.15 (s, 3H), 1.42 (s, 9H); ^{13}C NMR (101 MHz, $CDCl_3$) δ 148.8, 137.7, 136.1, 135.6, 130.9, 130.7, 128.5, 128.4, 128.2, 128.0, 127.0, 126.6, 34.7, 29.6, 20.8, 19.7; HRMS (FD) calcd for $C_{18}H_{22}O$ $[M]^+$ 254.1671, found 254.1653.

(*E*)-3-(*tert*-Butyl)-2-((1,2-dichlorovinyl)oxy)-2',5-dimethyl-1,1'-biphenyl (S3l)

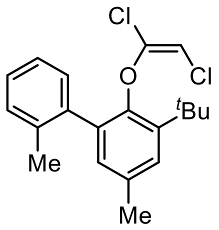

S3l was prepared from **S2l** (0.763 g, 3.00 mmol) according to the procedure for **S3a**. The residue was purified by silica gel column chromatography (*n*-hexane), which furnished **S3l** (0.837 g, 2.40 mmol, 84% yield). The diastereomers were observed on the NMR timescale.

Colorless oil; ^{1}H NMR (400 MHz, $CDCl_3$) δ 7.29–7.14 (m, 10H, major/minor), 6.93 (d, *J* = 1.6 Hz, 1H, minor), 6.87 (d, *J* = 1.6 Hz, 1H, major), 4.95 (s, 1H, minor), 4.91 (s, 1H, major), 2.36 (s, 3H, major), 2.35 (s, 3H, minor), 2.33 (s, 3H, minor), 2.17 (s, 3H, major), 1.42 (s, 9H, minor), 1.41 (s, 9H, major); ^{13}C NMR (101 MHz, $CDCl_3$) δ 147.5, 147.4, 142.3, 141.9, 140.6, 140.5, 137.6, 137.2, 136.1, 135.9, 135.2, 134.9, 134.4, 134.0, 131.5, 130.50, 130.47, 129.9, 129.8, 129.6, 127.7, 127.5, 127.1, 127.0, 125.4, 125.2, 94.7, 94.4, 35.2, 35.0, 31.7, 30.72, 30.67, 22.7, 21.3, 20.4, 20.3, 14.2; HRMS (FD) calcd for $C_{20}H_{22}Cl_2O$ $[M]^+$ 348.1048, found 348.1029.

Methyl 3-((3-(*tert*-butyl)-2',5-dimethyl-[1,1'-biphenyl]-2-yl)oxy)propiolate (2l)

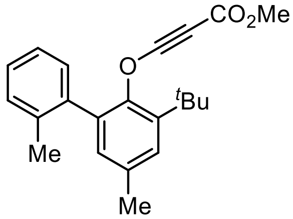

2l was prepared from **S3l** (0.416 g, 1.19 mmol) according to the procedure for **2a**. The residue was purified by silica gel column chromatography (*n*-hexane/EtOAc = 10:1), which furnished **2l** (0.326 g, 0.969 mmol, 81% yield).

Yellow oil; ^{1}H NMR (400 MHz, $CDCl_3$) δ 7.26–7.20 (m, 4H), 7.16 (d, *J* = 1.8 Hz, 1H), 6.93 (d, *J* = 1.6 Hz, 1H), 3.58 (s, 3H), 2.35 (s, 3H), 2.16 (s, 3H), 1.42 (s, 9H); ^{13}C NMR (101 MHz, $CDCl_3$) δ 154.7, 150.3, 140.3, 136.6, 136.1, 135.7, 134.2, 130.52, 130.47, 130.0, 128.2, 127.4, 125.6, 93.6, 52.1, 35.5, 35.0, 30.5, 21.1, 19.9; HRMS (FD) calcd for $C_{22}H_{24}O_3$ $[M]^+$ 336.1725, found 336.1705.

3-(2-(*tert*-Butyl)-6-methoxy-4-methylphenoxy)-*N*,*N*-dimethylpropiolamide (2m)

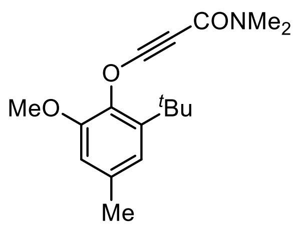

2m was prepared from **S3b** (0.434 g, 1.50 mmol) and dimethylcarbamoyl chloride (0.807 g, 7.50 mmol) according to the procedure for **2a**, except that the reaction time was extended to 72 h at −40 °C. The residue was purified by silica gel column chromatography (*n*-hexane/EtOAc = 3:1), which furnished **2m** (82.5 mg, 0.285 mmol, 19% yield).

White solid; mp 90.0–91.3 °C; ^{1}H NMR (400 MHz, $CDCl_3$) δ 6.71 (s, 1H), 6.69 (s, 1H), 3.89 (s, 3H), 3.12 (s, 3H), 2.95 (s, 3H), 2.32 (s, 3H), 1.38 (s, 9H); ^{13}C NMR (101 MHz, $CDCl_3$) δ 155.5, 151.0, 142.1, 141.2, 136.2, 119.3, 112.0, 98.1, 56.2, 38.2, 35.1, 35.0, 34.0, 30.4, 21.7; HRMS (FD) calcd for $C_{17}H_{23}NO_3$ $[M]^+$ 289.1678, found 289.1680.

3-(2-(*tert*-Butyl)-6-methylphenoxy)-*N*,*N*-dimethylpropiolamide (2n)

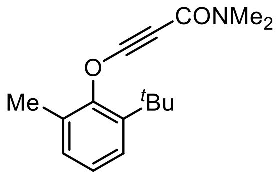

2n was prepared from **S3a** (0.391 g, 1.50 mmol) and dimethylcarbamoyl chloride (0.807 g, 7.50 mmol) according to the procedure for **2a**, except that the reaction time was extended to 72 h at −40 °C. The residue was purified by silica gel column chromatography (*n*-hexane/EtOAc = 7:3), which furnished **2n** (0.131 g, 0.503 mmol, 33% yield).

White solid; mp 56.5–58.4 °C; ^{1}H NMR (400 MHz, $CDCl_3$) δ 7.22–7.19 (m, 1H), 7.12–7.08 (m, 2H), 3.09 (s, 3H), 2.94 (s, 3H), 2.46 (s, 3H), 1.42 (s, 9H); ^{13}C NMR (101 MHz, $CDCl_3$) δ 155.1, 153.8, 140.7, 130.3, 130.2, 126.5, 125.4, 97.3, 38.2, 36.3, 35.0, 34.0, 30.6, 16.4; HRMS (FD) calcd for $C_{16}H_{21}NO_2$ $[M]^+$ 259.1572, found 259.1574.

3-((3-(*tert*-Butyl)-4',5-dimethyl-[1,1'-biphenyl]-2-yl)oxy)-*N*,*N*-dimethylpropiolamide (2o)

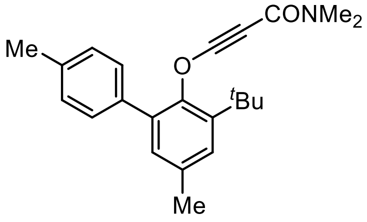

2o was prepared from **S3c** (0.173 g, 0.495 mmol) and dimethylcarbamoyl chloride (0.269 g, 2.50 mmol) according to the procedure for **2a**, except that the reaction time was extended to 72 h at −40 °C. The residue was purified by silica gel column chromatography (*n*-hexane/ EtOAc = 3:1), which furnished **2o** (0.147 g, 0.421 mmol, 84% yield).

White solid; mp 119.4–121.0 °C; ^{1}H NMR (400 MHz, $CDCl_3$) δ 7.38 (d, *J* = 8.1 Hz, 2H), 7.22 (d, *J* = 7.9 Hz, 2H), 7.13 (d, *J* = 2.0 Hz, 1H), 7.03 (d, *J* = 1.8 Hz, 1H), 2.79 (s, 3H), 2.64 (s, 3H), 2.38 (s, 3H), 2.35 (s, 3H), 1.44 (s, 9H); ^{13}C NMR (101 MHz, $CDCl_3$) δ 155.0, 150.4, 140.9, 137.5, 136.0, 134.1, 133.7, 130.5, 129.20, 129.17, 127.2, 97.1, 38.0, 36.4, 35.2, 33.9, 30.6, 21.2, 21.1; HRMS (FD) calcd for $C_{23}H_{27}NO_2$ $[M]^+$ 349.2042, found 349.2048.

1-(*tert*-Butyl)-2-((3-methoxyprop-1-yn-1-yl)oxy)-3-methylbenzene (2p)

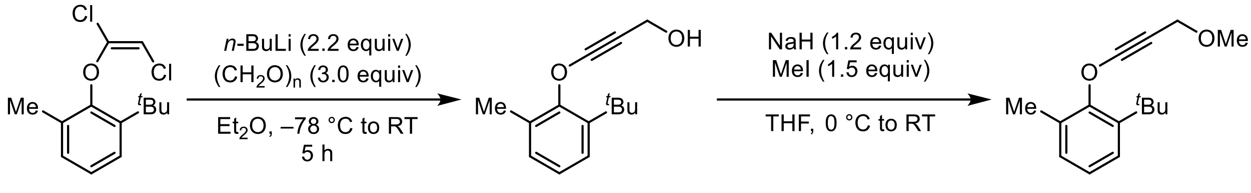

To a solution of **S3a** (7.23 g, 27.9 mmol) in Et_2O (100 mL) was added dropwise a 2.00 M solution of *n*-BuLi in hexane (30.7 mL, 61.4 mmol of *n*-BuLi) at −78 °C. After stirring at −78 °C for 0.5 h, the mixture was warmed to −40 °C, and was stirred at −40 °C for 0.5 h. The resulting mixture was cooled to −78 °C and paraformaldehyde (2.51 g, 83.7 mmol) was added, and the mixture was warmed to room temperature over 1 h. The resulting mixture was stirred at room temperature for 5 h. The reaction was quenched by the addition of phosphate buffer solution [NaH_2PO_4 (1.36 g, 10.0 mmol) and Na_2HPO_4 (1.74 g, 10.0 mmol) in water 100 mL], and organic layer was immediately separated and dried over Na_2SO_4. The aqueous layer was extracted with Et_2O, and organic layer was combined, dried over Na_2SO_4, and concentrated. The residue was purified by silica gel column chromatography (*n*-hexane/EtOAc = 5:1), which furnished **S6p** (0.451 g, 2.07 mmol, 7% yield).

To a 55 wt% sodium hydride dispersion in paraffin liquid (48.5 mg, 0.612 mmol of NaH) was added a solution of **S6p** (0.112 g, 0.514 mmol) in THF (6 mL) at 0 °C. After stirring at room temperature for 0.5 h, iodomethane (0.109 g, 0.772 mmol) was added, and the mixture was stirred at room temperature for 2 h. The reaction was quenched by the addition water and the aqueous layer was extracted with Et_2O. The combined organic layer was combined, dried over Na_2SO_4, and concentrated. The residue was purified by silica gel column chromatography (*n*-hexane/EtOAc = 10:1), which furnished **2p** (61.1 mg, 0.263 mmol, 51% yield).

3-(2-(*tert*-Butyl)-6-methylphenoxy)prop-2-yn-1-ol (S6p)

Colorless oil; ^{1}H NMR (400 MHz, $CDCl_3$) δ 7.21–7.17 (m, 1H), 7.09–7.05 (m, 2H), 4.30 (d, *J* = 6.0 Hz, 2H), 2.46 (s, 3H), 1.42 (s, 9H); ^{13}C NMR (101 MHz, $CDCl_3$) δ 154.3, 140.9, 130.7, 130.1, 125.9, 125.2, 92.6, 50.9, 38.2, 35.1, 30.6, 16.5; HRMS (FD) calcd for $C_{14}H_{18}O_2$ $[M]^+$ 218.1307, found 218.1312.

1-(*tert*-Butyl)-2-((3-methoxyprop-1-yn-1-yl)oxy)-3-methylbenzene (2p)

Yellow oil; ^{1}H NMR (400 MHz, $CDCl_3$) δ 7.20–7.18 (m, 1H), 7.09–7.04 (m, 2H), 4.14 (s, 2H), 3.34 (s, 3H), 2.47 (s, 3H), 1.43 (s, 9H); ^{13}C NMR (101 MHz, $CDCl_3$) δ 154.3, 140.9, 130.8, 130.0, 125.8, 125.1, 92.9, 59.6, 57.0, 35.4, 35.1, 30.6, 16.5; HRMS (FD) calcd for $C_{15}H_{20}O_2$ $[M]^+$ 232.1463, found 232.1450.

1-(*tert*-Butyl)-3-methyl-2-(prop-1-yn-1-yloxy)benzene (2q)

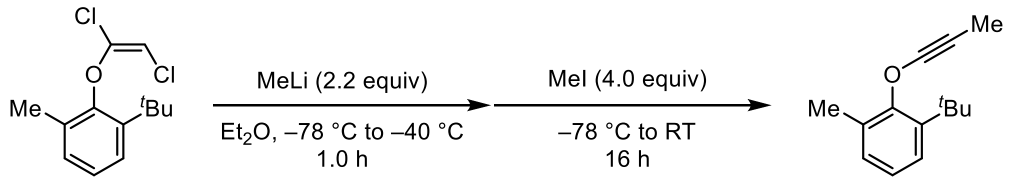

To a solution of **S2a** (2.592 g, 10.00 mmol) in Et_2O (130 mL) was added dropwise a 1.15 M solution of MeLi in Et_2O (19.1 mL, 22.0 mmol of MeLi) at −78 °C. After stirring at −78 °C for 0.5 h, the mixture was warmed to −40 °C, and was stirred at −40 °C for 0.5 h. The resulting mixture was again cooled to −78 °C and iodomethane (5.678 g, 40.00 mmol) was added, and the mixture was warmed to room temperature. The resulting mixture was stirred at room temperature for 16 h. The

reaction was quenched by the addition of phosphate buffer solution [NaH_2PO_4 (1.36 g, 10.0 mmol) and Na_2HPO_4 (1.74 g, 10.0 mmol) in water 100 mL], and organic layer was immediately separated and dried over Na_2SO_4. The aqueous layer was extracted with Et_2O, and organic layer was combined, dried over Na_2SO_4, and concentrated. The residue was purified by silica gel column chromatography (*n*-hexane), which furnished **2q** (1.236 g, 6.11 mmol, 61% yield).

Orange oil; ^{1}H NMR (400 MHz, $CDCl_3$) δ 7.17 (dd, *J* = 2.4, 7.1 Hz, 1H), 7.07–7.02 (m, 2H), 2.46 (s, 3H), 1.74 (s, 3H), 1.42 (s, 9H); ^{13}C NMR (101 MHz, $CDCl_3$) δ 153.0, 139.4, 129.4, 128.4, 123.9, 123.4, 84.8, 33.5, 32.3, 29.12, 29.07, 14.9; HRMS (FD) calcd for $C_{14}H_{18}O$ $[M]^+$ 202.1358, found 202.1345.

2.2. Rh-Catalyzed Enantioselective Synthesis of Axially Chiral Diaryl Ethers

General Procedure of Conditions A (Figure 2): (*S*)-Difluorphos (3.4 mg, 0.0050 mmol) and $[Rh(cod)_2]BF_4$ (2.0 mg, 0.0050 mmol) were dissolved in CH_2Cl_2 (1.0 mL) in a Schlenk tube, and the mixture was stirred at room temperature for 10 min. After introduction of H_2 and stirring at room temperature for 30 min, the resulting mixture was concentrated to dryness. The residue was dissolved in CH_2Cl_2 (2.3 mL) and EtOH (13.3 mL), followed by the addition of a solution of **1** (0.050 mmol) and **2** (0.100 mmol) in CH_2Cl_2 (1.0 mL, total 3.0 mM for **1** and CH_2Cl_2/EtOH = 1:4). The mixture was stirred at room temperature for 16 h and then concentrated. The crude product was purified by silica gel PTLC to furnish **3** and **4**.

General Procedure of Conditions B (Figure 2): (*S*)-Difluorphos (3.4 mg, 0.0050 mmol) and $[Rh(cod)_2]BF_4$ (2.0 mg, 0.0050 mmol) were dissolved in CH_2Cl_2 (1.0 mL) in a Schlenk tube, and the mixture was stirred at room temperature for 10 min. After introduction of H_2 and stirring at room temperature for 30 min, the resulting mixture was concentrated to dryness. The residue was dissolved in CH_2Cl_2 (15.7 mL), followed by the addition of a solution of **1** (0.050 mmol) and **2** (0.100 mmol) in CH_2Cl_2 (1.0 mL, total 3.0 mM for **1**). The mixture was stirred at room temperature for 16 h and then concentrated. The crude product was purified by silica gel PTLC to furnish **3** and **4**.

General Procedure of Conditions C (Figure 2): (*S*)-BINAP (3.1 mg, 0.0050 mmol) and $[Rh(cod)_2]BF_4$ (2.0 mg, 0.0050 mmol) were dissolved in CH_2Cl_2 (1.0 mL) in a Schlenk tube, and the mixture was stirred at room temperature for 10 min. After introduction of H_2 and stirring at room temperature for 30 min, the resulting mixture was concentrated to dryness. The residue was dissolved in CH_2Cl_2 (2.3 mL) and EtOH (13.3 mL), followed by the addition of a solution of **1** (0.050 mmol) and **2** (0.100 mmol) in CH_2Cl_2 (1.0 mL, total 3.0 mM for **1** and CH_2Cl_2/EtOH = 1:4). The mixture was stirred at room temperature for 16 h and then concentrated. The crude product was purified by silica gel PTLC to furnish **3** and **4**.

(+)-Dimethyl 6-(2-(*tert*-butyl)-6-methylphenoxy)-7-methyl-2-tosylisoindoline-4,5-dicarboxylate [(+)-3aa]

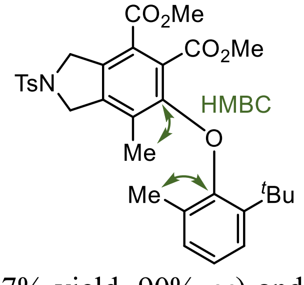

(+)-**3aa** (10.2 mg, 0.0181 mmol, 37% yield, 90% *ee*) and (+)-**4aa** (9.1 mg, 0.0161 mmol, 33% yield, 10% *ee*) were prepared from **1a** (15.7 mg, 0.0492 mmol) and **2a** (24.9 mg, 0.101 mmol) according to the general procedure of **conditions B**. Two regioisomers were isolated by silica gel PTLC (*n*-hexane/EtOAc = 2:1).

Pale yellow oil; $[\alpha]^{25}_{D}$ +88.2 (*c* 0.51, $CHCl_3$, 90% *ee*); ^1H NMR (400 MHz, $CDCl_3$) δ 7.77 (d, *J* = 8.3 Hz, 2H), 7.33 (d, *J* = 7.9 Hz, 2H), 7.22 (dd, *J* = 1.5, 7.8 Hz, 1H), 6.99 (dd, *J* = 7.6, 7.6 Hz, 1H), 6.92 (dd, *J* = 1.1, 7.4 Hz, 1H), 4.93 (dd, *J* = 1.4, 15.3 Hz, 1H), 4.81 (dd, *J* = 2.3, 15.3 Hz, 1H), 4.53 (d, *J* = 15.1 Hz, 1H), 4.47 (d, *J* = 14.4 Hz, 1H), 3.88 (s, 3H), 3.78 (s, 3H), 2.42 (s, 3H), 1.81 (s, 3H), 1.65 (s, 3H), 1.36 (s, 9H); ^{13}C NMR (101 MHz, $CDCl_3$) δ 167.5, 164.8, 153.0, 151.4, 143.9, 140.2, 139.7, 133.8, 131.6, 130.0, 129.9, 128.6, 127.52, 127.49, 126.5, 125.3, 124.1, 121.5, 55.3, 53.0, 52.7, 52.5, 35.2, 30.2, 21.5, 17.7, 14.3; HRMS (ESI) calcd for $C_{31}H_{35}NO_7S$ $[M+Na]^+$ 588.2032, found 588.2026; CHIRALPAK IF-3, *n*-hexane/2-PrOH = 80:20, 1.0 mL/min, retention times: 50.5 min (minor isomer) and 65.5 min (major isomer).

(+)-Dimethyl 5-(2-(*tert*-butyl)-6-methylphenoxy)-7-methyl-2-tosylisoindoline-4,6-dicarboxylate [(+)-4aa]

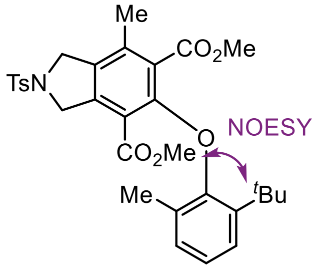

Pale yellow oil; $[\alpha]^{25}_{D}$ +5.2 (*c* 0.45, $CHCl_3$, 10% *ee*); ^1H NMR (400 MHz, $CDCl_3$) δ 7.75 (d, *J* = 8.2 Hz, 2H), 7.33 (d, *J* = 7.9 Hz, 2H), 7.20 (dd, *J* = 1.6, 7.7 Hz, 1H), 6.98 (dd, *J* = 7.6, 7.6 Hz, 1H), 6.93 (dd, *J* = 1.3, 7.5 Hz, 1H), 4.70 (ddd, *J* = 1.9, 1.9, 14.6 Hz, 1H), 4.52 (s, 2H), 4.48 (ddd, *J* = 1.1, 1.7, 13.0 Hz, 1H), 3.67 (s, 3H), 3.31 (s, 3H), 2.42 (s, 3H), 2.13 (s, 3H), 1.90 (s, 3H), 1.34 (s, 9H); ^{13}C NMR (101 MHz, $CDCl_3$) δ 167.2, 165.5, 151.7, 151.2, 143.9, 141.7, 137.9, 133.70, 133.68, 129.99, 129.95, 129.3, 129.1, 127.6, 125.3, 125.1, 124.6, 114.9, 53.9, 52.7, 52.3, 52.1, 35.3, 30.4, 21.5, 17.5, 16.5; HRMS (ESI) calcd for $C_{31}H_{35}NO_7S$ $[M+Na]^+$ 588.2032, found 588.2019; CHIRALPAK IF-3, *n*-hexane/2-PrOH = 80:20, 1.0 mL/min, retention times: 13.1 min (major isomer) and 19.8 min (minor isomer).

(+)-Dimethyl 6-(2-(*tert*-butyl)-6-methoxy-4-methylphenoxy)-7-methyl-2-tosylisoindoline-4,5-dicarboxylate [(+)-3ab]

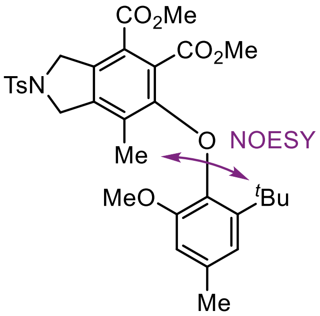

(+)-**3ab** (25.7 mg, 0.0432 mmol, 89% yield, 99% *ee*) and (–)-**4ab** (1.2 mg, 0.0020 mmol, 4% yield, 4% *ee*) were prepared from **1a** (15.4 mg, 0.0482 mmol) and **2b** (27.8 mg, 0.101 mmol) according to the general procedure of **conditions A**. Two regioisomers were isolated by silica gel PTLC (*n*-hexane/EtOAc = 2:1).

Pale yellow solid; mp 110.7–112.7 °C; $[\alpha]^{25}_{D}$ +60.2 (*c* 1.29, $CHCl_3$, 99% *ee*); ^1H NMR (400 MHz, $CDCl_3$) δ 7.78 (d, *J* = 8.3 Hz, 2H), 7.33 (d, *J* = 8.0 Hz, 2H), 6.76 (d, *J* = 1.4 Hz, 1H), 6.53 (d, *J* = 1.6 Hz, 1H), 4.96 (dd, *J* = 2.2, 15.1 Hz, 1H), 4.77 (dd, *J* = 1.5, 15.1 Hz, 1H), 4.59 (dd, *J* = 2.2, 13.7 Hz, 1H), 4.48 (dd, *J* = 1.4, 13.7 Hz, 1H), 3.85 (s, 3H), 3.65 (s, 3H), 3.42 (s, 3H), 2.42 (s, 3H), 2.29 (s, 3H), 1.85 (s, 3H), 1.37 (s, 9H); ^{13}C NMR (101 MHz, $CDCl_3$) δ 167.4, 165.2, 152.8, 150.1, 143.8, 141.6, 139.7, 138.6, 133.9, 132.8, 131.2, 129.9, 127.54, 127.53, 126.4, 120.9, 120.2, 112.4, 56.8, 55.3, 52.9, 52.5, 52.3, 35.2, 30.1, 21.52, 21.48, 14.1; HRMS (ESI) calcd for $C_{32}H_{37}NO_8S$ $[M+Na]^+$ 618.2138, found 618.2132; CHIRALPAK IG-3, *n*-hexane/2-PrOH = 85:15, 1.0 mL/min, retention times: 54.2 min (major isomer) and 63.2 min (minor isomer).

(–)-Dimethyl 5-(2-(*tert*-butyl)-6-methoxy-4-methylphenoxy)-7-methyl-2-tosylisoindoline-4,6-dicarboxylate [(–)-4ab]

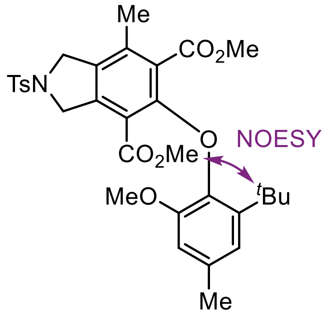

Colorless oil; $[\alpha]^{25}_{D}$ –2.5 (*c* 0.06, $CHCl_3$, 4% *ee*); ^1H NMR (400 MHz, $CDCl_3$) δ 7.76 (d, *J* = 8.3 Hz, 2H), 7.32 (d, *J* = 7.9 Hz, 2H), 6.74 (d, *J* = 1.4 Hz, 1H), 6.52 (d, *J* = 1.4 Hz, 1H), 4.81 (dd, *J* = 2.2, 14.7 Hz, 1H), 4.58–4.48 (m, 3H), 3.63 (s, 3H), 3.45 (s, 3H), 3.33 (s, 3H), 2.42 (s, 3H), 2.28 (s, 3H), 2.12 (s, 3H), 1.34 (s, 9H); ^{13}C NMR (101 MHz, $CDCl_3$) δ 167.4, 165.7, 152.7, 149.6, 143.8, 140.9, 140.3, 137.6, 133.8, 133.23, 133.19, 129.9, 129.0, 127.6, 125.3, 119.9, 115.1, 112.0, 56.1, 54.2, 52.8, 52.2, 51.8, 35.2, 30.1, 21.54, 21.52, 16.5; HRMS (FD) calcd for $C_{32}H_{37}NO_8S$ $[M]^+$ 595.2240, found 595.2264; CHIRALPAK IF-3, *n*-hexane/2-PrOH = 85:15, 1.0 mL/min, retention times: 24.1 min (minor isomer) and 26.7 min (major isomer).

(+)-Dimethyl 6-((3-(*tert*-butyl)-4',5-dimethyl-[1,1'-biphenyl]-2-yl)oxy)-7-methyl-2-tosylisoindoline-4,5-dicarboxylate [(+)-3ac]

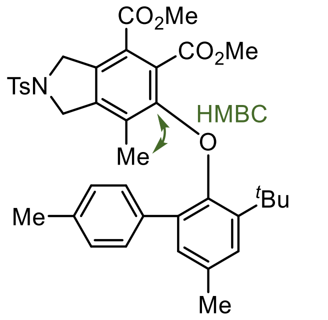

(+)-**3ac** (14.1 mg, 0.0215 mmol, 51% yield, 89% *ee*) and (+)-**4ac** (4.0 mg, 0.0061 mmol, 14% yield, 36% *ee*) were prepared from **1a** (15.8 mg, 0.0495 mmol) and **2c** (33.68 mg, 0.100 mmol) according to the general procedure of **conditions C**. Two regioisomers were isolated by silica gel PTLC (*n*-hexane/EtOAc = 2:1).

Pale yellow solid; mp 128.0–130.0 °C; $[\alpha]^{25}_{D}$ +25.8 (*c* 0.71, $CHCl_3$, 89% *ee*); ^1H NMR (400 MHz, $CDCl_3$) δ 7.74 (d, *J* = 8.3 Hz, 2H), 7.35 (d, *J* = 8.0 Hz, 2H), 7.16 (d, *J* = 2.1 Hz, 1H), 7.04 (d, *J* = 8.0 Hz, 2H), 6.82 (d, *J* = 1.7 Hz, 1H), 6.71 (d, *J* = 7.8 Hz, 2H), 4.60 (s, 2H), 4.26 (d, *J* = 13.7 Hz, 1H), 4.11 (d, *J* = 13.7 Hz, 1H), 3.83 (s, 3H), 3.79 (s, 3H), 2.44 (s, 3H), 2.33 (s, 3H), 2.00 (s, 3H), 1.56 (s, 3H), 1.42 (s, 9H); ^{13}C NMR (101 MHz, $CDCl_3$) δ 167.2, 165.0, 151.1, 149.8, 143.8, 140.1, 139.0, 136.4, 134.9, 133.7, 132.8, 132.0, 130.7, 130.3, 129.9, 128.8, 127.8, 127.5, 127.4, 126.8, 126.4, 121.0, 54.8, 52.6, 52.5, 52.3, 35.3, 30.2, 21.5, 21.0, 20.7, 14.6; HRMS (FD) calcd for $C_{38}H_{41}NO_7S$ $[M]^+$ 655.2604, found 655.2605; CHIRALPAK ID-3, *n*-hexane/2-PrOH = 80:20, 1.0 mL/min, retention times: 24.6 min (major isomer) and 31.3 min (minor isomer).

(+)-Dimethyl 5-((3-(*tert*-butyl)-4',5-dimethyl-[1,1'-biphenyl]-2-yl)oxy)-7-methyl-2-tosylisoindoline-4,6-dicarboxylate [(+)-4ac]

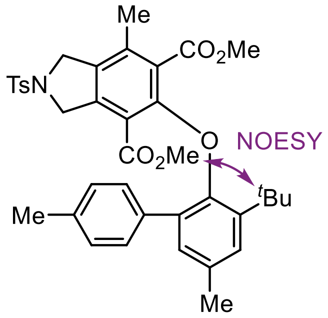

White solid; mp 167.8–169.8 °C; $[\alpha]^{25}_{D}$ +37.6 (*c* 0.17, $CHCl_3$, 36% *ee*); ^{1}H NMR (400 MHz, $CDCl_3$) δ 7.74 (d, *J* = 8.3 Hz, 2H), 7.35 (d, *J* = 8.1 Hz, 2H), 7.14 (d, *J* = 2.0 Hz, 1H), 7.04 (d, *J* = 8.1 Hz, 2H), 6.83 (d, *J* = 1.7 Hz, 1H), 6.70 (d, *J* = 7.9 Hz, 2H), 4.47 (q, *J* = 5.4 Hz, 1H), 4.35 (dd, *J* = 2.8, 12.9 Hz, 1H), 4.30 (dd, *J* = 2.5, 15.3 Hz, 1H), 4.19 (d, *J* = 13.1 Hz, 1H), 3.78 (s, 3H), 3.28 (s, 3H), 2.44 (s, 3H), 2.30 (s, 3H), 1.97 (s, 3H), 1.96 (s, 3H), 1.44 (s, 9H); ^{13}C NMR (101 MHz, $CDCl_3$) δ 167.2, 164.8, 152.4, 150.0, 143.8, 141.0, 138.4, 136.1, 134.9, 133.6, 133.5, 132.9, 130.9, 130.6, 129.9, 129.2, 128.8, 127.63, 127.62, 127.2, 126.1, 114.4, 54.4, 52.3, 52.2, 51.5, 35.5, 30.4, 21.5, 21.0, 20.7, 16.3; HRMS (FD) calcd for $C_{38}H_{41}NO_7S$ $[M]^+$ 655.2604, found 655.2593; CHIRALPAK IE-3, *n*-hexane/2-PrOH = 80:20, 1.0 mL/min, retention times: 23.7 min (major isomer) and 31.6 min (minor isomer).

(+)-Dimethyl 6-(2-(*tert*-butyl)-6-ethoxy-4-methylphenoxy)-7-methyl-2-tosylisoindoline-4,5-dicarboxylate [(+)-3ad]

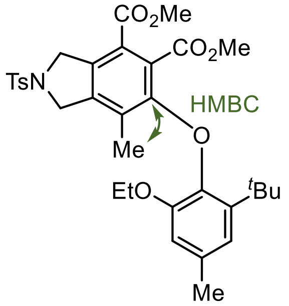

(+)-**3ad** (25.4 mg, 0.0416 mmol, 84% yield, 97% *ee*) was prepared from **1a** (15.8 mg, 0.0495 mmol) and **2d** (29.0 mg, 0.100 mmol) according to the general procedure of **conditions A** and isolated by silica gel PTLC (*n*-hexane/EtOAc = 2:1).

Colorless oil; $[\alpha]^{25}_{D}$ +46.9 (*c* 1.27, $CHCl_3$, 97% *ee*); ^{1}H NMR (400 MHz, $CDCl_3$) δ 7.78 (d, *J* = 8.2 Hz, 2H), 7.32 (d, *J* = 8.0 Hz, 2H), 6.74 (d, *J* = 1.4 Hz, 1H), 6.49 (d, *J* = 1.6 Hz, 1H), 4.99 (dd, *J* = 2.4, 15.2 Hz, 1H), 4.76 (dd, *J* = 1.5, 15.2 Hz, 1H), 4.59 (dd, *J* = 2.4, 13.8 Hz, 1H), 4.47 (dd, *J* = 1.3, 13.7 Hz, 1H), 3.85 (s, 3H), 3.69–3.62 (m, 2H), 3.65 (s, 3H), 2.41 (s, 3H), 2.27 (s, 3H), 1.87 (s, 3H), 1.37 (s, 9H), 0.68 (t, *J* = 7.0 Hz, 3H); ^{13}C NMR (101 MHz, $CDCl_3$) δ 167.3, 165.2, 153.2, 149.1, 143.8, 141.6, 139.4, 138.3, 133.8, 132.4, 131.1, 129.9, 128.1, 127.5, 126.8, 120.7, 119.9, 112.6, 64.4, 55.3, 52.9, 52.5, 52.2, 35.2, 30.0, 21.5, 21.4, 14.12, 14.05; HRMS (FD) calcd for $C_{33}H_{39}NO_8S$ $[M]^+$ 609.2396, found 609.2372; CHIRALPAK IF-3, *n*-hexane/2-PrOH = 90:10, 1.0 mL/min, retention times: 35.9 min (major isomer) and 41.7 min (minor isomer).

(+)-Dimethyl 6-(2-(*tert*-butyl)-6-(methoxymethoxy)-4-methylphenoxy)-7-methyl-2-tosylisoindoline-4,5-dicarboxylate [(+)-3ae]

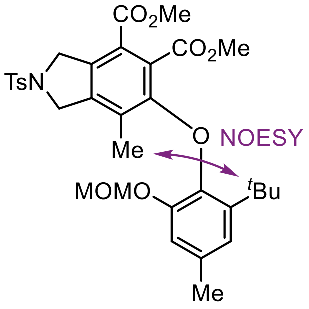

(+)-**3ae** (27.3 mg, 0.0435 mmol, 88% yield, 99% *ee*) was prepared from **1a** (15.7 mg, 0.0492 mmol) and **2e** (29.5 mg, 0.0963 mmol) according to the general procedure of **conditions A** and isolated by silica gel PTLC (*n*-hexane/EtOAc = 2:1).

Colorless oil; $[\alpha]^{25}_{D}$ +41.7 (*c* 0.99, $CHCl_3$, 99% *ee*); ^1H NMR (400 MHz, $CDCl_3$) δ 7.78 (d, *J* = 8.2 Hz, 2H), 7.33 (d, *J* = 7.9 Hz, 2H), 6.81 (d, *J* = 1.5 Hz, 1H), 6.72 (d, *J* = 1.5 Hz, 1H), 4.96 (dd, *J* = 2.1, 15.1 Hz, 1H), 4.78 (dd, *J* = 2.0, 14.1 Hz, 1H), 4.76 (d, *J* = 6.7 Hz, 1H), 4.62 (d, *J* = 6.6 Hz, 1H), 4.55 (dd, *J* = 2.2, 13.8 Hz, 1H), 4.47 (dd, *J* = 1.3, 13.5 Hz, 1H), 3.86 (s, 3H), 3.70 (s, 3H), 3.11 (s, 3H), 2.42 (s, 3H), 2.28 (s, 3H), 1.83 (s, 3H), 1.37 (s, 9H); ^{13}C NMR (101 MHz, $CDCl_3$) δ 167.4, 165.1, 152.8, 147.7, 143.8, 142.1, 139.8, 138.8, 133.8, 133.0, 131.2, 129.9, 127.53, 127.49, 126.8, 121.4, 120.9, 115.2, 95.6, 55.8, 55.3, 52.9, 52.6, 52.3, 35.2, 30.0, 21.5, 21.4, 14.2; HRMS (ESI) calcd for $C_{33}H_{39}NO_9S$ $[M+Na]^+$ 648.2243, found 648.2211; CHIRALPAK IG-3, *n*-hexane/2-PrOH = 85:15, 1.0 mL/min, retention times: 37.4 min (major isomer) and 45.0 min (minor isomer).

(+)-5-Ethyl 4-methyl 6-(2-(*tert*-butyl)-6-methoxy-4-methylphenoxy)-7-methyl-2-tosylisoindoline-4,5-dicarboxylate [(+)-3af]

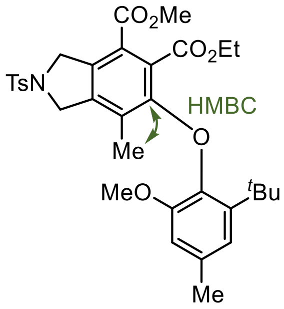

(+)-**3af** (26.3 mg, 0.0432 mmol, 87% yield, 98% *ee*) was prepared from **1a** (15.8 mg, 0.0495 mmol) and **2f** (29.1 mg, 0.100 mmol) according to the general procedure of **conditions A** and isolated by silica gel PTLC (*n*-hexane/EtOAc = 2:1).

Pale yellow oil; $[\alpha]^{25}_{D}$ +65.7 (*c* 1.32, $CHCl_3$, 98% *ee*); ^1H NMR (400 MHz, $CDCl_3$) δ 7.78 (d, *J* = 8.3 Hz, 2H), 7.33 (d, *J* = 7.9 Hz, 2H), 6.76 (d, *J* = 1.4 Hz, 1H), 6.53 (d, *J* = 1.6 Hz, 1H), 4.96 (dd, *J* = 2.2, 15.1 Hz, 1H), 4.78 (dd, *J* = 1.6, 15.2 Hz, 1H), 4.59 (dd, *J* = 2.3, 13.7 Hz, 1H), 4.46 (dd, *J* = 1.5, 13.5 Hz, 1H), 4.20–4.04 (m, 2H), 3.85 (s, 3H), 3.41 (s, 3H), 2.42 (s, 3H), 2.29 (s, 3H), 1.83 (s, 3H), 1.37 (s, 9H), 1.20 (t, *J* = 7.2 Hz, 3H); ^{13}C NMR (101 MHz, $CDCl_3$) δ 166.9, 165.2, 152.9, 150.1, 143.8, 141.9, 139.5, 138.5, 133.9, 132.6, 131.3, 129.9, 127.54, 127.53, 127.0, 120.8, 120.2, 112.5, 61.4, 56.8, 55.3, 52.9, 52.4, 35.2, 30.1, 21.52, 21.46, 14.1, 13.9; HRMS (FD) calcd for $C_{33}H_{39}NO_8S$ $[M]^+$ 609.2396, found 609.2418; CHIRALPAK IE-3, *n*-hexane/2-PrOH = 80:20, 1.0 mL/min, retention times: 48.3 min (major isomer) and 62.7 min (minor isomer).

(+)-5-(*tert*-Butyl) 4-methyl 6-(2-(*tert*-butyl)-6-methoxy-4-methylphenoxy)-7-methyl-2-tosylisoindoline-4,5-dicarboxylate [(+)-3ag]

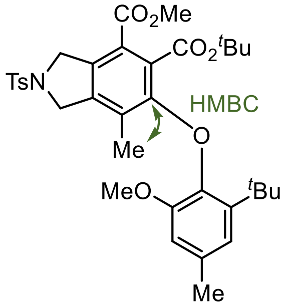

(+)-**3ag** (23.8 mg, 0.0372 mmol, 77% yield, 96% *ee*) was prepared from **1a** (15.6 mg, 0.0488 mmol) and **2g** (31.9 mg, 0.100 mmol) according to the general procedure of **conditions A** and isolated by silica gel PTLC (cyclohexane/EtOAc/Et_2O = 2:1:2).

Colorless oil; $[\alpha]^{25}_{D}$ +102.4 (*c* 0.45, $CHCl_3$, 96% *ee*); ^1H NMR (400 MHz, $CDCl_3$) δ 7.77 (d, *J* = 8.3 Hz, 2H), 7.32 (d, *J* = 8.0 Hz, 2H), 6.77 (d, *J* = 1.5 Hz, 1H), 6.54 (d, *J* = 1.7 Hz, 1H), 4.95 (dd, *J* = 2.4, 15.0 Hz, 1H), 4.78 (dd, *J* = 1.5, 15.1 Hz, 1H), 4.55 (dd, *J* = 2.4, 13.6 Hz, 1H), 4.41 (dd, *J* = 1.3, 13.6 Hz, 1H), 3.87 (s, 3H), 3.43 (s, 3H), 2.41 (s, 3H), 2.30 (s, 3H), 1.72 (s, 3H), 1.42 (s, 9H), 1.38 (s, 9H); ^{13}C NMR (101 MHz, $CDCl_3$) δ 165.8, 165.3, 152.9, 150.3, 143.7, 142.1, 139.4, 138.1, 133.9, 132.6, 131.1, 129.9, 128.8, 127.5, 126.8, 120.5, 120.2, 112.0, 81.8, 56.6, 55.3, 53.0, 52.2, 35.3, 30.3, 28.1, 21.52, 21.51, 14.2; HRMS (FD) calcd for $C_{35}H_{43}NO_8S$ $[M]^+$ 637.2709, found 637.2735; CHIRALPAK IF-3, *n*-hexane/2-PrOH = 80:20, 1.0 mL/min, retention times: 18.0 min (major isomer) and 23.1 min (minor isomer).

(+)-Methyl 5-acetyl-6-(2-(*tert*-butyl)-6-methoxy-4-methylphenoxy)-7-methyl-2-tosylisoindoline-4-carboxylate [(+)-3ah]

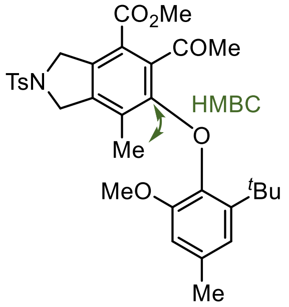

(+)-**3ah** (22.0 mg, 0.0380 mmol, 76% yield, 97% *ee*) was prepared from **1a** (15.9 mg, 0.0498 mmol) and **2h** (26.1 mg, 0.100 mmol) according to the general procedure of **conditions A** and isolated by silica gel PTLC (*n*-hexane/EtOAc = 2:1).

Yellow oil; $[\alpha]^{25}_{D}$ +42.1 (*c* 1.10, $CHCl_3$, 97% *ee*); ^1H NMR (400 MHz, $CDCl_3$) δ 7.78 (d, *J* = 8.2 Hz, 2H), 7.33 (d, *J* = 8.0 Hz, 2H), 6.78 (d, *J* = 1.4 Hz, 1H), 6.56 (d, *J* = 1.6 Hz, 1H), 4.92 (dd, *J* = 2.5, 15.0 Hz, 1H), 4.79 (dd, *J* = 1.2, 15.1 Hz, 1H), 4.57 (dd, *J* = 2.5, 13.5 Hz, 1H), 4.39 (dd, *J* = 0.9, 13.7 Hz, 1H), 3.86 (s, 3H), 3.47 (s, 3H), 2.48 (s, 3H), 2.42 (s, 3H), 2.31 (s, 3H), 2.17 (s, 3H), 1.33 (s, 9H); ^{13}C NMR (101 MHz, $CDCl_3$) δ 203.0, 165.6, 152.1, 150.0, 143.8, 141.5, 139.9, 138.2, 135.8, 133.9, 133.1, 131.1, 129.9, 127.5, 126.5, 120.3, 120.2, 111.2, 56.0, 55.2, 52.9, 52.3, 35.2, 31.0, 30.1, 21.55, 21.52, 14.2; HRMS (FD) calcd for $C_{32}H_{37}NO_7S$ $[M]^+$ 579.2291, found 579.2305; CHIRALPAK IF-3, *n*-hexane/2-PrOH = 80:20, 1.0 mL/min, retention times: 19.1 min (major isomer) and 23.0 min (minor isomer).

(+)-4-Ethyl 5-methyl 6-(2-(*tert*-butyl)-6-methoxy-4-methylphenoxy)-7-methyl-2-tosylisoindoline-4,5-dicarboxylate [(+)-3bb]

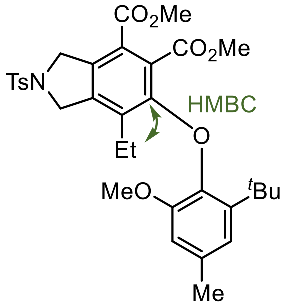

(+)-**3bb** (21.8 mg, 0.0358 mmol, 72% yield, 96% *ee*) was prepared from **1b** (16.5 mg, 0.0495 mmol) and **2b** (27.8 mg, 0.101 mmol) according to the general procedure of **conditions A** and isolated by silica gel PTLC (*n*-hexane/EtOAc = 2:1).

Colorless oil; $[\alpha]^{25}_D$ +38.9 (*c* 1.09, $CHCl_3$, 96% *ee*); ^1H NMR (400 MHz, $CDCl_3$) δ 7.79 (d, *J* = 8.3 Hz, 2H), 7.33 (d, *J* = 8.0 Hz, 2H), 6.74 (d, *J* = 1.4 Hz, 1H), 6.50 (d, *J* = 1.6 Hz, 1H), 4.95 (ddd, *J* = 1.4, 1.4, 15.5 Hz, 1H), 4.72–4.67 (m, 2H), 4.57 (dd, *J* = 2.3, 12.9 Hz, 1H), 3.80 (s, 3H), 3.39 (s, 3H), 3.36 (s, 3H), 2.63–2.45 (m, 2H), 2.42 (s, 3H), 2.28 (s, 3H), 1.38 (s, 9H), 1.03 (t, *J* = 7.5 Hz, 3H); ^{13}C NMR (101 MHz, $CDCl_3$) δ 166.9, 165.4, 152.7, 149.9, 143.8, 141.1, 140.2, 138.1, 134.3, 133.9, 132.7, 131.6, 129.9, 127.5, 124.9, 121.6, 119.8, 111.8, 55.9, 54.9, 52.44, 52.37, 52.0, 35.3, 30.3, 21.6, 21.53, 21.50, 12.7; HRMS (FD) calcd for $C_{33}H_{39}NO_8S$ $[M]^+$ 609.2396, found 609.2416; CHIRALPAK ID-3, *n*-hexane/2-PrOH = 80:20, 1.0 mL/min, retention times: 25.2 min (major isomer) and 37.9 min (minor isomer).

(+)-4-Ethyl 5-methyl 6-(2-(*tert*-butyl)-6-methoxy-4-methylphenoxy)-7-methyl-2-tosylisoindoline-4,5-dicarboxylate [(+)-3cb]

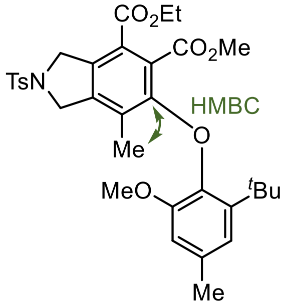

(+)-**3cb** (6.8 mg, 0.0111 mmol, 22% yield, 96% *ee*) was prepared from **1c** (16.7 mg, 0.0501 mmol) and **2b** (27.7 mg, 0.100 mmol) according to the general procedure of **conditions A** and isolated by silica gel PTLC (CH_2Cl_2/EtOAc = 20:1) and then silica gel PTLC (*n*-hexane/CH_2Cl_2/EtOAc = 10:1:10).

Colorless oil; $[\alpha]^{25}_D$ +69.7 (*c* 0.34, $CHCl_3$, 96% *ee*); ^1H NMR (400 MHz, $CDCl_3$) δ 7.78 (d, *J* = 8.3 Hz, 2H), 7.32 (d, *J* = 7.9 Hz, 2H), 6.76 (d, *J* = 1.4 Hz, 1H), 6.54 (d, *J* = 1.6 Hz, 1H), 4.97 (dd, *J* = 2.2, 15.2 Hz, 1H), 4.77 (dd, *J* = 1.5, 15.1 Hz, 1H), 4.59 (dd, *J* = 2.3, 13.6 Hz, 1H), 4.48 (dd, *J* = 1.5, 13.6 Hz, 1H), 4.31 (qd, *J* = 7.1, 1.8 Hz, 2H), 3.65 (s, 3H), 3.42 (s, 3H), 2.42 (s, 3H), 2.29 (s, 3H), 1.84 (s, 3H), 1.37 (s, 9H), 1.35 (t, *J* = 7.0 Hz, 3H); ^{13}C NMR (101 MHz, $CDCl_3$) δ 167.4, 164.7, 152.8, 150.2, 143.8, 141.7, 139.7, 138.6, 133.9, 132.8, 131.3, 129.9, 127.5, 127.3, 126.2, 121.1, 120.2, 112.4, 61.6, 56.8, 55.3, 52.9, 52.2, 35.2, 30.1, 21.52, 21.48, 14.13, 14.13; HRMS (FD) calcd for $C_{33}H_{39}NO_8S$ $[M]^+$ 609.2396, found 609.2400; CHIRALPAK ID-3, *n*-hexane/2-PrOH = 80:20, 1.0 mL/min, retention times: 38.2 min (major isomer) and 51.1 min (minor isomer).

(+)-Methyl 4-acetyl-6-(2-(*tert*-butyl)-6-methoxy-4-methylphenoxy)-7-methyl-2-tosylisoindoline-5-carboxylate [(+)-3db]

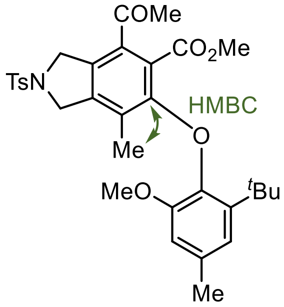

(+)-**3db** (4.6 mg, 0.0079 mmol, 16% yield, 91% *ee*) was prepared from **1d** (15.2 mg, 0.0476 mmol) and **2b** (27.6 mg, 0.100 mmol) according to the general procedure of **conditions A** and isolated by silica gel PTLC (CH_2Cl_2/EtOAc = 20:1) and then silica gel PTLC (*n*-hexane/CH_2Cl_2/EtOAc = 10:1:10).

Colorless oil; $[\alpha]^{25}_{D}$ +60.9 (*c* 0.23, $CHCl_3$, 91% *ee*); ^{1}H NMR (400 MHz, $CDCl_3$) δ 7.78 (d, *J* = 8.3 Hz, 2H), 7.33 (d, *J* = 7.9 Hz, 2H), 6.77 (d, *J* = 1.4 Hz, 1H), 6.53 (d, *J* = 1.6 Hz, 1H), 4.82 (dd, *J* = 2.3, 13.6 Hz, 1H), 4.60 (dd, *J* = 1.6, 11.2 Hz, 1H), 4.57 (dd, *J* = 2.2, 10.9 Hz, 1H), 4.47 (dd, *J* = 1.3, 13.4 Hz, 1H), 3.57 (s, 3H), 3.41 (s, 3H), 2.42 (s, 3H), 2.39 (s, 3H), 2.29 (s, 3H), 1.90 (s, 3H), 1.38 (s, 9H); ^{13}C NMR (101 MHz, $CDCl_3$) δ 199.5, 167.5, 153.2, 149.7, 143.9, 141.7, 139.6, 139.3, 133.7, 132.7, 131.8, 129.9, 128.1, 127.6, 126.2, 123.2, 120.2, 112.3, 56.6, 54.3, 52.8, 52.4, 35.2, 30.1, 29.8, 21.53, 21.47, 14.1; HRMS (FD) calcd for $C_{32}H_{37}NO_7S$ $[M]^+$ 579.2291, found 579.2273; CHIRALPAK IG-3, *n*-hexane/2-PrOH = 80:20, 1.0 mL/min, retention times: 38.9 min (major isomer) and 52.9 min (minor isomer).

(+)-Dimethyl 6-(2-(*tert*-butyl)-6-methoxy-4-methylphenoxy)-7-methyl-1,3-dihydroisobenzofuran-4,5-dicarboxylate [(+)-3eb]

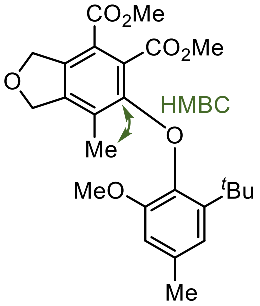

(+)-**3eb** (18.9 mg, 0.0426 mmol, 85% yield, 95% *ee*) and (–)-**4eb** (1.9 mg, 0.00427 mmol, 9% yield, 13% *ee*) were prepared from **1e** (8.3 mg, 0.0499 mmol) and **2b** (27.8 mg, 0.101 mmol) according to the general procedure of **conditions A**. Two regioisomers were isolated by silica gel PTLC (*n*-hexane/ EtOAc = 2:1) and **4eb** was further purified by silica gel PTLC (*n*-hexane/CH_2Cl_2/EtOAc = 2:1:1) and then silica gel PTLC (cyclohexane/ EtOAc = 3:1).

Colorless oil; $[\alpha]^{25}_{D}$ +104.9 (*c* 0.94, $CHCl_3$, 95% *ee*); ^{1}H NMR (400 MHz, $CDCl_3$) δ 6.78 (d, *J* = 1.5 Hz, 1H), 6.56 (d, *J* = 1.7 Hz, 1H), 5.39 (ddd, *J* = 1.8, 1.8, 14.0 Hz, 1H), 5.29 (ddd, *J* = 2.0, 2.0, 14.0 Hz, 1H), 5.07 (ddd, *J* = 2.0, 2.0, 15.0 Hz, 1H), 5.03 (ddd, *J* = 2.2, 2.2, 15.1 Hz, 1H), 3.85 (s, 3H), 3.70 (s, 3H), 3.48 (s, 3H), 2.30 (s, 3H), 1.88 (s, 3H), 1.40 (s, 9H); ^{13}C NMR (101 MHz, $CDCl_3$) δ 167.8, 165.5, 152.7, 150.3, 141.9, 141.6, 139.7, 134.4, 132.6, 126.4, 125.9, 120.2, 119.6, 112.4, 75.6, 72.9, 56.8, 52.3, 52.2, 35.2, 30.1, 21.5, 14.3; HRMS (FD) calcd for $C_{25}H_{30}O_7$ $[M]^+$ 442.1992, found 442.1964; CHIRALPAK IE-3, *n*-hexane/2-PrOH = 80:20, 1.0 mL/min, retention times: 9.5 min (minor isomer) and 11.1 min (major isomer).

(–)-Dimethyl 5-(2-(*tert*-butyl)-6-methoxy-4-methylphenoxy)-7-methyl-1,3-dihydroisobenzofuran-4,6-dicarboxylate [(–)-4eb]

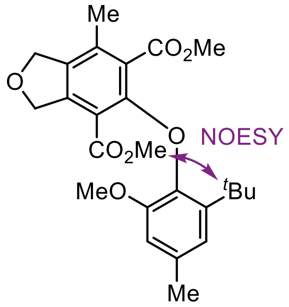

Colorless oil; $[\alpha]^{25}_{D}$ –1.3 (*c* 0.09, $CHCl_3$, 13% *ee*); ^1H NMR (400 MHz, $CDCl_3$) δ 6.77 (d, *J* = 1.4 Hz, 1H), 6.55 (d, *J* = 1.7 Hz, 1H), 5.31 (ddd, *J* = 1.7, 2.9, 14.4 Hz, 1H), 5.10–5.02 (m, 3H), 3.63 (s, 3H), 3.50 (s, 3H), 3.37 (s, 3H), 2.29 (s, 3H), 2.17 (s, 3H), 1.39 (s, 9H); ^{13}C NMR (101 MHz, $CDCl_3$) δ 167.7, 165.9, 153.1, 149.5, 141.5, 140.9, 140.6, 132.7, 132.3, 132.0, 125.0, 119.9, 114.0, 112.2, 74.7, 72.9, 56.3, 52.1, 51.6, 35.2, 30.1, 21.5, 16.7; HRMS (FD) calcd for $C_{25}H_{30}O_7$ $[M]^+$ 442.1992, found 442.1965; CHIRALPAK IG-3, *n*-hexane/2-PrOH = 80:20, 1.0 mL/min, retention times: 6.3 min (minor isomer) and 7.9 min (major isomer).

(+)-Tetramethyl 6-(2-(*tert*-butyl)-6-methoxy-4-methylphenoxy)-7-methyl-1,3-dihydro-2H-indene-2,2,4,5-tetracarboxylate [(+)-3fb]

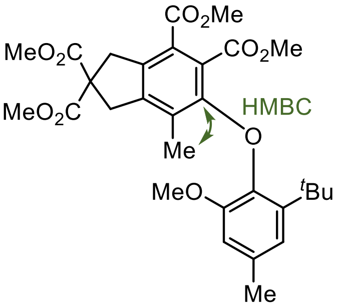

(+)-**3fb** (15.1 mg, 0.0271 mmol, 68% yield, 96% *ee*) was prepared from **1f** (11.2 mg, 0.0400 mmol) and **2b** (22.1 mg, 0.0800 mmol) according to the general procedure of **conditions A** and isolated by silica gel PTLC (*n*-hexane/ EtOAc = 2:1) and then silica gel PTLC ($CHCl_3$/ Et_2O = 3:1).

Pale yellow oil; $[\alpha]^{25}_{D}$ +72.3 (*c* 0.75, $CHCl_3$, 96% *ee*); ^1H NMR (400 MHz, $CDCl_3$) δ 6.77 (d, *J* = 1.5 Hz, 1H), 6.54 (d, *J* = 1.6 Hz, 1H), 3.90 (d, *J* = 18.0 Hz, 1H), 3.85 (s, 3H), 3.83 (d, *J* = 17.9 Hz, 1H), 3.77 (s, 3H), 3.76 (s, 3H), 3.63 (s, 3H), 3.51 (s, 1H), 3.51 (s, 1H), 3.42 (s, 3H), 2.29 (s, 3H), 1.95 (s, 3H), 1.40 (s, 9H); ^{13}C NMR (101 MHz, $CDCl_3$) δ 172.0, 171.9, 167.9, 166.3, 152.5, 150.1, 142.4, 142.0, 139.6, 134.7, 132.2, 128.7, 125.2, 122.2, 120.1, 112.5, 59.4, 56.7, 53.1, 53.0, 52.2, 52.1, 41.7, 39.6, 35.2, 30.1, 21.4, 14.3; HRMS (FD) calcd for $C_{30}H_{36}O_{10}$ $[M]^+$ 556.2309, found 556.2314; CHIRALPAK ID-3, *n*-hexane/2-PrOH = 92:8, 1.0 mL/min, retention times: 26.0 min (major isomer) and 31.2 min (minor isomer).

(+)-Dimethyl 6-(2-((3*r*,5*r*,7*r*)-adamantan-1-yl)-4,6-dimethylphenoxy)-7-methyl-2-tosylisoindoline-4,5-dicarboxylate [(+)-3ai]

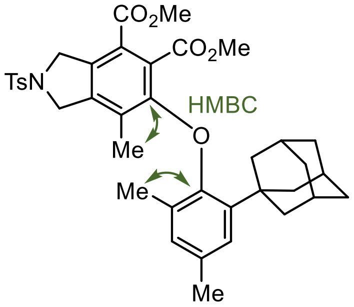

(+)-**3ai** (15.4 mg, 0.0235 mmol, 48% yield, 89% *ee*) and (+)-**4ai** (7.5 mg, 0.0115 mmol, 23% yield, 6% *ee*) were prepared from **1a** (15.8 mg, 0.0495 mmol) and **2i** (32.3 mg, 0.0954 mmol) according to the general procedure of **conditions B**. Two regioisomers were isolated by silica gel PTLC (*n*-hexane/EtOAc = 3:1).

Pale yellow solid; mp 126.7–128.7 °C; $[\alpha]^{25}_{D}$ +73.6 (*c* 0.77, $CHCl_3$, 89% *ee*); ^1H NMR (400 MHz, $CDCl_3$) δ 7.77 (d, *J* = 8.2 Hz, 2H), 7.32 (d, *J* = 8.0 Hz, 2H), 6.94 (d, *J* = 1.9 Hz, 1H), 6.71 (d, *J* = 0.9 Hz, 1H), 4.94 (dd, *J* = 1.6, 15.2 Hz, 1H), 4.80 (dd, *J* = 1.3, 15.3 Hz, 1H), 4.52 (d, *J* = 15.3 Hz, 1H), 4.47 (d, *J* = 14.3 Hz, 1H), 3.87 (s, 3H), 3.81 (s, 3H), 2.42 (s, 3H), 2.27 (s, 3H), 2.17 (s, 3H), 2.11–1.98 (m, 9H), 1.75 (s, 3H), 1.72 (s, 6H); ^{13}C NMR (101 MHz, $CDCl_3$) δ 167.7, 164.8, 151.5, 151.1, 143.8, 140.2, 139.7, 133.9, 133.5, 131.3, 129.9, 129.8, 128.4, 127.7, 127.5, 126.3, 125.8, 121.3, 55.3, 53.1, 52.6, 52.3, 41.0, 37.2, 36.8, 29.0, 21.5, 21.0, 17.6, 14.5; HRMS (ESI) calcd for $C_{38}H_{43}NO_7S$ $[M+Na]^+$ 680.2658, found 680.2652; CHIRALPAK IF-3, *n*-hexane/2-PrOH = 80:20, 1.0 mL/min, retention times: 19.0 min (minor isomer) and 23.9 min (major isomer).

(+)-Dimethyl 6-(2-((3*r*,5*r*,7*r*)-adamantan-1-yl)-4,6-dimethylphenoxy)-7-methyl-2-tosylisoindoline-4,5-dicarboxylate [(+)-4ai]

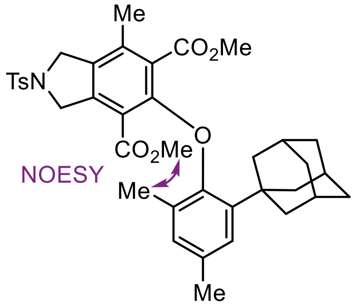

Colorless oil; $[\alpha]^{25}_{D}$ +3.2 (*c* 0.38, $CHCl_3$, 6% *ee*); ^1H NMR (400 MHz, $CDCl_3$) δ 7.75 (d, *J* = 8.2 Hz, 2H), 7.33 (d, *J* = 8.0 Hz, 2H), 6.93 (d, *J* = 1.8 Hz, 1H), 6.72 (d, *J* = 1.3 Hz, 1H), 4.69 (ddd, *J* = 1.9, 1.9, 14.5 Hz, 1H), 4.52 (s, 2H), 4.45 (ddd, *J* = 1.7, 1.7, 14.6 Hz, 1H), 3.69 (s, 3H), 3.27 (s, 3H), 2.42 (s, 3H), 2.25 (s, 3H), 2.13 (s, 3H), 2.07–1.98 (m, 9H), 1.83 (s, 3H), 1.73 (s, 6H); ^{13}C NMR (101 MHz, $CDCl_3$) δ 167.3, 165.5, 151.4, 149.9, 143.9, 141.8, 137.6, 133.8, 133.7, 133.5, 129.99, 129.95, 129.0, 128.6, 127.6, 125.6, 125.4, 114.8, 53.8, 52.8, 52.2, 52.0, 41.1, 37.3, 36.8, 29.1, 21.5, 21.0, 17.4, 16.5; HRMS (FD) calcd for $C_{38}H_{43}NO_7S$ $[M]^+$ 657.2760, found 657.2770; CHIRALPAK IF-3, *n*-hexane/2-PrOH = 80:20, 1.0 mL/min, retention times: 14.2 min (major isomer) and 21.5 min (minor isomer).

(+)-Methyl 4-(3,5-bis(trifluoromethyl)phenyl)-6-(2-(*tert*-butyl)-6-methylphenoxy)-7-methyl-2-tosylisoindoline-5-carboxylate [(+)-3ga]

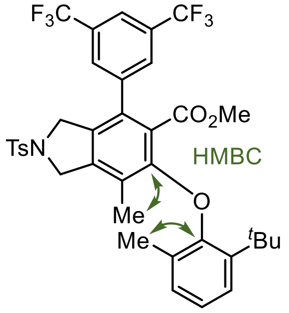

(+)-**3ga** (24.1 mg, 0.0335 mmol, 68% yield, 72% *ee*) was prepared from **1g** (23.5 mg, 0.0496 mmol) and **2a** (25.1 mg, 0.102 mmol) according to the general procedure of **condition B** and isolated by silica gel PTLC (*n*-hexane/EtOAc = 4:1).

Pale yellow solid; mp 72.2–73.9 °C; $[\alpha]^{25}_D$ +32.1 (*c* 1.21, $CHCl_3$, 72% *ee*); ^1H NMR (400 MHz, $CDCl_3$) δ 7.89 (s, 1H), 7.72 (d, *J* = 8.2 Hz, 2H), 7.67 (s, 2H), 7.33 (d, *J* = 8.0 Hz, 2H), 7.22 (dd, *J* = 1.5, 7.7 Hz, 1H), 6.99 (dd, *J* = 7.6, 7.6 Hz, 1H), 6.94 (dd, *J* = 1.1, 7.4 Hz, 1H), 4.63 (d, *J* = 13.6 Hz, 1H), 4.57 (d, *J* = 14.4 Hz, 1H), 4.44 (dd, *J* = 1.2, 13.5 Hz, 1H), 4.28 (dd, *J* = 1.3, 13.3 Hz, 1H), 3.29 (s, 3H), 2.43 (s, 3H), 1.90 (s, 3H), 1.83 (s, 3H), 1.38 (s, 9H); ^{13}C NMR (101 MHz, $CDCl_3$) δ 166.6, 152.6, 151.4, 144.1, 140.7, 139.3, 139.0, 133.6, 132.4 (q, *J* = 33.5 Hz), 130.4, 130.0, 129.8, 129.1, 128.8 (q, *J* = 3.2 Hz), 128.0, 127.5, 125.3, 124.8, 124.4, 123.0 (q, *J* = 274.4 Hz), 122.3, 122.1 (qq, *J* = 3.5, 3.3 Hz), 53.7, 53.1, 51.8, 35.3, 30.3, 21.5, 17.7, 13.9; ^{19}F NMR (377 MHz, $CDCl_3$) δ –62.8; HRMS (ESI) calcd for $C_{37}H_{35}F_6NO_5S$ $[M+Na]^+$ 742.2038, found 742.1972; CHIRALPAK IF-3, *n*-hexane/2-PrOH = 95.5:0.5, 1.0 mL/min, retention times: 39.6 min (major isomer) and 43.4 min (minor isomer).

(+)-Methyl 6-(2-(*tert*-butyl)-6-methylphenoxy)-4,7-dimethyl-2-tosylisoindoline-5-carboxylate [(+)-3ha]

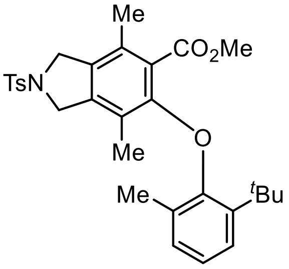

(+)-**3ha** (37.2 mg, 0.0713 mmol, 72% yield, 50% *ee*) was prepared from **1h** (27.8 mg, 0.101 mmol) and **2a** (24.5 mg, 0.0995 mmol) according to the general procedure of **conditions B** except for using (*S*)-Segphos (6.1 mg, 0.010 mmol), $[Rh(cod)_2]BF_4$ (4.1 mg, 0.010 mmol), and CH_2Cl_2 (2.0 mL) and isolation by silica gel PTLC (*n*-hexane/EtOAc = 2:1).

Brown oil; $[\alpha]^{25}_D$ +36.5 (*c* 1.86, $CHCl_3$, 50% *ee*); ^1H NMR (400 MHz, $CDCl_3$) δ 7.77 (d, *J* = 8.3 Hz, 2H), 7.33 (d, *J* = 7.9 Hz, 2H), 7.20 (dd, *J* = 1.6, 7.7 Hz, 1H), 6.97 (dd, *J* = 7.6, 7.6 Hz, 1H), 6.92 (dd, *J* = 1.3, 7.4 Hz, 1H), 4.61–4.47 (m, 4H), 3.63 (s, 3H), 2.42 (s, 3H), 2.07 (s, 3H), 1.84 (s, 3H), 1.68 (s, 3H), 1.36 (s, 9H); ^{13}C NMR (101 MHz, $CDCl_3$) δ 168.1, 152.9, 151.0, 143.8, 140.4, 138.0, 133.8, 129.9, 129.8, 129.1, 128.8, 128.1, 127.5, 125.2, 125.1, 123.8, 118.7, 53.8, 53.3, 52.0, 35.2, 30.3, 21.5, 17.7, 16.0, 13.6; HRMS (ESI) calcd for $C_{30}H_{35}NO_5S$ $[M+Na]^+$ 544.2134, found 544.2134; CHIRALPAK IG-3, *n*-hexane/2-PrOH = 95:5, 1.0 mL/min, retention times: 42.9 min (minor isomer) and 49.2 min (major isomer).

(–)-Methyl 6-(2-(*tert*-butyl)-6-methylphenoxy)-4,7-diphenyl-2-tosylisoindoline-5-carboxylate [(–)-3ia]

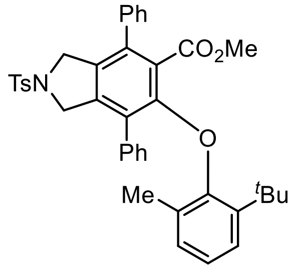

(–)-**3ia** (53.0 mg, 0.0820 mmol, 83% yield, 45% *ee*) was prepared from **1i** (40.2 mg, 0.101 mmol) and **2a** (24.4 mg, 0.0991 mmol) according to the general procedure of **conditions B** except for using (*S*)-Segphos (6.1 mg, 0.010 mmol), $[Rh(cod)_2]BF_4$ (4.1 mg, 0.010 mmol) and CH_2Cl_2 (2.0 mL) and isolation by silica gel PTLC (*n*-hexane/EtOAc = 4:1).

Brown oil; $[\alpha]^{25}_{D}$ –16.5 (*c* 2.65, $CHCl_3$, 45% *ee*); ^{1}H NMR (400 MHz, $CDCl_3$) δ 7.64 (d, *J* = 8.2 Hz, 2H), 7.39–7.25 (m, 10H), 7.16 (d, *J* = 1.8 Hz, 1H), 7.14 (d, *J* = 1.9 Hz, 1H), 7.04 (dd, *J* = 1.7, 7.6 Hz, 1H), 6.83 (dd, *J* = 7.5, 7.5 Hz, 1H), 6.79 (dd, *J* = 1.5, 7.5 Hz, 1H), 4.54 (dd, *J* = 1.4, 13.5 Hz, 1H), 4.49 (dd, *J* = 1.5, 14.4 Hz, 1H), 4.34 (dd, *J* = 1.2, 14.3 Hz, 1H), 4.25 (dd, *J* = 1.2, 13.5 Hz, 1H), 2.81 (s, 3H), 2.41 (s, 3H), 1.98 (s, 3H), 1.21 (s, 9H); ^{13}C NMR (101 MHz, $CDCl_3$) δ 166.1, 151.8, 150.5, 143.7, 141.6, 138.4, 137.0, 135.9, 135.2, 133.8, 129.8, 129.7, 129.5, 129.1, 128.5, 128.4, 128.31, 128.26, 128.0, 127.8, 127.6, 127.2, 124.9, 124.3, 122.6, 53.9, 53.6, 51.3, 35.2, 30.5, 21.6, 18.1; HRMS (ESI) calcd for $C_{40}H_{39}NO_5S$ $[M+Na]^+$ 668.2447, found 668.2441; CHIRALPAK ID-3, *n*-hexane/2-PrOH = 95:5, 1.0 mL/min, retention times: 22.8 min (minor isomer) and 25.5 min (major isomer).

(–)-Methyl 6-(2-(*tert*-butyl)-6-methylphenoxy)-4,7-bis(4-chlorophenyl)-2-tosylisoindoline-5-carboxylate [(–)-3ja]

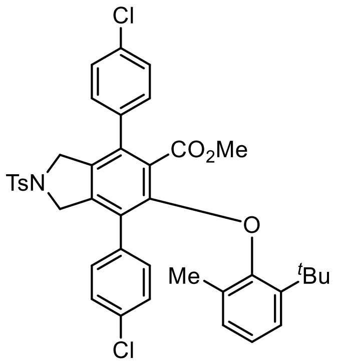

(–)-**3ja** (41.9 mg, 0.0586 mmol, 59% yield, 55% *ee*) was prepared from **1j** (46.8 mg, 0.0999 mmol) and **2a** (24.6 mg, 0.0999 mmol) according to the general procedure of **conditions B** except for using (*S*)-Segphos (6.1 mg, 0.010 mmol), $[Rh(cod)_2]BF_4$ (4.1 mg, 0.010 mmol), and CH_2Cl_2 (2.0 mL) and isolation by silica gel PTLC (*n*-hexane/EtOAc = 10:1 twice).

Colarless oil; $[\alpha]^{25}_{D}$ –17.8 (*c* 1.72, $CHCl_3$, 55% *ee*); ^{1}H NMR (400 MHz, $CDCl_3$) δ 7.65 (d, *J* = 8.2 Hz, 2H), 7.35 (d, *J* = 8.5 Hz, 2H), 7.31 (dd, *J* = 8.7, 8.7 Hz, 4H), 7.18 (d, *J* = 8.4 Hz, 2H), 7.09 (d, *J* = 8.1 Hz, 2H), 7.06 (dd, *J* = 1.5, 7.7 Hz, 1H), 6.87 (dd, *J* = 7.6, 7.6 Hz, 1H), 6.80 (dd, *J* = 1.0, 7.6 Hz, 1H), 4.48 (dd, *J* = 1.4, 13.2 Hz, 1H), 4.45 (dd, *J* = 1.3, 13.2 Hz, 1H), 4.30 (dd, *J* = 1.5, 14.4 Hz, 1H), 4.21 (dd, *J* = 1.4, 13.6 Hz, 1H), 2.86 (s, 3H), 2.42 (s, 3H), 1.96 (s, 3H), 1.21 (s, 9H); ^{13}C NMR (101 MHz, $CDCl_3$) δ 165.9, 151.6, 150.7, 143.9, 141.8, 138.6, 130.6, 130.0, 129.9, 129.8, 129.7, 128.9, 128.8, 127.7, 126.3, 125.2, 124.7, 122.8, 53.9, 53.5, 51.7, 35.4, 30.7, 21.7, 18.1; HRMS (FD) calcd for $C_{40}H_{37}Cl_2NO_5S$ $[M]^+$ 713.1764, found 713.1774; CHIRALPAK IE-3, *n*-hexane/2-PrOH = 98:2, 1.0 mL/min, retention times: 61.5 min (minor isomer) and 67.8 min (major isomer).

(–)-Methyl 6-(2-(*tert*-butyl)-6-methylphenoxy)-4,7-di(thiophen-3-yl)-2-tosylisoindoline-5-carboxylate [(–)-3ka]

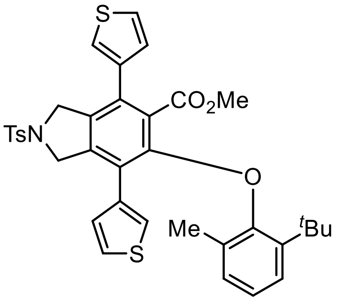

(–)-**3ka** (55.4 mg, 0.0842 mmol, 84% yield, 48% *ee*) was prepared from **1k** (41.2 mg, 0.100 mmol) and **2a** (24.6 mg, 0.0999 mmol) according to the general procedure of **conditions B** except for using (*S*)-Segphos (6.1 mg, 0.010 mmol), $[Rh(cod)_2]BF_4$ (4.1 mg, 0.010 mmol) and CH_2Cl_2 (2.0 mL) and isolation by silica gel PTLC (*n*-hexane/EtOAc = 5:1).

White solid; decomp at 99.3 °C; $[\alpha]^{25}_D$ –14.6 (*c* 2.04, $CHCl_3$, 48% *ee*); ^1H NMR (400 MHz, $CDCl_3$) δ 7.67 (d, *J* = 8.2 Hz, 2H), 7.35–7.28 (m, 3H), 7.26 (dd, *J* = 2.9, 5.0 Hz, 1H), 7.14 (dd, *J* = 1.3, 3.0 Hz, 1H), 7.08 (dd, *J* = 1.3, 3.0 Hz, 1H), 7.07–7.03 (m, 1H), 6.97 (dd, *J* = 1.2, 4.8 Hz, 1H), 6.95 (dd, *J* = 1.3, 4.7 Hz, 1H), 6.86 (dd, *J* = 7.5, 7.5 Hz, 1H), 6.81 (dd, *J* = 1.3, 7.1 Hz, 1H), 4.57 (dd, *J* = 1.4, 13.3 Hz, 1H), 4.49 (dd, *J* = 1.5, 14.1 Hz, 1H), 4.40 (m, *J* = 4.2 Hz, 1H), 4.35 (dd, *J* = 1.1, 13.5 Hz, 1H), 3.03 (s, 3H), 2.42 (s, 3H), 1.99 (s, 3H), 1.22 (s, 9H); ^{13}C NMR (101 MHz, $CDCl_3$) δ 166.6, 152.0, 151.0, 143.9, 141.6, 139.0, 136.7, 134.4, 133.9, 131.0, 130.0, 129.74, 129.71, 129.1, 128.5, 127.8, 127.7, 125.9, 125.4, 125.1, 124.5, 124.2, 124.0, 123.7, 122.4, 54.2, 53.8, 51.8, 35.3, 30.6, 21.7, 18.2; HRMS (FD) calcd for $C_{36}H_{35}NO_5S_3$ $[M]^+$ 657.1672, found 657.1619; CHIRALPAK IG-3, *n*-hexane/2-PrOH = 95:5, 1.0 mL/min, retention times: 29.4 min (minor isomer) and 35.0 min (major isomer).

(+)-Trimethyl 6-(2-(*tert*-butyl)-6-methylphenoxy)-2-tosylisoindoline-4,5,7-tricarboxylate [(+)-3la]

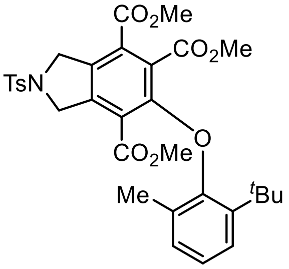

(+)-**3la** (15.1 mg, 0.0248 mmol, 50% yield, 43% *ee*) was prepared from **1l** (18.0 mg, 0.0495 mmol) and **2a** (12.5 mg, 0.0507 mmol) according to the general procedure of **condition B** except for using (*S*)-Segphos (3.1 mg, 0.0050 mmol), $[Rh(cod)_2]BF_4$ (2.0 mg, 0.0050 mmol) and CH_2Cl_2 (2.0 mL) and isolation by silica gel PTLC (*n*-hexane/EtOAc = 2:1).

Pale brown oil; $[\alpha]^{25}_D$ +12.3 (*c* 0.76, $CHCl_3$, 43% *ee*); ^1H NMR (400 MHz, $CDCl_3$) δ 7.73 (d, *J* = 8.3 Hz, 2H), 7.31 (d, *J* = 7.9 Hz, 2H), 7.22 (dd, *J* = 1.4, 7.8 Hz, 1H), 7.01 (dd, *J* = 7.6, 7.6 Hz, 1H), 6.94 (dd, *J* = 1.1, 7.5 Hz, 1H), 4.83 (dd, *J* = 1.8, 1.8 Hz, 2H), 4.61 (ddd, *J* = 2.0, 2.0, 14.8 Hz, 1H), 4.39 (ddd, *J* = 1.8, 1.8, 14.8 Hz, 1H), 3.91 (s, 3H), 3.83 (s, 3H), 3.22 (s, 3H), 2.41 (s, 3H), 1.89 (s, 3H), 1.34 (s, 9H); ^{13}C NMR (101 MHz, $CDCl_3$) δ 166.5, 164.5, 164.2, 151.3, 150.8, 144.0, 142.1, 139.0, 133.6, 131.4, 129.99, 129.98, 129.3, 127.54, 127.52, 125.31, 125.27, 125.2, 120.5, 54.7, 53.0, 52.8, 52.7, 52.4, 35.3, 30.4, 21.5, 17.4; HRMS (ESI) calcd for $C_{32}H_{35}NO_9S$ $[M+Na]^+$ 632.1930, found 632.1925; CHIRALPAK IF-3, *n*-hexane/2-PrOH = 85:15, 1.0 mL/min, retention times: 19.9 min (major isomer) and 25.6 min (minor isomer).

(+)-Methyl 6-(2-(*tert*-butyl)-6-methoxy-4-methylphenoxy)-4,7-dimethyl-2-tosylisoindoline-5-carboxylate [(+)-3hb]

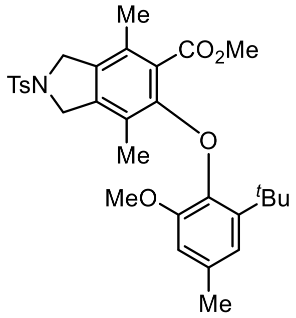

(+)-**3hb** (20.7 mg, 0.0376 mmol, 78% yield, 66% *ee*) was prepared from **1h** (13.5 mg, 0.0490 mmol) and **2b** (13.9 mg, 0.0503 mmol) according to the general procedure of **conditions A** and isolated by silica gel PTLC (*n*-hexane/EtOAc/Et_2O = 3:1:1).

Colorless oil; $[\alpha]^{25}_{D}$ +28.4 (*c* 1.04, $CHCl_3$, 66% *ee*); ^{1}H NMR (400 MHz, $CDCl_3$) δ 7.78 (d, *J* = 8.2 Hz, 2H), 7.33 (d, *J* = 8.0 Hz, 2H), 6.75 (d, *J* = 1.4 Hz, 1H), 6.54 (d, *J* = 1.6 Hz, 1H), 4.62 (d, *J* = 13.2 Hz, 1H), 4.61 (d, *J* = 12.8 Hz, 1H), 4.49 (dd, *J* = 3.7, 13.3 Hz, 1H), 4.48 (dd, *J* = 3.5, 13.6 Hz, 1H), 3.50 (s, 3H), 3.45 (s, 3H), 2.42 (s, 3H), 2.29 (s, 3H), 2.05 (s, 3H), 1.86 (s, 3H), 1.36 (s, 9H); ^{13}C NMR (101 MHz, $CDCl_3$) δ 168.1, 152.5, 150.6, 143.7, 141.4, 140.3, 137.1, 134.0, 132.7, 129.9, 128.2, 127.5, 127.4, 124.1, 120.0, 119.4, 112.3, 56.5, 53.8, 53.4, 51.8, 35.2, 30.2, 21.52, 21.51, 16.0, 13.5; HRMS (FD) calcd for $C_{31}H_{37}NO_6S$ $[M]^+$ 551.2342, found 551.2310; CHIRALPAK IE-3, *n*-hexane/2-PrOH = 80:20, 1.0 mL/min, retention times: 16.8 min (major isomer) and 18.5 min (minor isomer).

(+)-Dimethyl 6-((3-(*tert*-butyl)-4'-methoxy-5-methyl-[1,1'-biphenyl]-2-yl)oxy)-7-methyl-2-tosylisoindoline-4,5-dicarboxylate [(+)-3aj]

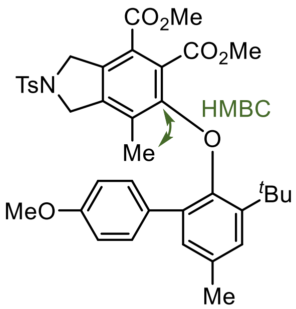

(+)-**3aj** (14.6 mg, 0.0217 mmol, 44% yield, 91% *ee*) was prepared from **1a** (15.6 mg, 0.0488 mmol) and **2j** (35.5 mg, 0.101 mmol) according to the general procedure of **conditions C** and isolated by silica gel PTLC (*n*-hexane/EtOAc = 3:1).

Pale yellow solid; mp 86.6–88.6 °C; $[\alpha]^{25}_{D}$ +47.0 (*c* 0.73, $CHCl_3$, 91% *ee*); ^{1}H NMR (400 MHz, $CDCl_3$) δ 7.72 (d, *J* = 8.3 Hz, 2H), 7.33 (d, *J* = 8.1 Hz, 2H), 7.15 (d, *J* = 2.1 Hz, 1H), 7.12 (d, *J* = 8.7 Hz, 2H), 6.84 (d, *J* = 1.7 Hz, 1H), 6.49 (d, *J* = 8.7 Hz, 2H), 4.66 (dd, *J* = 1.7, 15.0 Hz, 1H), 4.59 (dd, *J* = 1.5, 14.8 Hz, 1H), 4.25 (dd, *J* = 1.4, 13.7 Hz, 1H), 4.16 (dd, *J* = 1.5, 13.7 Hz, 1H), 3.83 (s, 3H), 3.81 (s, 3H), 3.60 (s, 3H), 2.43 (s, 3H), 2.33 (s, 3H), 1.55 (s, 3H), 1.41 (s, 9H); ^{13}C NMR (101 MHz, $CDCl_3$) δ 167.3, 165.0, 158.5, 151.0, 149.7, 143.8, 140.2, 139.1, 133.7, 132.9, 131.8, 130.7, 130.3, 130.1, 130.0, 129.9, 127.4, 127.3, 126.8, 126.1, 121.1, 112.8, 55.2, 54.8, 52.6, 52.5, 52.4, 35.3, 30.2, 21.5, 21.0, 14.6; HRMS (FD) calcd for $C_{38}H_{41}NO_8S$ $[M]^+$ 671.2553, found 671.2565; CHIRALPAK IF-3, *n*-hexane/2-PrOH = 80:20, 1.0 mL/min, retention times: 15.9 min (major isomer) and 21.9 min (minor isomer).

(+)-Dimethyl 6-((3-(*tert*-butyl)-5-methyl-4'-(trifluoromethyl)-[1,1'-biphenyl]-2-yl)oxy)-7-methyl-2-tosylisoindoline-4,5-dicarboxylate [(+)-3ak]

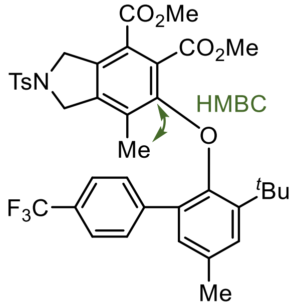

(+)-**3ak** (8.0 mg, 0.0113 mmol, 23% yield, 93% *ee*) was prepared from **1a** (15.9 mg, 0.0498 mmol) and **2k** (39.0 mg, 0.100 mmol) according to the general procedure of **conditions C** and isolated by silica gel PTLC (*n*-hexane/EtOAc = 3:1).

Pale yellow solid; mp 89.6–91.3 °C; $[\alpha]^{25}_{D}$ +33.3 (*c* 0.40, $CHCl_3$, 93% *ee*); ^1H NMR (400 MHz, $CDCl_3$) δ 7.73 (d, *J* = 8.3 Hz, 2H), 7.35 (d, *J* = 8.0 Hz, 2H), 7.27 (d, *J* = 8.0 Hz, 2H), 7.23 (dd, *J* = 0.4, 2.2 Hz, 1H), 7.11 (d, *J* = 8.0 Hz, 2H), 6.81 (dd, *J* = 0.6, 2.2 Hz, 1H), 4.65 (dd, *J* = 2.3, 15.0 Hz, 1H), 4.49 (dd, *J* = 1.8, 15.1 Hz, 1H), 4.34 (dd, *J* = 2.5, 13.8 Hz, 1H), 4.05 (dd, *J* = 1.8, 13.7 Hz, 1H), 3.83 (s, 3H), 3.80 (s, 3H), 2.44 (s, 3H), 2.34 (s, 3H), 1.57 (s, 3H), 1.43 (s, 9H); ^{13}C NMR (101 MHz, $CDCl_3$) δ 167.0, 164.7, 151.1, 150.0, 143.9, 141.5, 140.4, 139.3, 133.5, 133.1, 131.1, 130.6, 129.9, 129.8, 129.4, 128.7 (q, *J* = 32.4 Hz), 128.5, 127.4, 127.0, 126.2, 123.8 (q, *J* = 265.0 Hz),123.9 (q, *J* = 3.8 Hz), 121.4, 54.7, 52.58, 52.57, 52.4, 35.4, 30.2, 21.5, 21.0, 14.5; ^{19}F NMR (377 MHz, $CDCl_3$) δ −62.8; HRMS (FD) calcd for $C_{38}H_{38}F_3NO_7S$ $[M]^+$ 709.2321, found 709.2349; CHIRALPAK IF-3, *n*-hexane/2-PrOH = 80:20, 1.0 mL/min, retention times: 12.6 min (major isomer) and 18.4 min (minor isomer).

(+)-Dimethyl 6-((3-(*tert*-butyl)-2',5-dimethyl-[1,1'-biphenyl]-2-yl)oxy)-7-methyl-2-tosylisoindoline-4,5-dicarboxylate [(+)-3al]

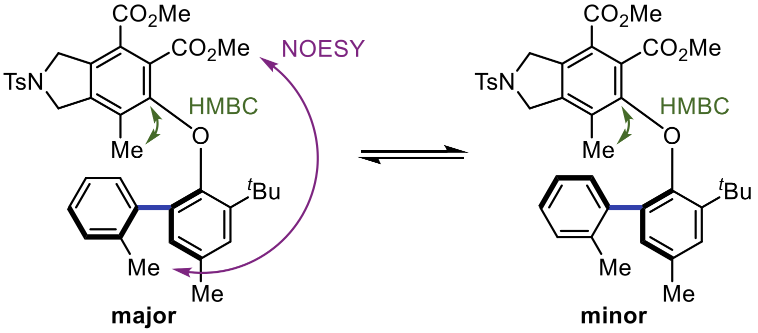

(+)-**3al** (8.0 mg, 0.0122 mmol, 25% yield, 93% *ee*) was prepared from **1a** (15.5 mg, 0.0485 mmol) and **2l** (34.1 mg, 0.101 mmol) according to the general procedure of **conditions C** and isolated by silica gel PTLC (*n*-hexane/EtOAc = 3:1). The rotamers were observed on the NMR timescale (major/minor = 51:49).

Colorless oil; $[\alpha]^{25}_{D}$ +8.0 (*c* 0.40, $CHCl_3$, 93% *ee*); ^1H NMR (400 MHz, $CDCl_3$) δ 7.76 (d, *J* = 7.7 Hz, major: 2H, minor: 2H), 7.38 (d, *J* = 7.1 Hz, minor: 2H), 7.36 (d, *J* = 7.7 Hz, major: 2H), 7.18 (d, *J* = 2.0 Hz, major: 1H), 7.14 (d, *J* = 2.0 Hz, minor: 1H), 6.96 (d, *J* = 7.9 Hz, minor 1H), 6.94 (dd, *J* = 1.4, 7.8 Hz, major: 1H), 6.83–6.78 (m, major: 1H, minor: 1H), 6.71 (d, *J* = 1.7 Hz, major: 1H), 6.66 (d, *J* = 1.6 Hz, minor: 1H), 6.62–6.56 (m, major: 1H, minor: 1H), 6.52 (ddd, *J* = 0.8, 7.5, 7.5 Hz, minor: 1H), 6.39 (ddd, *J* = 0.5, 7.5, 7.5 Hz, major: 1H), 4.74 (d, *J* = 15.2 Hz, major: 1H), 4.73 (d, *J* = 15.0 Hz, minor: 1H), 4.57 (dd, *J* = 2.2, 15.1 Hz, major: 1H), 4.56 (ddd, *J* = 1.8, 1.8, 15.0 Hz, minor: 1H), 4.35 (s, minor: 2H), 4.29 (dd, *J* = 2.3, 13.8 Hz, major: 1H), 4.14 (dd, *J* = 2.3, 13.5 Hz, major: 1H), 3.79 (s, major: 3H, minor: 3H), 3.77 (s, major: 3H), 3.40 (s, minor: 3H), 2.45 (s, minor: 3H), 2.43 (s, major: 3H), 2.34 (s, major: 3H), 2.30 (s, minor: 3H), 1.96 (s, minor: 3H), 1.89 (s, major: 3H), 1.78 (s, minor: 3H), 1.58 (s, major: 3H), 1.44 (s, minor: 9H), 1.41 (s, major: 9H); ^{13}C NMR (101

MHz, $CDCl_3$) δ 166.5, 166.3, 165.4, 165.0, 152.7, 152.2, 150.9, 150.6, 143.89, 143.86, 140.7, 140.2, 139.4, 138.4, 137.9, 137.4, 136.9, 135.5, 133.7, 133.6, 132.9, 132.8, 132.1, 130.9, 130.2, 130.0, 129.89, 129.88, 129.81, 129.79, 129.5, 129.0, 128.7, 127.9, 127.8, 127.58, 127.56, 127.5, 127.15, 127.08, 126.3, 125.8, 125.0, 124.8, 124.1, 122.1, 121.5, 54.9, 53.1, 52.54, 52.45, 52.4, 52.2, 52.0, 35.7, 35.2, 30.7, 30.1, 21.54, 21.53, 21.1, 21.0, 20.22, 20.17, 15.1, 13.8; HRMS (FD) calcd for $C_{38}H_{41}NO_7S$ $[M]^+$ 655.2604, found 655.2625; CHIRALPAK IG-3, *n*-hexane/2-PrOH = 80:20, 1.0 mL/min, retention times: 18.6 min (major isomer) and 28.1 min (minor isomer).

(+)-Methyl 6-((3-(*tert*-butyl)-4',5-dimethyl-[1,1'-biphenyl]-2-yl)oxy)-7-methyl-4-(4-nitrophenyl)-2-tosylisoindoline-5-carboxylate [(+)-3mc]

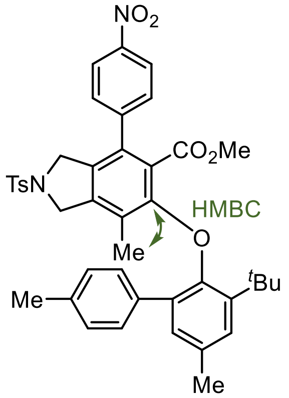

(+)-**3mc** (29.0 mg, 0.0403 mmol, 81% yield, 75% *ee*) was prepared from **1m** (19.0 mg, 0.0497 mmol) and **2c** (33.5 mg, 0.100 mmol) according to the general procedure of **conditions C** and isolated by silica gel PTLC (*n*-hexane/EtOAc = 4:1).

Pale brown solid; mp 115.3–117.0 °C; $[\alpha]^{25}_{D}$ +111.1 (*c* 1.45, $CHCl_3$, 75% *ee*); ^1H NMR (400 MHz, $CDCl_3$) δ 8.23 (d, *J* = 8.7 Hz, 2H), 7.68 (d, *J* = 8.2 Hz, 2H), 7.34 (d, *J* = 8.1 Hz, 2H), 7.20 (d, *J* = 8.7 Hz, 2H), 7.16 (d, *J* = 2.1 Hz, 1H), 7.04 (d, *J* = 8.0 Hz, 2H), 6.81–6.78 (m, 3H), 4.35 (dd, *J* = 1.2, 13.6 Hz, 1H), 4.25 (dd, *J* = 1.4, 13.4 Hz, 1H), 4.13 (dd, *J* = 1.6, 13.1 Hz, 1H), 4.04 (dd, *J* = 1.4, 13.1 Hz, 1H), 3.33 (s, 3H), 2.44 (s, 3H), 2.31 (s, 3H), 2.10 (s, 3H), 1.71 (s, 3H), 1.45 (s, 9H); ^{13}C NMR (101 MHz, $CDCl_3$) δ 166.5, 151.6, 150.0, 147.5, 144.2, 143.9, 140.3, 138.5, 136.2, 135.5, 133.4, 132.8, 132.1, 130.7, 130.4, 129.9, 129.1, 129.0, 127.7, 127.5, 127.4, 127.1, 124.5, 123.7, 122.4, 53.3, 52.9, 51.8, 35.4, 30.4, 21.5, 21.0, 20.9, 14.1; HRMS (FD) calcd for $C_{42}H_{42}N_2O_7S$ $[M]^+$ 718.2713, found 718.2736; CHIRALPAK IE-3, *n*-hexane/2-PrOH = 80:20, 1.0 mL/min, retention times: 19.5 min (major isomer) and 28.6 min (minor isomer).

(*S*)-(+)-Methyl 4-(3,5-bis(trifluoromethyl)phenyl)-6-((3-(*tert*-butyl)-4',5-dimethyl-[1,1'-biphenyl]-2-yl)oxy)-7-methyl-2-tosylisoindoline-5-carboxylate [(*S*)-(+)-3gc]

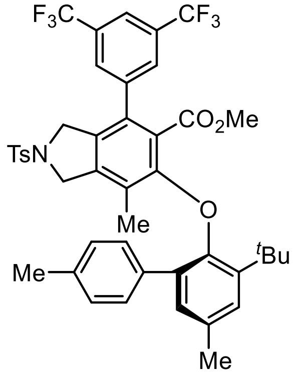

(*S*)-(+)-**3gc** (37.3 mg, 0.0460 mmol, 93% yield, 92% *ee*) was prepared from **1g** (23.5 mg, 0.0496 mmol) and **2c** (33.9 mg, 0.101 mmol) according to the general procedure of **conditions C** and isolated by silica gel PTLC (*n*-hexane/EtOAc = 4:1).

Pale brown solid; mp 78.3–80.3 °C; $[\alpha]^{25}_{D}$ +50.3 (*c* 0.25, $CHCl_3$, 92% *ee*); ^1H NMR (400 MHz, $CDCl_3$) δ 7.87 (s, 1H), 7.69 (d, *J* = 8.3 Hz, 2H), 7.45 (s, 2H), 7.36 (d, *J* = 7.9 Hz, 2H), 7.16 (dd, *J* = 0.5, 2.3 Hz, 1H), 7.01 (d, *J* = 8.0 Hz, 2H), 6.80 (dd, *J* = 0.6, 2.2 Hz, 1H), 6.76 (d, *J* = 7.8 Hz, 2H), 4.38 (dd, *J* = 1.2, 13.8 Hz, 1H), 4.27 (dd, *J* = 0.7, 14.1 Hz, 1H), 4.11 (dd, *J* = 1.2, 13.2 Hz, 1H), 4.05 (dd, *J* = 1.2, 13.3 Hz, 1H), 3.37 (s, 3H), 2.45 (s, 3H), 2.31 (s, 3H), 2.10 (s, 3H), 1.70 (s, 3H), 1.45 (s, 9H); ^{13}C NMR (101 MHz, $CDCl_3$) δ 166.3, 151.6, 150.0, 144.0, 140.2, 139.4, 138.8, 136.2, 135.4, 133.3, 132.8, 132.1, 131.8 (q, *J* = 33.6 Hz), 130.4, 130.0, 129.7, 129.0, 128.35 (q, *J* = 2.7 Hz), 127.7, 127.5, 127.4, 127.3, 125.0, 123.1 (q, *J* = 266.3 Hz), 122.5, 121.8–121.7 (m), 53.4, 52.9, 51.7, 35.4, 30.3, 21.5, 21.0, 20.7, 14.1; ^{19}F NMR (377 MHz, $CDCl_3$) δ −62.9; HRMS (FD) calcd for $C_{44}H_{41}F_6NO_5S$ $[M]^+$ 809.2610, found 809.2615; CHIRALPAK IB, *n*-hexane/2-PrOH = 99:1, 1.0 mL/min, retention times: 9.1 min (major isomer) and 25.2 min (minor isomer).

2.3. Rh-Catalyzed Enantio- and Diastereoselective Synthesis of Axially Chiral Diaryl Ethers

General Procedure of Conditions A1 (Figure 3): (*S*)-Difluorphos (3.4 mg, 0.0050 mmol) and $[Rh(cod)_2]BF_4$ (2.0 mg, 0.0050 mmol) were dissolved in CH_2Cl_2 (1.0 mL) in a Schlenk tube, and the mixture was stirred at room temperature for 10 min. After introduction of H_2 and stirring at room temperature for 30 min, the resulting mixture was concentrated to dryness. The residue was dissolved in CH_2Cl_2 (0.5 mL) and EtOH (0.2 mL), followed by the addition of a solution of **1** (0.050 mmol) and **2** (0.100 mmol) in CH_2Cl_2 [0.5 mL, total CH_2Cl_2/EtOH (1.2 mL) = 5:1]. The mixture was stirred at room temperature for 16 h and then concentrated. The crude product was purified by silica gel PTLC to furnish **3** and **4**.

General Procedure of Conditions A2 (Figure 3): (*S*)-Difluorphos (3.4 mg, 0.0050 mmol) and $[Rh(cod)_2]BF_4$ (2.0 mg, 0.0050 mmol) were dissolved in CH_2Cl_2 (1.0 mL) in a Schlenk tube, and the mixture was stirred at room temperature for 10 min. After introduction of H_2 and stirring at room temperature for 30 min, the resulting mixture was concentrated to dryness. The residue was dissolved in EtOH (0.1 mL), followed by the addition of a solution of **1** (0.050 mmol) and **2** (0.100 mmol) in CH_2Cl_2 [0.5 mL, total CH_2Cl_2/EtOH (0.6 mL) = 5:1]. The mixture was stirred at room temperature for 16 h and then concentrated. The crude product was purified by silica gel PTLC to furnish **3** and **4**.

General Procedure of Conditions A3 (Figure 3): (*S*)-Difluorphos (3.4 mg, 0.0050 mmol) and $[Rh(cod)_2]BF_4$ (2.0 mg, 0.0050 mmol) were dissolved in CH_2Cl_2 (1.0 mL) in a Schlenk tube, and the mixture was stirred at room temperature for 10 min. After introduction of H_2 and stirring at room temperature for 30 min, the resulting mixture was concentrated to dryness. The residue was dissolved in CH_2Cl_2 (2.3 mL) and EtOH (13.3 mL), followed by the addition of a solution of **1** (0.050 mmol) and **2** (0.100 mmol) in CH_2Cl_2 [1.0 mL, total CH_2Cl_2/EtOH (16.7 mL) = 1:4]. The mixture was stirred at room temperature for 16 h and then concentrated. The crude product was purified by silica gel PTLC to furnish **3** and **4**.

General Procedure of Conditions B1 (Figure 3): (*S*)-Difluorphos (6.8 mg, 0.010 mmol) and $[Rh(cod)_2]BF_4$ (4.1 mg, 0.010 mmol) were dissolved in CH_2Cl_2 (2.0 mL) in a Schlenk tube, and the mixture was stirred at room temperature for 10 min. After introduction of H_2 and stirring at room temperature for 30 min, the resulting mixture was concentrated to dryness. The residue was dissolved in CH_2Cl_2 (0.5 mL), followed by the addition of a solution of **1** (0.10 mmol) and **2** (0.20 mmol) in CH_2Cl_2 (0.5 mL). The mixture was stirred at room temperature for 16 h and then concentrated. The crude product was purified by silica gel PTLC to furnish **3** and **4**.

General Procedure of Conditions B2 (Figure 3): (*S*)-Difluorphos (3.4 mg, 0.0050 mmol) and $[Rh(cod)_2]BF_4$ (2.0 mg, 0.0050 mmol) were dissolved in CH_2Cl_2 (2.0 mL) in a Schlenk tube, and the mixture was stirred at room temperature for 10 min. After introduction of H_2 and stirring at room temperature for 30 min, the resulting mixture was concentrated to dryness. The residue was dissolved in CH_2Cl_2 (0.5 mL), followed by the addition of a solution of **1** (0.050 mmol) and **2** (0.100 mmol) in CH_2Cl_2 (0.5 mL). The mixture was stirred at room temperature for 16 h and then concentrated. The crude product was purified by silica gel PTLC to furnish **3** and **4**.

General Procedure of Conditions B3 (Figure 3): (*S*)-Difluorphos (3.4 mg, 0.0050 mmol) and $[Rh(cod)_2]BF_4$ (2.0 mg, 0.0050 mmol) were dissolved in CH_2Cl_2 (2.0 mL) in a Schlenk tube, and the mixture was stirred at room temperature for 10 min. After introduction of H_2 and stirring at room temperature for 30 min, the resulting mixture was concentrated to dryness. The residue was dissolved in CH_2Cl_2 (0.2 mL), followed by the addition of a solution of **1** (0.050 mmol) and **2** (0.100 mmol) in CH_2Cl_2 (0.3 mL). The mixture was stirred at room temperature for 16 h and then concentrated. The crude product was purified by silica gel PTLC to furnish **3** and **4**.

Note: For chiral HPLC analyses, racemic samples were prepared using *rac*-BINAP. Due to the low diastereoselectivity and regioselectivity under these conditions, the peaks of the resulting products overlapped in the chromatogram of the racemic samples.

(*S,R*)-(–)-Methyl 6-(2-(*tert*-butyl)-6-methylphenoxy)-7-methyl-4-(naphthalen-1-yl)-2-tosylisoindoline-5-carboxylate [(*S,R*)-(–)-3na]

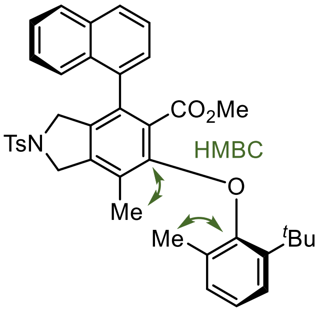

(*S,R*)-(–)-**3na** (43.2 mg, 0.0682 mmol, 68% yield, 89% *ee*, dr ≥ 95:5) was prepared from **1n** (38.8 mg, 0.100 mmol) and **2a** (50.3 mg, 0.204 mmol) according to the general procedure of **conditions B1** and isolated by silica gel PTLC (*n*-hexane/EtOAc = 3:1).

White solid; mp 109.3–111.3 °C; $[\alpha]^{25}_{D}$ –73.6 (*c* 2.26, $CHCl_3$, 89% *ee*); ^{1}H NMR (400 MHz, $CDCl_3$) δ 7 .86 (d, *J* = 6.7 Hz, 1H), 7.84 (d, *J* = 7.2 Hz, 1H), 7.63 (d, *J* = 8.2 Hz, 2H), 7.49–7.45 (m, 2H), 7.37–7.35 (m, 2H), 7.29–7.27 (m, 3H), 7.21–7.18 (m, 1H), 6.98–6.92 (m, 2H), 4.61 (s, 2H), 4.20 (ddd, *J* = 1.7, 1.7, 13.4 Hz, 1H), 4.11 (ddd, *J* = 1.5, 1.5, 13.6 Hz, 1H), 3.04 (s, 3H), 2.41 (s, 3H), 1.98 (s, 3H), 1.83 (s, 3H), 1.38 (s, 9H); ^{13}C NMR (101 MHz, $CDCl_3$) δ 167.0, 153.0, 151.1, 143.7, 140.4, 138.2, 134.0, 133.9, 133.5, 132.2, 130.9, 129.9, 129.8, 129.6, 129.1, 128.7, 128.3, 127.6, 127.1, 126.4, 126.1, 126.0, 125.5, 125.15, 125.06, 123.9, 121.0, 53.9, 53.5, 51.5, 35.3, 30.4, 21.6, 17.9, 13.9; HRMS (FD) calcd for $C_{39}H_{39}NO_5S$ $[M]^+$ 633.2549, found 633.2547; CHIRALPAK IA, *n*-hexane/2-PrOH = 90:10, 1.0 mL/min, retention times: 6.1 min (minor isomer) and 6.9 min (major isomer).

(–)-Methyl 6-(2-(*tert*-butyl)-6-methylphenoxy)-7-methyl-4-(phenanthren-9-yl)-2-tosylisoindoline-5-carboxylate [(–)-3oa]

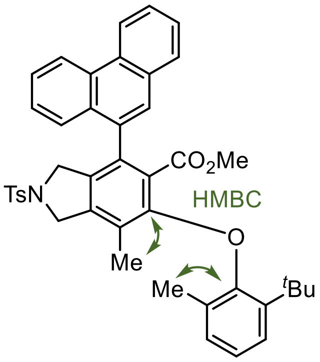

(–)-**3oa** (13.2 mg, 0.0193 mmol, 39% yield, 91% *ee*, dr ≥ 95:5) was prepared from **1o** (21.5 mg, 0.0491 mmol) and **2a** (24.8 mg, 0.101 mmol) according to the general procedure of **conditions B2** and isolated by silica gel PTLC twice (*n*-hexane/EtOAc = 4:1 and then *n*-hexane/ CH_2Cl_2 = 2:3).

Pale yellow solid; mp 137.1–139.7 °C; $[\alpha]^{25}_{D}$ –62.7 (*c* 0.66, $CHCl_3$, 91% *ee*); ^{1}H NMR (400 MHz, $CDCl_3$) δ 8.74 (d, *J* = 8.3 Hz, 1H), 8.71 (d, *J* = 8.2 Hz, 1H), 7.86 (d, *J* = 7.1 Hz, 1H), 7.72–7.60 (m, 5H), 7.56 (s, 1H), 7.48 (ddd, *J* = 0.6, 7.7, 7.2 Hz, 1H), 7.42 (dd, *J* = 1.0, 8.1 Hz, 1H), 7.27 (d, *J* = 8.1 Hz, 2H), 7.21 (dd, *J* = 3.1, 6.4 Hz, 1H), 7.00–6.95 (m, 2H), 4.65 (d, *J* = 14.0 Hz, 1H), 4.60 (d, *J* = 14.2 Hz, 1H), 4.22 (d, *J* = 13.6 Hz, 1H), 4.11 (d, *J* = 13.8 Hz, 1H), 3.05 (s, 3H), 2.42 (s, 3H), 2.03 (s, 3H), 1.84 (s, 3H), 1.39 (s, 9H); ^{13}C NMR (101 MHz, $CDCl_3$) δ 167.0, 153.0, 151.2, 143.6, 140.4, 138.3, 133.9, 132.7, 132.1, 131.1, 130.40, 130.40, 129.90, 129.86, 129.8, 129.7, 129.2, 128.9, 128.0, 127.6, 127.1, 126.87, 126.85, 126.8, 126.3, 126.2, 125.2, 123.9, 122.9, 122.6, 121.0, 53.9, 53.4, 51.6, 35.3, 30.3, 21.6, 17.9, 13.9; HRMS (FD) calcd for $C_{43}H_{41}NO_5S$ $[M]^+$ 683.2705, found 683.2703;

CHIRALPAK IG-3, *n*-hexane/2-PrOH = 92:8, 1.0 mL/min, retention times: 20.5 min (major isomer) and 29.3 min (minor isomer).

(*S,R*)-(–)-Methyl 6-(2-(*tert*-butyl)-6-methylphenoxy)-7-methyl-4-(pyren-4-yl)-2-tosylisoindoline-5-carboxylate [(*S,R*)-(–)-3pa]

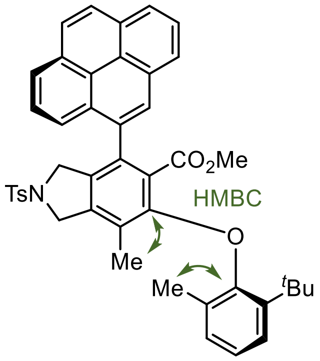

(*S,R*)-(–)-**3pa** (18.6 mg, 0.0263 mmol, 52% yield, 98% *ee*, dr ≥ 95:5) was prepared from **1p** (22.9 mg, 0.0496 mmol) and **2a** (25.0 mg, 0.102 mmol) according to the general procedure of **conditions B2** and isolated by silica gel PTLC twice (*n*-hexane/EtOAc = 4:1 and then *n*-hexane/ CH_2Cl_2 = 2:3).

White solid; mp 147.1–150.1 °C; $[\alpha]^{25}_{D}$ –144.8 (*c* 0.86, $CHCl_3$, 98% *ee*); ^1H NMR (400 MHz, $CDCl_3$) δ 8.22 (d, *J* = 7.6 Hz, 1H), 8.18 (d, *J* = 6.4 Hz, 1H), 8.16 (d, *J* = 5.9 Hz, 1H), 8.13 (d, *J* = 9.0 Hz, 1H), 8.09 (d, *J* = 8.9 Hz, 1H), 8.03 (t, *J* = 7.6 Hz, 1H), 7.96 (d, *J* = 9.2 Hz, 1H), 7.81 (d, *J* = 7.8 Hz, 1H), 7.62–7.59 (m, 3H), 7.26 (d, *J* = 7.9 Hz, 2H), 7.20 (dd, *J* = 3.2, 6.4 Hz, 1H), 6.99–6.95 (m, 2H), 4.66 (s, 2H), 4.17 (ddd, *J* = 1.5, 1.5, 13.6 Hz, 1H), 4.10 (ddd, *J* = 1.5, 1.5, 13.7 Hz, 1H), 2.94 (s, 3H), 2.41 (s, 3H), 2.03 (s, 3H), 1.90 (s, 3H), 1.41 (s, 9H); ^{13}C NMR (101 MHz, $CDCl_3$) δ 167.0, 153.0, 151.2, 143.6, 140.5, 138.3, 133.9, 132.7, 131.29, 131.27, 130.9, 129.93, 129.88, 129.8, 129.2, 128.5, 128.0, 127.9, 127.5, 127.3, 127.2, 126.18, 126.15, 125.5, 125.3, 125.2, 124.69, 124.68, 124.66, 124.4, 123.9, 121.1, 53.9, 53.5, 51.5, 35.3, 30.4, 21.6, 17.9, 14.0; HRMS (FD) calcd for $C_{45}H_{41}NO_5S$ $[M]^+$ 707.2705, found 707.2674; CHIRALPAK IF-3, *n*-hexane/2-PrOH = 80:20, 1.0 mL/min, retention times: 11.4 min (major isomer) and 13.1 min (minor isomer).

(–)-Methyl 6-((3-(*tert*-butyl)-4',5-dimethyl-[1,1'-biphenyl]-2-yl)oxy)-7-methyl-4-(naphthalen-1-yl)-2-tosylisoindoline-5-carboxylate [(–)-3nc]

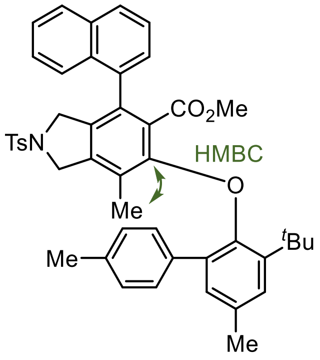

(–)-**3nc** (30.5 mg, 0.0422 mmol, 89% yield, 97% *ee*, dr ≥ 95:5) was prepared from **1n** (18.3 mg, 0.0472 mmol) and **2c** (33.0 mg, 0.0981 mmol) according to the general procedure of **conditions A1** and isolated by silica gel PTLC (cyclohexane/acetone = 3:1).

Pale yellow solid; mp 115.7–116.7 °C; $[\alpha]^{25}_{D}$ –180.7 (*c* 1.30, $CHCl_3$, 97% *ee*); ^1H NMR (400 MHz, $CDCl_3$) δ 7.84 (d, *J* = 8.4 Hz, 1H), 7.83 (d, *J* = 8.4 Hz, 1H), 7.60 (d, *J* = 8.3 Hz, 2H), 7.49 (dd, *J* = 7.6, 7.6 Hz, 1H), 7.43 (ddd, *J* = 1.3, 6.8, 8.1 Hz, 1H), 7.31–7.27 (m, 3H), 7.21 (d, *J* = 8.0 Hz, 1H), 7.15–7.12 (m, 4H), 6.84–6.81 (m, 3H), 4.40 (d, *J* = 13.6 Hz, 1H), 4.21 (d, *J* = 13.6 Hz, 1H), 3.93 (d, *J* = 14.4 Hz, 1H), 3.89 (d, *J* = 14.5 Hz, 1H), 3.07 (s, 3H), 2.44 (s, 3H), 2.31 (s, 3H), 2.10 (s, 3H), 1.71 (s, 3H), 1.44 (s, 9H); ^{13}C NMR (101 MHz, $CDCl_3$) δ 166.8, 151.2, 150.3, 143.6, 139.9, 137.7, 136.0, 135.7, 134.4, 133.6, 133.5, 132.2, 132.0, 131.2, 130.8, 130.5, 129.8, 129.04, 128.98, 128.4,

128.2, 127.6, 127.5, 127.3, 126.6, 126.2, 126.0, 125.9, 125.3, 125.1, 121.2, 53.5, 53.3, 51.4, 35.4, 30.3, 21.5, 21.0, 20.9, 14.1; HRMS (FD) calcd for $C_{46}H_{45}NO_5S$ $[M]^+$ 723.3018, found 723.2985; CHIRALPAK IG-3, *n*-hexane/2-PrOH = 80:20, 1.0 mL/min, retention times: 8.1 min (minor isomer) and 9.6 min (major isomer).

(–)-Methyl 6-((3-(*tert*-butyl)-4',5-dimethyl-[1,1'-biphenyl]-2-yl)oxy)-7-methyl-4-(phenanthren-9-yl)-2-tosylisoindoline-5-carboxylate [(–)-3oc]

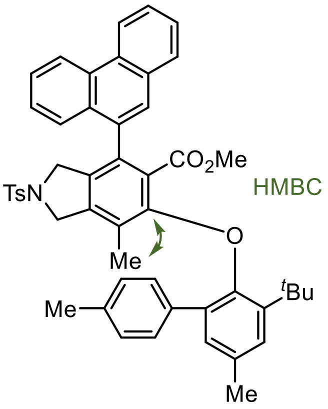

(–)-**3oc** (35.1 mg, 0.0453 mmol, 92% yield, 97% *ee*, dr ≥ 95:5) was prepared from **1o** (21.6 mg, 0.0494 mmol) and **2c** (34.0 mg, 0.101 mmol) according to the general procedure of **conditions A1** and isolated by silica gel PTLC twice (*n*-hexane/EtOAc = 4:1 and then *n*-hexane/ CH_2Cl_2 = 1:1).

Yellow solid; mp 127.7–129.4 °C; $[\alpha]^{25}_D$ –189.6 (*c* 1.75, $CHCl_3$, 97% *ee*); ^1H NMR (400 MHz, $CDCl_3$) δ 8.70 (d, *J* = 8.1 Hz, 2H), 7.91 (dd, *J* = 1.4, 7.7 Hz, 1H), 7.72–7.65 (m, 2H), 7.64–7.58 (m, 3H), 7.41 (ddd, *J* = 1.0, 7.1, 8.1 Hz, 1H), 7.38 (s, 1H), 7.31–7.28 (m, 3H), 7.16–7.14 (m, 3H), 6.86 (d, *J* = 7.8 Hz, 2H), 6.83 (d, *J* = 1.7 Hz, 1H), 4.44 (d, *J* = 13.7 Hz, 1H), 4.23 (d, *J* = 13.7 Hz, 1H), 3.95 (d, *J* = 13.8 Hz, 1H), 3.91 (d, *J* = 13.5 Hz, 1H), 3.09 (s, 3H), 2.43 (s, 3H), 2.32 (s, 3H), 2.16 (s, 3H), 1.72 (s, 3H), 1.46 (s, 9H); ^{13}C NMR (101 MHz, $CDCl_3$) δ 166.8, 151.3, 150.4, 143.6, 139.9, 137.9, 136.0, 135.8, 133.6, 133.2, 132.2, 131.9, 131.14, 131.11, 130.6, 130.33, 130.30, 129.8, 129.1, 128.8, 127.7, 127.61, 127.57, 127.3, 127.1, 126.9, 126.8, 126.7, 126.2, 126.1, 122.9, 122.6, 121.3, 53.6, 53.3, 51.5, 35.4, 30.3, 21.6, 20.99, 20.98, 14.2; HRMS (FD) calcd for $C_{50}H_{47}NO_5S$ $[M]^+$ 773.3175, found 773.3142; CHIRALPAK IE-3, *n*-hexane/2-PrOH = 60:40, 1.0 mL/min, retention times: 7.8 min (minor isomer) and 8.9 min (major isomer).

(–)-Methyl 6-((3-(*tert*-butyl)-4',5-dimethyl-[1,1'-biphenyl]-2-yl)oxy)-7-methyl-4-(pyren-4-yl)-2-tosylisoindoline-5-carboxylate [(–)-3pc]

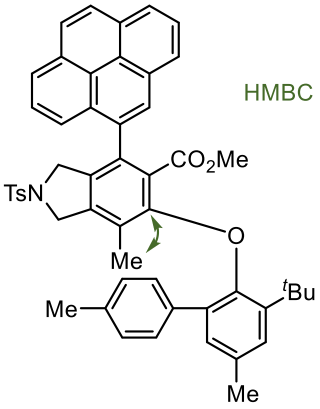

(–)-**3pc** (27.8 mg, 0.0349 mmol, 72% yield, 95% *ee*, dr ≥ 95:5) was prepared from **1p** (22.8 mg, 0.0494 mmol) and **2c** (33.5 mg, 0.0996 mmol) according to the general procedure of **conditions A1** and isolated by silica gel PTLC twice (*n*-hexane/EtOAc = 4:1 *n*-hexane/ CH_2Cl_2 = 1:1).

Yellow solid; mp 161.5–163.5 °C; $[\alpha]^{25}_D$ –209.4 (*c* 1.44, $CHCl_3$, 95% *ee*); ^1H NMR (400 MHz, $CDCl_3$) δ 8.20 (d, *J* = 7.9 Hz, 2H), 8.14 (d, *J* = 7.1 Hz, 1H), 8.10 (d, *J* = 1.8 Hz, 2H), 8.00 (dd, *J* =

7.6, 7.6 Hz, 1H), 7.89 (d, J = 9.2 Hz, 1H), 7.67 (d, J = 7.8 Hz, 1H), 7.56 (d, J = 8.2 Hz, 2H), 7.47 (d, J = 9.2 Hz, 1H), 7.27 (d, J = 7.9 Hz, 2H), 7.18 (d, J = 8.0 Hz, 2H), 7.16 (d, J = 2.3 Hz, 1H), 6.91 (d, J = 7.8 Hz, 2H), 6.84 (dd, J = 0.5, 2.2 Hz, 1H), 4.45 (d, J = 13.7 Hz, 1H), 4.29 (d, J = 13.7 Hz, 1H), 3.91 (d, J = 14.3 Hz, 1H), 3.87 (d, J = 14.2 Hz, 1H), 2.99 (s, 3H), 2.42 (s, 3H), 2.31 (s, 3H), 2.17 (s, 3H), 1.77 (s, 3H), 1.47 (s, 9H); ^{13}C NMR (101 MHz, $CDCl_3$) δ 166.8, 151.3, 150.3, 143.6, 140.1, 137.8, 136.2, 135.7, 133.6, 132.3, 132.1, 131.8, 131.7, 131.3, 131.1, 130.9, 130.5, 129.8, 129.3, 129.1, 128.2, 127.81, 127.78, 127.5, 127.4, 127.3, 126.8, 126.1, 125.4, 125.2, 124.7, 124.6, 124.5, 124.4, 121.3, 53.6, 53.3, 51.4, 35.4, 30.4, 21.6, 21.0, 20.9, 14.2; HRMS (FD) calcd for $C_{52}H_{47}NO_5S$ $[M]^+$ 797.3175, found 797.3200; CHIRALPAK IF-3, *n*-hexane/2-PrOH = 97:3, 1.0 mL/min, retention times: 31.8 min (minor isomer) and 42.7 min (major isomer).

(–)-Methyl 6-(2-(*tert*-butyl)-6-methylphenoxy)-4-(1H-indol-7-yl)-7-methyl-2-tosylisoindoline-5-carboxylate [(–)-3qa]

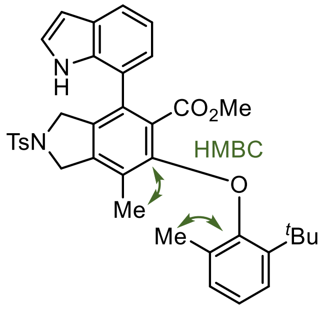

(–)-**3qa** (37.5 mg, 0.0603 mmol, 60% yield, 92% *ee*, dr = 51:49) was prepared from **1q** (38.0 mg, 0.101 mmol) and **2a** (51.3 mg, 0.208 mmol) according to the general procedure of **conditions B1** and isolated by silica gel PTLC (*n*-hexane/EtOAc = 4:1). Two diastereomers of **3qa** were able to separate by silica gel PTLC (*n*-hexane/EtOAc = 4:1), but epimerization rapidly proceeded at room temperature.

Brown solid; mp 99.0–101.0 °C; $[\alpha]^{25}_D$ –91.8 (*c* 1.88, $CHCl_3$, 92% *ee*); ^{1}H NMR (**3qa**, 400 MHz, $CDCl_3$) δ 7.94 (s, 1H), 7.68 (d, J = 8.2 Hz, 2H), 7.63 (d, J = 7.8 Hz, 1H), 7.29 (d, J = 8.0 Hz, 2H), 7.20 (dd, J = 1.8, 7.6 Hz, 1H), 7.14 (dd, J = 2.8, 2.8 Hz, 1H), 7.11 (dd, J = 7.6, 7.6 Hz, 1H), 6.98 (dd, J = 0.8, 7.1 Hz, 1H), 6.97 (dd, J = 7.5, 7.5 Hz, 1H), 6.93 (dd, J = 1.5, 7.4 Hz, 1H), 6.56 (dd, J = 2.0, 3.1 Hz, 1H), 4.62 (dd, J = 1.7, 14.0 Hz, 1H), 4.59 (dd, J = 1.5, 13.9 Hz, 1H), 4.41 (d, J = 13.7 Hz, 1H), 4.16 (d, J = 13.7 Hz, 1H), 3.12 (s, 3H), 2.41 (s, 3H), 1.93 (s, 3H), 1.81 (s, 3H), 1.40 (s, 9H); ^{1}H NMR (**3qa'**, 400 MHz, $CDCl_3$) δ 7.91 (s, 1H), 7.67 (d, J = 8.2 Hz, 1H), 7.62 (d, J = 7.9 Hz, 2H), 7.29 (d, J = 8.0 Hz, 2H), 7.21 (dd, J = 1.7, 7.7 Hz, 1H), 7.18 (dd, J = 2.8, 2.8 Hz, 1H), 7.07 (dd, J = 7.6, 7.6 Hz, 1H), 6.97 (dd, J = 7.5, 7.5 Hz, 1H), 6.92 (dd, J = 1.6, 7.6 Hz, 1H), 6.89 (dd, J = 0.8, 7.2 Hz, 1H), 6.57 (dd, J = 2.0, 3.2 Hz, 1H), 4.70 (dd, J = 2.0, 13.7 Hz, 1H), 4.52 (d, J = 13.8 Hz, 1H), 4.27 (dd, J = 2.0, 13.6 Hz, 1H), 4.21 (ddd, J = 1.8, 1.8, 14.0 Hz, 1H), 3.07 (s, 3H), 2.41 (s, 3H), 1.96 (s, 3H), 1.86 (s, 3H), 1.39 (s, 9H); ^{13}C NMR (101 MHz, $CDCl_3$) δ 167.3, 167.1, 152.9, 152.8, 151.28, 151.25, 143.8, 143.7, 140.8, 140.4, 138.9, 138.8, 133.9, 133.7, 133.6, 133.4, 130.3, 130.1, 129.86, 129.86, 129.7, 129.6, 129.4, 129.1, 129.0, 128.3, 128.2, 127.6, 125.9, 125.6, 125.3, 125.2, 124.7, 124.6, 124.1, 124.0, 122.5, 122.1, 121.2, 121.1, 121.0, 120.9, 119.8, 119.7, 103.0, 102.9, 53.83, 53.82, 53.43, 53.39, 51.8, 51.7, 35.30, 35.30, 30.40, 30.37, 21.55, 21.55, 17.87, 17.85, 13.87, 13.87; HRMS (FD) calcd for $C_{37}H_{38}N_2O_5S$ $[M]^+$ 622.2501, found 622.2526; CHIRALPAK IE-3, *n*-hexane/2-PrOH = 85:15, 1.0 mL/min, retention times: 12.8 min (major isomer of **3qa/3qa'**), 15.2 min (minor isomer of **3qa/3qa'**), 17.2 min (minor isomer of **3qa'/3qa**) and 20.6 min (major isomer of **3qa'/3qa**).

(–)-Methyl 7-([1,1'-biphenyl]-2-yl)-6-(2-(*tert*-butyl)-6-methylphenoxy)-4-methyl-2-tosylisoindoline-5-carboxylate [(–)-3ra]

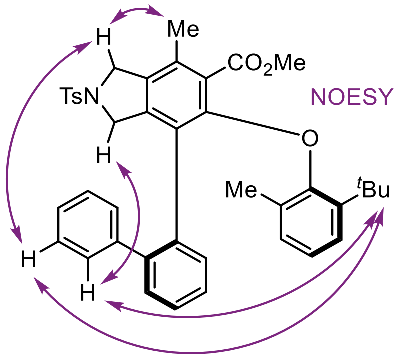

V

(–)-**3ra** (6.1 mg, 0.0093 mmol, 19% yield, 99% *ee*, dr ≥ 95:5) was prepared from **1r** (20.1 mg, 0.0486 mmol) and **2a** (24.7 mg, 0.100 mmol) according to the general procedure of **conditions B3** and isolated by silica gel PTLC (*n*-hexane/EtOAc = 2:1).

Brown oil; $[\alpha]^{25}_{D}$ –67.5 (*c* 0.31, $CHCl_3$, 99% *ee*); ^{1}H NMR (400 MHz, $CDCl_3$) δ 7.75 (d, *J* = 8.2 Hz, 2H), 7.42–7.38 (m, 3H), 7.35 (d, *J* = 8.0 Hz, 2H), 7.17–7.07 (m, 6H), 7.03 (dd, *J* = 1.3, 7.8 Hz, 1H), 6.86 (dd, *J* = 7.6, 7.6 Hz, 1H), 6.74 (dd, *J* = 1.0, 7.5 Hz, 1H), 4.64–4.55 (m, 3H), 4.48 (dd, *J* = 2.0, 14.0 Hz, 1H), 2.96 (s, 3H), 2.46 (s, 3H), 1.98 (s, 3H), 1.54 (s, 3H), 0.98 (s, 9H); ^{13}C NMR (101 MHz, $CDCl_3$) δ 166.7, 150.8, 150.4, 143.8, 142.2, 141.5, 141.4, 138.6, 133.7, 133.5, 131.00, 130.99, 130.7, 130.4, 130.0, 129.4, 128.7, 128.5, 128.4, 128.0, 127.6, 127.4, 127.0, 125.2, 124.41, 124.39, 120.3, 54.5, 53.6, 51.5, 34.9, 29.9, 21.6, 16.5, 15.8; HRMS (FD) calcd for $C_{41}H_{41}NO_5S$ $[M]^+$ 659.2705, found 659.2730; CHIRALPAK IF-3, *n*-hexane/^{i}PrOH = 80:20, 1.0 mL/min, retention times: 10.9 min (minor isomer) and 12.0 min (major isomer).

(–)-Methyl 7-(2-aminophenyl)-6-(2-(*tert*-butyl)-6-methylphenoxy)-4-methyl-2-tosylisoindoline-5-carboxylate [(–)-3sa]

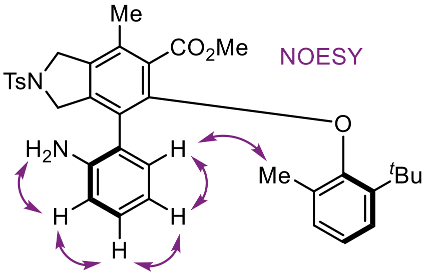

(–)-**3sa** (31.8 mg, 0.0481 mmol, 50% yield, 95% *ee*, dr ≥ 95:5) was prepared from **1s** (35.1 mg, 0.0996 mmol) and **2a** (49.7 mg, 0.202 mmol) according to the general procedure of **conditions B1** and isolated by silica gel PTLC (*n*-hexane/EtOAc = 16:9).

Preparative scale synthesis: (*S*)-Difluorphos (34.1 mg, 0.0500 mmol) and $[Rh(cod)_2]BF_4$ (20.3 mg, 0.0500 mmol) were dissolved in CH_2Cl_2 (2.0 mL) in a Schlenk tube, and the mixture was stirred at room temperature for 10 min. After introduction of H_2 and stirring at room temperature for 30 min, the resulting mixture was concentrated to dryness. The residue was dissolved in CH_2Cl_2 (8.0 mL) followed by the addition of a solution of **1s** (353.2 mg, 1.002 mmol) and **2a** (493.4 mg, 2.003 mmol) in CH_2Cl_2 (2.0 mL). The mixture was stirred at room temperature for 72 h and then concentrated. The crude product was purified by silica gel column chromatography (*n*-hexane/EtOAc = 1:1) followed by silica gel PTLC (*n*-hexane/CH_2Cl_2/Et_2O = 2:1:1) to furnish (–)-**3sa** (314.8 mg, 0.4770 mmol, 52% yield, 94% *ee*, dr ≥ 95:5).

Pale brown solid; mp131.8–133.4 °C; $[\alpha]^{25}_{D}$ –43.5 (*c* 0.11, $CHCl_3$, 95% *ee*); ^{1}H NMR (400 MHz, $CDCl_3$) δ 7.73 (d, *J* = 8.2 Hz, 2H), 7.31 (d, *J* = 8.0 Hz, 2H), 7.12–7.07 (m, 2H), 6.93–6.89 (m, 2H), 6.87 (dd, *J* = 1.4, 7.6 Hz, 1H), 6.72 (dd, *J* = 0.6, 8.1 Hz, 1H), 6.67 (ddd, *J* = 0.9, 7.4, 7.4 Hz, 1H), 4.58 (s, 2H), 4.47 (d, *J* = 14.4 Hz, 1H), 4.35 (d, *J* = 14.4 Hz, 1H), 3.47 (s, 2H), 3.18 (s, 3H), 2.42 (s, 3H), 2.05 (s, 3H), 1.99 (s, 3H), 1.16 (s, 9H); ^{13}C NMR (101 MHz, $CDCl_3$) δ 166.9, 151.9, 151.2,

143.9, 143.7, 141.5, 139.4, 134.0, 130.9, 129.9, 129.8, 129.7, 129.6, 129.3, 129.1, 127.6, 124.9, 124.2, 122.7, 122.0, 120.4, 118.4, 115.5, 53.9, 53.4, 51.7, 35.1, 30.3, 21.5, 18.1, 16.4; HRMS (ESI) calcd for $C_{35}H_{38}N_2O_5S$ $[M+Na]^+$ 621.2399, found 621.2392; CHIRALPAK IF-3, *n*-hexane/2-PrOH = 92:8, 1.0 mL/min, retention times: 35.3 min (minor isomer) and 41.4 min (major isomer).

(–)-Methyl 6-(2-((3*r*,5*r*,7*r*)-adamantan-1-yl)-4,6-dimethylphenoxy)-7-(2-aminophenyl)-4-methyl-2-tosylisoindoline-5-carboxylate [(–)-3si]

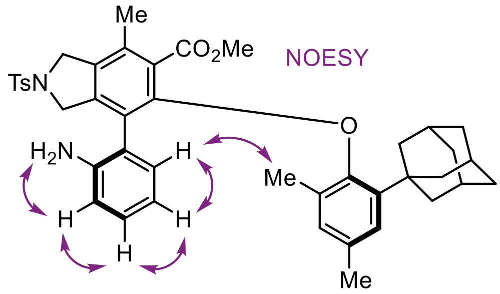

(–)-**3si** (17.3 mg, 0.0251 mmol, 45% yield, 85% *ee*, dr = 89:11) was prepared from **1s** (17.5 mg, 0.0497 mmol) and **2i** (33.9 mg, 0.100 mmol) according to the general procedure of **conditions B2** and isolated by silica gel PTLC (*n*-hexane/EtOAc = 16:9) twice.

Pale brown solid; mp 150.1–152.1 °C; $[\alpha]^{25}_{D}$ –21.6 (*c* 0.87, $CHCl_3$, 85% *ee*); ^{1}H NMR (400 MHz, $CDCl_3$) δ 7.74 (d, *J* = 8.2 Hz, 2H), 7.31 (d, *J* = 8.0 Hz, 2H), 7.08 (ddd, *J* = 1.1, 7.7, 7.7 Hz, 1H), 6.88 (dd, *J* = 1.4, 7.6 Hz, 1H), 6.81 (d, *J* = 1.8 Hz, 1H), 6.70–6.68 (m, 2H), 6.63 (ddd, *J* = 1.0, 7.4, 7.4 Hz, 1H), 4.60 (dd, *J* = 1.6, 14.3 Hz, 1H), 4.58 (s, 2H), 4.32 (dd, *J* = 2.2, 14.5 Hz, 1H), 3.45 (s, 2H), 3.20 (s, 3H), 2.42 (s, 3H), 2.21 (s, 3H), 2.06 (s, 3H), 1.96 (s, 3H), 1.87–1.79 (m, 9H), 1.63 (s, 6H); ^{13}C NMR (101 MHz, $CDCl_3$) δ 167.0, 151.7, 150.1, 143.7, 143.6, 141.6, 139.4, 133.9, 133.1, 130.8, 129.9, 129.8, 129.0, 128.94, 128.91, 127.64,127.63, 125.5, 122.9, 121.6, 120.0, 118.2, 115.3, 54.0, 53.4, 51.6, 41.1, 37.3, 36.9, 29.1, 21.5, 20.9, 18.3, 16.5; HRMS (FD) calcd for $C_{42}H_{46}N_2O_5S$ $[M]^+$ 690.3127, found 690.3119; CHIRALPAK ID-3+IE-3, *n*-hexane/2-PrOH = 95:5, 1.0 mL/min, retention times: 138.1 min (minor isomer) and 168.1 min (major isomer).

Methyl 6-(2-(*tert*-butyl)-6-methylphenoxy)-7-(2-hydroxyphenyl)-4-methyl-2-tosylisoindoline-5-carboxylate and (3ta) and methyl 6-(2-(*tert*-butyl)-6-methylphenoxy)-4-(2-hydroxyphenyl)-7-methyl-2-tosylisoindoline-5-carboxylate (4ta)

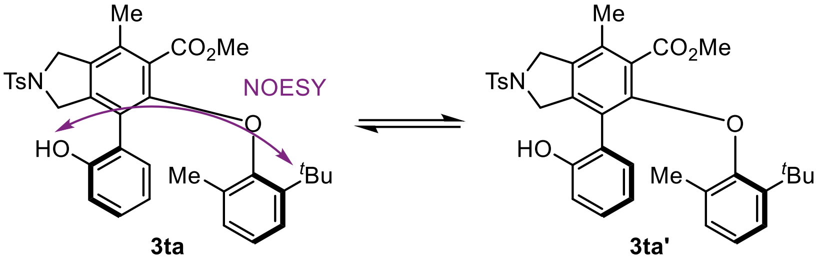

3ta **3ta'**

3ta and **4ta** (43.0 mg, 0.0717 mmol, 71% yield, 84% *ee*, **3ta**/**4ta** = 92:8) were prepared from **1t** (35.3 mg, 0.0999 mmol) and **2a** (49.3 mg, 0.200 mmol) according to the general procedure of **conditions B1** and isolated by silica gel PTLC (*n*-hexane/CH_2Cl_2/Et_2O = 2:2:1). Two diastereomers of **3ta** were able to separate by silica gel PTLC (*n*-hexane/CH_2Cl_2/Et_2O = 2:2:1), but epimerization rapidly proceeded at room temperature.

White solid; mp 113.7–116.7 °C; $[\alpha]^{25}_{D}$ –1.0 (*c* 0.11, $CHCl_3$, 84% *ee*); ^{1}H NMR (**3ta**, 400 MHz, $CDCl_3$) δ 7.72 (d, *J* = 8.2 Hz, 2H), 7.32 (d, *J* = 8.0 Hz, 2H), 7.22 (ddd, *J* = 1.7, 7.4, 8.1 Hz, 1H), 7.08 (dd, *J* = 2.6, 7.0 Hz, 1H), 6.98 (dd, *J* = 1.6, 7.6 Hz, 1H), 6.93–6.85 (m, 4H), 4.68 (s, 1H), 4.64 (dd, *J* = 1.6, 12.9 Hz, 1H), 4.54 (d, *J* = 13.2 Hz, 1H), 4.41 (dd, *J* = 1.5, 14.3 Hz, 1H), 4.34 (dd, *J* = 1.1, 14.4 Hz, 1H), 3.19 (s, 3H), 2.42 (s, 3H), 2.06 (s, 3H), 1.97 (s, 3H), 1.16 (s, 9H); ^{1}H NMR (**3ta'**, 400 MHz, $CDCl_3$) δ 7.72 (d, *J* = 8.2 Hz, 2H), 7.31 (d, *J* = 8.0 Hz, 2H), 7.22 (ddd, *J* = 1.7, 7.8, 7.8 Hz, 1H), 7.12 (dd, *J* = 1.6, 7.6 Hz, 1H), 7.10 (dd, *J* = 1.7, 7.4 Hz, 1H), 6.97–6.86 (m, 4H), 4.79 (s, 1H),

4.61 (d, J = 12.4 Hz, 1H), 4.56 (d, J = 13.4 Hz, 1H), 4.45 (dd, J = 1.0, 14.1 Hz, 1H), 4.36 (dd, J = 0.7, 14.2 Hz, 1H), 3.14 (s, 3H), 2.42 (s, 3H), 2.03 (s, 3H), 2.01 (s, 3H), 1.17 (s, 9H); ^{13}C NMR (101 MHz, $CDCl_3$) δ 166.8, 152.7, 152.4, 151.7, 151.5, 151.2, 151.1, 143.80, 143.76, 142.1, 141.7, 139.5, 139.3, 133.9, 133.8, 131.7, 131.34, 131.30, 130.9, 130.0, 129.90, 129.90, 129.88, 129.8, 129.71, 129.68, 129.5, 129.4, 128.8, 127.6, 124.94, 124.93, 124.7, 124.4, 122.8, 121.9, 121.7, 121.6, 120.9, 120.8, 120.3, 119.8, 115.8, 115.7, 60.4, 53.9, 53.8, 53.4, 51.73, 51.69, 35.2, 35.1, 30.4, 30.3, 21.5, 18.0, 17.7, 16.50, 16.48, 14.2; HRMS (ESI) calcd for $C_{35}H_{37}NO_6S$ [M+Na]$^+$ 622.2239, found 622.2225; CHIRALPAK IG-3, *n*-hexane/2-PrOH = 97:3, 1.0 mL/min, retention times: 76.2 min (minor isomer of **3ta**/**3ta'**), 83.6 min (minor isomer of **3ta**/**3ta'**), 94.2 min (major isomer of **3ta**/**3ta'**) and 98.0 min (major isomer of **3ta**/**3ta'**).

(*R,R*)-(–)-Methyl 5-(2-(*tert*-butyl)-6-methoxy-4-methylphenoxy)-6-(dimethylcarbamoyl)-7-methyl-2-tosylisoindoline-4-carboxylate [(*R,R*)-(–)-3am]

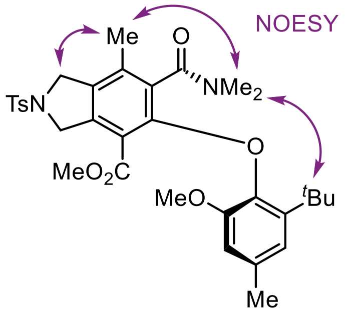

(*R,R*)-(–)-**3am** (18.5 mg, 0.0303 mmol, 62% yield, 92% *ee*, dr ≥ 95:5) was prepared from **1a** (15.5 mg, 0.0485 mmol) and **2m** (29.0 mg, 0.100 mmol) according to the general procedure of **conditions A3** and isolated by silica gel PTLC (*n*-hexane/EtOAc/Et_2O = 1:2:1). During the crystallization study, (*R,S*)-(−)-**3am** gradually underwent epimerization, with the amide moiety rotating to (*R,S*)-(−)-**3am'**, and (*R,S*)-(–)-**3am'** (1.3 mg, 0.0022 mmol, 87% *ee*) was isolated by silica gel PTLC (*n*-hexane/CH_2Cl_2/ EtOAc = 1:4:1).

Pale yellow oil; $[\alpha]^{25}_D$ –33.4 (c 0.05, $CHCl_3$, 92% *ee*); ^{1}H NMR (400 MHz, $CDCl_3$) δ 7.76 (d, J = 8.2 Hz, 2H), 7.32 (d, J = 8.0 Hz, 2H), 6.71 (d, J = 1.4 Hz, 1H), 6.50 (d, J = 1.6 Hz, 1H), 4.75 (ddd, J = 1.9, 1.9, 15.4 Hz, 1H), 4.57 (ddd, J = 1.6, 1.6, 13.4 Hz, 1H), 4.51–4.44 (m, 2H), 3.41 (s, 3H), 3.13 (s, 3H), 3.07 (s, 3H), 2.94 (s, 3H), 2.42 (s, 3H), 2.25 (s, 3H), 2.12 (s, 3H), 1.35 (s, 9H); ^{13}C NMR (101 MHz, $CDCl_3$) δ 167.5, 165.6, 151.3, 149.3, 143.7, 141.0, 139.7, 136.2, 133.8, 132.8, 132.0, 129.9, 129.2, 128.2, 127.6, 119.8, 114.9, 112.6, 56.5, 54.0, 52.8, 51.5, 38.0, 35.1, 34.3, 29.9, 21.52, 21.46, 16.1; HRMS (FD) calcd for $C_{33}H_{40}N_2O_7S$ [M]$^+$ 608.2556, found 608.2523; CHIRALPAK IF-3, *n*-hexane/2-PrOH = 80:20, 1.0 mL/min, retention times: 19.3 min (minor isomer) and 23.7 min (major isomer).

(*R,S*)-(–)- (–)-Methyl 5-(2-(*tert*-butyl)-6-methoxy-4-methylphenoxy)-6-(dimethylcarbamoyl)-7-methyl-2-tosylisoindoline-4-carboxylate [(*R,S*)-(–)-3am']

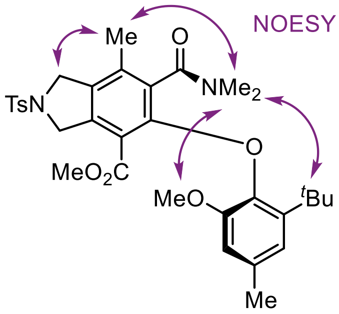

Pale yellow oil; $[\alpha]^{25}_D$ –57.3 (c 0.85, $CHCl_3$, 87% *ee*); ^{1}H NMR (400 MHz, $CDCl_3$) δ 7.74 (d, J = 7.7 Hz, 2H), 7.32 (d, J = 7.9 Hz, 2H), 6.77 (s, 1H), 6.53 (s, 1H), 4.86 (d, J = 13.6 Hz, 1H), 4.62 (d, J = 12.4 Hz, 1H), 4.44 (d, J = 12.6 Hz, 1H), 4.30 (d, J = 14.6 Hz, 1H), 3.49 (s, 3H), 3.06 (s, 3H),

3.02 (s, 3H), 2.83 (s, 3H), 2.42 (s, 3H), 2.29 (s, 3H), 2.13 (s, 3H), 1.33 (s, 9H); ^{13}C NMR (101 MHz, $CDCl_3$) δ 167.6, 165.7, 150.7, 148.8, 143.8, 141.7, 139.8, 135.9, 133.9, 133.2, 132.5, 129.9, 128.8, 128.4, 127.5, 119.8, 114.3, 110.6, 55.0, 53.9, 52.9, 51.5, 37.5, 35.2, 34.4, 30.1, 21.6, 21.5, 16.2; HRMS (FD) calcd for $C_{33}H_{40}N_2O_7S$ $[M]^+$ 608.2556, found 608.2563; CHIRALPAK IF-3, *n*-hexane/2-PrOH = 80:20, 1.0 mL/min, retention times: 23.6 min (major isomer) and 43.2 min (minor isomer).

(+)-7-(2-Aminophenyl)-6-(2-(*tert*-butyl)-6-methylphenoxy)-*N*,*N*,4-trimethyl-2-tosylisoindoline-5-carboxamide [(+)-3sn]

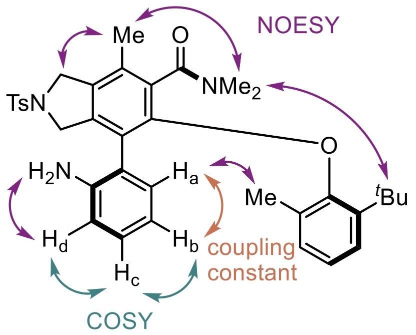

(+)-**3sn** (6.0 mg, 0.0099 mmol, 20% yield, 89% *ee*, dr = 95:3:2) was prepared from **1s** (17.3 mg, 0.0491 mmol) and **2n** (26.1 mg, 0.101 mmol) according to the general procedure of **conditions A2** and isolated by silica gel PTLC twice (*n*-hexane/EtOAc = 2:1 and then *n*-hexane/CH_2Cl_2/ EtOAc = 2:2:1).

Colorless oil; $[\alpha]^{25}_D$ +14.0 (*c* 0.30, $CHCl_3$, 89% *ee*); ^{1}H NMR (400 MHz, $CDCl_3$) δ 7.70 (d, *J* = 8.3 Hz, 2H), 7.31 (d, *J* = 7.9 Hz, 2H), 6.87 (ddd, *J* = 2.0, 6.8, 8.0 Hz, 1H), 6.81–6.79 (m, 2H), 6.75 (dd, *J* = 5.8, 8.9 Hz, 1H), 6.48 (dd, *J* = 0.6, 8.0 Hz, 1H), 6.20 (dd, *J* = 1.9, 7.7 Hz, 1H), 6.16 (ddd, *J* = 1.0, 6.8, 7.7 Hz, 1H), 4.67 (dd, *J* = 2.1, 13.1 Hz, 1H), 4.52 (dd, *J* = 1.3, 13.1 Hz, 1H), 4.32 (dd, *J* = 2.0, 14.3 Hz, 1H), 4.10 (dd, *J* = 1.4, 14.3 Hz, 1H), 3.15 (s, 2H), 3.06 (s, 3H), 2.93 (s, 3H), 2.42 (s, 3H), 2.16 (s, 3H), 2.02 (s, 3H), 1.09 (s, 9H); ^{13}C NMR (101 MHz, $CDCl_3$) δ 168.2, 155.2, 151.1, 143.6, 143.4, 139.0, 138.8, 134.1, 130.9, 130.6, 130.4, 130.0, 129.8, 129.4, 128.7, 128.2, 127.58, 127.55, 124.5, 123.1, 121.6, 118.8, 117.6, 114.9, 54.0, 37.9, 34.9, 34.3, 30.2, 21.6, 19.0, 15.9; HRMS (FD) calcd for $C_{36}H_{41}N_3O_4S$ $[M+H]^+$ 612.2891, found 612.2864; CHIRALPAK IF-3, *n*-hexane/2-PrOH = 60:40, 1.0 mL/min, retention times: 7.8 min (minor isomer) and 8.8 min (major isomer).

(+)-6-((3-(*tert*-Butyl)-4',5-dimethyl-[1,1'-biphenyl]-2-yl)oxy)-*N*,*N*,4-trimethyl-7-(naphthalen-1-yl)-2-tosylisoindoline-5-carboxamide [(+)-3no]

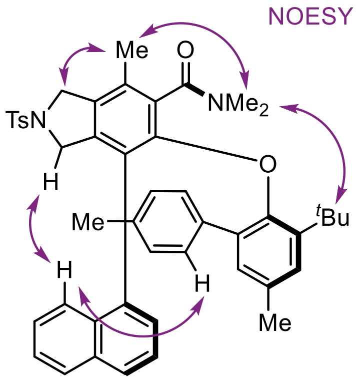

(–)-**3no** (4.4 mg, 0.0059 mmol, 12% yield, 84% *ee*, dr ≥ 95:5) and (+)-**4no** (6.3 mg, 0.0086 mmol, 14% yield, 30% *ee*, dr ≥ 95:5) were prepared from **1n** (19.4 mg, 0.0501 mmol) and **2o** (35.3 mg, 0.101 mmol) according to the general procedure of **conditions A1** and isolated by silica gel PTLC (*n*-hexane/EtOAc = 2:1).

White solid; mp 132.7–136.1 °C; $[\alpha]^{25}_D$ +90.3 (*c* 0.22, $CHCl_3$, 84% *ee*); ^{1}H NMR (400 MHz, $CDCl_3$) δ 7.79 (d, *J* = 8.1 Hz, 1H), 7.58 (d, *J* = 8.2 Hz, 1H), 7.45–7.41 (m, 3H), 7.20 (d, *J* = 8.0 Hz,

2H), 6.97–6.92 (m, 2H), 6.80–6.76 (m, 4H), 6.72 (d, J = 8.3 Hz, 2H), 6.49 (d, J = 8.2 Hz, 1H), 6.09 (d, J = 1.7 Hz, 1H), 4.52 (dd, J = 1.7, 13.0 Hz, 1H), 4.35 (d, J = 13.1 Hz, 1H), 3.78 (d, J = 14.6 Hz, 1H), 3.71 (dd, J = 2.0, 14.5 Hz, 1H), 3.10 (s, 3H), 2.97 (s, 3H), 2.40 (s, 3H), 2.20 (s, 3H), 2.07 (s, 3H), 2.01 (s, 3H), 1.41 (s, 9H); ^{13}C NMR (101 MHz, $CDCl_3$) δ 168.0, 151.5, 149.4, 143.4, 140.1, 138.9, 135.8, 134.0, 133.6, 133.4, 133.3, 133.2, 131.7, 130.5, 129.71, 129.66, 129.6, 129.0, 128.3, 128.0, 127.6, 127.52, 127.47, 127.4, 127.3, 127.0, 126.6, 126.1, 125.6, 125.3, 124.5, 54.4, 53.2, 38.5, 35.4, 34.4, 31.0, 21.5, 21.1, 20.8, 16.0; HRMS (FD) calcd for $C_{47}H_{48}N_2O_4S$ $[M]^+$ 736.3335, found 736.3310; CHIRALPAK ID-3, *n*-hexane/2-PrOH = 70:30, 1.0 mL/min, retention times: 18.3 min (minor isomer) and 24.3 min (major isomer).

(+)-6-((3-(*tert*-Butyl)-4',5-dimethyl-[1,1'-biphenyl]-2-yl)oxy)-*N*,*N*,7-trimethyl-4-(naphthalen-1-yl)-2-tosylisoindoline-5-carboxamide [(+)-4no]

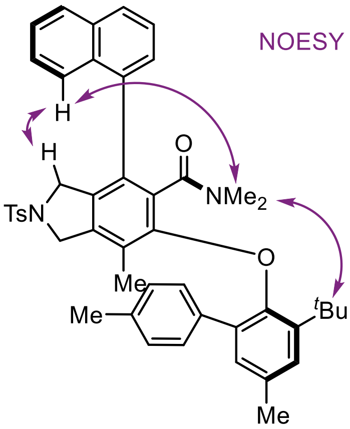

White solid; mp 136.1–143.1 °C; $[\alpha]^{25}_{D}$ +59.8 (*c* 0.32, $CHCl_3$, 30% *ee*); ^{1}H NMR (400 MHz, $CDCl_3$) δ 7.89 (d, J = 8.1 Hz, 1H), 7.85 (d, J = 8.0 Hz, 1H), 7.60 (d, J = 8.2 Hz, 2H), 7.55 (dd, J = 7.6, 7.6 Hz, 1H), 7.50 (dd, J = 1.4, 7.0 Hz, 1H), 7.45 (ddd, J = 1.3, 6.7, 8.1 Hz, 1H), 7.33 (d, J = 7.8 Hz, 4H), 7.29 (ddd, J = 1.3, 6.9, 8.3 Hz, 1H), 7.23 (d, J = 8.3 Hz, 1H), 7.15 (d, J = 2.1 Hz, 1H), 6.86 (d, J = 1.7 Hz, 1H), 6.79 (d, J = 7.8 Hz, 2H), 4.41 (dd, J = 2.2, 13.1 Hz, 1H), 4.05 (dd, J = 2.3, 6.5 Hz, 1H), 4.02 (dd, J = 1.7, 7.2 Hz, 1H), 3.67 (dd, J = 2.4, 12.4 Hz, 1H), 2.53 (s, 3H), 2.47 (s, 3H), 2.46 (s, 3H), 2.33 (s, 3H), 1.92 (s, 3H), 1.56 (s, 3H), 1.43 (s, 9H); ^{13}C NMR (101 MHz, $CDCl_3$) δ 166.8, 150.2, 150.1, 143.6, 138.7, 136.5, 135.8, 135.2, 133.6, 133.5, 133.3, 131.8, 131.4, 130.9, 130.2, 130.0, 129.8, 129.6, 129.3, 128.9, 128.4, 128.2, 128.1, 127.6, 127.4, 127.2, 126.2, 125.62, 125.60, 124.6, 120.2, 53.4, 53.3, 38.1, 35.3, 34.0, 30.0, 21.6, 21.0, 20.6, 14.4; HRMS (FD) calcd for $C_{47}H_{48}N_2O_4S$ $[M]^+$ 736.3335, found 736.3294; CHIRALPAK IG-3, *n*-hexane/2-PrOH = 70:30, 1.0 mL/min, retention times: 12.3 min (major isomer) and 17.5 min (minor isomer).

(+)-7-(2-Aminophenyl)-6-(2-(*tert*-butyl)-6-methylphenoxy)-*N*,*N*-dimethyl-4-(naphthalen-1-yl)-2-tosylisoindoline-5-carboxamide [(+)-3un]

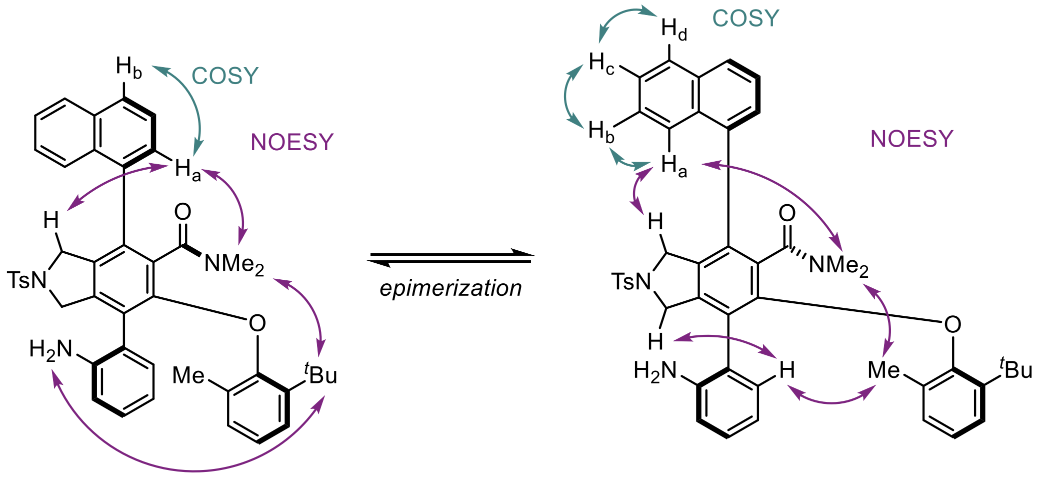

3un **3un'**

(+)-**3un** (6.9 mg, 0.0096 mmol, 19% yield, 97% *ee*, dr = 74:26) and (+)-**3un''** (4.0 mg, 0.0055 mmol, 11% yield, 97% *ee*, dr = 88:12) were prepared from **1u** (23.2 mg, 0.0499 mmol) and **2n** (26.2 mg, 0.101 mmol) according to the general procedure of **conditions A1** and isolated by silica gel PTLC (*n*-hexane/EtOAc = 2:1). (+)-**3un** was further purified by silica gel PTLC (*n*-hexane/CH_2Cl_2/EtOAc = 5:10:2), and (+)-**3un''** was further purified by silica gel PTLC (*n*-hexane/EtOAc = 1:1). Two diastereomers of **3un** were able to separate by silica gel PTLC (*n*-hexane/CH_2Cl_2/Et_2O = 2:2:1), but epimerization rapidly proceeded at room temperature.

Pale brown solid; mp 81.0–83.0 °C; $[\alpha]^{25}_D$ +17.2 (*c* 0.35, $CHCl_3$, 97% *ee*); ^1H NMR (**3un**, 400 MHz, $CDCl_3$) δ 7.89 (d, *J* = 8.2 Hz, 1H), 7.88 (d, *J* = 8.1 Hz, 1H), 7.55–7.49 (m, 3H), 7.48–7.43 (m, 2H), 7.41 (ddd, *J* = 0.7, 0.7, 8.3 Hz, 1H), 7.26 (d, *J* = 7.9 Hz, 2H), 7.18 (dd, *J* = 1.0, 7.0 Hz, 1H), 6.93 (ddd, *J* = 2.2, 6.4, 8.1 Hz, 1H), 6.86 (dd, *J* = 1.8, 7.1 Hz, 1H), 6.84 (dd, *J* = 2.0, 8.0 Hz, 1H), 6.79 (dd, *J* = 7.1, 7.8 Hz, 1H), 6.55 (d, *J* = 7.8 Hz, 1H), 6.27–6.21 (m, 2H), 4.36 (ddd, *J* = 0.9, 1.9, 14.6 Hz, 1H), 4.21 (dd, *J* = 2.7, 18.1 Hz, 1H), 4.21 (dd, *J* = 3.3, 13.8 Hz, 1H), 4.08 (ddd, *J* = 1.1, 2.8, 14.4 Hz, 1H), 3.27 (s, 2H), 3.05 (s, 3H), 2.66 (s, 3H), 2.43 (s, 3H), 2.16 (s, 3H), 1.11 (s, 9H); ^1H NMR (**3un'**, 400 MHz, $CDCl_3$) δ 7.91 (d, *J* = 7.8 Hz, 1H), 7.82 (dd, *J* = 1.8, 7.4 Hz, 1H), 7.58–7.54 (m, 3H), 7.51 (ddd, *J* = 1.5, 6.9, 8.1 Hz, 1H), 7.46 (ddd, *J* = 1.4, 6.8, 8.1 Hz, 1H), 7.41 (dd, *J* = 7.2, 7.2 Hz, 1H), 7.38 (dd, *J* = 1.9, 7.1 Hz, 1H), 7.25 (d, *J* = 7.9 Hz, 2H), 7.16 (ddd, *J* = 1.3, 7.6, 7.6 Hz, 1H), 7.08 (dd, *J* = 4.7, 4.7 Hz, 1H), 7.07 (dd, *J* = 1.6, 7.7 Hz, 1H), 6.78 (dd, *J* = 2.8, 3.9 Hz, 1H), 6.77–6.75 (m, 3H), 4.59 (dd, *J* = 2.1, 14.3 Hz, 1H), 4.38 (dd, *J* = 2.1, 13.7 Hz, 1H), 4.31 (dd, *J* = 1.4, 14.3 Hz, 1H), 3.90 (dd, *J* = 1.5, 13.7 Hz, 1H), 3.67 (s, 2H), 2.41 (s, 3H), 2.00 (s, 3H), 1.88 (s, 3H), 1.80 (s, 3H), 1.34 (s, 9H); ^{13}C NMR (101 MHz, $CDCl_3$) δ 166.6, 164.5, 155.6, 153.1, 152.7, 151.4, 144.4, 143.6, 143.5, 143.4, 142.1, 139.2, 139.0, 138.7, 134.3, 134.1, 134.0, 133.8, 133.5, 133.3, 133.2, 132.1, 131.4, 131.1, 130.8, 130.4, 129.9, 129.8, 129.7, 129.5, 129.23, 129.19, 129.16, 129.1, 128.94, 128.91, 128.8, 128.2, 128.0, 127.9, 127.58, 127.56, 127.5, 126.8, 126.7, 126.6, 126.5, 126.4, 125.93, 125.85, 125.31, 125.28, 124.7, 124.6, 124.3, 123.9, 123.8, 123.1, 120.8, 118.8, 118.3, 117.8, 115.6, 115.1, 54.0, 53.91, 53.88, 53.6, 38.5, 38.3, 35.9, 35.0, 33.9, 33.6, 31.7, 30.2, 21.6, 21.5, 19.2, 19.0; HRMS (FD) calcd for $C_{45}H_{45}N_3O_4S$ $[M+H]^+$ 724.3204, found 724.3182; CHIRALPAK IG-3, *n*-hexane/2-PrOH = 90:10, 1.0 mL/min, retention times: 31.4 min (minor isomer) and 37.4 min (major isomer).

(+)-7-(2-Aminophenyl)-6-(2-(*tert*-butyl)-6-methylphenoxy)-*N*,*N*-dimethyl-4-(naphthalen-1-yl)-2-tosylisoindoline-5-carboxamide [(+)-3un'']

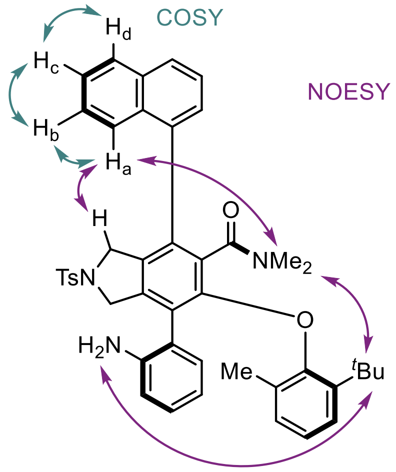

Brown solid; mp 152.5–155.1 °C; $[\alpha]^{25}_{D}$ +143.5 (*c* 0.20, $CHCl_3$, 97% *ee*); ^{1}H NMR (400 MHz, $CDCl_3$) δ 7.95 (d, *J* = 8.0 Hz, 1H), 7.89 (d, *J* = 7.9 Hz, 1H), 7.59 (dd, *J* = 1.4, 7.1 Hz, 1H), 7.56–7.50 (m, 4H), 7.40 (dd, *J* = 1.0, 6.6 Hz, 1H), 7.35 (d, *J* = 8.2 Hz, 1H), 7.25 (d, *J* = 7.8 Hz, 2H), 6.93 (ddd, *J* = 1.3, 7.7, 7.6 Hz, 1H), 6.87 (dd, *J* = 1.8, 7.1 Hz, 1H), 6.85–6.79 (m, 2H), 6.57 (dd, *J* = 0.7, 8.0 Hz, 1H), 6.35 (dd, *J* = 1.4, 7.7 Hz, 1H), 6.26 (ddd, *J* = 0.8, 7.5, 7.5 Hz, 1H), 4.45 (dd, *J* = 1.9, 14.6 Hz, 1H), 4.31 (dd, *J* = 1.7, 14.2 Hz, 1H), 4.18 (dd, *J* = 1.3, 14.7 Hz, 1H), 4.08 (dd, *J* = 1.2, 14.6 Hz, 1H), 3.30 (s, 2H), 2.57 (s, 3H), 2.51 (s, 3H), 2.43 (s, 3H), 2.23 (s, 3H), 1.10 (s, 9H); ^{13}C NMR (101 MHz, $CDCl_3$) δ 166.6, 155.1, 151.4, 143.5, 143.3, 139.1, 139.0, 134.0, 133.6, 133.5, 132.7, 131.7, 130.9, 130.6, 130.1, 129.7, 129.3, 129.2, 129.0, 128.82, 128.79, 128.5, 127.6, 126.5, 125.8, 125.6, 124.6, 124.3, 123.4, 123.2, 118.9, 117.9, 115.2, 54.2, 53.9, 38.3, 35.0, 33.9, 30.3, 21.6, 18.9; HRMS (FD) calcd for $C_{45}H_{45}N_3O_4S$ $[M]^+$ 723.3131, found 723.3116; CHIRALPAK IF-3, *n*-hexane/2-PrOH = 90:10, 1.0 mL/min, retention times: 30.7 min (major isomer) and 40.6 min (minor isomer).

2.4. Synthetic Transformations

2.4.1. Oxidation

(+)-Methyl 6-(2-(*tert*-butyl)-6-formylphenoxy)-7-(2-hydroxyphenyl)-4-methyl-2-tosylisoindoline-5-carboxylate [(+)-5]

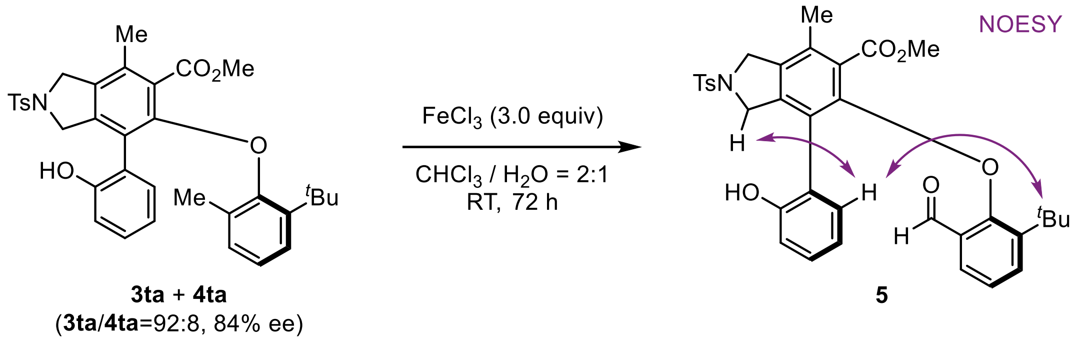

To a solution of **3ta** + **4ta** [12.1 mg, **3ta**/**4ta** = 92:8, 0.0186 mmol of **3ta** (84% *ee*)] in $CHCl_3$ (4.0 mL) and $FeCl_3$ (9.7 mg, 0.060 mmol) was added dropwise H_2O (2.0 mL) at 0 °C. The resulting mixture was stirred at room temperature for 72 h. The reaction was quenched by the addition of aqueous $Na_2S_2O_3$ at 0 °C and extracted with CH_2Cl_2. The combined organic layer was washed with brine, dried over Na_2SO_4, and concentrated. The residue was purified by silica gel PTLC (*n*-hexane/CH_2Cl_2/Et_2O = 2:2:1), which furnished (+)-**5** (2.3 mg, 0.0038 mmol, 20 % yield from **3ra**, 83% *ee*) and recovered **3ta** + **4ta** (1.2 mg, 0.0020 mmol, **3ta**/**4ta** = 84:16, 9% recovery of **3ta**).

Colorless oil; $[\alpha]^{25}_D$ + 4.8 (*c* 0.04, $CHCl_3$, 84% *ee*); ^1H NMR (400 MHz, $CDCl_3$) δ 9.62 (s, 1H), 7.73 (d, *J* = 8.3 Hz, 2H), 7.59 (dd, *J* = 1.7, 7.8 Hz, 1H), 7.50 (dd, *J* = 1.7, 7.6 Hz, 1H), 7.34 (d, *J* = 7.9 Hz, 2H), 7.23–7.19 (m, 3H), 7.02 (dd, *J* = 1.7, 7.5 Hz, 1H), 6.93 (ddd, *J* = 1.2, 7.4, 7.4 Hz, 1H), 6.89 (dd, *J* = 0.9, 8.2 Hz, 1H), 4.65 (dd, *J* = 2.5, 12.9 Hz, 1H), 4.60 (dd, *J* = 2.5, 14.5 Hz, 1H), 4.53 (dd, *J* = 1.8, 13.1 Hz, 1H), 4.33 (dd, *J* = 1.5, 14.3 Hz, 1H), 3.11 (s, 3H), 2.43 (s, 3H), 2.03 (s, 3H), 1.09 (s, 9H); ^{13}C NMR (101 MHz, $CDCl_3$) δ 191.6, 166.8, 153.9, 152.4, 151.0, 143.9, 143.4, 140.0, 133.9, 133.7, 132.6, 131.5, 130.3, 130.2, 130.03, 129.99, 127.5, 126.4, 124.2, 121.8, 121.3, 120.3, 119.4, 116.9, 53.9, 53.5, 51.5, 30.0, 29.7, 29.2, 21.5; HRMS (ESI) calcd for $C_{35}H_{35}NO_7S$ $[M+Na]^+$ 636.2032, found 636.2018; CHIRALPAK IF-3, *n*-hexane/2-PrOH = 85:15, 1.0 mL/min, retention times: 18.4 min (minor isomer) and 33.0 min (major isomer).

2.4.2. Reduction

(–)-(7-(2-Aminophenyl)-6-(2-(*tert*-butyl)-6-methylphenoxy)-4-methyl-2-tosylisoindolin-5-yl)methanol [(–)-6]

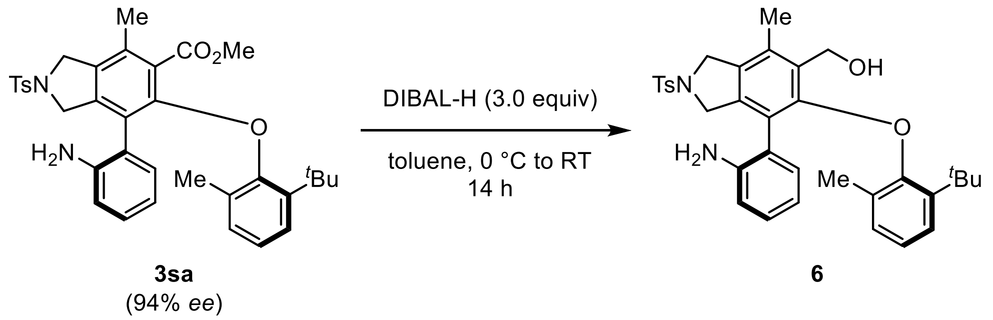

To a solution of (–)-**3sa** (119.0 mg, 0.1987 mmol) in toluene (5.0 mL) was added dropwise a 1.0 M solution of DIBAL-H in hexane (0.60 mL, 0.60 mmol of DIBAL-H) at 0 °C. After stirring at 0 °C for 2 h, the resulting mixture was stirred at room temperature for 15 h. The reaction was quenched by the addition of aqueous Rochelle salt and EtOAc at 0 °C, stirred for 0.5 h, and extracted with EtOAc. The combined organic layer was washed with brine, dried over Na_2SO_4, and concentrated. The

residue was purified by silica gel PTLC (*n*-hexane/EtOAc = 2:1), which furnished (–)-**6** (88.2 mg, 0.155 mmol, 78% yield, 92% *ee*).

Pale brown solid; mp 115.7–117.7 °C; $[\alpha]^{25}_{D}$ –192.9 (*c* 1.25, $CHCl_3$, 92% *ee*); ^1H NMR (400 MHz, $CDCl_3$) δ 7.74 (d, *J* = 8.2 Hz, 2H), 7.31 (d, *J* = 8.0 Hz, 2H), 7.15 (dd, *J* = 2.0, 7.4 Hz, 1H), 7.08 (ddd, *J* = 1.1, 7.7, 7.7 Hz, 1H), 6.91 (dd, *J* = 7.4, 7.4 Hz, 1H), 6.87 (dd, *J* = 2.1, 7.6 Hz, 1H), 6.83 (dd, *J* = 1.4, 7.5 Hz, 1H), 6.69–6.64 (m, 2H), 4.67 (dd, *J* = 1.8, 12.9 Hz, 1H), 4.57 (dd, *J* = 1.3, 13.1 Hz, 1H), 4.47 (dd, *J* = 1.8, 14.1 Hz, 1H), 4.40 (d, *J* = 12.6 Hz, 1H), 4.29–4.21 (m, 2H), 3.47 (br, 2H), 2.42 (s, 3H), 2.26 (s, 3H), 2.17 (s, 1H), 1.80 (s, 3H), 1.26 (s, 9H); ^{13}C NMR (101 MHz, $CDCl_3$) δ 156.0, 153.4, 144.2, 143.6, 139.5, 137.8, 134.7, 134.1, 131.3, 130.8, 129.8, 129.2, 128.9, 128.1, 127.6, 126.0, 125.8, 123.4, 123.0, 121.5, 118.4, 115.4, 57.0, 53.8, 53.7, 35.3, 30.1, 21.5, 18.8, 15.8; HRMS (FD) calcd for $C_{34}H_{38}N_2O_4S$ $[M]^+$ 570.2552, found 570.2542; CHIRALPAK IG-3, *n*-hexane/2-PrOH = 80:20, 1.0 mL/min, retention times: 24.5 min (major isomer) and 29.6 min (minor isomer).

2.4.3. Mitsunobu Reaction

(–)-2-(5-(2-(*tert*-Butyl)-6-methylphenoxy)-7-methyl-6-((p-tolyloxy)methyl)-2-tosylisoindolin-4-yl)aniline [(–)-7]

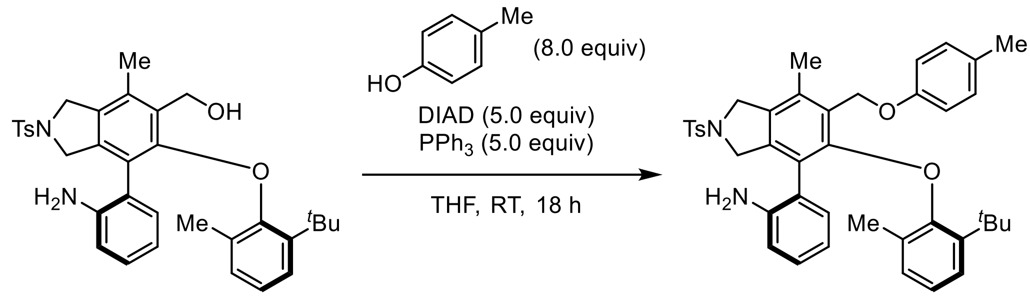

6 **7**

Diisopropyl azodicarboxylate (DIAD, 16.8 mg, 0.0829 mmol) was added to a solution of **6** (9.5 mg, 0.017 mmol), 4-methylphenol (14.4 mg, 0.133 mmol), and PPh_3 (21.8 mg, 0.0829 mmol) in THF (2.0 mL) at 0 °C. After stirring at room temperature for 16 h, the reaction mixture was concentrated. The residue was purified by silica gel PTLC (*n*-hexane/CH_2Cl_2/EtOAc = 5:5:1), which furnished (–)-**7** (9.3 mg, 0.014 mmol, 85% yield, 93% *ee*).

Pale yellow solid; mp 94.0–96.0 °C; $[\alpha]^{25}_{D}$ –145.2 (*c* 0.46, $CHCl_3$, 93% *ee*); ^1H NMR (400 MHz, $CDCl_3$) δ 7.74 (d, *J* = 8.2 Hz, 2H), 7.32 (d, *J* = 8.0 Hz, 2H), 7.06 (ddd, *J* = 1.2, 7.7, 7.7 Hz, 1H), 6.99 (dd, *J* = 2.0, 7.4 Hz, 1H), 6.95 (d, *J* = 8.2 Hz, 2H), 6.79 (dd, *J* = 1.3, 7.6 Hz, 1H), 6.73 (dd, *J* = 1.7, 7.4 Hz, 1H), 6.69 (dd, *J* = 7.5, 7.5 Hz, 1H), 6.67 (d, *J* = 7.4 Hz, 1H), 6.61 (dd, *J* = 7.5, 7.5 Hz, 1H), 6.46 (d, *J* = 8.6 Hz, 2H), 4.68 (d, *J* = 13.9 Hz, 1H), 4.64–4.55 (m, 3H), 4.43 (d, *J* = 15.7 Hz, 1H), 4.33 (d, *J* = 13.9 Hz, 1H), 3.45 (br, 2H), 2.43 (s, 3H), 2.24 (s, 3H), 2.21 (s, 3H), 1.86 (s, 3H), 1.19 (s, 9H); ^{13}C NMR (101 MHz, $CDCl_3$) δ 156.4, 155.1, 153.7, 144.1, 143.6, 139.1, 138.5, 135.0, 134.0, 130.9, 130.8, 130.0, 129.8, 129.6, 129.3, 128.8, 127.6, 126.9, 124.9, 124.7, 123.0, 122.9, 121.3, 118.3, 115.3, 114.1, 61.6, 54.0, 53.7, 35.1, 30.9, 30.0, 20.4, 18.8, 16.1; HRMS (FD) calcd for $C_{41}H_{44}N_2O_4S$ $[M]^+$ 660.3022, found 660.3035; CHIRALPAK IF-3, *n*-hexane/2-PrOH = 92:8, 1.0 mL/min, retention times: 25.4 min (minor isomer) and 26.8 min (major isomer).

2.5. Stability of Axial Chirality

2.5.1. Racemization

The time-dependent *ee* decay at 100 °C in toluene was measured by HPLC and summarized in **Table S1** together with the half-life ($t_{1/2}^{rac}$) and rotational barrier of racemization ($\Delta G^{‡}$) obtained from the Eyring analysis. The corresponding Eyring plots are shown in **Figures S1–S4**.

Table S1. Time-dependent *ee* decay at 100 °C in toluene, half-life($t_{1/2}^{rac}$) and rotational barrier($\Delta G^{‡}$) of racemization.

**3aa**		**3ab**		**3ac**		**3ai**	
Time (min)	% *ee*	Time (min)	% *ee*	Time (min)	% *ee*	Time (min)	% *ee*
0	91.542	0	97.130	0	90.440	0	88.338
90	90.992	30	89.766	30	90.432	1425	87.712
180	90.482	60	86.802	60	90.360	1785	87.444
320	90.446	90	77.980	120	90.342	2580	84.502
410	89.476	120	77.590	180	90.264	2940	82.142
500	88.796	150	68.530	360	90.260	3320	80.800
590	88.352	180	64.986	720	90.240		
680	87.664	210	60.920	1440	89.824		
770	87.474	240	57.476	2160	89.784		
1370	84.818	300	50.734	7200	89.096		
1550	84.600	360	44.940	12960	87.738		
1790	83.696	420	39.722	17280	86.472		
1970	83.102	540	30.578				
		720	20.538				
		1440	3.810				
		2880	0.168				
$t_{1/2}^{rac}$ (h)	232.745	$t_{1/2}^{rac}$ (h)	5.181	$t_{1/2}^{rac}$ (h)	5229.014	$t_{1/2}^{rac}$ (h)	416.202
$\Delta G^{‡}$ (kcal mol^{-1})	32.907	$\Delta G^{‡}$ (kcal mol^{-1})	30.086	$\Delta G^{‡}$ (kcal mol^{-1})	35.215	$\Delta G^{‡}$ (kcal mol^{-1})	33.338

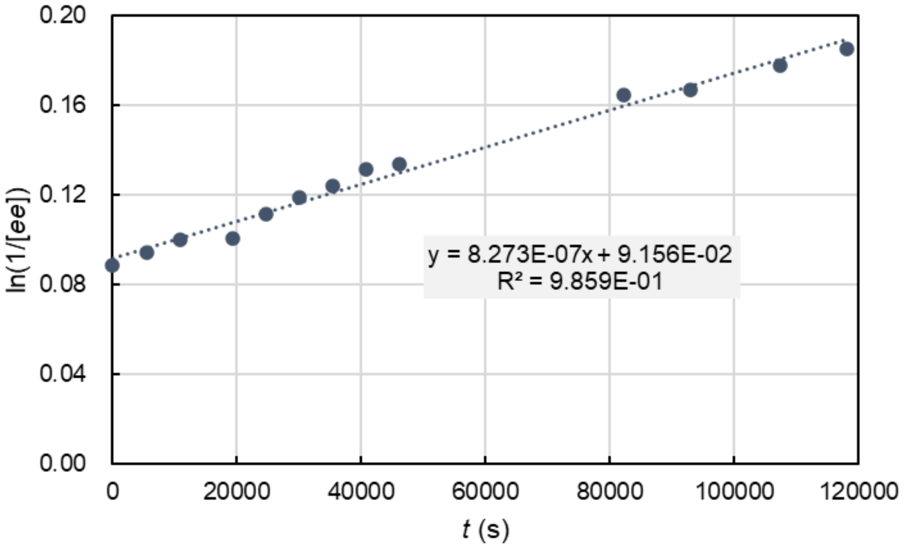

Figure S1. Eyring plots of **3aa**.

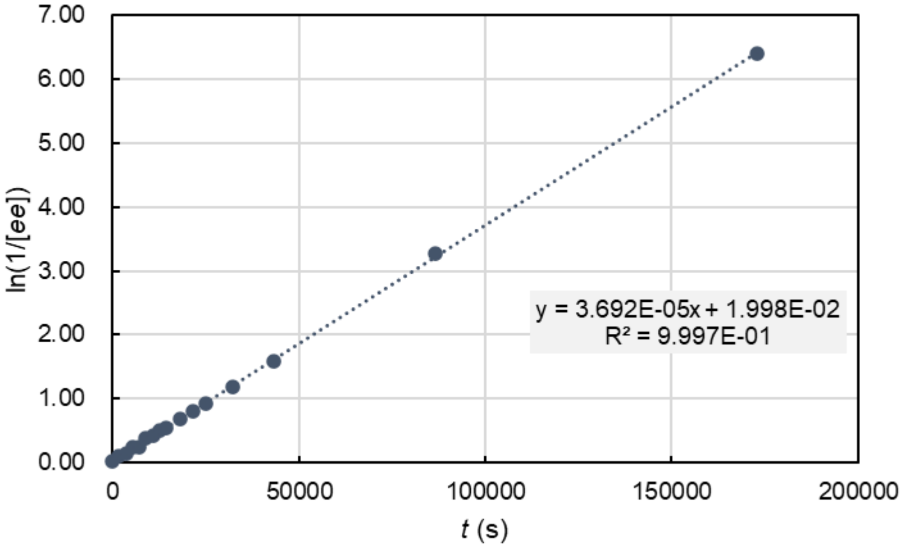

Figure S2. Eyring plots of **3ab**.

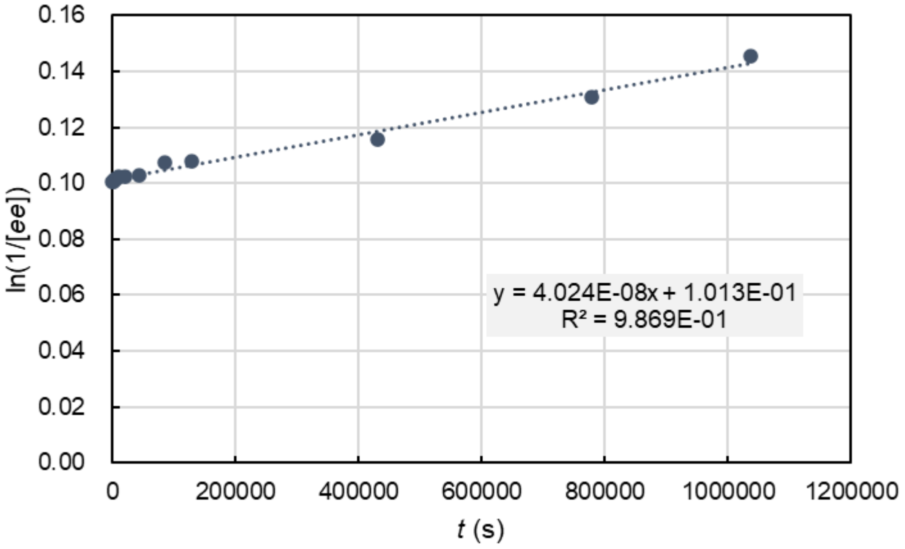

Figure S3. Eyring plots of **3ac**.

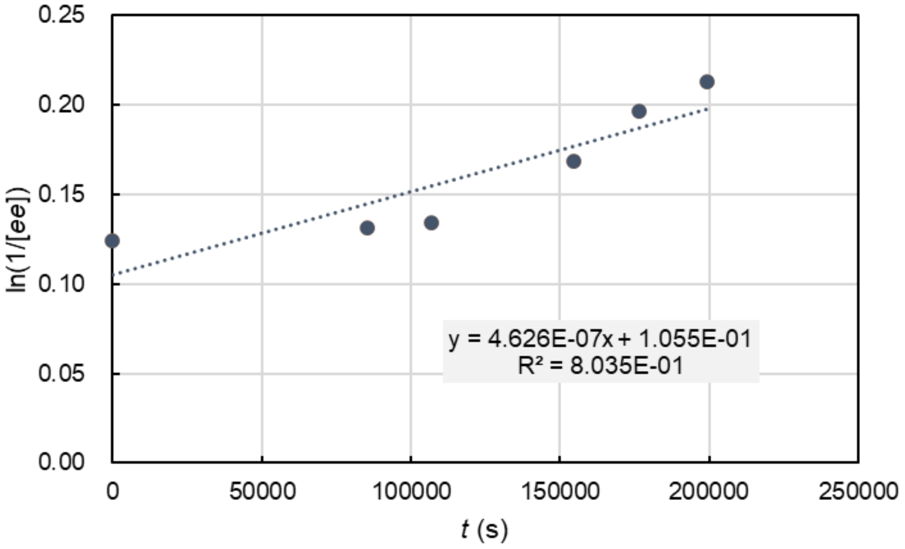

Figure S4. Eyring plots of **3ai**.

2.5.2. Epimerization

The time-dependent diastereomeric ratio decay at room temperature (**3oa**, **3ra**, **3sn**) or 60 °C (**3am**) in $CDCl_3$ was measured by ^{1}H NMR summarized in **Table S2** together with the half-life ($t_{1/2}^{epi}$) and rotational barrier of epimerization ($\Delta G^{‡}$) obtained from the Eyring analysis. The diastereomeric ratio and the difference of thermodynamic stability (ΔG) at the equilibrium state was also determined by the ^{1}H NMR. The corresponding Eyring plots are shown in **Figures S5–S8**.

Epimerization is considered as a reversible first order reaction. The kinetic parameters can be written as below.[17]

$$\mathrm{A} \underset{k'}{\overset{k}{\rightleftarrows}} \mathrm{B} \tag{1}$$

Considering the reversible reaction between A and B, the net rate of change of concentration A is

$$\frac{d[\mathrm{A}]}{dt} = -k[\mathrm{A}] + k'[\mathrm{B}] \tag{2}$$

If $[\mathrm{A}]_e$ and $[\mathrm{B}]_e$ are the concentration at the equilibrium state, and $[\mathrm{A}]_0$ and $[\mathrm{B}]_0$ are the concentration of the initial state, the following equation holds.

$$0 = -k[\mathrm{A}]_e + k'[\mathrm{B}]_e \tag{3}$$

Elimination of the k' of the Eq. (2) gives rise to

$$\frac{d[\mathrm{A}]}{dt} = -k\frac{[\mathrm{A}]_0 + [\mathrm{B}]_0}{[\mathrm{B}]_e}([\mathrm{A}] - [\mathrm{A}]_e) \tag{4}$$

Integration of this equation gives

$$\ln\frac{[\mathrm{A}] - [\mathrm{A}]_e}{[\mathrm{A}]_0 - [\mathrm{A}]_e} = -k\frac{[\mathrm{A}]_0 + [\mathrm{B}]_0}{[\mathrm{B}]_e}t + Const. \tag{5}$$

The rotational barrier of epimerization ($\Delta G^{‡}$) and the half-life ($t_{1/2}^{epi}$) can be obtained from the Eyring analysis using Eq. (5). The difference of thermodynamic stability (ΔG) of the diastereomers also calculated from the $[\mathrm{A}]_e$ and $[\mathrm{B}]_e$ as below.

$$\Delta G = -RT\exp\left(\frac{k'}{k}\right) = -RT\exp\left(\frac{[\mathrm{A}]_e}{[\mathrm{B}]_e}\right) \tag{6}$$

Table S2. Time-dependent diastereomeric ratio (dr) decay in $CDCl_3$, half-life($t_{1/2}^{epi}$), rotational barrier($\Delta G^{\ddagger}$) of epimerization and the difference of thermodynamic stability (ΔG).

**3qa** (RT)		**3ta** (RT)		**3am** (60 °C)		**3un** (RT)	
Time (h)	dr	Time (h)	dr	Time (h)	dr	Time (h)	dr
0.0	0.880:0.120	0.0	0.880:0.120	0.0	0.891:0.109	0.0	0.983:0.017
0.2	0.867:0.133	0.5	0.775:0.225	0.2	0.859:0.141	0.3	0.970:0.030
0.5	0.859:0.141	1.0	0.699:0.301	0.4	0.816:0.184	0.5	0.972:0.028
0.7	0.844:0.156	1.5	0.652:0.348	0.6	0.804:0.196	0.8	0.948:0.052
0.8	0.836:0.164	2.0	0.619:0.381	0.8	0.780:0.220	1.0	0.949:0.051
1.0	0.826:0.174	2.5	0.595:0.405	0.9	0.762:0.238	1.3	0.936:0.064
1.2	0.809:0.191	3.0	0.579:0.421	1.1	0.754:0.246	1.5	0.922:0.078
1.5	0.796:0.204	3.5	0.565:0.435	1.3	0.746:0.254	2.0	0.890:0.110
1.8	0.780:0.220	4.0	0.558:0.442	1.5	0.738:0.262	2.5	0.892:0.108
2.2	0.768:0.232	4.5	0.551:0.449	1.7	0.719:0.281	3.0	0.859:0.141
2.7	0.749:0.251			1.8	0.709:0.291	3.5	0.834:0.166
3.2	0.730:0.270						
3.7	0.712:0.288						
equilibrium	0.444:0.556	equilibrium	0.531:0.469	equilibrium	0.632:0.368	equilibrium	0.542:0.458
ΔG (kcal mol^{-1})	0.133	ΔG (kcal mol^{-1})	–0.074	ΔG (kcal mol^{-1})	–0.357	ΔG (kcal mol^{-1})	–0.112
$t_{1/2}^{epi}$ (h)	5.177	$t_{1/2}^{epi}$ (h)	1.207	$t_{1/2}^{epi}$ (h)	1.077	$t_{1/2}^{epi}$ (h)	6.017
$\Delta G^{\ddagger}$ (kcal mol^{-1})	23.828	$\Delta G^{\ddagger}$ (kcal mol^{-1})	23.067	$\Delta G^{\ddagger}$ (kcal mol^{-1})	25.933	$\Delta G^{\ddagger}$ (kcal mol^{-1})	26.927

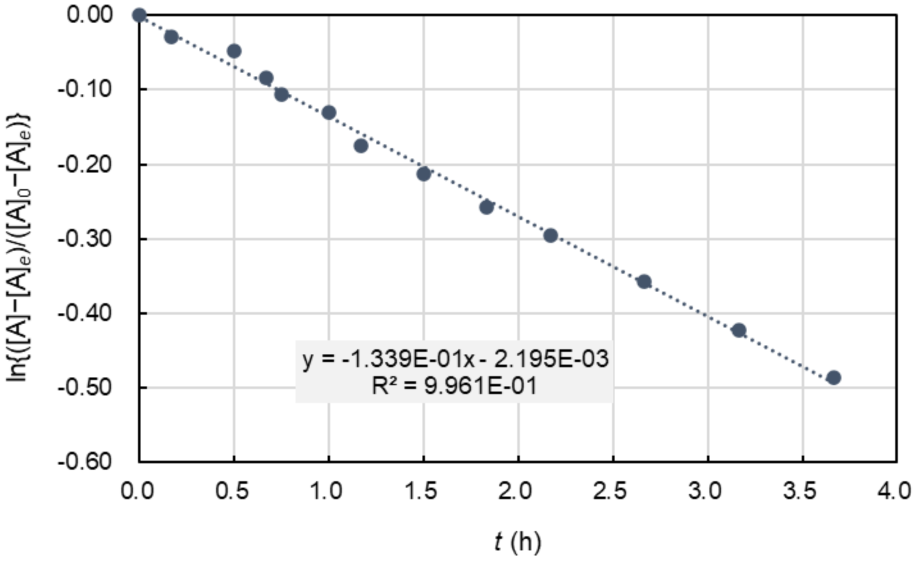

Figure S5. Eyring plots of **3qa**.

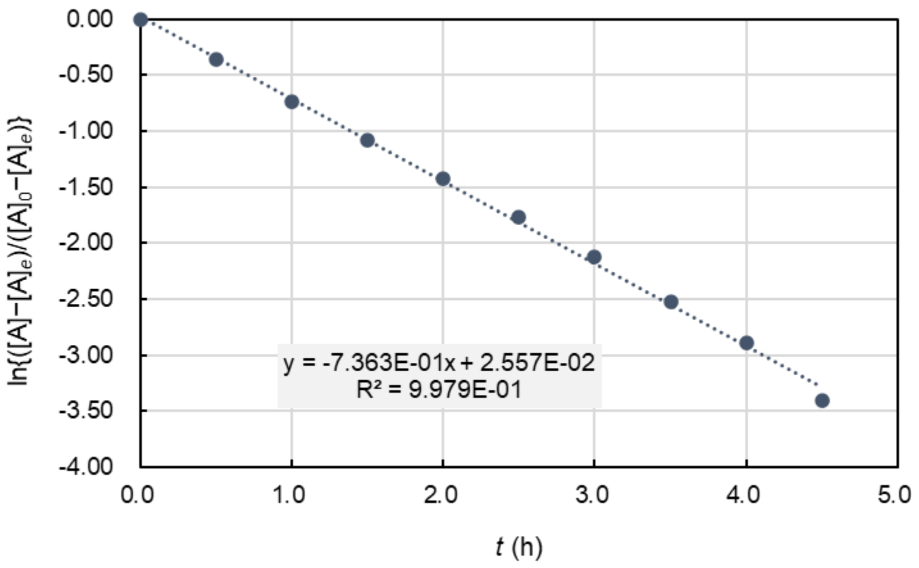

Figure S6. Eyring plots of **3ea**.

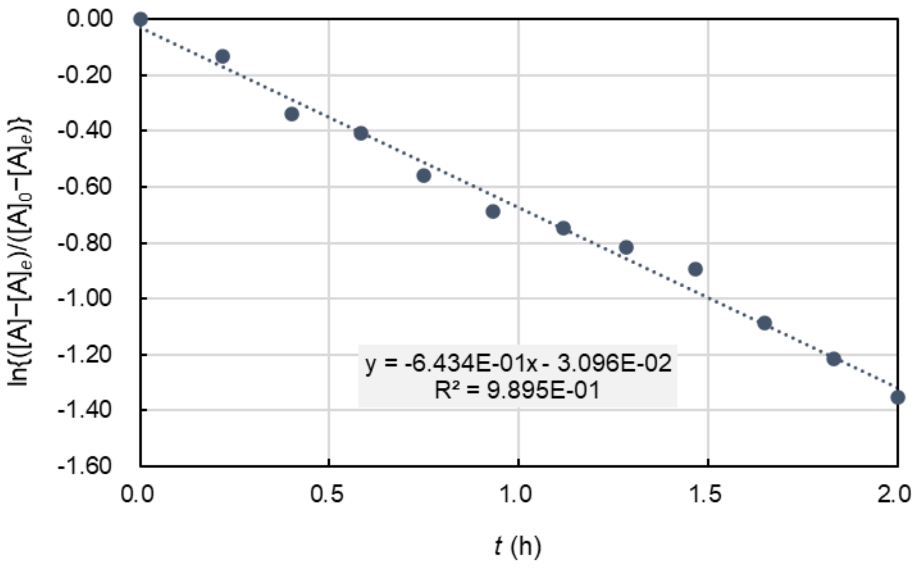

Figure S7. Eyring plots of **3am**.

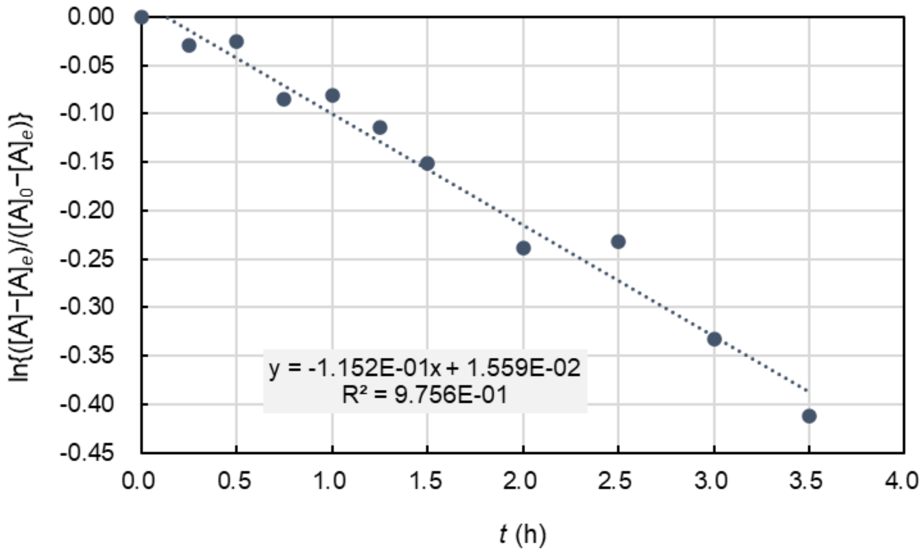

Figure S8. Eyring plots of **3un**.

2.6. Experimental Mechanistic Studies

General Procedure of Control Experiments (Figure 4): (*S*)-Difluorphos (3.4 mg, 0.0050 mmol) and $[Rh(cod)_2]BF_4$ (2.0 mg, 0.0050 mmol) were dissolved in CH_2Cl_2 (1.0 mL) in a Schlenk tube, and the mixture was stirred at room temperature for 10 min. After introduction of H_2 and stirring at room temperature for 30 min, the resulting mixture was concentrated to dryness. The residue was dissolved in CH_2Cl_2 (15.7 mL), followed by the addition of a solution of **1** (0.050 mmol) and **2** (0.100 mmol) in CH_2Cl_2 (1.0 mL, totally 3.0 mM for **1**) was added, and the mixture was stirred at room temperature for 16 h, then concentrated. The crude product was further by silica gel PTLC to furnish **3** and **4**.

2.6.1. Effect of Terminal Substituent on Monyne

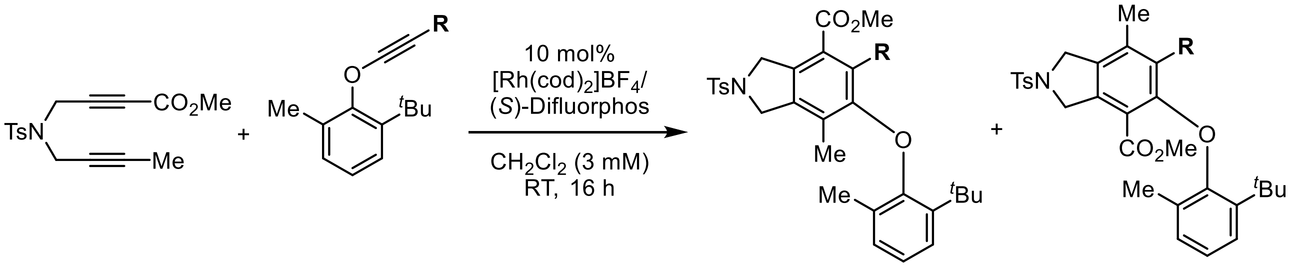

1a **2a** (R = CO_2Me) **2p** (R = CH_2OMe) **2q** (R = Me) (2 equiv) **3aa** / 37%, 90% *ee* **3ap** / 22%, 5% *ee* **3aq** / 3%, 18% *ee* **4aa** / 33%, 10% *ee* **4ap** / 29%, 14% *ee* **4aq** / 21%, 8% *ee*

(+)-Methyl 6-(2-(*tert*-butyl)-6-methylphenoxy)-5-(methoxymethyl)-7-methyl-2-tosylisoindoline-4-carboxylate [(+)-3ap]

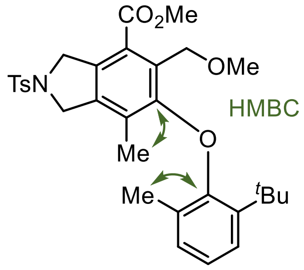

(+)-**3ap** (3.3 mg, 0.0059 mmol, 22% yield, 5% *ee*) and **4ap** (4.4 mg, 0.0080 mmol, 29% yield, 14% *ee*) were prepared from **1a** (8.7 mg, 0.027 mmol) and **2p** (12.7 mg, 0.0547 mmol) according to the general procedure of control experiment. Two regioisomers were isolated by silica gel PTLC twice (*n*-hexane/EtOAc = 4:1 and then *n*-hexane/CH_2Cl_2/EtOAc = 5:5:1), and **3ap** was further purified by silica gel PTLC (*n*-hexane/ EtOAc = 4:1).

Colorless oil; $[\alpha]^{25}_{D}$ +1.0 (*c* 0.16, $CHCl_3$, 5% *ee*); ^{1}H NMR (400 MHz, $CDCl_3$) δ 7.76 (d, *J* = 8.3 Hz, 2H), 7.32 (d, *J* = 8.0 Hz, 2H), 7.24 (dd, *J* = 1.7, 7.9 Hz, 1H), 6.97 (dd, *J* = 7.6, 7.6 Hz, 1H), 6.91 (dd, *J* = 1.2, 7.4 Hz, 1H), 4.74 (dd, *J* = 1.7, 14.1 Hz, 1H), 4.65 (d, *J* = 15.1 Hz, 1H), 4.57–4.51 (m, 3H), 4.46 (d, *J* = 15.6 Hz, 1H), 3.88 (s, 3H), 3.20 (s, 3H), 2.42 (s, 3H), 1.75 (s, 3H), 1.69 (s, 3H), 1.40 (s, 9H); ^{13}C NMR (101 MHz, $CDCl_3$) δ 167.7, 154.2, 152.7, 143.8, 139.4, 137.6, 133.8, 130.3, 129.90, 129.87, 127.8, 127.6, 127.2, 126.4, 125.4, 124.8, 123.4, 66.4, 58.5, 54.1, 53.3, 52.3, 35.3, 30.3, 21.5, 18.4, 14.3; HRMS (FD) calcd for $C_{31}H_{37}NO_6S$ $[M]^+$ 551.2342, found 551.2331; CHIRALPAK IE-3, *n*-hexane/^{i}PrOH = 70:30, 1.0 mL/min, retention times: 42.2 min (major isomer) and 75.5 min (minor isomer).

(–)-Methyl 5-(2-(*tert*-butyl)-6-methylphenoxy)-6-(methoxymethyl)-7-methyl-2-tosylisoindoline-4-carboxylate [(–)-4ap]

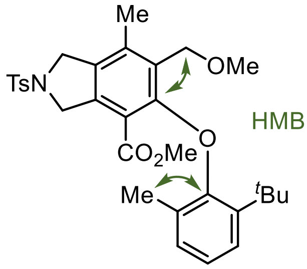

Colorless oil; $[\alpha]^{25}_{D}$ –7.3 (*c* 0.22, $CHCl_3$, 14% *ee*); ^1H NMR (400 MHz, $CDCl_3$) δ 7.75 (d, *J* = 8.3 Hz, 2H), 7.31 (d, *J* = 8.0 Hz, 2H), 7.22 (dd, *J* = 1.8, 7.6 Hz, 1H), 6.96 (dd, *J* = 7.5, 7.5 Hz, 1H), 6.92 (dd, *J* = 1.5, 7.5 Hz, 1H), 4.69 (s, 1H), 4.54–4.41 (m, 5H), 3.27 (s, 3H), 3.24 (s, 3H), 2.41 (s, 3H), 2.26 (s, 3H), 1.80 (s, 3H), 1.39 (s, 9H); ^{13}C NMR (101 MHz, $CDCl_3$) δ 166.1, 152.94, 152.93, 143.7, 141.0, 137.4, 136.4, 133.8, 130.3, 129.9, 129.7, 128.2, 127.6, 126.4, 125.1, 123.9, 115.2, 65.7, 58.4, 54.0, 53.2, 51.9, 35.4, 30.5, 21.5, 18.0, 16.0; HRMS (FD) calcd for $C_{31}H_{37}NO_6S$ $[M]^+$ 551.2342, found 551.2309; CHIRALPAK IE-3, *n*-hexane/^{i}PrOH = 80:20, 1.0 mL/min, retention times: 29.5 min (minor isomer) and 34.3 min (major isomer).

Methyl 6-(2-(*tert*-butyl)-6-methylphenoxy)-5,7-dimethyl-2-tosylisoindoline-4-carboxylate (3aq) and methyl 6-(2-(*tert*-butyl)-6-methylphenoxy)-5,7-dimethyl-2-tosylisoindoline-4-carboxylate (4aq)

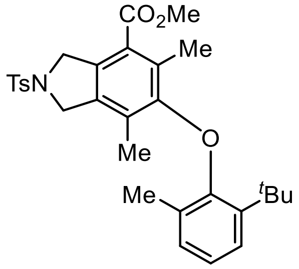

3aq

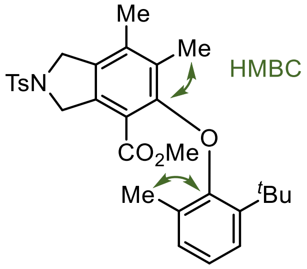

4aq

A mixture of **3aq** and **4aq** (6.0 mg, 0.012 mmol, 24% yield, **3aq**/**4aq** = 13:84, **3aq**: 18% *ee*, **4aq**: 8% *ee*) was prepared from **1a** (15.6 mg, 0.0488 mmol) and **2q** (20.5 mg, 0.101 mmol) according to the general procedure of control experiment and purified by silica gel PTLC (*n*-hexane/EtOAc = 2:1).

Colorless oil; $[\alpha]^{25}_{D}$ –2.1 (*c* 0.30, $CHCl_3$); ^1H NMR (400 MHz, $CDCl_3$) δ 7.78–7.75 (m, **3aq**: 2H, **4aq**: 2H), 7.32 (d, *J* = 8.0 Hz, **3aq**: 2H, **4aq**: 2H), 7.22 (dd, *J* = 1.8, 7.5 Hz, **3aq**: 1H, **4aq**: 1H), 6.96–6.89 (m, , **3aq**: 2H, **4aq**: 2H), 4.78 (d, *J* = 14.0 Hz, **3aq**: 1H), 4.68 (d, *J* = 15.0 Hz, **4aq**: 1H), 4.59–4.46 (m, **3aq**: 3H, **4aq**: 3H), 3.89 (s, **3aq**: 3H), 3.33 (s, **4aq**: 3H), 2.41 (s, **3aq**: 3H, **4aq**: 3H), 2.31 (s, **3aq**: 3H), 2.15 (s, **4aq**: 3H), 2.10 (s, **4aq**: 3H), 1.75 (s, **4aq**: 3H), 1.74 (s, **3aq**: 3H), 1.63 (s, **3aq**: 3H), 1.41 (s, **3aq**: 9H), 1.39 (s, **4aq**: 9H); ^{13}C NMR (101 MHz, $CDCl_3$) δ 166.5, 153.1, 152.3, 143.6, 140.5, 135.2, 133.9, 133.6, 130.2, 129.87, 129.86, 129.3, 127.6, 126.9, 125.2, 123.5, 115.2, 54.0, 53.3, 51.8, 35.4, 30.5, 21.5, 18.0, 16.8, 13.2; HRMS (FD) calcd for $C_{30}H_{35}NO_5S$ $[M]^+$ 521.2236, found 521.2211; CHIRALPAK IG-3, *n*-hexane/^{i}PrOH = 50:50, 1.0 mL/min, retention times: 9.6 min (major isomer of **4aq**), 12.5 min (minor isomer of **4aq**), 15.8 min (major isomer of **3aq**) and 20.8 min (minor isomer of **3aq**).

2.6.2. Effect of *ortho*-Substituent on Monoyne

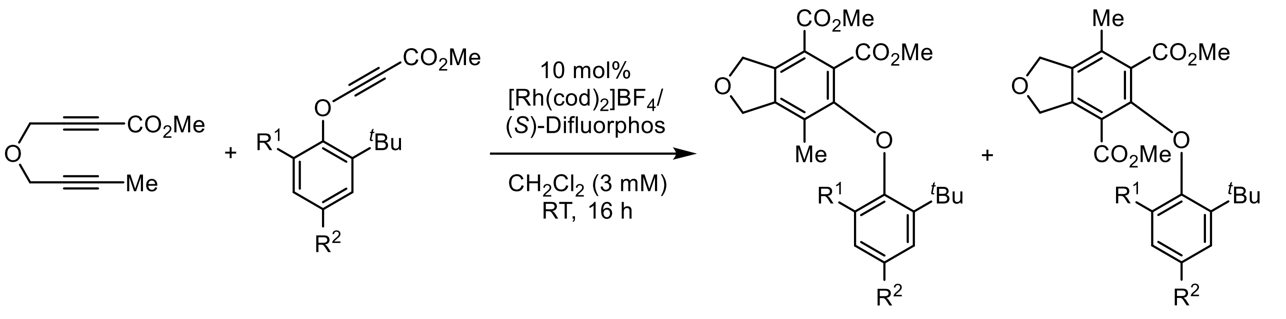

1e | **2a** (R^1 = Me, R^2 = H) **2b** (R^1 = OMe, R^2 = Me) (2 equiv) | **3ea** / 41%, 90% *ee* **3eb** / 28%, 95% *ee* | **4ea** / 15%, 13% *ee* **4eb** / 8%, 22% *ee*

(+)-**3eb** (6.2 mg, 0.014 mmol, 28% yield, 95% *ee*) and (+)-**4eb** (1.7 mg, 0.0038 mmol, 8% yield, 22% *ee*) were prepared from **1e** (8.3 mg, 0.050 mmol) and **2b** (27.5 mg, 0.0995 mmol) according to the general procedure of control experiment. Two regioisomers were isolated by silica gel PTLC (*n*-hexane/EtOAc = 2:1).

(+)-Dimethyl 6-(2-(*tert*-butyl)-6-methylphenoxy)-7-methyl-1,3-dihydroisobenzofuran-4,5-dicarboxylate [(+)-3ea]

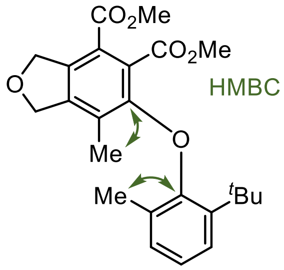

3ea (8.4 mg, 0.020 mmol, 41% yield, 90% *ee*) and **4ea** (3.1 mg, 0.0074 mmol, 15% yield, 13% *ee*) were prepared from **1e** (8.3 mg, 0.050 mmol) and **2a** (24.6 mg, 0.0999 mmol) according to the general procedure of control experiment. Two regioisomers were isolated by silica gel PTLC (*n*-hexane/EtOAc = 2:1).

Pale yellow oil; $[\alpha]^{25}_D$ +157.9 (*c* 0.42, $CHCl_3$, 90% *ee*); ^{1}H NMR (400 MHz, $CDCl_3$) δ 7.23 (dd, *J* = 1.7, 7.7 Hz, 1H), 6.99 (dd, *J* = 7.6, 7.6 Hz, 1H), 6.94 (dd, *J* = 1.2, 7.4 Hz, 1H), 5.38 (ddd, *J* = 2.1, 2.2, 14.0 Hz, 1H), 5.31 (ddd, *J* = 2.1, 2.1, 14.1 Hz, 1H), 5.02 (s, 2H), 3.88 (s, 3H), 3.83 (s, 3H), 1.90 (s, 3H), 1.67 (s, 3H), 1.40 (s, 9H); ^{13}C NMR (101 MHz, $CDCl_3$) δ 167.9, 165.1, 153.2, 151.3, 142.7, 140.2, 134.6, 129.9, 128.8, 127.0, 125.4, 125.3, 124.0, 120.2, 75.7, 73.0, 52.5, 35.3, 30.9, 30.2, 17.8, 14.6; HRMS (FD) calcd for $C_{24}H_{28}O_6$ $[M]^+$ 412.1886, found 412.1900; CHIRALPAK IF-3, *n*-hexane/^{i}PrOH = 95:5, 1.0 mL/min, retention times: 11.9 min (minor isomer) and 14.0 min (major isomer).

(+)-Dimethyl 5-(2-(*tert*-butyl)-6-methylphenoxy)-7-methyl-1,3-dihydroisobenzofuran-4,6-dicarboxylate [(+)-4ea]

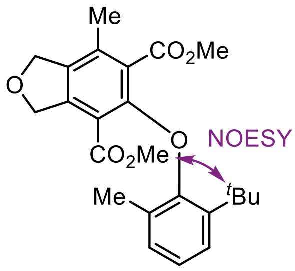

Pale yellow oil; $[\alpha]^{25}_D$ +7.6 (*c* 0.15, $CHCl_3$, 13% *ee*); ^{1}H NMR (400 MHz, $CDCl_3$) δ 7.22 (dd, *J* = 2.2, 7.3 Hz, 1H), 6.98 (dd, *J* = 7.4, 7.4 Hz, 1H), 6.95 (dd, *J* = 2.5, 7.9 Hz, 1H), 5.23 (ddd, *J* = 2.1,

2.1, 13.8 Hz, 1H), 5.07–5.00 (m, 3H), 3.67 (s, 3H), 3.33 (s, 3H), 2.17 (s, 3H), 1.96 (s, 3H), 1.39 (s, 9H); ^{13}C NMR (101 MHz, $CDCl_3$) δ 167.5, 165.8, 152.1, 151.5, 141.6, 141.5, 132.8, 132.3, 130.1, 128.7, 125.1, 125.0, 124.3, 113.7, 74.4, 73.0, 52.2, 51.8, 35.3, 30.4, 17.7, 16.7; HRMS (FD) calcd for $C_{24}H_{28}O_6$ $[M]^+$ 412.1886, found 412.1894; CHIRALPAK IG-3, *n*-hexane/^{i}PrOH = 95:5, 1.0 mL/min, retention times: 11.9 min (major isomer) and 13.9 min (minor isomer).

2.6.3. Competition Experiments

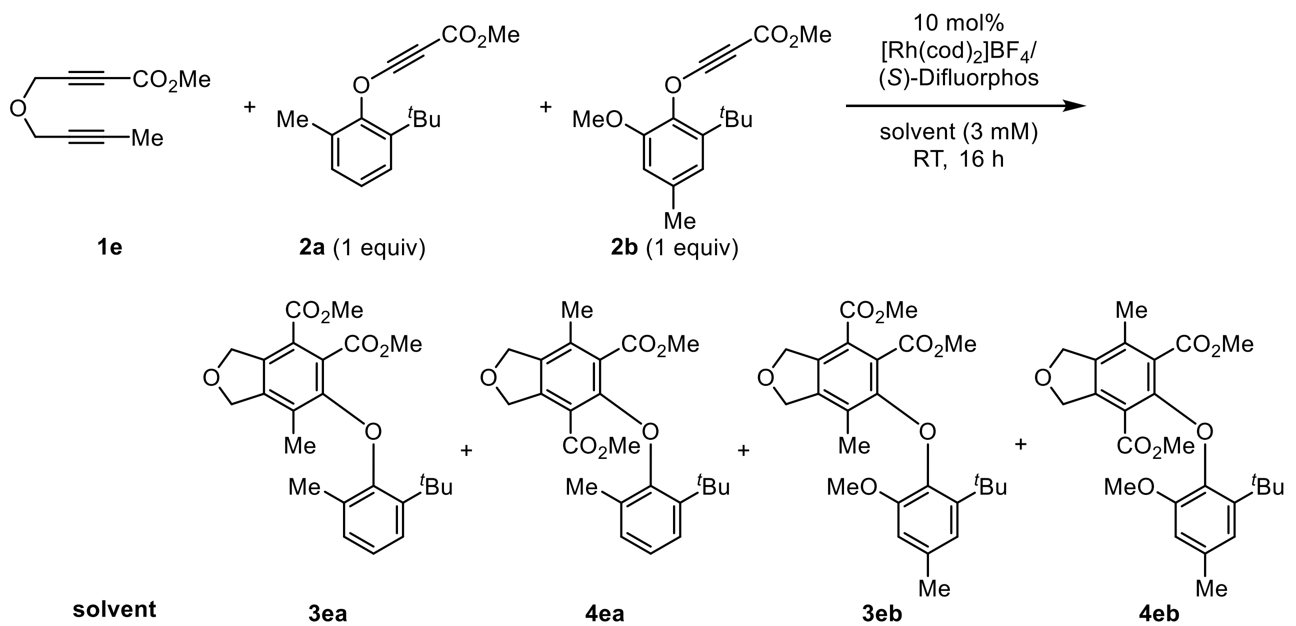

solvent	3ea	4ea	3eb	4eb
CH_2Cl_2	4%, 79% *ee*	5%, 26% *ee*	46%, 94% *ee*	13%, 24% *ee*
CH_2Cl_2/EtOH = 1:4	4%, 56% *ee*	3%, 1% *ee*	83%, 94% *ee*	8%, 13% *ee*

Solvent: CH_2Cl_2

(+)-**3ea** (0.9 mg, 0.0022 mmol, 4% yield, 79% *ee*), (+)-**4ea** (1.0 mg, 0.0023 mmol, 5% yield, 26% *ee*), (+)-**3eb** (10.2 mg, 0.0231 mmol, 46% yield, 94% *ee*) and (–)-**4eb** (2.9 mg, 0.0066 mmol, 13% yield, 24% *ee*) were prepared from **1e** (8.3 mg, 0.050 mmol), **2a** (12.5 mg, 0.0507 mmol), and **2b** (13.9 mg, 0.0503 mmol) according to the general procedure of **conditions B**. Products were isolated by silica gel PTLC twice (*n*-hexane/EtOAc = 2:1 and then *n*-hexane/EtOAc/CH_2Cl_2 = 2:1:1).

Solvent: CH_2Cl_2/EtOH = 1:4

(+)-**3ea** (0.8 mg, 0.0019 mmol, 4% yield, 56% *ee*), (+)-**4ea** (0.7 mg, 0.0017 mmol, 3% yield, 1% *ee*), (+)-**3eb** (18.4 mg, 0.0202 mmol, 83% yield, 94% *ee*) and (–)-**4eb** (1.6 mg, 0.0074 mmol, 8% yield, 13% *ee*) were prepared from **1e** (8.3 mg, 0.050 mmol), **2a** (12.4 mg, 0.0503 mmol), and **2b** (13.9 mg, 0.0503 mmol) according to the general procedure of c **conditions A**. Products were isolated by silica gel PTLC (*n*-hexane/EtOAc = 2:1). **4ea** was further purified by silica gel PTLC twice (*n*-hexane/ EtOAc/CH_2Cl_2 = 2:1:1 and then cyclohexane/EtOAc = 3:1). **4eb** was further purified by silica gel PTLC twice (*n*-hexane/EtOAc/CH_2Cl_2 = 2:1:1 and then cyclohexane/EtOAc = 3:1).

2.6.4. Observation of Rh Complexes in Various Solvents by NMR

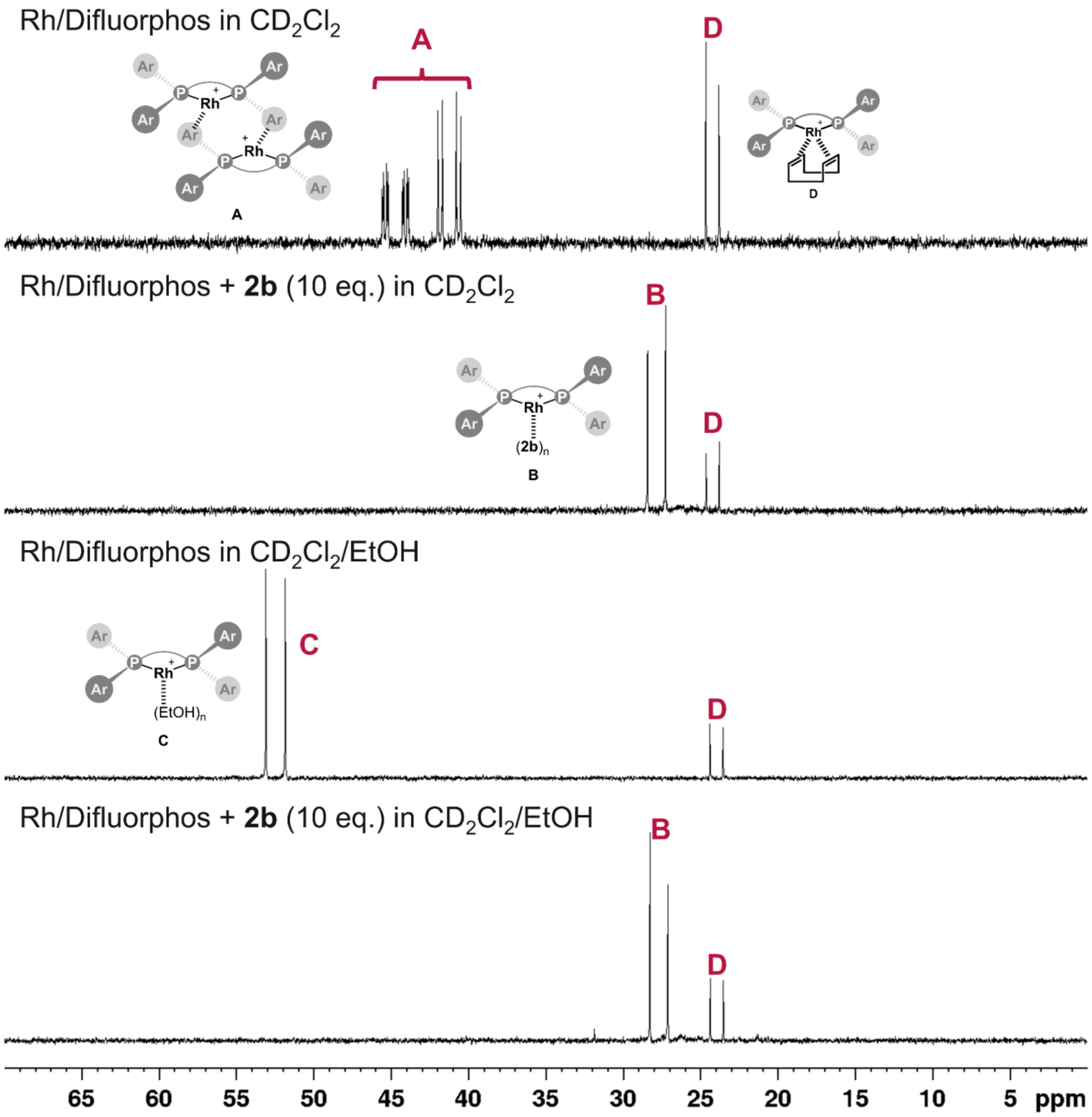

Figure S9. ^{31}P NMR (162 MHz) spectrum of Rh compexes in various conditions.

2.6.5. Reaction via in-situ Generation of Rh-Monoyne Complex

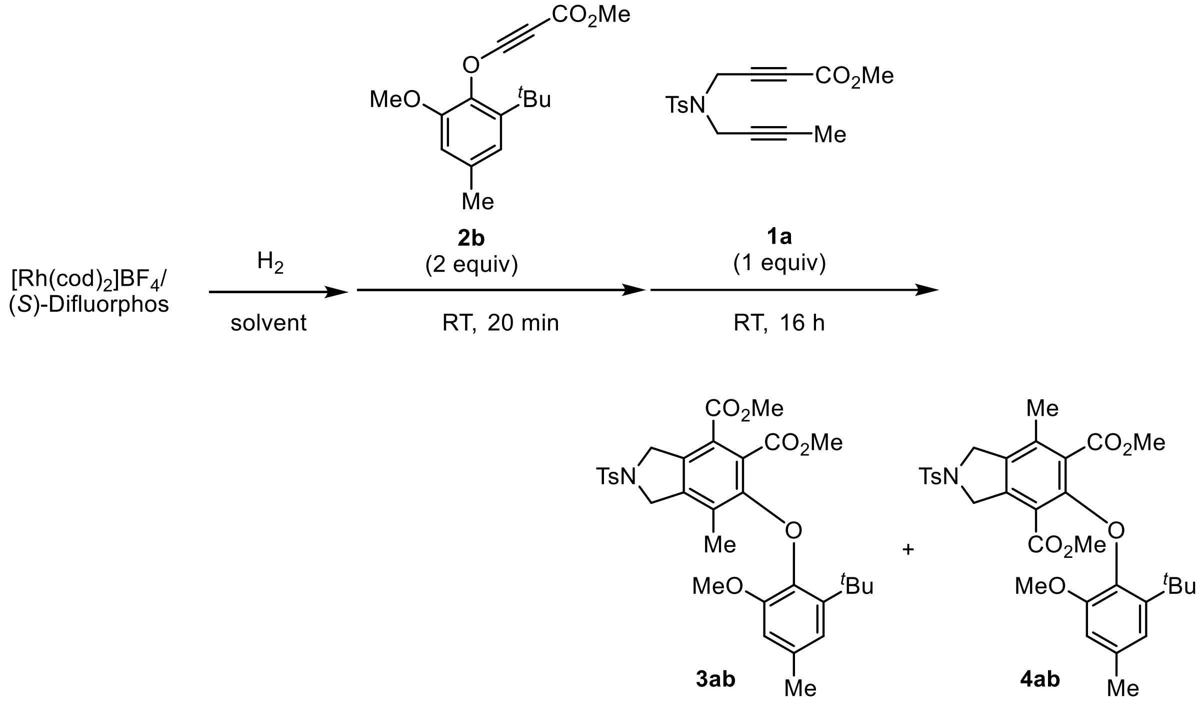

solvent	
CH_2Cl_2	8%, 97% *ee* (**3ab**/**4ab** = 90:10)
CH_2Cl_2/EtOH (1:4)	68%, 97% *ee* (**3ab**/**4ab** = 90:10)

(*S*)-Difluorphos (3.4 mg, 0.0050 mmol) and $[Rh(cod)_2]BF_4$ (2.0 mg, 0.0050 mmol) were dissolved in CH_2Cl_2 (1.0 mL) in a Schlenk tube, and the mixture was stirred at room temperature for 10 min. After the introduction of H_2 and stirring at room temperature for 30 min, the resulting mixture was concentrated to dryness. The residue was dissolved in a solution of **2b** in CH_2Cl_2 (11.7 mL) or CH_2Cl_2/EtOH (1:4, 11.7 mL), and the mixture was stirred at room temperature for 20 min. After the addition of a solution of **1a** in CH_2Cl_2 (5.0 mL) or CH_2Cl_2/EtOH (1:4, 5.0 mL), the mixture was stirred at room temperature for 16 h, then concentrated. The crude product was further purified by silica gel PTLC (*n*-hexane/ EtOAc = 2:1) to furnish **3ab** and **4ab**.

Solvent: CH_2Cl_2

3ab (2.5 mg, 0.0041 mmol, 8% yield, 97% *ee*) was prepared from **1a** (16.1 mg, 0.0483 mmol), **2b** (27.5 mg, 0.0995 mmol). The ratio of **3ab**/**4ab** was determined by ^1H NMR of the crude product.

Solvent: CH_2Cl_2/EtOH = 1:4

3ab (19.9 mg, 0.0334 mmol, 68% yield, 97% *ee*) was prepared from **1a** (15.8 mg, 0.0474 mmol), **2b** (27.6 mg, 0.0999 mmol). The ratio of **3ab**/**4ab** was determined by ^1H NMR of the crude product.

3. Single-Crystal X-Ray Diffraction Analysis

Single crystal X-ray diffraction data for **3gc**, **3na** and **3pa** were collected using a Rigaku XtaLAB Synergy R diffractometer equipped with a HyPix-6000HE Hybrid Pixel Array detector with monochromatized Mo-Kα (0.71073 Å) radiation. Data for **3am'** was collected using a Rigaku XtaLAB Synergy S diffractometer equipped with a HyPix-6000HE Hybrid Pixel Array detector with monochromatized Cu-Kα (1.54184 Å) radiation. The initial structure was solved by an intrinsic phasing method using SHELXT-2018/2[18] software and refined by a full matrix least-squares method using SHELXL-2018/3[19] software. All hydrogen atoms were located at geometrically calculated positions and included in least-squares calculations using riding models.

Details of the crystal data and the summaries of the intensity data collection parameters for **3gc**, **3na**, **3pa** and **3am'** are listed in Tables S3–S6 and structures are shown in Figures S10–S13. Single crystals suitable for X-ray analyses were grown by vapor diffusion of a Et_2O/*n*-heptane solution of **3gc**, a Et_2O/*n*-hexane solution of **3na** and an acetone/*n*-hexane solution of **3pa**, and by evaporation of solvent from a Et_2O solution of **3am'**.

Crystallographic data have been, deposited with the Cambridge Crystallographic Data Centre: Deposition code CCDC 2537265 [(+)-**3gc**], CCDC 2537266 [(–)-**3na**], CCDC 2537267 [(–)-**3pa**], and CCDC 2537268 [(–)-**3am'**]. They contain the supplementary crystallographic data for this paper. The data can be obtained free of charge from The Cambridge Crystallographic Data Centre via www.ccdc.cam.ac.uk/structures.

Table S3. Crystal data and data collection parameters of (*S*)-(+)-**3gc**.

Empirical formula	$C_{47.5}H_{49}F_6NO_5S$
Formula weight	859.93
Temperature/K	10.00(10)
Crystal system	orthorhombic
Space group	$P2_12_12_1$
a/Å	8.3571(2)
b/Å	16.6443(3)
c/Å	30.9663(6)
α /°	90
β /°	90
γ /°	90
V/Å^3	4307.35(15)
Z	4
ρ_{calc}/g cm^{-3}	1.326
μ/mm^{-1}	0.149
F(000)	1804.0
Crystal size/mm^3	0.39 × 0.25 × 0.19
Radiation	Mo Kα (λ = 0.71073)
2θ range for data collection/°	4.644 to 62.594
Index ranges	$-11 \leq h \leq 11$, $-20 \leq k \leq 24$, $-44 \leq l \leq 40$
Reflections collected	36853
Independent reflections	11883 [R_{int} = 0.0326, R_{sigma} = 0.0343]
Data/restraints/parameters	11883/0/522
Goodness-of-fit on F^2	1.056
Final R indexes [$I \geq 2\sigma(I)$]	R_1 = 0.0382, wR_2 = 0.0941
Final R indexes [all data]	R_1 = 0.0441, wR_2 = 0.0962
Largest diff. peak/hole.e. Å^{-3}	0.45/-0.37
Absolute structure parameter	-0.02(2)

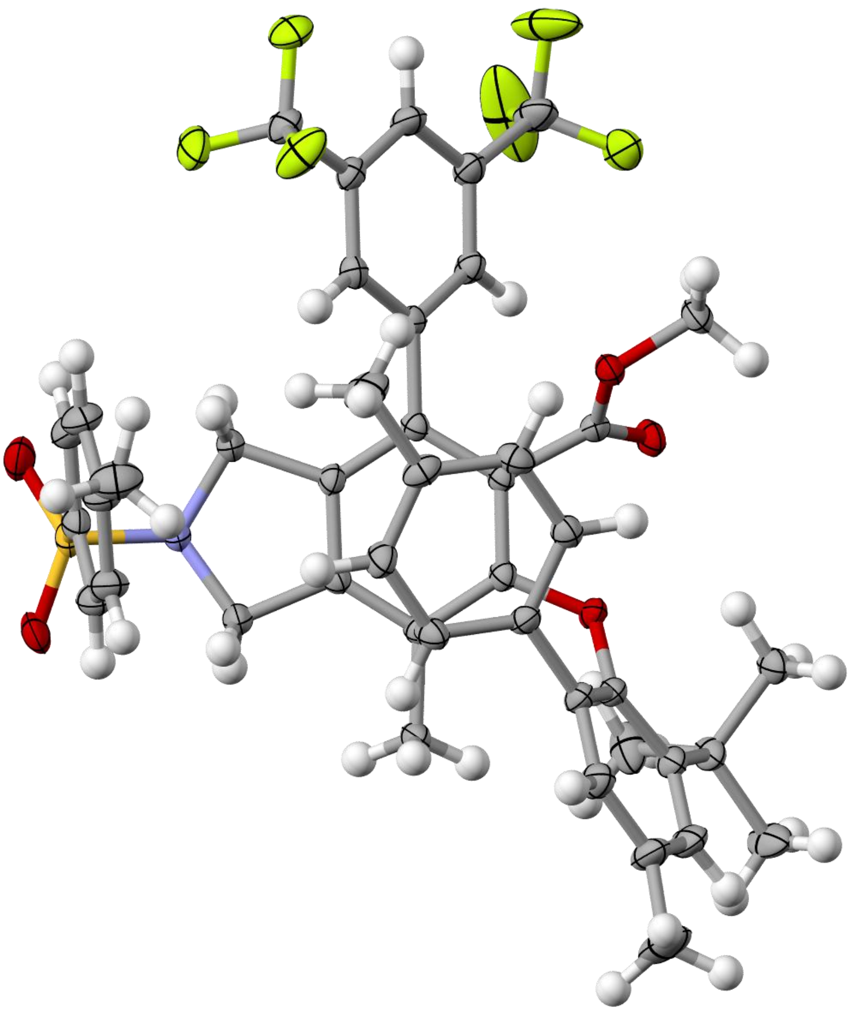

Figure S10. X-ray crystal structure of (*S*)-(+)-**3gc**, showing thermal ellipsoids at the 50% probability level.

Table S4. Crystal data and data collection parameters of (*S*,*R*)-(–)-**3na**.

Empirical formula	$C_{45}H_{46}NO_5S$
Formula weight	676.86
Temperature/K	100.00(10)
Crystal system	monoclinic
Space group	*I*2
a/Å	12.1550(2)
b/Å	8.37170(10)
c/Å	36.1188(6)
α /°	90
β /°	92.4120(10)
γ /°	90
V/Å^3	3672.12(10)
Z	4
ρ_{calc}/g cm^{-3}	1.224
μ/mm^{-1}	0.134
F(000)	1444.0
Crystal size/mm^3	0.57 × 0.2 × 0.15
Radiation	Mo Kα (λ = 0.71073)
2θ range for data collection/°	4.514 to 62.552
Index ranges	$-17 \leq h \leq 17$, $-11 \leq k \leq 11$, $-51 \leq l \leq 49$
Reflections collected	53973
Independent reflections	10275 [R_{int} = 0.0273, R_{sigma} = 0.0182]
Data/restraints/parameters	10275/1/450
Goodness-of-fit on F^2	1.025
Final R indexes [$I \geq 2\sigma(I)$]	R_1 = 0.0499, wR_2 = 0.1388
Final R indexes [all data]	R_1 = 0.0513 wR_2 = 0.1400
Largest diff. peak/hole.e. Å^{-3}	1.86/-0.36
Absolute structure parameter	0.012(12)

Alert level B

PLAT094_ALERT_2_B Ratio of Maximum / Minimum Residual Density .... 5.09 Report

Author Response: Due to the disorder of the solvent molecules.

PLAT097_ALERT_2_B Large Reported Max. (Positive) Residual Density 1.86 eA-3

Author Response: Due to the disorder of the solvent molecules.

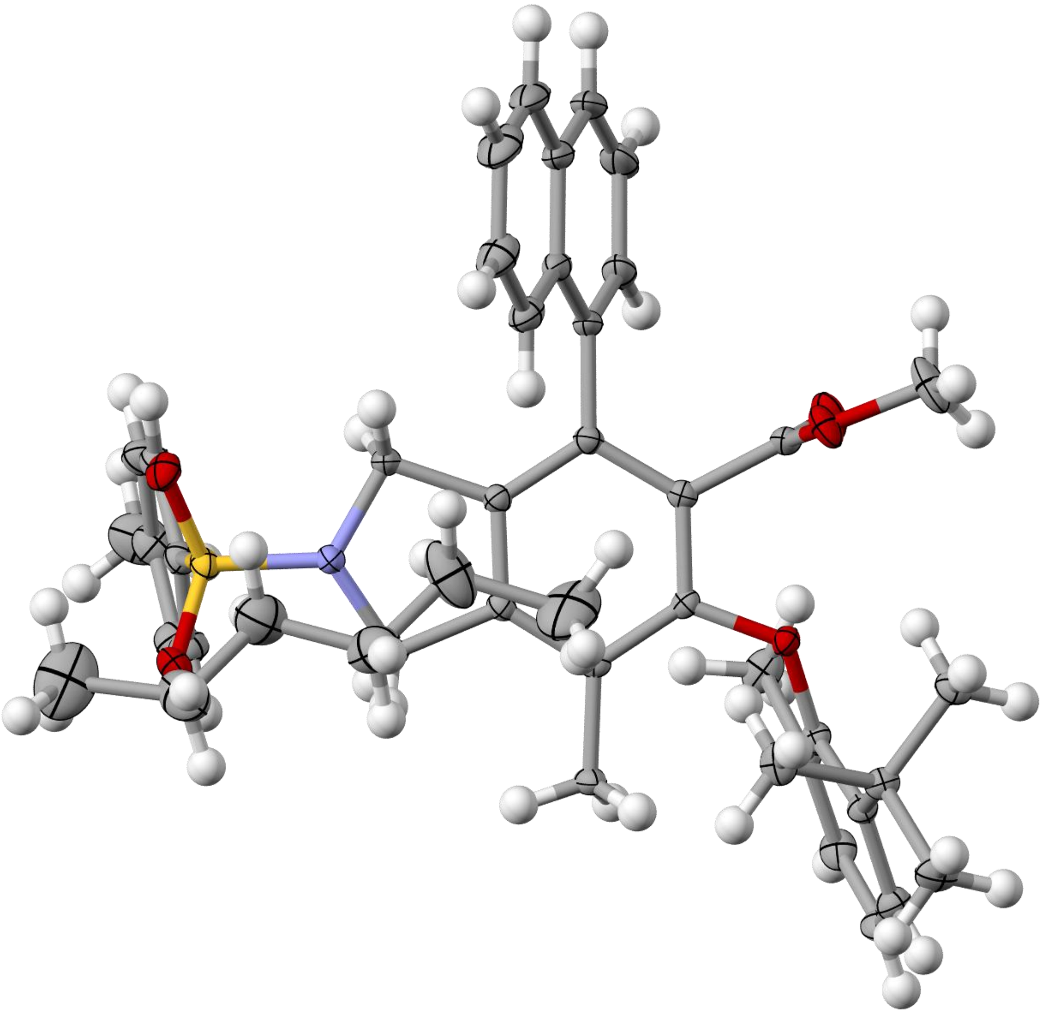

Figure S11. X-ray crystal structure of (*S*,*R*)-(–)-**3na**, showing thermal ellipsoids at the 50% probability level.

Table S5. Crystal data and data collection parameters of (*S*,*R*)-(–)-**3pa**.

Empirical formula	$C_{48}H_{48}NO_5S$
Formula weight	750.93
Temperature/K	100.02(18)
Crystal system	monoclinic
Space group	$P2_1$
a/Å	13.7461(4)
b/Å	8.0888(2)
c/Å	17.9637(5)
α /°	90
β /°	101.902(3)
γ /°	90
V/Å^3	1954.43(9)
Z	2
ρ_{calc}/g cm^{-3}	1.276
μ/mm^{-1}	0.133
F(000)	798.0
Crystal size/mm^3	0.37 × 0.07 × 0.05
Radiation	Mo Kα (λ = 0.71073)
2θ range for data collection/°	4.176 to 56.562
Index ranges	$-18 \leq h \leq 17$, $-10 \leq k \leq 10$, $-23 \leq l \leq 23$
Reflections collected	27844
Independent reflections	9625 [R_{int} = 0.0299, R_{sigma} = 0.0426]
Data/restraints/parameters	9625/1/476
Goodness-of-fit on F^2	1.046
Final R indexes [$I \geq 2\sigma(I)$]	R_1 = 0.0368, wR_2 = 0.0780
Final R indexes [all data]	R_1 = 0.0467, wR_2 = 0.0805
Largest diff. peak/hole.e. Å^{-3}	0.22/-0.31
Absolute structure parameter	0.03(2)

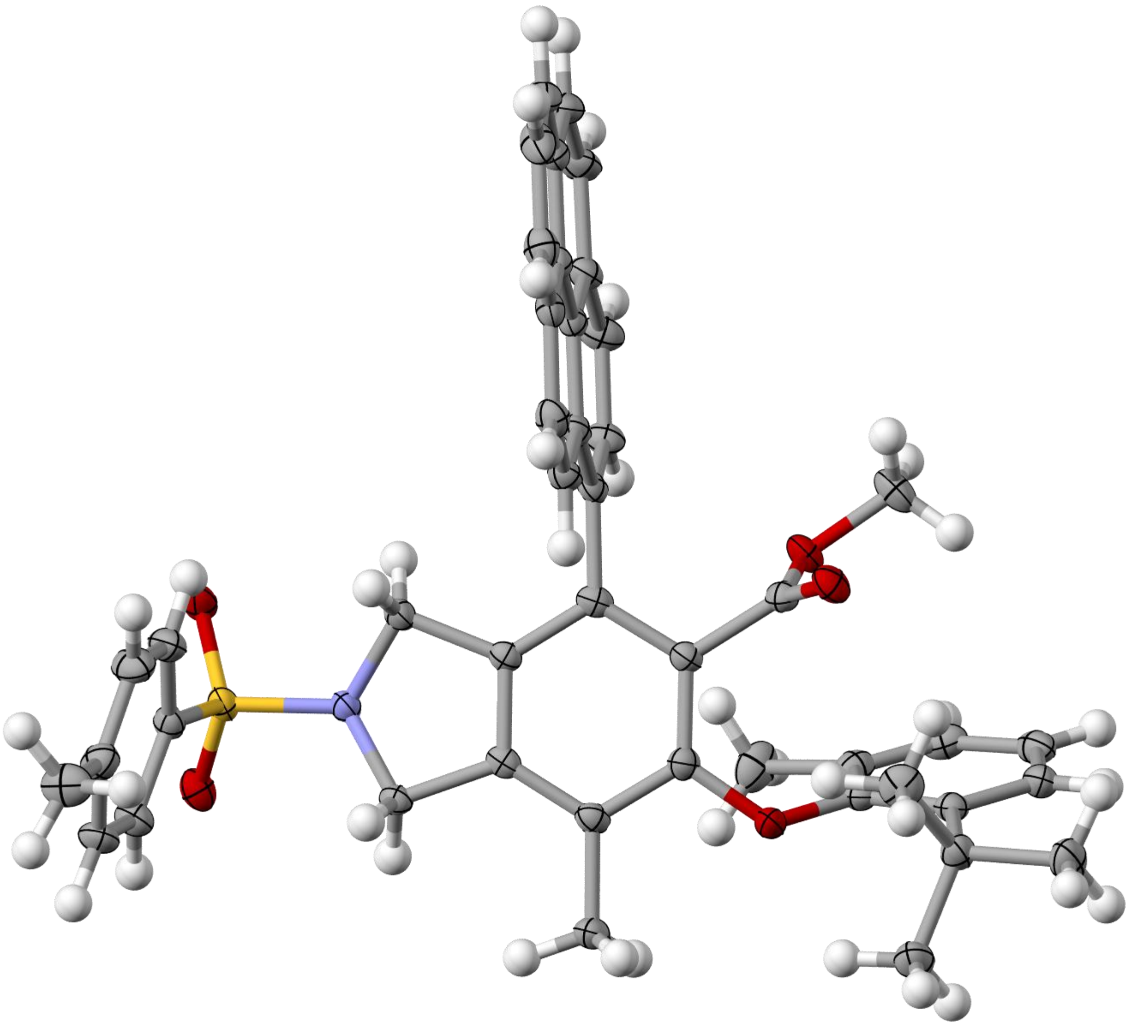

Figure S12. X-ray crystal structure of (*S*,*R*)-(–)-**3pa**, showing thermal ellipsoids at the 50% probability level.

Table S6. Crystal data and data collection parameters of (*R*,*S*)-(–)-**3am'**.

Empirical formula	$C_{33}H_{42.5}N_2O_{8.25}S$
Formula weight	631.25
Temperature/K	92(2)
Crystal system	triclinic
Space group	*P*1
a/Å	9.7364(3)
b/Å	10.1184(3)
c/Å	17.3259(4)
α /°	83.778(2)
β /°	89.005(2)
γ /°	87.208(2)
V/Å^3	1694.70(8)
Z	2
ρ_{calc}/g cm^{-3}	1.237
μ/mm^{-1}	1.277
F(000)	673.0
Crystal size/mm^3	0.11 × 0.1 × 0.06
Radiation	Cu K*α* (*λ* = 1.54184)
2*θ* range for data collection/°	8.8 to 155.016
Index ranges	$-12 \leq h \leq 11$, $-12 \leq k \leq 12$, $-21 \leq l \leq 21$
Reflections collected	46381
Independent reflections	12720 [R_{int} = 0.0419, R_{sigma} = 0.0310]
Data/restraints/parameters	12720/3/807
Goodness-of-fit on F^2	1.046
Final *R* indexes [$I \geq 2\sigma(I)$]	R_1 = 0.0437, wR_2 = 0.1125
Final *R* indexes [all data]	R_1 = 0.0458, wR_2 = 0.1138
Largest diff. peak/hole.e. Å^{-3}	0.29/-0.47
Absolute structure parameter	-0.007(8)

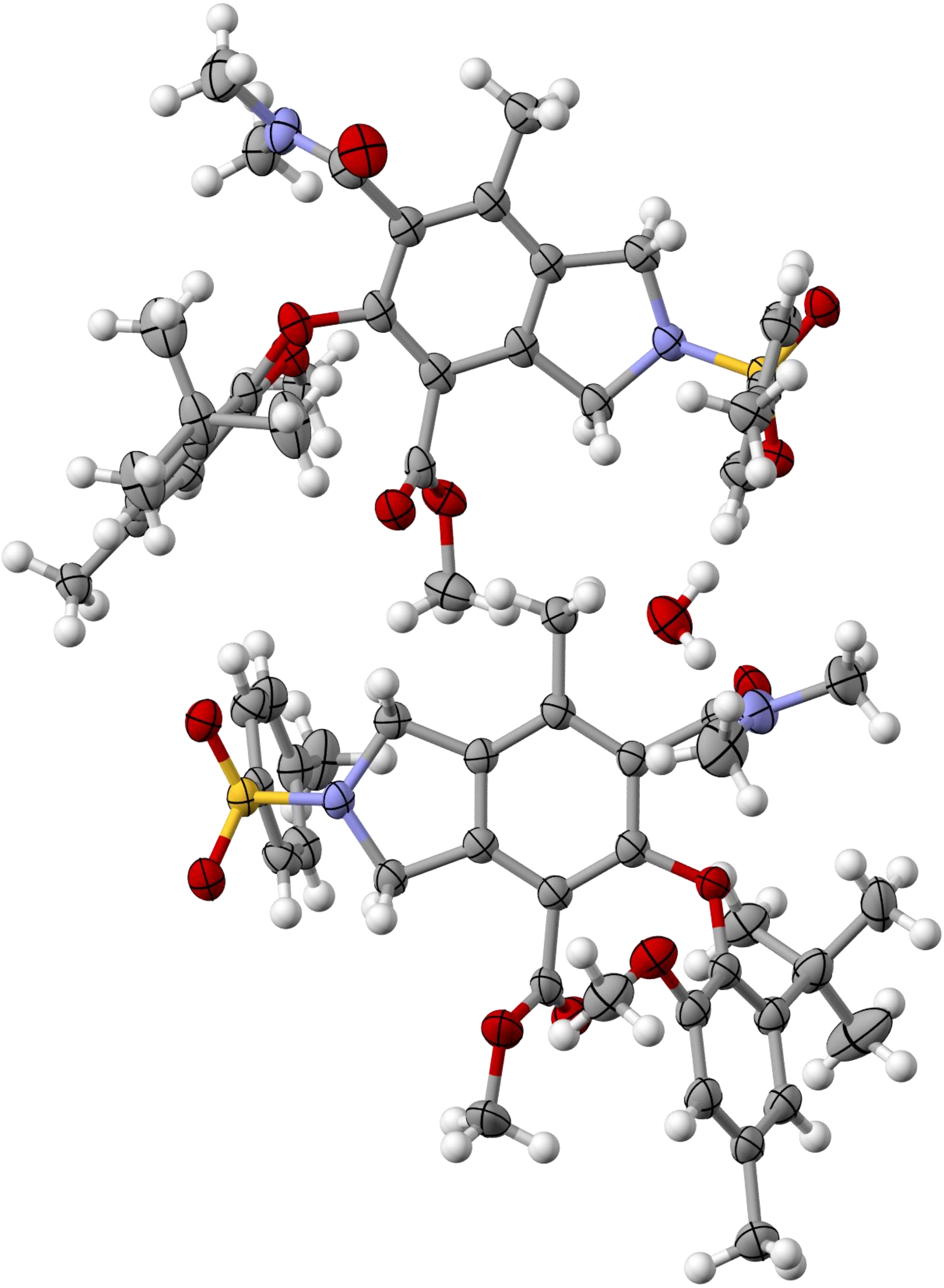

Figure S13. X-ray crystal structure of (*R*,*S*)-(–)-**3am’**, showing thermal ellipsoids at the 50% probability level.

4. Theoretical Calculations

4.1. Computational Methods

All the calculations were performed at the DFT level of theory with the B3LYP hybrid functional[20,21] as implemented in Gaussian 16.[22] To describe the dispersion properly, an explicit dispersion correction term (D3) developed by Grimme and co-workers,[23,24] was also employed in the DFT calculations. The Ahlrichs-type Def2-SVP basis sets[25,26] along with the associated ECP were used for C, H, O, F, P, and Rh atoms in both the geometry optimization and the corresponding frequency calculations.

Due to the evident π−π stacking interaction found in all transition states, to achieve a considerably high accuracy, the large basis sets with diffuse function, Def2-TZVPPD,[25,26] were then employed for the single-point calculations. Solvation effects were introduced using the IEF-PCM method[27,28] with dichloromethane as the solvent. Notably, the solvent accessible surface (SAS) was used in both the optimization and frequency calculations, while a better solvent excluded surface (SES) was then adopted during the single-point calculations.

The artificial force induced reaction (AFIR) method[29–32] was applied to explore all the pathways with accessible barriers. Because the transition states involved in this reaction are conformationally flexible, the transition-state (TS) conformation sampling calculations were conducted to ensure that the transition states discussed in this article adopt the most stable conformation. This sampling calculation was realized using the SC-AFIR method at the semi-empirical GFN-xTB[33] level of theory as implemented in ORCA 4.0 software package,[34], and a collision energy of 50 kJ/mol was applied to all the pendant functional groups for any possible conformational change.

For the Rh-catalyzed cyclization step, the number of conformers identified for each TS is as follows: **TS1a** (282), **TS1b** (260), **TS1c** (213), and **TS1d** (212). For the subsequent enantio-determining insertion step, the number of conformers identified for each TS is as follows: **TS3A** (448) and **TS3B** (465).

For those conformers having lowest GFN-xTB energies, further optimizations were subsequently conducted at the DFT level of theory as what was discussed previously. All the minima and transition states were fully optimized without any constraints. An intrinsic reaction coordinate (IRC) calculation[35] was performed for each transition state to confirm it connecting to the correct reactant and product. The free energies were computed at 298.15 K and 1 atm. All the geometries shown in this article are visualized by the CYLview software[36].

The plots for the qualitative noncovalent interactions (NCI) analysis[37] and the independent gradient model based on Hirshfeld partition (IGMH) analysis[38,39] were generated using Multiwfn[40,41] with VMD[42] using the optimized electron density at the B3LYP-D3/Def2-TZVPPD/IEFPCM(DCM) level of theory as the SCF energy correction. Energy decomposition analysis was performed with sobEDA[43] with Multiwfn.

4.2. Computational Energies of All Optimized Structures

Table S7. Sums of thermal free energies (*G*) and electronic energies (*E*) for optimized structures.

	G_{corr} (= $G' - E'$) (hartree) B3LYP-D3/Def2-SVP/PCM(DCM)	Imaginary frequency (cm^{-1}) B3LYP-D3/Def2-SVP/PCM(DCM)	*E* (hartree) B3LYP-D3/Def2-TZVPPD/PCM(DCM)	*G* (= $E + G_{corr}$) (hartree) B3LYP-D3/Def2-TZVPPD/PCM(DCM)
**Rh-Difluorphos**	0.455404	none	-2957.011864	-2956.556459
**diyne**	0.127877	none	-574.766331	-574.638455
**alkyne**	0.249072	none	-808.393335	-808.144262
**IM1a**	0.617122	none	-3531.821795	-3531.204674
**TS1a**	0.613301	-244.5547	-3531.802761	-3531.189460
**IM2a**	0.615583	none	-3531.853701	-3531.238118
**IM1b**	0.617184	none	-3531.819775	-3531.202591
**TS1b**	0.614869	-419.4970	-3531.798123	-3531.183254
**IM2b**	0.615757	none	-3531.842558	-3531.226801
**IM1c**	0.614921	none	-3531.810639	-3531.195718
**TS1c**	0.614485	-314.8995	-3531.798879	-3531.184394
**IM2c**	0.616294	none	-3531.847868	-3531.231574
**IM1d**	0.616231	none	-3531.820848	-3531.204617
**TS1d**	0.614126	-326.3273	-3531.791876	-3531.177750
**IM2d**	0.616955	none	-3531.842237	-3531.225282
**TS2a-c**	0.615855	-163.6798	-3531.832622	-3531.216767
**TS2a-d**	0.617488	-42.2077	-3531.837382	-3531.219894
**TS2b-c**	0.616050	-52.1888	-3531.834270	-3531.218220
**TS2b-d**	0.615900	-145.0052	-3531.824142	-3531.208242
**IM3A**	0.897324	none	-4340.281791	-4339.384467
**TS2A**	0.899311	-36.1117	-4340.279745	-4339.380434
**IM4A**	0.899895	none	-4340.284513	-4339.384618
**TS3A**	0.897403	-237.5036	-4340.275791	-4339.378388
**IM5A**	0.900906	none	-4340.308754	-4339.407848
**IM3A-V**	0.897626	none	-4340.283434	-4339.385808
**TS3A-V**	0.893564	-168.3949	-4340.263857	-4339.370293
**IM5A-V**	0.901521	none	-4340.314602	-4339.413082
**IM3B**	0.899247	none	-4340.283967	-4339.384720
**TS2B**	0.897695	-20.7185	-4340.278377	-4339.380683
**IM4B**	0.898107	none	-4340.279811	-4339.381704
**TS3B**	0.897784	-227.4975	-4340.271358	-4339.373574
**IM5B**	0.899383	none	-4340.306789	-4339.407406
**IM3B-V**	0.897821	none	-4340.283738	-4339.385918
**TS3B-V**	0.894946	-138.3495	-4340.266202	-4339.371256
**IM5B-V**	0.900004	none	-4340.312052	-4339.412048
**TS4A**	0.901300	-260.0247	-4340.284904	-4339.383604
**IM6A**	0.907456	none	-4340.422007	-4339.514551

4.3. Computational Studies for Reaction Pathways

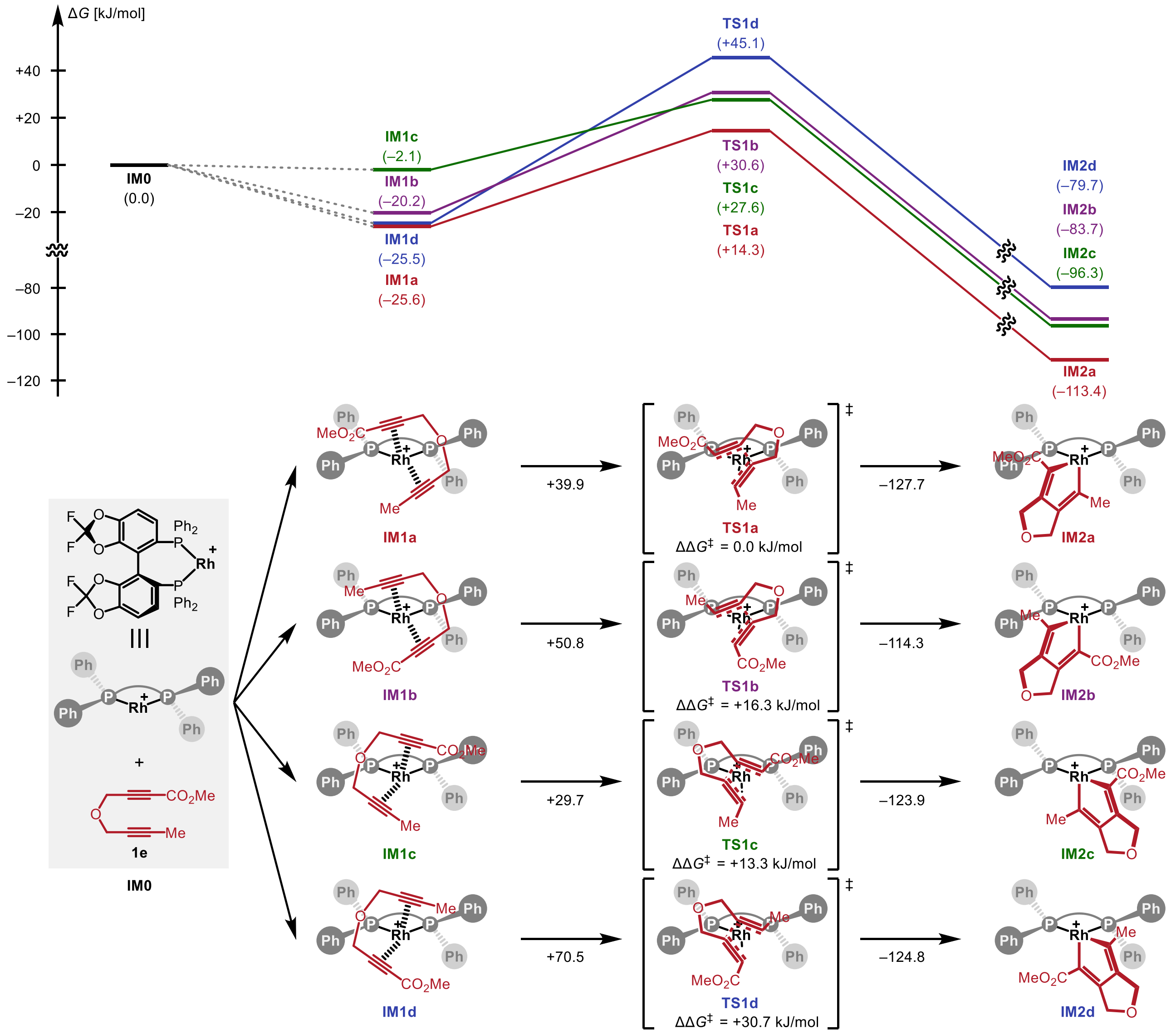

Figure S14. Computed free energy profiles for the oxidative cyclization step (**TS1**). Energy changes are shown in kJ mol^{-1}, and represent the relative free energies calculated at the B3LYP-D3/Def2-TZVPPD/IEFPCM(DCM)// B3LYP-D3/Def2-SVP/IEFPCM(DCM) level of theory.

a) Relative energies and structures of TS1

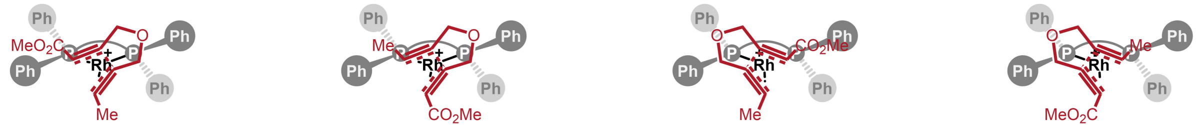

TS1a	TS1b	TS1c	TS1d
$\Delta\Delta G^{\ddagger}$ = 0.0 kJ/mol	$\Delta\Delta G^{\ddagger}$ = +16.3 kJ/mol	$\Delta\Delta G^{\ddagger}$ = +13.3 kJ/mol	$\Delta\Delta G^{\ddagger}$ = +30.7 kJ/mol

b) Distortion-interaction analysis (kJ/mol)

	TS1a	TS1b	TS1c	TS1d
**ΔE_{int}**	**–355.0**	**–410.3**	**–376.2**	**–433.7**
ΔE_{dis_diyne}	216.6	297.4	239.7	300.3
ΔE_{dis_Rh}	73.9	60.6	82.3	97.5

c) Energy decomposition analysis (kJ/mol)

	TS1a	TS1b	TS1c	TS1d
**ΔE_{int}**	**–433.8**	**–503.2**	**–458.6**	**–518.7**
ΔE_{els}	–714.8	–699.3	–744.0	–796.2
ΔE_{xrep}	1453.0	1430.3	1555.2	1659.3
ΔE_{orb}	–923.3	–1007.2	–1023.5	–1143.3
$\Delta E_{DFT\text{-}c}$	–143.9	–139.3	–145.5	–144.9
ΔE_{dc}	–104.8	–87.7	–100.6	–93.6

d) Optimized structures, NCI and IGMH analyses of TS1

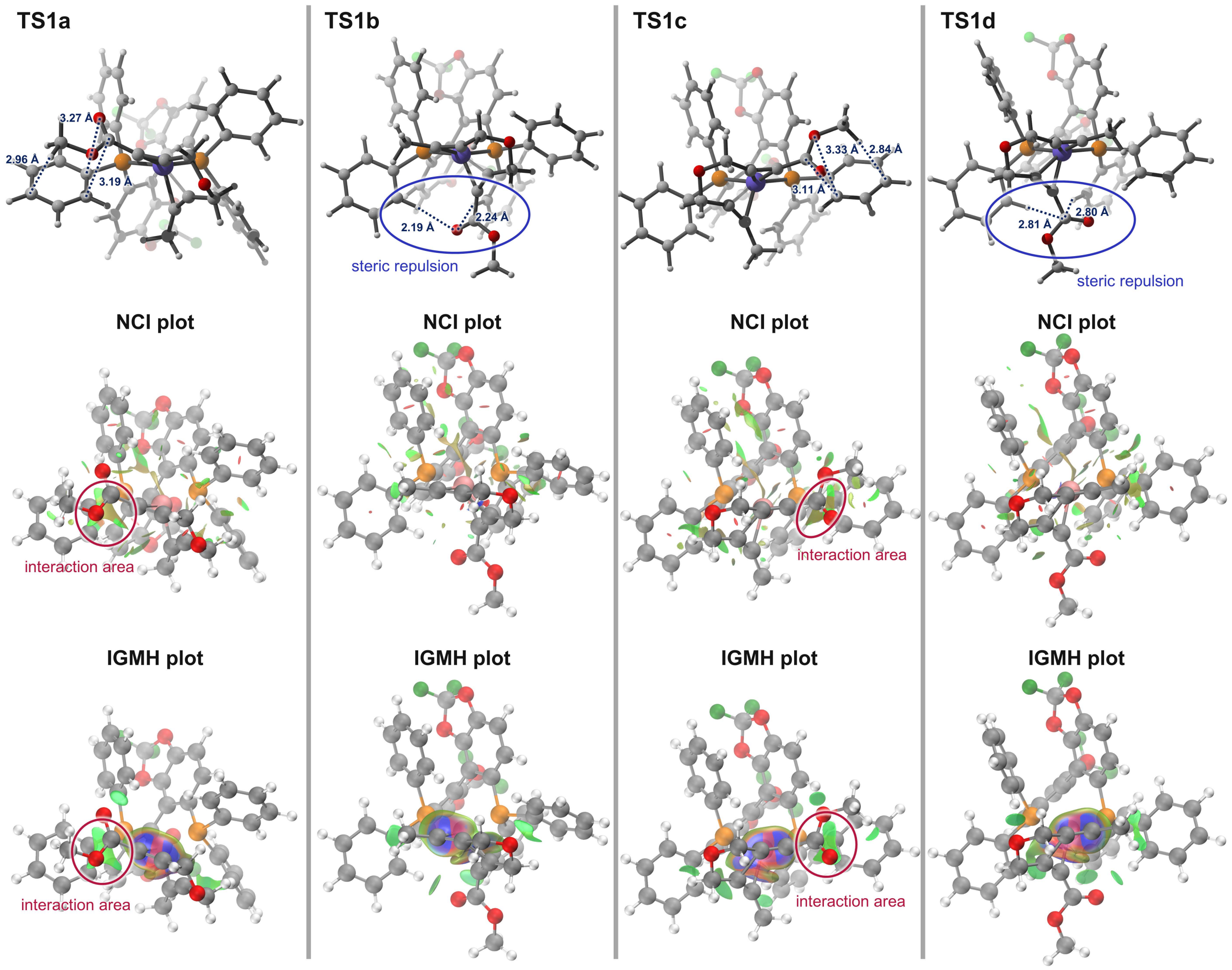

Figure S15. Theoretical mechanistic studies of the transition states for the oxidative cyclization step.

Table S8. Valence angles (θ) and dihedral angles (φ) of diynes at **TS1**.

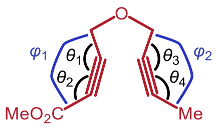	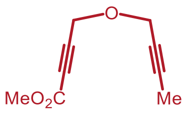 **1e**	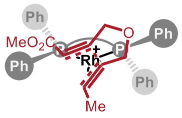 **TS1a**	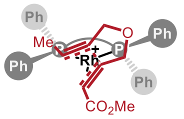 **TS1b**	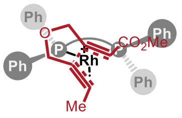 **TS1c**	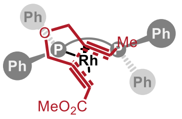 **TS1d**
θ_1	178.0°	150.0°	145.9°	148.9°	150.4°
θ_2	178.3°	145.7°	142.1°	143.3°	145.1°
θ_3	176.8°	149.1°	146.5°	149.9°	149.3°
θ_4	177.0°	149.5°	144.2°	148.1°	143.2°
$\Delta\theta_{\text{total}}$	–	−115.8	−131.3	−119.9	−122.1
φ_1	24.5	4.5	29.9	14.9	35.9
φ_2	8.5	18.6	14.9	40.0	12.1

$\Delta\theta_{\text{total}} = (\theta_1 + \theta_2 + \theta_3 + \theta_4) - (\theta_{1\_1e} + \theta_{2\_1e} + \theta_{3\_1e} + \theta_{4\_1e})$

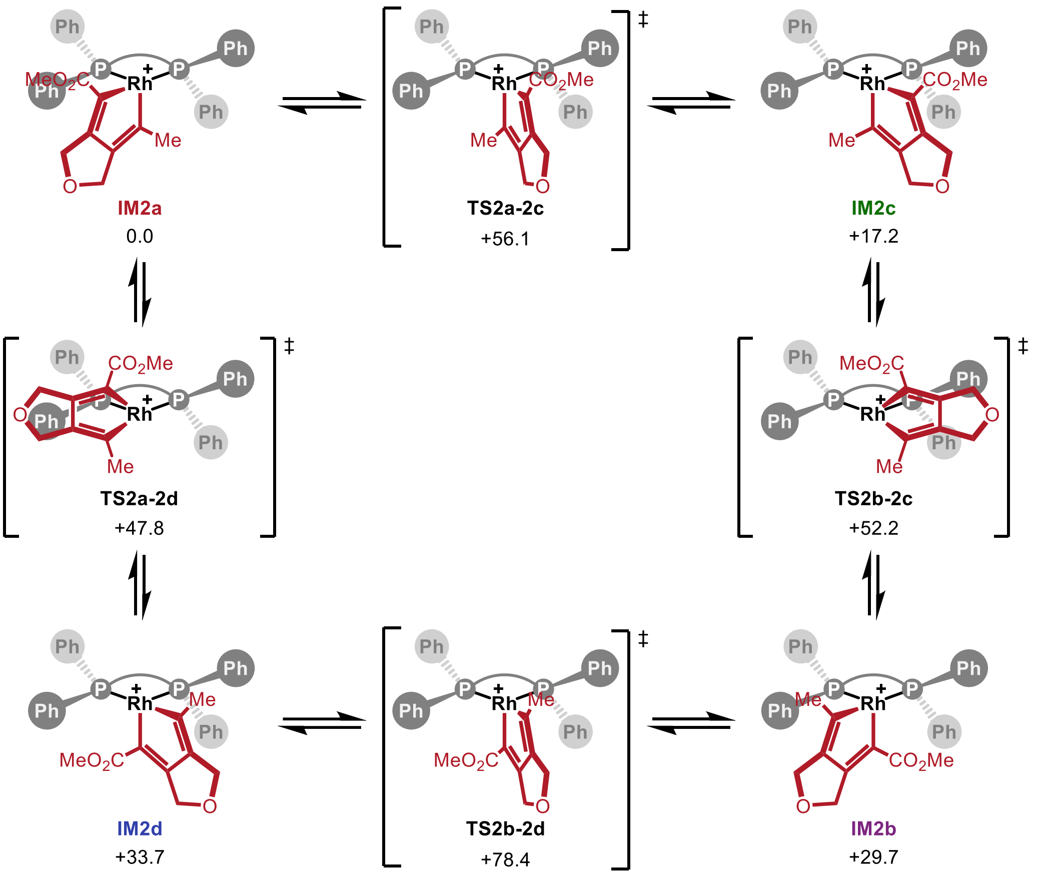

Figure S16. Computed free energy profiles for the interconversion of **IM2**. Energy changes are shown in kJ mol^{-1}, and represent the relative free energies calculated at the B3LYP-D3/Def2-TZVPPD/IEFPCM(DCM)// B3LYP-D3/Def2-SVP/IEFPCM(DCM) level of theory.

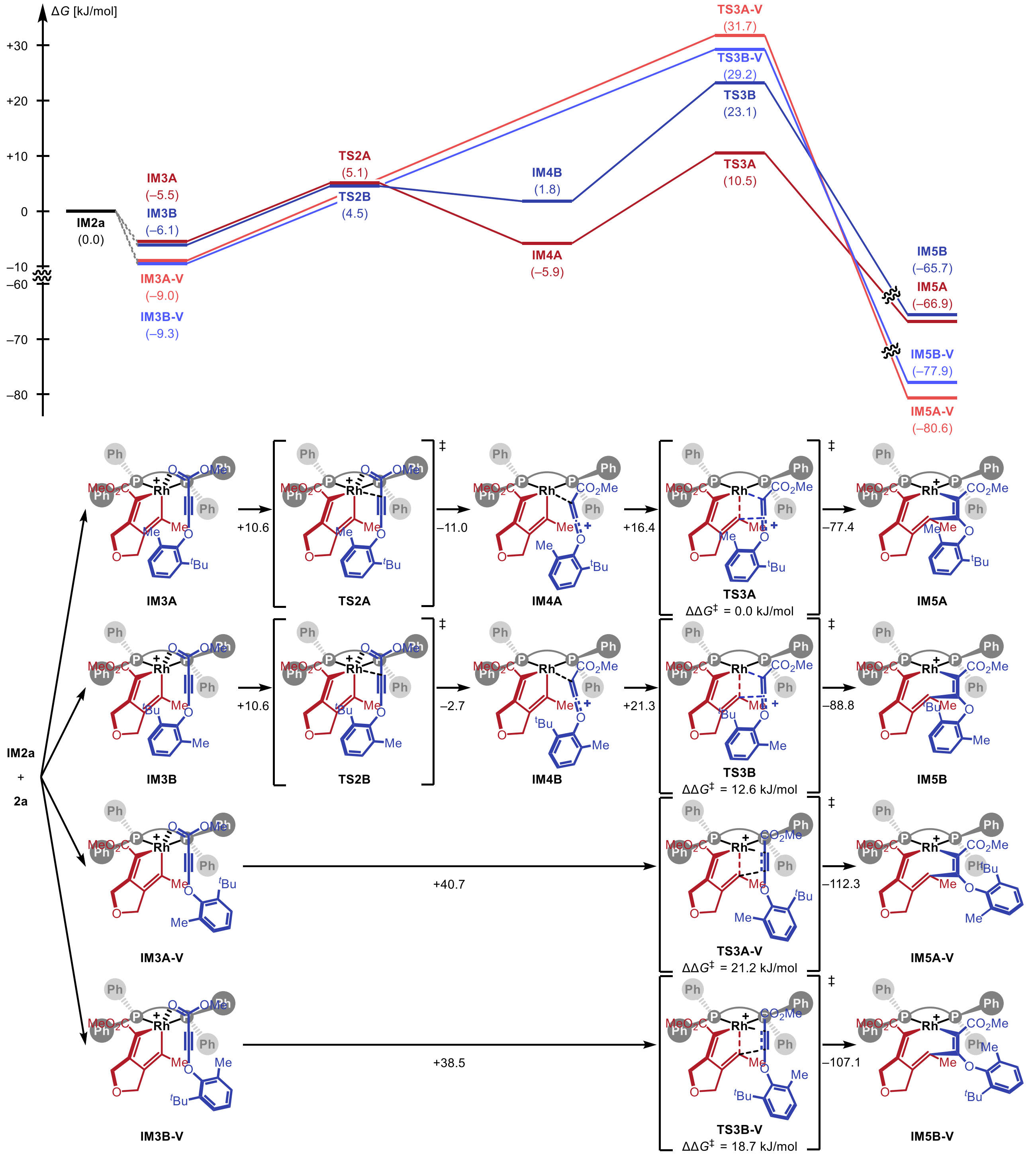

Figure S17. Computed free energy profiles for the insertion step. Energy changes are shown in kJ mol^{-1}, and represent the relative free energies calculated at the B3LYP-D3/Def2-TZVPPD/IEFPCM(DCM)// B3LYP-D3/Def2-SVP/IEFPCM(DCM) level of theory.

a) Relative energies and structures of TS3

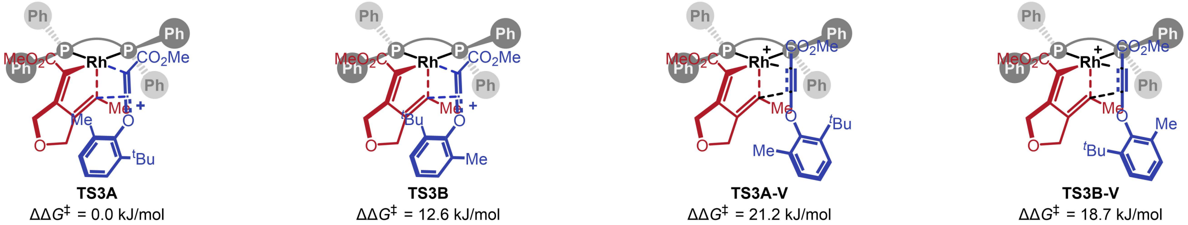

b) Distortion-interaction analysis (kJ/mol)

	TS3A	TS3B	TS3A-V	TS3B-V
ΔE_{int}	**–227.4**	**–216.9**	**–163.4**	**–173.3**
ΔE_{dis_alkyne}	110.9	112.5	88.2	88.3
ΔE_{dis_Rh}	41.0	40.5	31.1	34.7

c) Energy decomposition analysis (kJ/mol)

	TS3A	TS3B	TS3A-V	TS3B-V
ΔE_{int}	**–260.9**	**–252.5**	**–201.2**	**–208.7**
ΔE_{els}	–386.5	–378.6	–286.4	–326.2
ΔE_{xrep}	830.2	821.8	639.9	707.8
ΔE_{orb}	–451.7	–434.8	–332.4	–365.3
$\Delta E_{DFT\text{-}c}$	–121.3	–125.0	–106.0	–109.1
ΔE_{dc}	–131.4	–135.9	–116.3	–115.9

d) Optimized structures, NCI and IGMH analyses of TS3

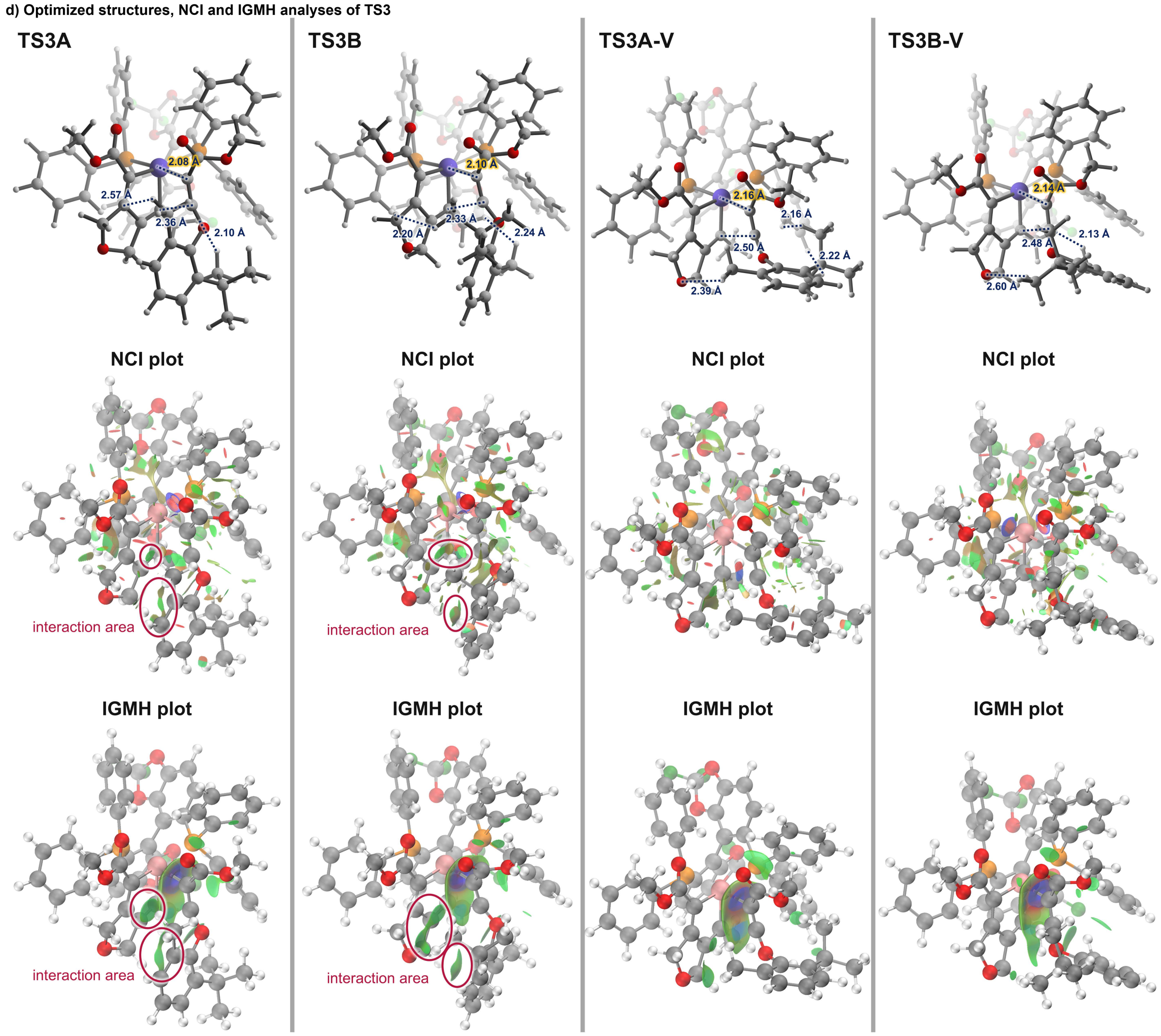

Figure S18. Theoretical mechanistic studies of the transition states for the insertion step.

Table S9. Valence angles, bond lengths and charges from **IM3A** to **IM5A**.

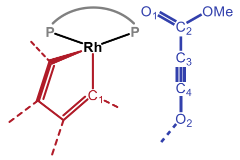

		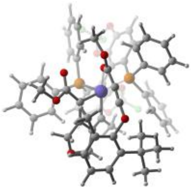 **IM3A**	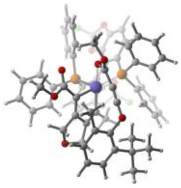 **TS2A**	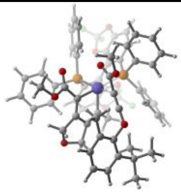 **IM4A**	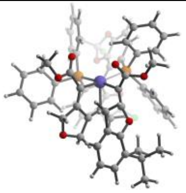 **TS3A**	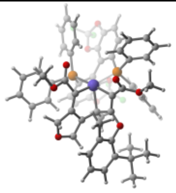 **IM5A**
∠$C_2C_3C_4$		173.7°	160.5°	139.9°	123.6°	130.9°
∠$C_3C_4O_2$		176.3°	176.4°	173.8°	158.1°	128.3°
Rh–O_1 [Å]		2.23	2.25	2.54	3.51	3.61
Rh–C_3 [Å]		3.42	2.86	2.25	2.08	2.03
C_1–C_4 [Å]		4.81	4.18	3.37	2.36	1.54
C_2–C_3 [Å]		1.43	1.44	1.46	1.49	1.47
C_3–C_4 [Å]		1.22	1.22	1.25	1.28	1.33
C_4–O_2 [Å]		1.28	1.27	1.25	1.27	1.37
NPA charge	Rh	0.031	−0.040	−0.188	−0.145	−0.054
	C_2	0.759	0.759	0.768	0.763	0.736
	C_3	−0.280	−0.312	−0.372	−0.360	−0.233
	C_4	0.469	0.519	0.627	0.604	0.296
	O_2	−0.451	−0.438	−0.404	−0.434	−0.496
Hirshfeld charge	Rh	0.664	0.783	0.804	0.908	0.790
	C_2	0.565	0.555	0.526	0.490	0.468
	C_3	−0.064	−0.081	−0.114	−0.483	−0.260
	C_4	0.210	0.230	0.311	0.734	0.115
	O_2	−0.331	−0.340	−0.368	−0.329	−0.333

Table S10. Valence angles, bond lengths and charges from **IM3B** to **IM5B**.

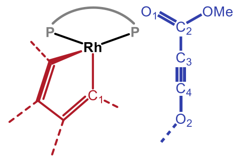

		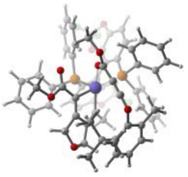 **IM3B**	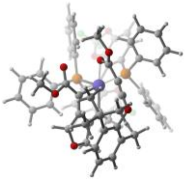 **TS2B**	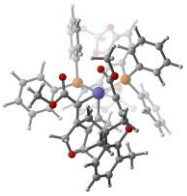 **IM4B**	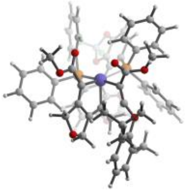 **TS3B**	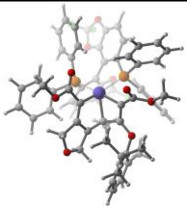 **IM5B**
∠$C_2C_3C_4$		175.2°	153.3	142.9	126.3	129.9
∠$C_3C_4O_2$		178.3°	176.2	174.2	155.6	126.1
Rh–O_1 [Å]		2.23	2.23	2.44	3.60	3.62
Rh–C_3 [Å]		3.50	2.85	2.30	2.10	2.03
C_1–C_4 [Å]		4.98	4.29	3.56	2.33	1.54
C_2–C_3 [Å]		1.42	1.44	1.46	1.49	1.47
C_3–C_4 [Å]		1.22	1.23	1.25	1.28	1.34
C_4–O_2 [Å]		1.28	1.26	1.25	1.27	1.36
NPA charge	Rh	0.042	−0.056	−0.192	−0.168	−0.048
	C_2	0.759	0.761	0.772	0.765	0.737
	C_3	−0.273	−0.325	−0.374	−0.361	−0.252
	C_4	0.466	0.529	0.617	0.600	0.310
	O_2	−0.441	−0.434	−0.408	−0.437	−0.492
Hirshfeld charge	Rh	0.653	0.764	0.811	0.909	0.816
	C_2	0.574	0.553	0.535	0.493	0.469
	C_3	−0.064	−0.095	−0.107	−0.489	−0.265
	C_4	0.210	0.244	0.299	0.74	0.121
	O_2	−0.331	−0.345	−0.366	−0.332	−0.340

5. References

1. B. J. Lee, A. R. Ickes, A. K. Gupta, S. C. Ensign, T. D. Ho, A. Tarasewicz, E. P. Vanable, G. D. Kortman, K. L. Hull, "Synthesis of Unsymmetrical Vicinal Diamines via Directed Hydroamination" *Organic Letters* **24** (2022): 5513–5518, https://doi.org/10.1021/acs.orglett.2c01911.
2. S.-H. Kim-Lee, I. Alonso, P. Mauleón, R. G. Arrayás, J. C. Carretero, "Rationalizing the Role of NaO^{t}Bu in Copper-Catalyzed Carboboration of Alkynes: Assembly of Allylic All-Carbon Quaternary Stereocenters" *ACS Catalysis* **8** (2018): 8993–9005, https://doi.org/10.1021/acscatal.8b02123.
3. B. M. Trost, M. T. Rudd, "A Mechanistic Dichotomy in Ruthenium-Catalyzed Propargyl Alcohol Reactivity: A Novel Hydrative Diyne Cyclization" *Journal of the American Chemical Society* **125** (2003): 11516–11517, https://doi.org/10.1021/ja036410f.
4. J. M. Halford-McGuff, M. Varga, D. B. Cordes, A. P. McKay, A. J. B. Watson, "Modular Synthesis of Complex Benzoxaboraheterocycles through Chelation-Assisted Rh-Catalyzed [2 + 2 + 2] Cycloaddition" *ACS Catalysis* **14** (2024): 1846–1854, https://doi.org/10.1021/acscatal.3c05766.
5. Y. Yamamoto, A. Nagata, H. Nagata, Y. Ando, Y. Arikawa, K. Tatsumi, K. Itoh, "Palladium(0)-Catalyzed Intramolecular [2+2+2] Alkyne Cyclotrimerizations with Electron-Deficient Diynes and Triynes" *Chemistry – A European Journal* **9** (2003): 2469–2483, https://doi.org/10.1002/chem.200204540.
6. D. Yokose, Y. Nagashima, S. Kinoshita, J. Nogami, K. Tanaka, "Enantioselective Synthesis of Axially Chiral Styrene-Carboxylic Esters by Rhodium-Catalyzed Chelation-Controlled [2+2+2] Cycloaddition" *Angewandte Chemie International Edition* **61** (2022): e202202542, https://doi.org/10.1002/anie.202202542.
7. F. Ye, M. Haddad, V. Michelet, V. Ratovelomanana-Vidal, "Solvent-free ruthenium trichloride-mediated [2 + 2 + 2] cycloaddition of α,ω-diynes and cyanamides: a convenient access to 2-aminopyridines" *Organic Chemistry Frontiers* **4** (2017): 1063–1068, https://doi.org/10.1039/c7qo00058h.
8. S. Moulin, H. Dentel, A. Pagnoux-Ozherelyeva, S. Gaillard, A. Poater, L. Cavallo, J. Lohier, J. Renaud, "Bifunctional (Cyclopentadienone)Iron–Tricarbonyl Complexes: Synthesis, Computational Studies and Application in Reductive Amination" *Chemistry – A European Journal* **19** (2013): 17881–17890, https://doi.org/10.1002/chem.201302432.
9. T. Xu, Q. Yang, W. Ye, Q. Jiang, Z. Xu, J. Chen, Z. Yu, "Substituent-Dependent, Iron-Mediated Tandem Cyclization of Diynes with Benzaldehyde Acetals to Form Highly Functionalized Indene Derivatives" *Chemistry – A European Journal* **17** (2011): 10547–10551, https://doi.org/10.1002/chem.201101667.
10. J. Lee, K. H. Kim, O. S. Lee, T.-L. Choi, H.-S. Lee, H. Ihee, J.-H. Sohn, "Preference of Ruthenium-Based Metathesis Catalysts toward *Z*- and *E*-Alkenes as a Guide for Selective Reactions to Alkene Stereoisomers" *The Journal of Organic Chemistry* **81** (2016): 7591–7596, https://doi.org/10.1021/acs.joc.6b01276.
11. Q. Wang, Y. Jiang, R. Sun, X. Tang, M. Shi, "Gold-Catalyzed Fluorination–Hydration: Synthesis of *α*-Fluorobenzofuranones from 2-Alkynylphenol Derivatives" *Chemistry – A European Journal* **22** (2016): 14739–14745, https://doi.org/10.1002/chem.201602545.

12. J. Buter, D. Heijnen, C. Vila, V. Hornillos, E. Otten, M. Giannerini, A. J. Minnaard, B. L. Feringa, "Palladium-Catalyzed, *tert*-Butyllithium-Mediated Dimerization of Aryl Halides and Its Application in the Atropselective Total Synthesis of Mastigophorene A" *Angewandte Chemie International Edition* **55** (2016): 3620–3624, https://doi.org/10.1002/anie.201510328.
13. H. Bao, Y. Chen, X. Yang, "Catalytic Asymmetric Synthesis of Axially Chiral Diaryl Ethers through Enantioselective Desymmetrization" *Angewandte Chemie International Edition* **62** (2023): e202300481, https://doi.org/10.1002/anie.202300481.
14. Y. Liu, L. Yuan, L. Dai, Q. Zhu, G. Zhong, X. Zeng, "Carbene-Catalyzed Atroposelective Construction of Chiral Diaryl Ethers" *The Journal of Organic Chemistry* **89** (2024): 7630–7643, https://doi.org/10.1021/acs.joc.4c00330.
15. H. Egami, T. Ide, Y. Kawato, Y. Hamashima, "Benzylic C–H trifluoromethylation of phenol derivatives" *Chemical Communications* **51** (2015): 16675–16678, https://doi.org/10.1039/c5cc07011b.
16. W. Yang, P. Dai, K. Luo, L. Wu, "Iodide/*tert*-Butyl Hydroperoxide-Mediated Benzylic C–H Sulfonylation and Peroxidation of Phenol Derivatives" *Advanced Synthesis & Catalysis* **358** (2016): 3184–3190, https://doi.org/10.1002/adsc.201600541.
17. K. J. Laidler, *Chemical Kinetics*, 3rd ed.; Pearson Education: Singapore (1987).
18. G. M. Sheldrick, "SHELXT – Integrated space-group and crystal-structure determination" *Acta Crystallographica Section A: Foundations and Advances* **71** (2015): 3–8, https://doi.org/10.1107/s2053273314026370.
19. G. M. Sheldrick, "Crystal structure refinement with SHELXL" *Acta Crystallographica Section C* **71** (2015): 3–8, https://doi.org/10.1107/s2053229614024218.
20. A. D. Becke, "A new mixing of Hartree–Fock and local density-functional theories" *The Journal of Chemical Physics* **98** (1993): 1372–1377, https://doi.org/10.1063/1.464304.
21. C. Lee, W. Yang, R. G. Parr, "Development of the Colle-Salvetti correlation-energy formula into a functional of the electron density" *Physical Review B* **37** (1988): 785–789, https://doi.org/10.1103/physrevb.37.785.
22. M. J. Frisch, G. W. Trucks, H. B. Schlegel, G. E. Scuseria, M. A. Robb, J. R. Cheeseman, G. Scalmani, V. Barone, G. A. Petersson, H. Nakatsuji, X. Li, M. Caricato, A. V. Marenich, J. Bloino, B. G. Janesko, R. Gomperts, B. Mennucci, H. P. Hratchian, J. V. Ortiz, A. F. Izmaylov, J. L. Sonnenberg, D. Williams-Young, F. Ding, F. Lipparini, F. Egidi, J. Goings, B. Peng, A. Petrone, T. Henderson, D. Ranasinghe, V. G. Zakrzewski, J. Gao, N. Rega, G. Zheng, W. Liang, M. Hada, M. Ehara, K. Toyota, R. Fukuda, J. Hasegawa, M. Ishida, T. Nakajima, Y. Honda, O. Kitao, H. Nakai, T. Vreven, K. Throssell, J. A. Montgomery, Jr., J. E. Peralta, F. Ogliaro, M. J. Bearpark, J. J. Heyd, E. N. Brothers, K. N. Kudin, V. N. Staroverov, T. A. Keith, R. Kobayashi, J. Normand, K. Raghavachari, A. P. Rendell, J. C. Burant, S. S. Iyengar, J. Tomasi, M. Cossi, J. M. Millam, M. Klene, C. Adamo, R. Cammi, J. W. Ochterski, R. L. Martin, K. Morokuma, O. Farkas, J. B. Foresman, and D. J. Fox, *Gaussian 16*, Revision C.01, Gaussian, Inc., Wallingford CT (2016).
23. S. Grimme, J. Antony, S. Ehrlich, H. Krieg, "A consistent and accurate ab initio parametrization of density functional dispersion correction (DFT-D) for the 94 elements H-Pu" *The Journal of Chemical Physics* **132** (2010): 154104, https://doi.org/10.1063/1.3382344.

24. S. Grimme, S. Ehrlich, L. Goerigk, "Effect of the damping function in dispersion corrected density functional theory" *Journal of Computational Chemistry* **32** (2011): 1456–1465, https://doi.org/10.1002/jcc.21759.
25. F. Weigend, R. Ahlrichs, "Balanced basis sets of split valence, triple zeta valence and quadruple zeta valence quality for H to Rn: Design and assessment of accuracy" *Physical Chemistry Chemical Physics* **7** (2005): 3297–3305, https://doi.org/10.1039/b508541a.
26. F. Weigend, "Accurate Coulomb-fitting basis sets for H to Rn" *Physical Chemistry Chemical Physics* **8** (2006): 1057–1065, https://doi.org/10.1039/b515623h.
27. J. Tomasi, B. Mennucci, R. Cammi, "Quantum Mechanical Continuum Solvation Models" *Chemical Reviews* **105** (2005): 2999–3094, https://doi.org/10.1021/cr9904009.
28. G. Scalmani, M. J. Frisch, "Continuous surface charge polarizable continuum models of solvation. I. General formalism" *The Journal of Chemical Physics* **132** (2010): 114110, https://doi.org/10.1063/1.3359469.
29. S. Maeda, K. Ohno, K. Morokuma, "Systematic exploration of the mechanism of chemical reactions: the global reaction route mapping (GRRM) strategy using the ADDF and AFIR methods" *Physical Chemistry Chemical Physics* **15** (2013): 3683–3701, https://doi.org/10.1039/c3cp44063j.
30. S. Maeda, Y. Harabuchi, M. Takagi, T. Taketsugu, K. Morokuma, "Artificial Force Induced Reaction (AFIR) Method for Exploring Quantum Chemical Potential Energy Surfaces" *The Chemical Record* **16** (2016): 2232–2248, https://doi.org/10.1002/tcr.201600043.
31. S. Maeda, Y. Harabuchi, M. Takagi, K. Saita, K. Suzuki, T. Ichino, Y. Sumiya, K. Sugiyama, Y. Ono, "Implementation and performance of the artificial force induced reaction method in the GRRM17 program" *Journal of Computational Chemistry* **39** (2018): 233–251, https://doi.org/10.1002/jcc.25106.
32. S. Maeda, Y. Harabuchi, "Exploring paths of chemical transformations in molecular and periodic systems: An approach utilizing force" *Wiley Interdisciplinary Reviews: Computational Molecular Science* **11** (2021), https://doi.org/10.1002/wcms.1538.
33. S. Grimme, C. Bannwarth, P. Shushkov, "A Robust and Accurate Tight-Binding Quantum Chemical Method for Structures, Vibrational Frequencies, and Noncovalent Interactions of Large Molecular Systems Parametrized for All spd-Block Elements (Z = 1–86)" *Journal of Chemical Theory and Computation* **13** (2017): 1989–2009, https://doi.org/10.1021/acs.jctc.7b00118.
34. F. Neese, "Software update: the ORCA program system, version 4.0" *Wiley Interdisciplinary Reviews: Computational Molecular Science* **8** (2018), https://doi.org/10.1002/wcms.1327.
35. K. Fukui, "The path of chemical reactions - the IRC approach" *Accounts of Chemical Research* **14** (1981): 363–368, https://doi.org/10.1021/ar00072a001.
36. C. Y. Legault, *CYLview*, version 1.0b; Université de Sherbrooke (2009).
37. E. R. Johnson, S. Keinan, P. Mori-Sánchez, J. Contreras-García, A. J. Cohen, W. Yang, "Revealing Noncovalent Interactions" *J. Am. Chem. Soc.* **132** (2010); 6498–6506, https://doi.org/10.1021/ja100936w.
38. T. Lu, Q. Chen, "Independent gradient model based on Hirshfeld partition: A new method for visual study of interactions in chemical systems" *Journal of Computational Chemistry* **43** (2022): 539–555, https://doi.org/10.1002/jcc.26812.

39. T. Lu, Q. Chen, "Erratum to 'Independent gradient model based on Hirshfeld partition: A new method for visual study of interactions in chemical systems'" *ChemRxiv* (2022), https://doi.org/10.26434/chemrxiv-2022-g1m34.
40. T. Lu, F. Chen, "Multiwfn: A multifunctional wavefunction analyzer" *Journal of Computational Chemistry* **33** (2012): 580–592, https://doi.org/10.1002/jcc.22885.
41. T. Lu, "A comprehensive electron wavefunction analysis toolbox for chemists, Multiwfn" *The Journal of Chemical Physics* **161** (2024): 082503, https://doi.org/10.1063/5.0216272.
42. W. Humphrey, A. Dalke, K. Schulten, "VMD: Visual molecular dynamics" *Journal of Molecular Graphics* **14** (1996): 33–38, https://doi.org/10.1016/0263-7855(96)00018-5.
43. T. Lu, Q. Chen, "Simple, Efficient, and Universal Energy Decomposition Analysis Method Based on Dispersion-Corrected Density Functional Theory" *The Journal of Physical Chemistry A* **127** (2023): 7023–7035, https://doi.org/10.1021/acs.jpca.3c04374.

6. ^{1}H, ^{13}C, and ^{19}F NMR Spectra

Methyl 4-((4-methyl-*N*-(pent-2-yn-1-yl)phenyl)sulfonamido)but-2-ynoat (1b)

^{1}H NMR ($CDCl_3$, 400 MHz)

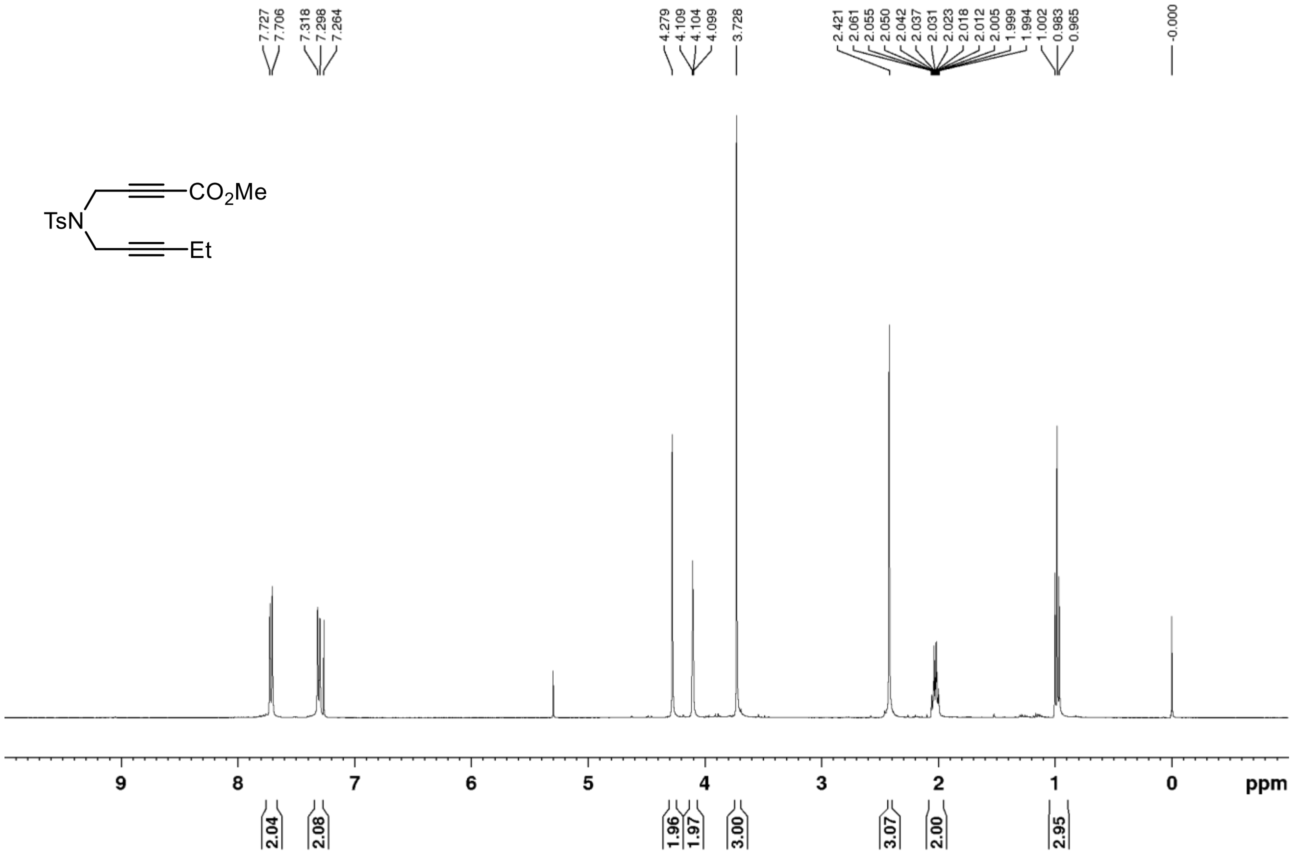

^{13}C NMR ($CDCl_3$, 101 MHz)

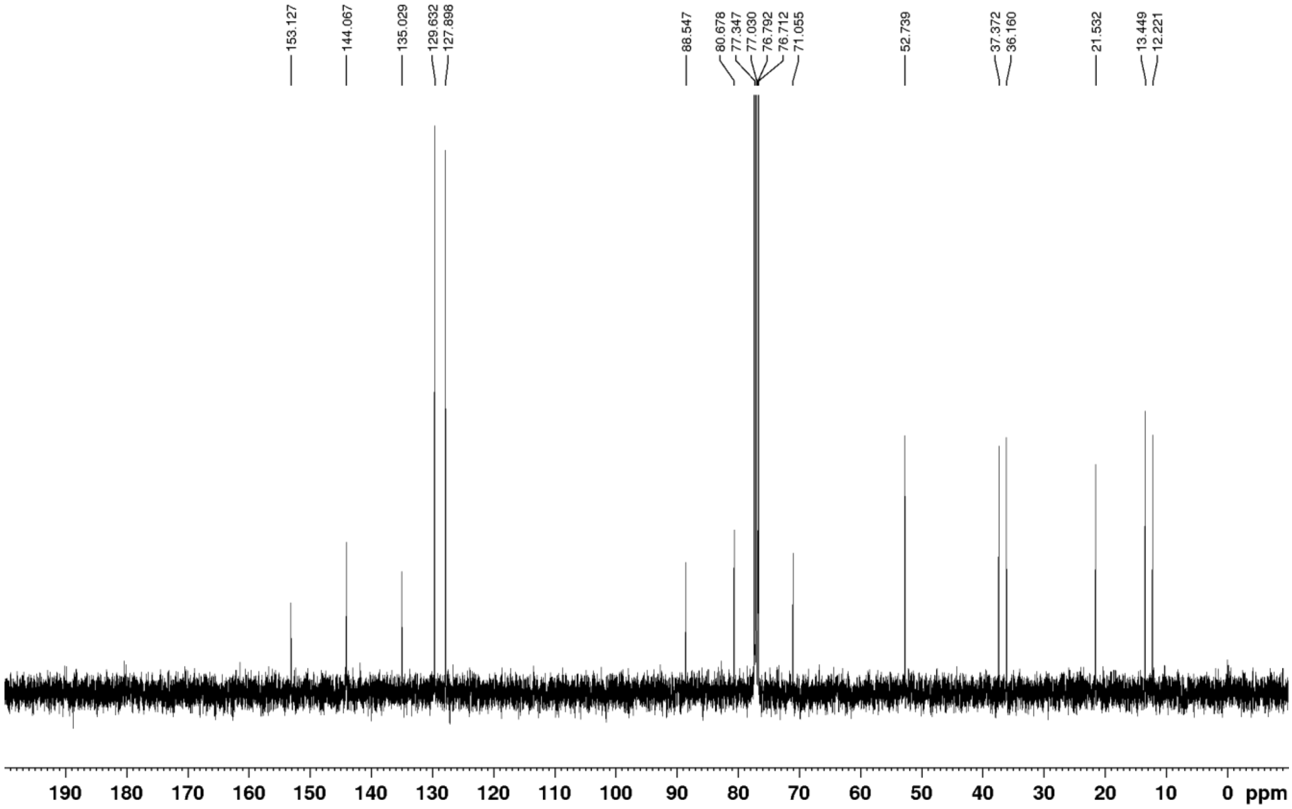

Ethyl 4-((*N*-(but-2-yn-1-yl)-4-methylphenyl)sulfonamido)but-2-ynoate (1c)

^{1}H NMR ($CDCl_3$, 400 MHz)

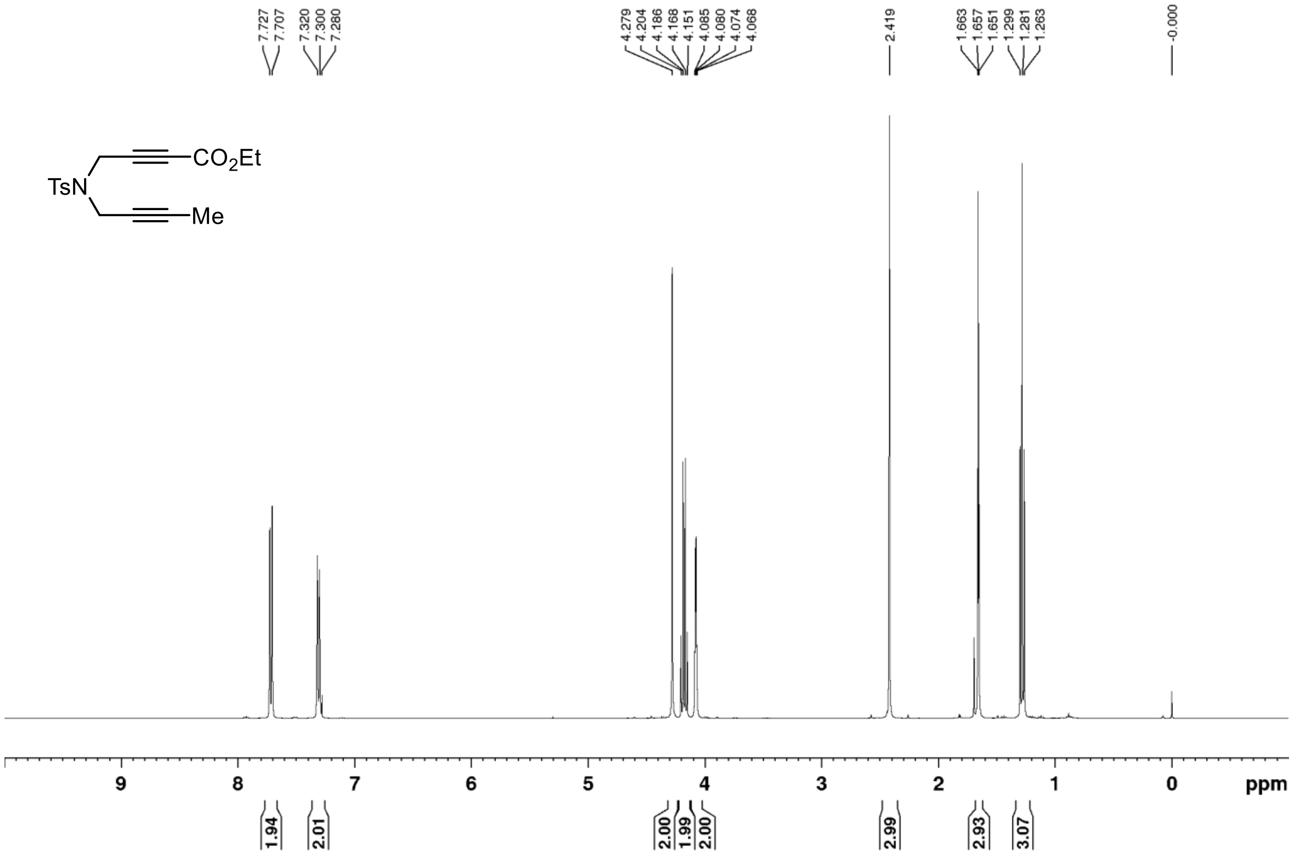

^{13}C NMR ($CDCl_3$, 101 MHz)

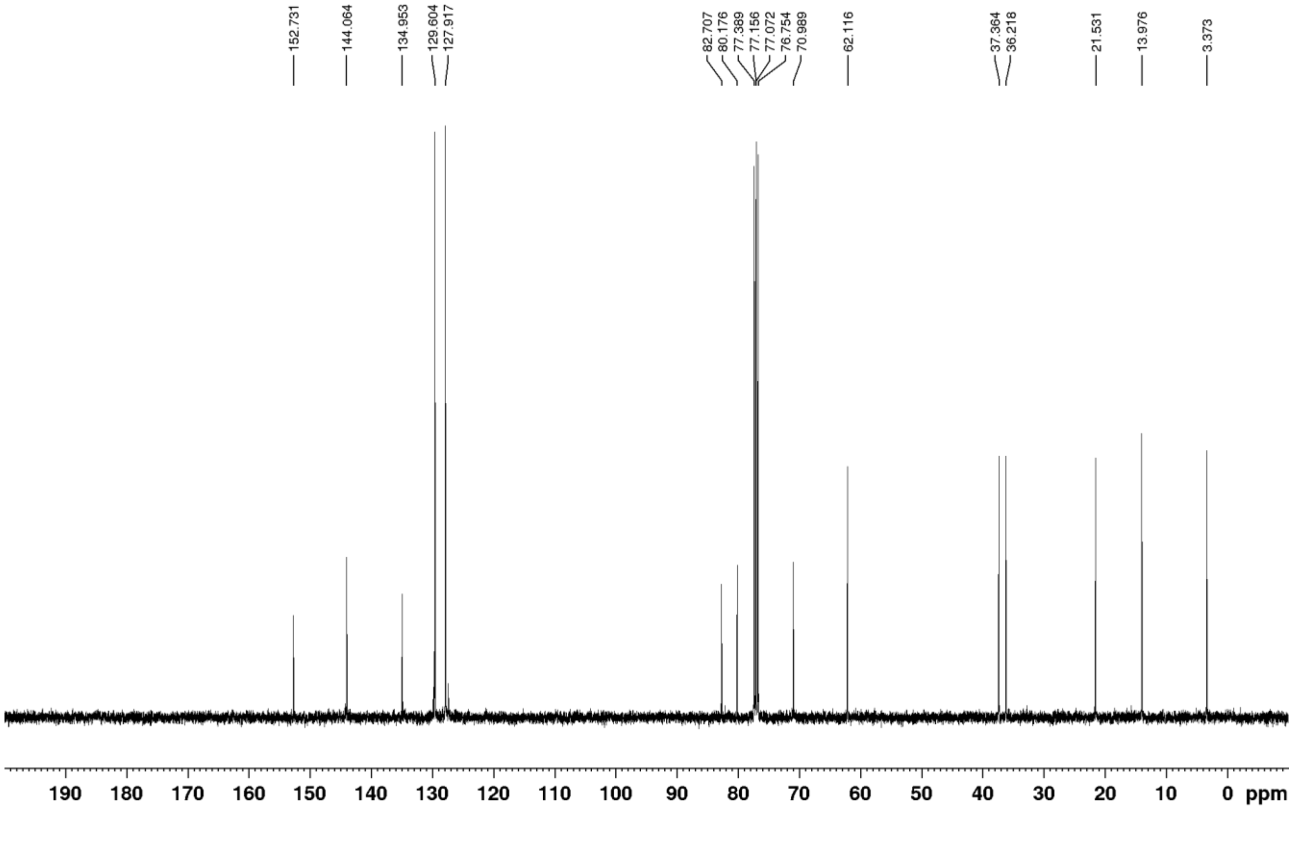

N-(But-2-yn-1-yl)-4-methyl-N-(4-oxopent-2-yn-1-yl)benzenesulfonamide (1d)

^{1}H NMR ($CDCl_3$, 400 MHz)

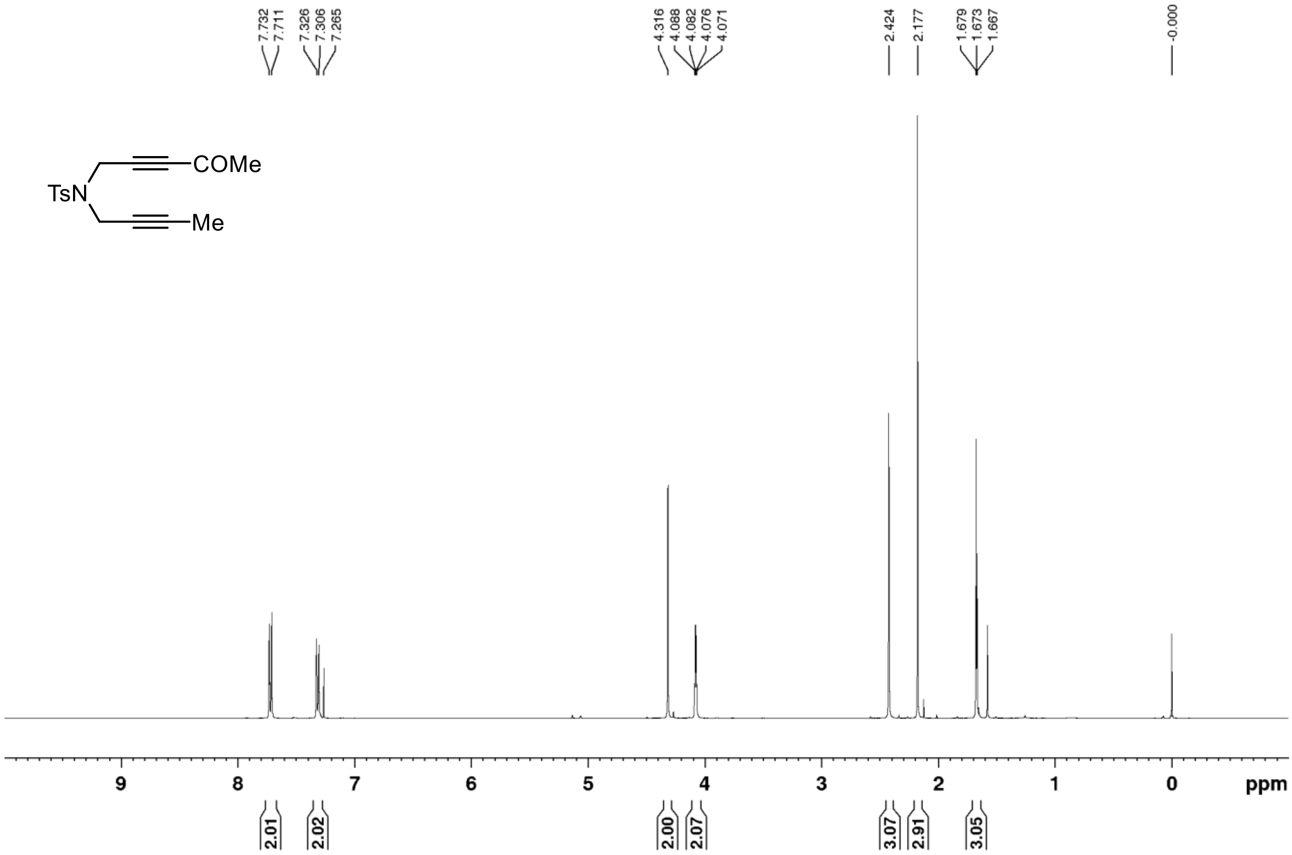

^{13}C NMR ($CDCl_3$, 101 MHz)

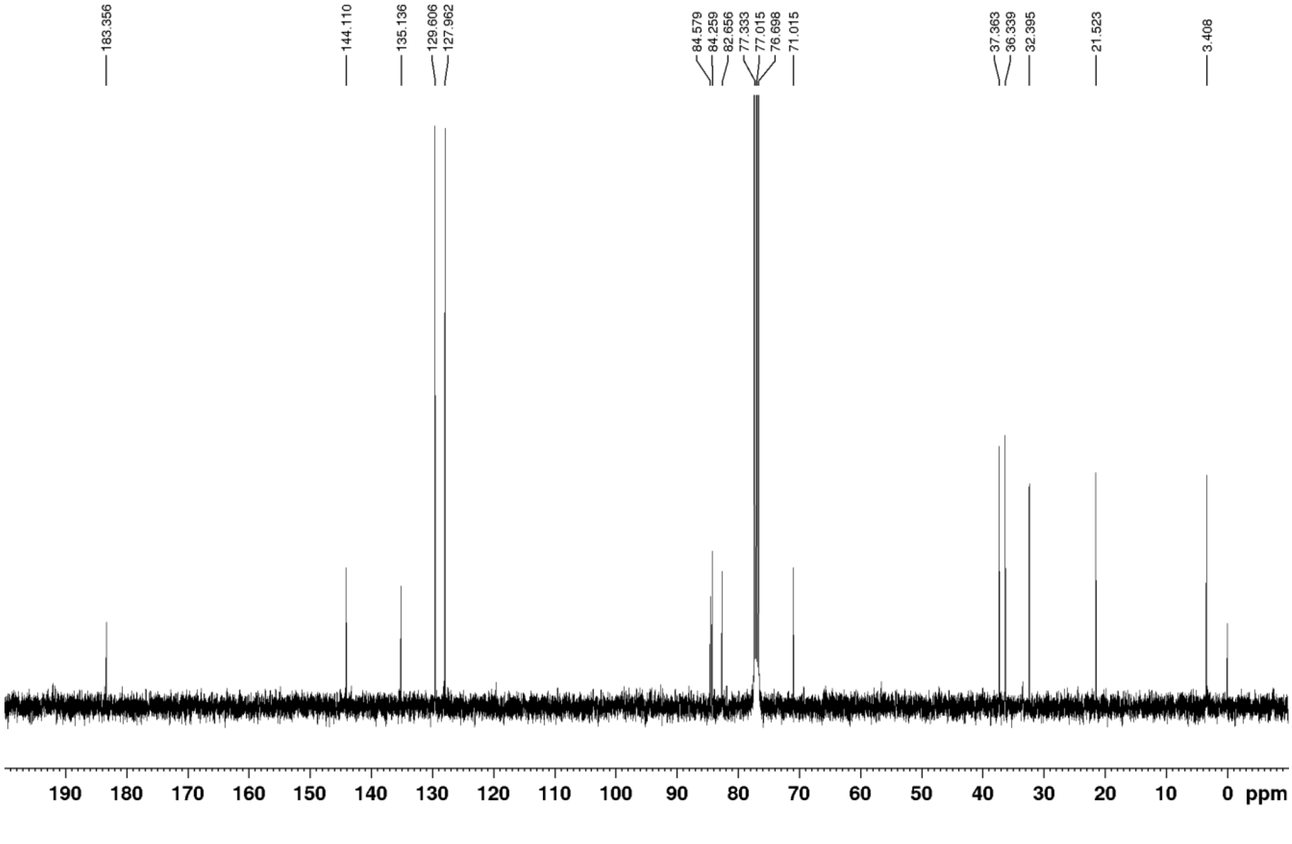

***N*-(3-(3,5-Bis(trifluoromethyl)phenyl)prop-2-yn-1-yl)-*N*-(but-2-yn-1-yl)-4-methylbenzenesulfonamide (1g)**

^{1}H NMR ($CDCl_3$, 400 MHz)

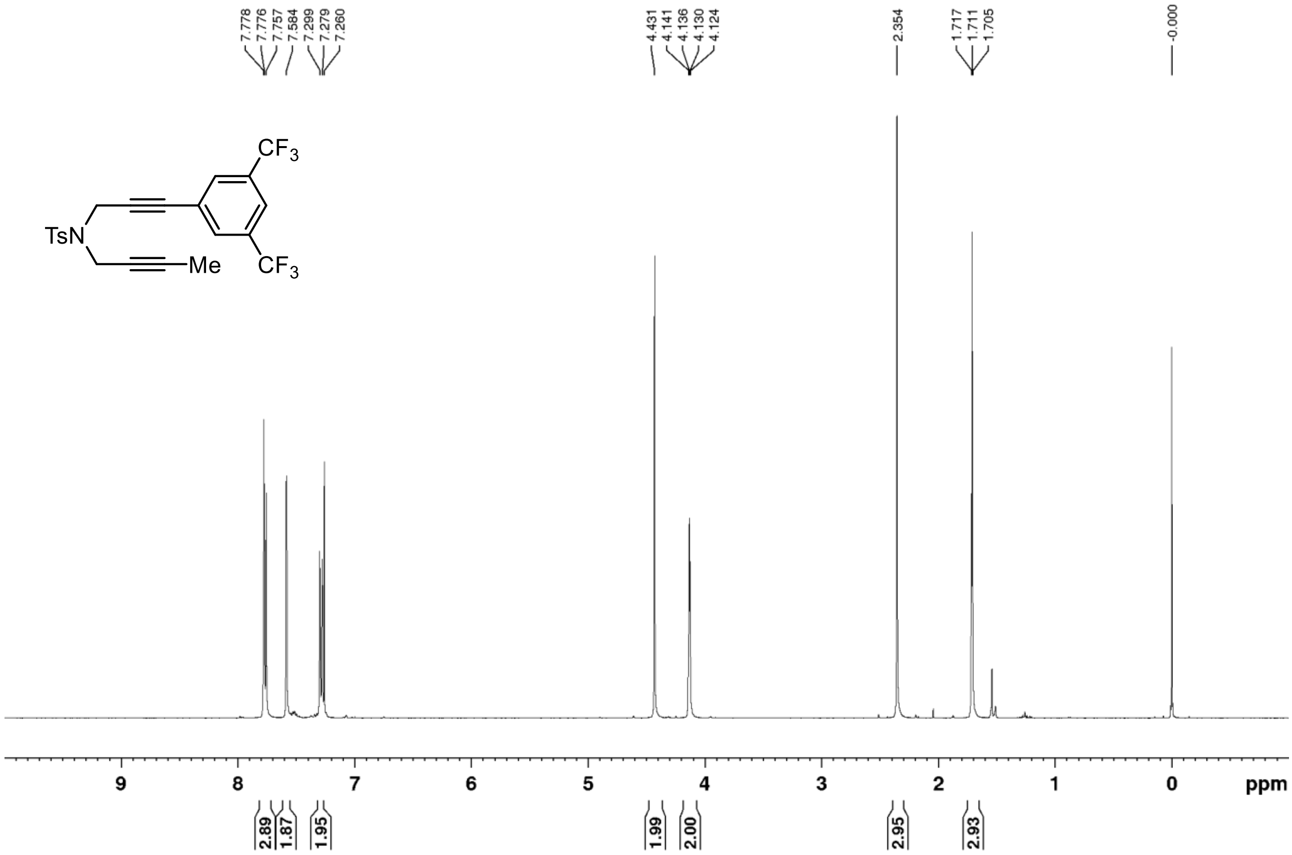

^{13}C NMR ($CDCl_3$, 101 MHz)

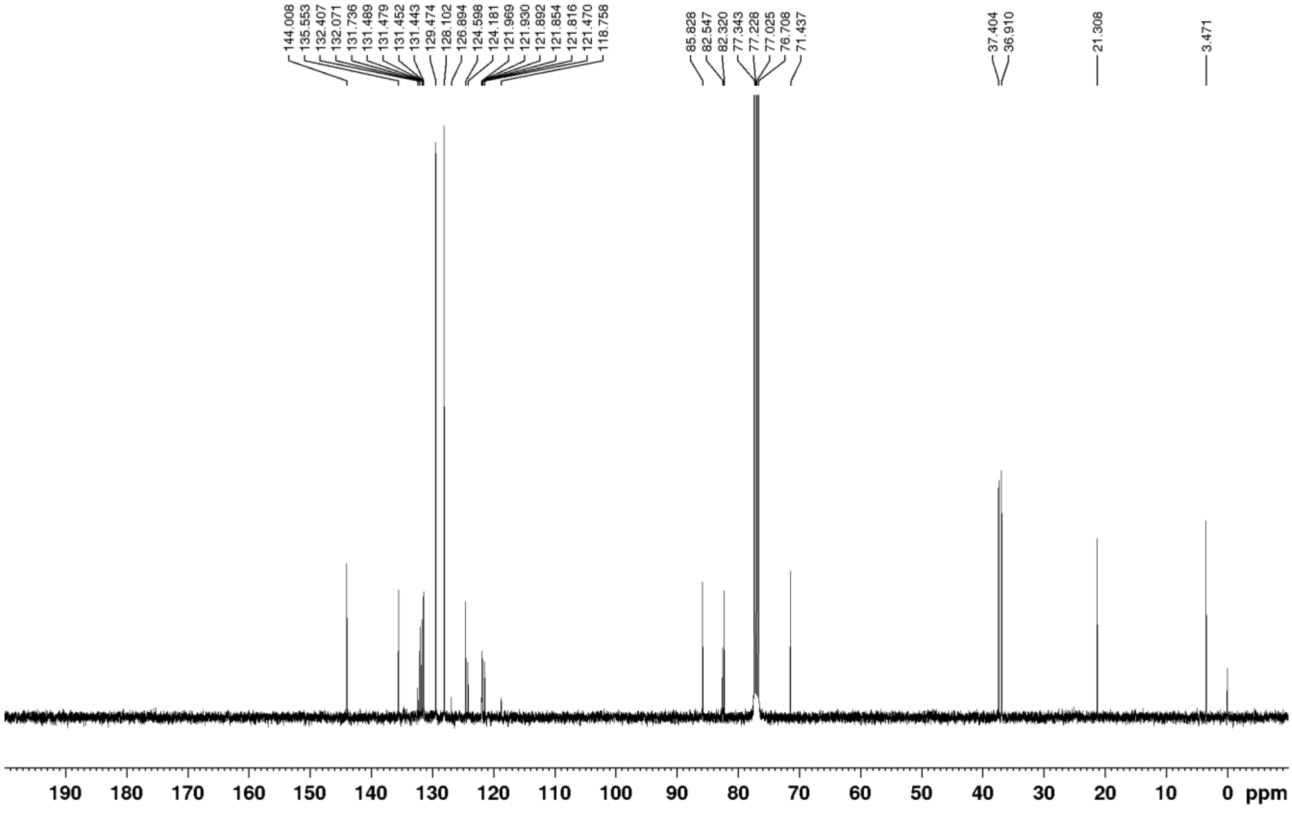

^{19}F NMR ($CDCl_3$, 377 MHz)

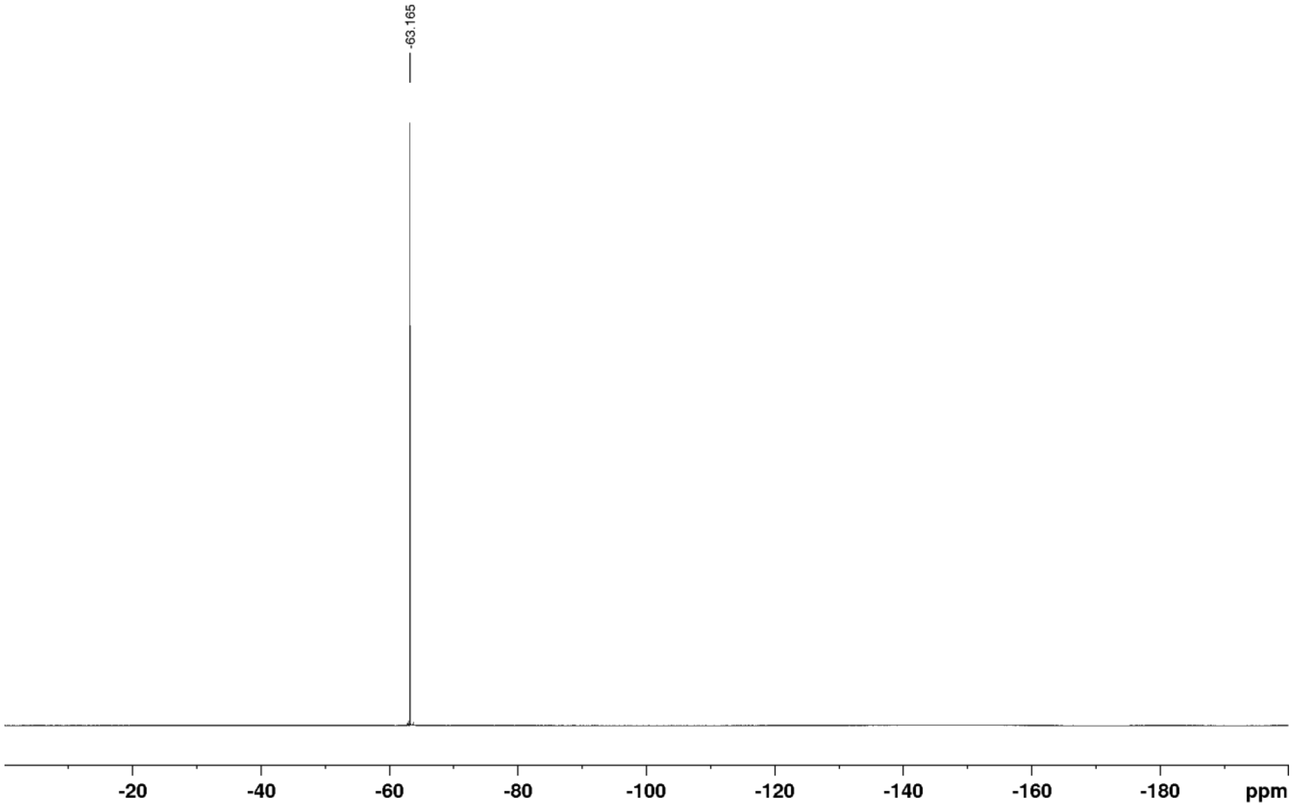

4-Methyl-*N*,*N*-bis(3-(thiophen-3-yl)prop-2-yn-1-yl)benzenesulfonamide (1k)

^{1}H NMR ($CDCl_3$, 400 MHz)

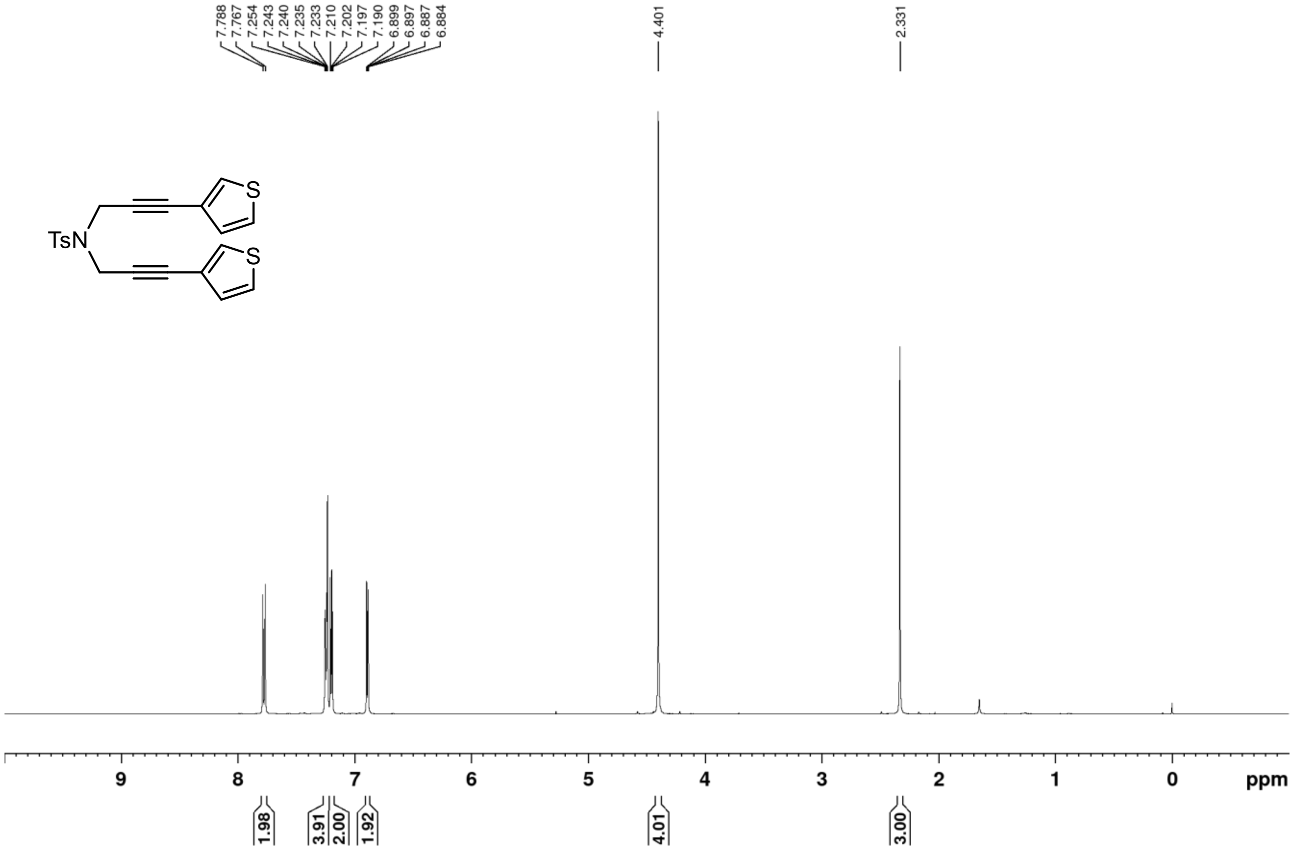

^{13}C NMR ($CDCl_3$, 101 MHz)

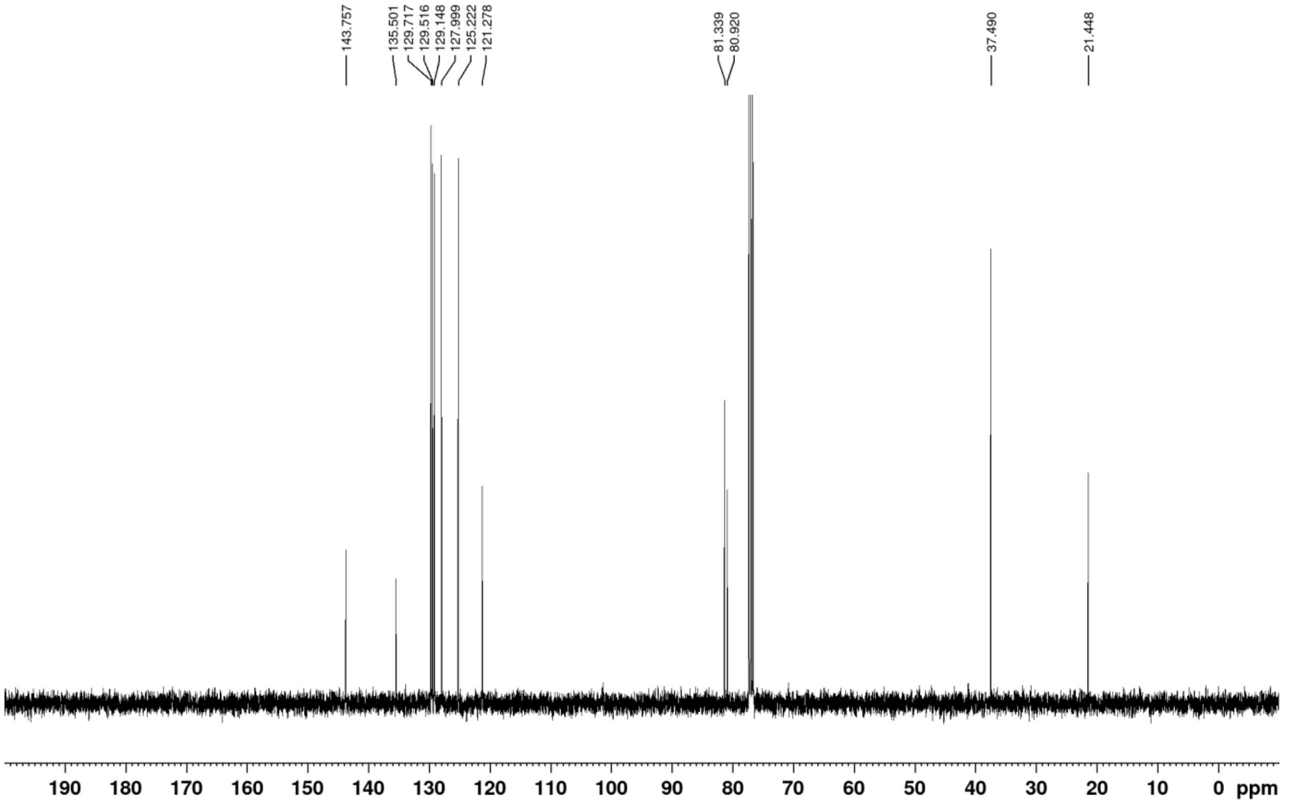

Dimethyl 4,4'-(tosylazanediyl)bis(but-2-ynoate) (1l)

^{1}H NMR ($CDCl_3$, 400 MHz)

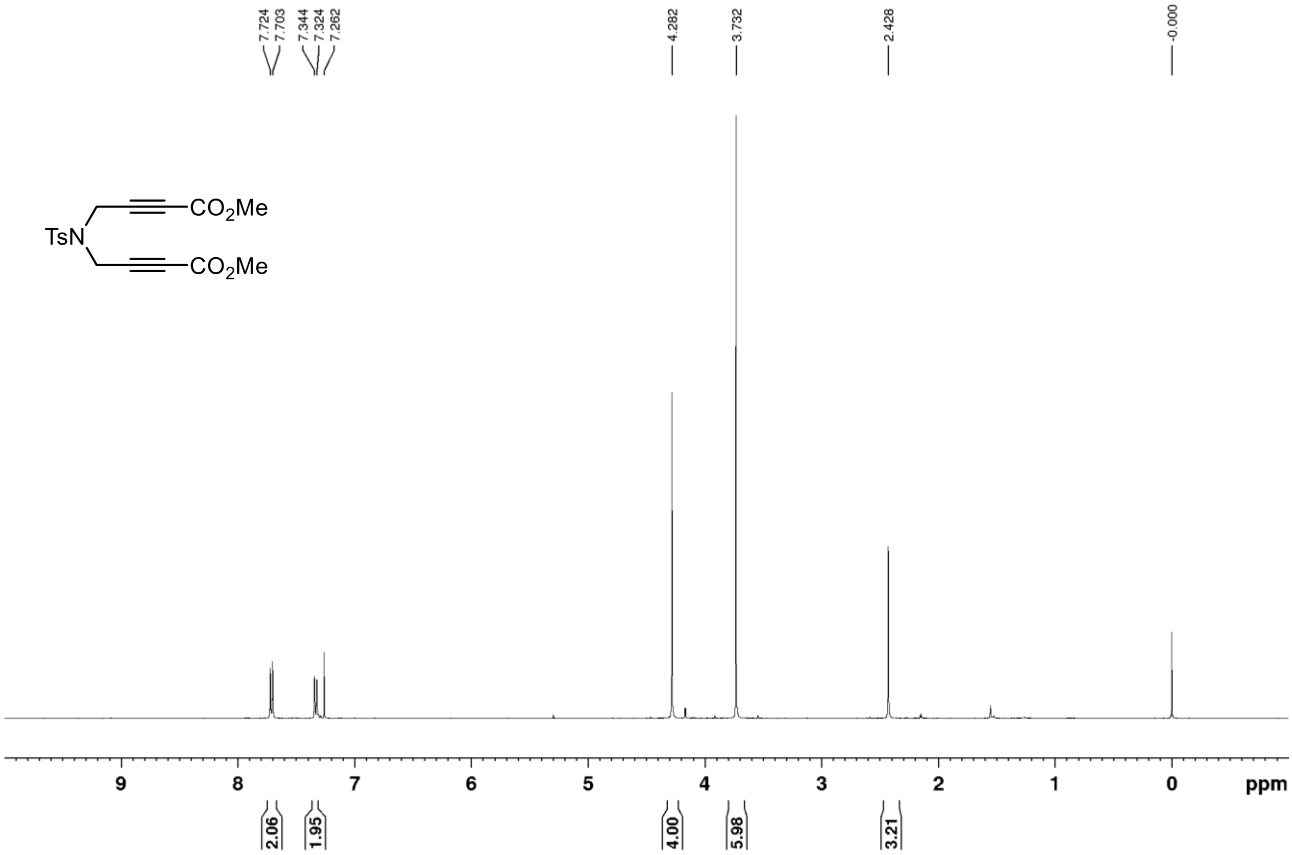

^{13}C NMR ($CDCl_3$, 101 MHz)

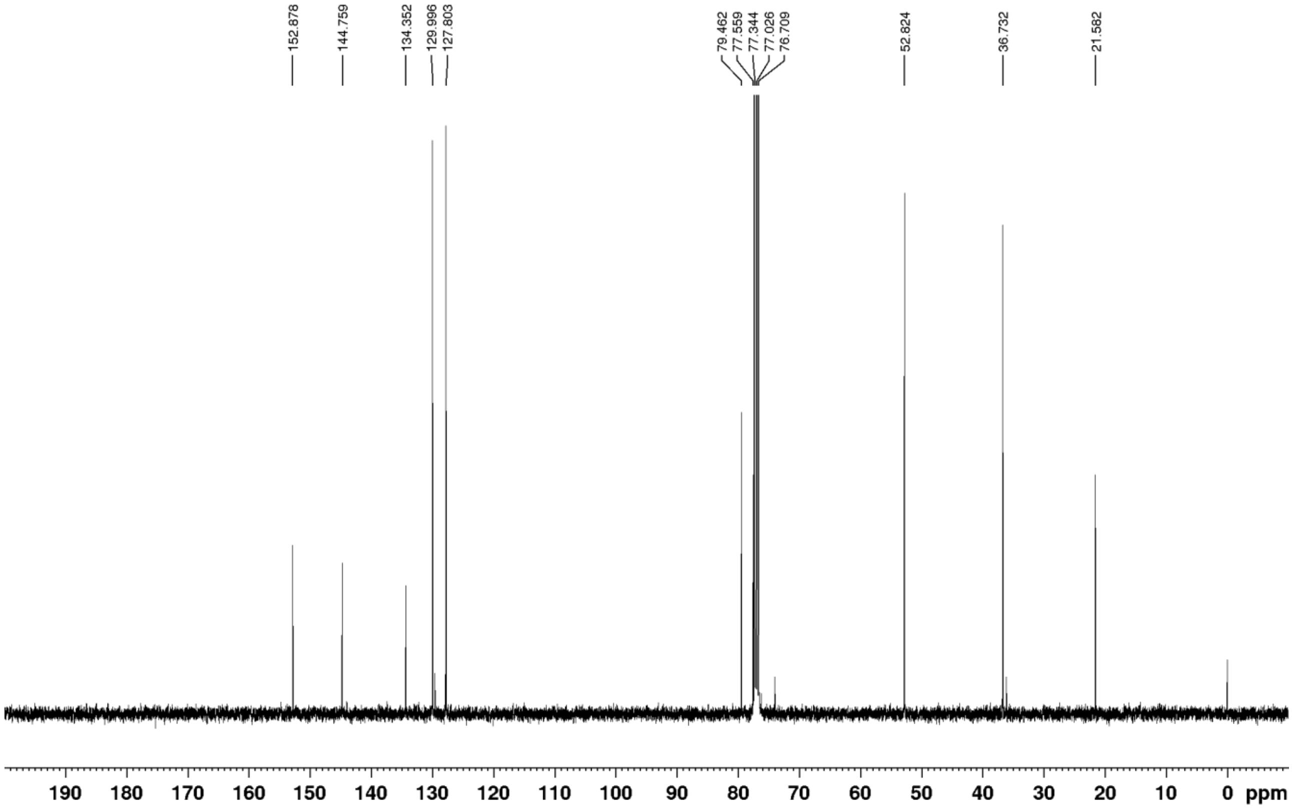

N-(But-2-yn-1-yl)-4-methyl-N-(3-(4-nitrophenyl)prop-2-yn-1-yl)benzenesulfonamide (1m)

^{1}H NMR ($CDCl_3$, 400 MHz)

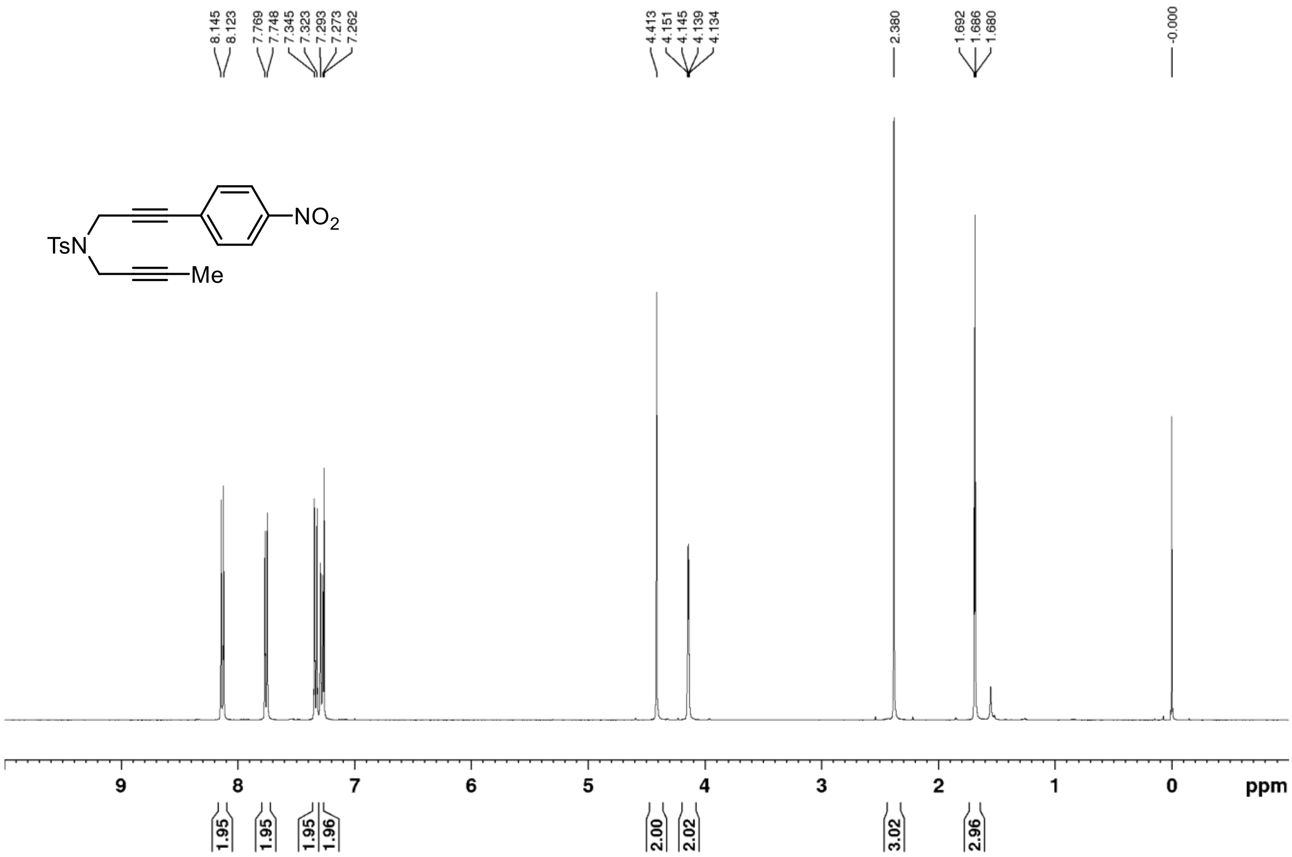

^{13}C NMR ($CDCl_3$, 101 MHz)

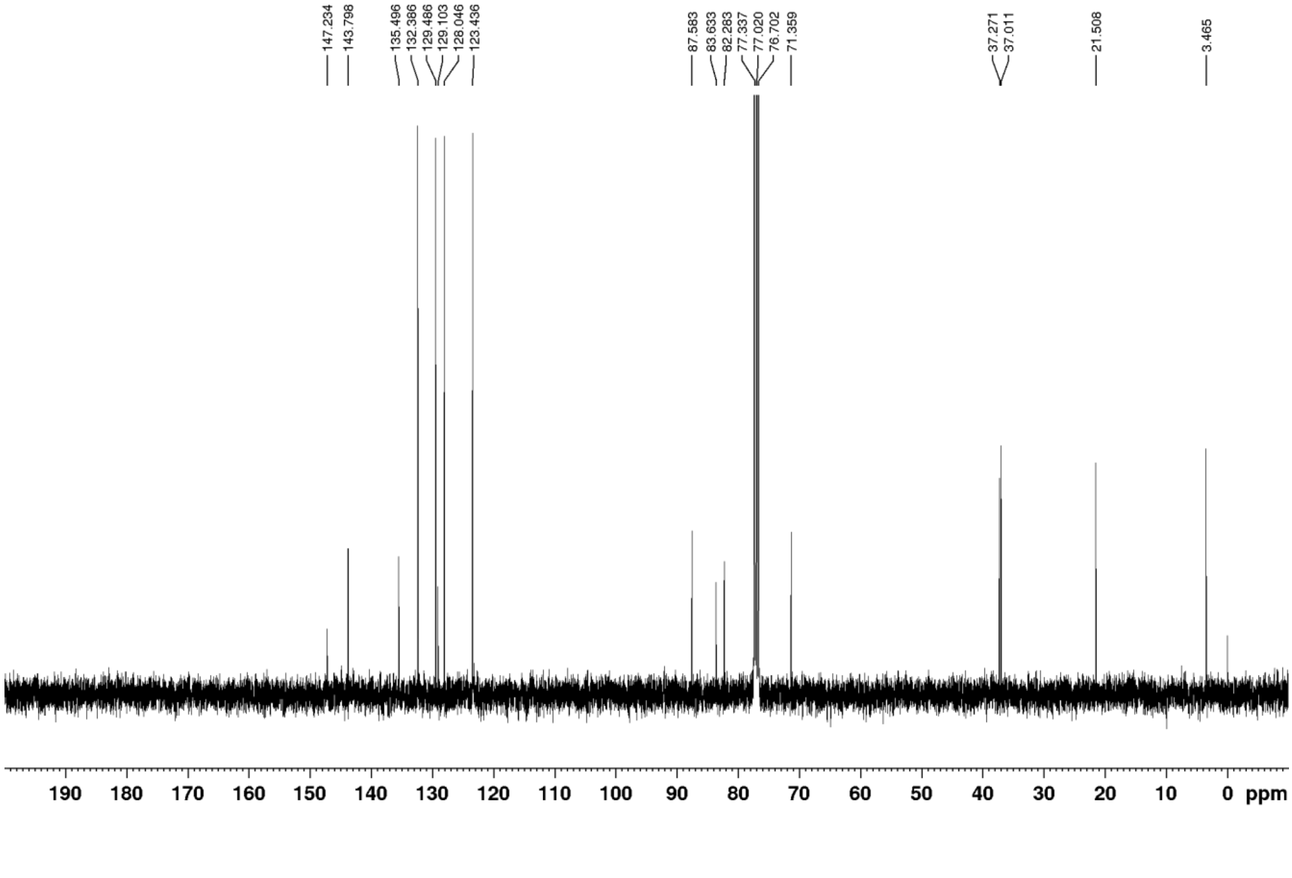

***N*-(But-2-yn-1-yl)-4-methyl-*N*-(3-(naphthalen-1-yl)prop-2-yn-1-yl)benzenesulfonamide (1n)**

^{1}H NMR ($CDCl_3$, 400 MHz)

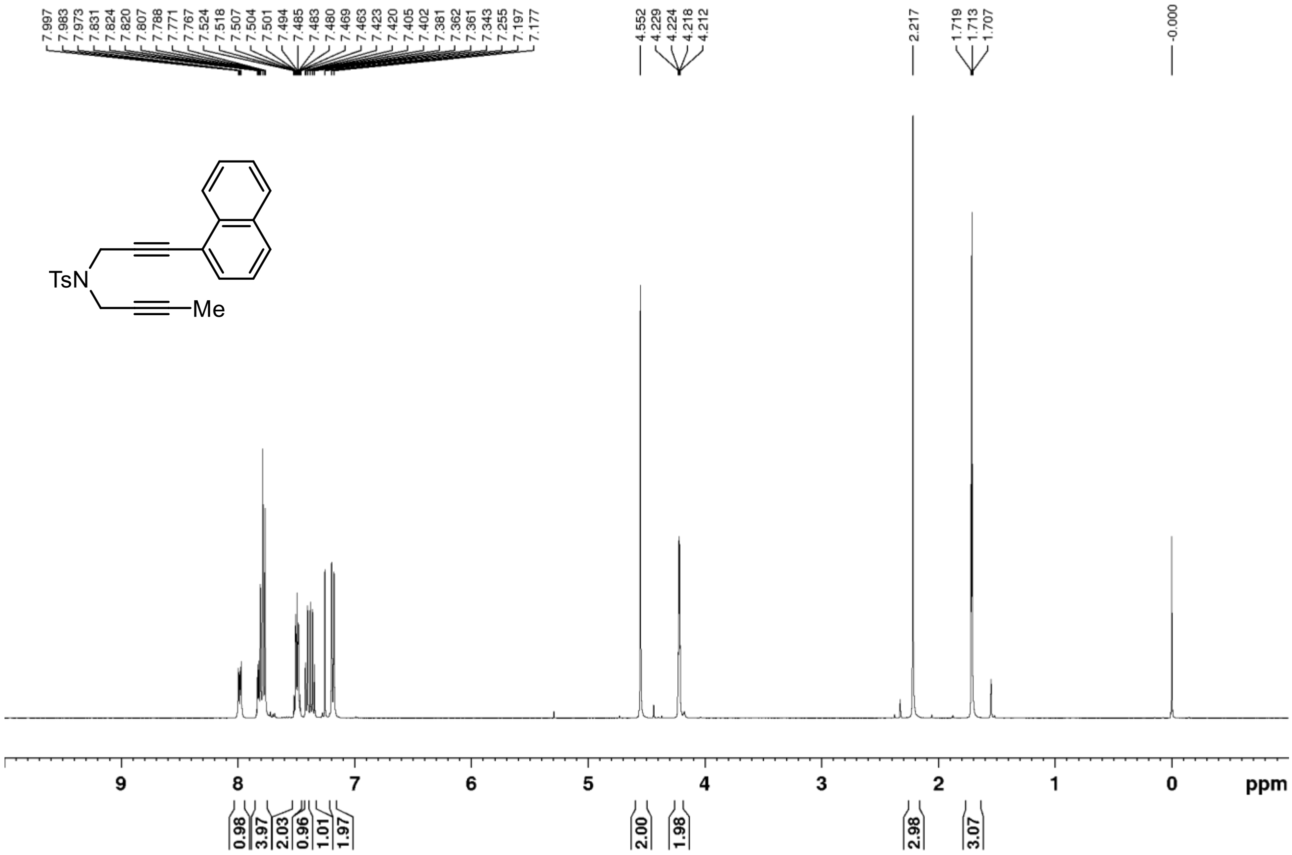

^{13}C NMR ($CDCl_3$, 101 MHz)

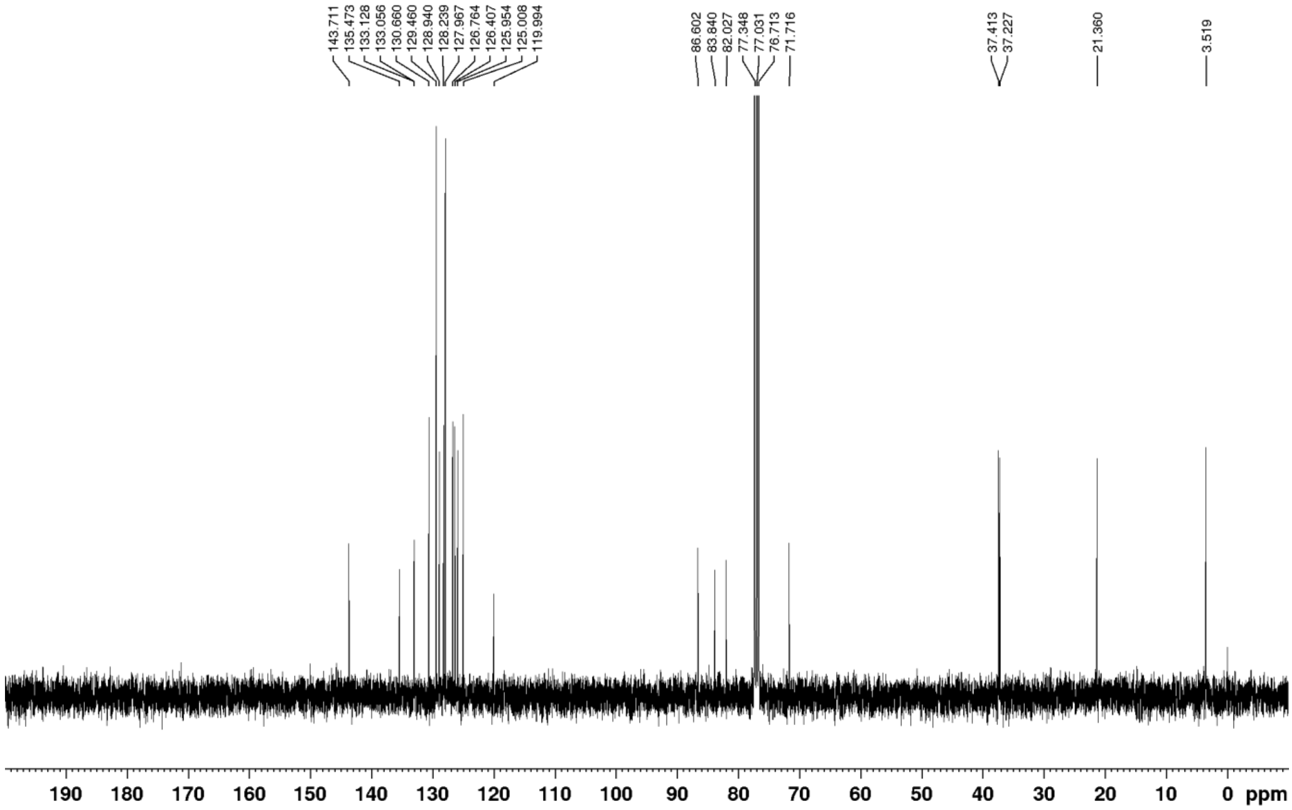

***N*-(But-2-yn-1-yl)-4-methyl-*N*-(3-(phenanthren-9-yl)prop-2-yn-1-yl)benzenesulfonamide (1o)**

^{1}H NMR ($CDCl_3$, 400 MHz)

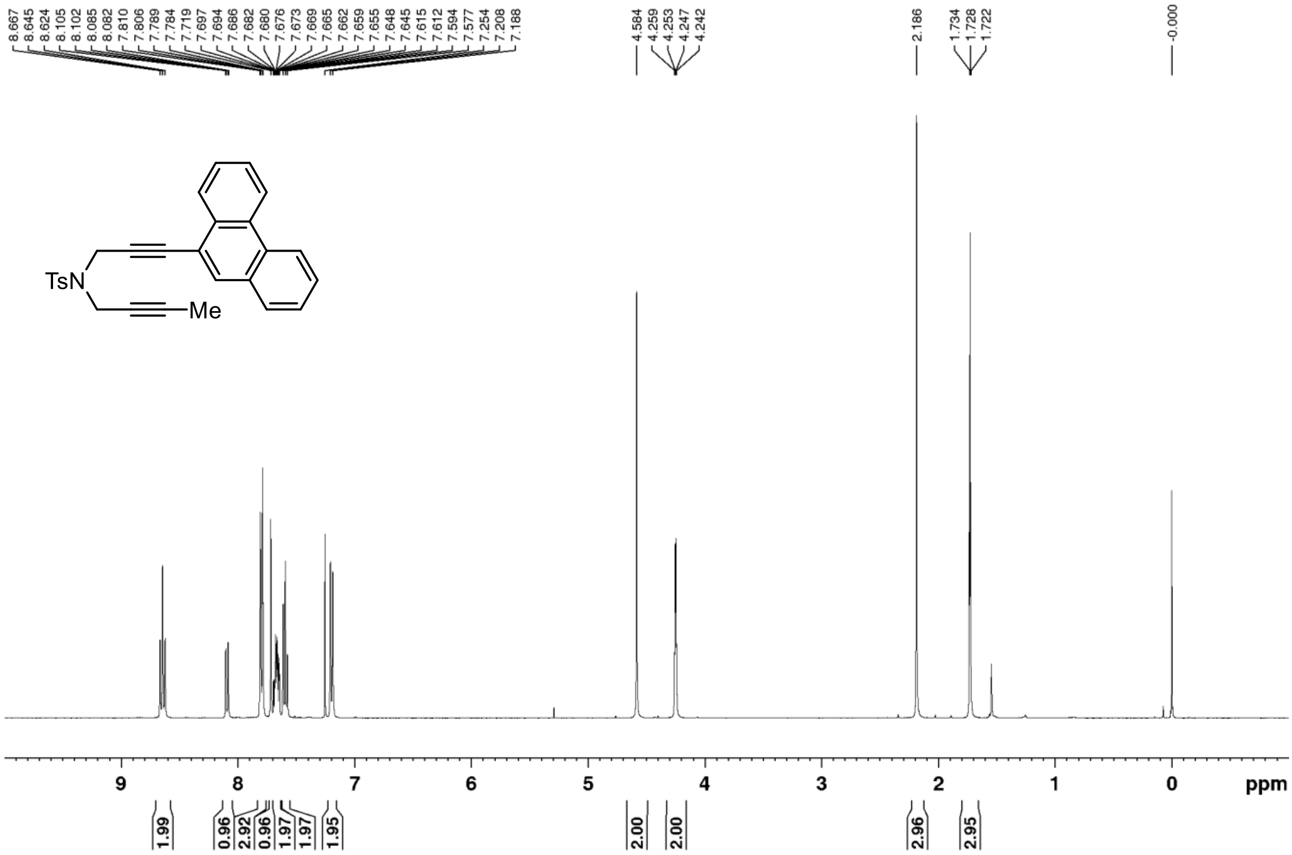

^{13}C NMR ($CDCl_3$, 101 MHz)

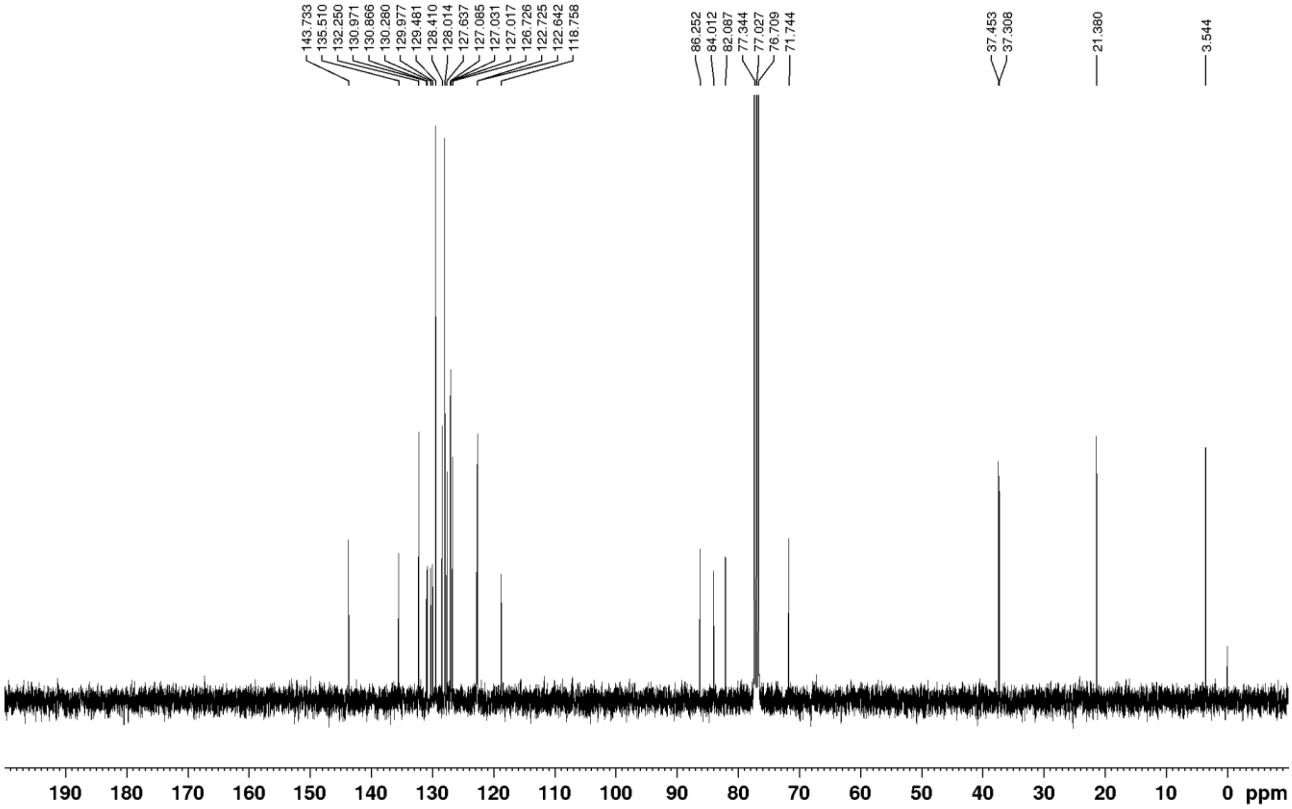

_N_-(But-2-yn-1-yl)-4-methyl-_N_-(3-(pyren-4-yl)prop-2-yn-1-yl)benzenesulfonamide (1p)

^{1}H NMR ($CDCl_3$, 400 MHz)

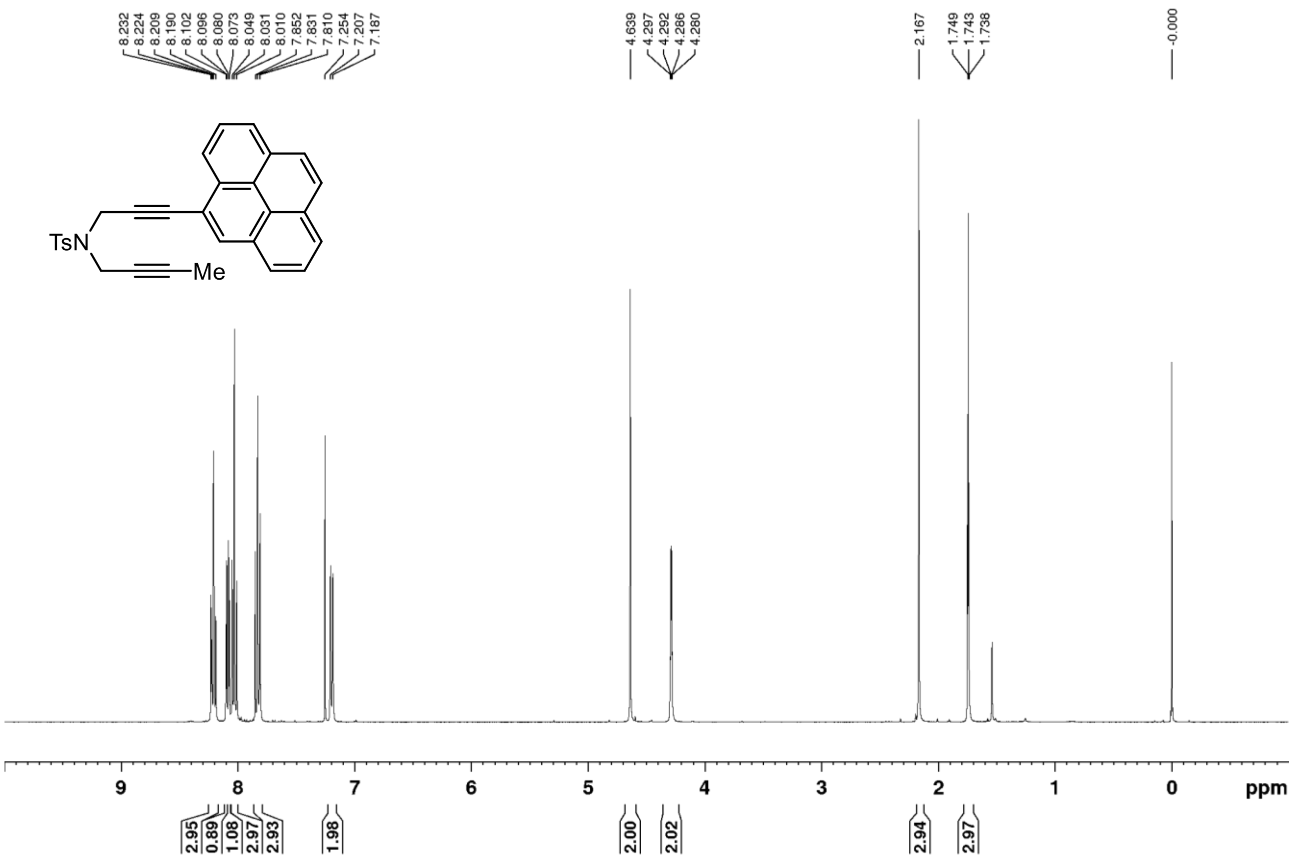

^{13}C NMR ($CDCl_3$, 101 MHz)

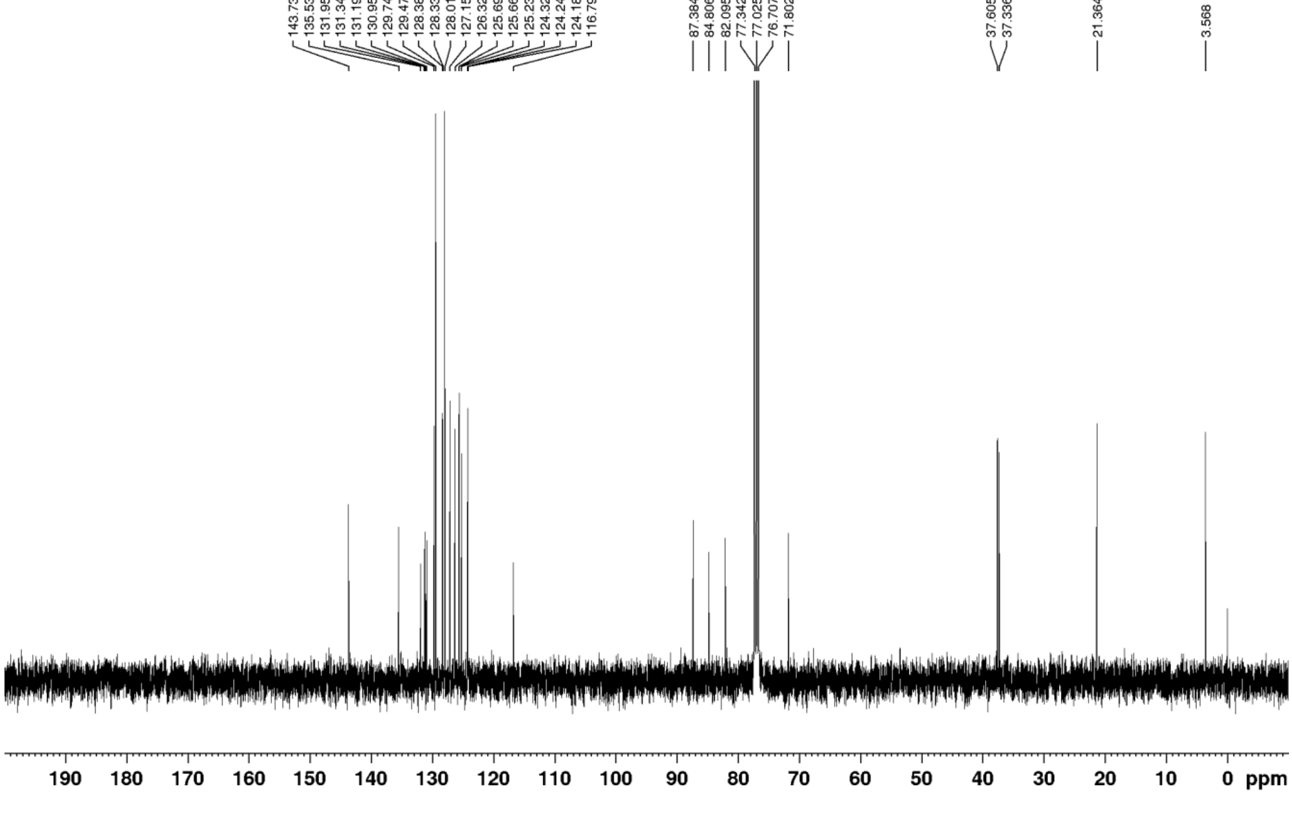

***N*-(3-(1H-Indol-7-yl)prop-2-yn-1-yl)-*N*-(but-2-yn-1-yl)-4-methylbenzenesulfonamide (1q)**

^{1}H NMR ($CDCl_3$, 400 MHz)

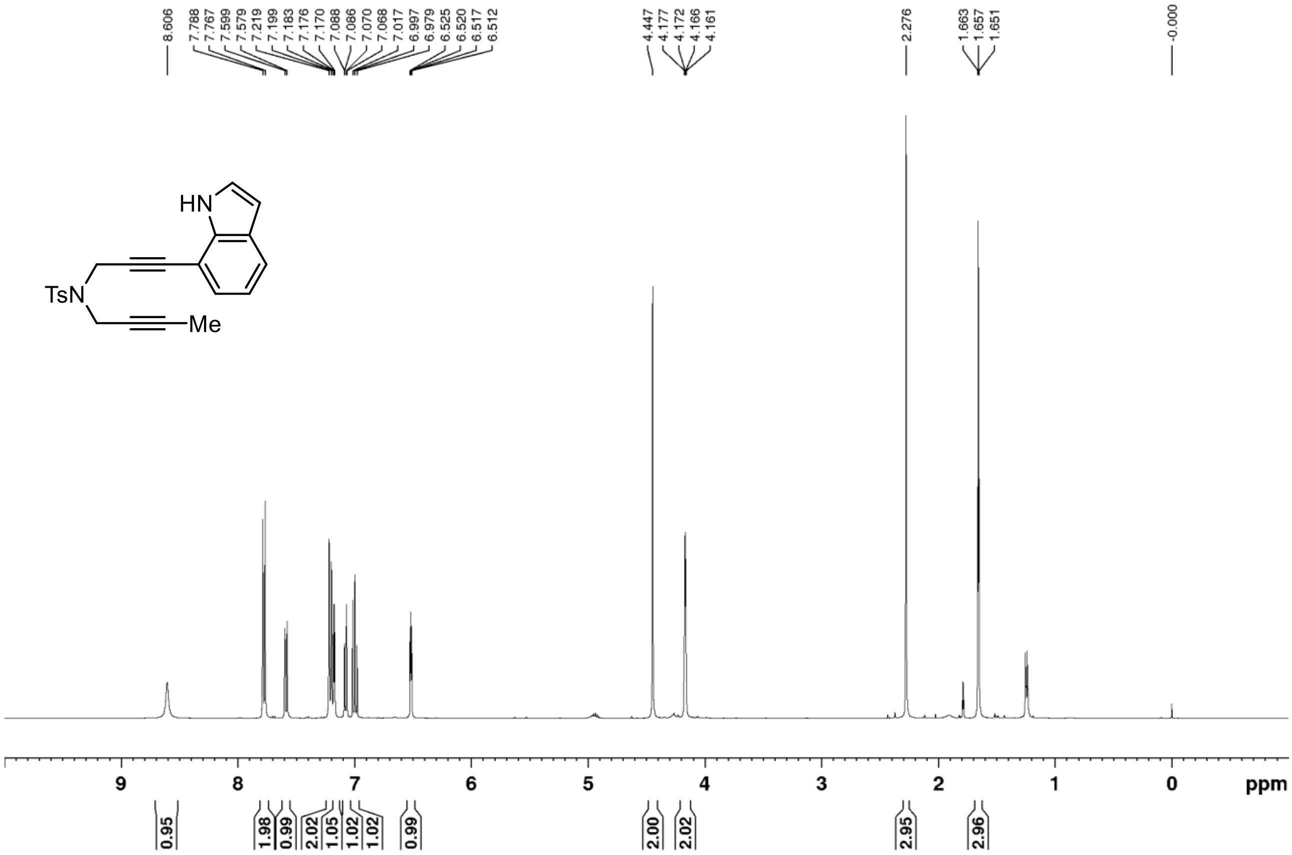

^{13}C NMR ($CDCl_3$, 101 MHz)

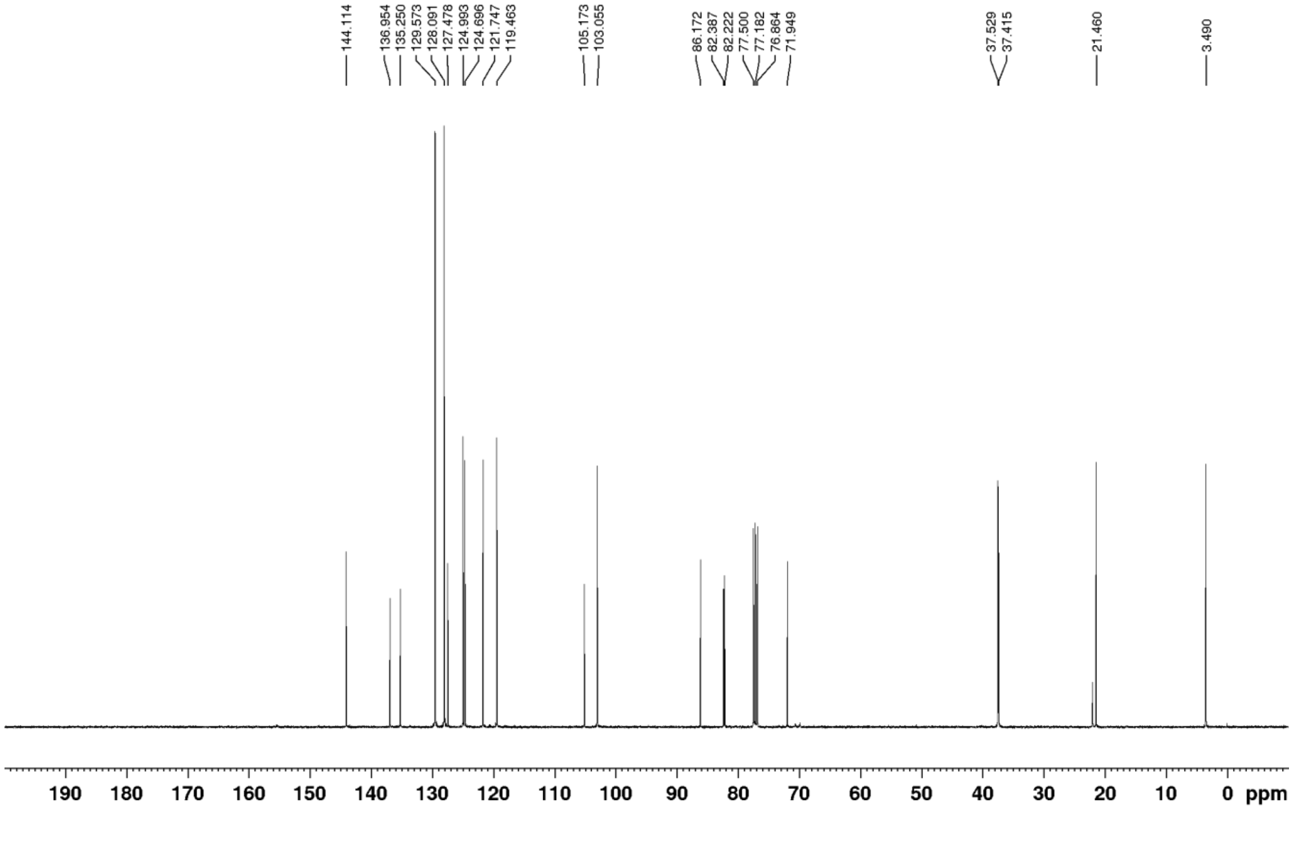

N-(3-([1,1'-Biphenyl]-2-yl)prop-2-yn-1-yl)-N-(but-2-yn-1-yl)-4-methylbenzenesulfonamide (1r)

^{1}H NMR ($CDCl_3$, 400 MHz)

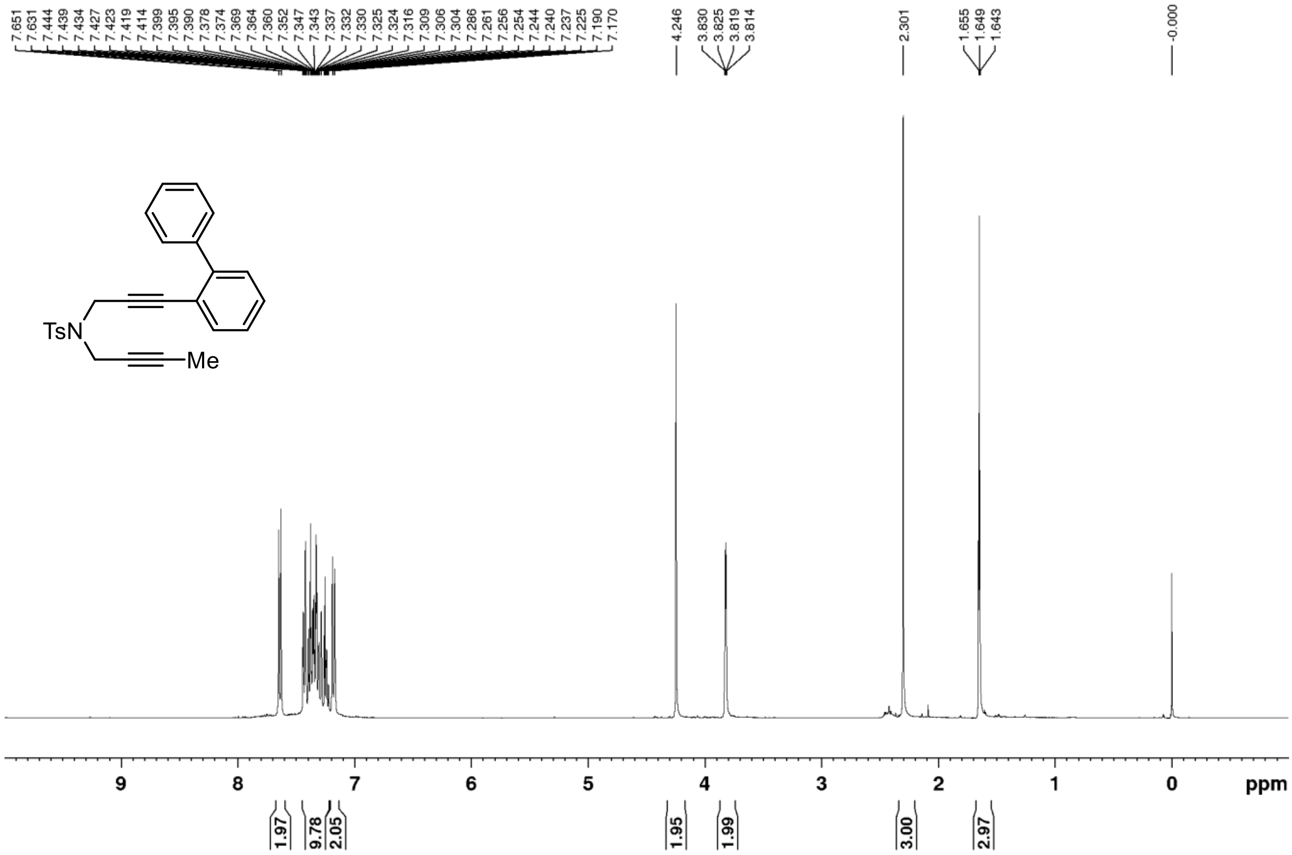

^{13}C NMR ($CDCl_3$, 101 MHz)

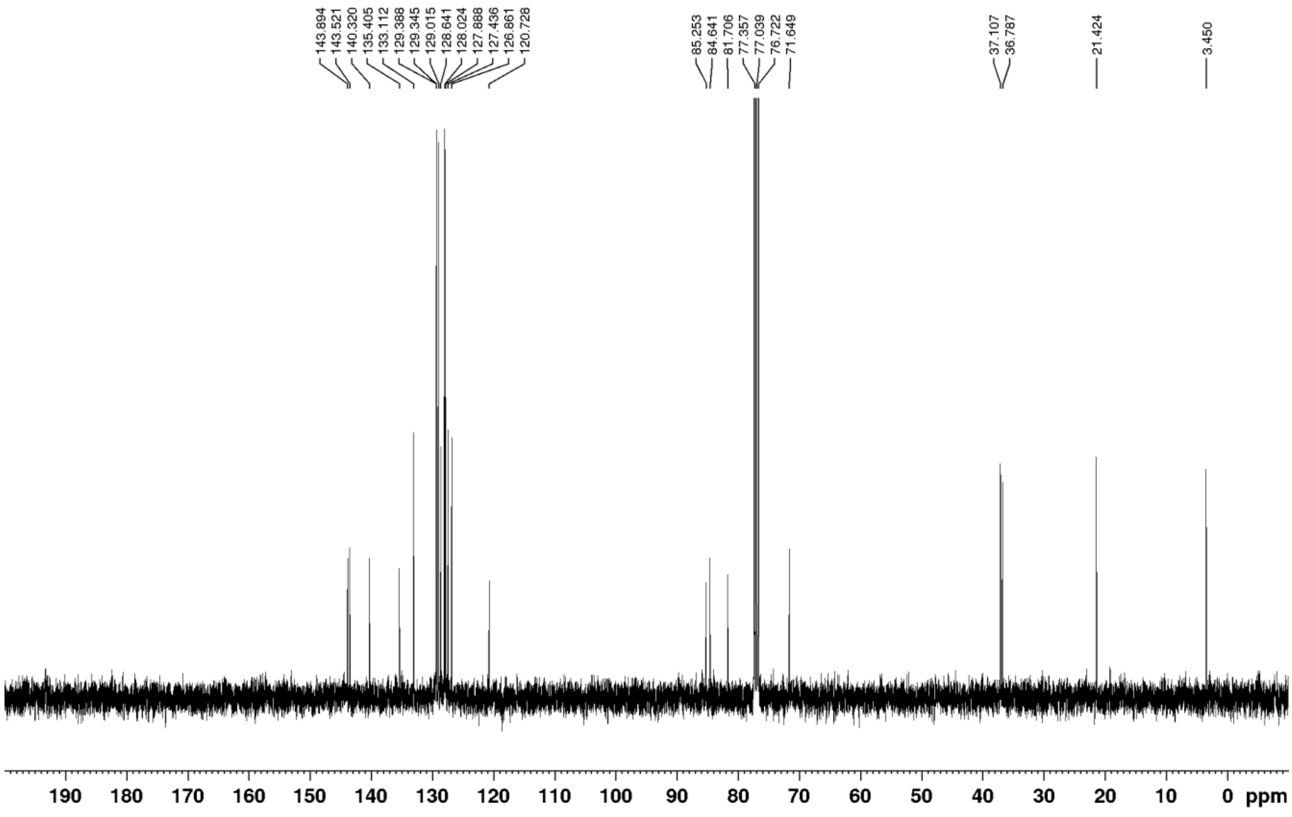

_N_-(3-(2-Aminophenyl)prop-2-yn-1-yl)-4-methyl-_N_-(3-(naphthalen-1-yl)prop-2-yn-1-yl)benzenesulfonamide (1u)

^{1}H NMR ($CDCl_3$, 400 MHz)

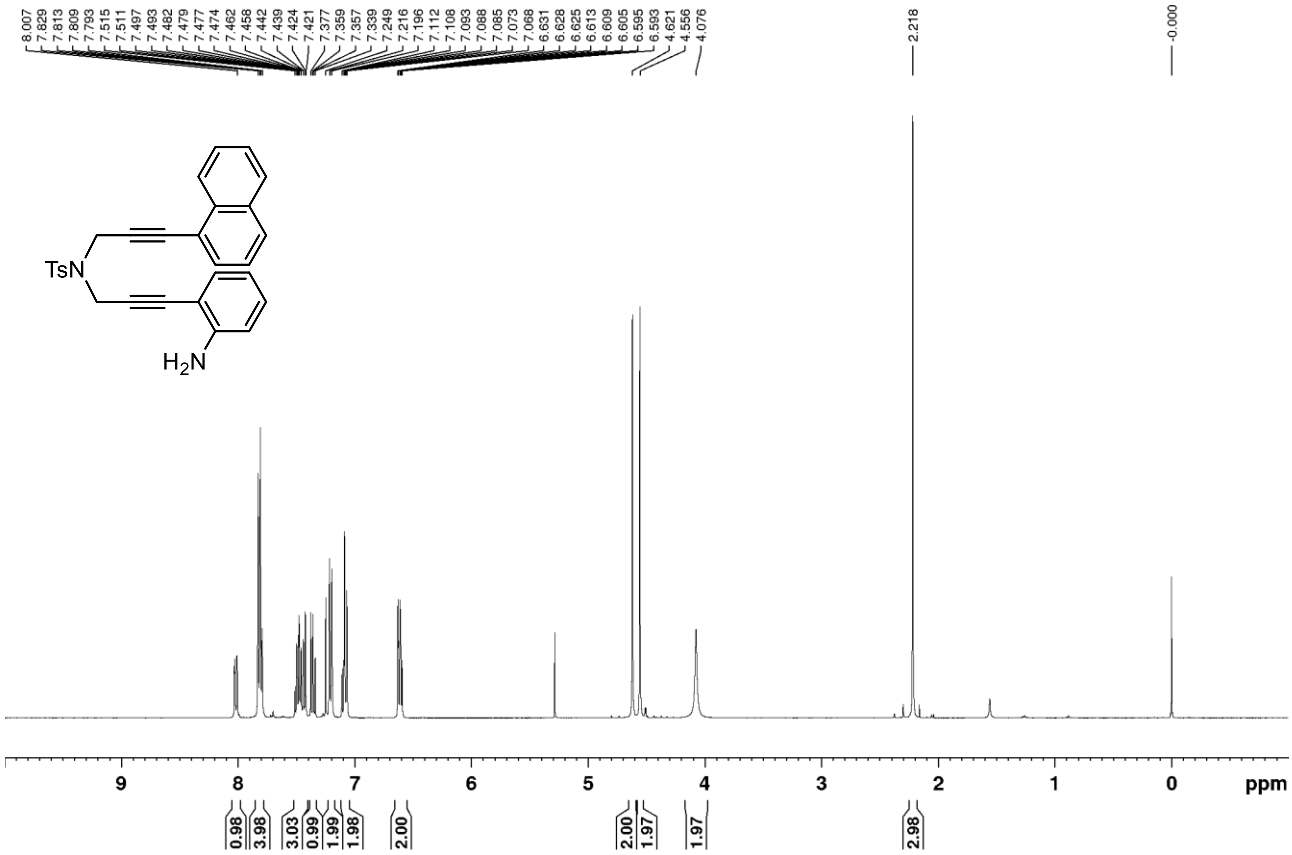

^{13}C NMR ($CDCl_3$, 101 MHz)

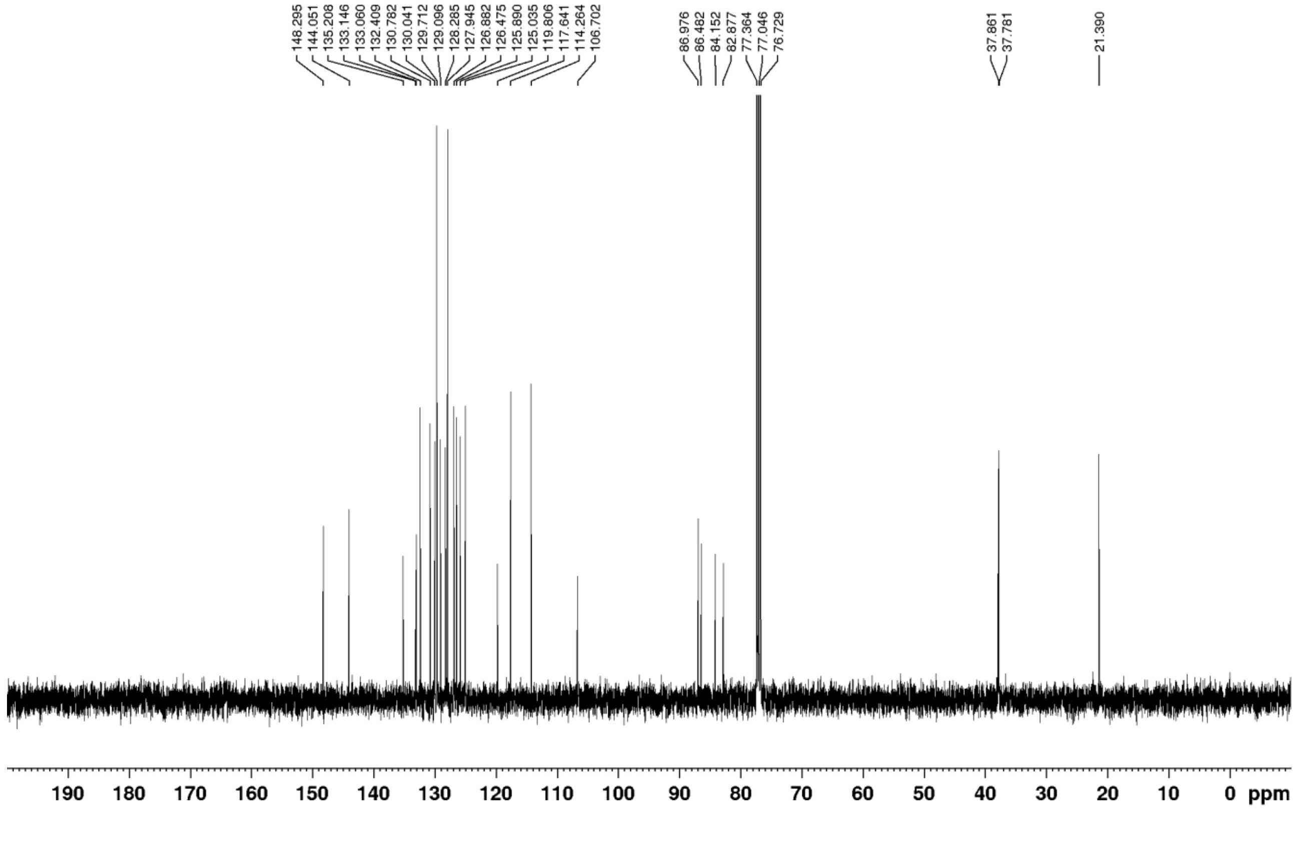

(*E*)-1-(*tert*-Butyl)-2-((1,2-dichlorovinyl)oxy)-3-methylbenzene (S3a)

^{1}H NMR ($CDCl_3$, 400 MHz)

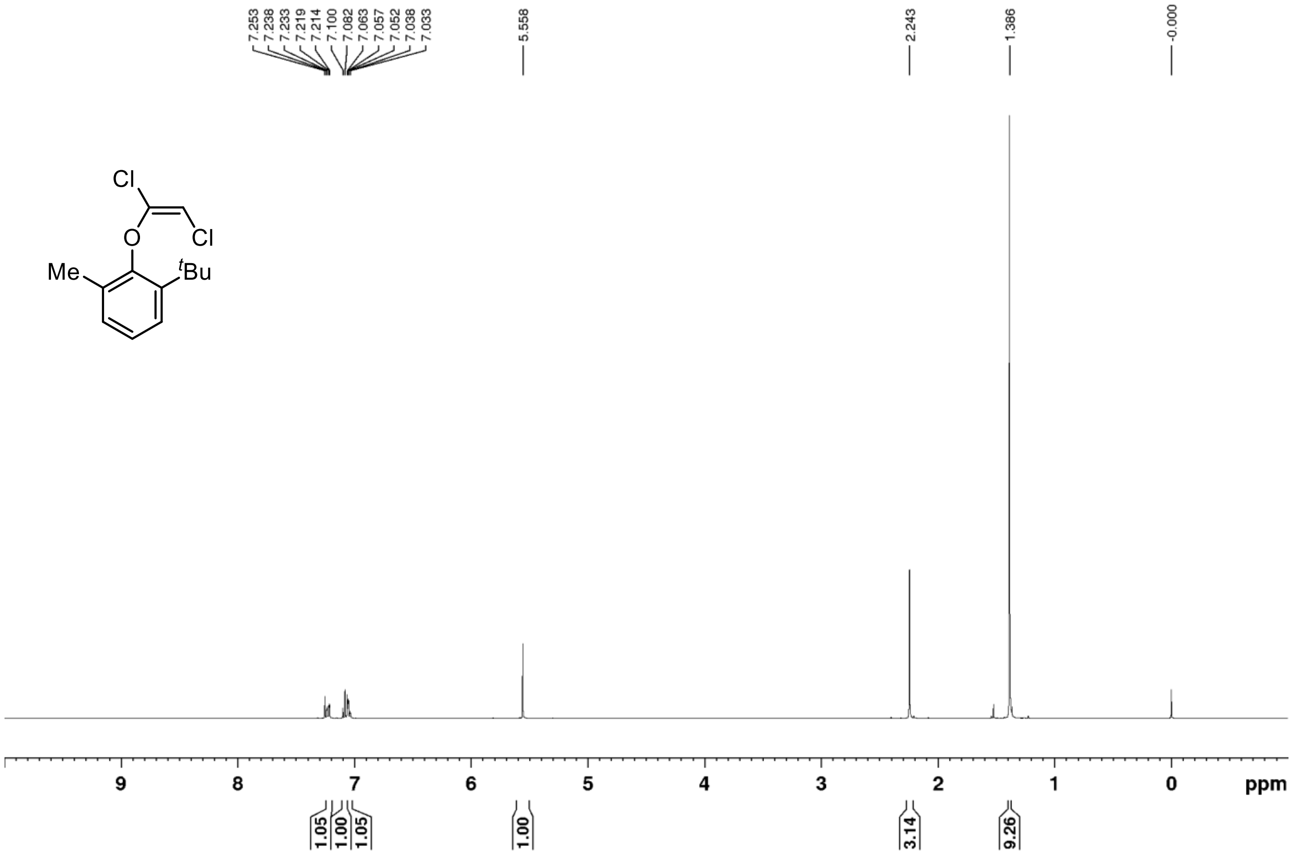

^{13}C NMR ($CDCl_3$, 101 MHz)

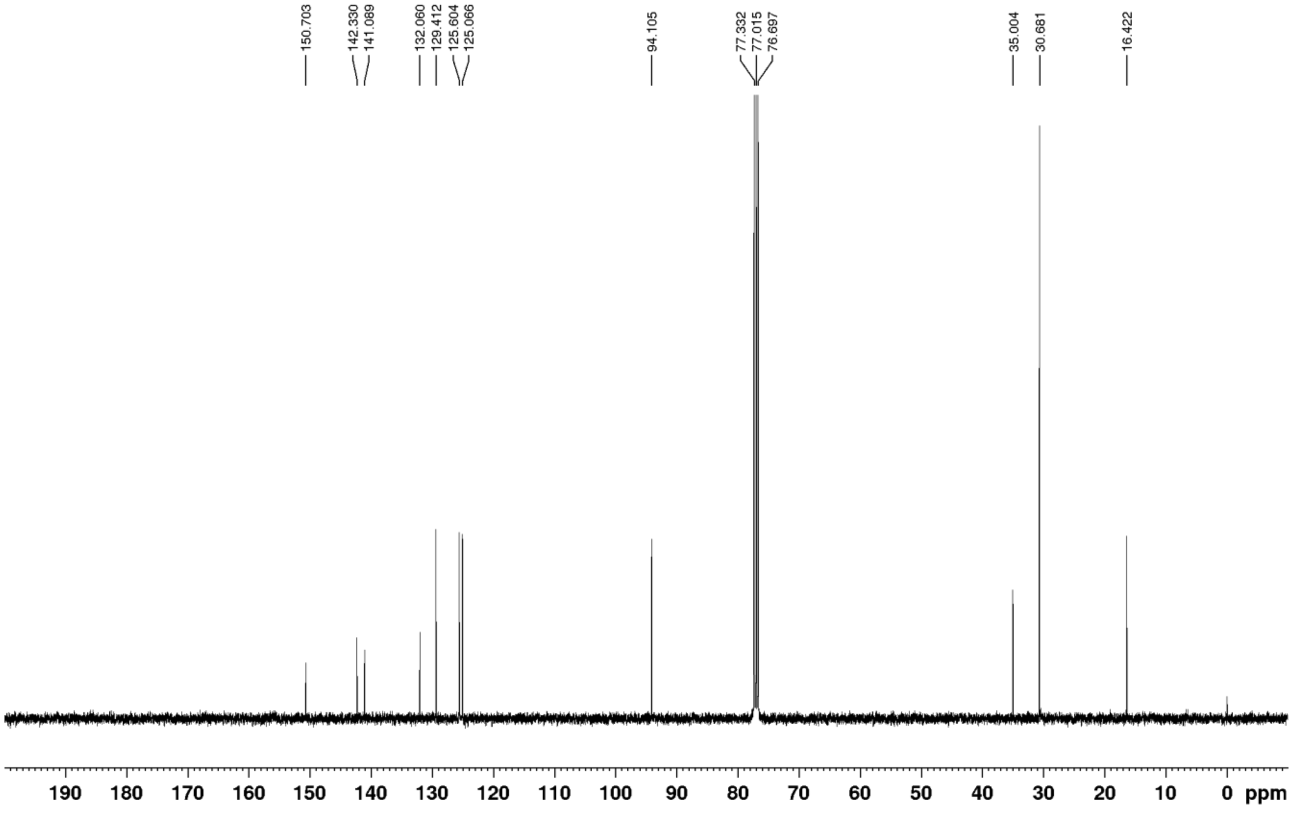

Methyl 3-(2-(*tert*-butyl)-6-methylphenoxy)propiolate (2a)

^{1}H NMR ($CDCl_3$, 400 MHz)

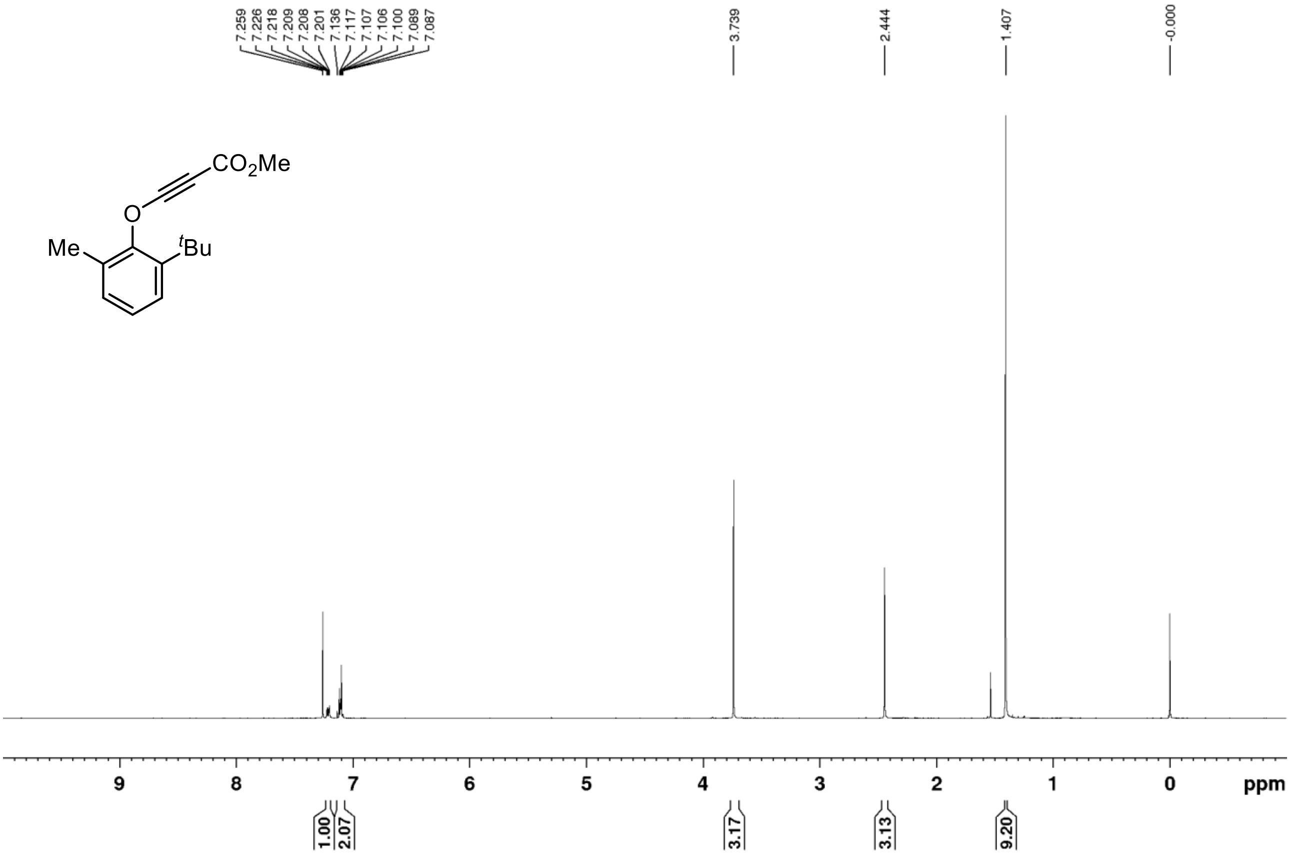

^{13}C NMR ($CDCl_3$, 101 MHz)

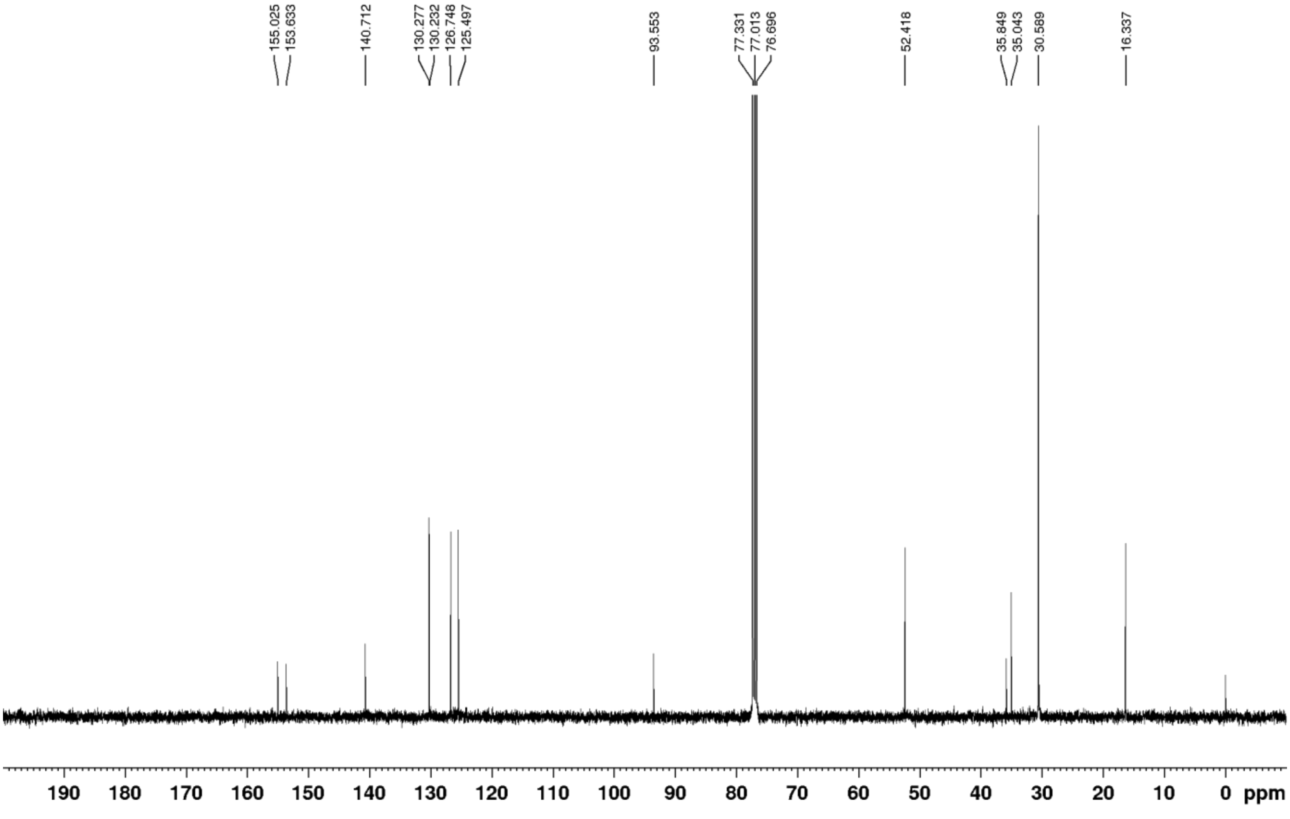

(*E*)-1-(*tert*-Butyl)-2-((1,2-dichlorovinyl)oxy)-3-methoxy-5-methylbenzene (S3b)

^{1}H NMR ($CDCl_3$, 400 MHz)

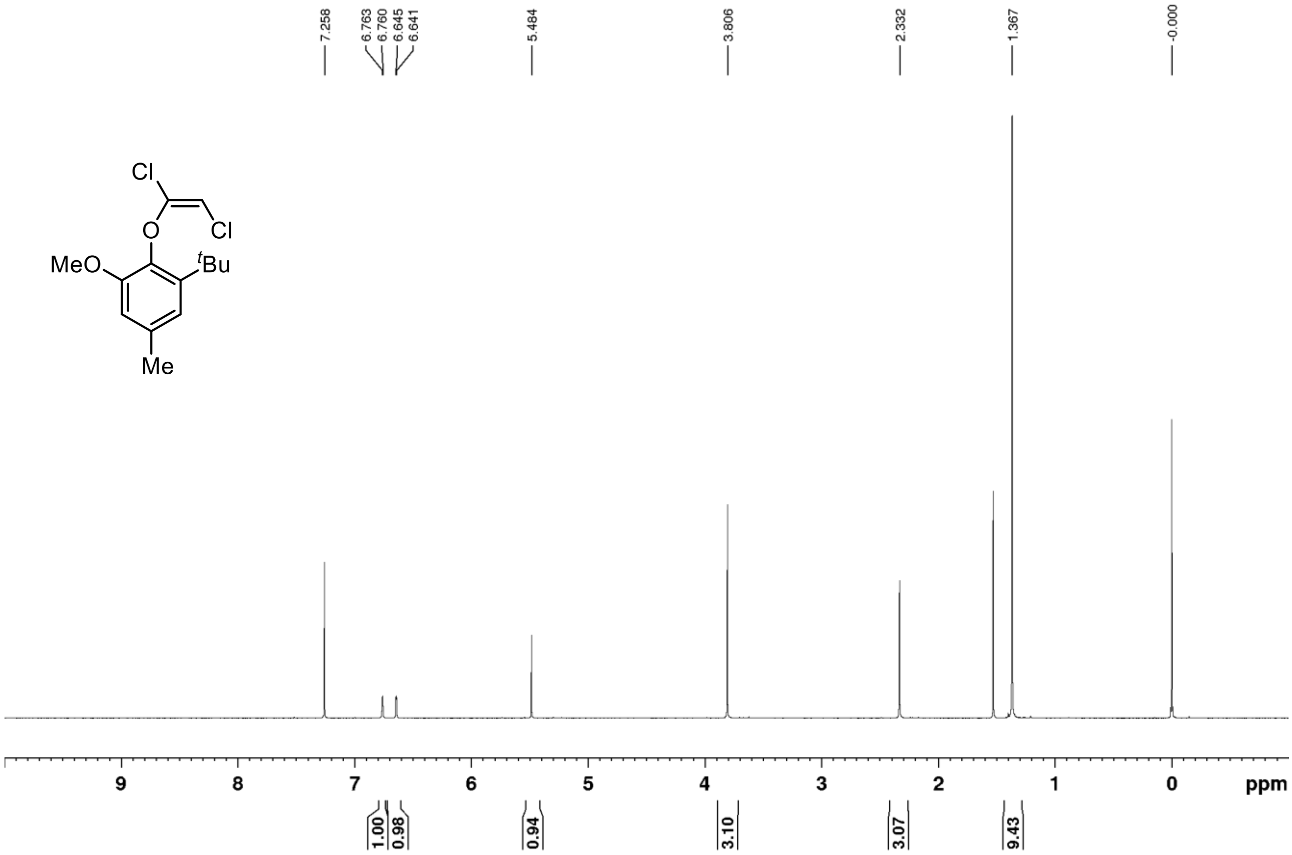

^{13}C NMR ($CDCl_3$, 101 MHz)

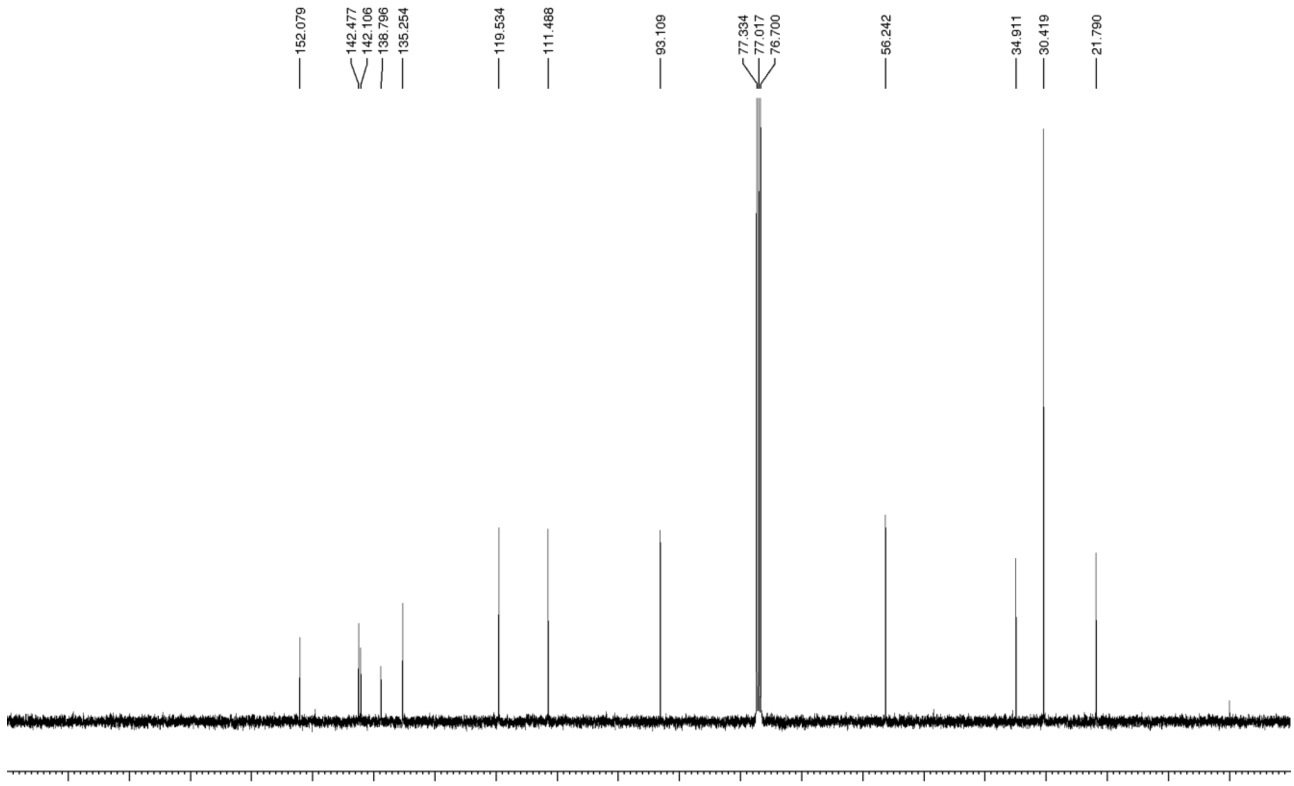

Methyl 3-(2-(*tert*-butyl)-6-methoxy-4-methylphenoxy)propiolate (2b)

^{1}H NMR ($CDCl_3$, 400 MHz)

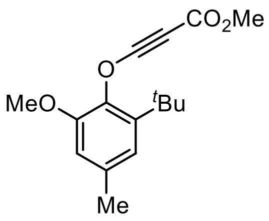

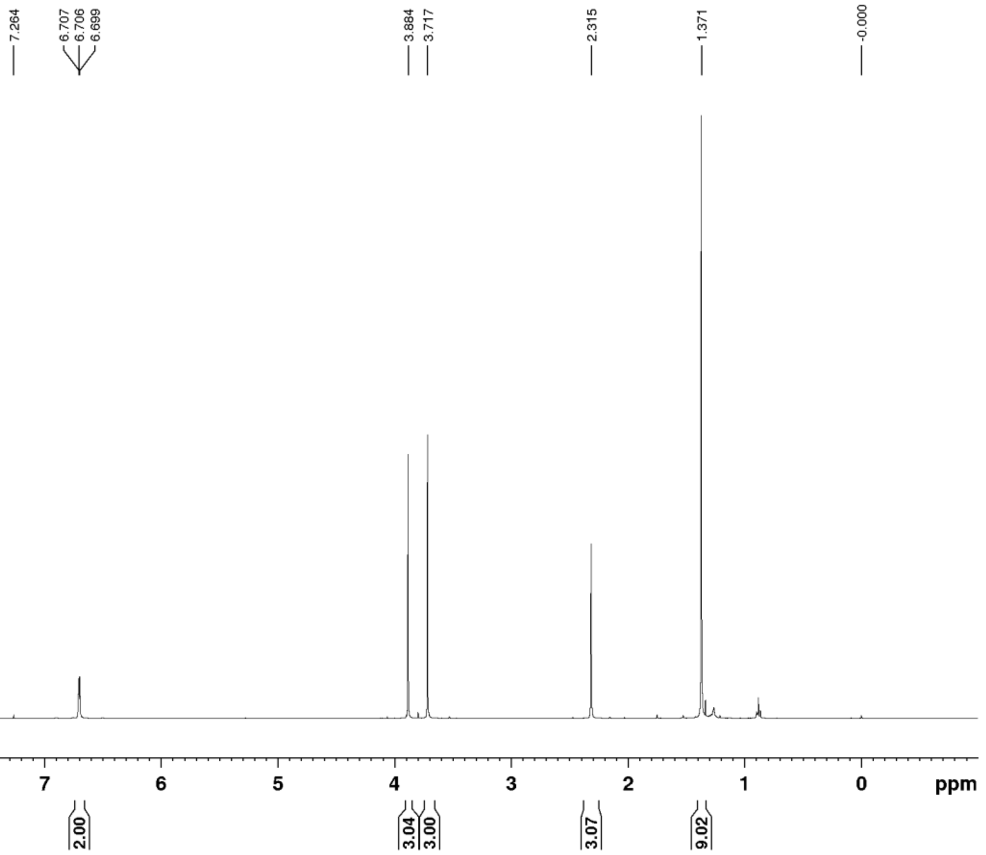

^{13}C NMR ($CDCl_3$, 101 MHz)

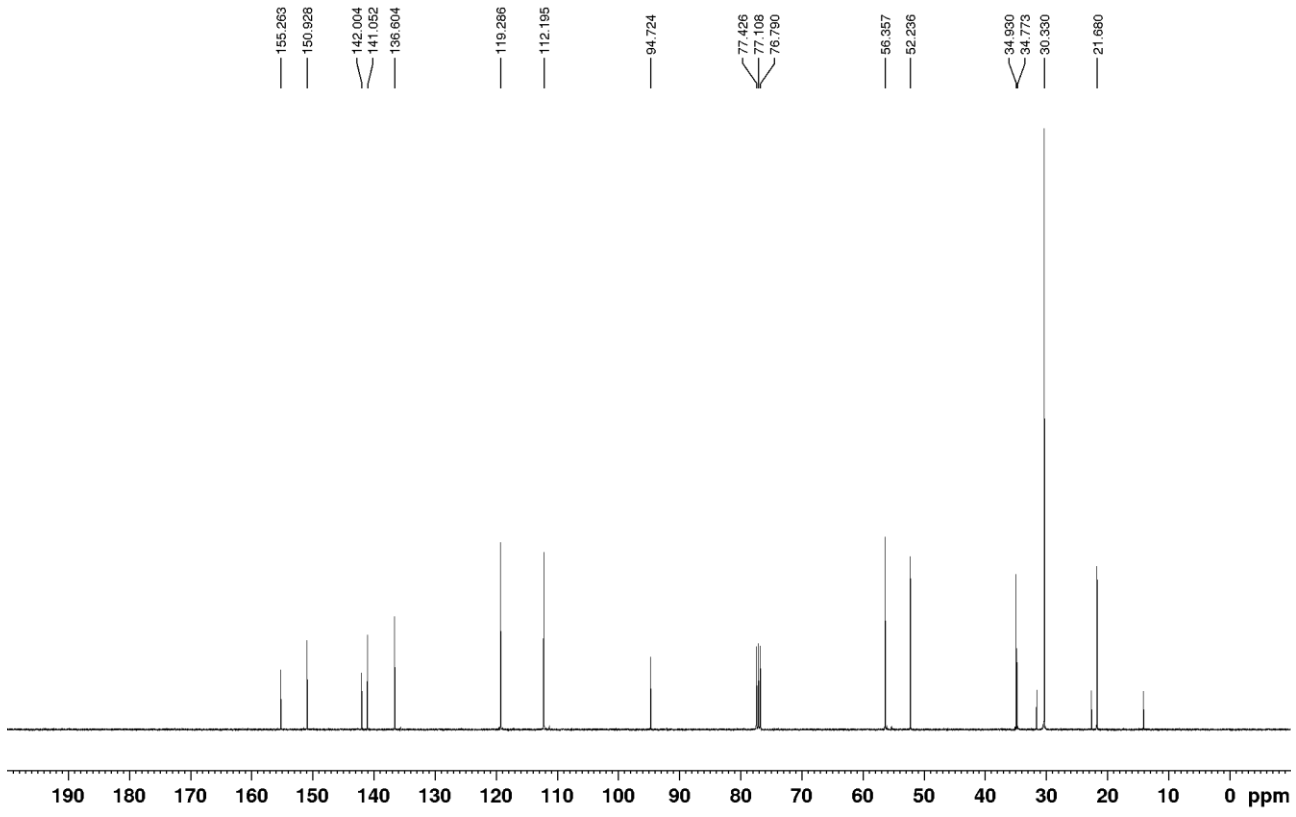

(*E*)-3-(*tert*-Butyl)-2-((1,2-dichlorovinyl)oxy)-4',5-dimethyl-1,1'-biphenyl (S3c)

^{1}H NMR ($CDCl_3$, 400 MHz)

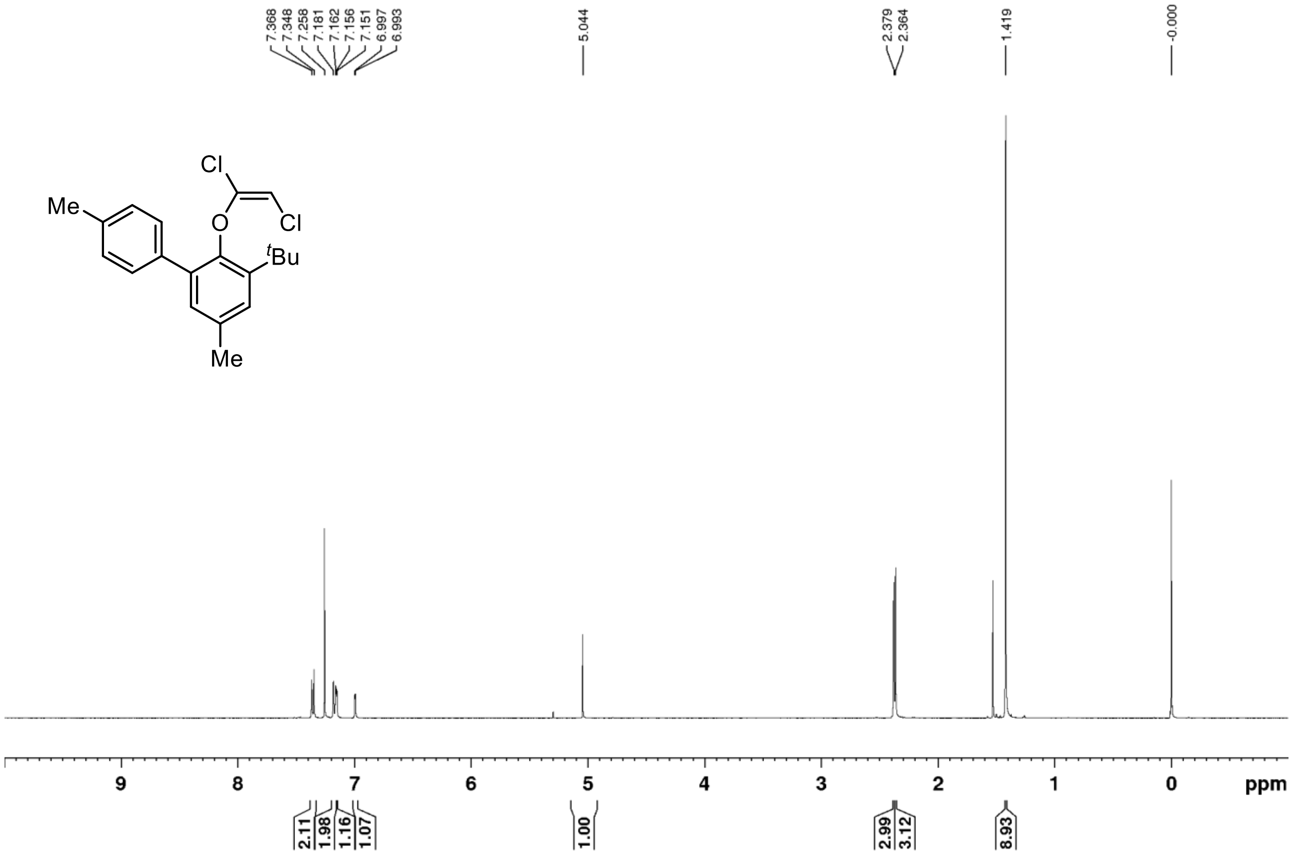

^{13}C NMR ($CDCl_3$, 101 MHz)

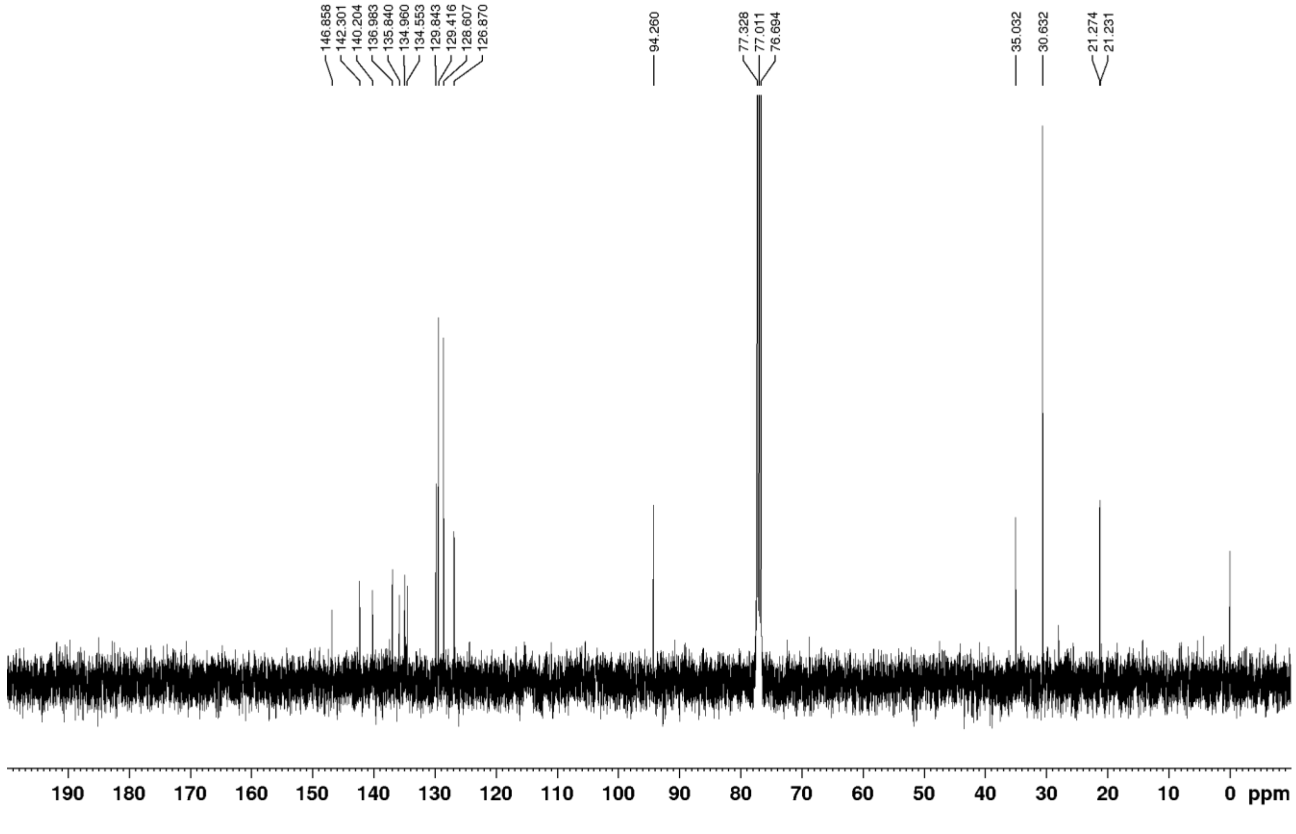

Methyl 3-((3-(*tert*-butyl)-4',5-dimethyl-[1,1'-biphenyl]-2-yl)oxy)propiolate (2c)

^{1}H NMR ($CDCl_3$, 400 MHz)

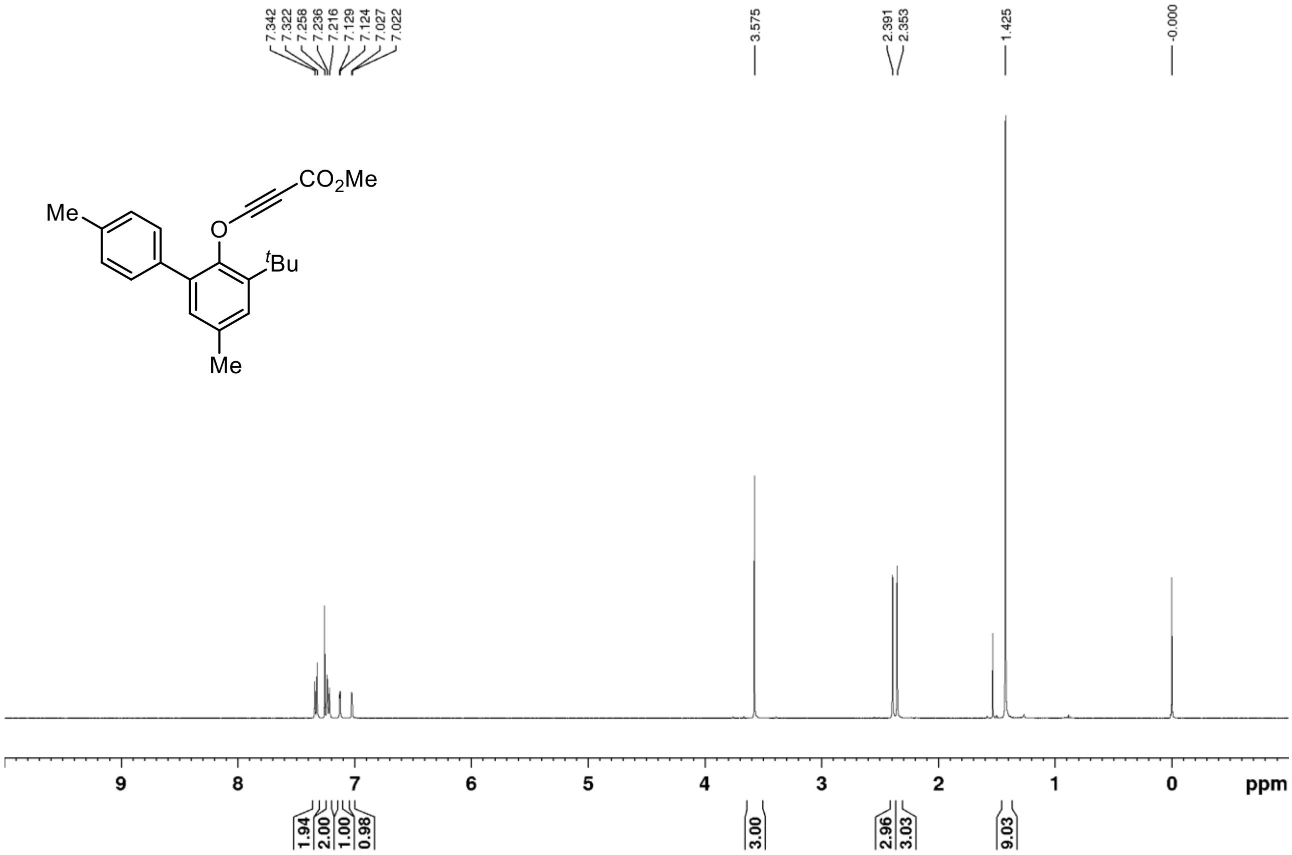

^{13}C NMR ($CDCl_3$, 101 MHz)

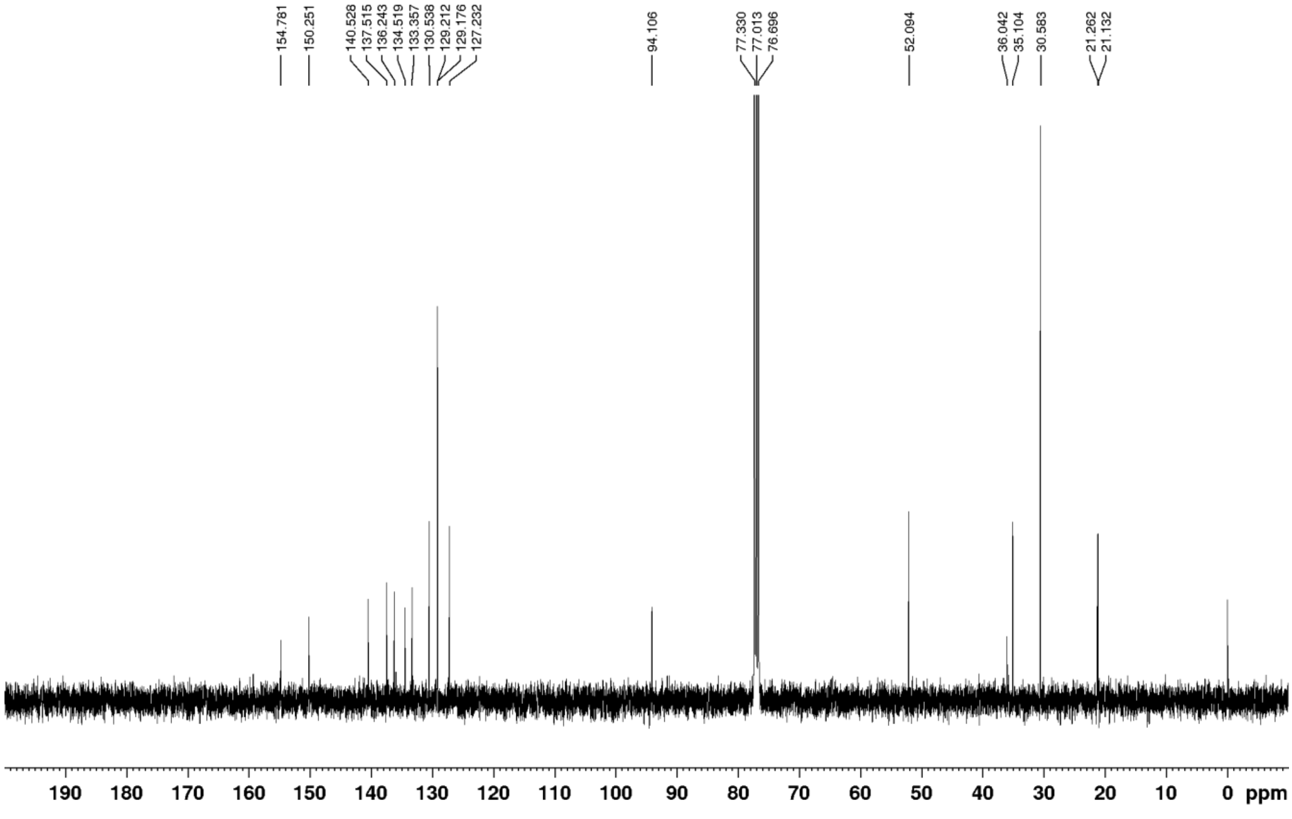

Methyl 3-(2-(*tert*-butyl)-6-ethoxy-4-methylphenoxy)propiolate (2d)

^{1}H NMR ($CDCl_3$, 400 MHz)

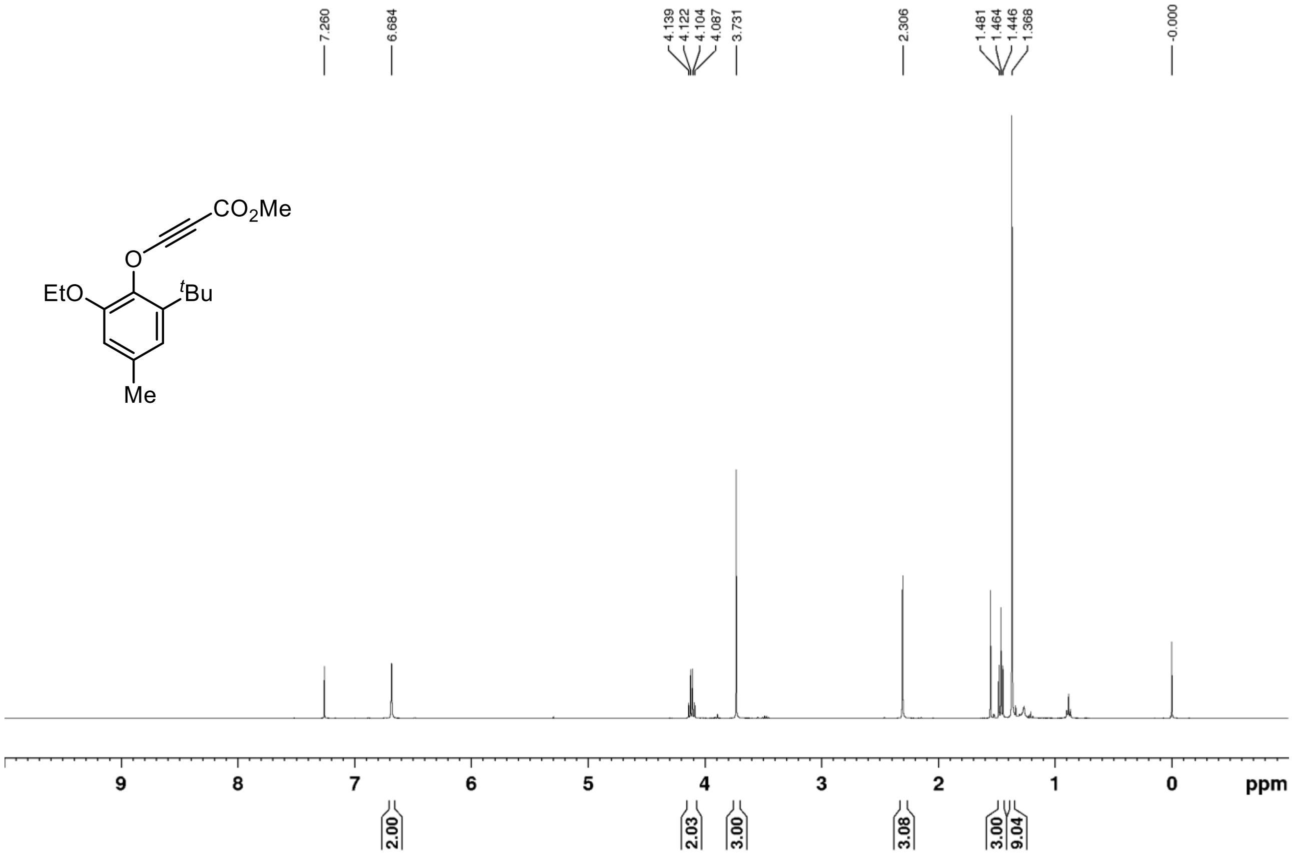

^{13}C NMR ($CDCl_3$, 101 MHz)

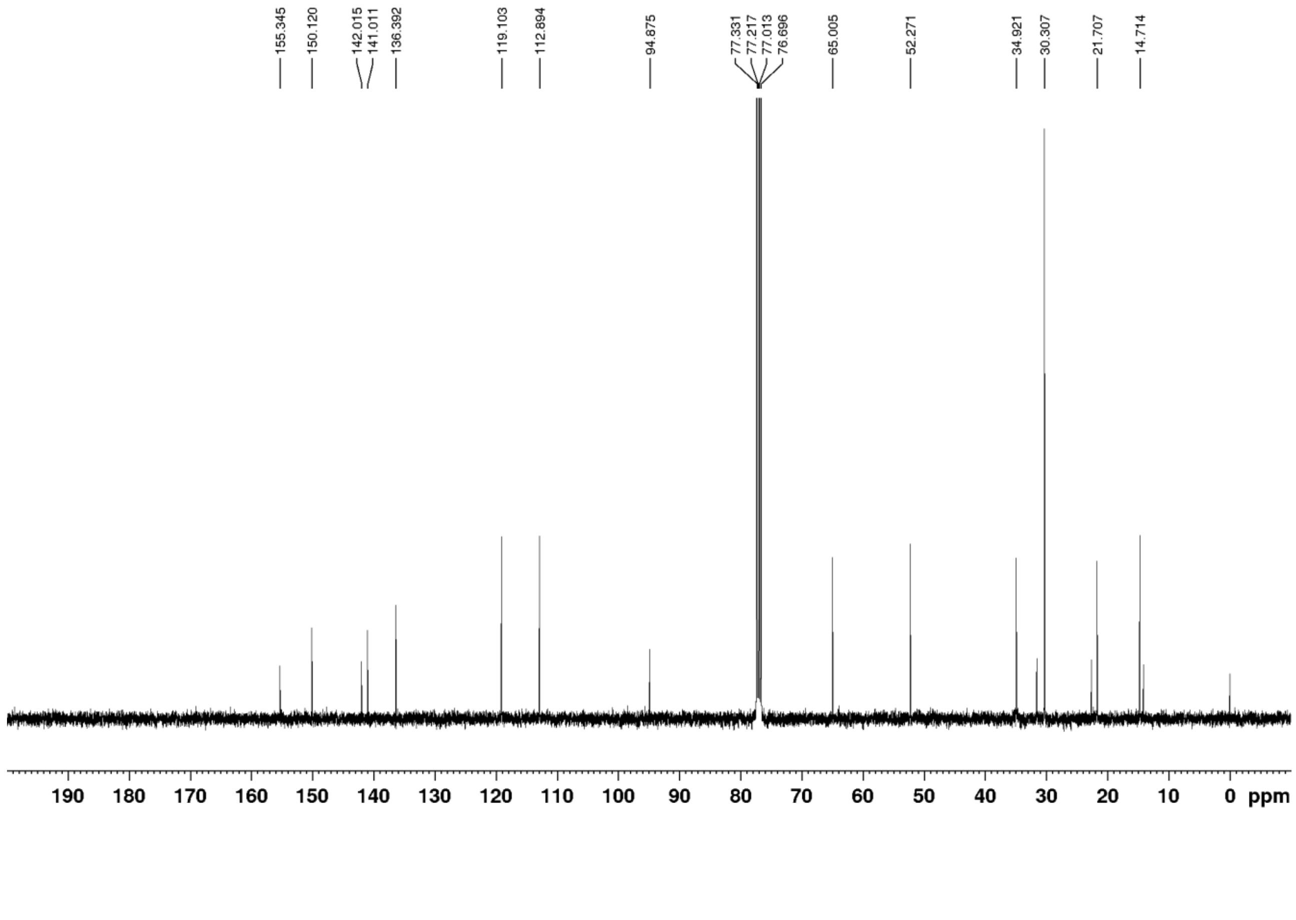

Methyl 3-(2-(*tert*-butyl)-6-(methoxymethoxy)-4-methylphenoxy)propiolate (2e)

^{1}H NMR ($CDCl_3$, 400 MHz)

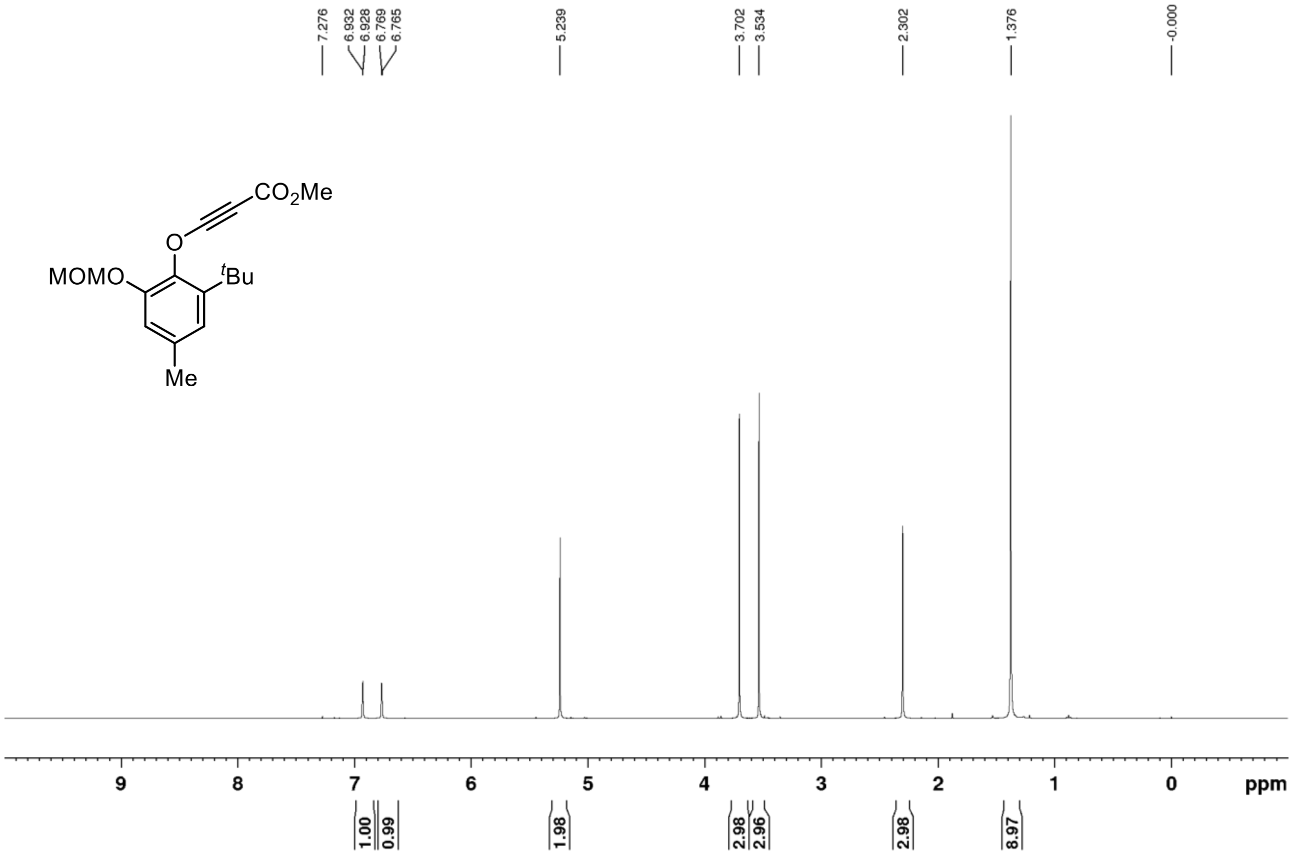

^{13}C NMR ($CDCl_3$, 101 MHz)

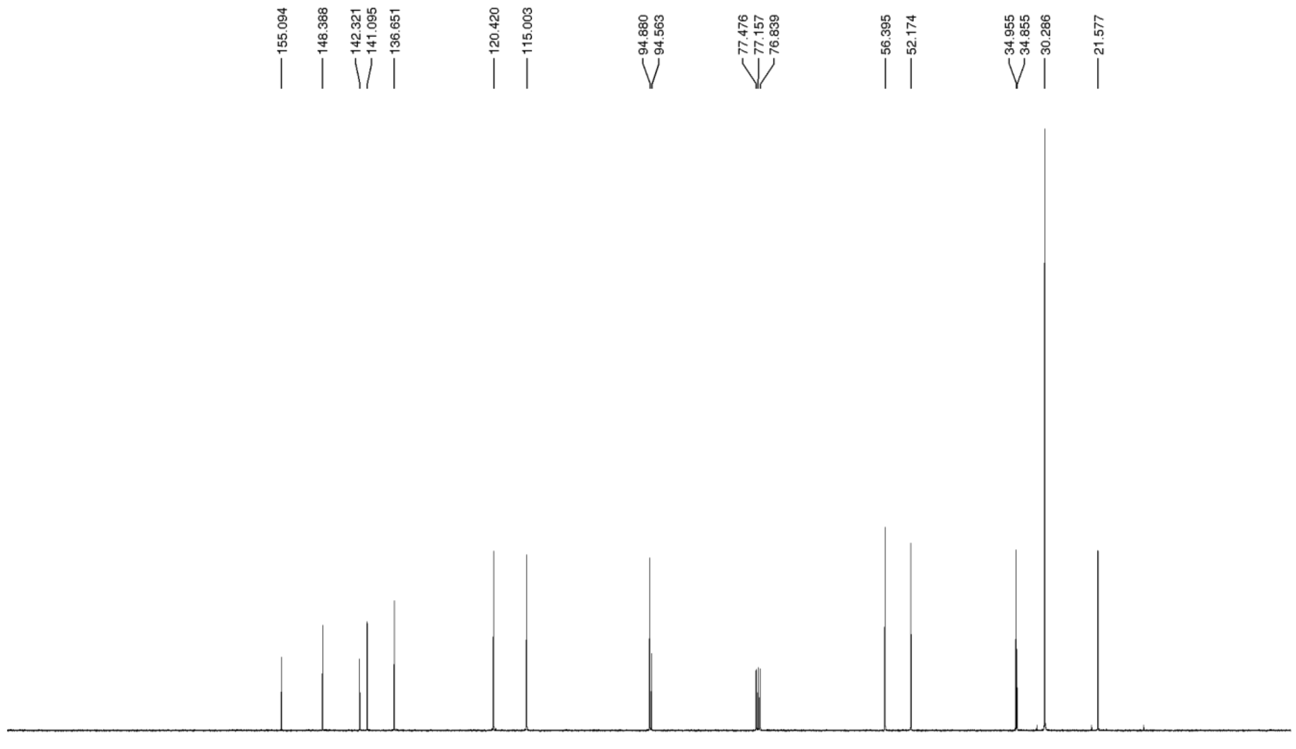

Ethyl 3-(2-(*tert*-butyl)-6-methoxy-4-methylphenoxy)propiolate (2f)

^{1}H NMR ($CDCl_3$, 400 MHz)

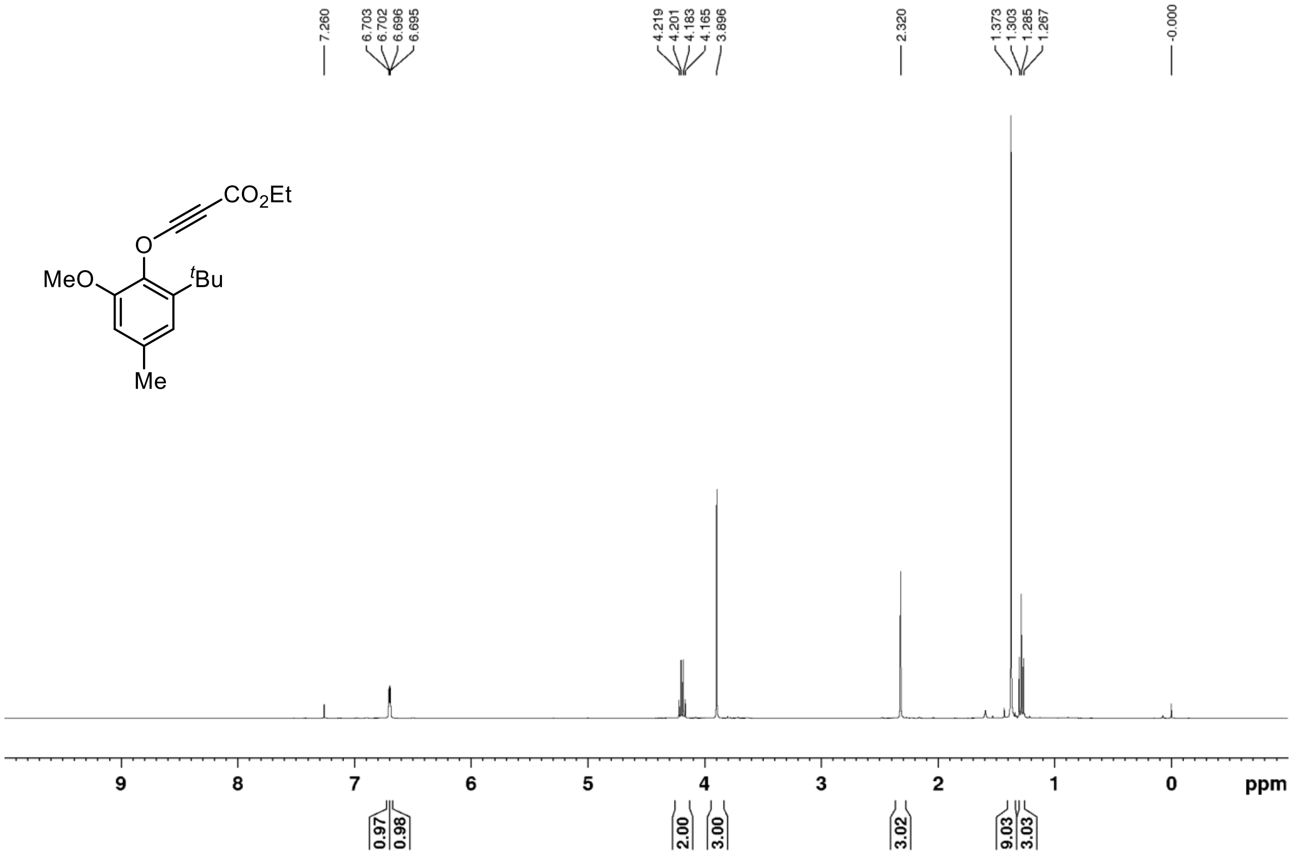

^{13}C NMR ($CDCl_3$, 101 MHz)

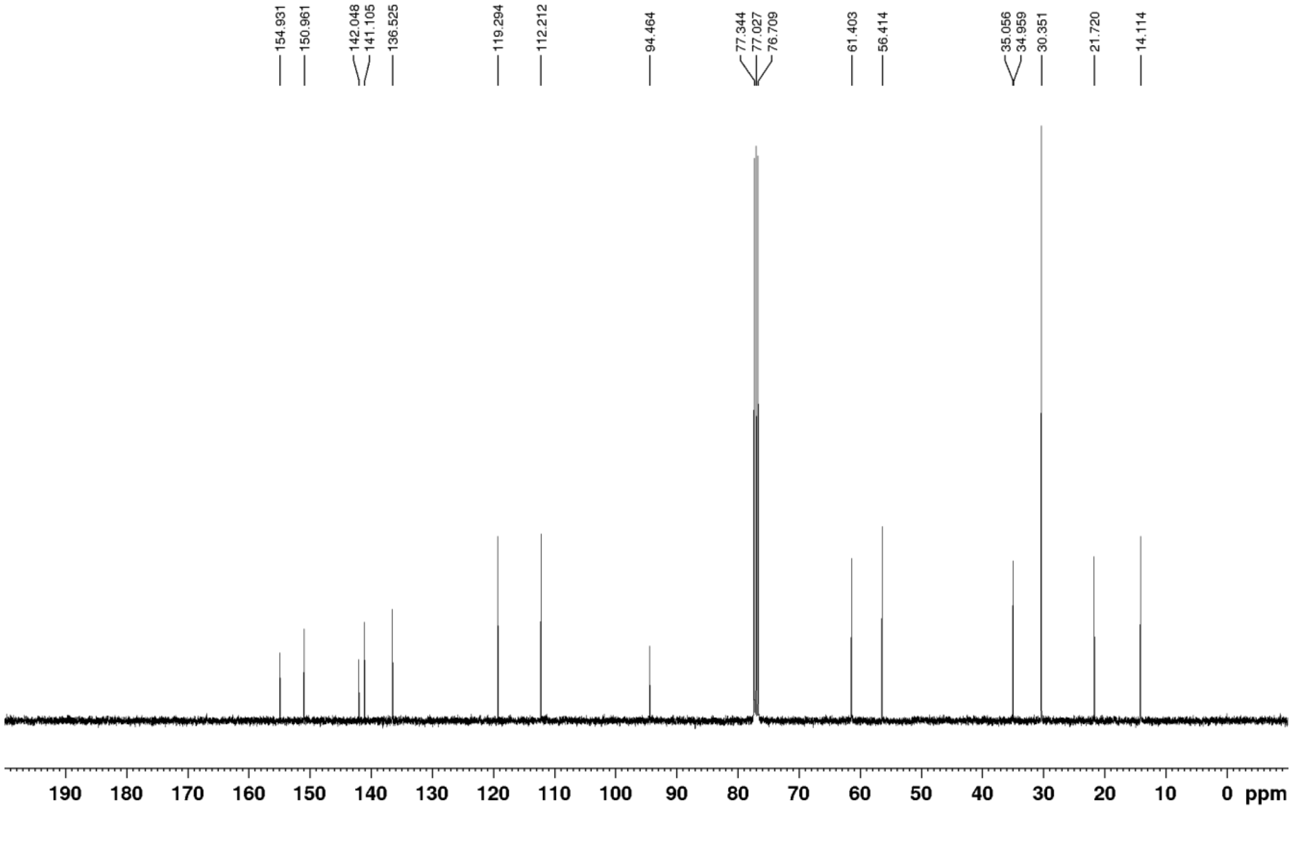

tert-Butyl 3-(2-(tert-butyl)-6-methoxy-4-methylphenoxy)propiolate (2g)

^{1}H NMR ($CDCl_3$, 400 MHz)

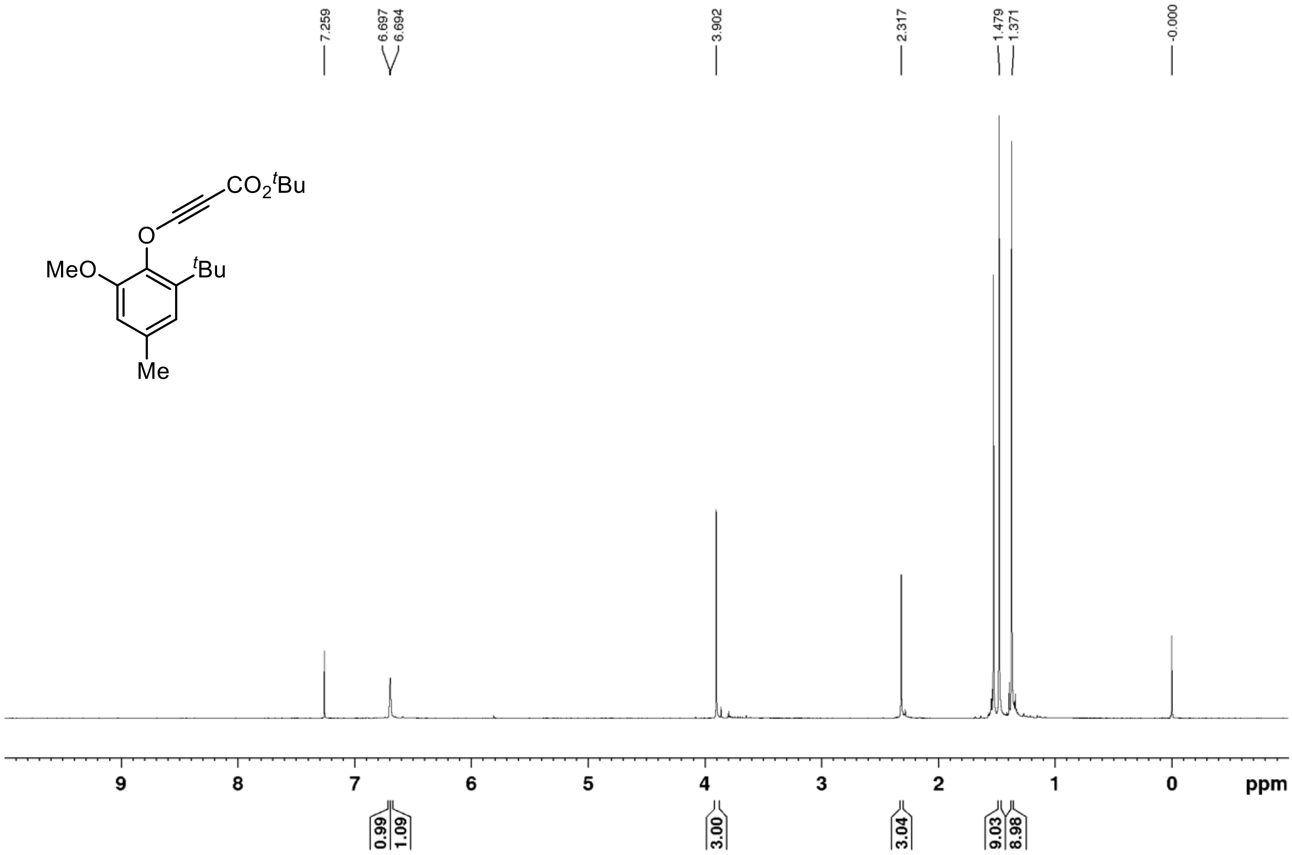

^{13}C NMR ($CDCl_3$, 101 MHz)

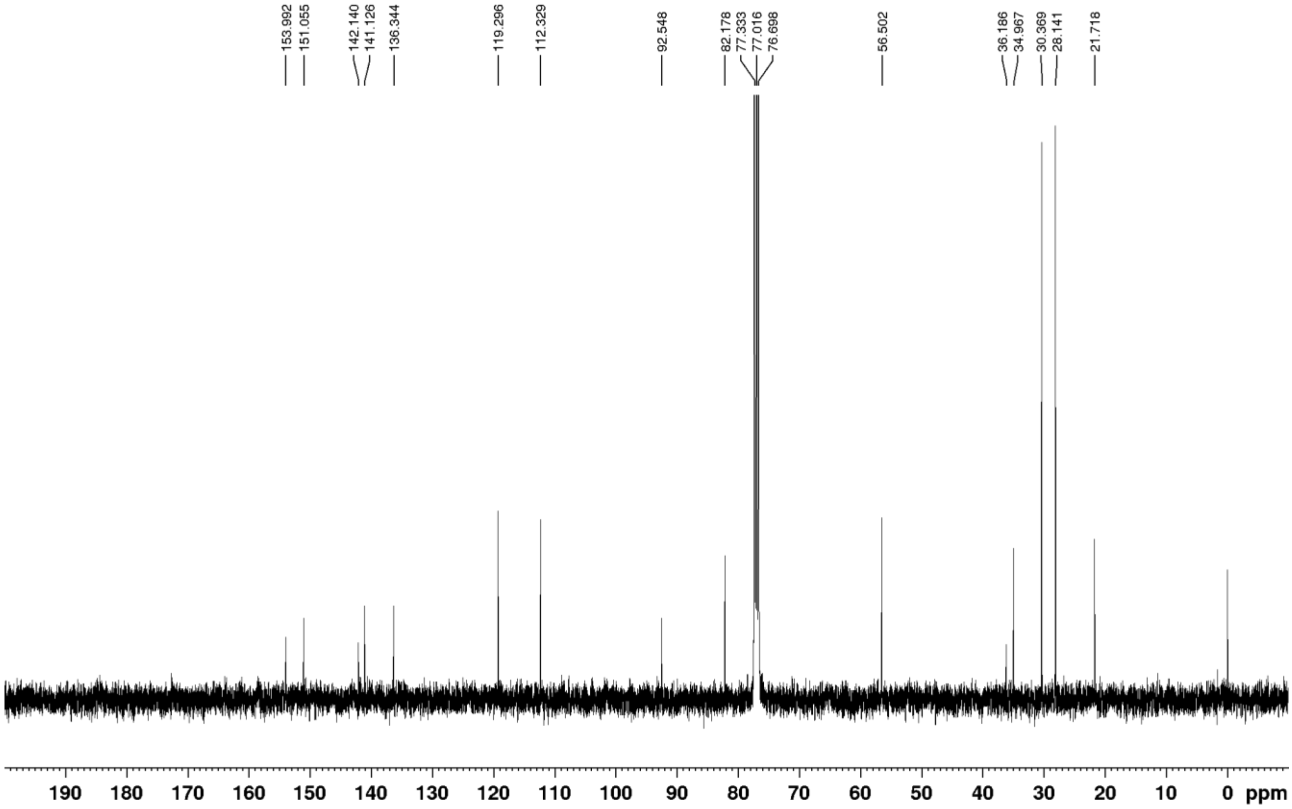

4-(2-(*tert*-Butyl)-6-methoxy-4-methylphenoxy)but-3-yn-2-one (2h)

^{1}H NMR ($CDCl_3$, 400 MHz)

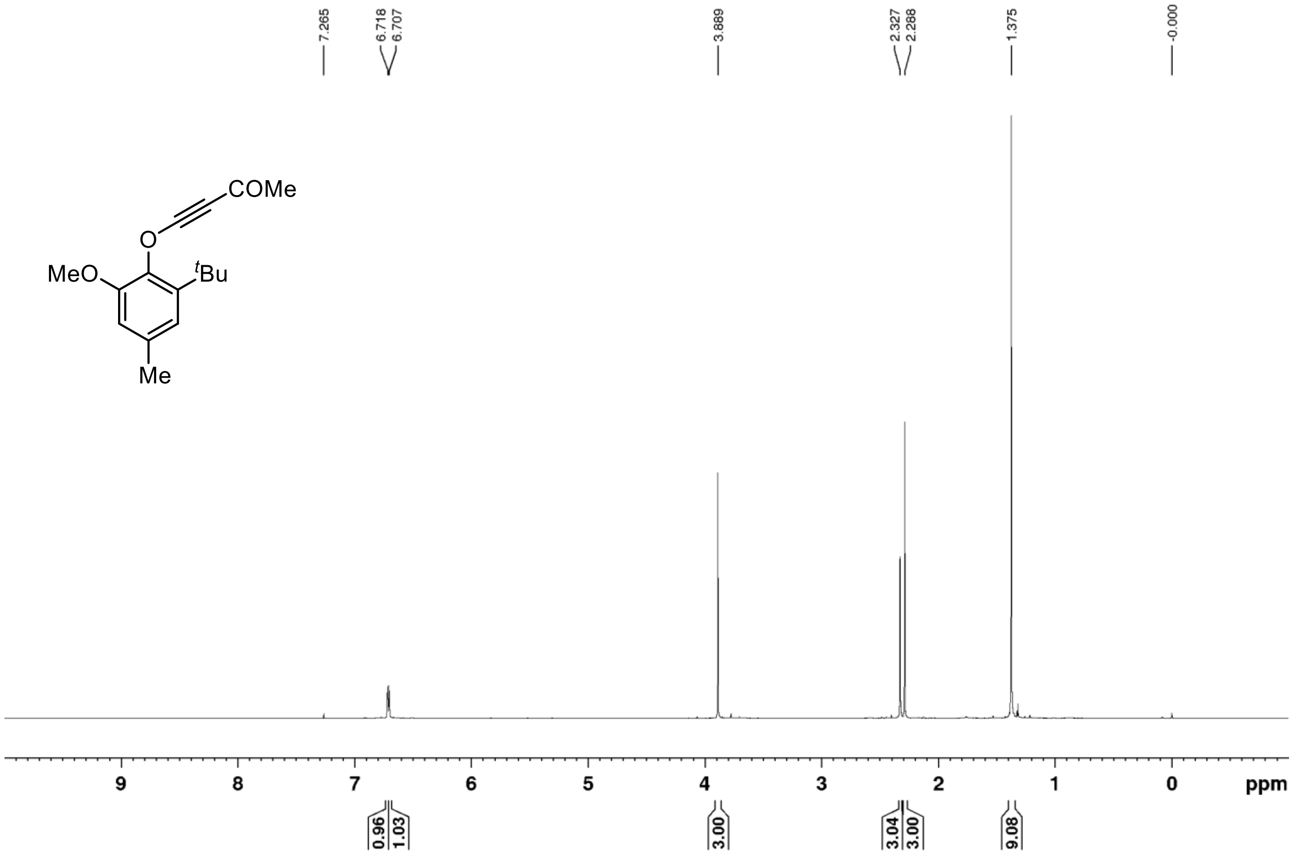

^{13}C NMR ($CDCl_3$, 101 MHz)

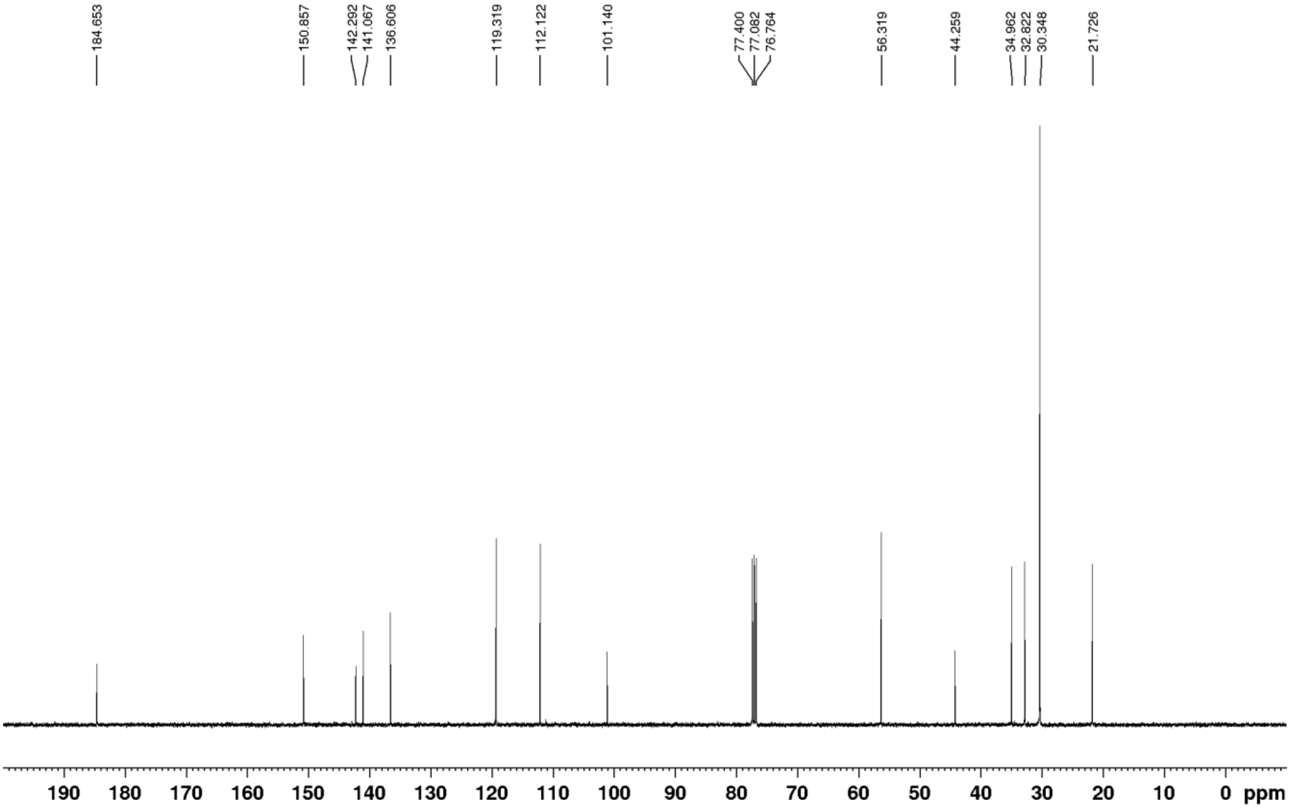

Methyl 3-(2-((3r,5r,7r)-adamantan-1-yl)-4,6-dimethylphenoxy)propiolate (2i)

^{1}H NMR ($CDCl_3$, 400 MHz)

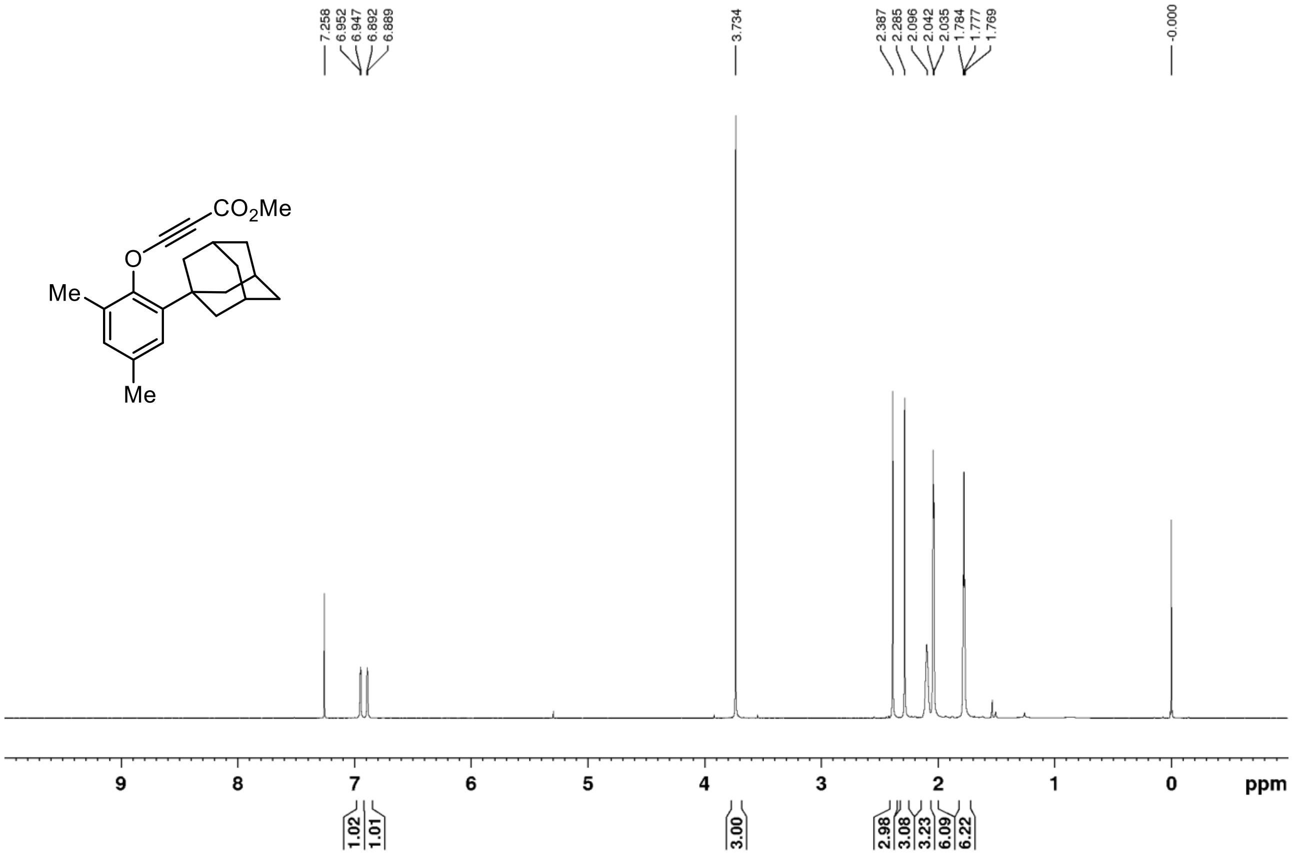

^{13}C NMR ($CDCl_3$, 101 MHz)

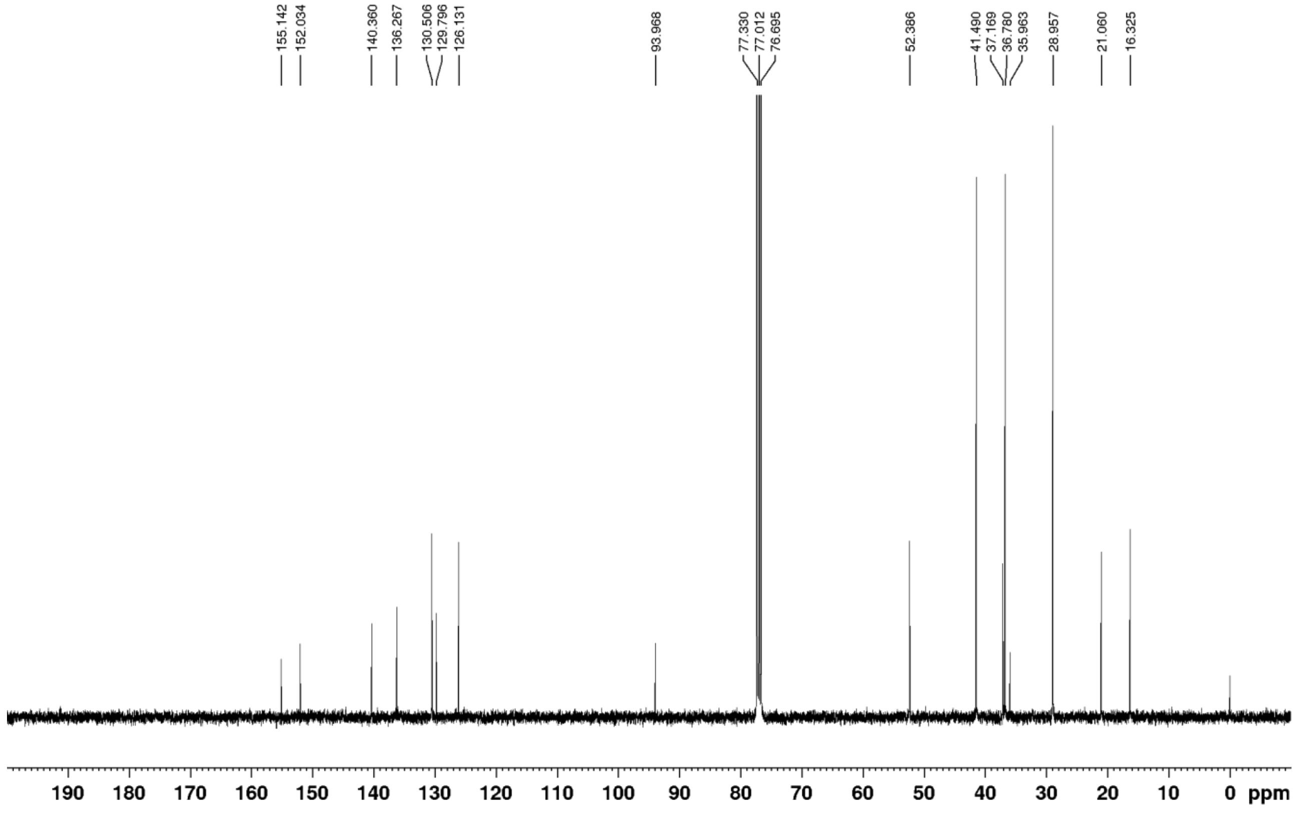

(*E*)-3-(*tert*-Butyl)-2-((1,2-dichlorovinyl)oxy)-4'-methoxy-5-methyl-1,1'-biphenyl (S3j)

^{1}H NMR ($CDCl_3$, 400 MHz)

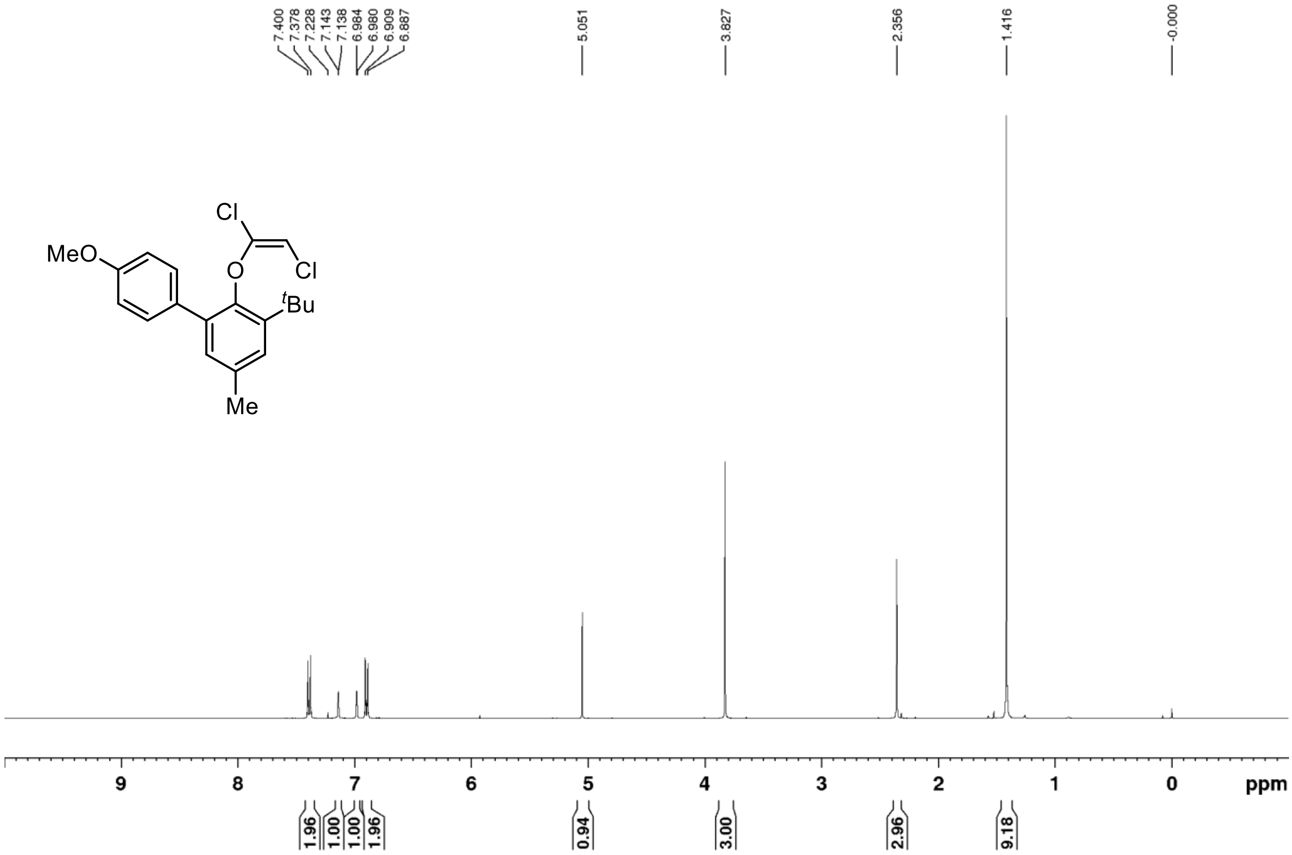

^{13}C NMR ($CDCl_3$, 101 MHz)

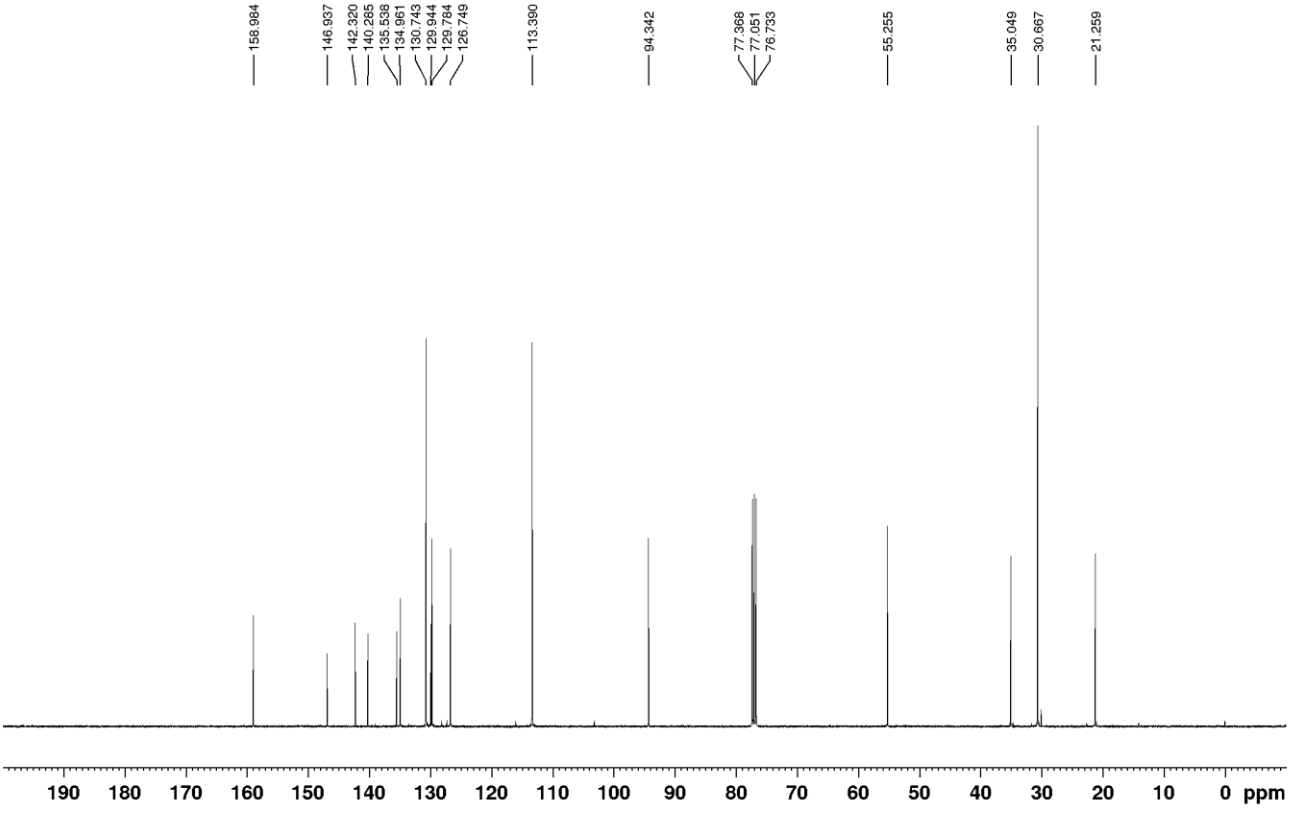

Methyl 3-((3-(*tert*-butyl)-4'-methoxy-5-methyl-[1,1'-biphenyl]-2-yl)oxy)propiolate (2j)

^{1}H NMR ($CDCl_3$, 400 MHz)

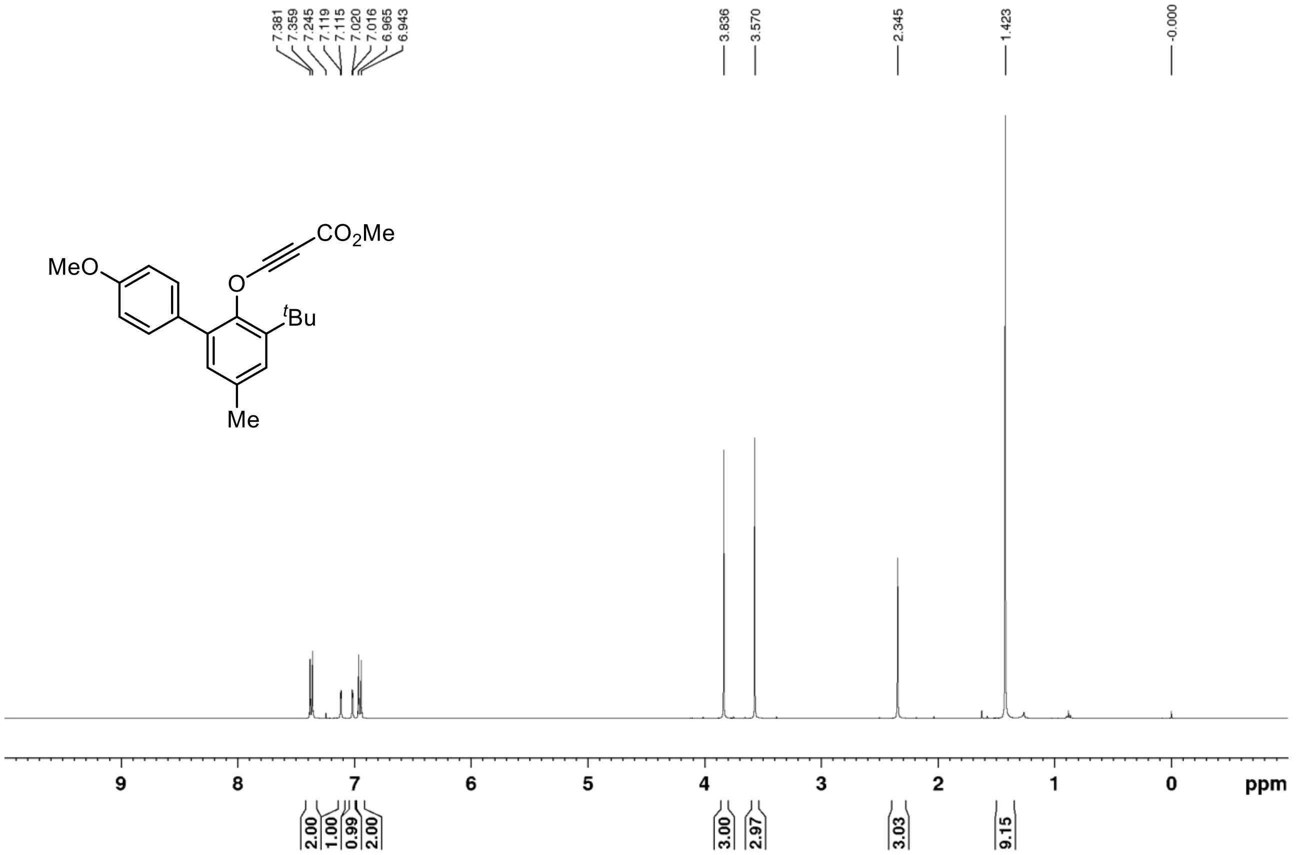

^{13}C NMR ($CDCl_3$, 101 MHz)

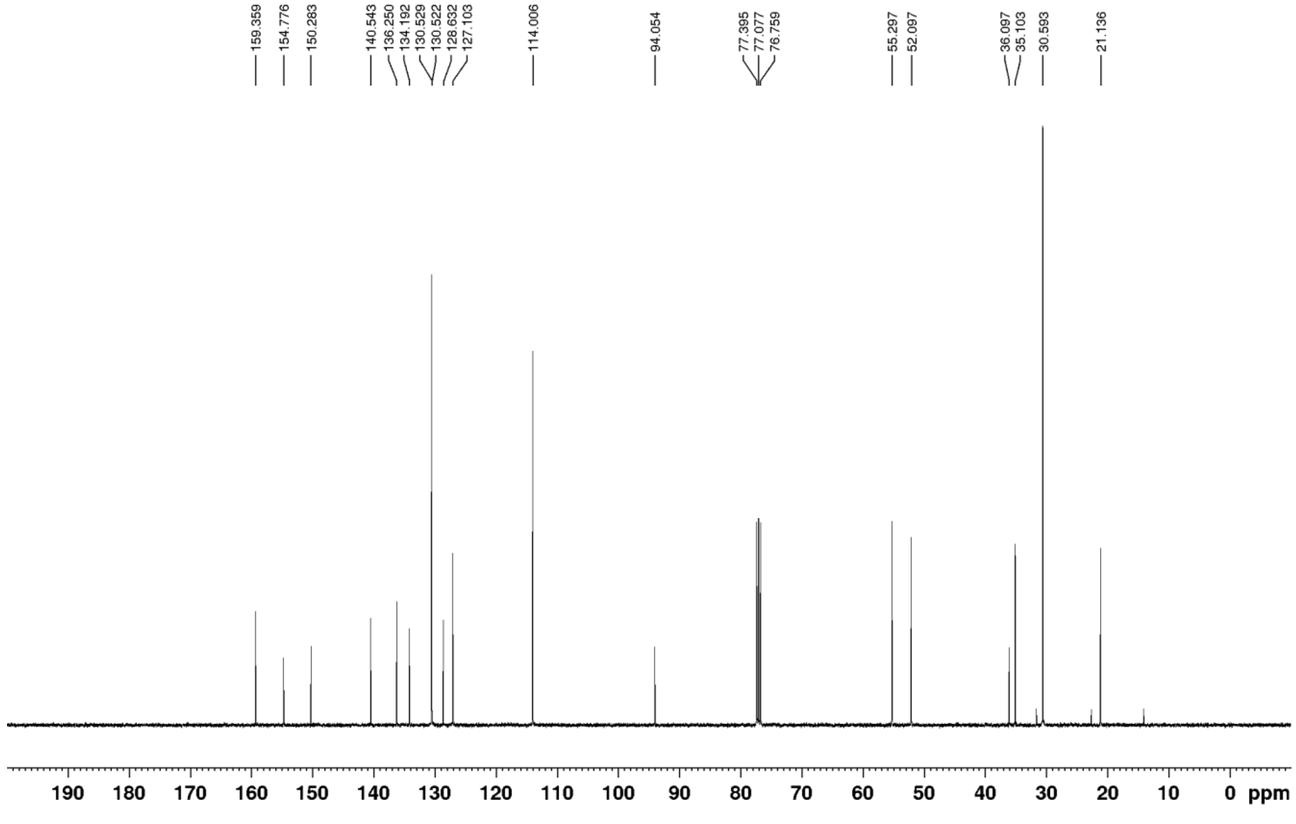

(*E*)-3-(*tert*-Butyl)-2-((1,2-dichlorovinyl)oxy)-5-methyl-4'-(trifluoromethyl)-1,1'-bipheny (S3k)

^{1}H NMR ($CDCl_3$, 400 MHz)

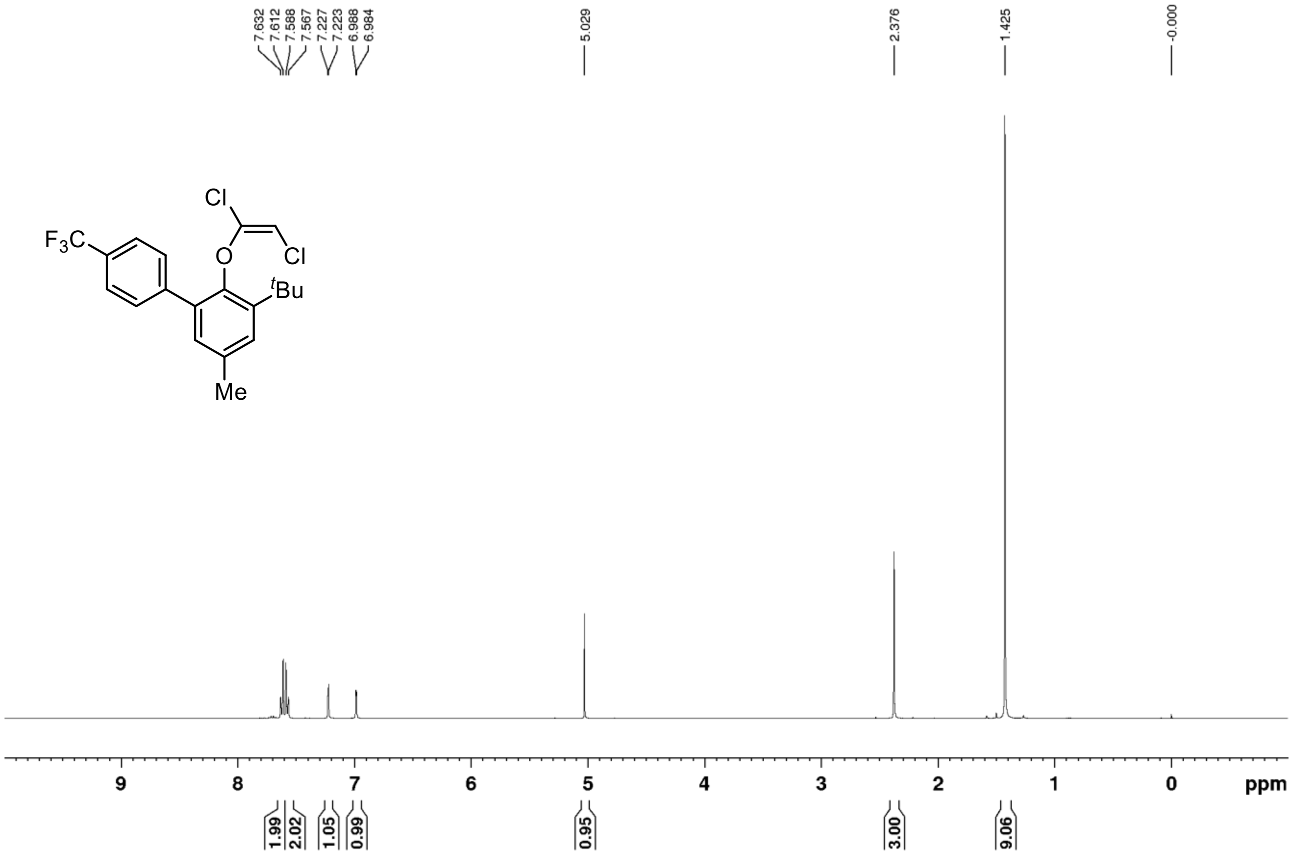

^{13}C NMR ($CDCl_3$, 101 MHz)

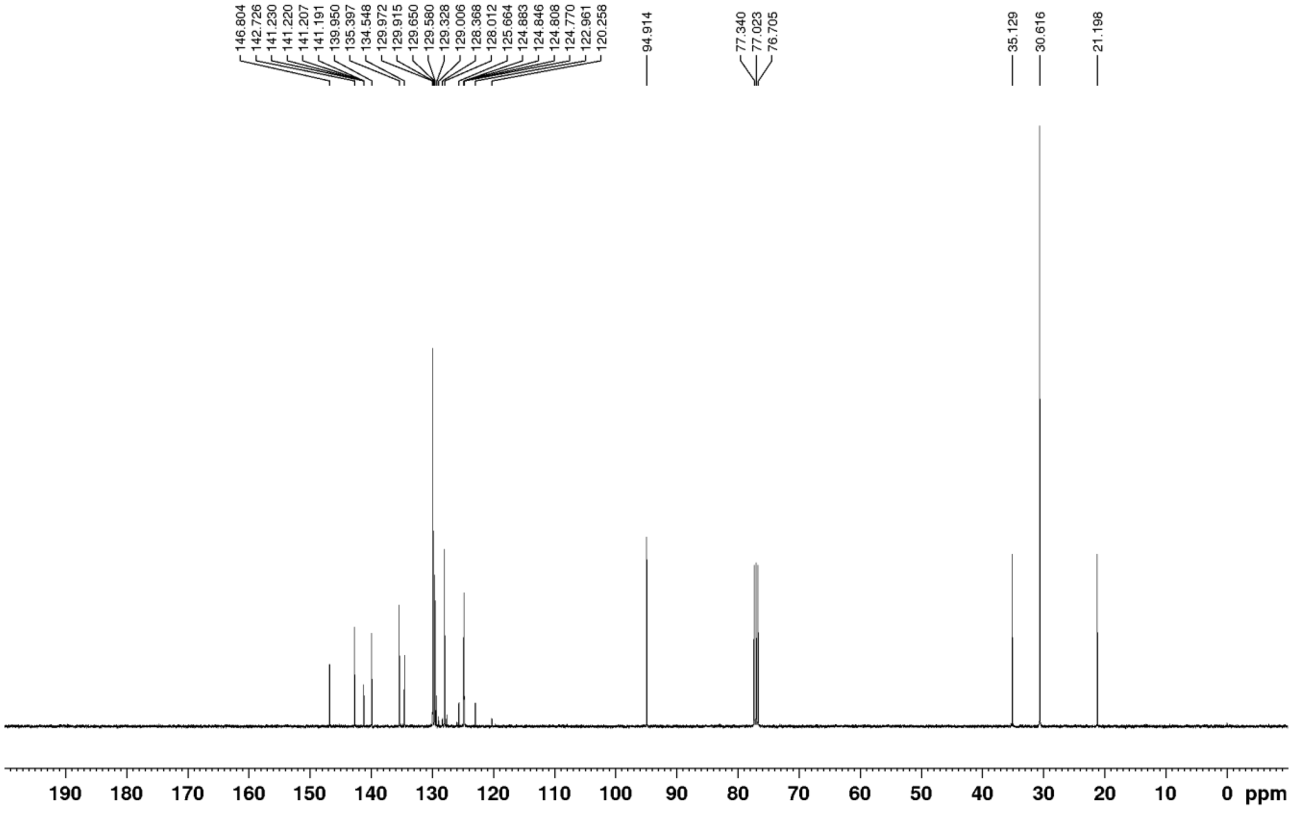

^{19}F NMR ($CDCl_3$, 377 MHz)

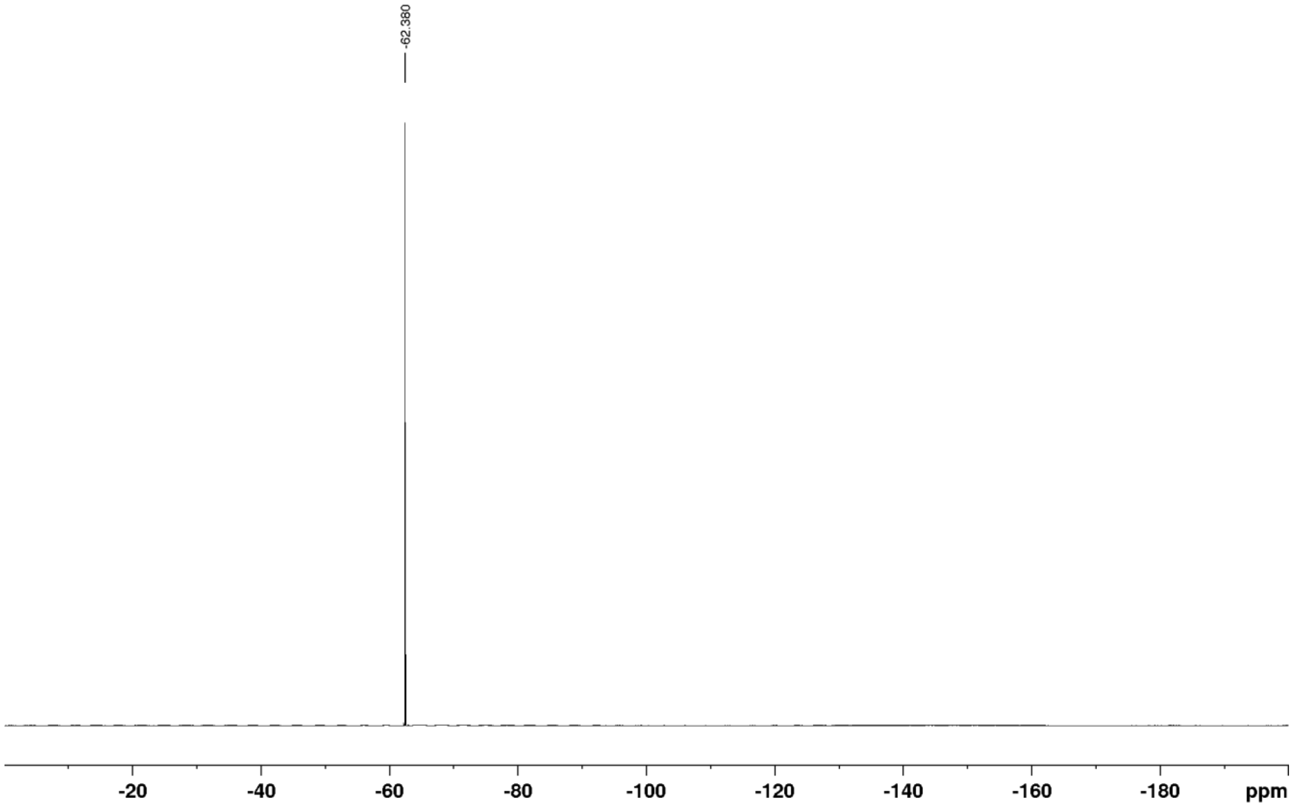

Methyl 3-((3-(*tert*-butyl)-5-methyl-4'-(trifluoromethyl)-[1,1'-biphenyl]-2-yl)oxy)propiolate (2k)

^{1}H NMR ($CDCl_3$, 400 MHz)

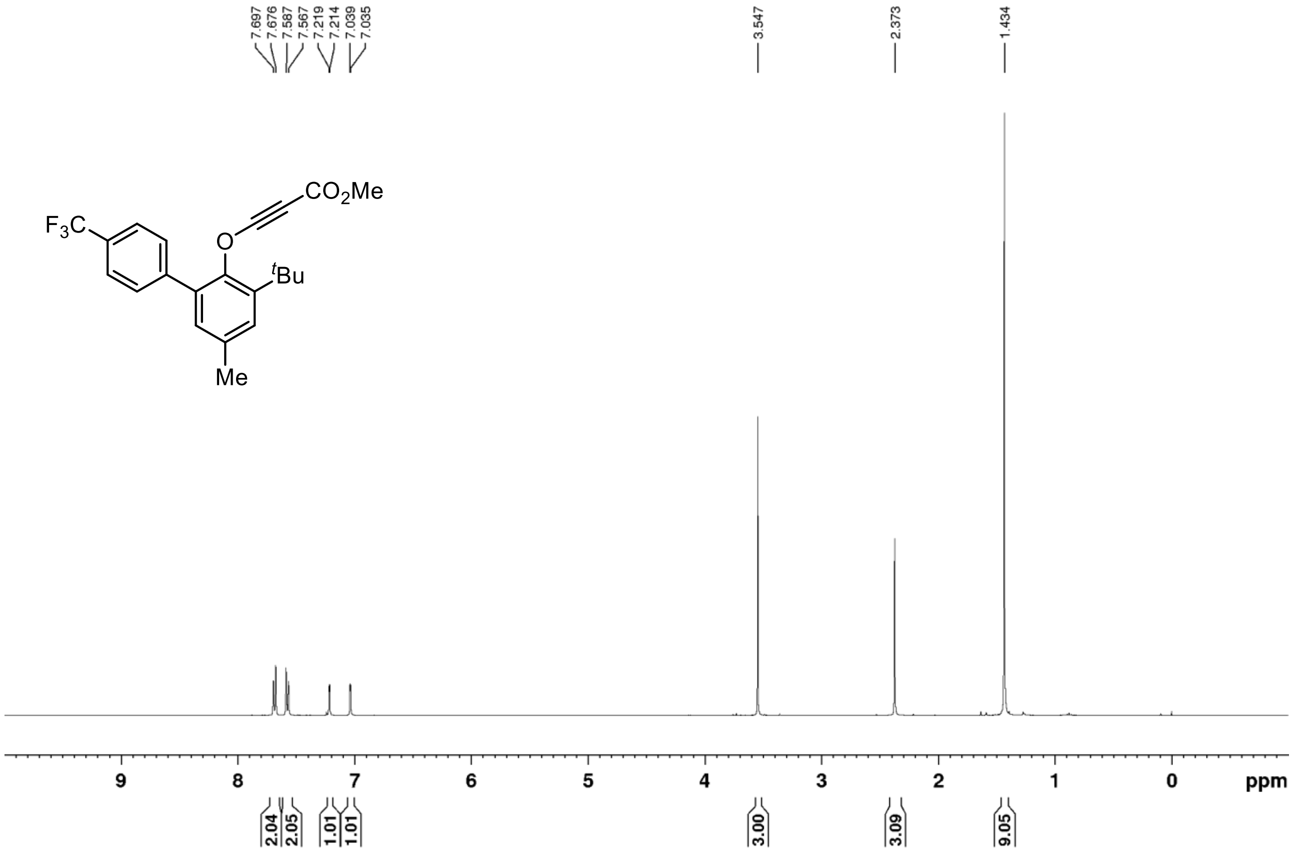

^{13}C NMR ($CDCl_3$, 101 MHz)

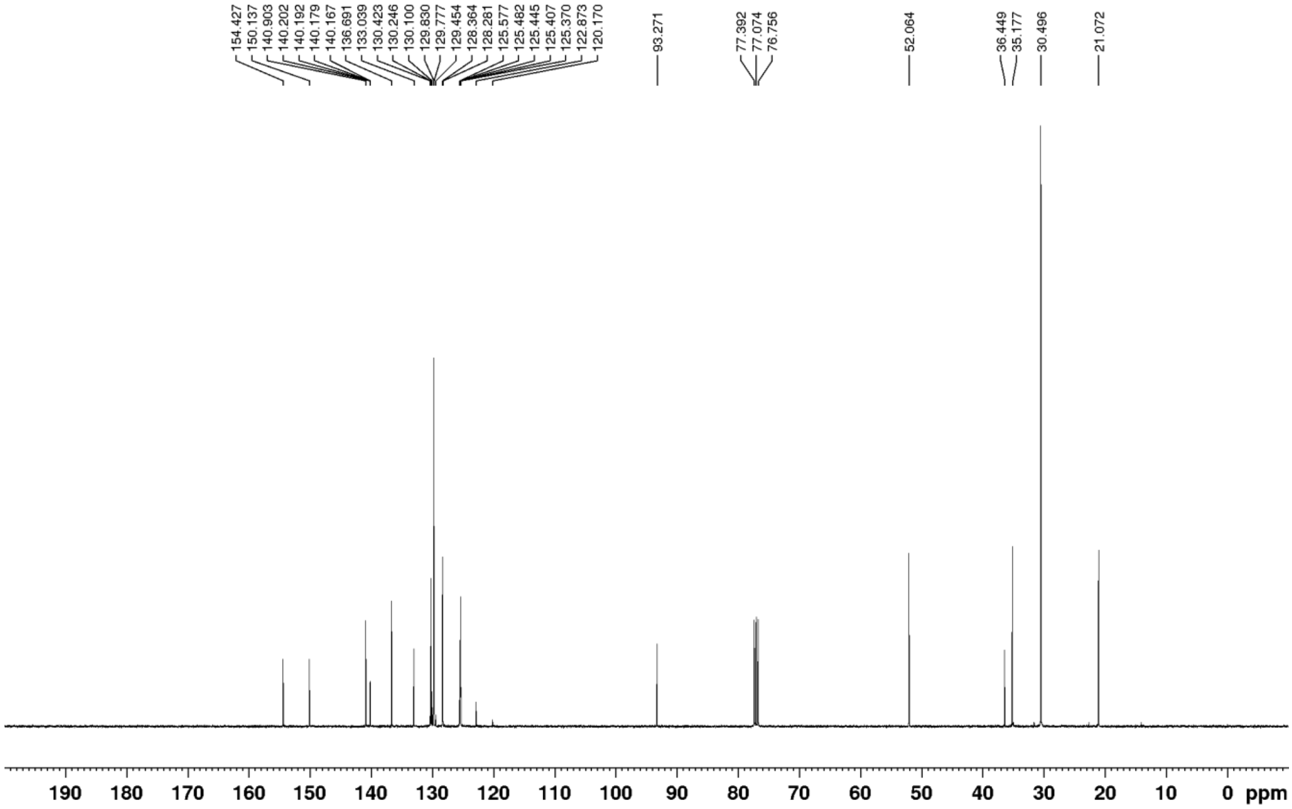

^{19}F NMR ($CDCl_3$, 377 MHz)

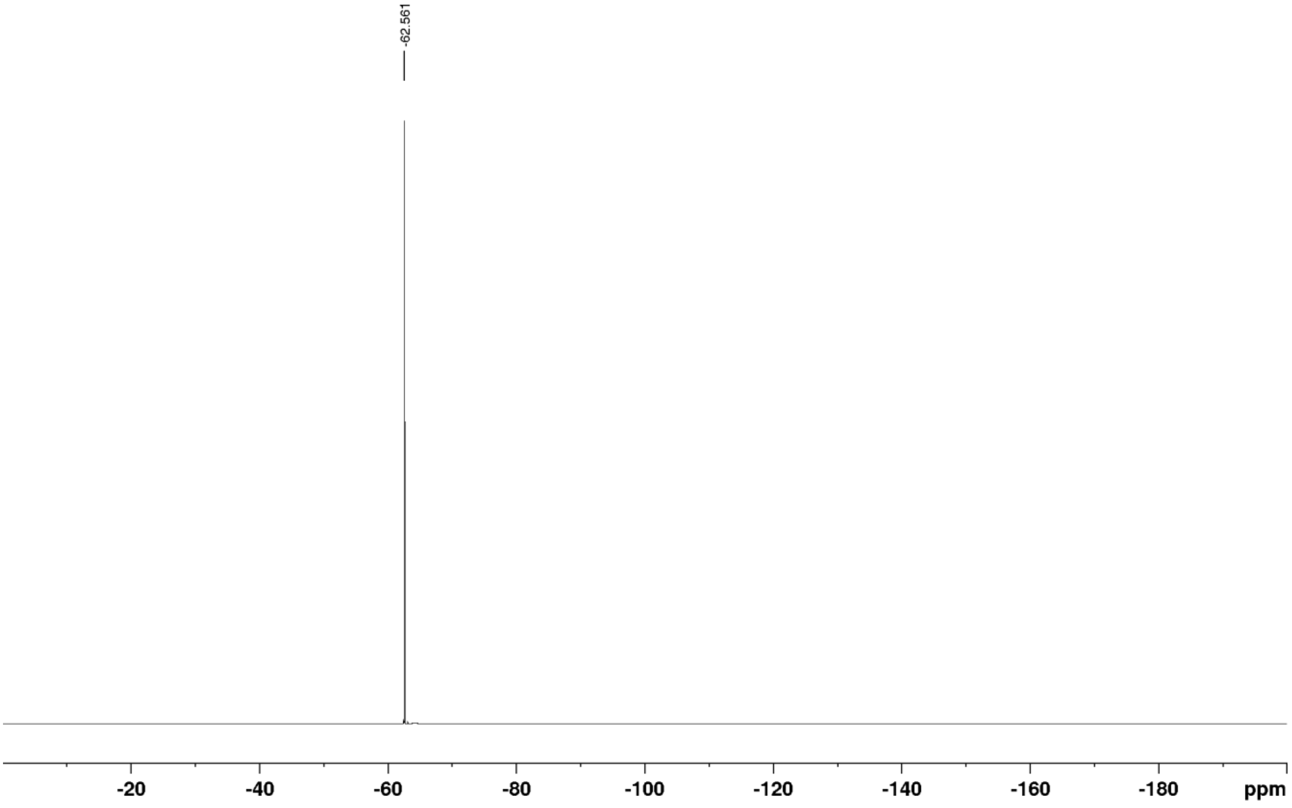

3-(*tert*-Butyl)-2',5-dimethyl-[1,1'-biphenyl]-2-ol (S2l)

^{1}H NMR ($CDCl_3$, 400 MHz)

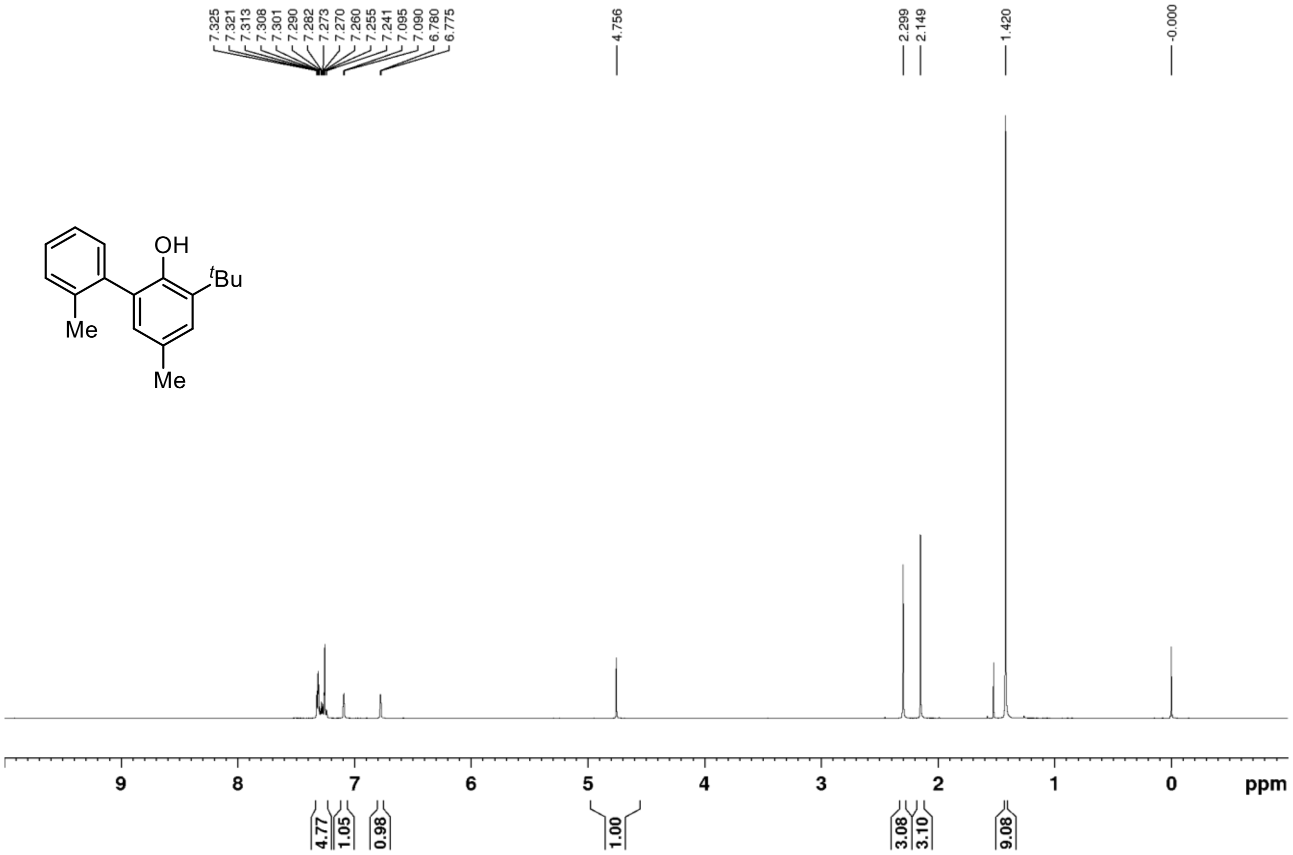

^{13}C NMR ($CDCl_3$, 101 MHz)

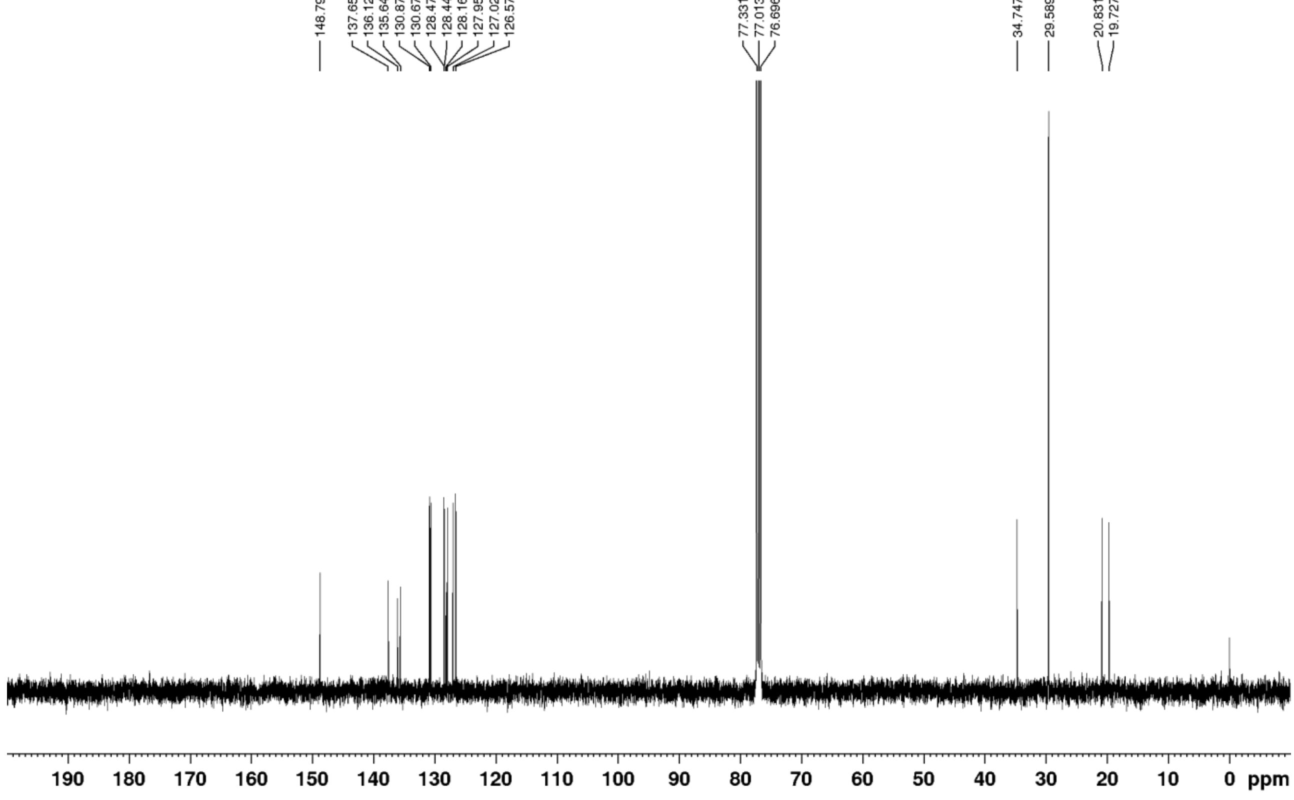

(*E*)-3-(*tert*-Butyl)-2-((1,2-dichlorovinyl)oxy)-2',5-dimethyl-1,1'-biphenyl (S3l)

^{1}H NMR ($CDCl_3$, 400 MHz)

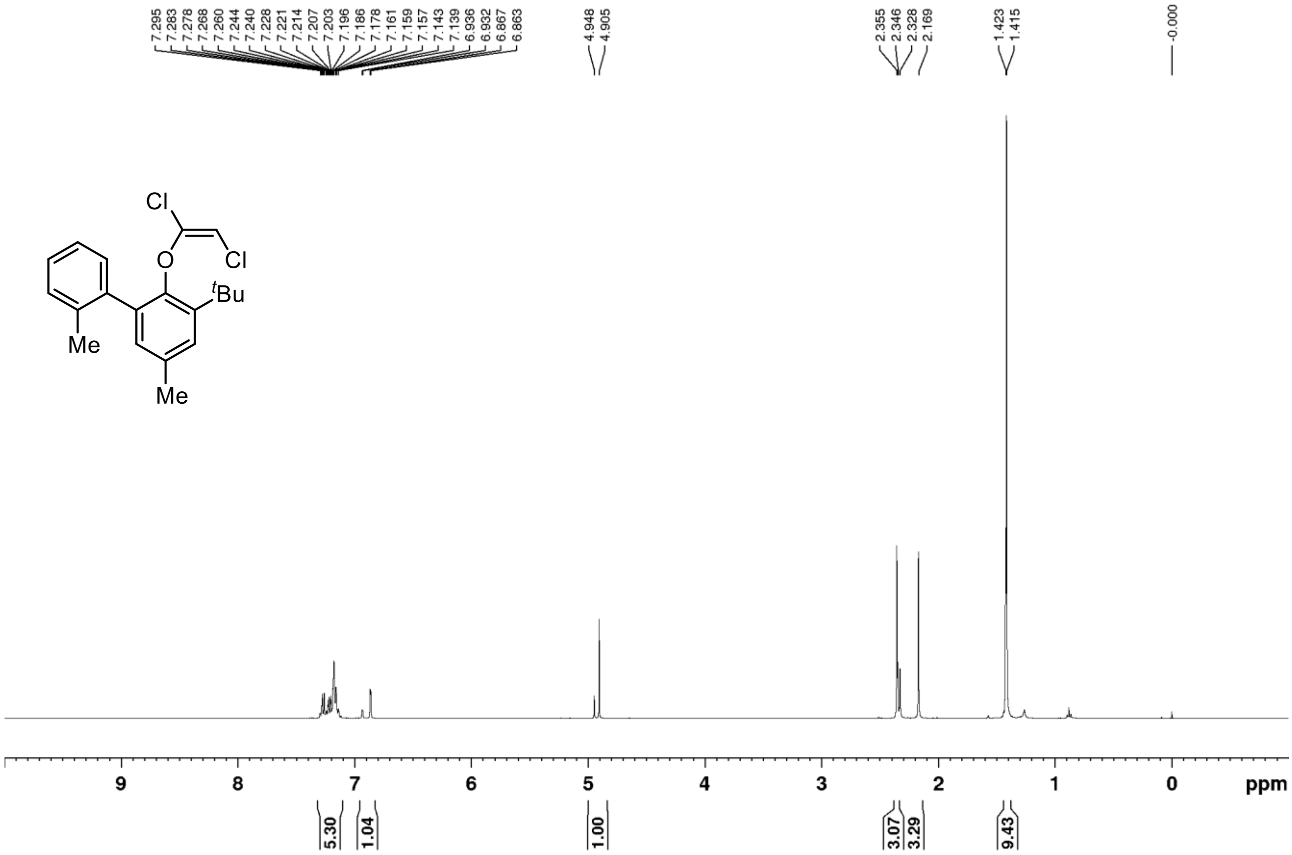

^{13}C NMR ($CDCl_3$, 101 MHz)

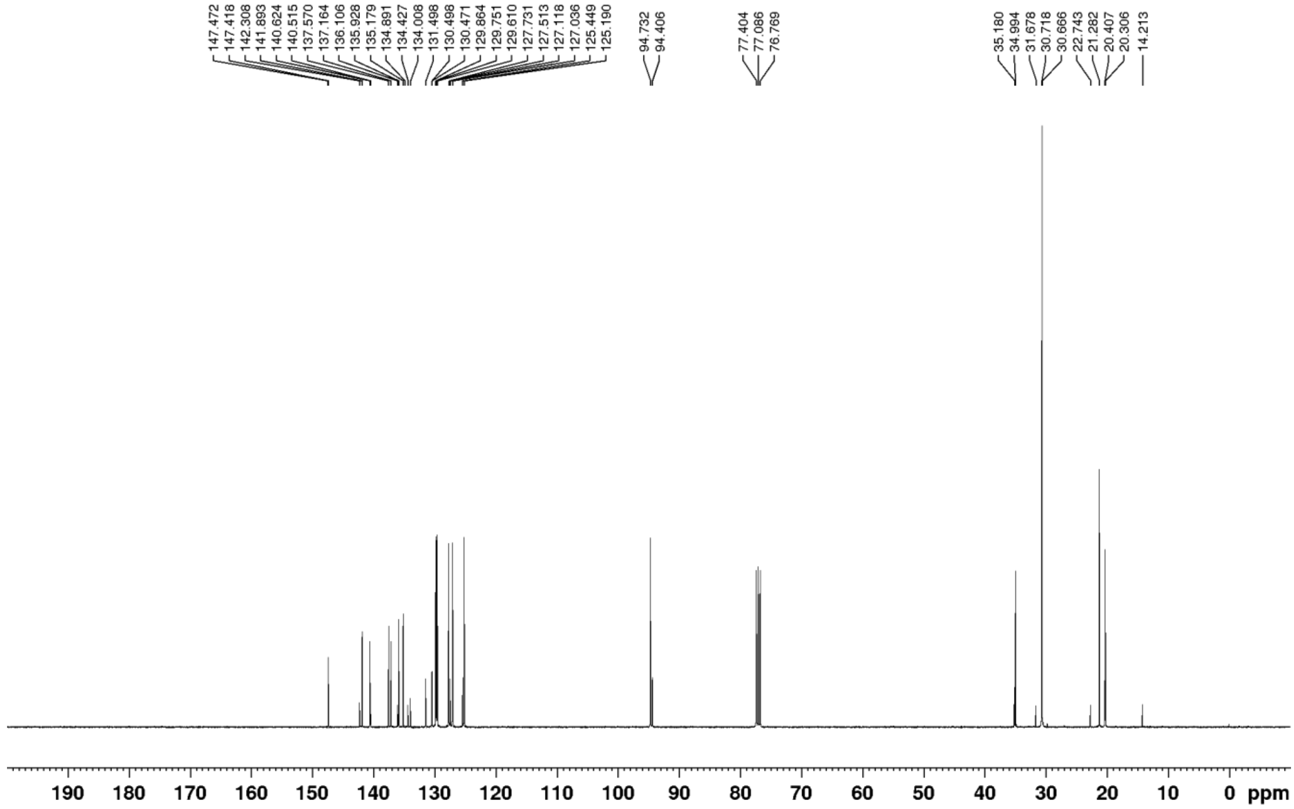

Methyl 3-((3-(*tert*-butyl)-2',5-dimethyl-[1,1'-biphenyl]-2-yl)oxy)propiolate (2l)

^{1}H NMR ($CDCl_3$, 400 MHz)

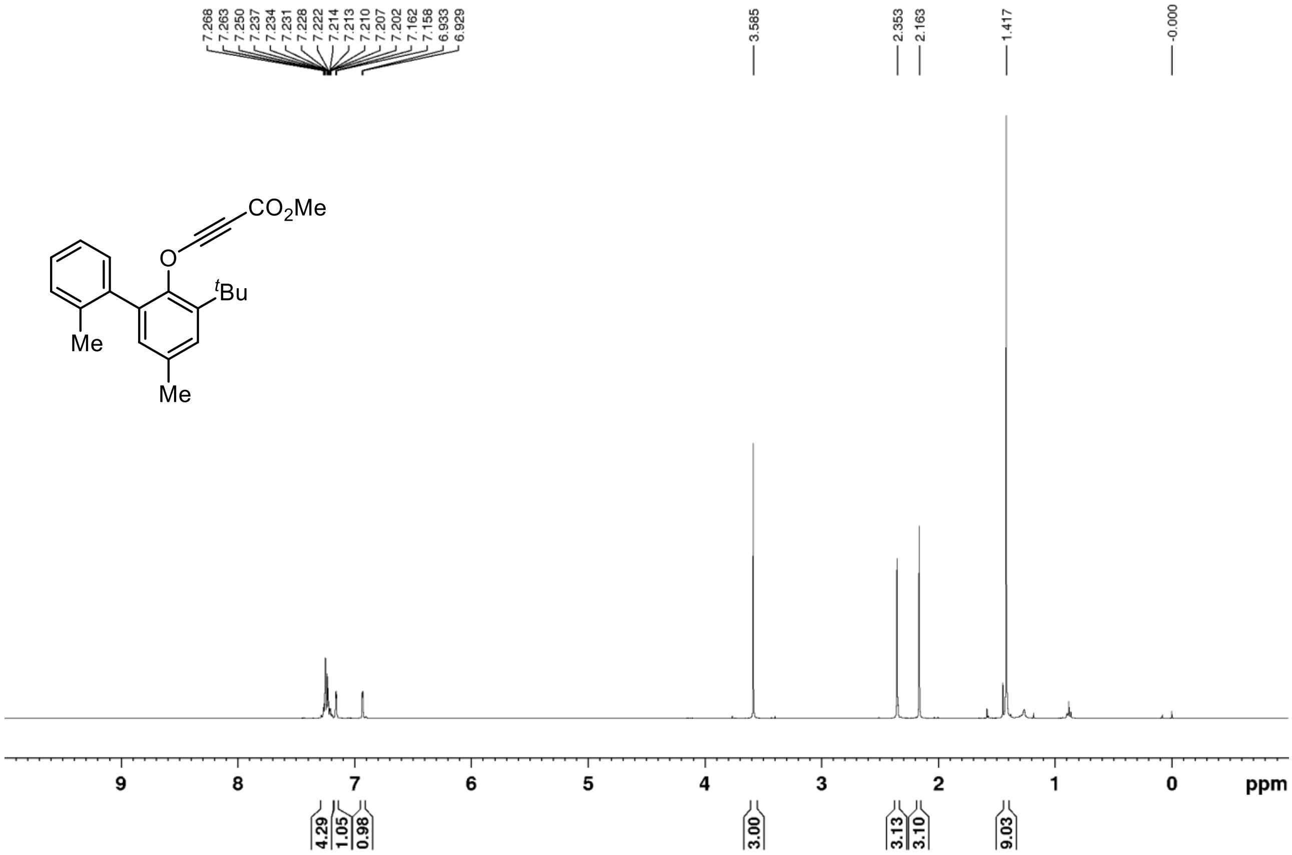

^{13}C NMR ($CDCl_3$, 101 MHz)

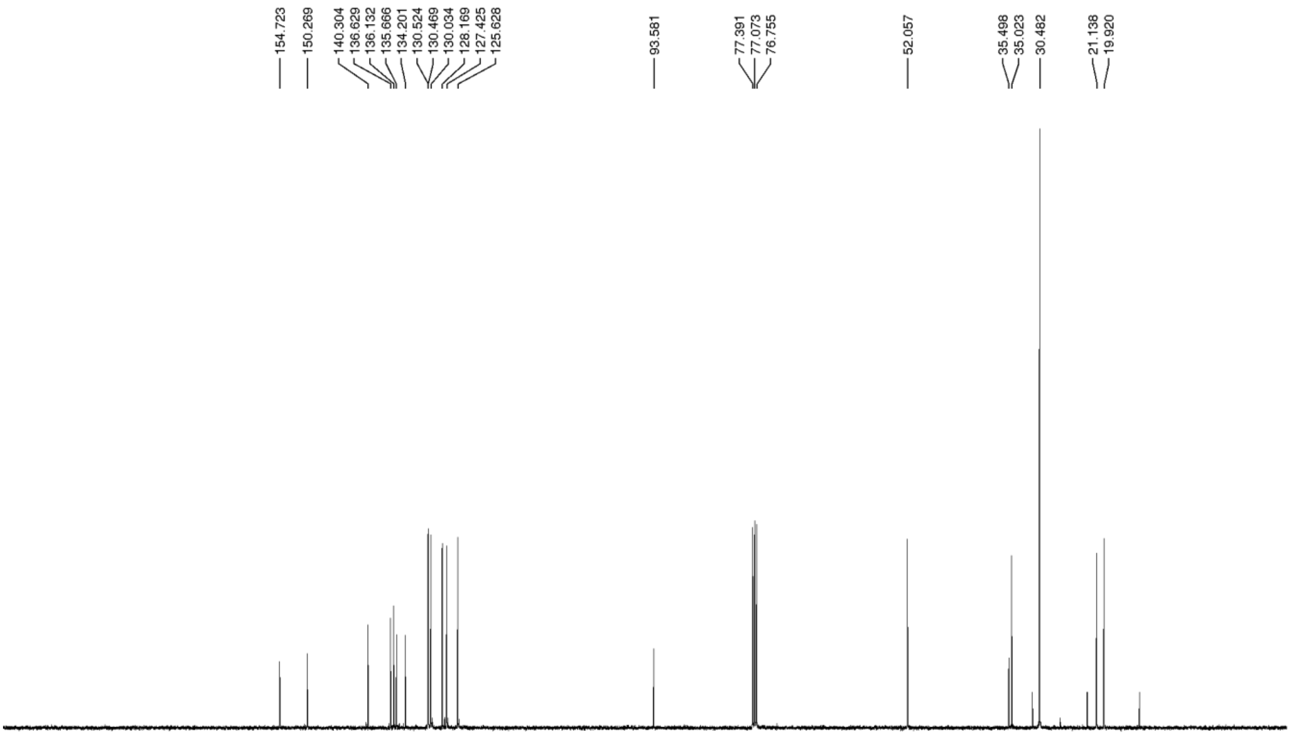

3-(2-(*tert*-Butyl)-6-methoxy-4-methylphenoxy)-*N*,*N*-dimethylpropiolamide (2m)

^{1}H NMR ($CDCl_3$, 400 MHz)

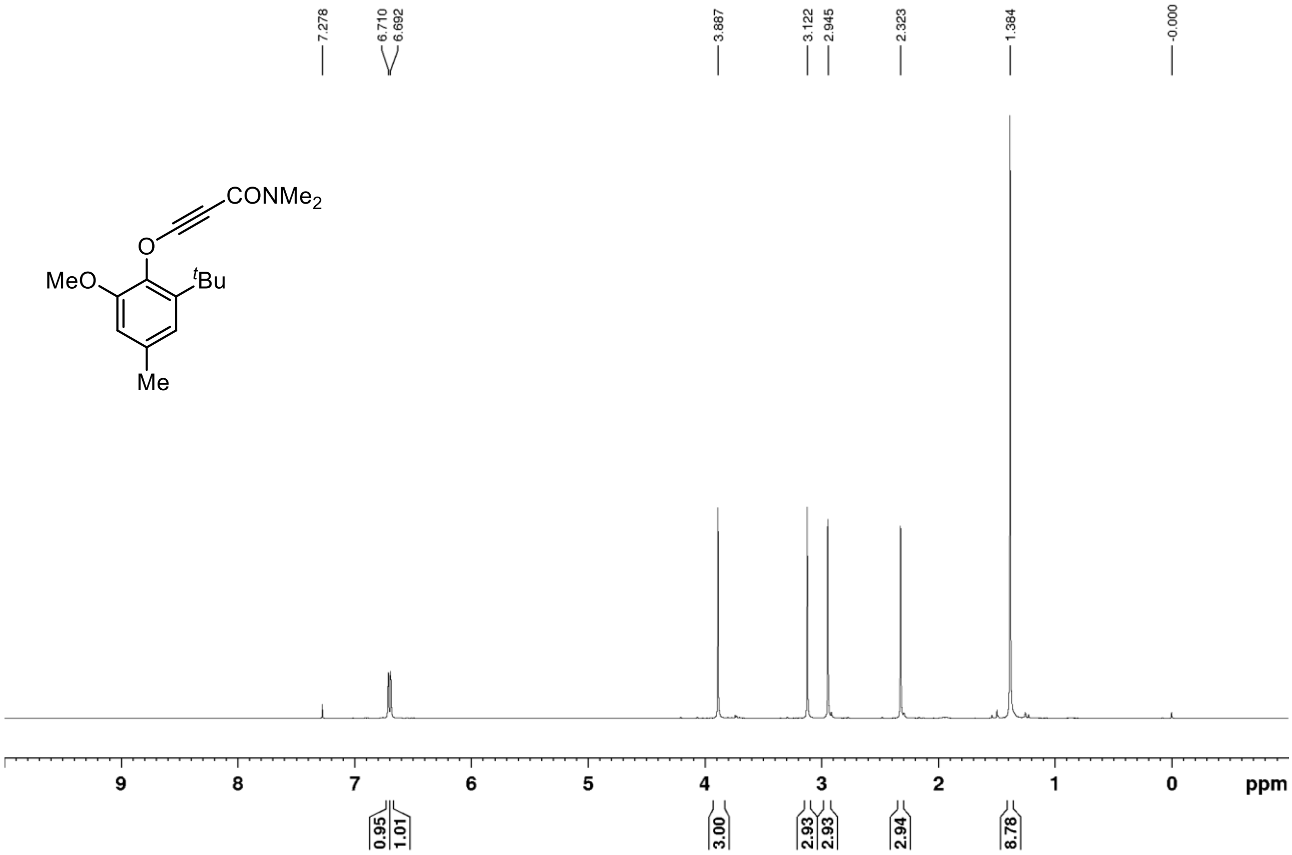

^{13}C NMR ($CDCl_3$, 101 MHz)

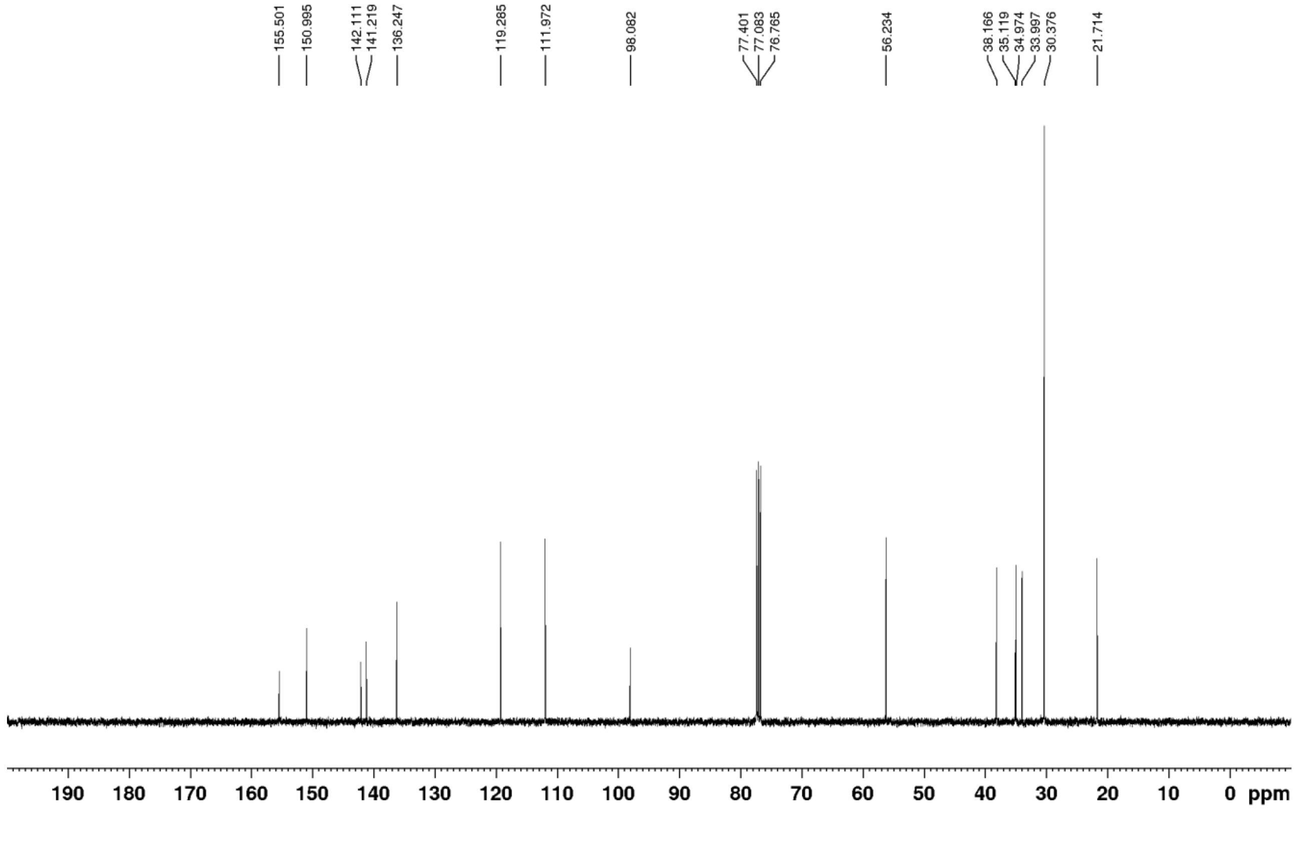

3-(2-(*tert*-Butyl)-6-methylphenoxy)-*N*,*N*-dimethylpropiolamide (2n)

^{1}H NMR ($CDCl_3$, 400 MHz)

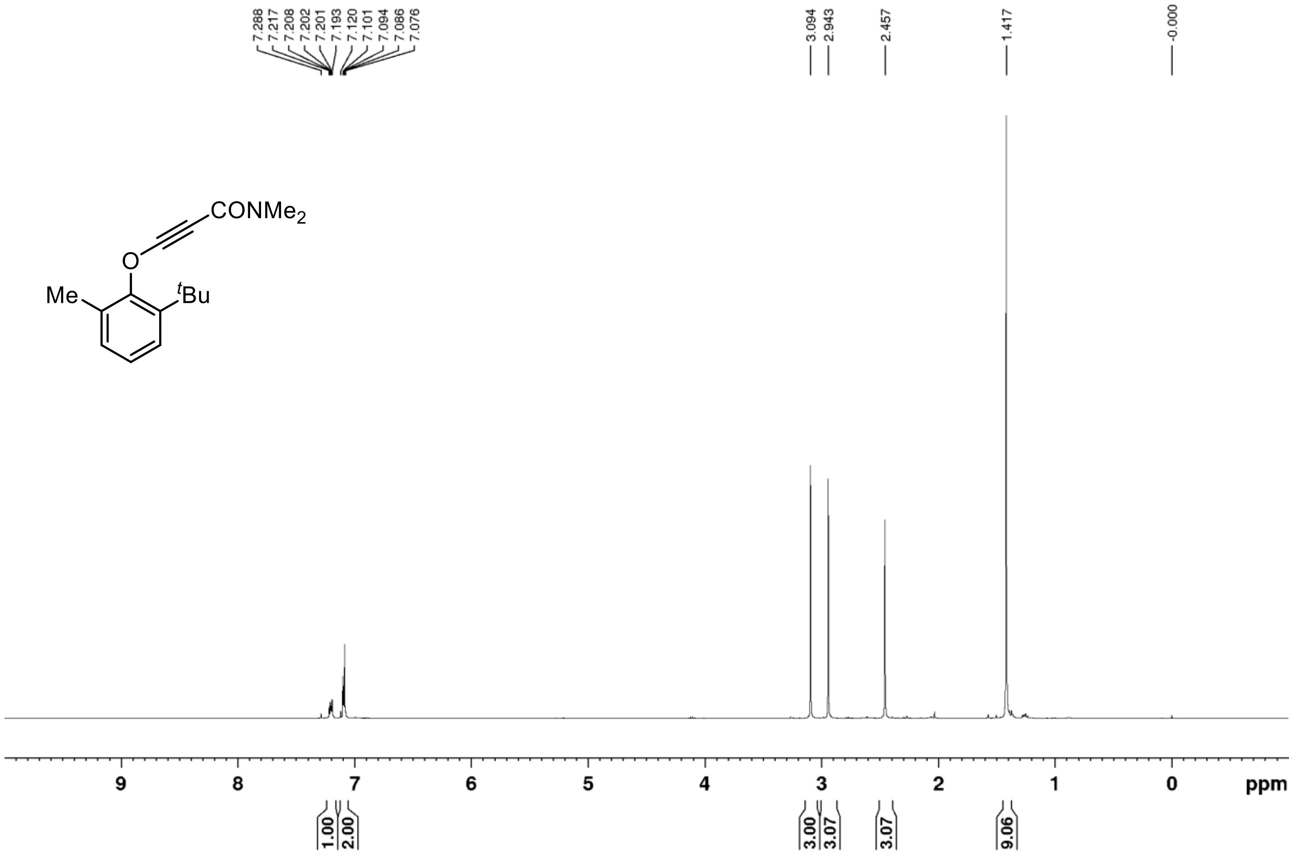

^{13}C NMR ($CDCl_3$, 101 MHz)

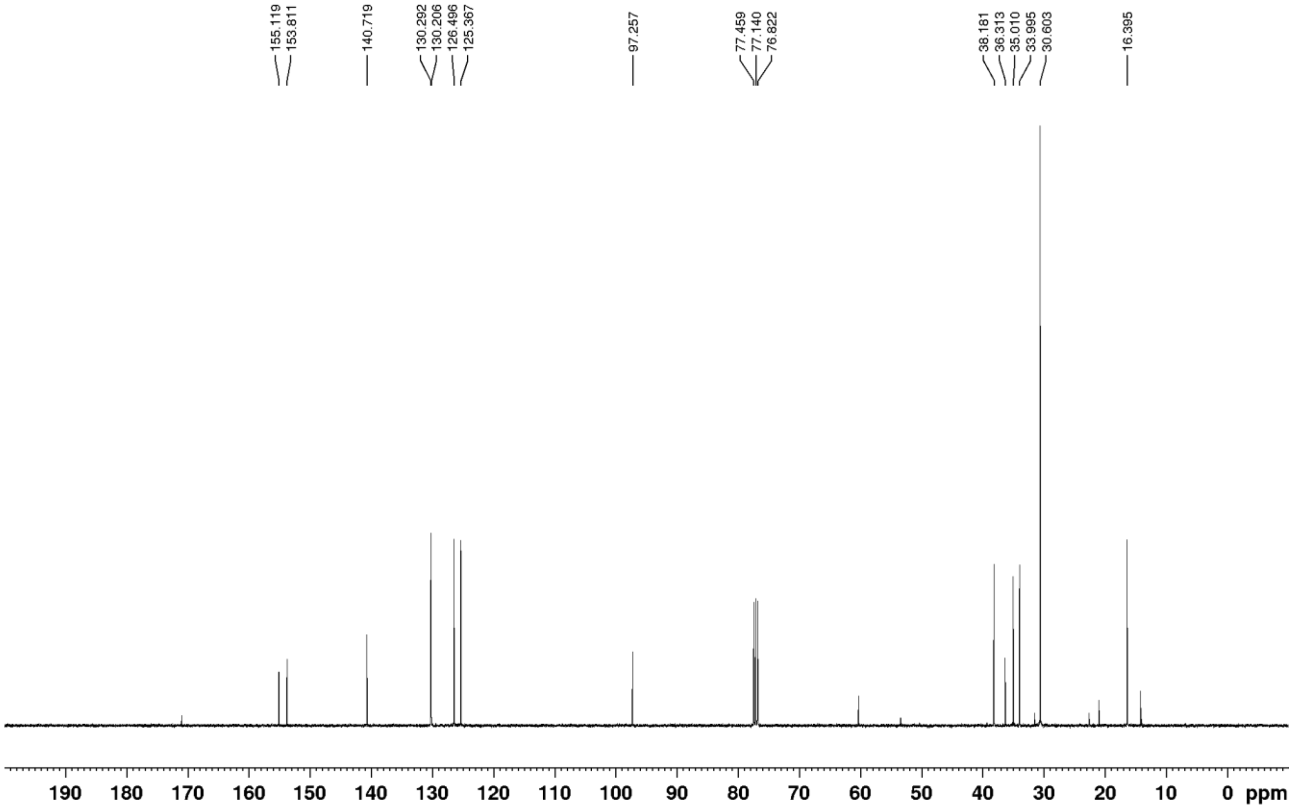

3-((3-(*tert*-Butyl)-4',5-dimethyl-[1,1'-biphenyl]-2-yl)oxy)-*N*,*N*-dimethylpropiolamide (2o)

^{1}H NMR ($CDCl_3$, 400 MHz)

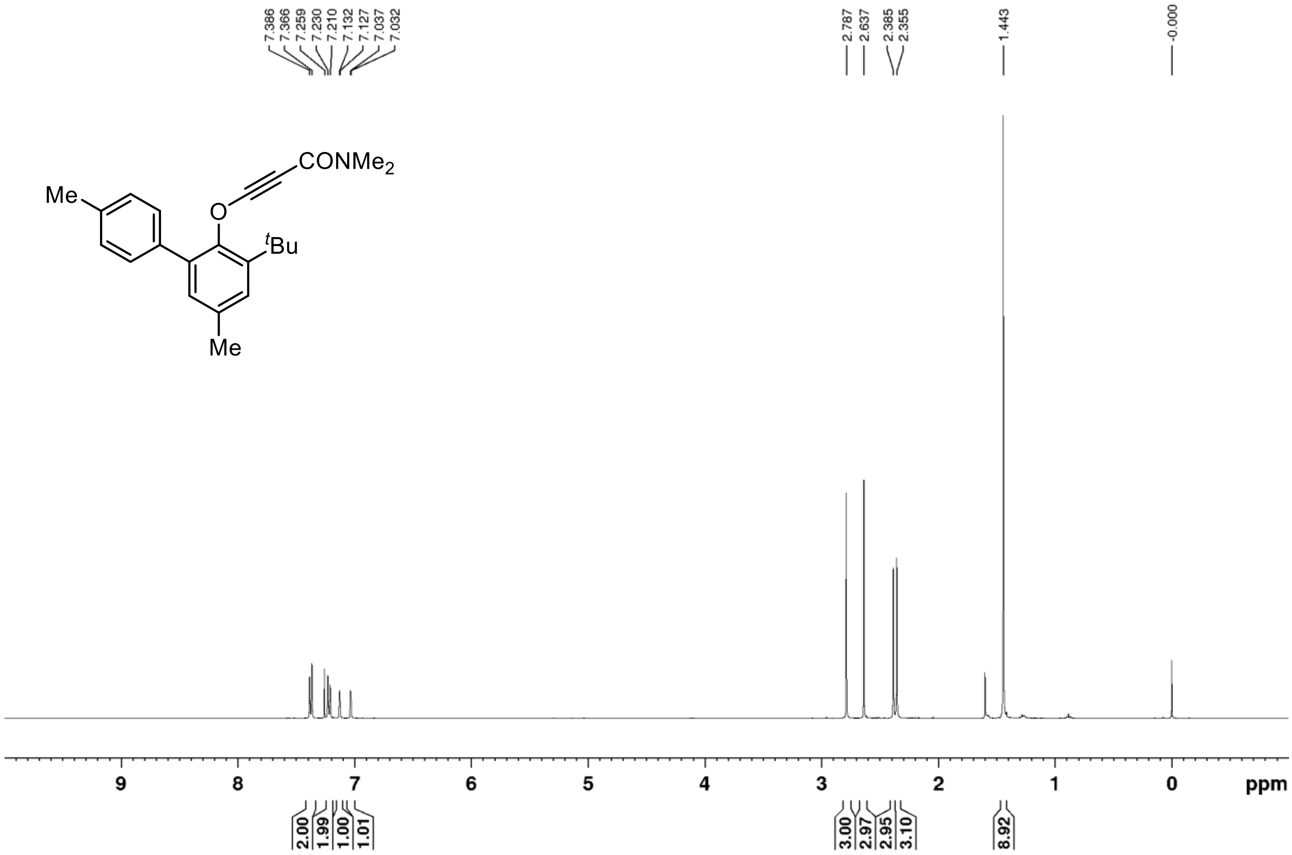

^{13}C NMR ($CDCl_3$, 101 MHz)

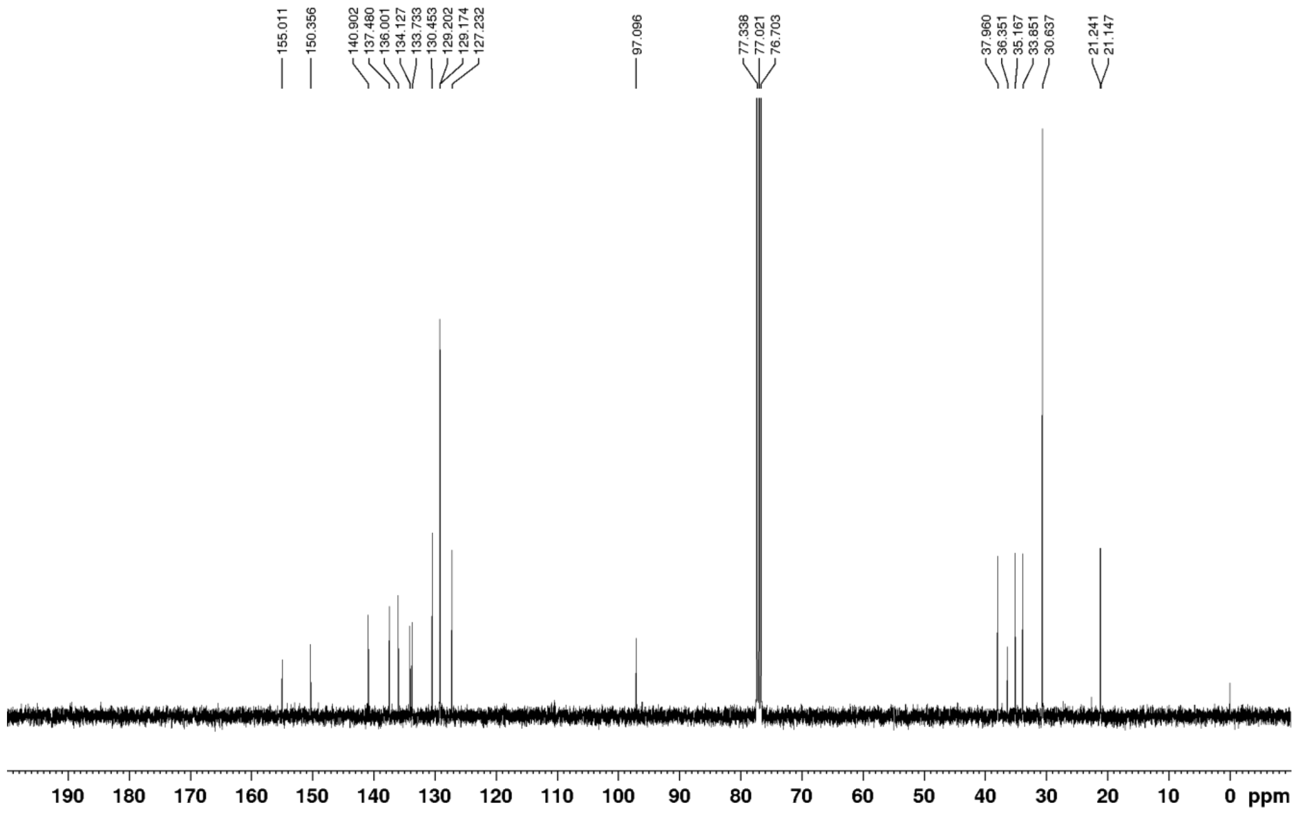

3-(2-(*tert*-Butyl)-6-methylphenoxy)prop-2-yn-1-ol (S6p)

^{1}H NMR (CDCl$_3$, 400 MHz)

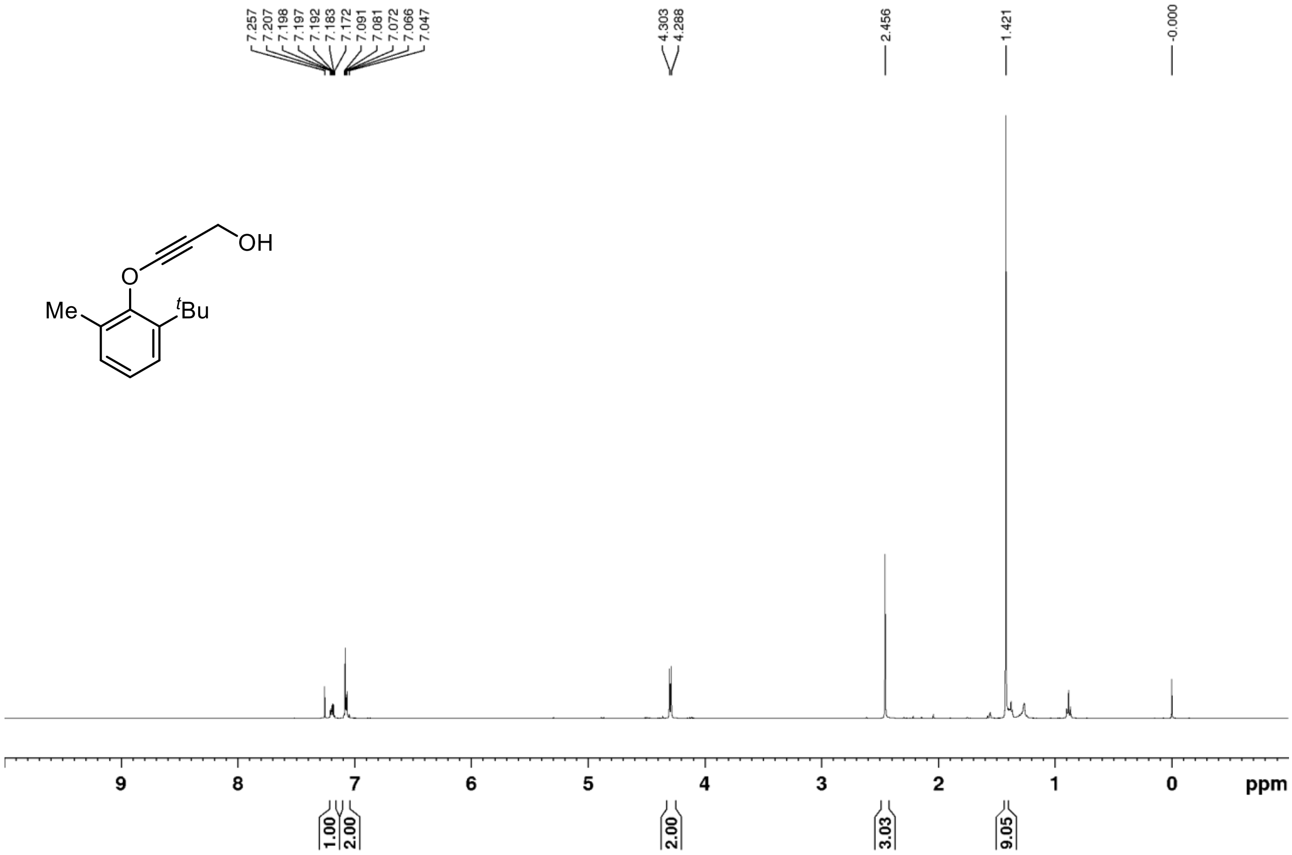

^{13}C NMR (CDCl$_3$, 101 MHz)

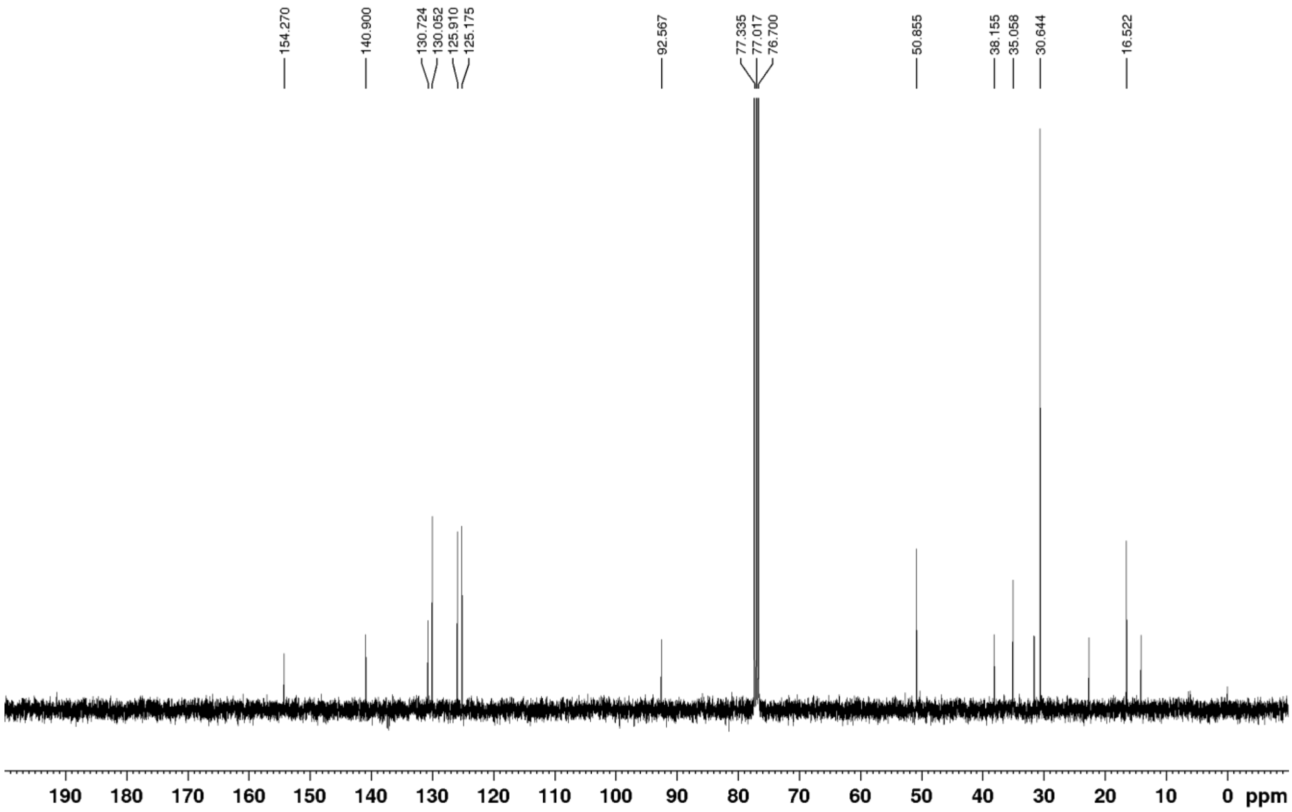

1-(*tert*-Butyl)-2-((3-methoxyprop-1-yn-1-yl)oxy)-3-methylbenzene (2p)

^{1}H NMR ($CDCl_3$, 400 MHz)

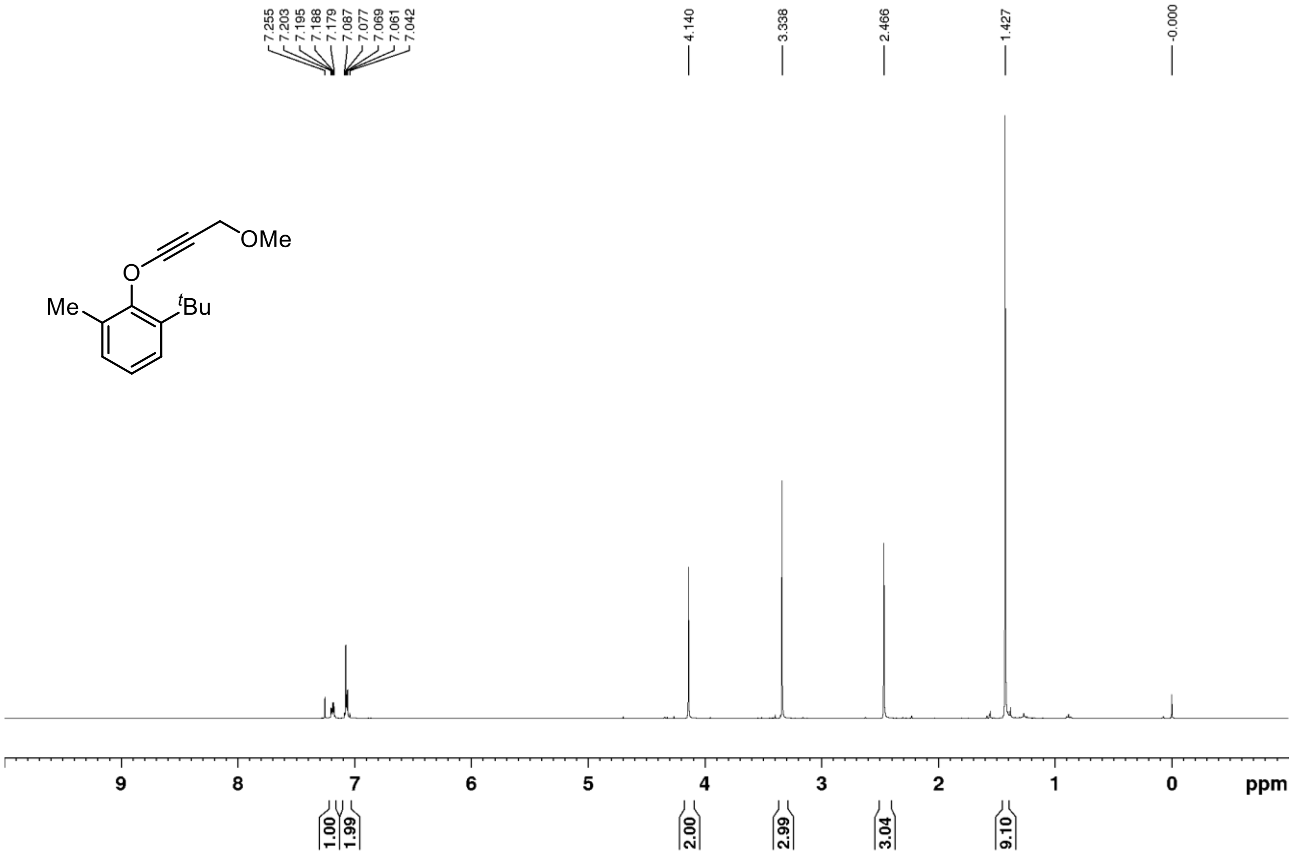

^{13}C NMR ($CDCl_3$, 101 MHz)

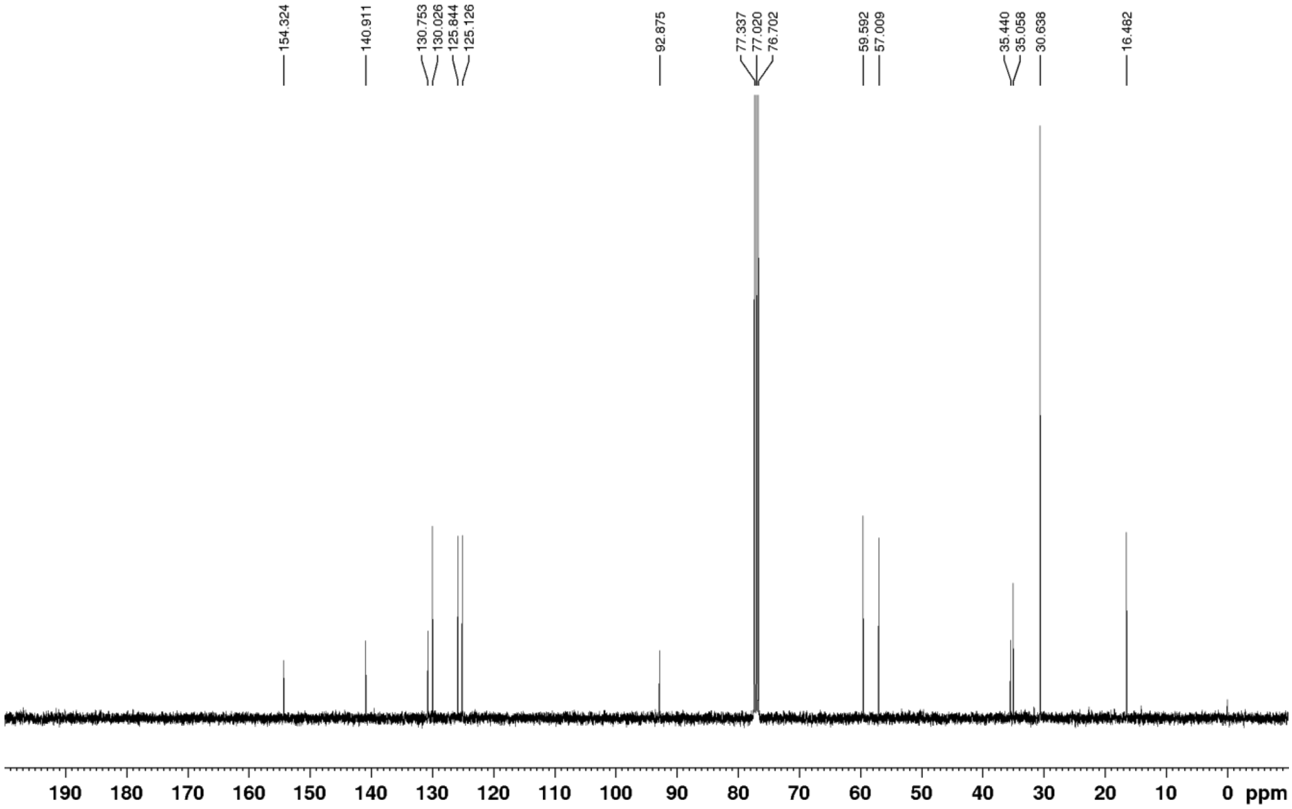

1-(*tert*-Butyl)-3-methyl-2-(prop-1-yn-1-yloxy)benzene (2q)

^{1}H NMR ($CDCl_3$, 400 MHz)

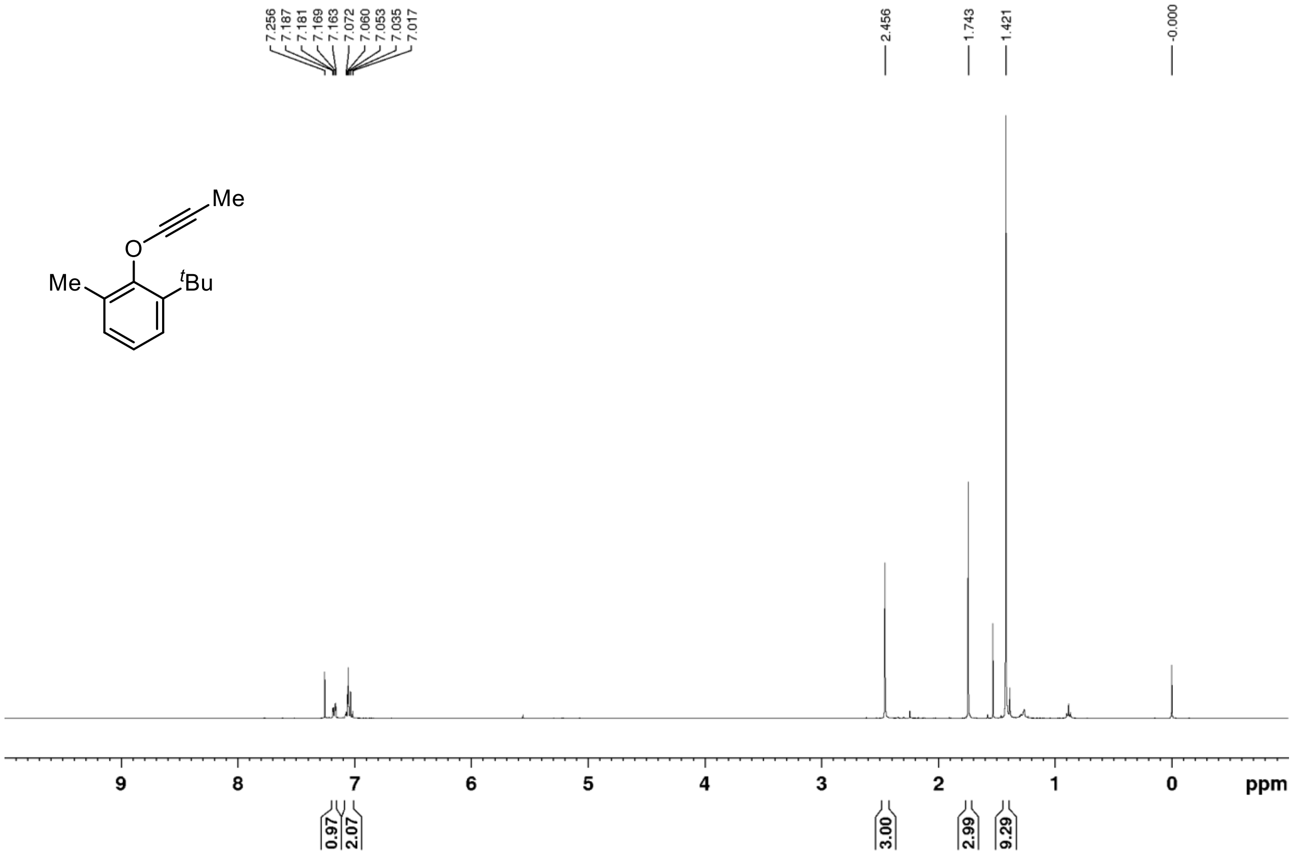

^{13}C NMR ($CDCl_3$, 101 MHz)

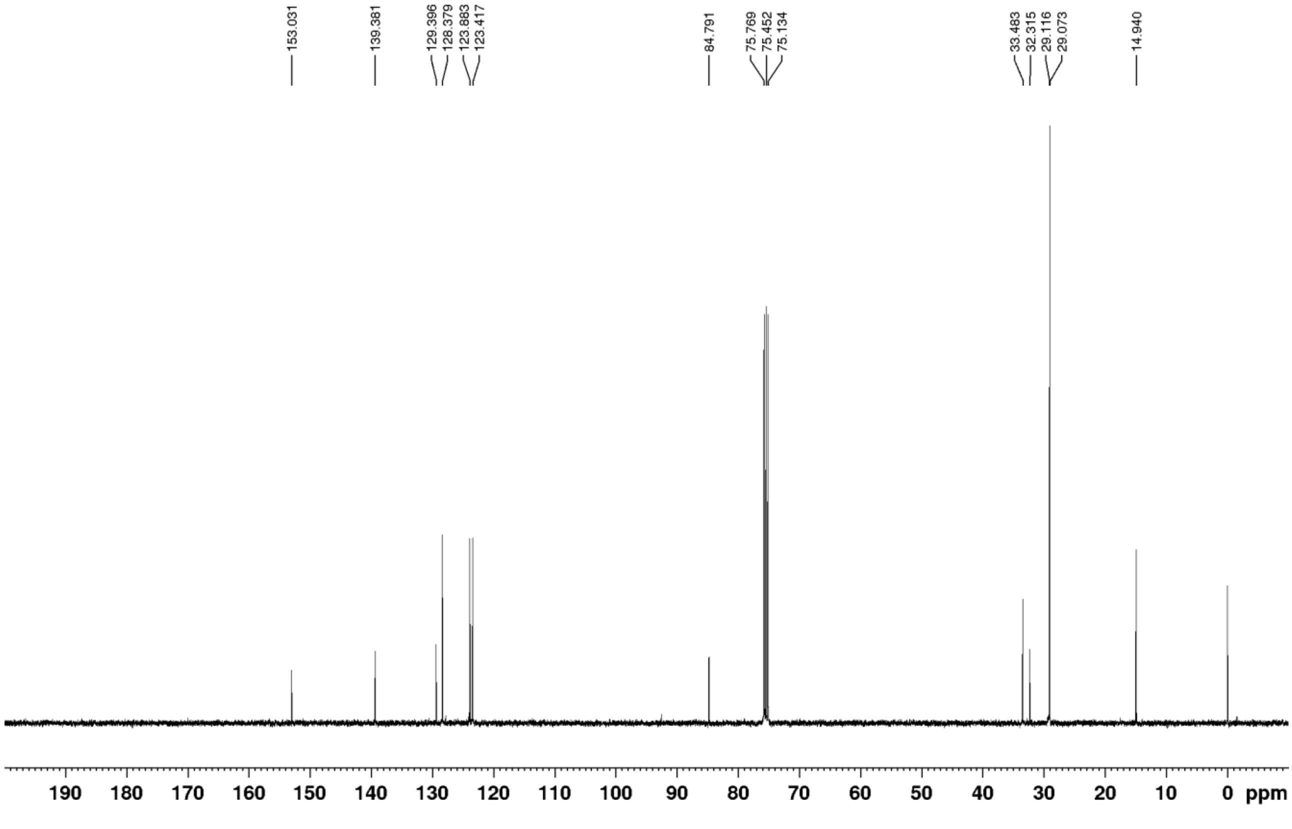

(+)-Dimethyl 6-(2-(*tert*-butyl)-6-methylphenoxy)-7-methyl-2-tosylisoindoline-4,5-dicarboxylate [(+)-3aa]

^{1}H NMR ($CDCl_3$, 400 MHz)

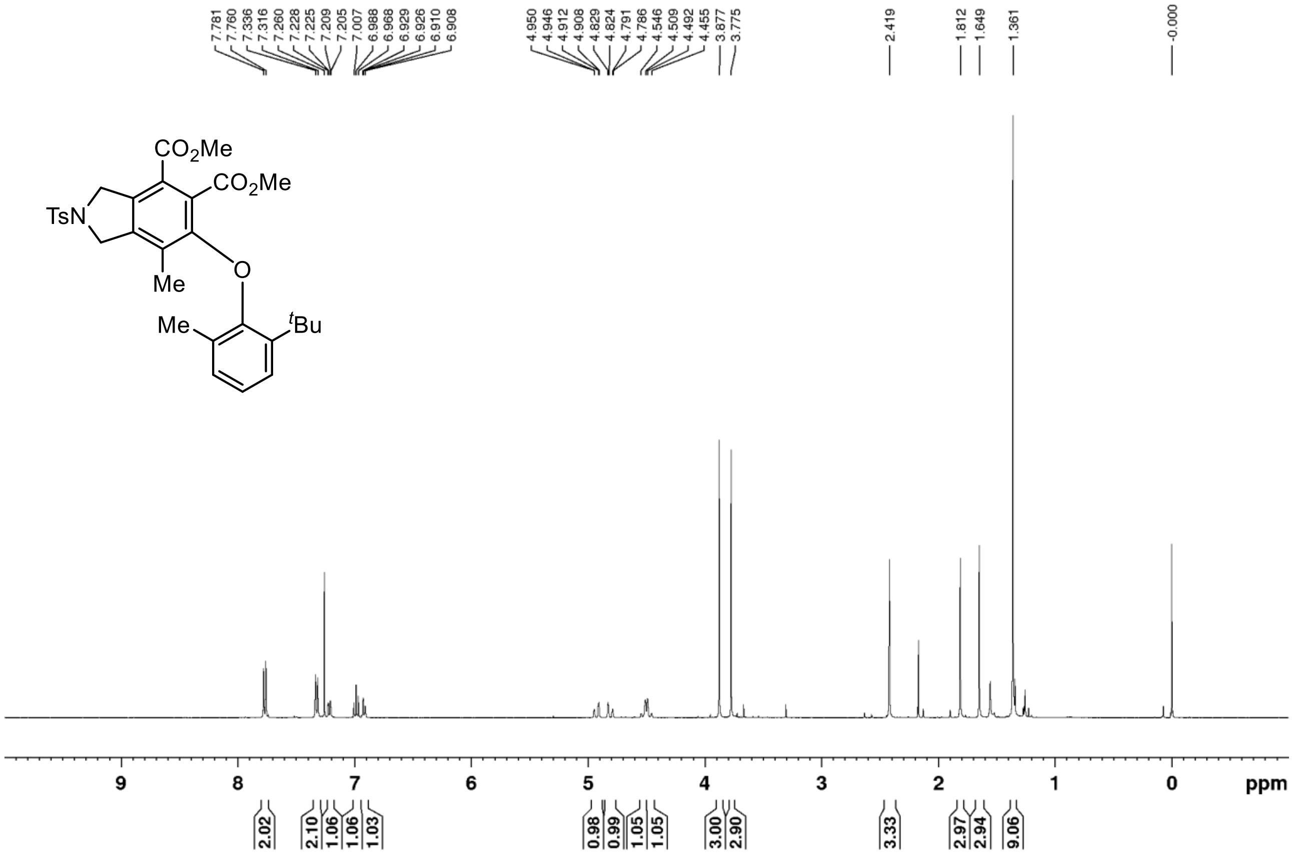

^{13}C NMR ($CDCl_3$, 101 MHz)

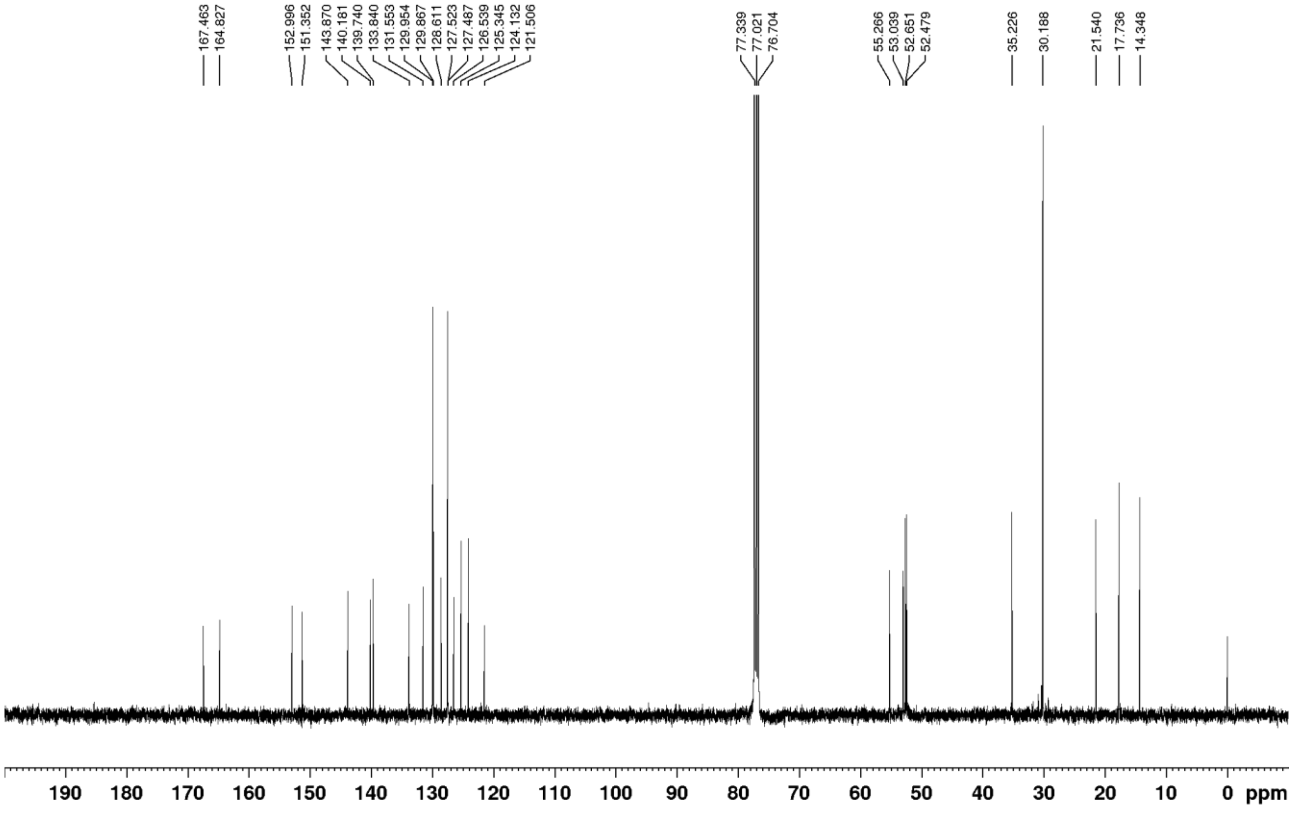

HMBC ($CDCl_3$, 400 MHz)

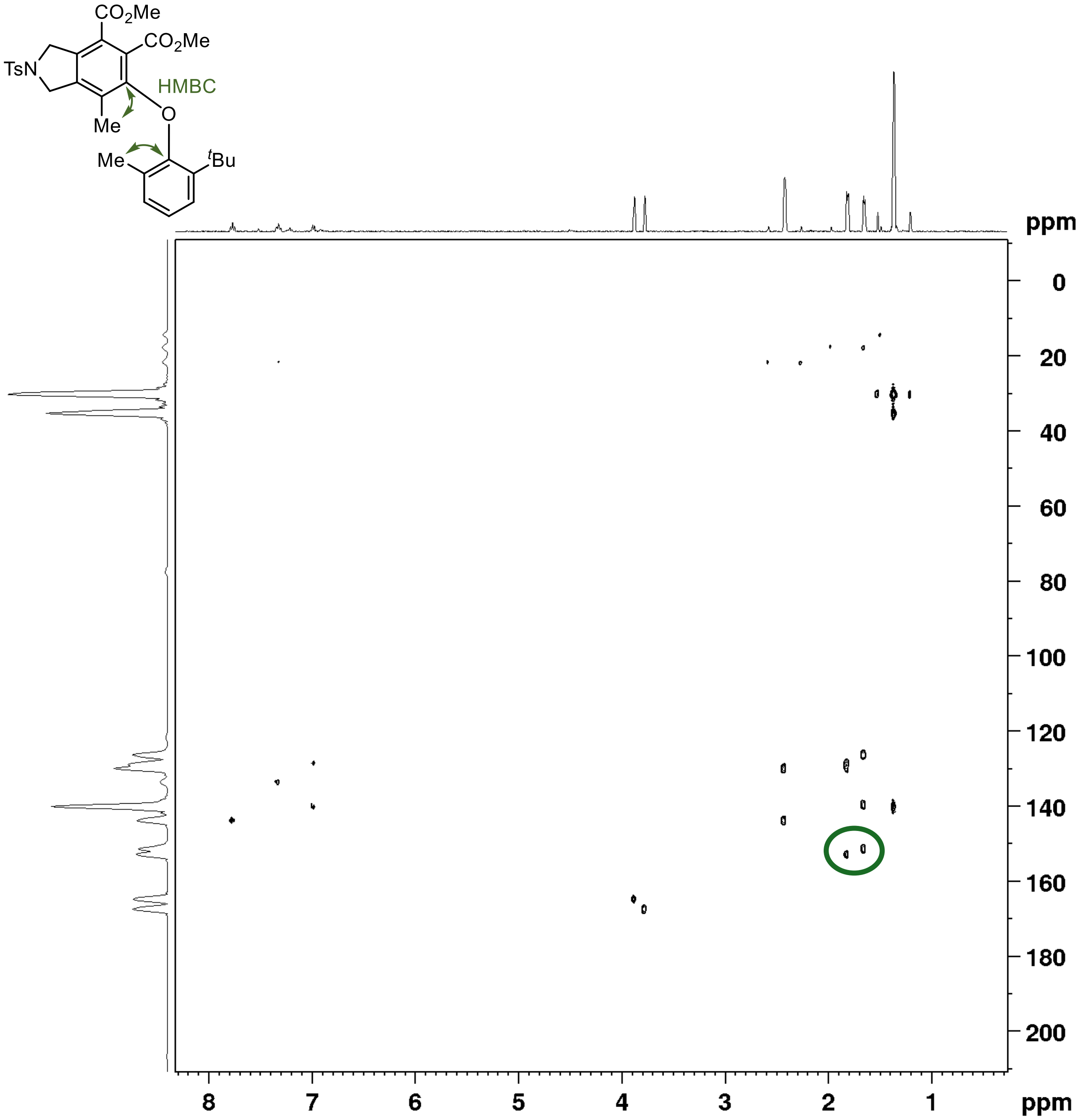

(+)-Dimethyl 5-(2-(*tert*-butyl)-6-methylphenoxy)-7-methyl-2-tosylisoindoline-4,6-dicarboxylate [(+)-4aa]

^{1}H NMR ($CDCl_3$, 400 MHz)

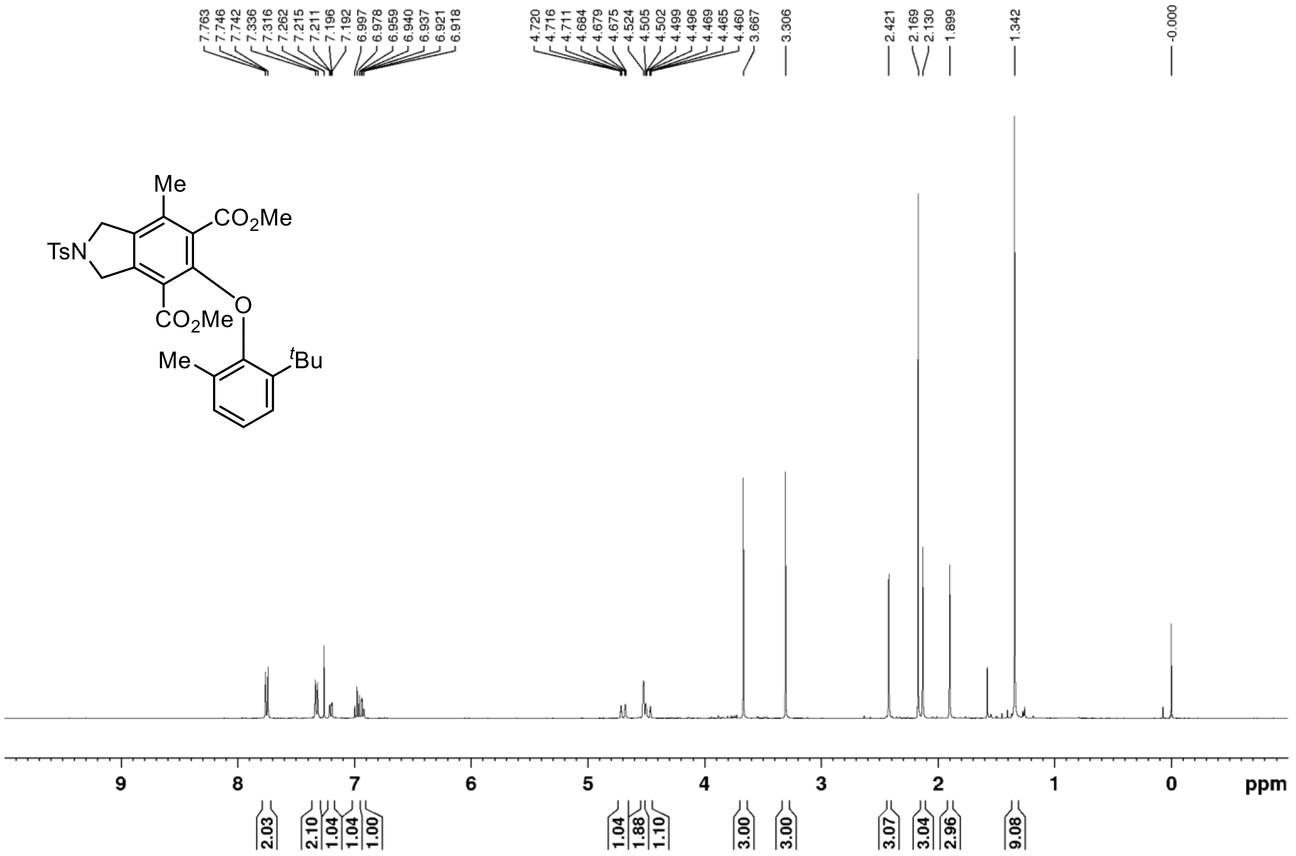

^{13}C NMR ($CDCl_3$, 101 MHz)

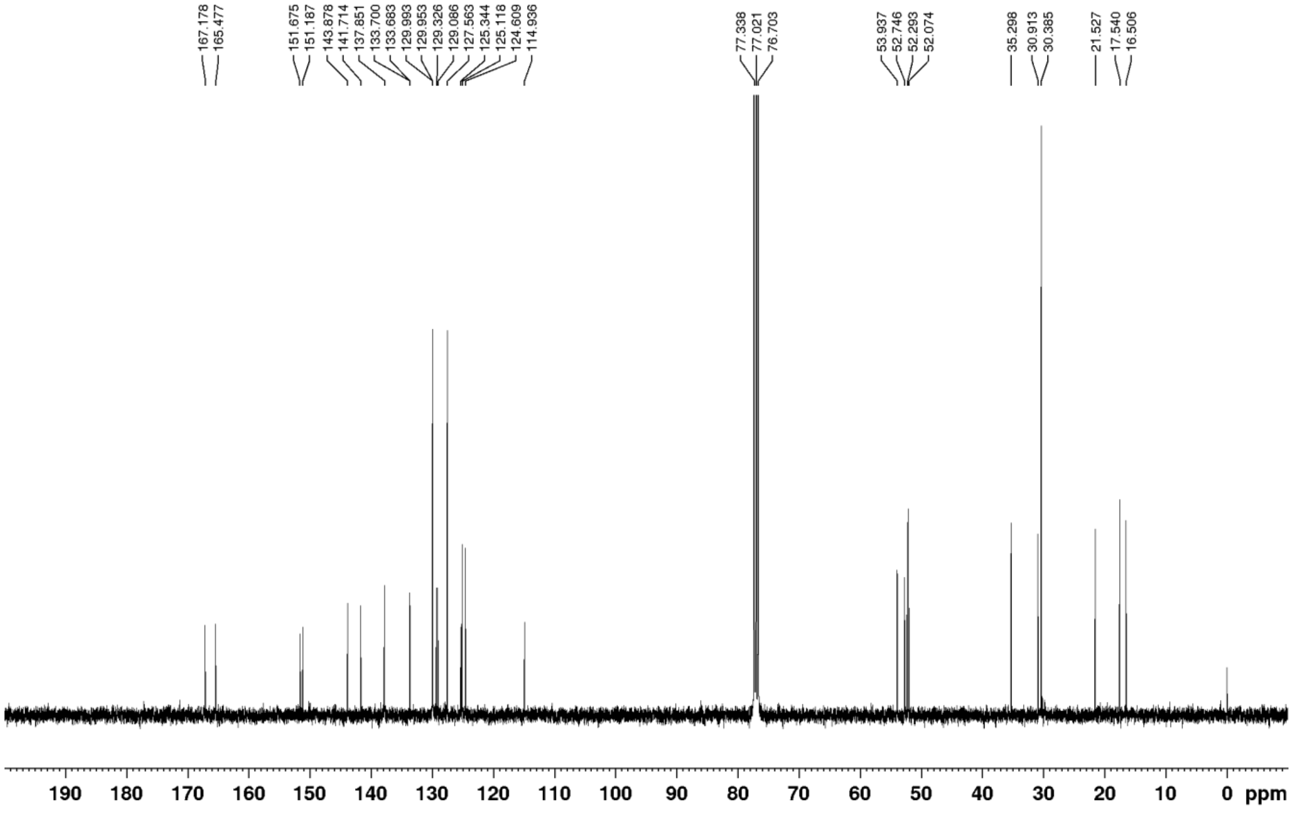

NOESY ($CDCl_3$, 400 MHz)

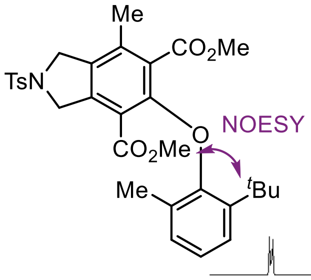

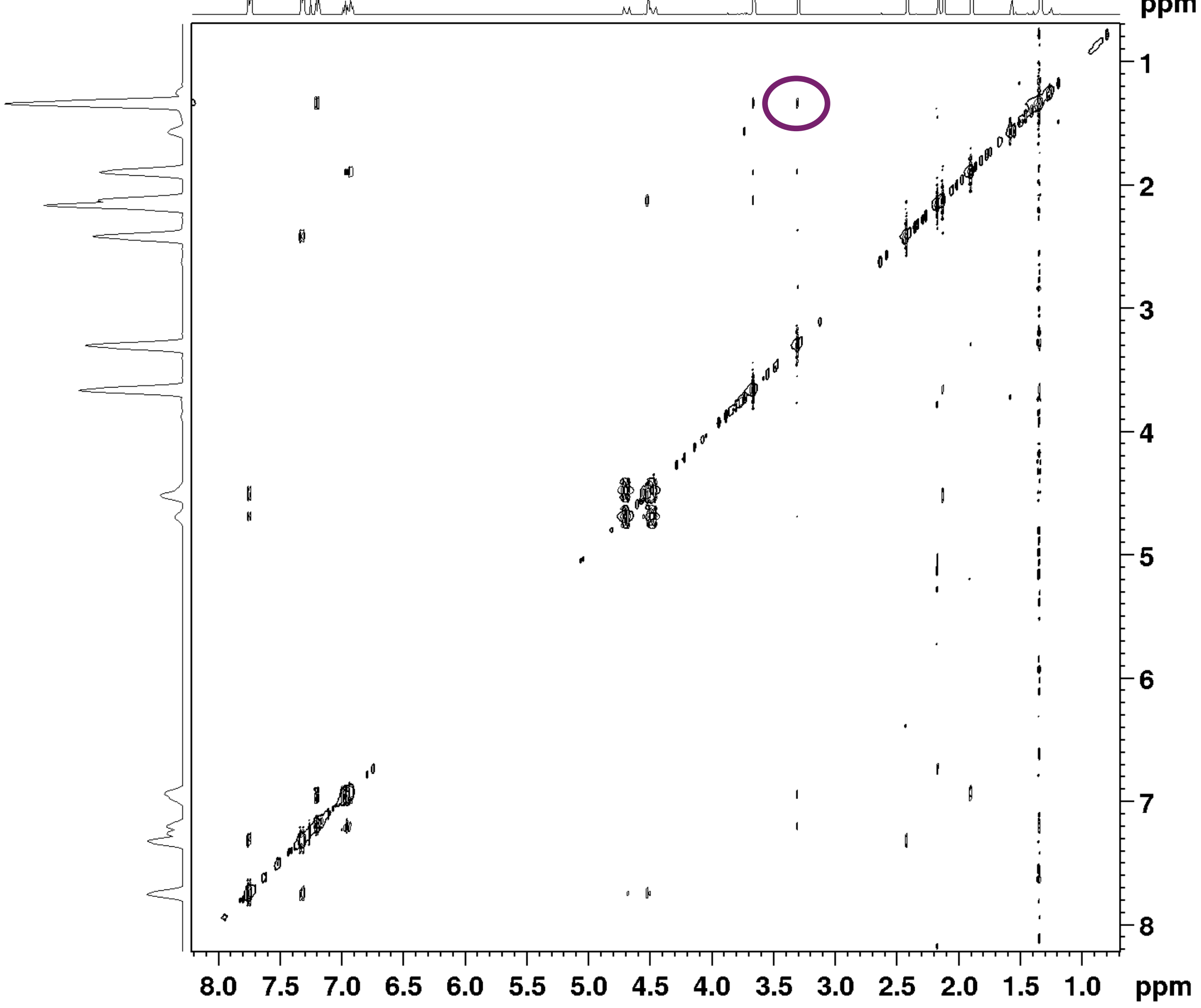

(+)-Dimethyl 6-(2-(*tert*-butyl)-6-methoxy-4-methylphenoxy)-7-methyl-2-tosylisoindoline-4,5-dicarboxylate [(+)-3ab]

^{1}H NMR ($CDCl_3$, 400 MHz)

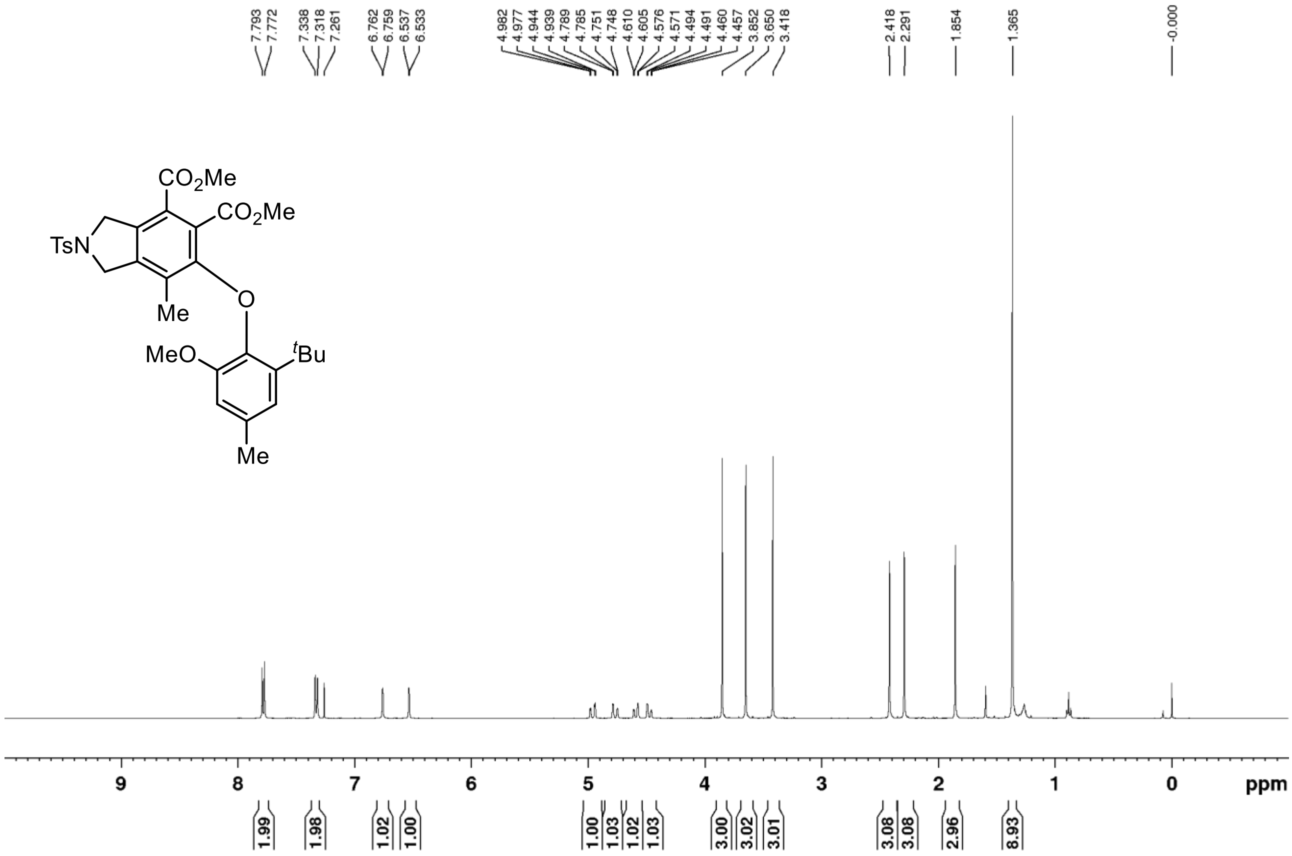

^{13}C NMR ($CDCl_3$, 101 MHz)

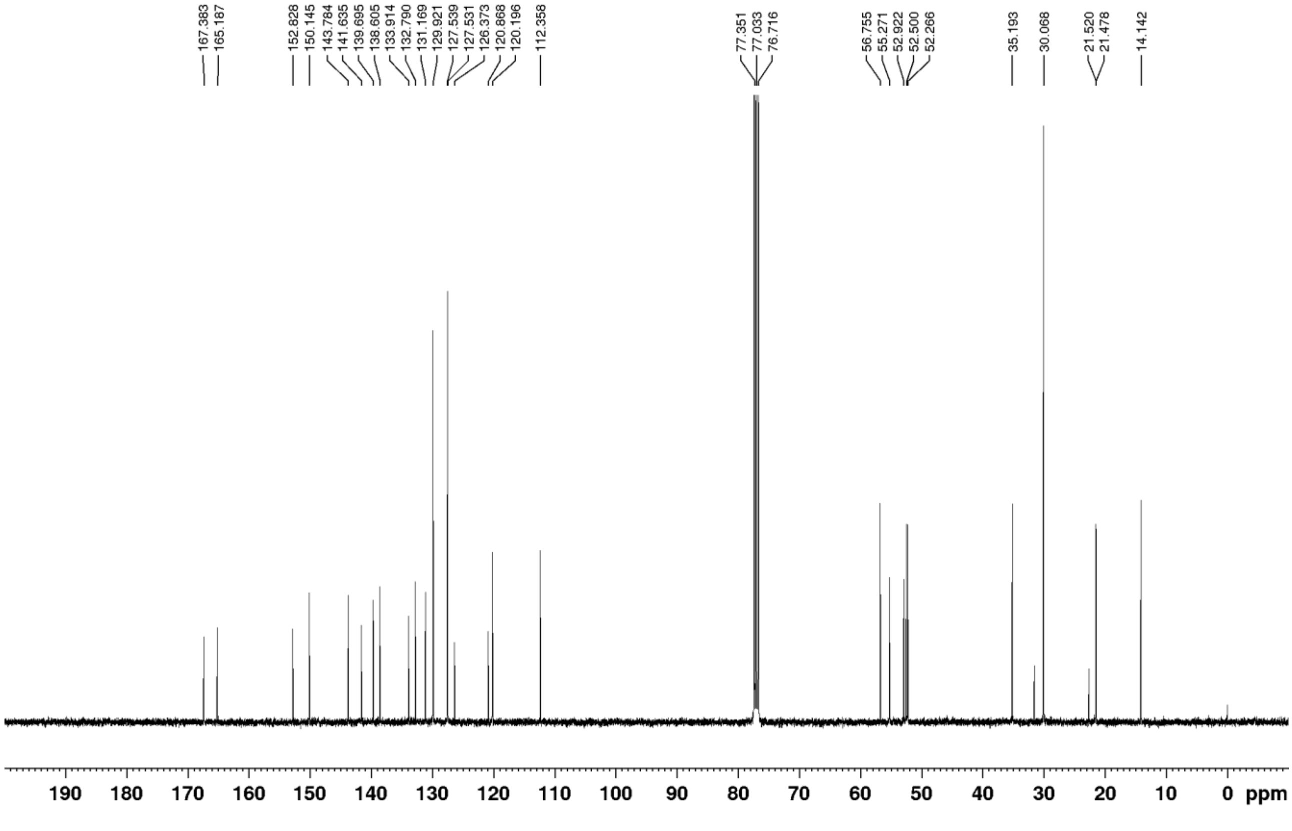

NOESY ($CDCl_3$, 400 MHz)

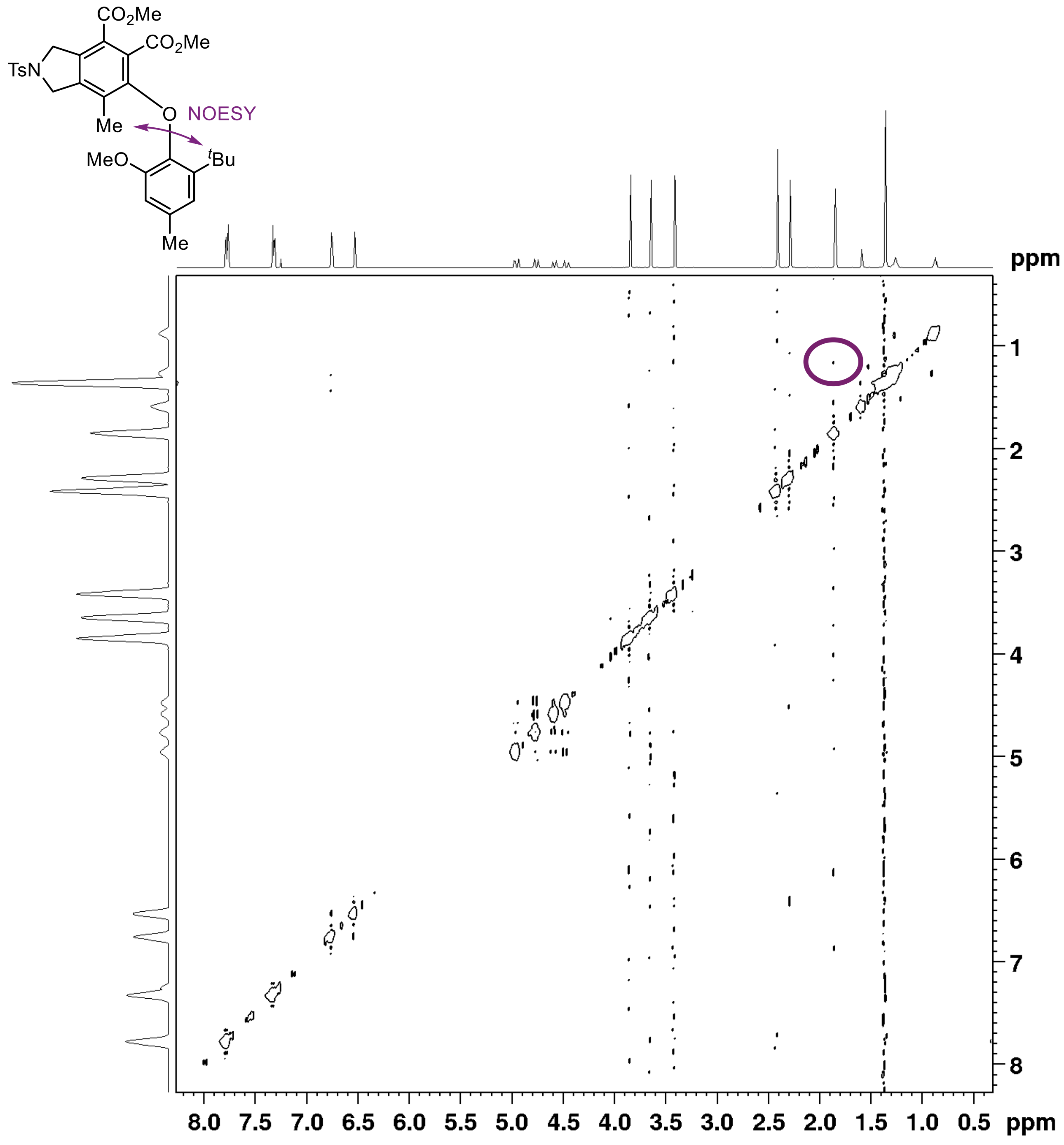

(–)-Dimethyl 5-(2-(*tert*-butyl)-6-methoxy-4-methylphenoxy)-7-methyl-2-tosylisoindoline-4,6-dicarboxylate [(–)-4ab]

^{1}H NMR ($CDCl_3$, 400 MHz)

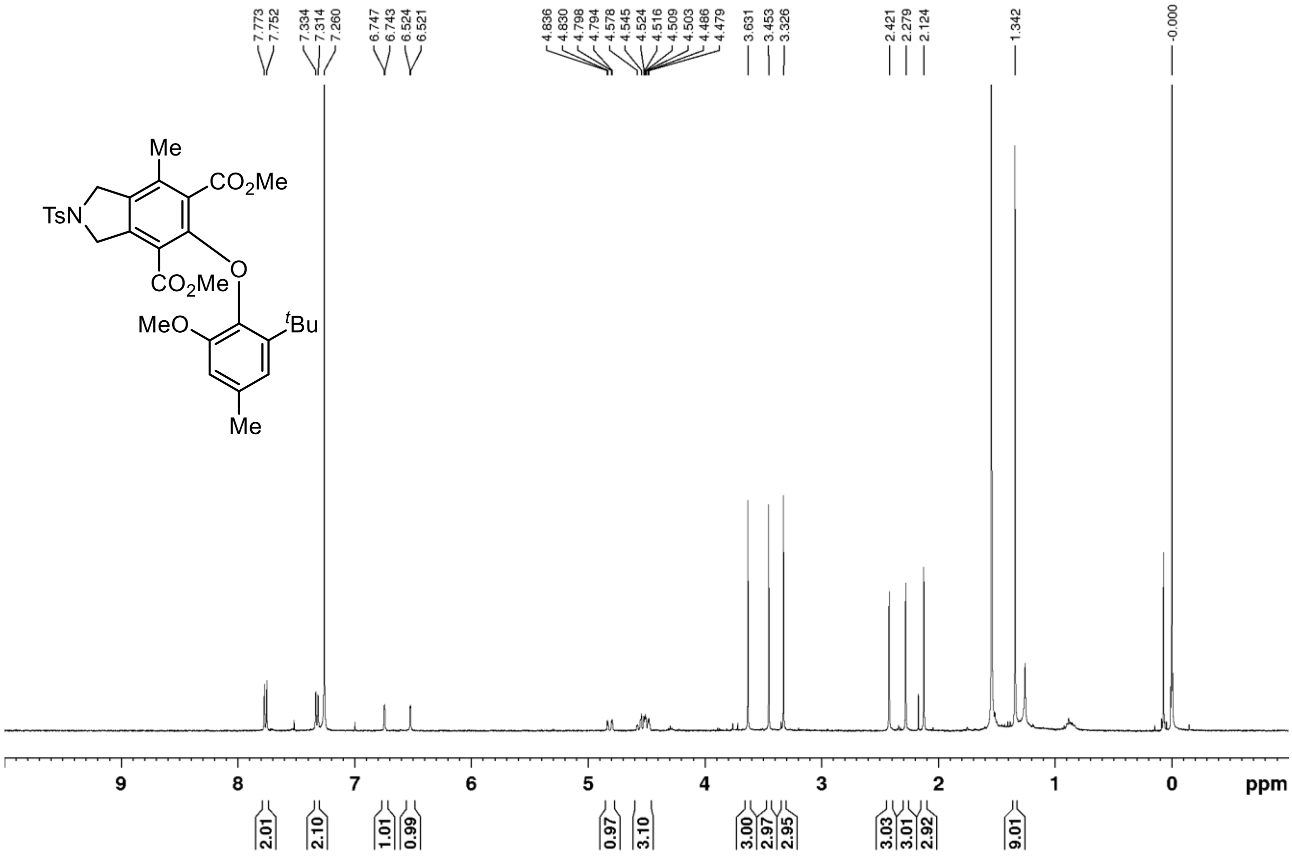

^{13}C NMR ($CDCl_3$, 101 MHz)

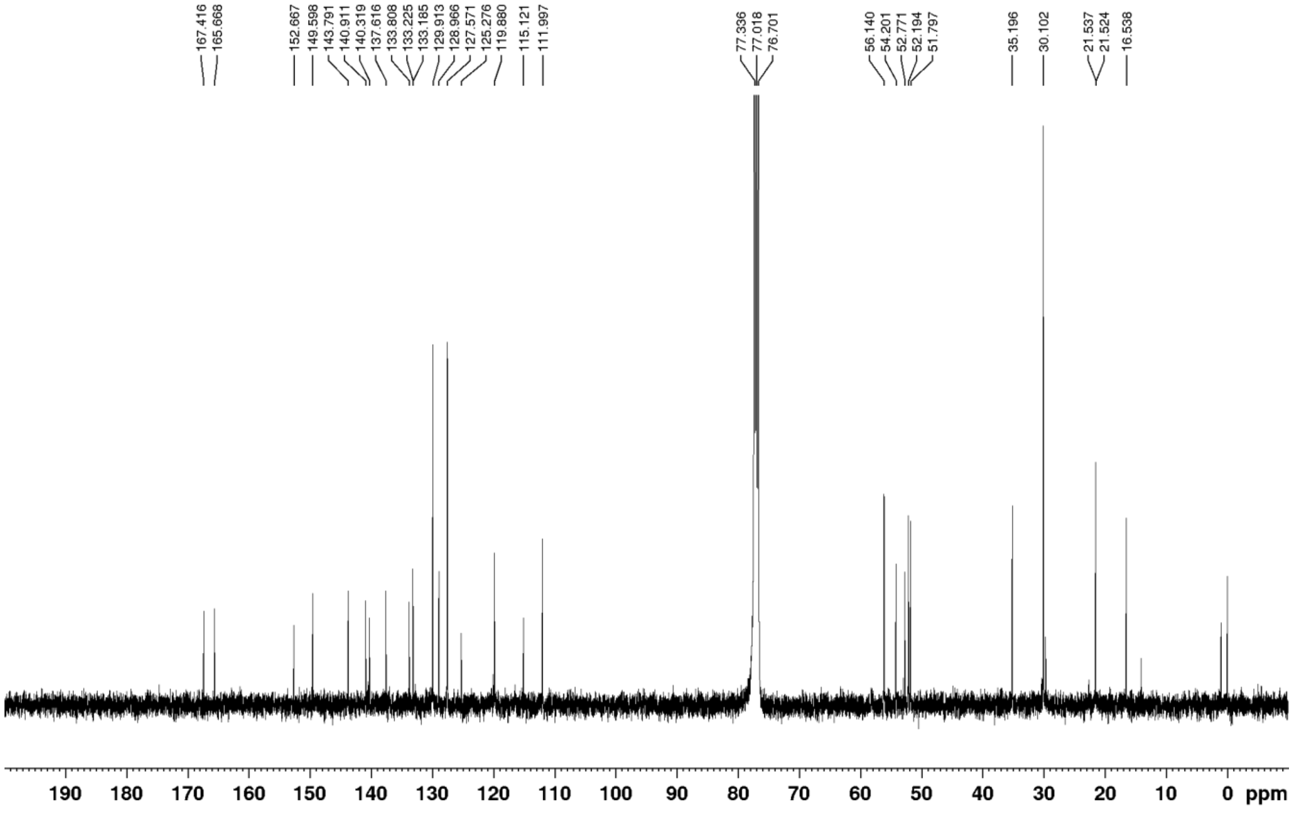

NOESY ($CDCl_3$, 400 MHz)

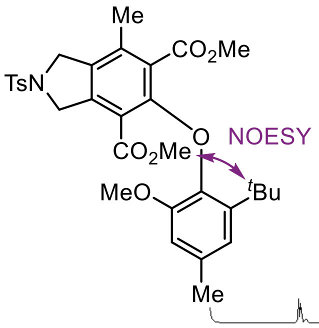

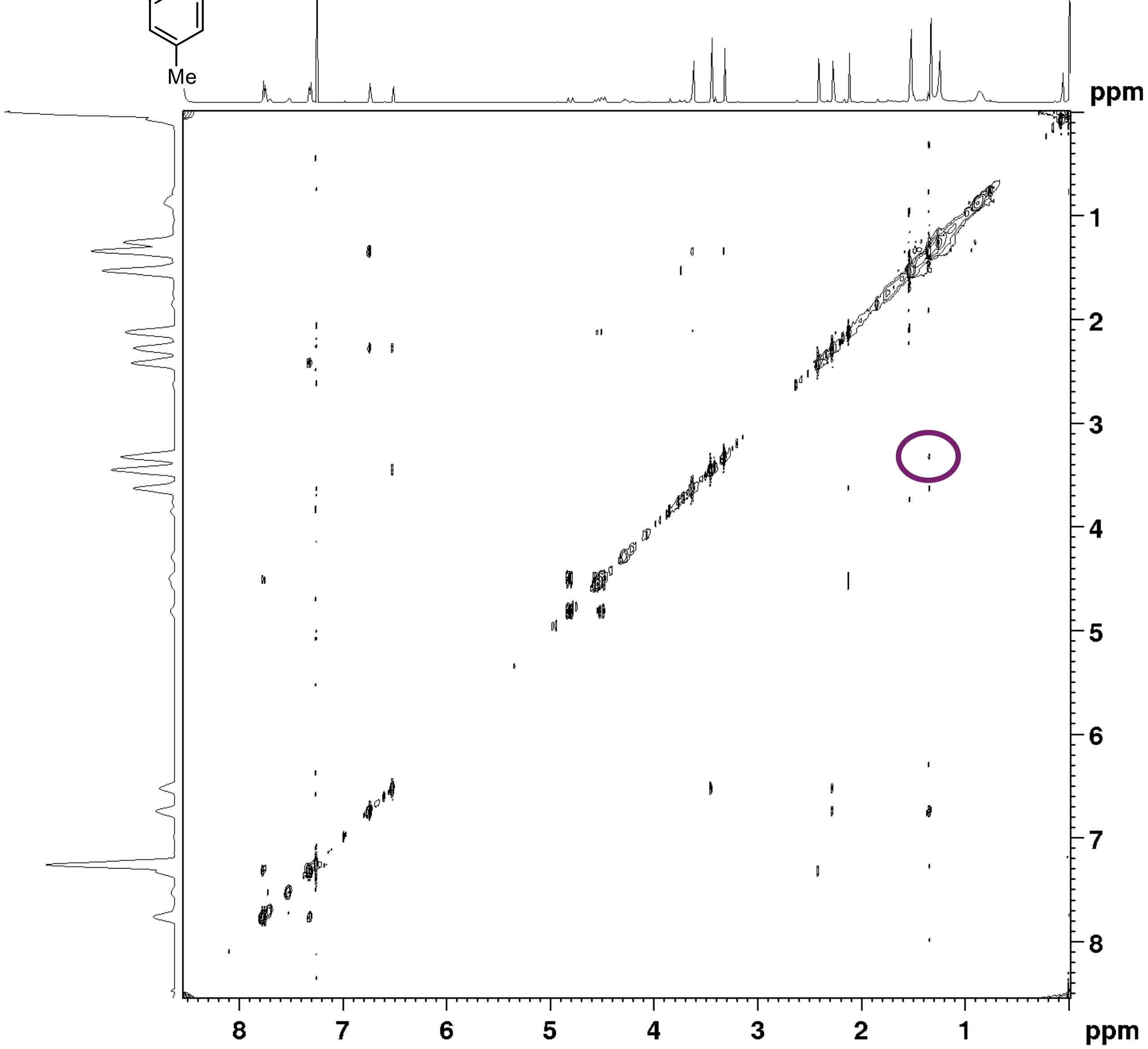

(+)-Dimethyl 6-((3-(*tert*-butyl)-4',5-dimethyl-[1,1'-biphenyl]-2-yl)oxy)-7-methyl-2-tosylisoindoline-4,5-dicarboxylate (3ac)

^{1}H NMR ($CDCl_3$, 400 MHz)

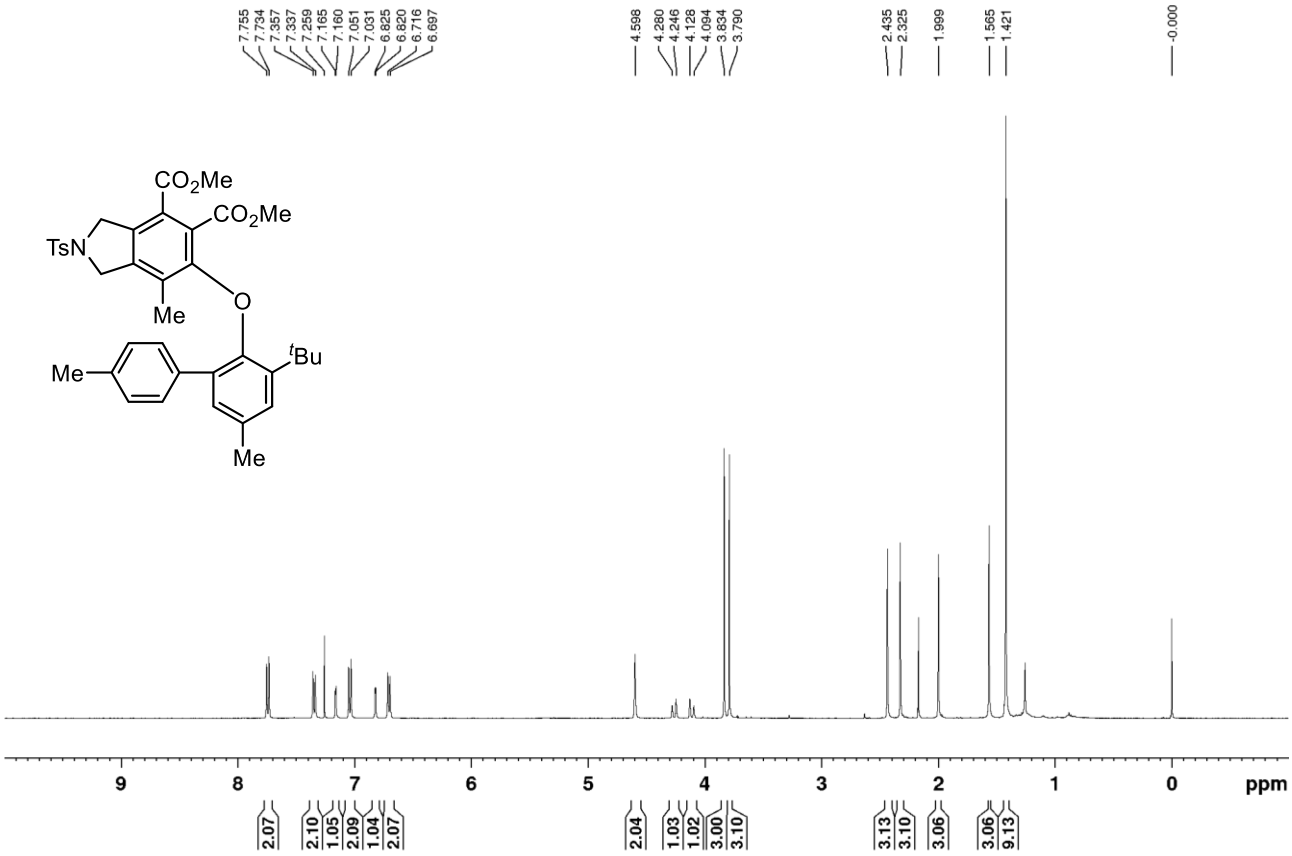

^{13}C NMR ($CDCl_3$, 101 MHz)

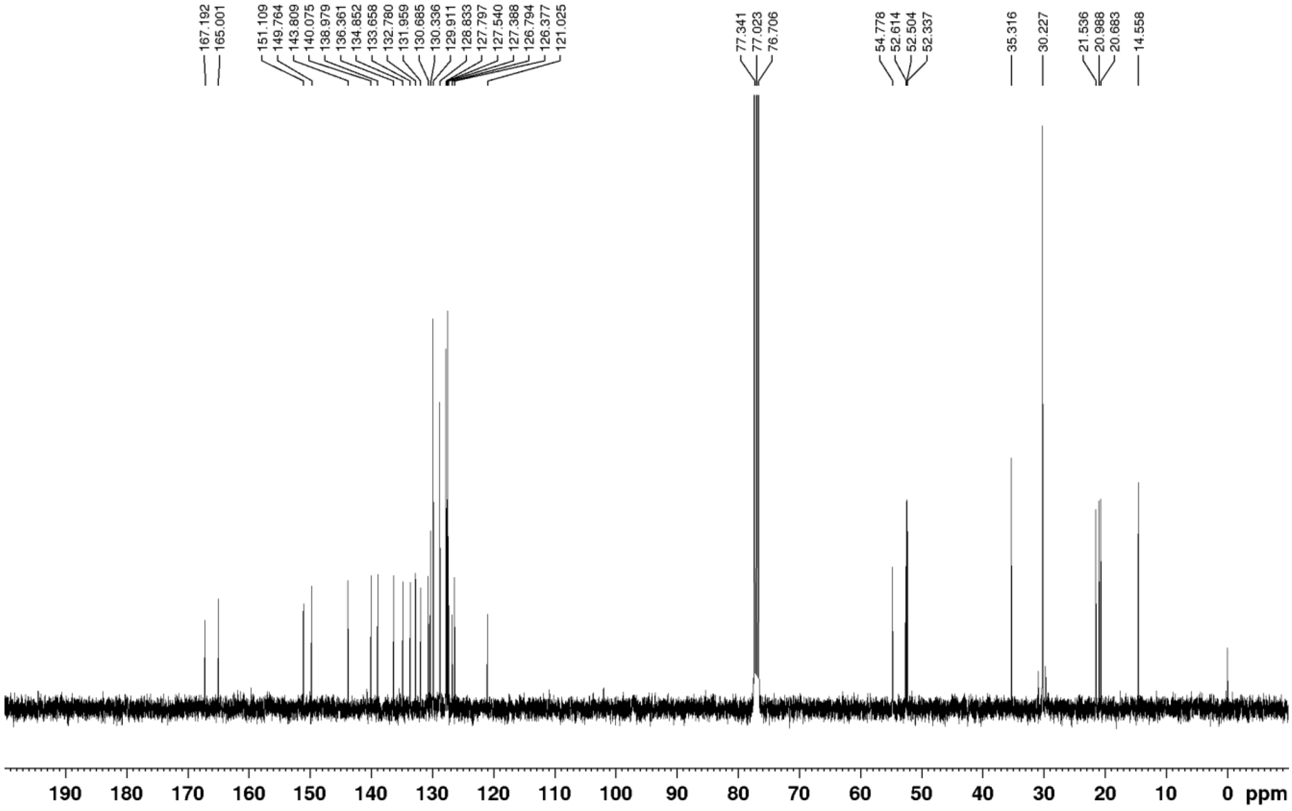

HMBC ($CDCl_3$, 400 MHz)

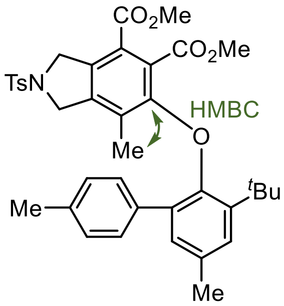

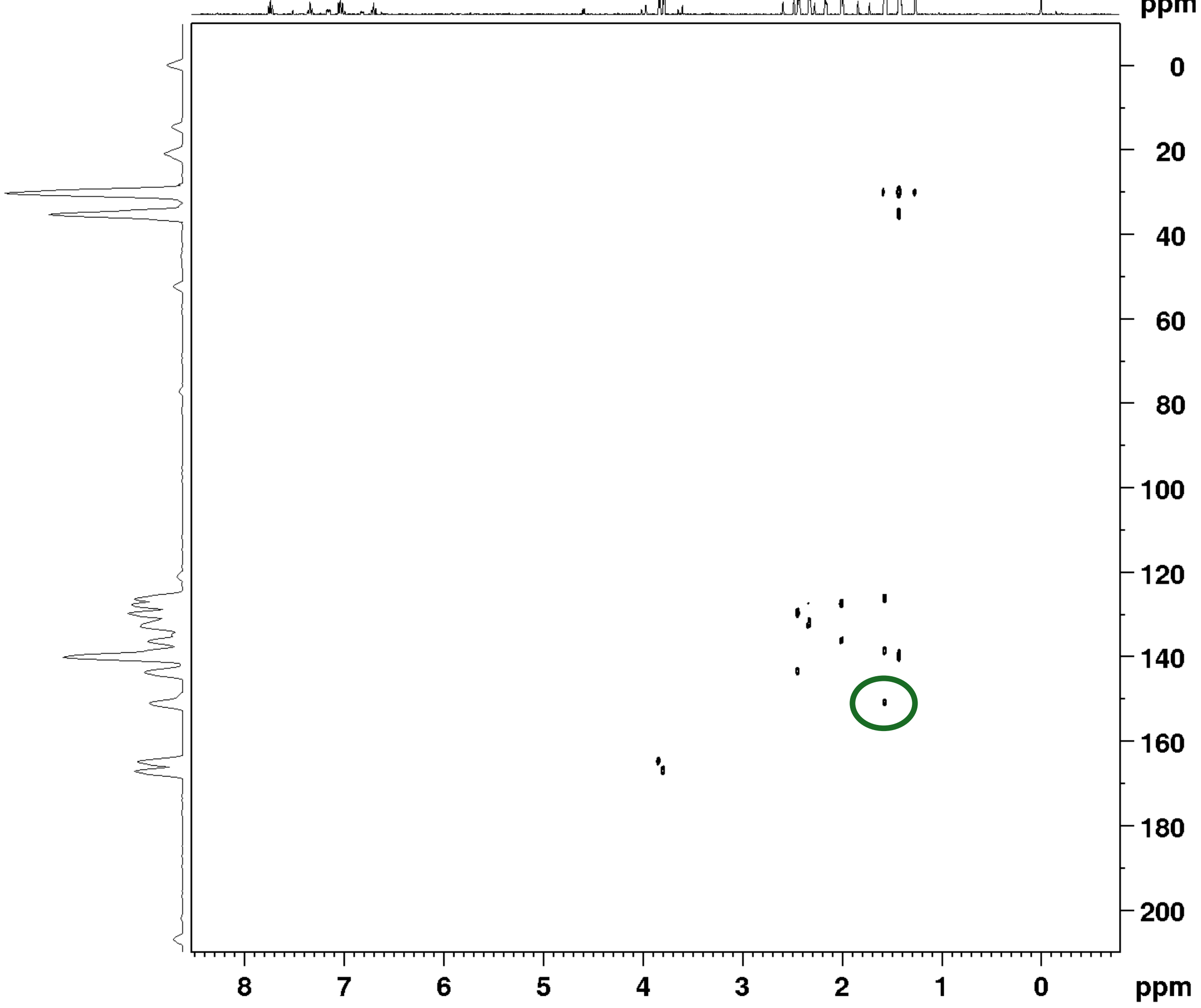

(+)-Dimethyl 5-((3-(*tert*-butyl)-4',5-dimethyl-[1,1'-biphenyl]-2-yl)oxy)-7-methyl-2-tosylisoindoline-4,6-dicarboxylate [(+)-4ac]

^{1}H NMR ($CDCl_3$, 400 MHz)

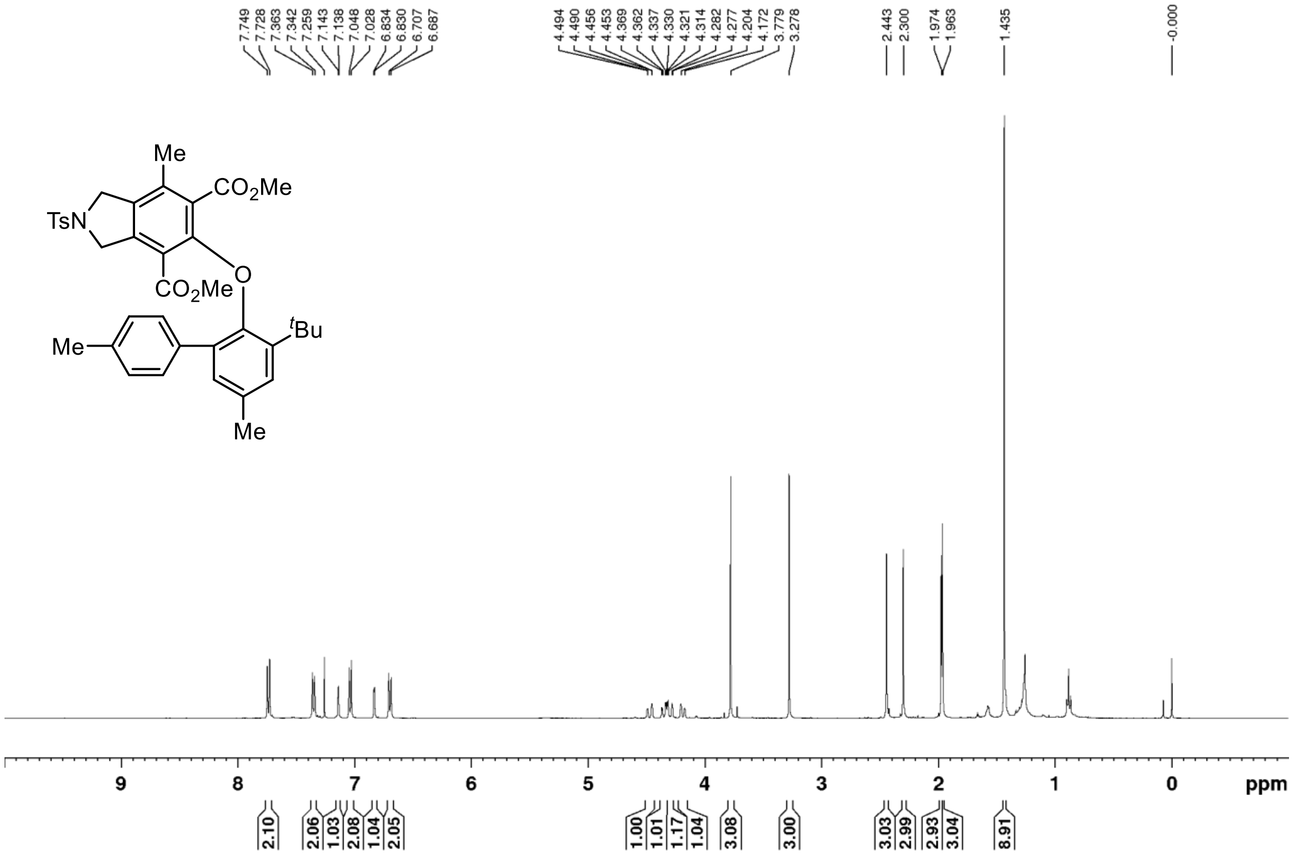

^{13}C NMR ($CDCl_3$, 101 MHz)

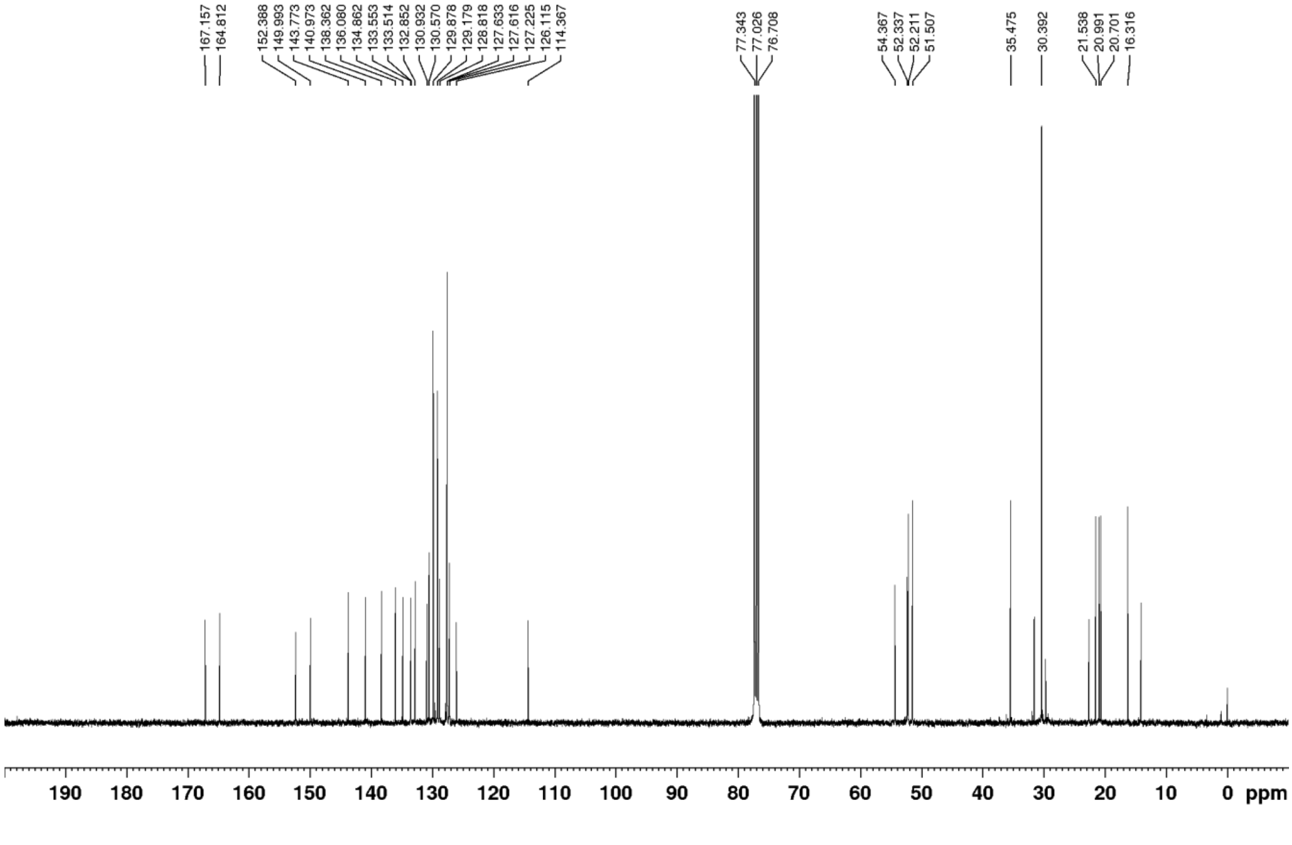

NOESY ($CDCl_3$, 400 MHz)

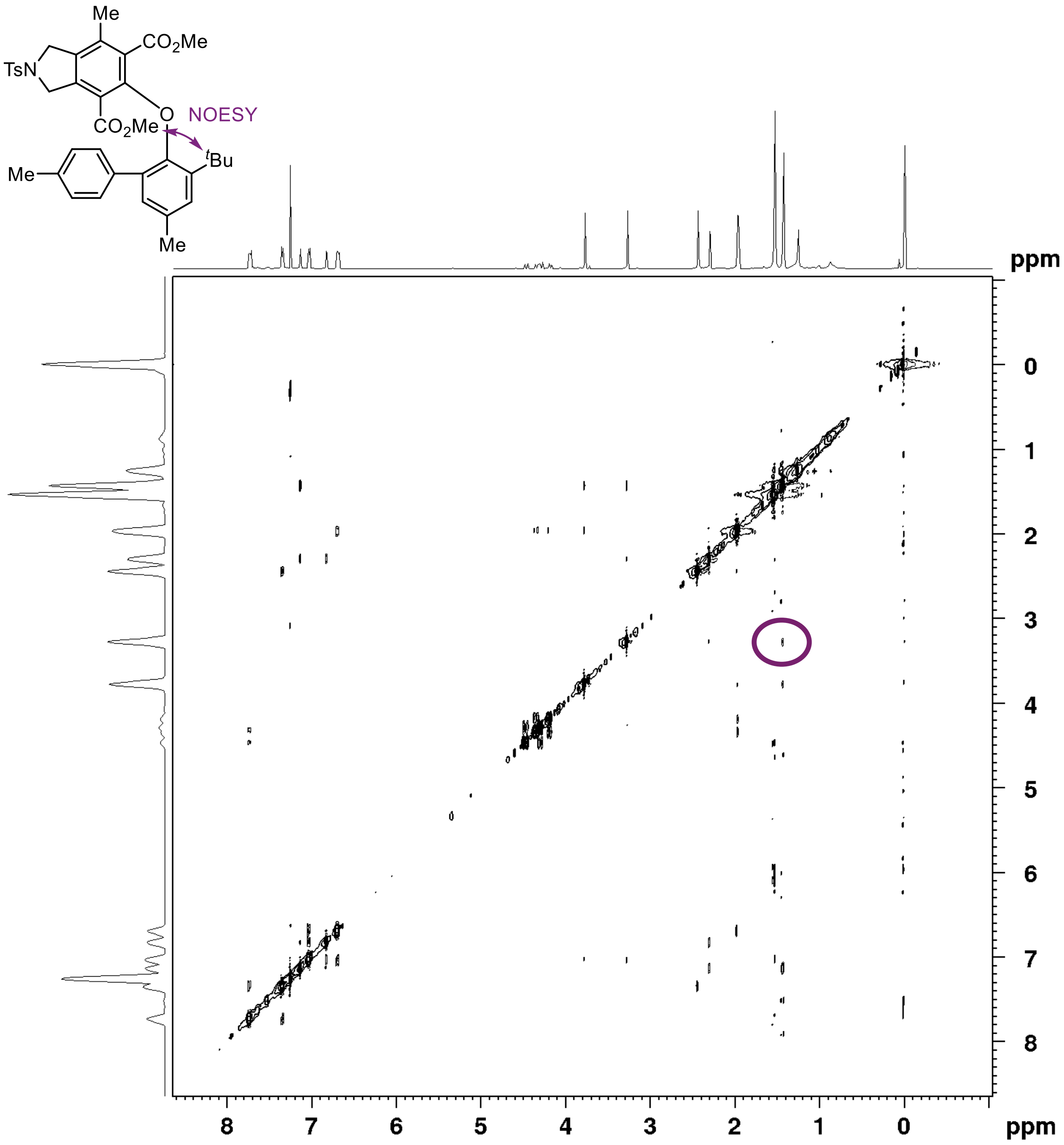

(+)-Dimethyl 6-(2-(*tert*-butyl)-6-ethoxy-4-methylphenoxy)-7-methyl-2-tosylisoindoline-4,5-dicarboxylate [(+)-3ad]

^{1}H NMR ($CDCl_3$, 400 MHz)

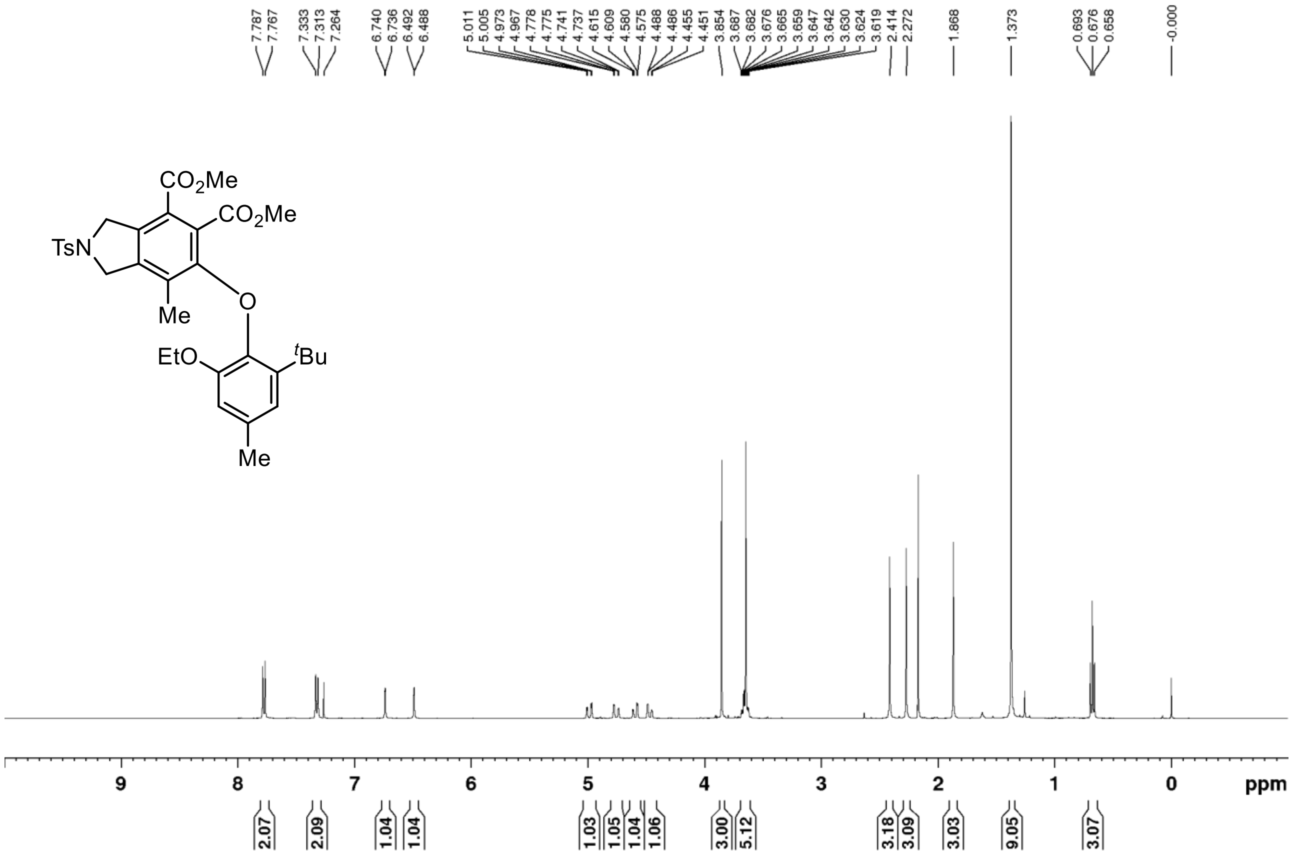

^{13}C NMR ($CDCl_3$, 101 MHz)

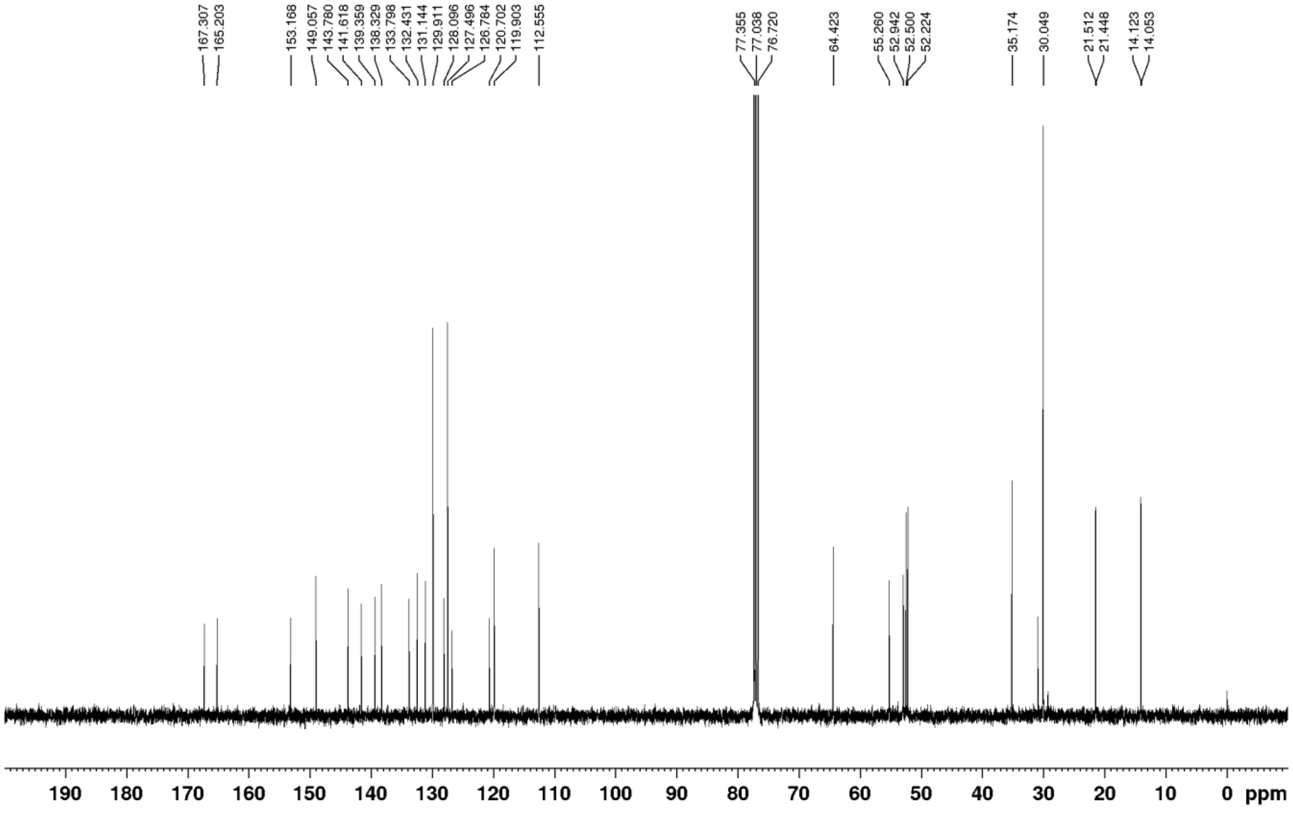

HMBC ($CDCl_3$, 400 MHz)

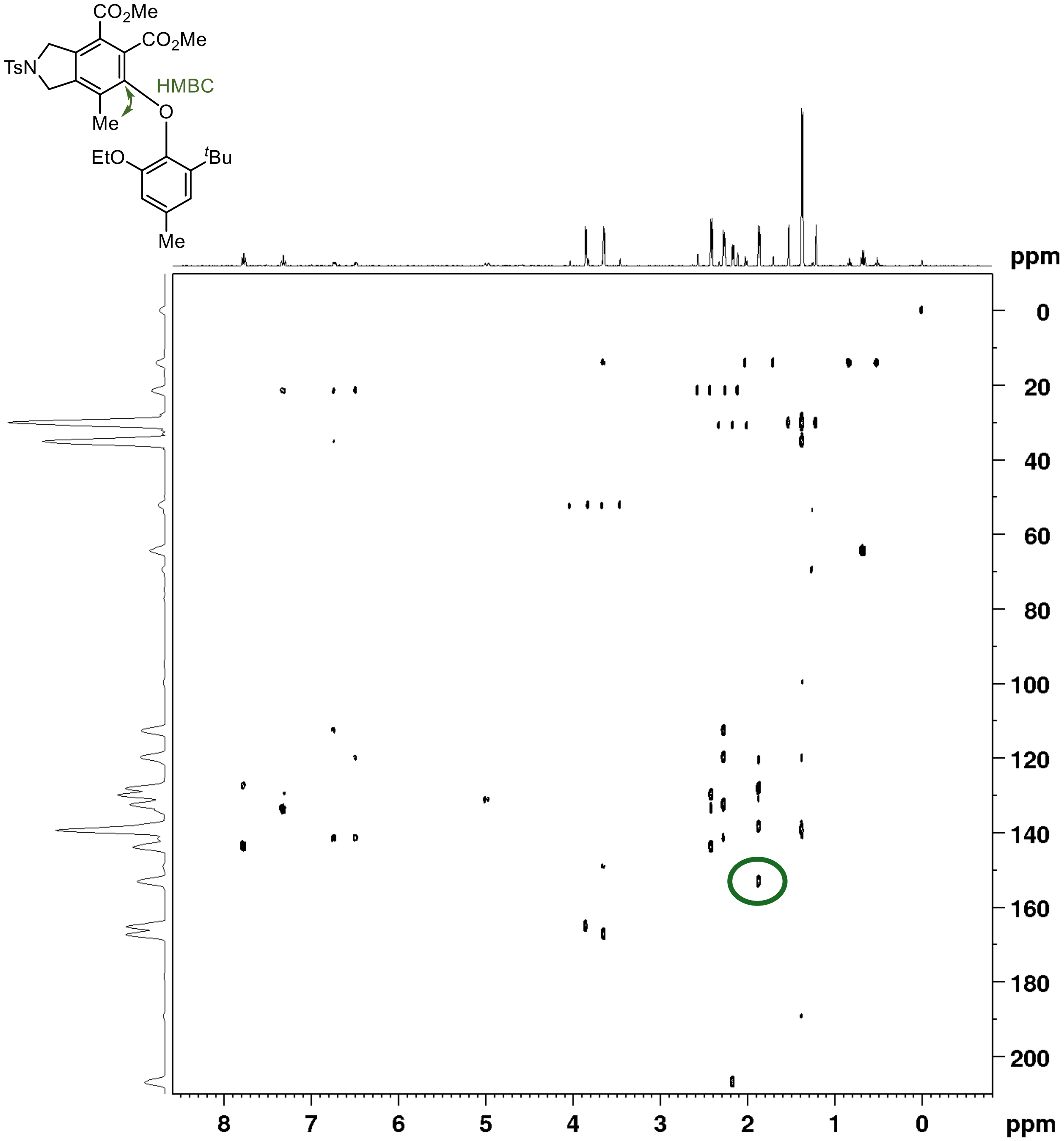

(+)-Dimethyl 6-(2-(*tert*-butyl)-6-(methoxymethoxy)-4-methylphenoxy)-7-methyl-2-tosylisoindoline-4,5-dicarboxylate [(+)-3ae]

^{1}H NMR ($CDCl_3$, 400 MHz)

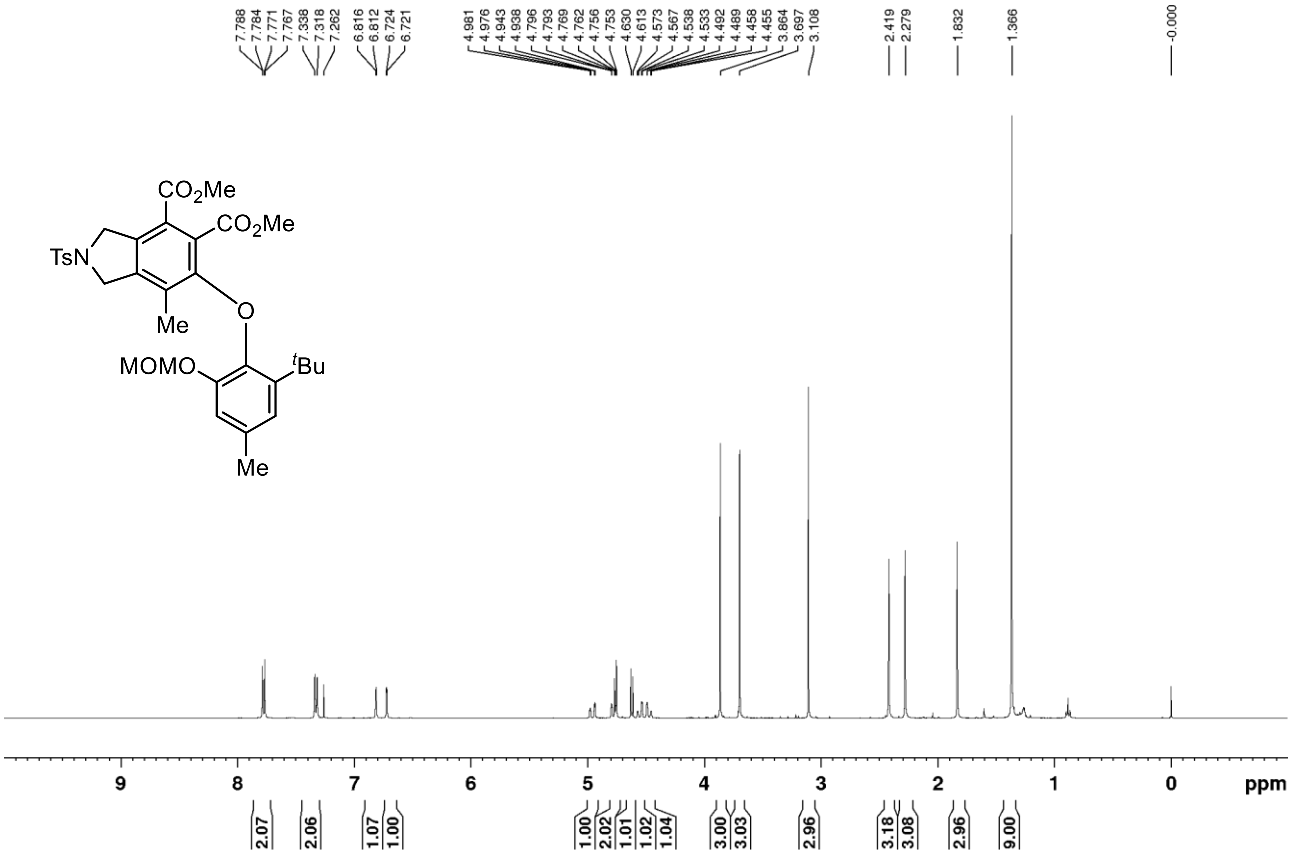

^{13}C NMR ($CDCl_3$, 101 MHz)

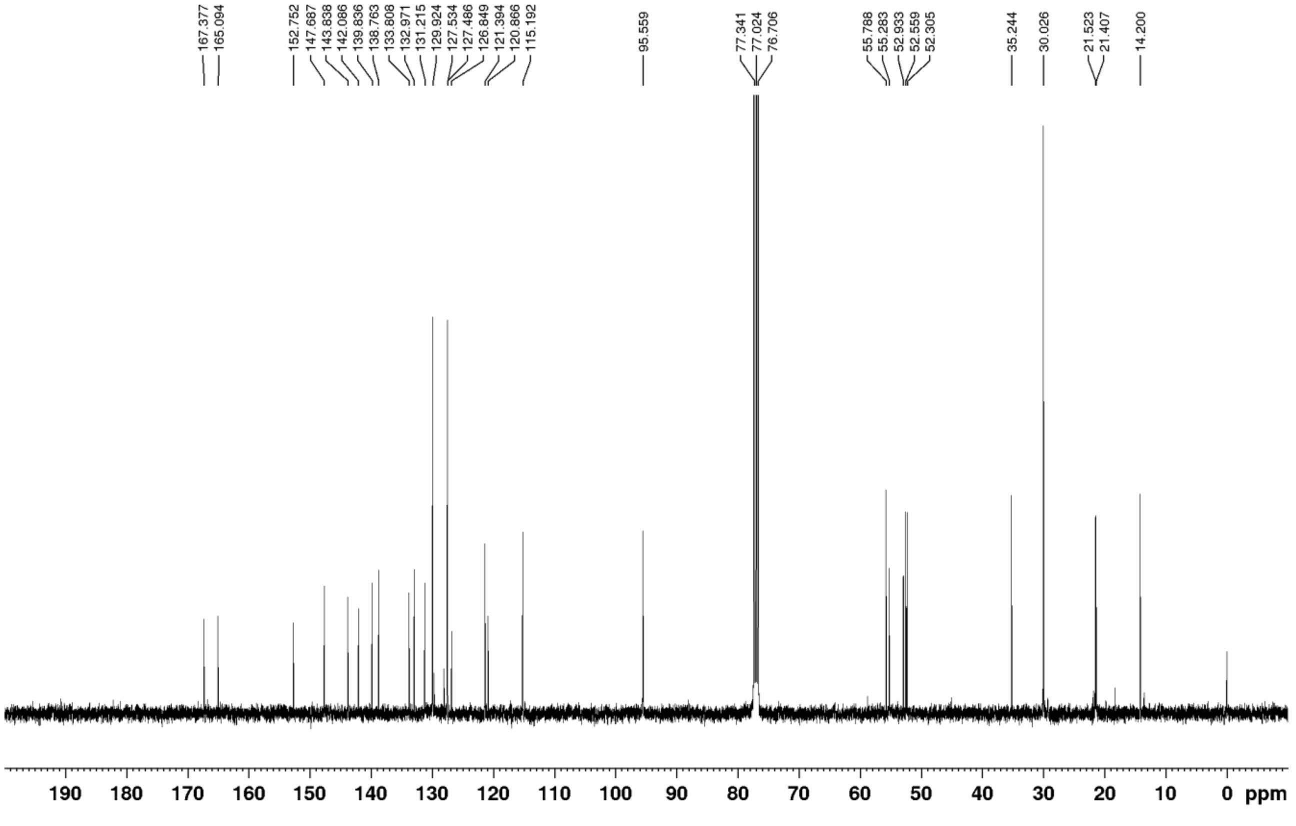

NOESY ($CDCl_3$, 400 MHz)

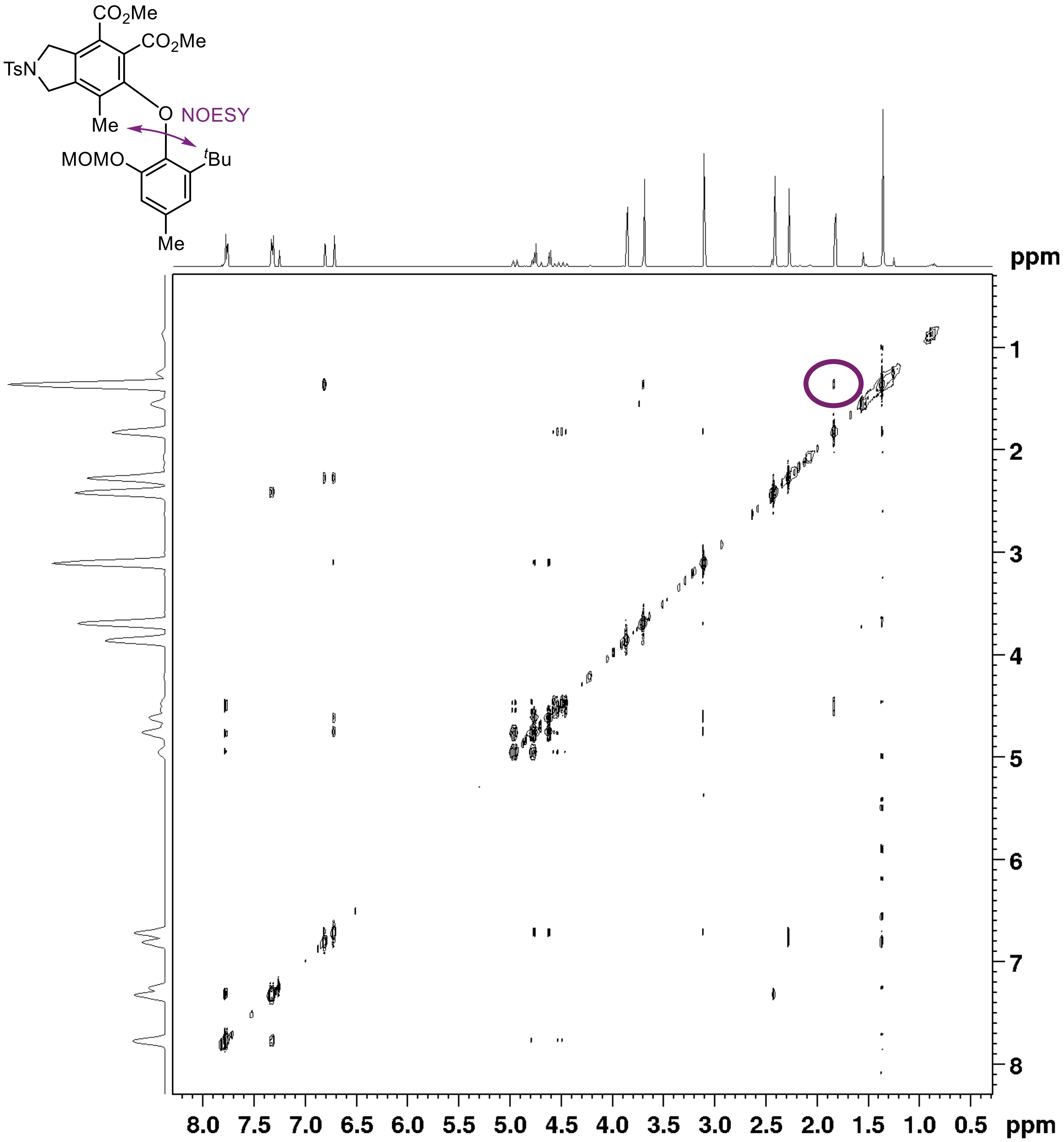

(+)-5-Ethyl 4-methyl 6-(2-(*tert*-butyl)-6-methoxy-4-methylphenoxy)-7-methyl-2-tosylisoindoline-4,5-dicarboxylate [(+)-3af]

^{1}H NMR ($CDCl_3$, 400 MHz)

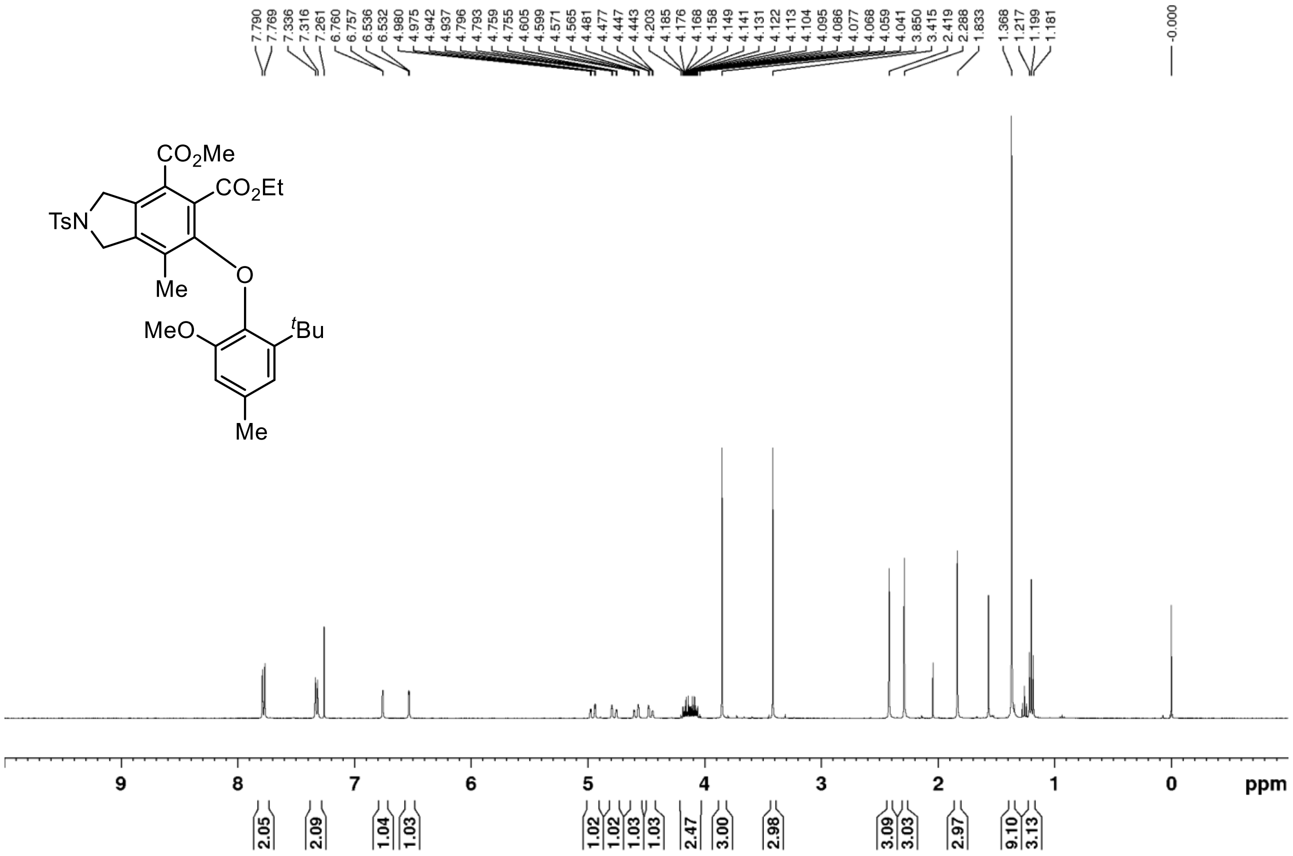

^{13}C NMR ($CDCl_3$, 101 MHz)

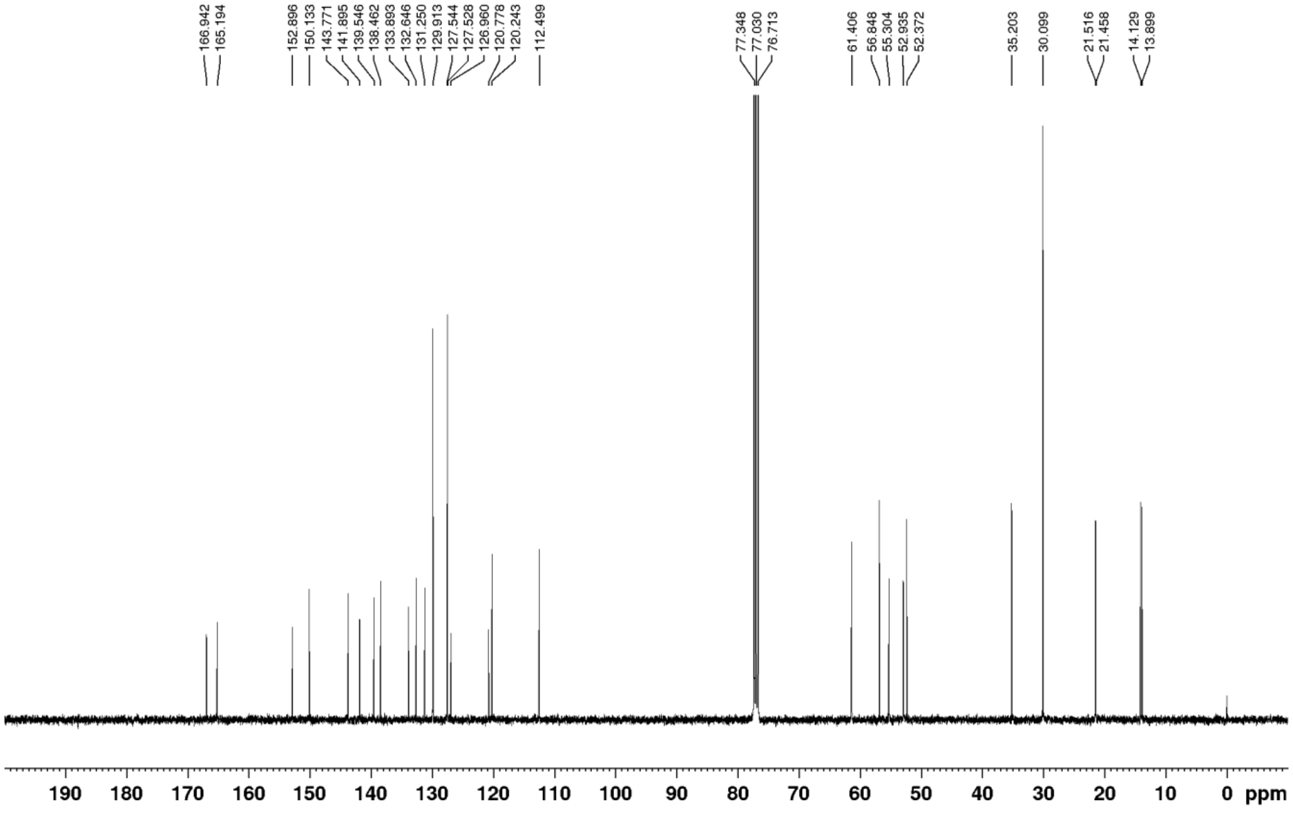

HMBC ($CDCl_3$, 400 MHz)

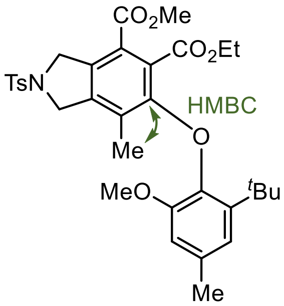

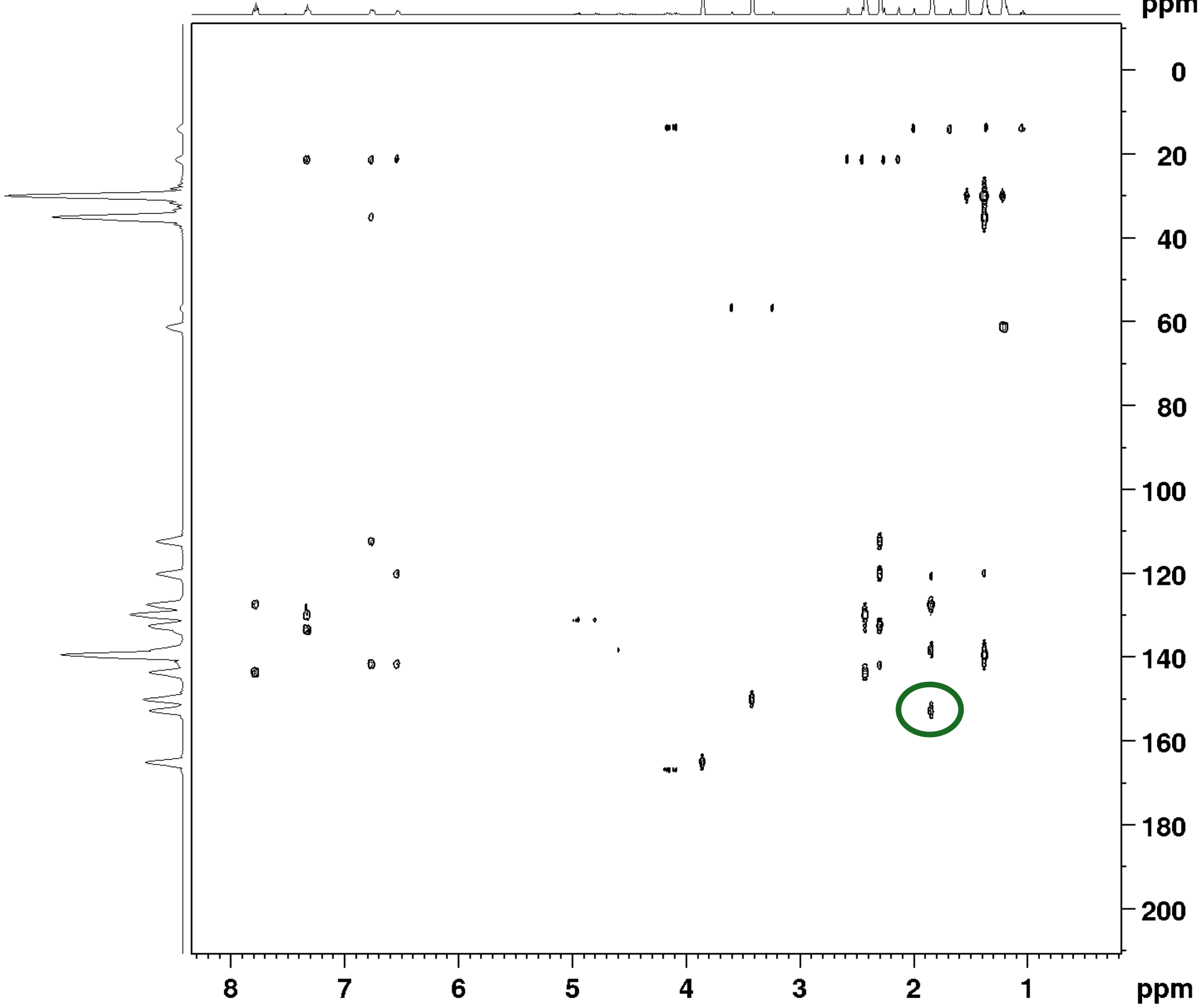

(+)-5-(*tert*-Butyl) 4-methyl 6-(2-(*tert*-butyl)-6-methoxy-4-methylphenoxy)-7-methyl-2-tosylisoindoline-4,5-dicarboxylate [(+)-3ag]

^{1}H NMR ($CDCl_3$, 400 MHz)

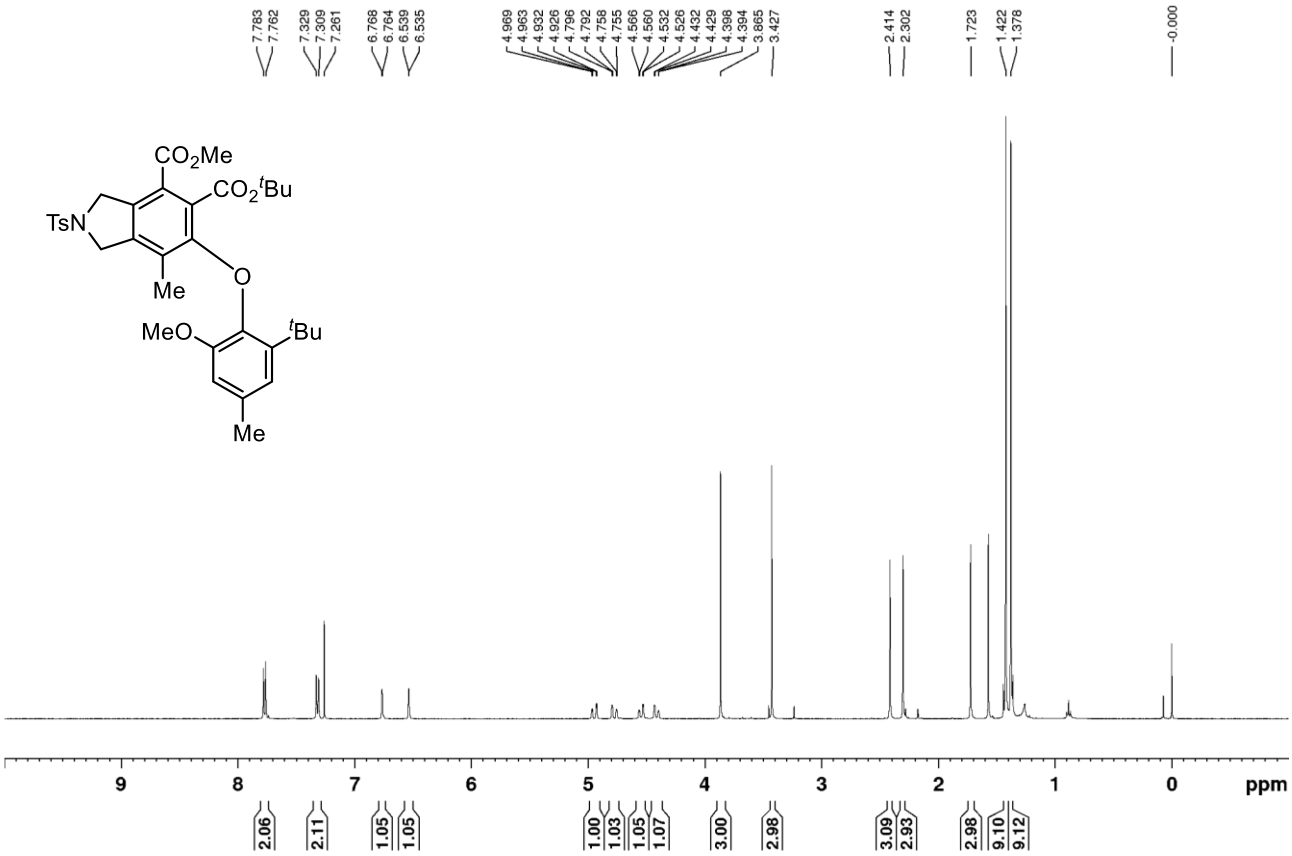

^{13}C NMR ($CDCl_3$, 101 MHz)

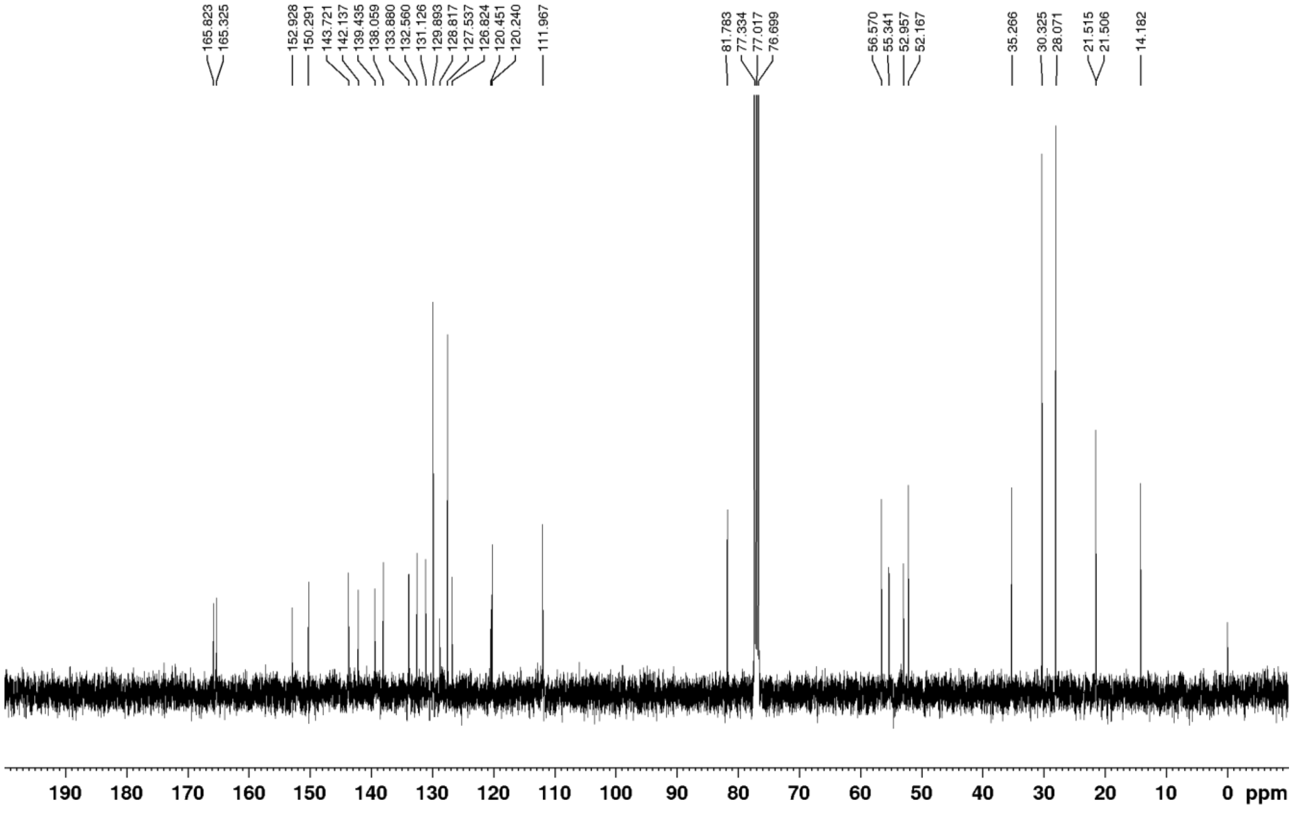

HMBC ($CDCl_3$, 400 MHz)

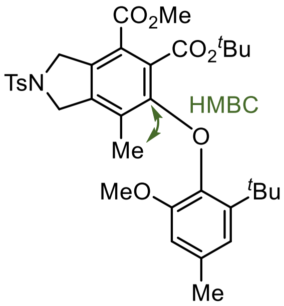

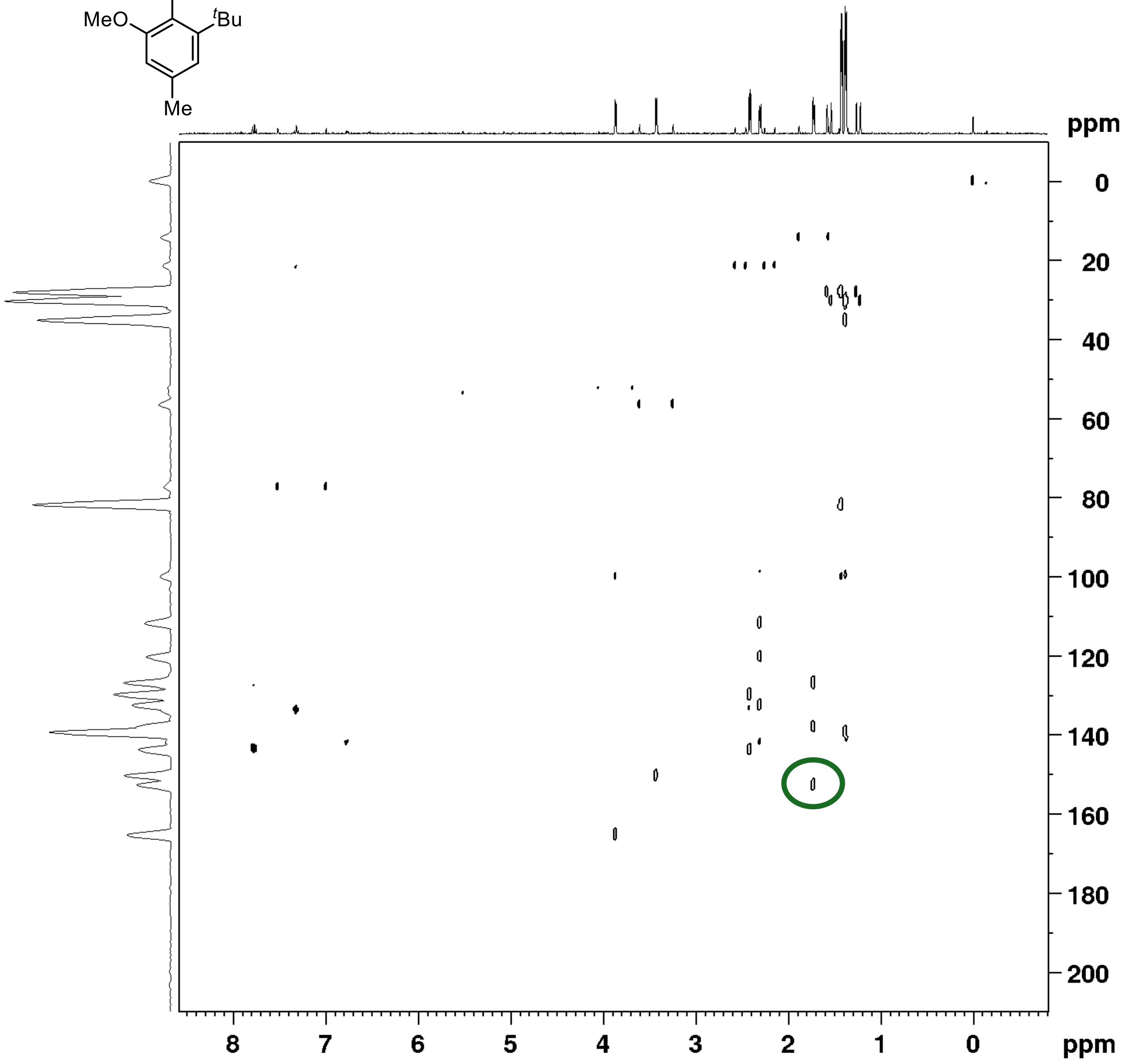

(+)-Methyl 5-acetyl-6-(2-(*tert*-butyl)-6-methoxy-4-methylphenoxy)-7-methyl-2-tosylisoindoline-4-carboxylate [(+)-3ah]

^{1}H NMR ($CDCl_3$, 400 MHz)

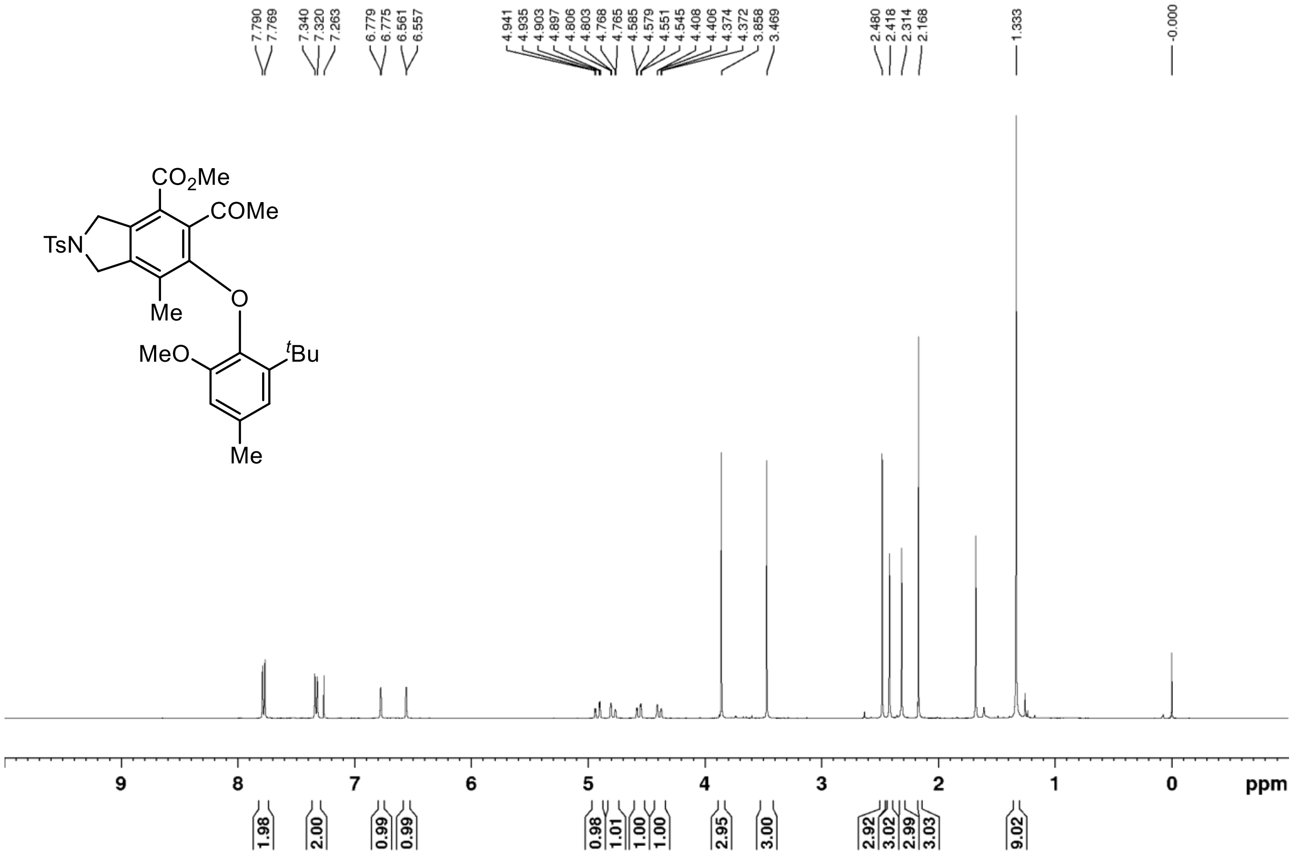

^{13}C NMR ($CDCl_3$, 101 MHz)

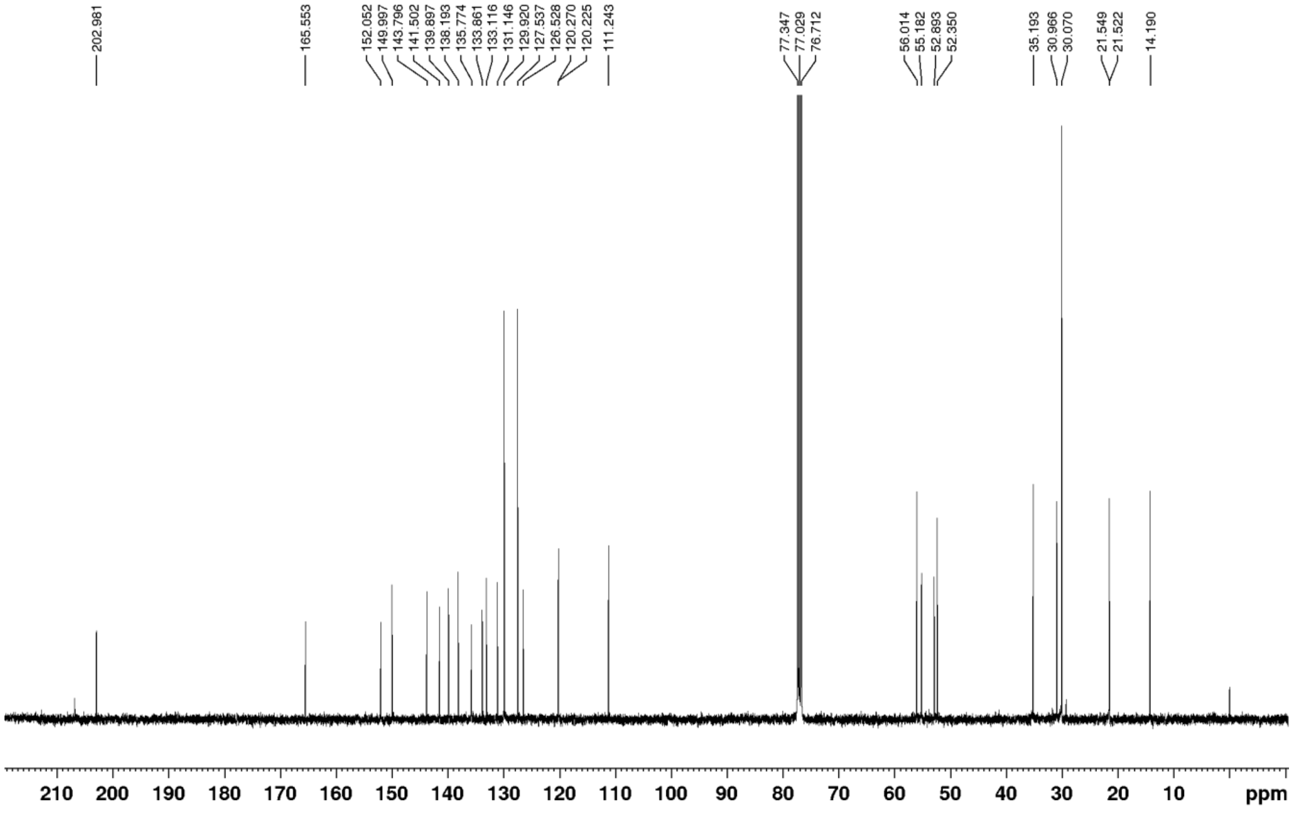

HMBC ($CDCl_3$, 400 MHz)

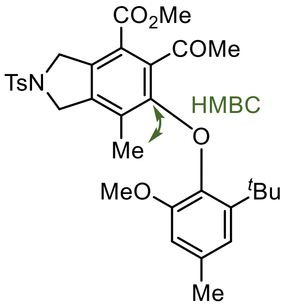

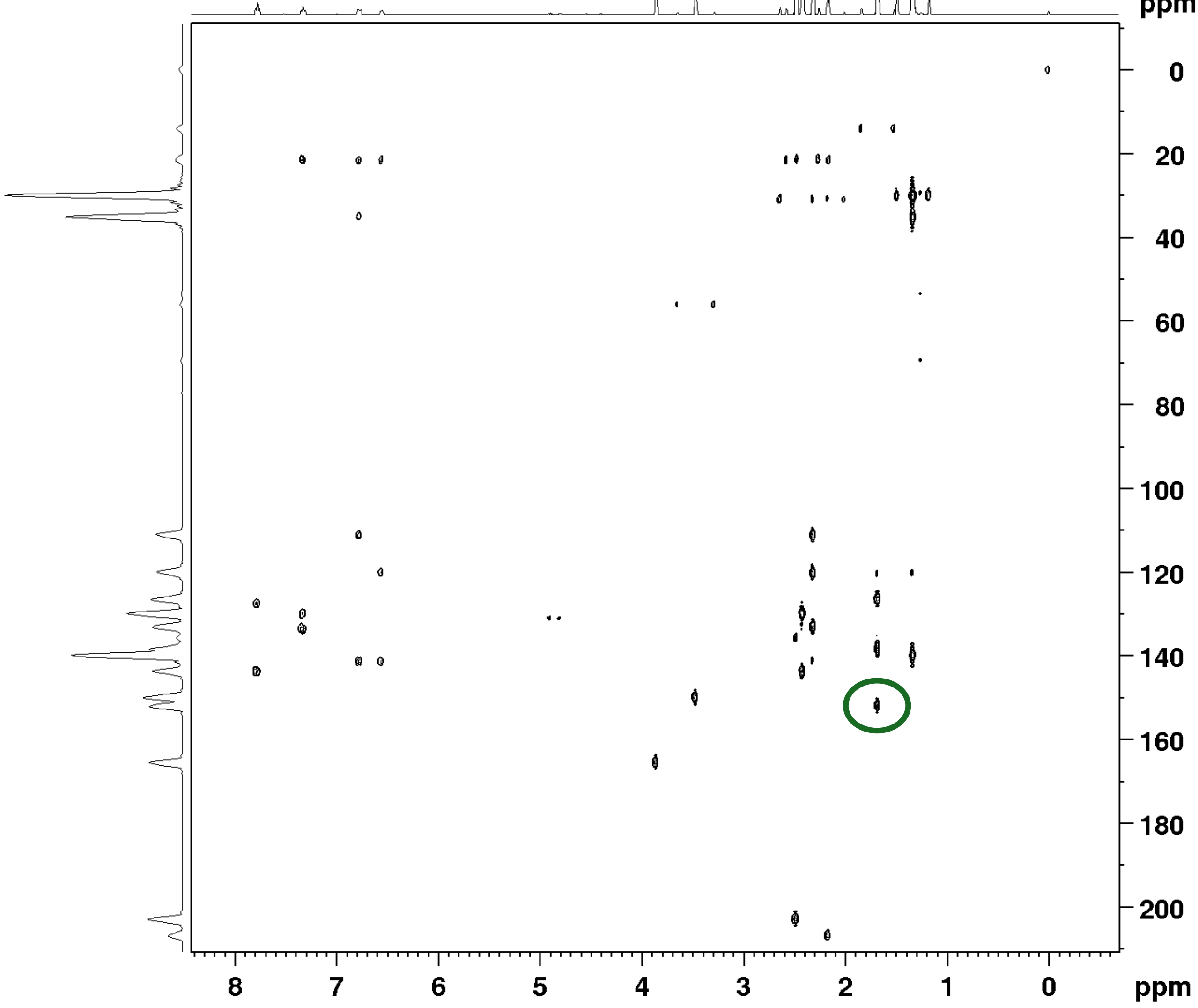

(+)-4-Ethyl 5-methyl 6-(2-(*tert*-butyl)-6-methoxy-4-methylphenoxy)-7-methyl-2-tosylisoindoline-4,5-dicarboxylate [(+)-3bb]

^{1}H NMR ($CDCl_3$, 400 MHz)

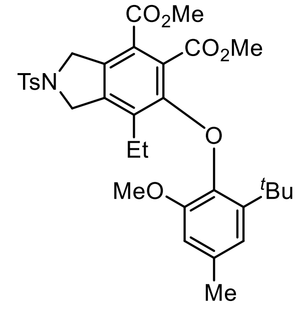

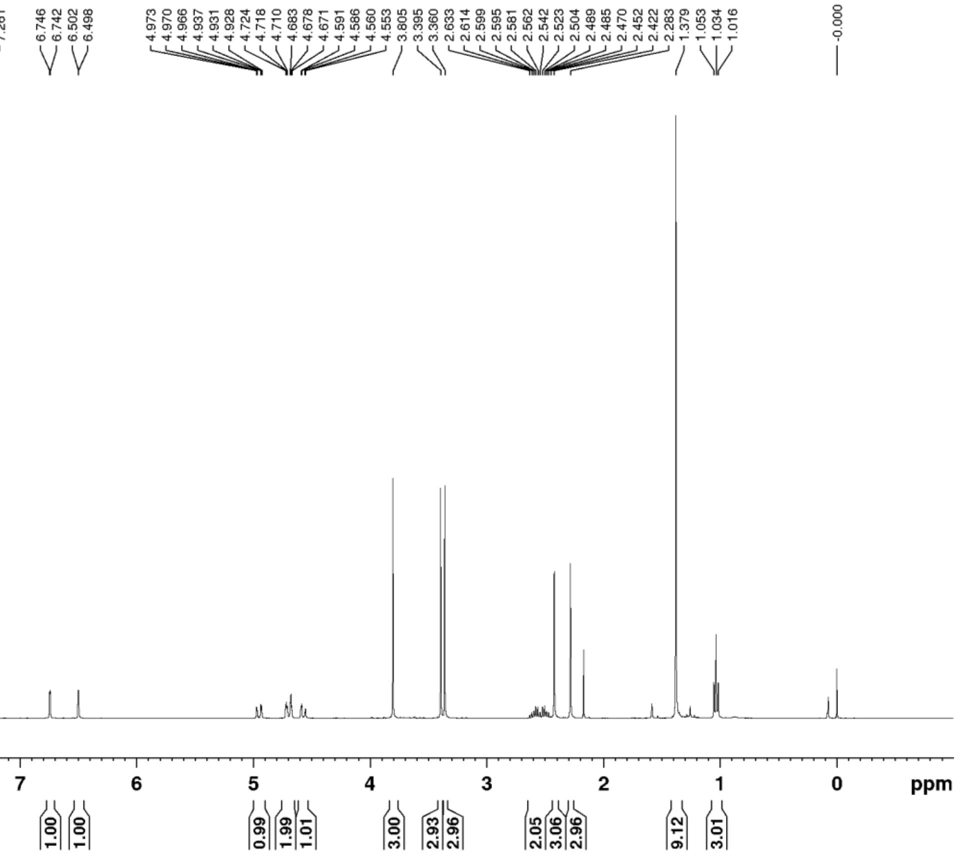

^{13}C NMR ($CDCl_3$, 101 MHz)

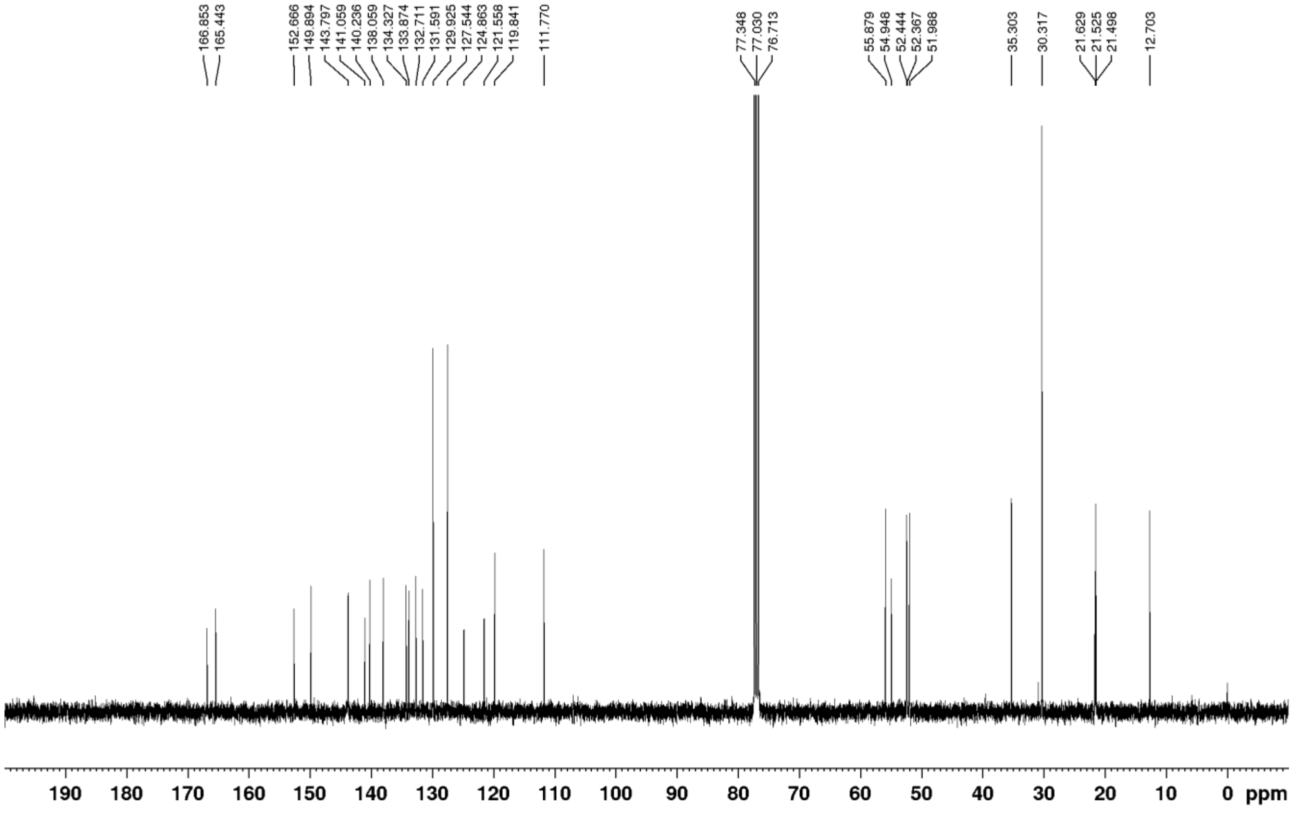

HMBC ($CDCl_3$, 400 MHz)

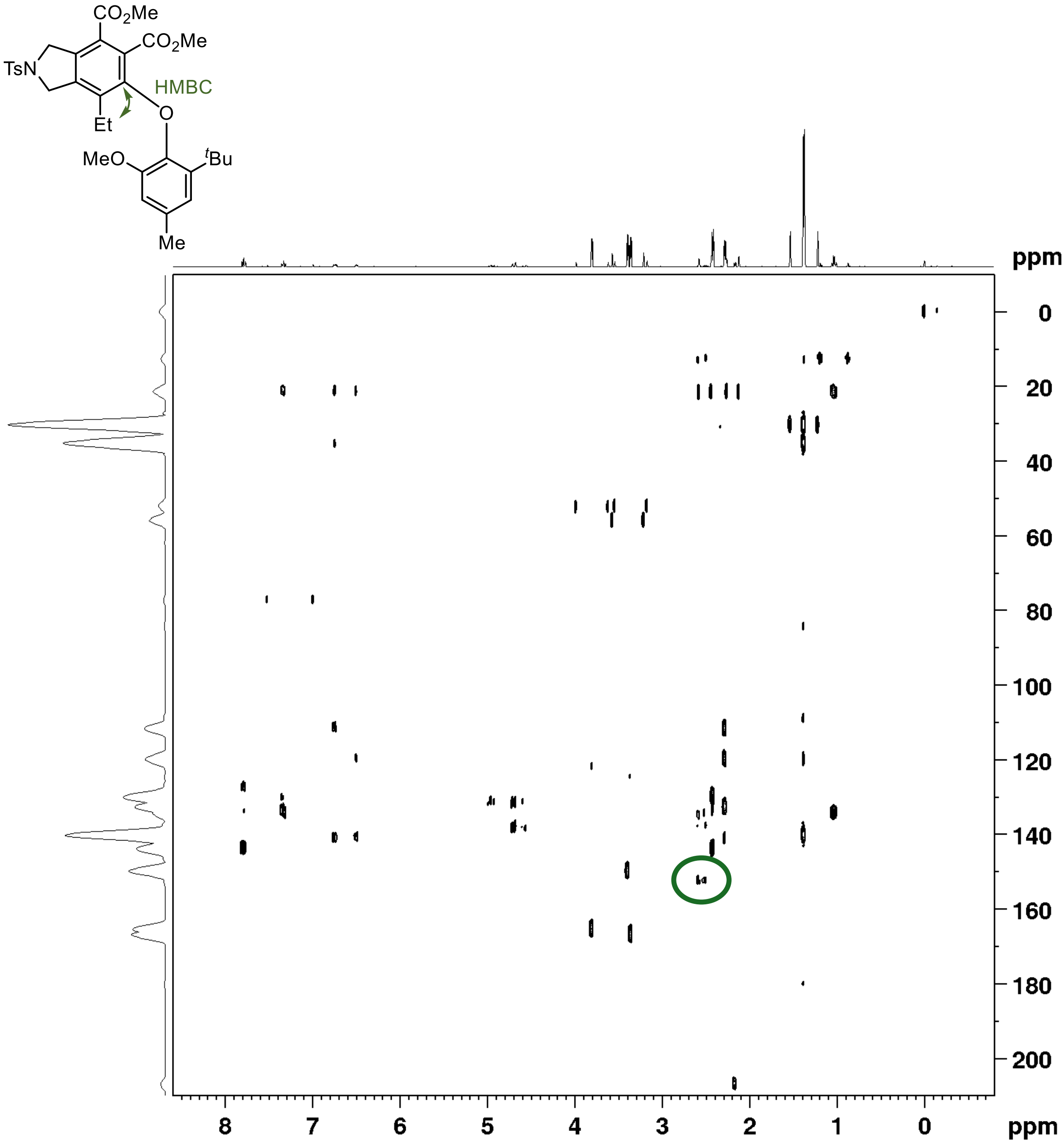

(+)-4-Ethyl 5-methyl 6-(2-(*tert*-butyl)-6-methoxy-4-methylphenoxy)-7-methyl-2-tosylisoindoline-4,5-dicarboxylate [(+)-3cb]

^{1}H NMR ($CDCl_3$, 400 MHz)

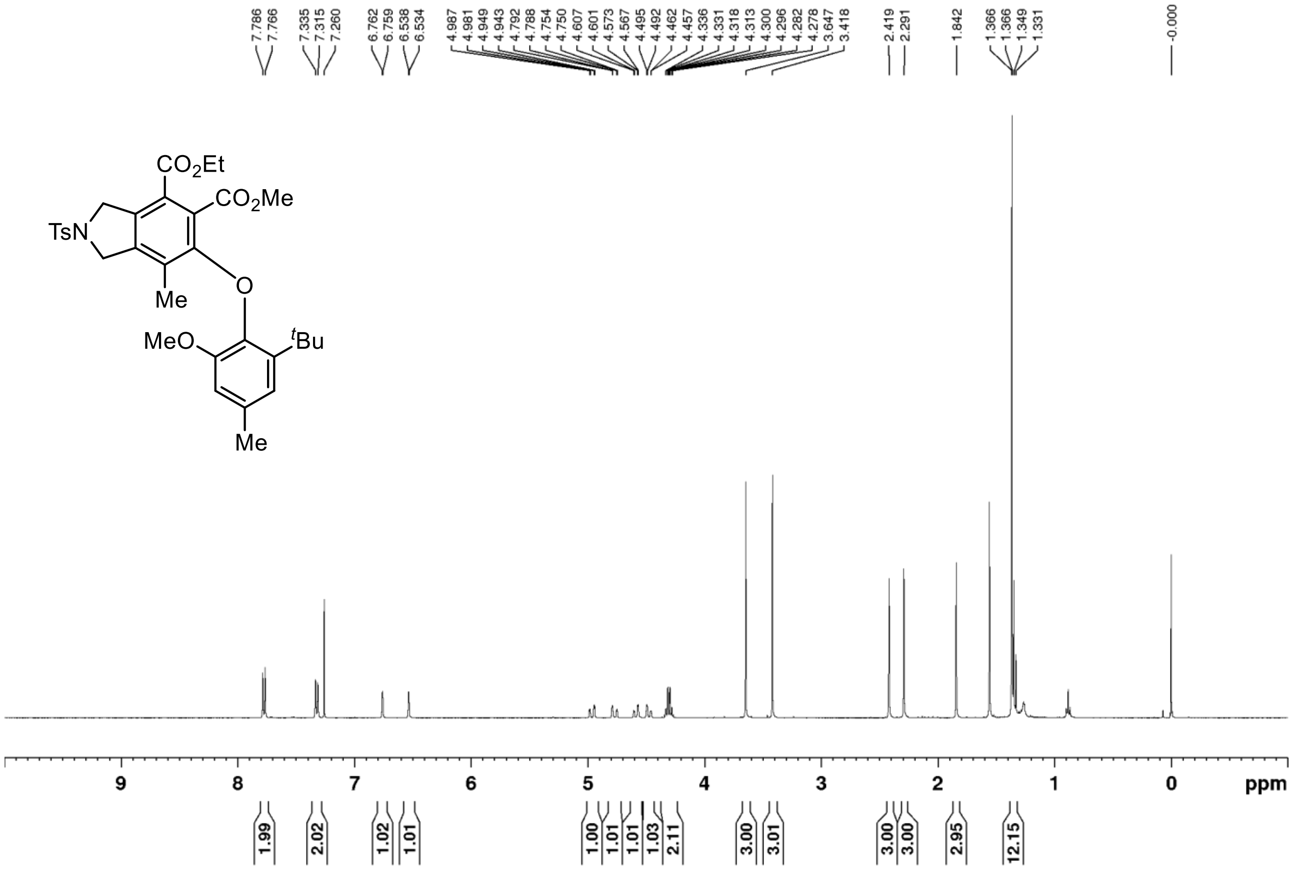

^{13}C NMR ($CDCl_3$, 101 MHz)

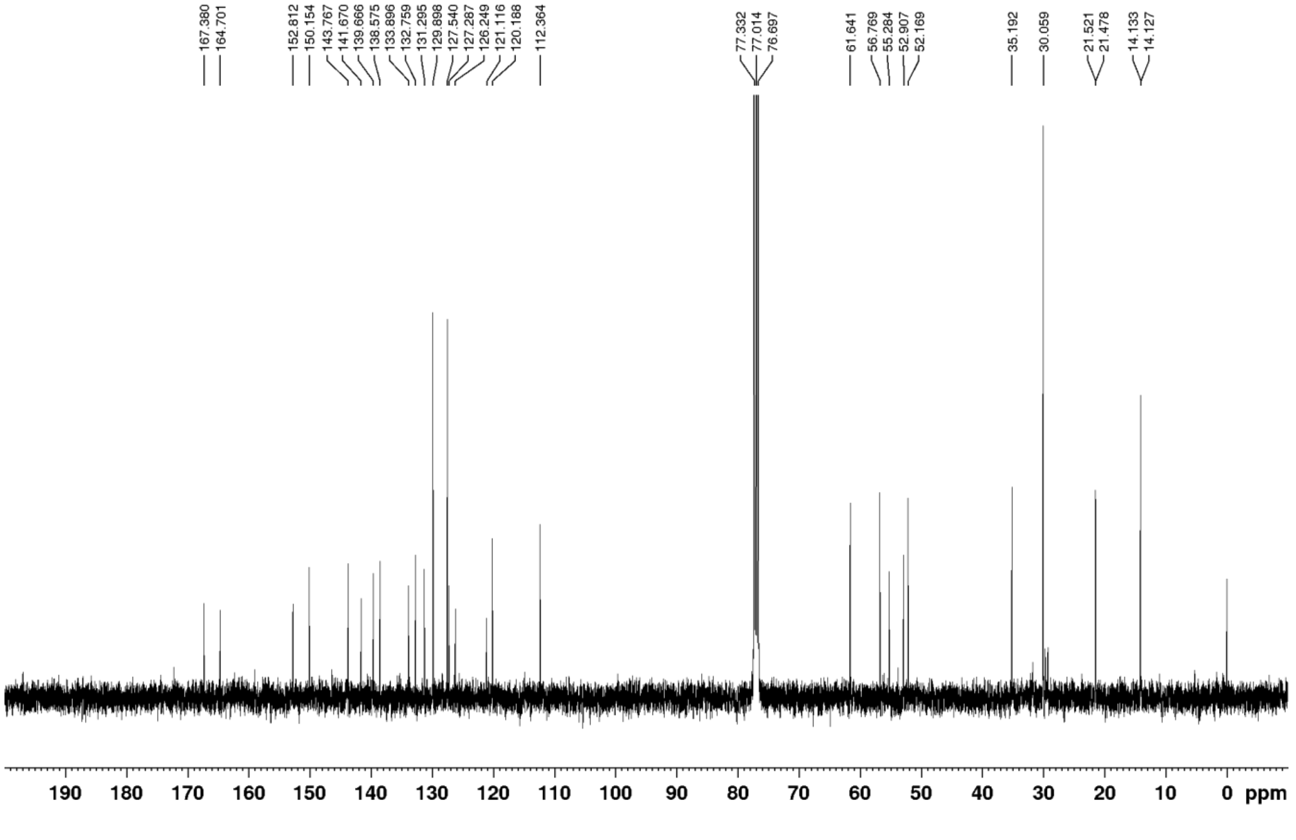

HMBC ($CDCl_3$, 400 MHz)

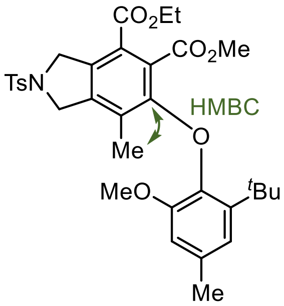

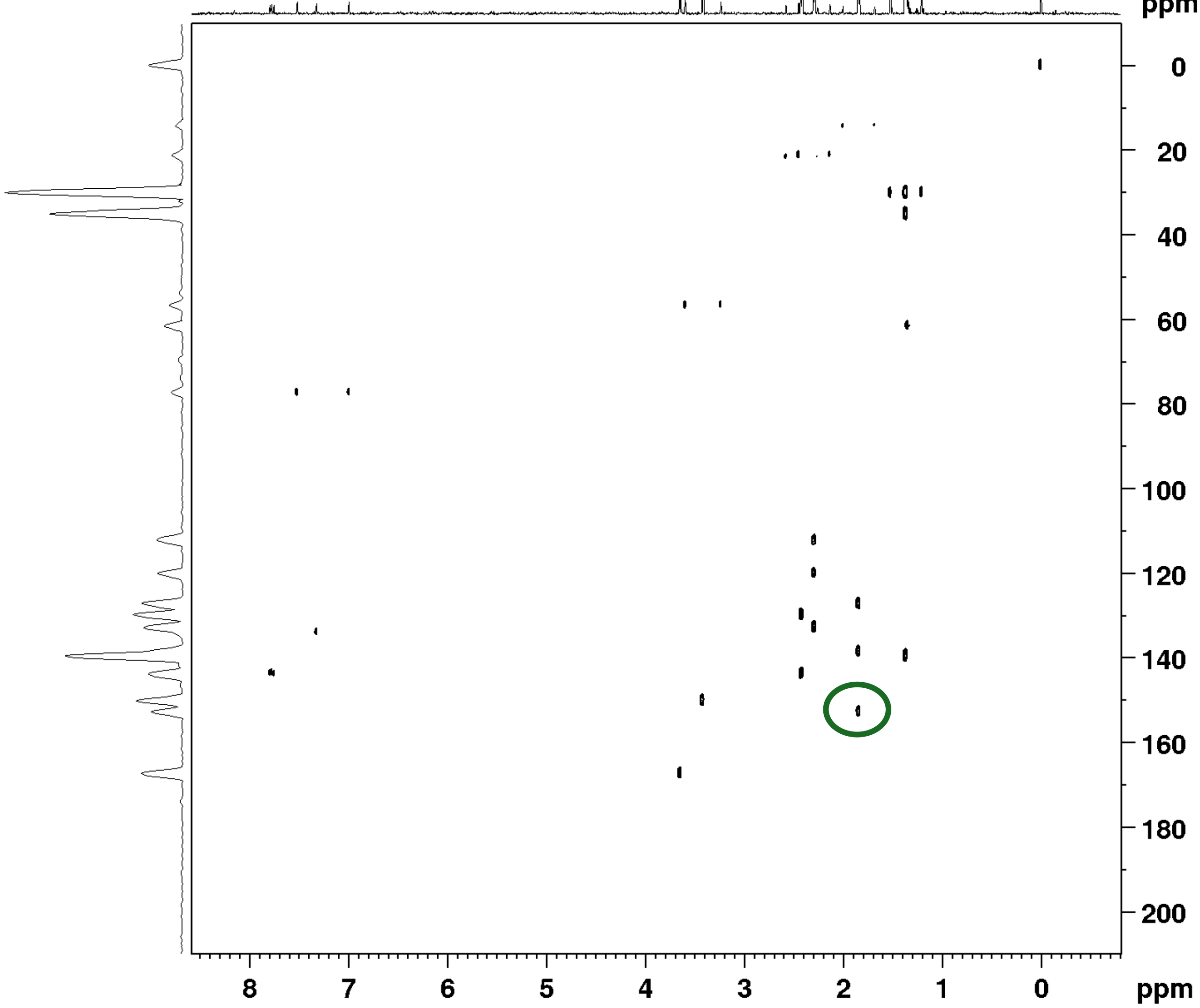

(+)-Methyl 4-acetyl-6-(2-(*tert*-butyl)-6-methoxy-4-methylphenoxy)-7-methyl-2-tosylisoindoline-5-carboxylate [(+)-3db]

^{1}H NMR ($CDCl_3$, 400 MHz)

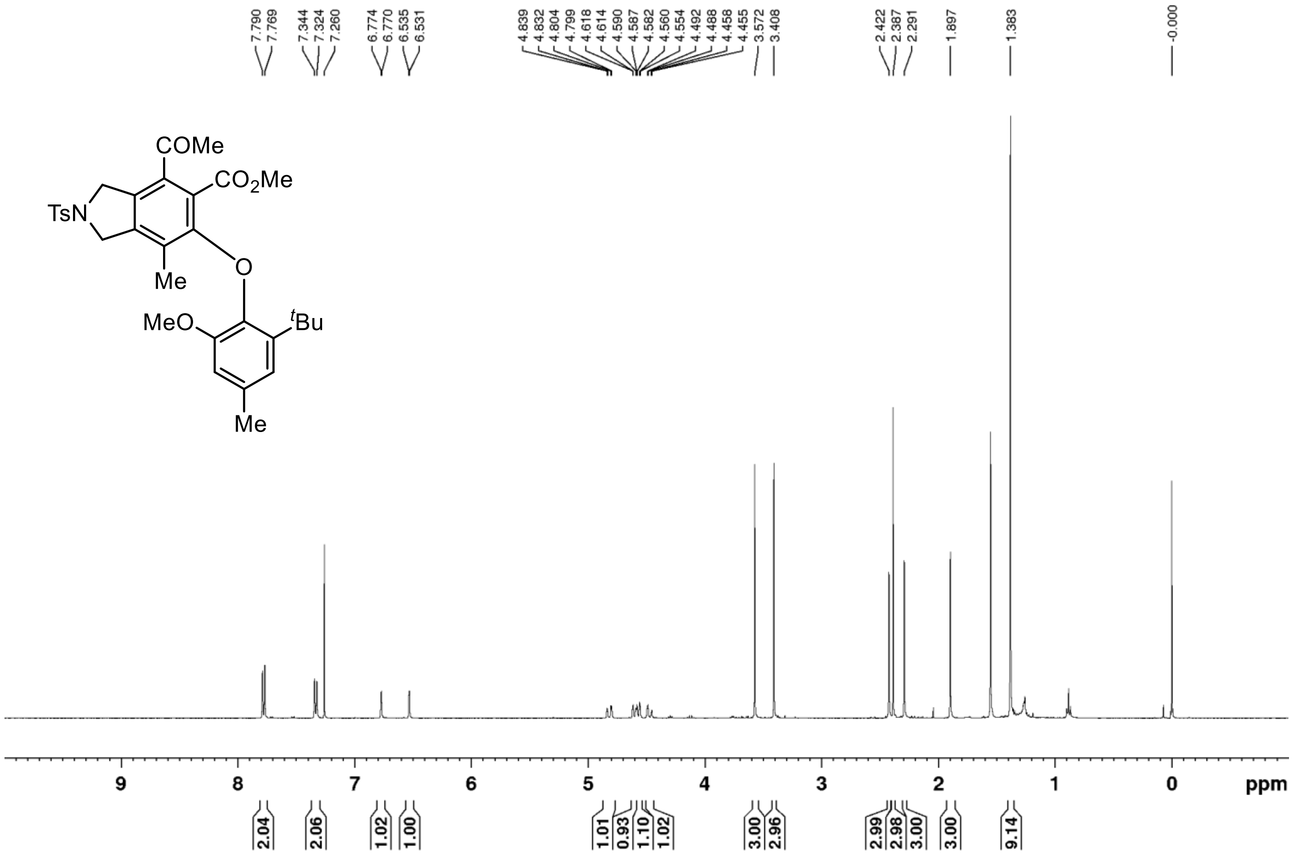

^{13}C NMR ($CDCl_3$, 101 MHz)

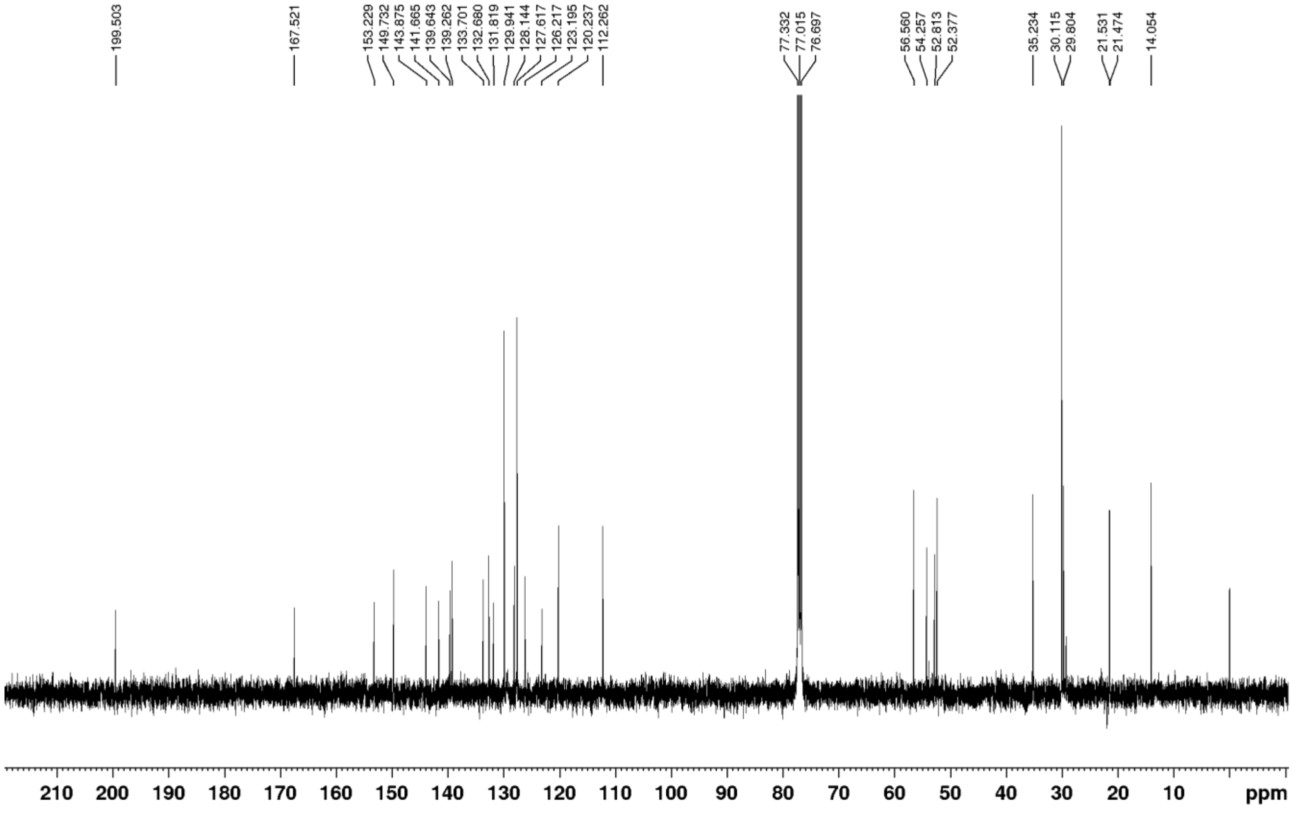

HMBC ($CDCl_3$, 400 MHz)

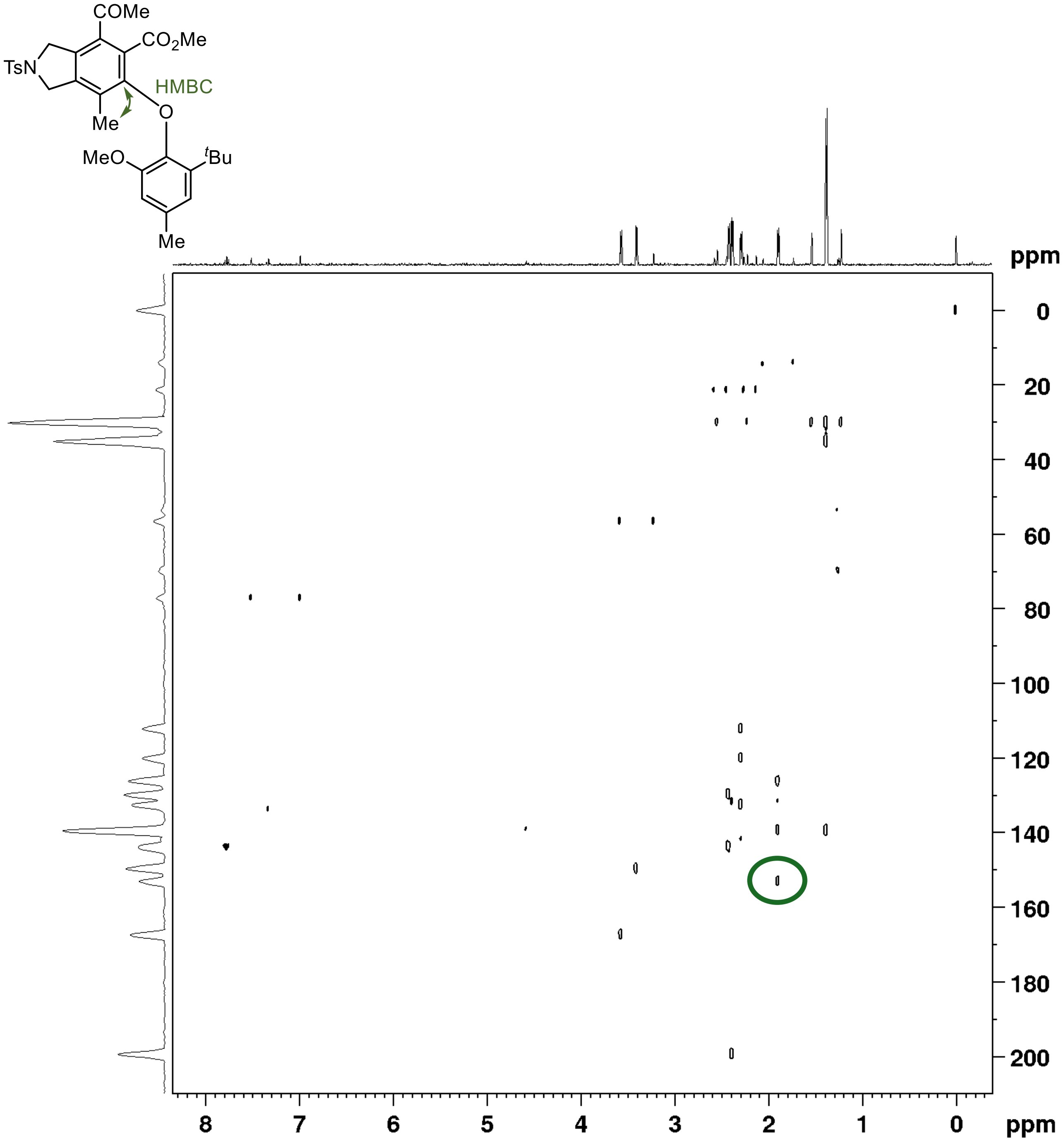

(+)-Dimethyl 6-(2-(*tert*-butyl)-6-methoxy-4-methylphenoxy)-7-methyl-1,3-dihydroisobenzofuran-4,5-dicarboxylate [(+)-3eb]

^{1}H NMR ($CDCl_3$, 400 MHz)

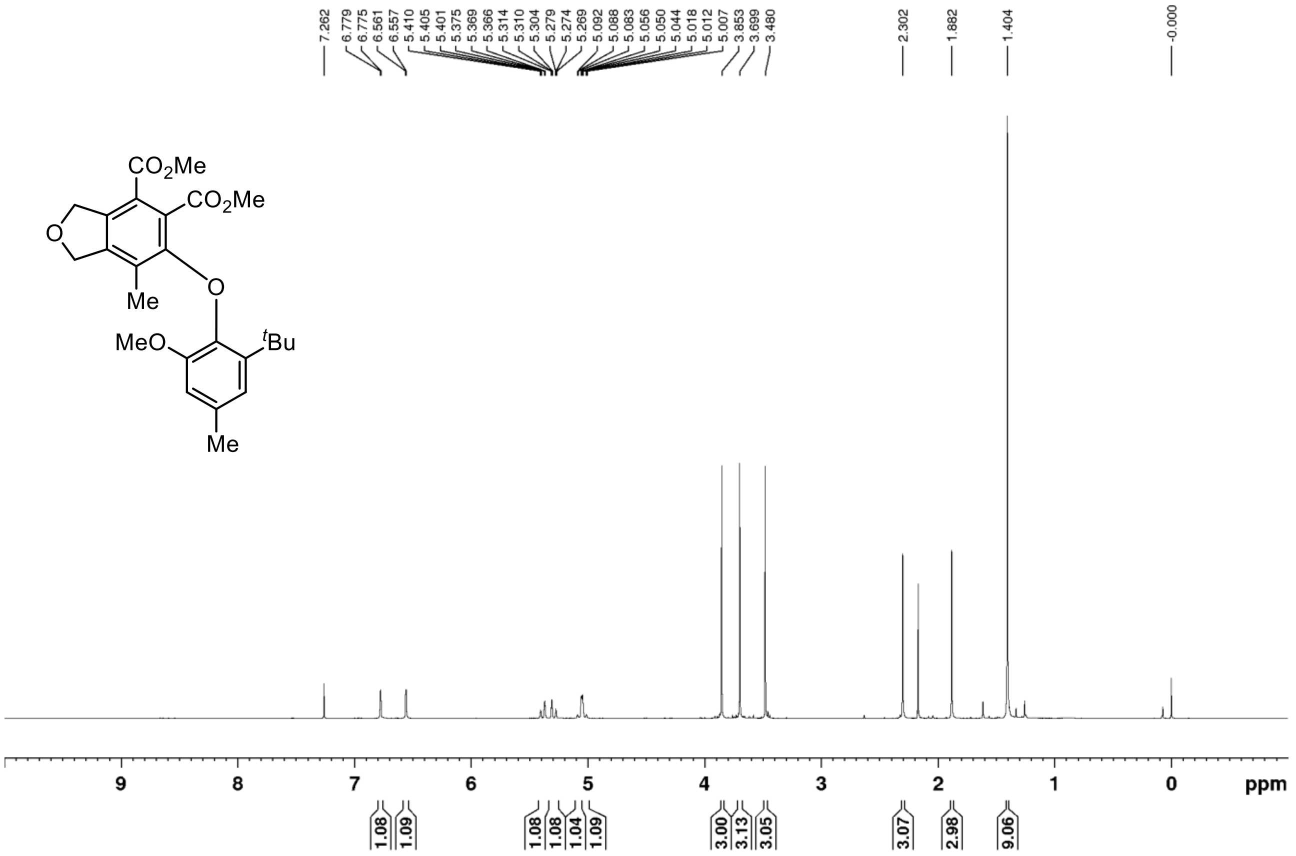

^{13}C NMR ($CDCl_3$, 101 MHz)

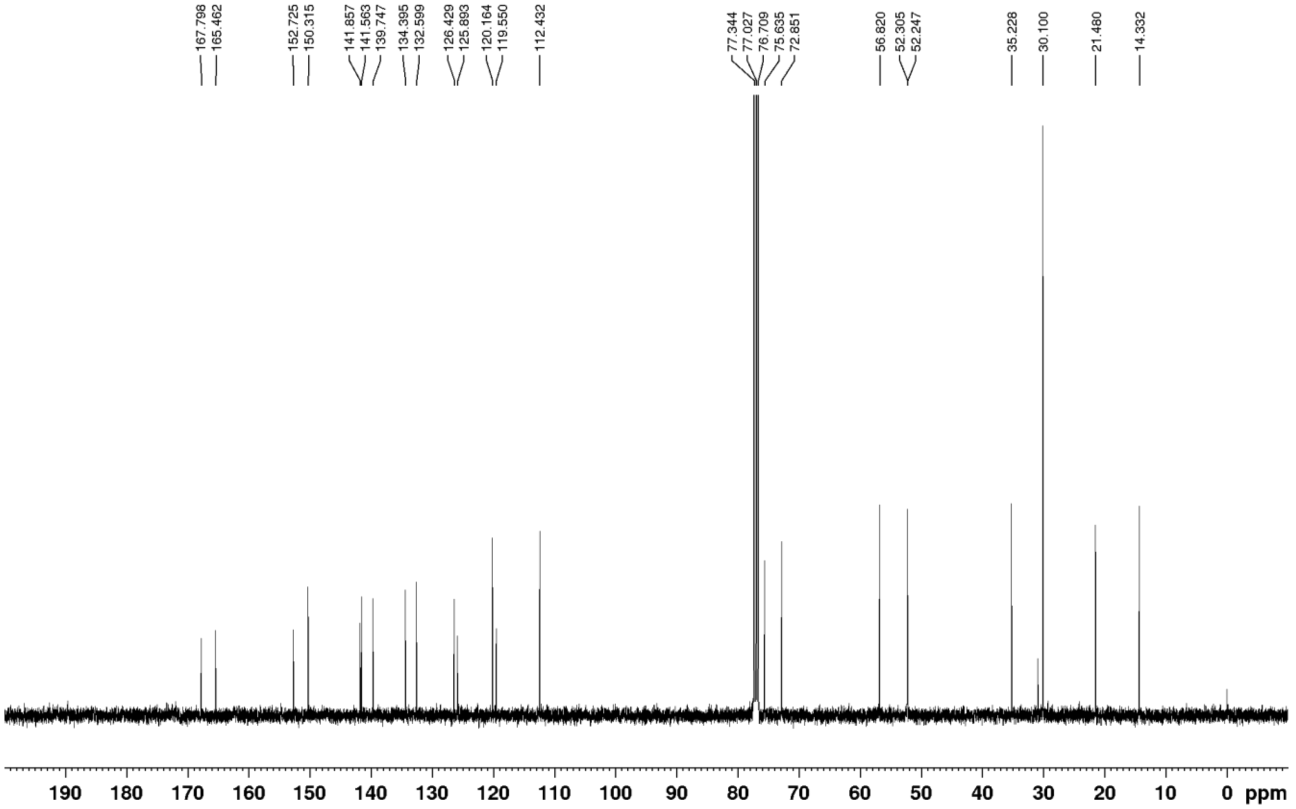

HMBC ($CDCl_3$, 400 MHz)

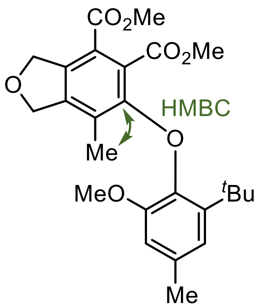

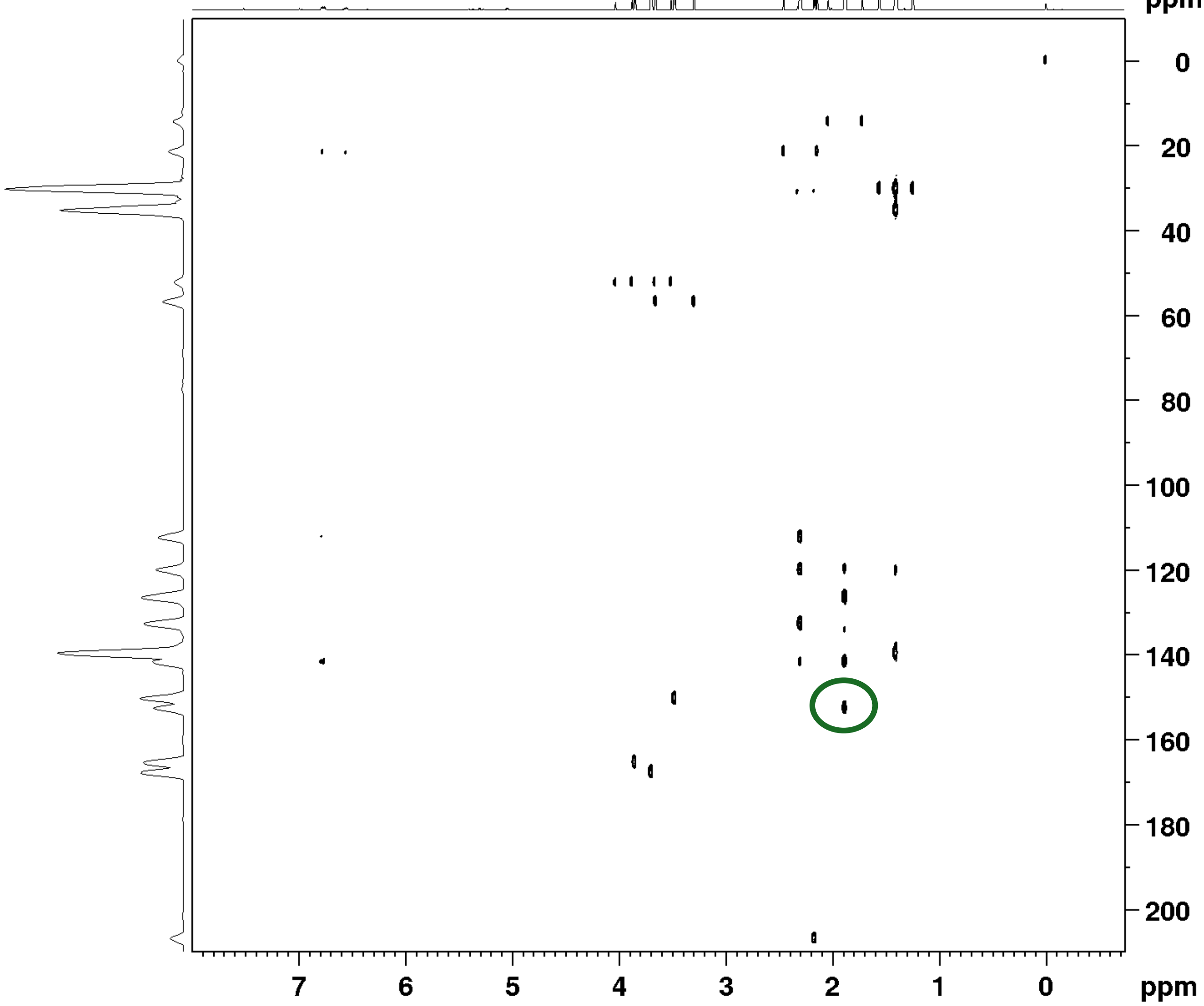

(–)-Dimethyl 5-(2-(*tert*-butyl)-6-methoxy-4-methylphenoxy)-7-methyl-1,3-dihydroisobenzofuran-4,6-dicarboxylate [(–)-4eb]

^{1}H NMR ($CDCl_3$, 400 MHz)

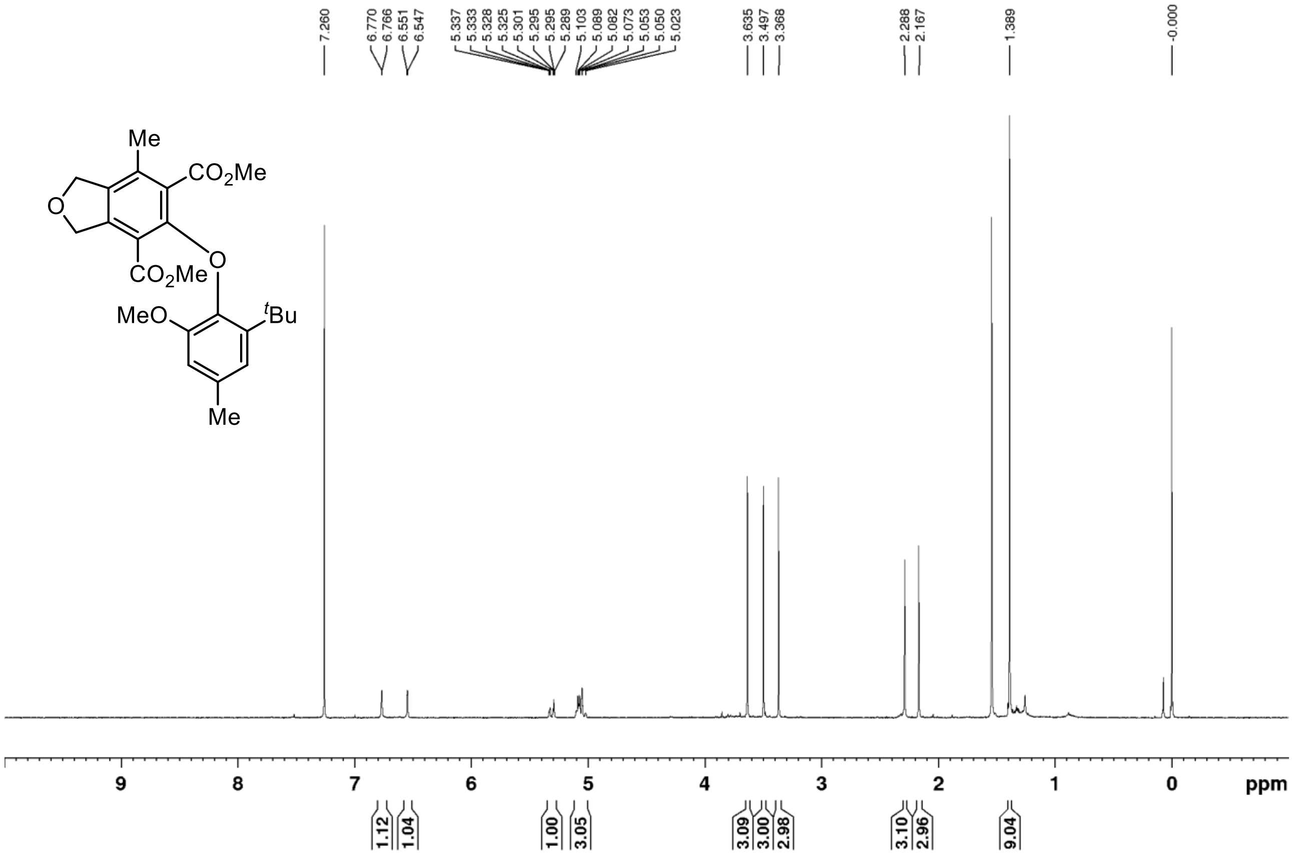

^{13}C NMR ($CDCl_3$, 101 MHz)

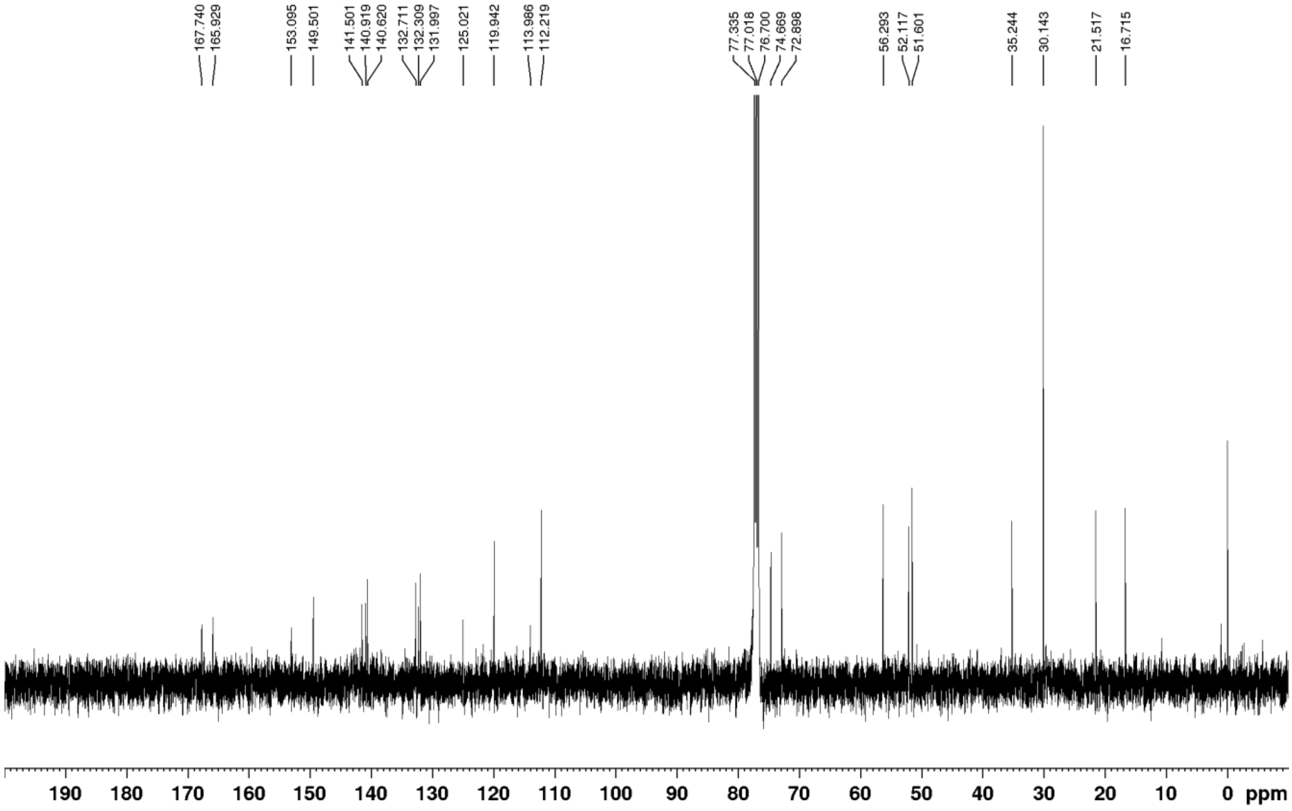

NOESY ($CDCl_3$, 400 MHz)

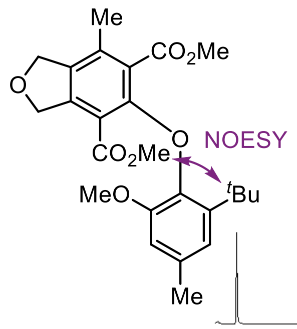

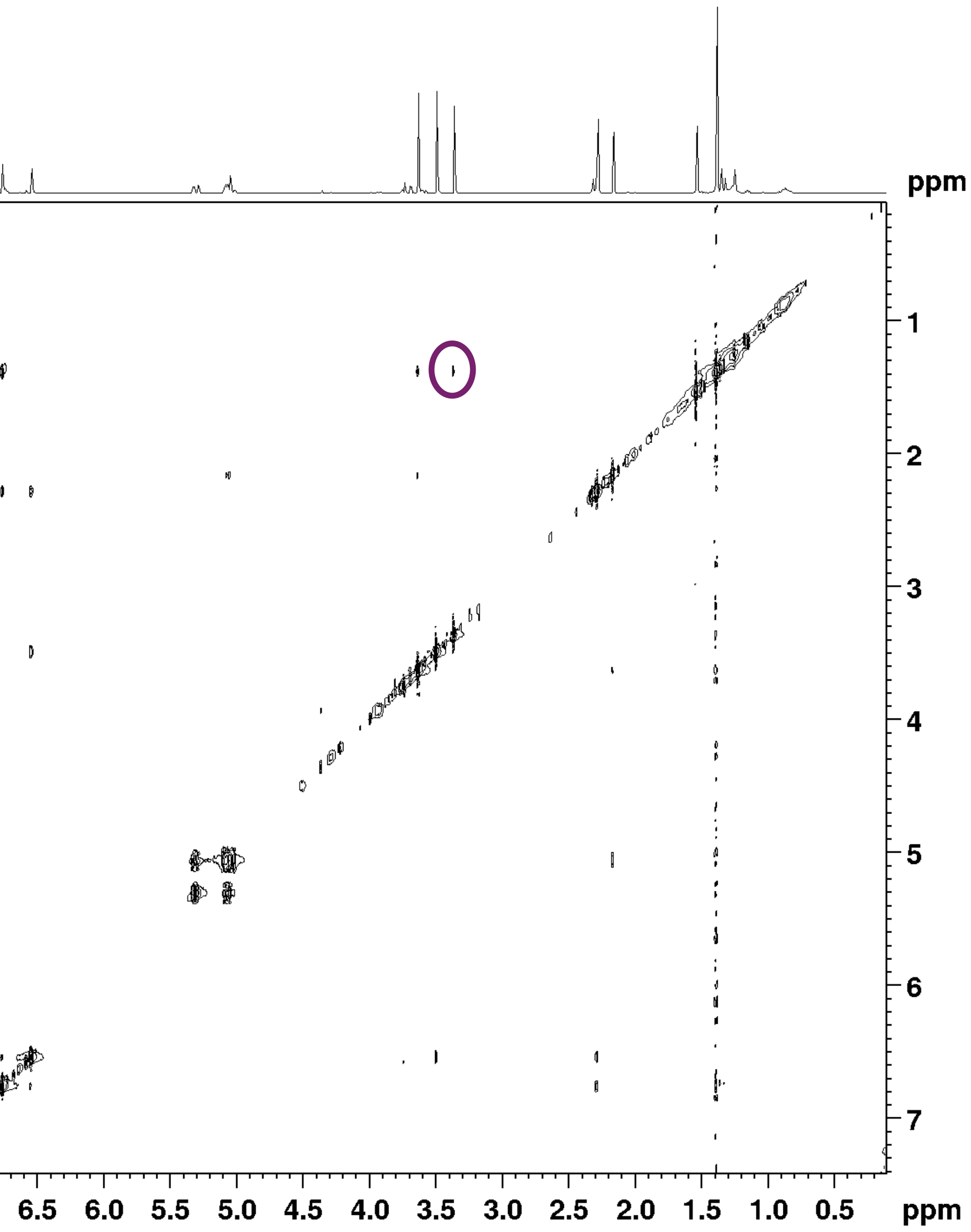

(+)-Tetramethyl 6-(2-(*tert*-butyl)-6-methoxy-4-methylphenoxy)-7-methyl-1,3-dihydro-2H-indene-2,2,4,5-tetracarboxylate [(+)-3fb]

^{1}H NMR ($CDCl_3$, 400 MHz)

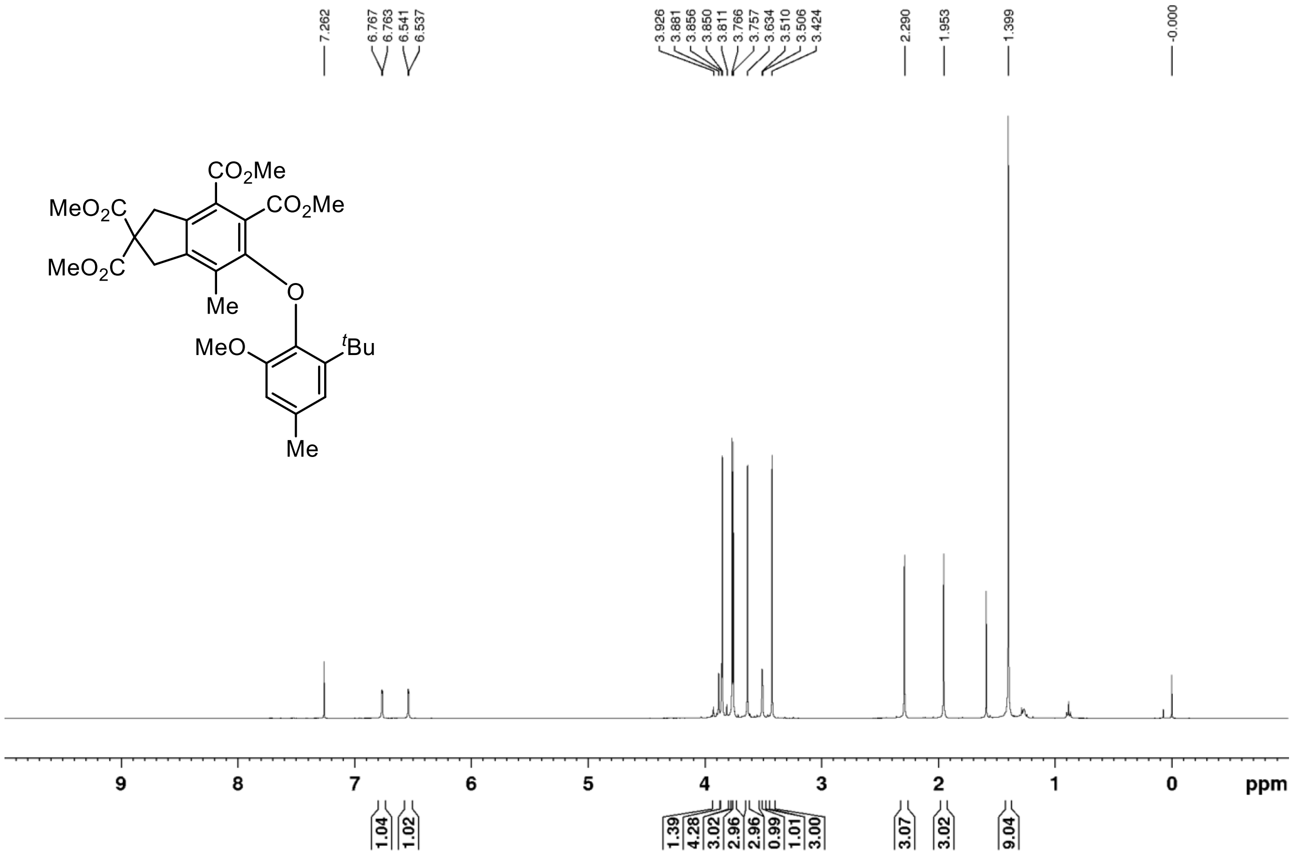

^{13}C NMR ($CDCl_3$, 101 MHz)

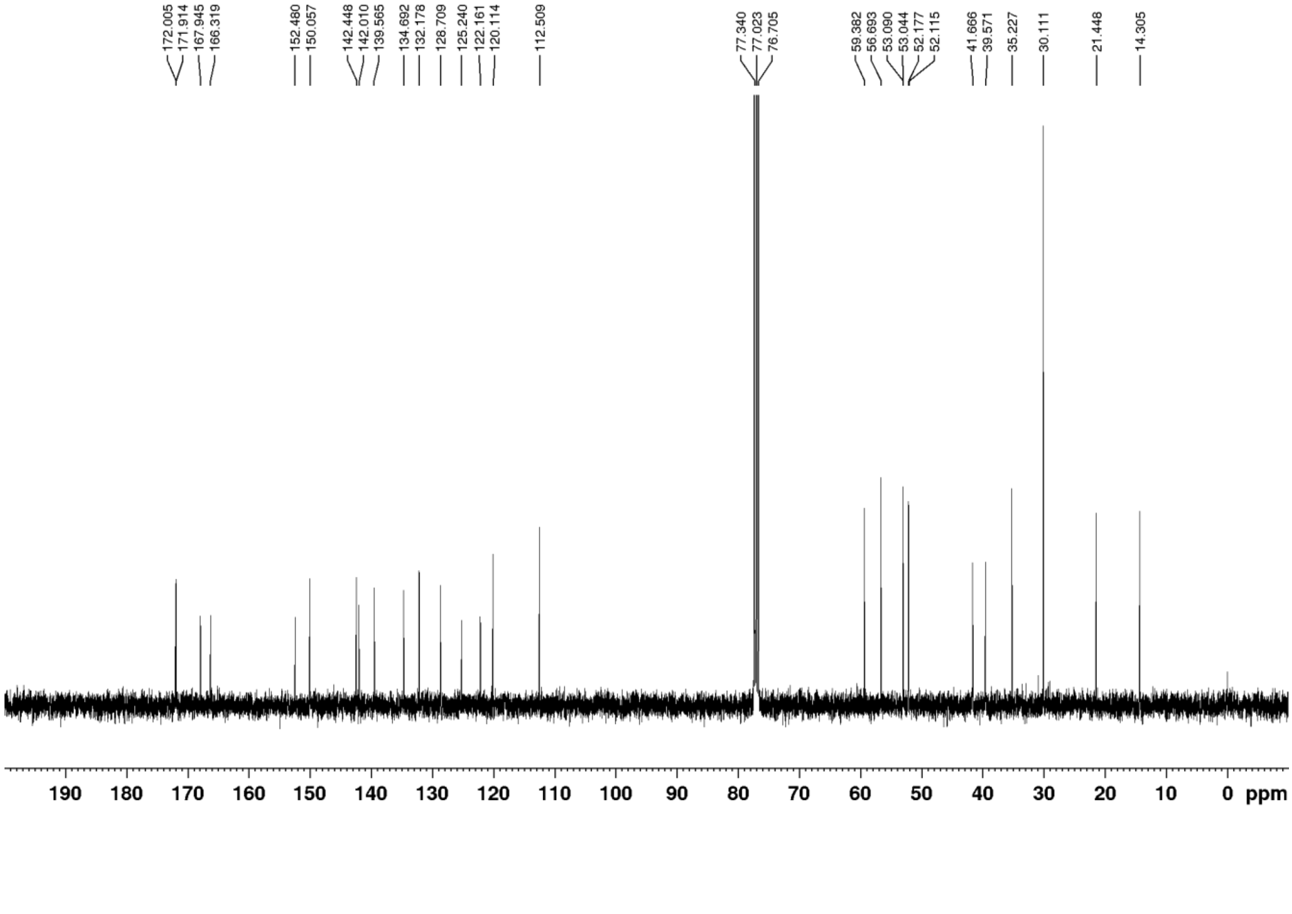

HMBC ($CDCl_3$, 400 MHz)

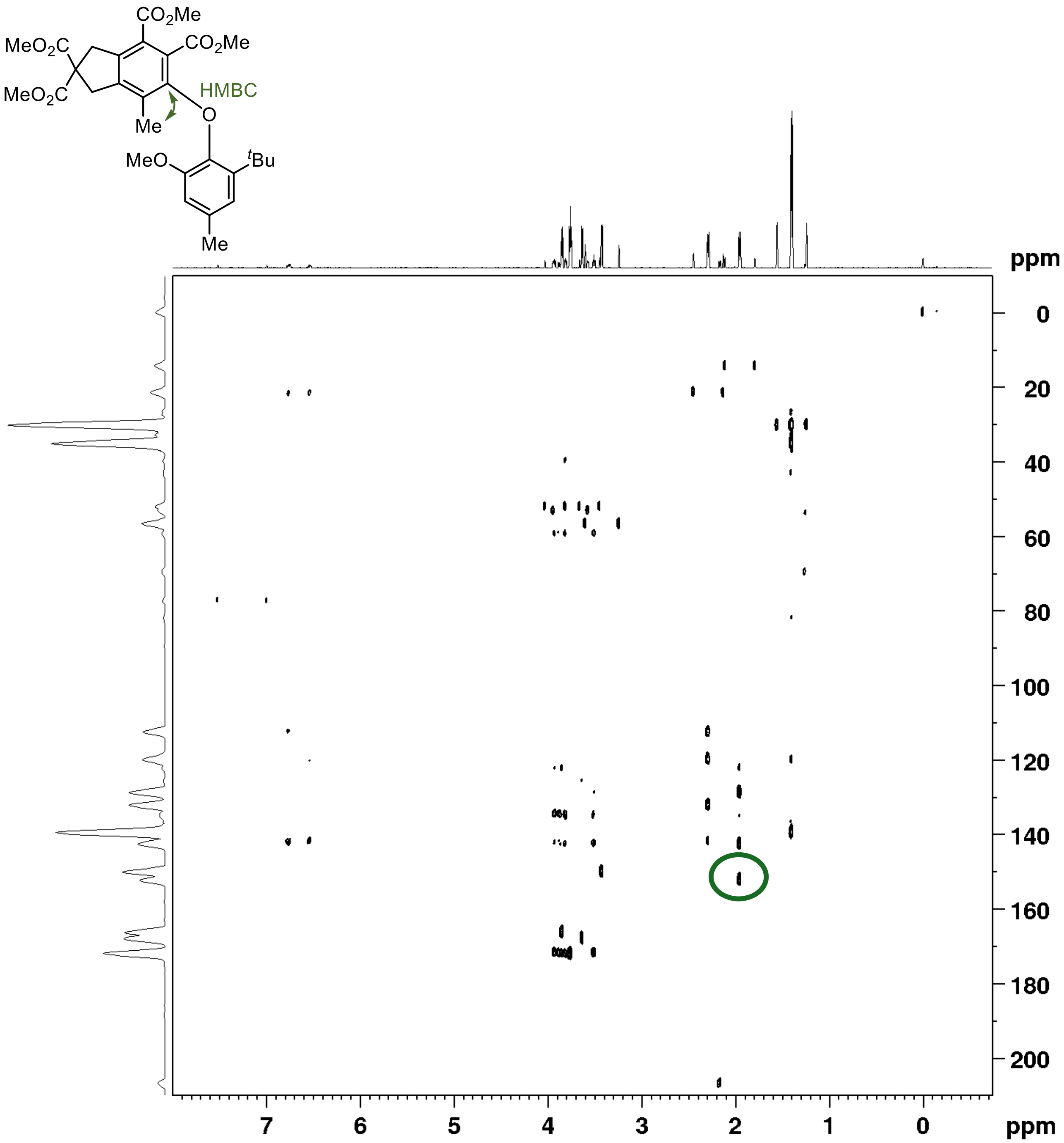

(+)-Dimethyl 6-(2-((3*r*,5*r*,7*r*)-adamantan-1-yl)-4,6-dimethylphenoxy)-7-methyl-2-tosylisoindoline-4,5-dicarboxylate [(+)-3ai]

^{1}H NMR ($CDCl_3$, 400 MHz)

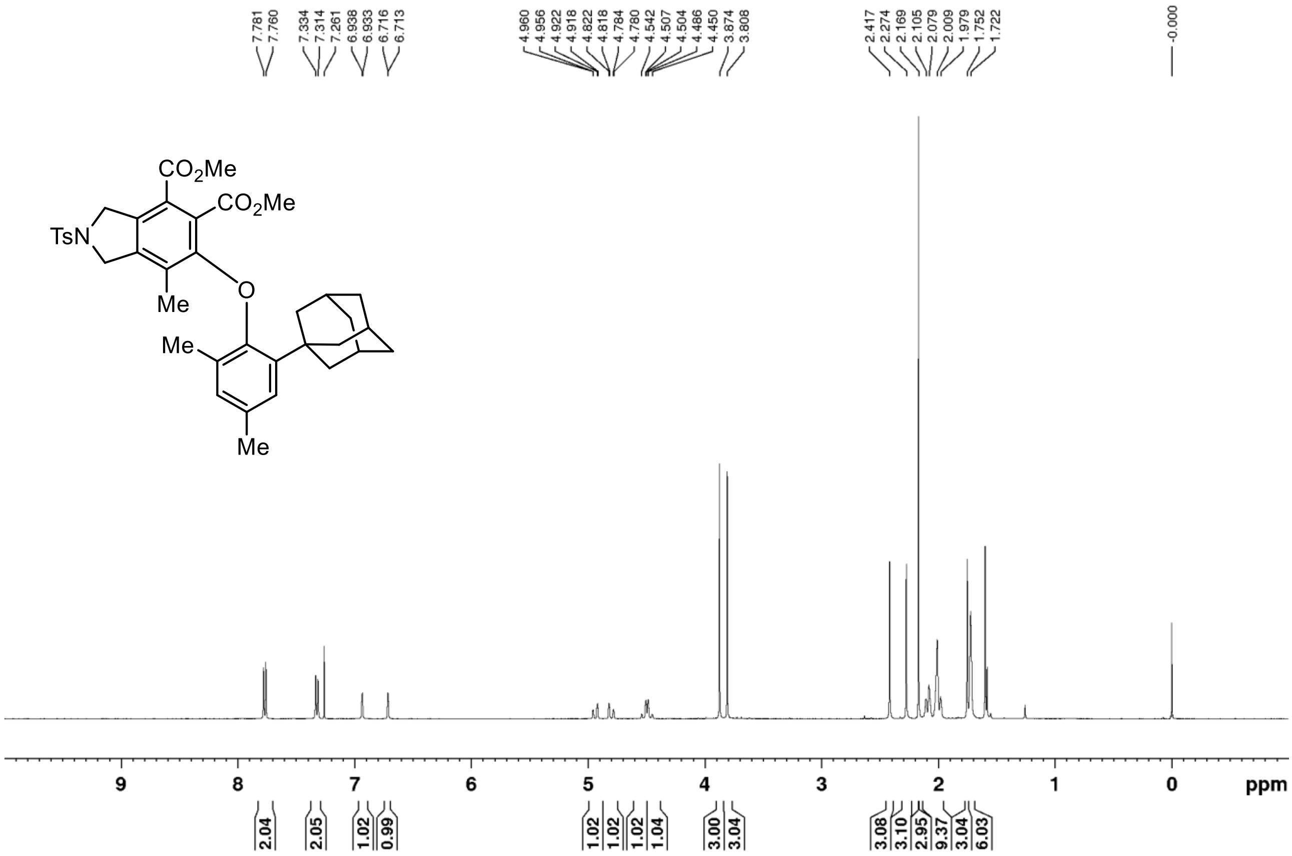

^{13}C NMR ($CDCl_3$, 101 MHz)

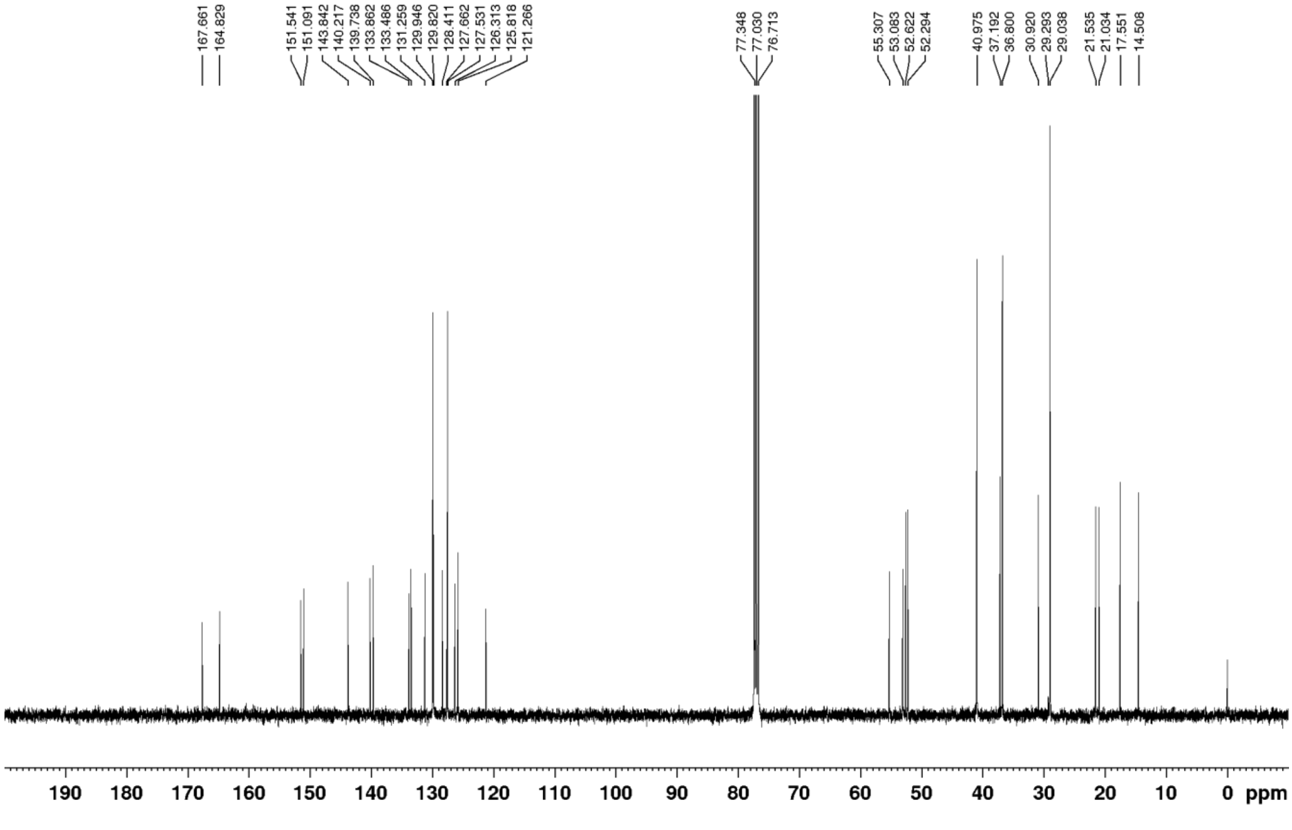

HMBC ($CDCl_3$, 400 MHz)

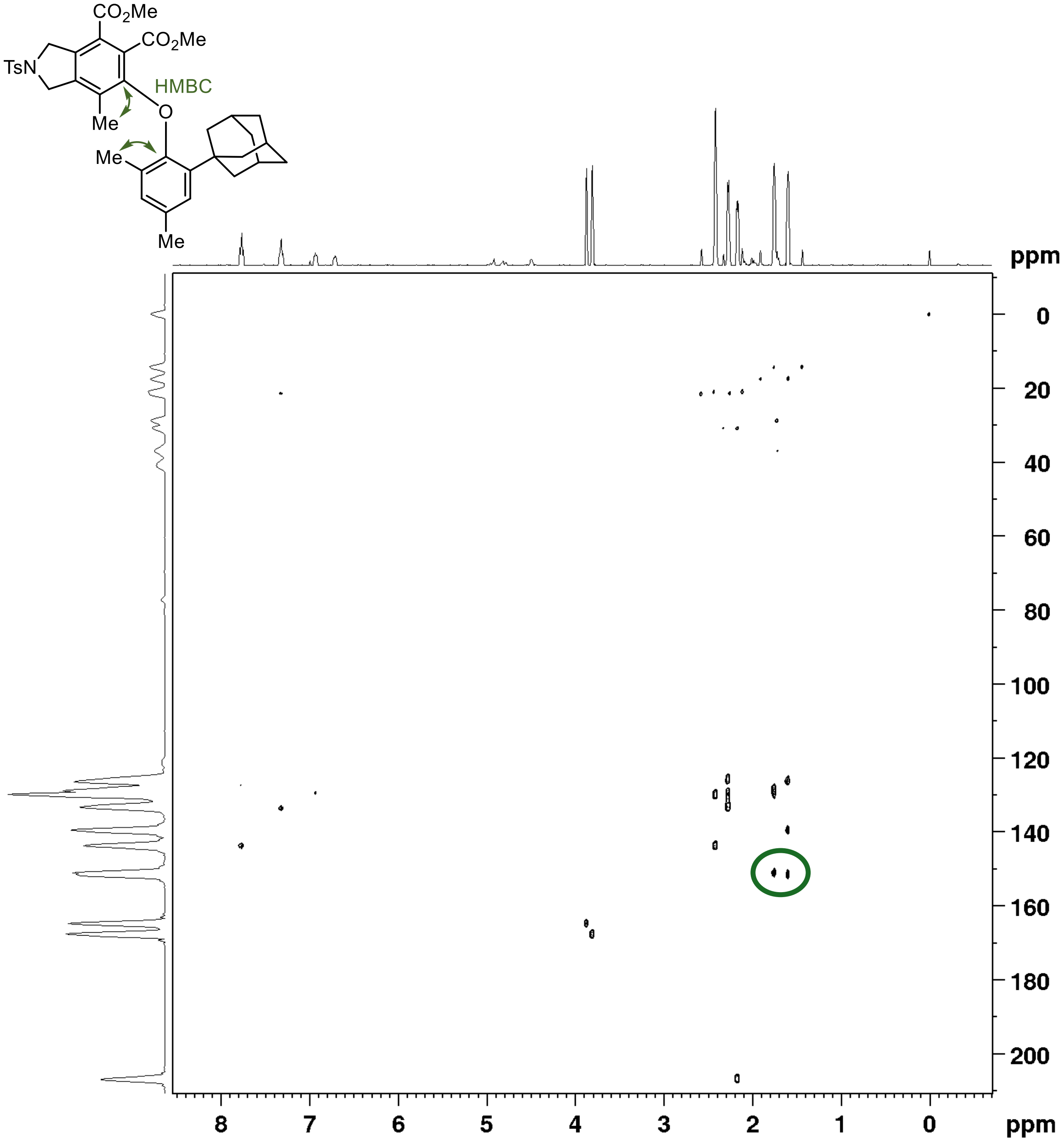

(+)-Dimethyl 6-(2-((3*r*,5*r*,7*r*)-adamantan-1-yl)-4,6-dimethylphenoxy)-7-methyl-2-tosylisoindoline-4,5-dicarboxylate [(+)-4ai]

^{1}H NMR ($CDCl_3$, 400 MHz)

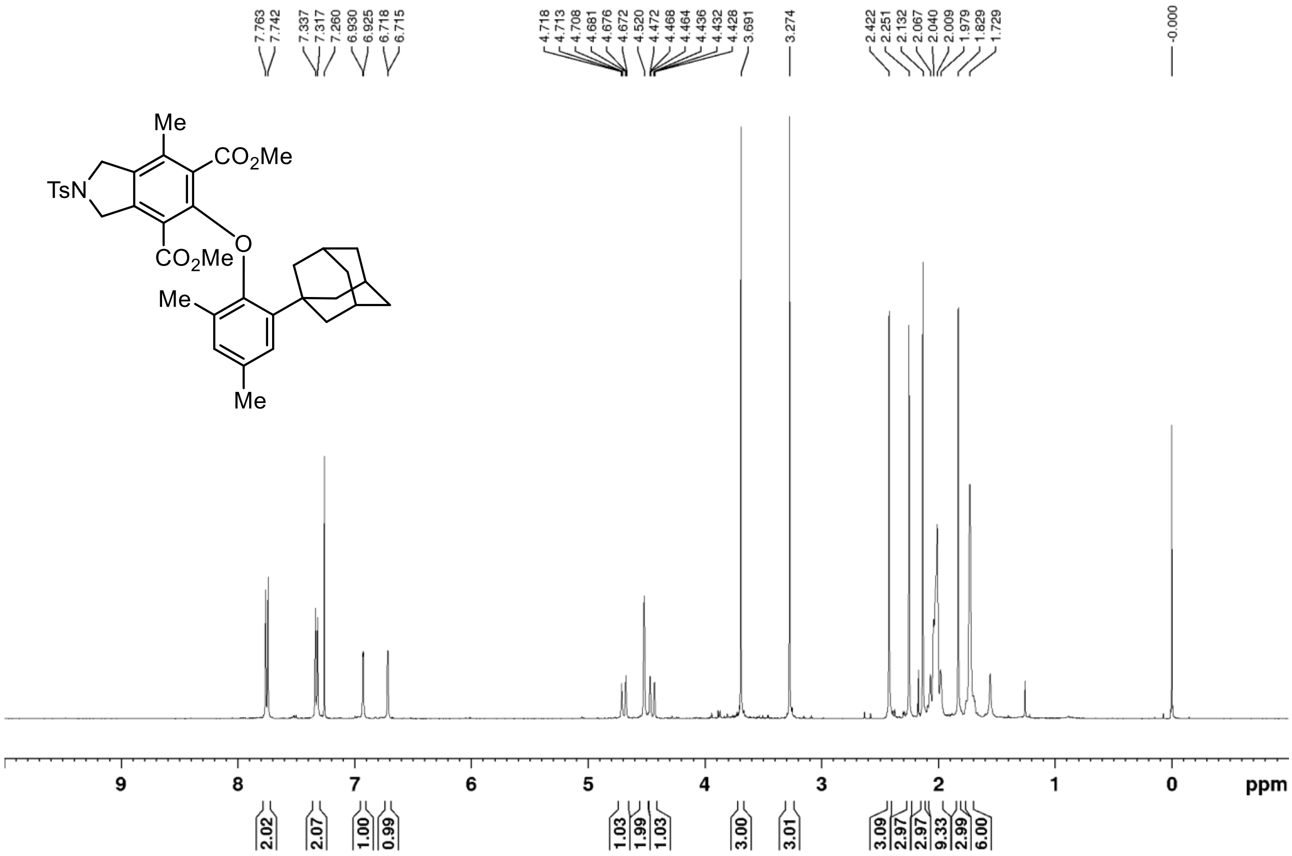

^{13}C NMR ($CDCl_3$, 101 MHz)

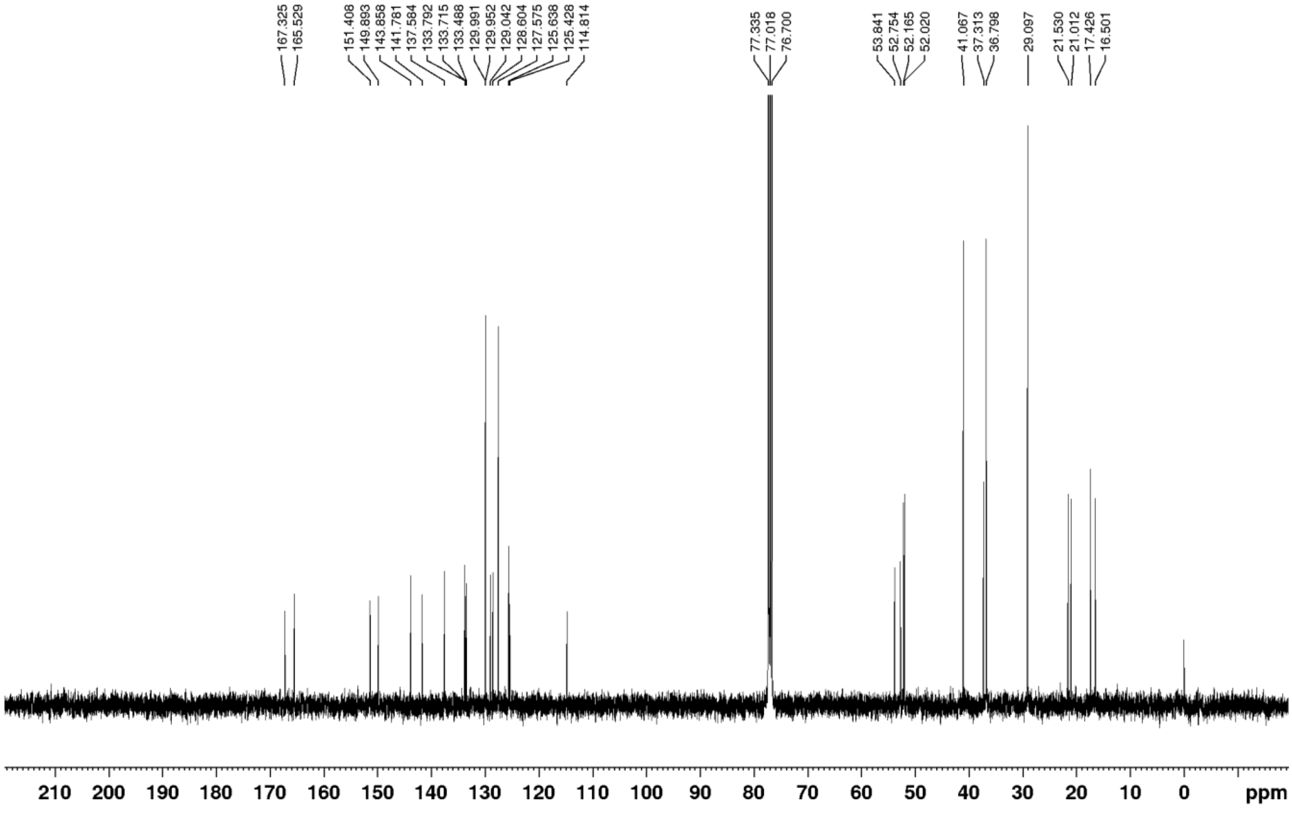

NOESY ($CDCl_3$, 400 MHz)

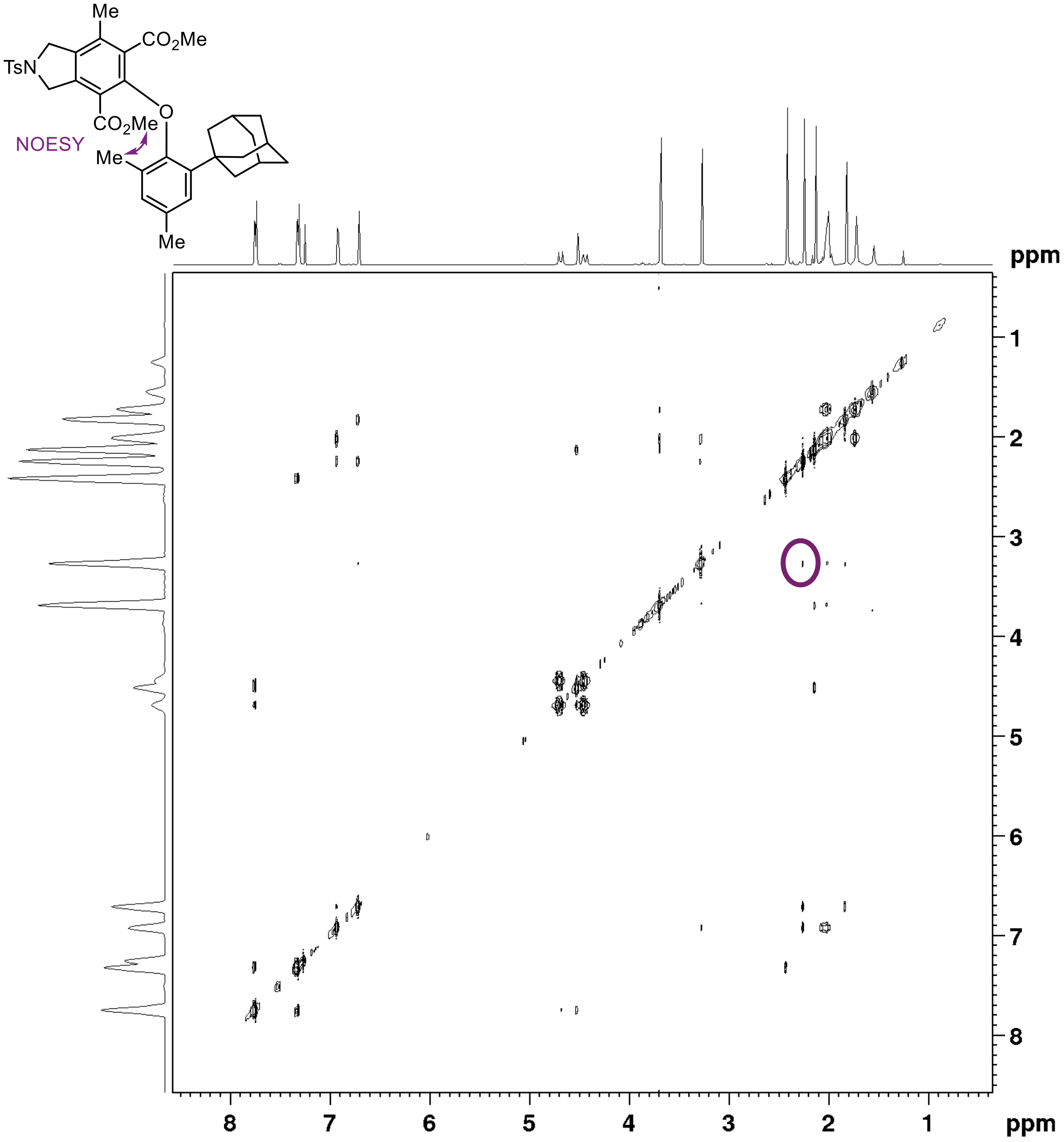

(+)-Methyl 4-(3,5-bis(trifluoromethyl)phenyl)-6-(2-(*tert*-butyl)-6-methylphenoxy)-7-methyl-2-tosylisoindoline-5-carboxylate [(+)-3ga]

^{1}H NMR ($CDCl_3$, 400 MHz)

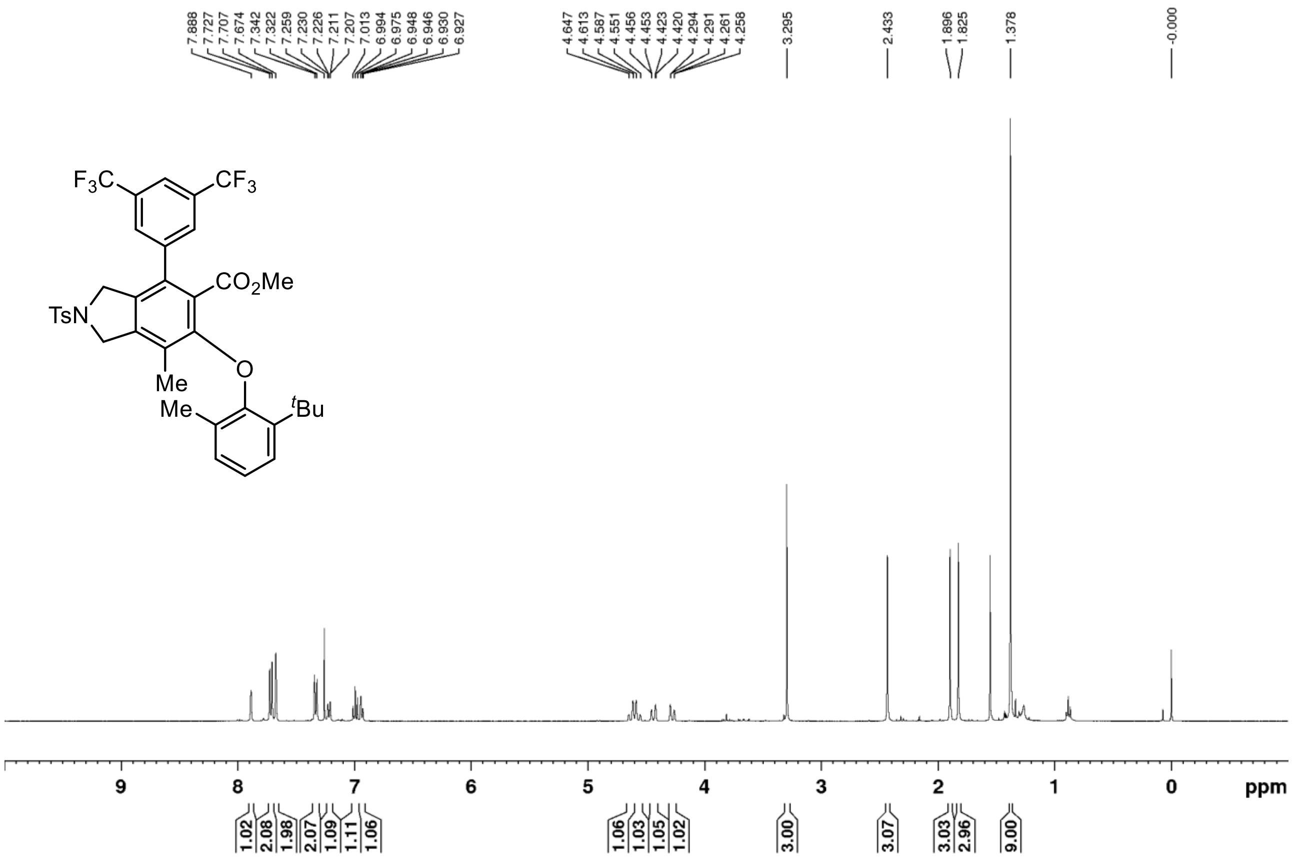

^{13}C NMR ($CDCl_3$, 101 MHz)

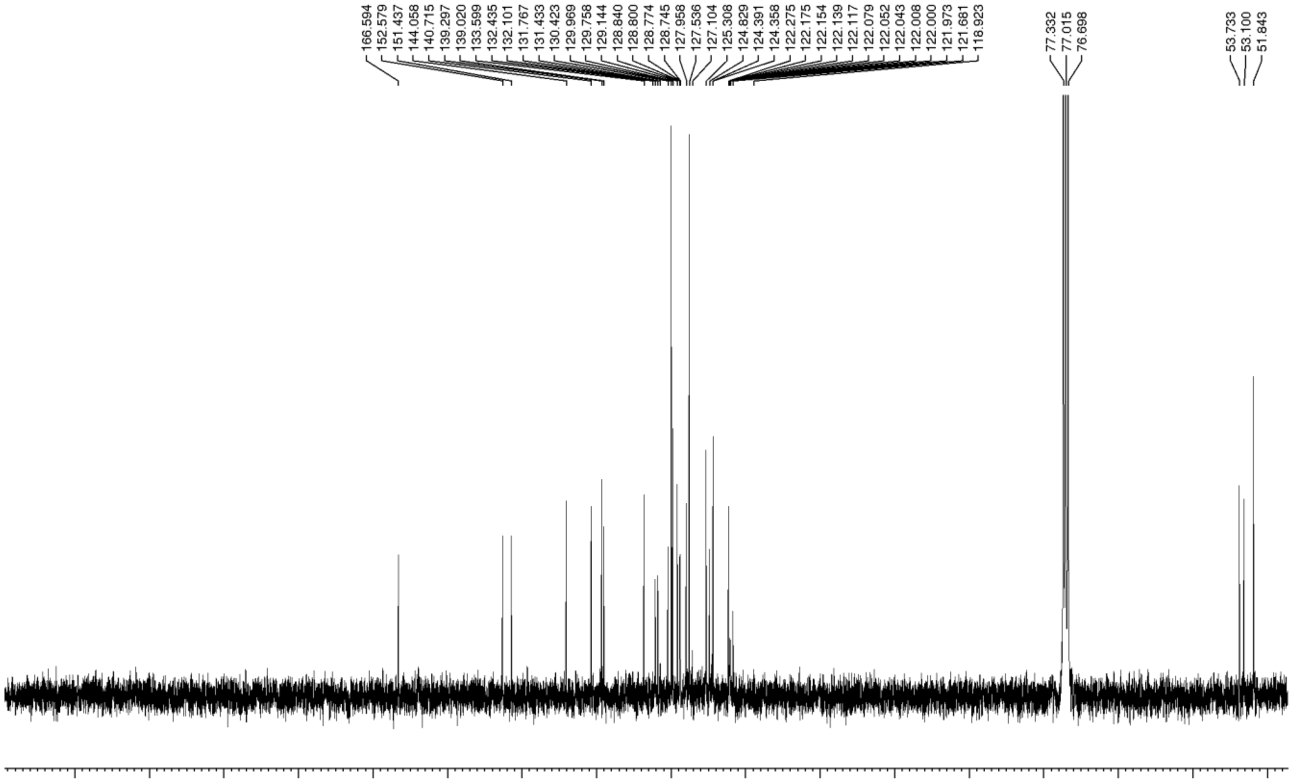

^{19}F NMR ($CDCl_3$, 377 MHz)

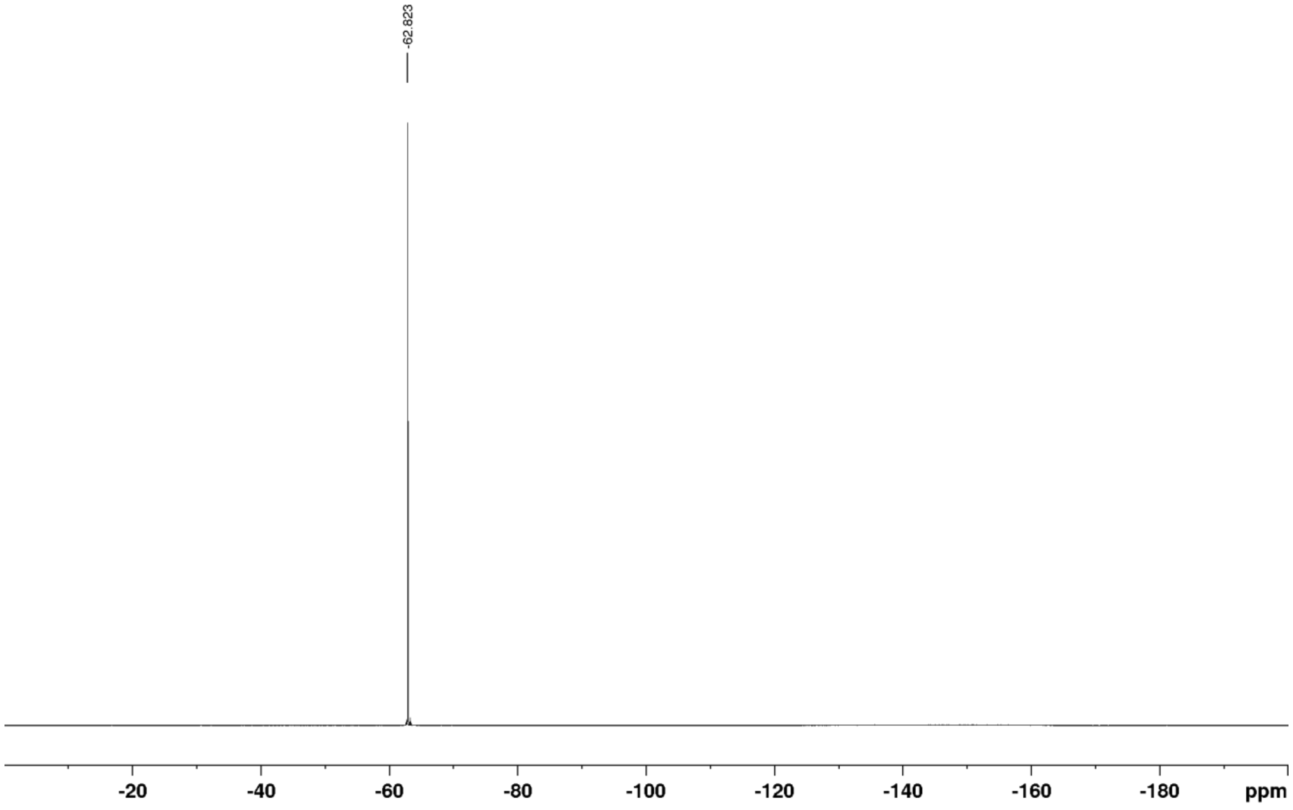

HMBC ($CDCl_3$, 400 MHz)

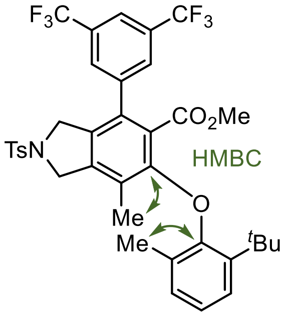

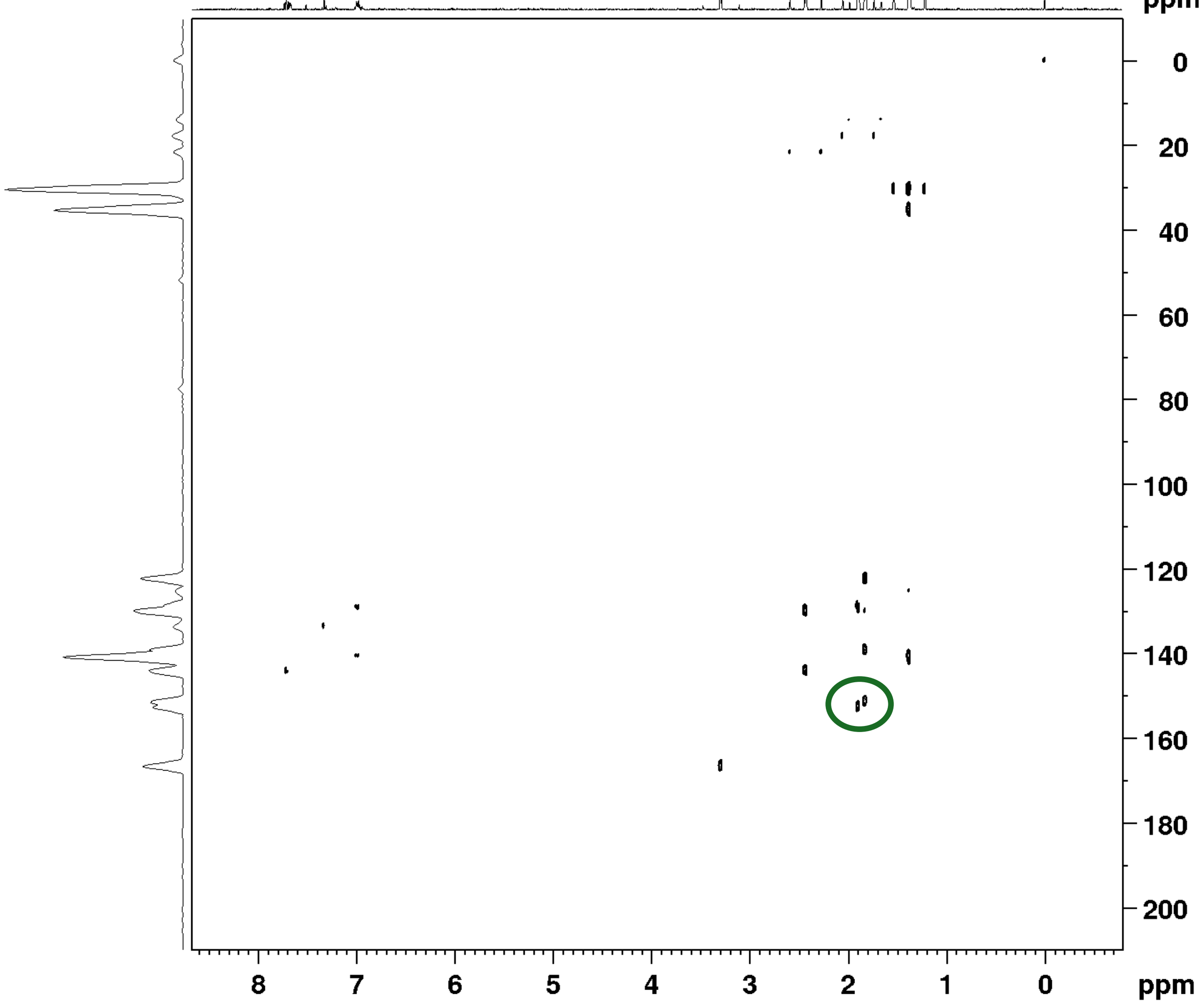

(+)-Methyl 6-(2-(*tert*-butyl)-6-methylphenoxy)-4,7-dimethyl-2-tosylisoindoline-5-carboxylate [(+)-3ha]

^{1}H NMR ($CDCl_3$, 400 MHz)

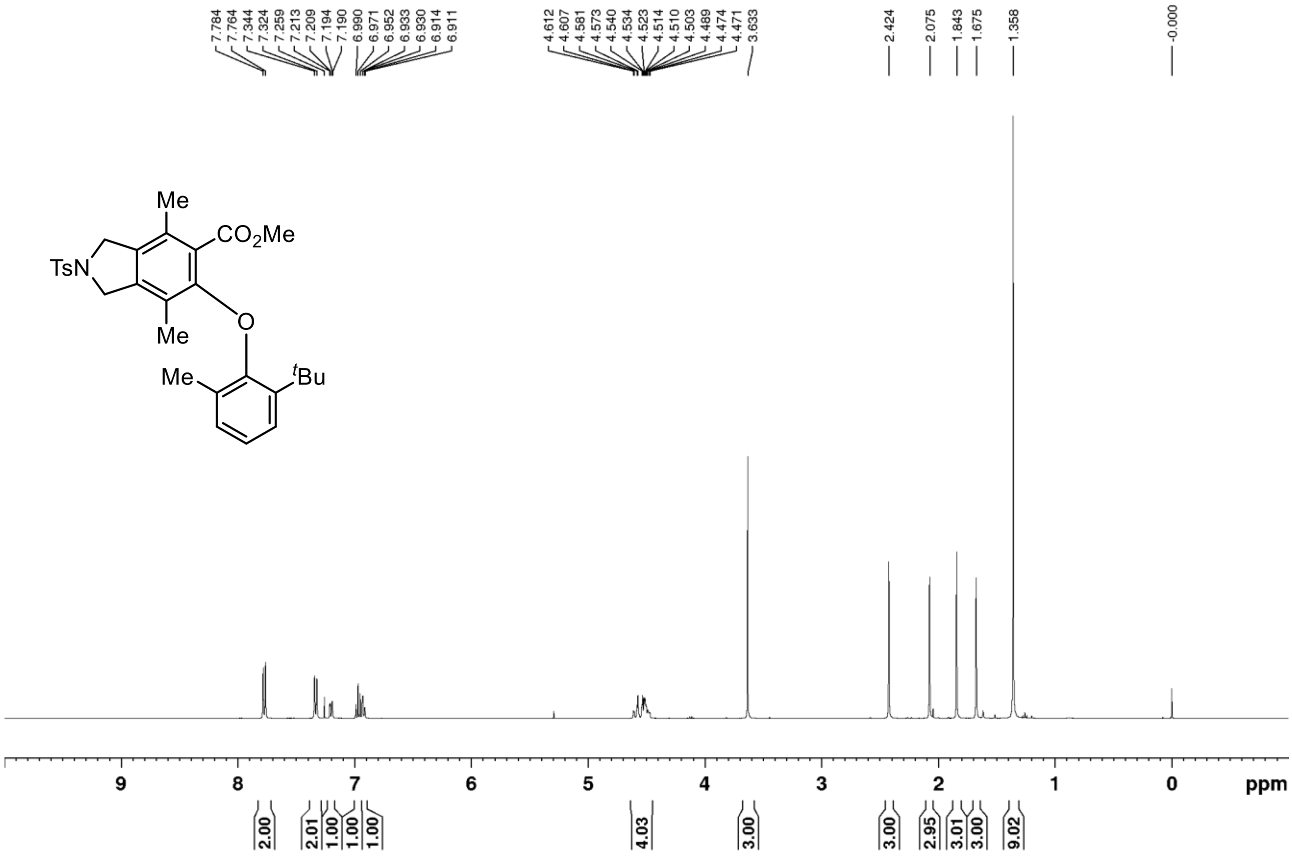

^{13}C NMR ($CDCl_3$, 101 MHz)

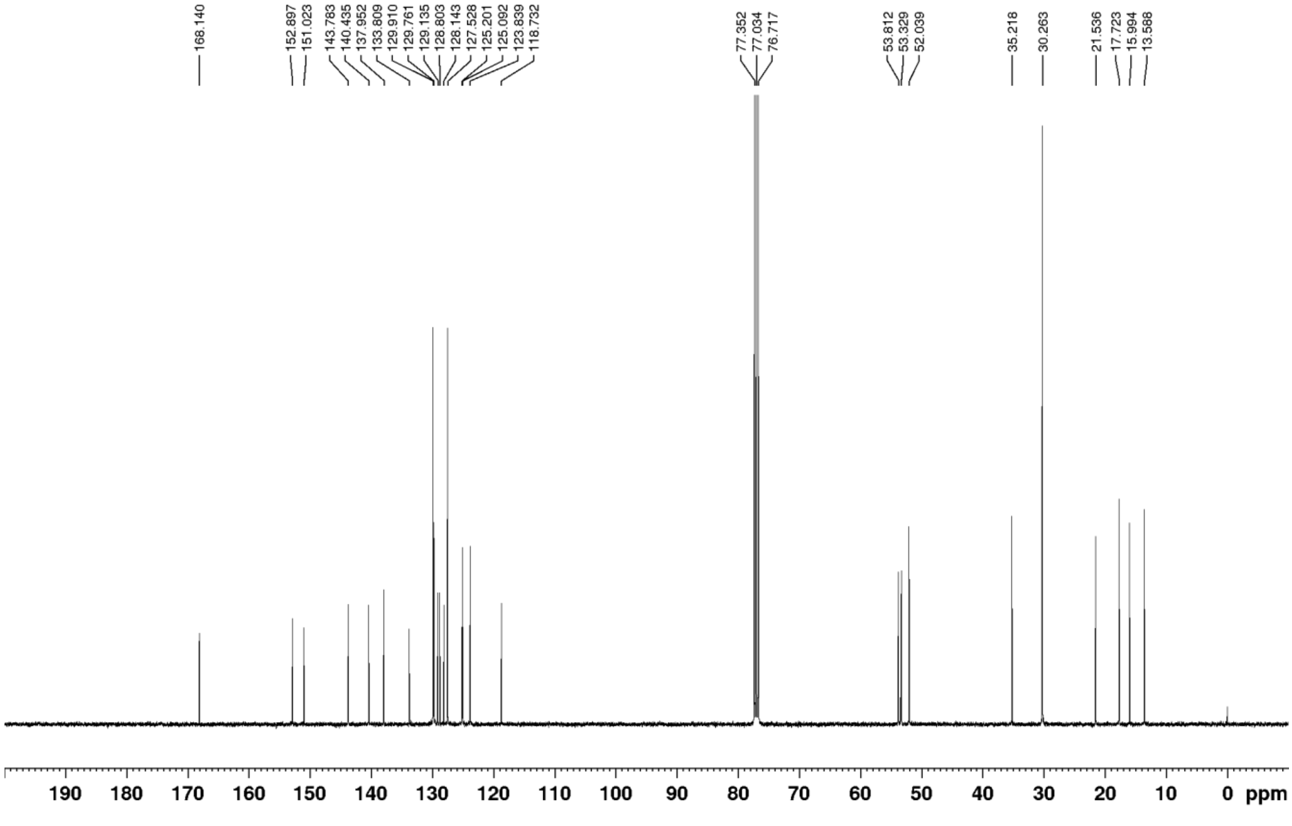

(–)-Methyl 6-(2-(*tert*-butyl)-6-methylphenoxy)-4,7-diphenyl-2-tosylisoindoline-5-carboxylate [(–)-3ia]

^{1}H NMR ($CDCl_3$, 400 MHz)

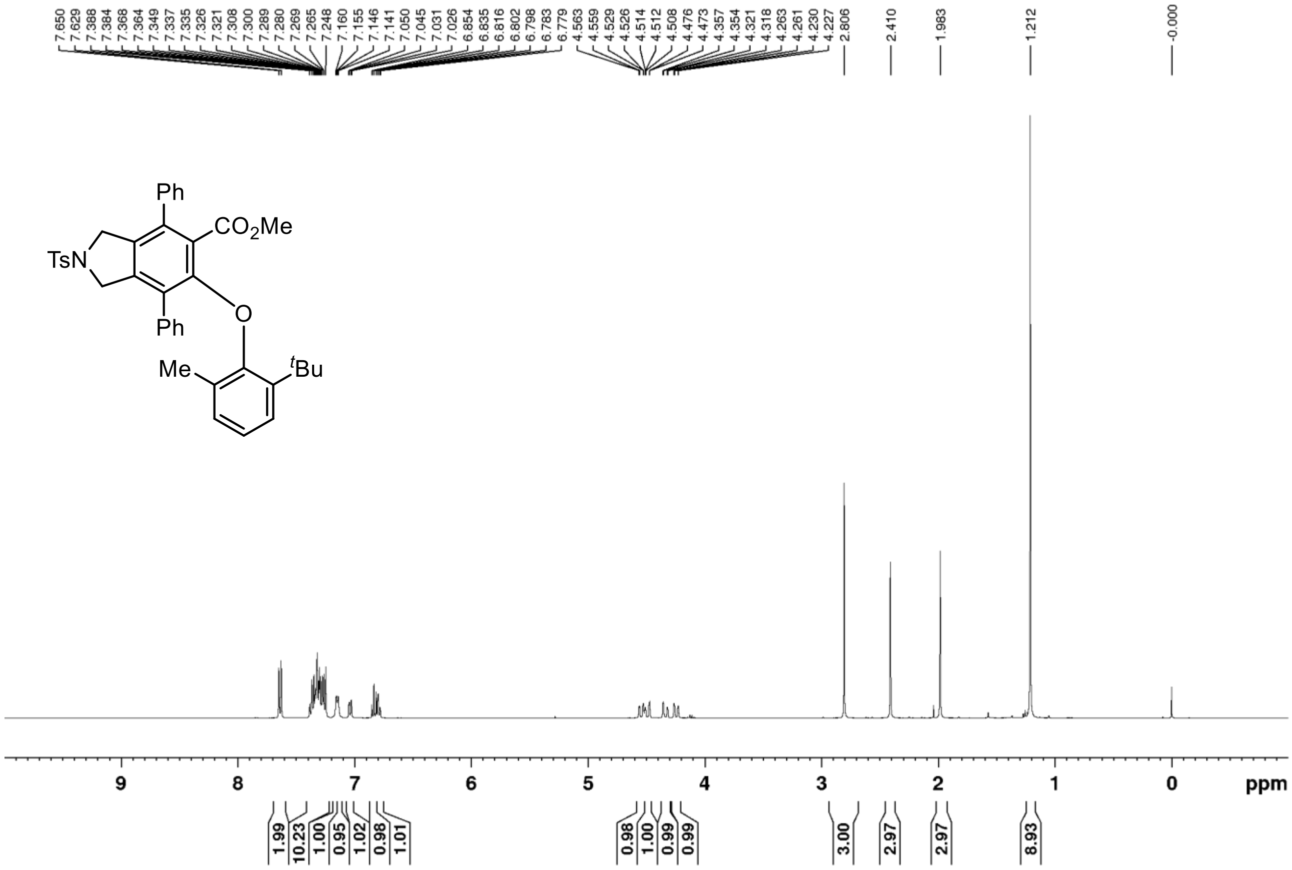

^{13}C NMR ($CDCl_3$, 101 MHz)

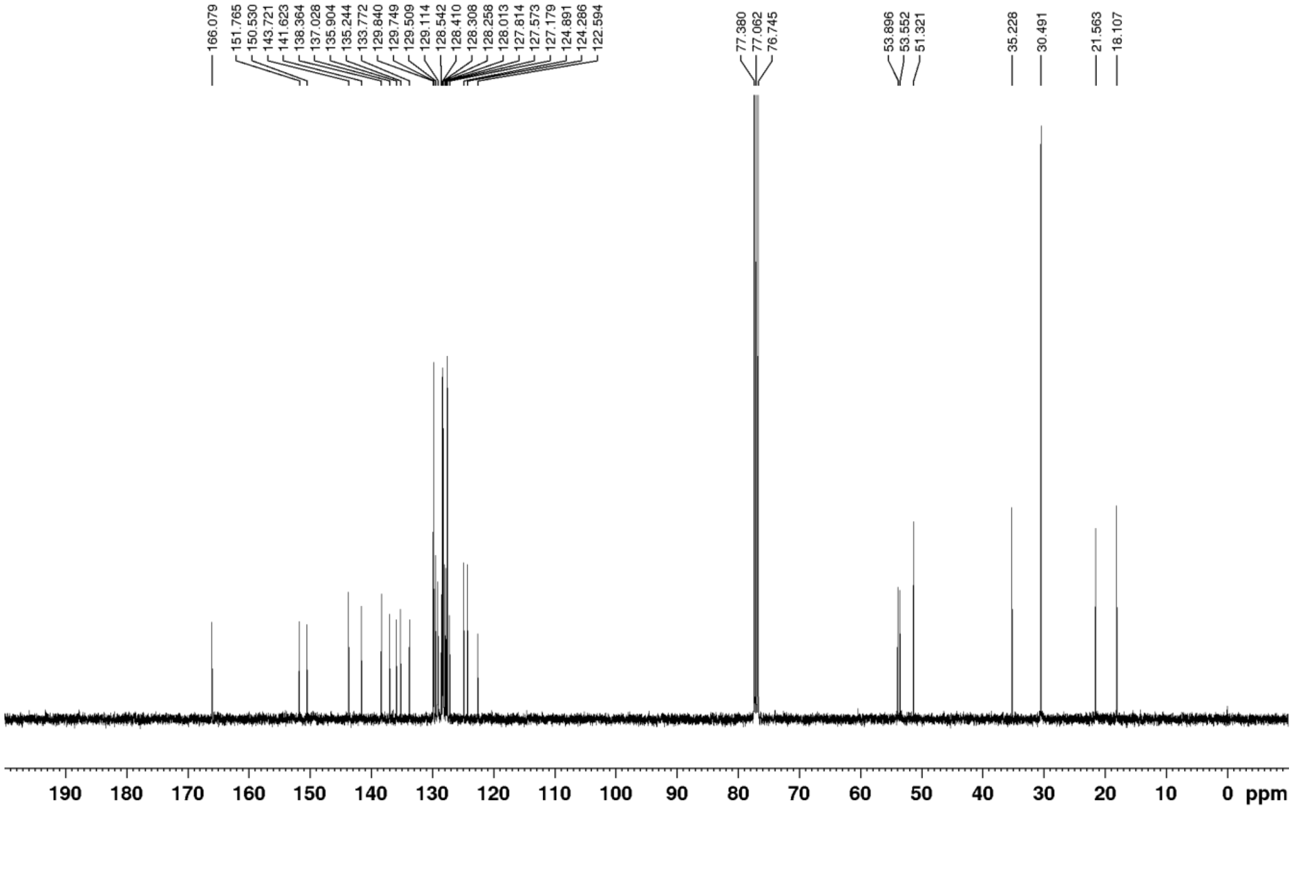

(–)-Methyl 6-(2-(*tert*-butyl)-6-methylphenoxy)-4,7-bis(4-chlorophenyl)-2-tosylisoindoline-5-carboxylate [(–)-3ja]

^{1}H NMR ($CDCl_3$, 400 MHz)

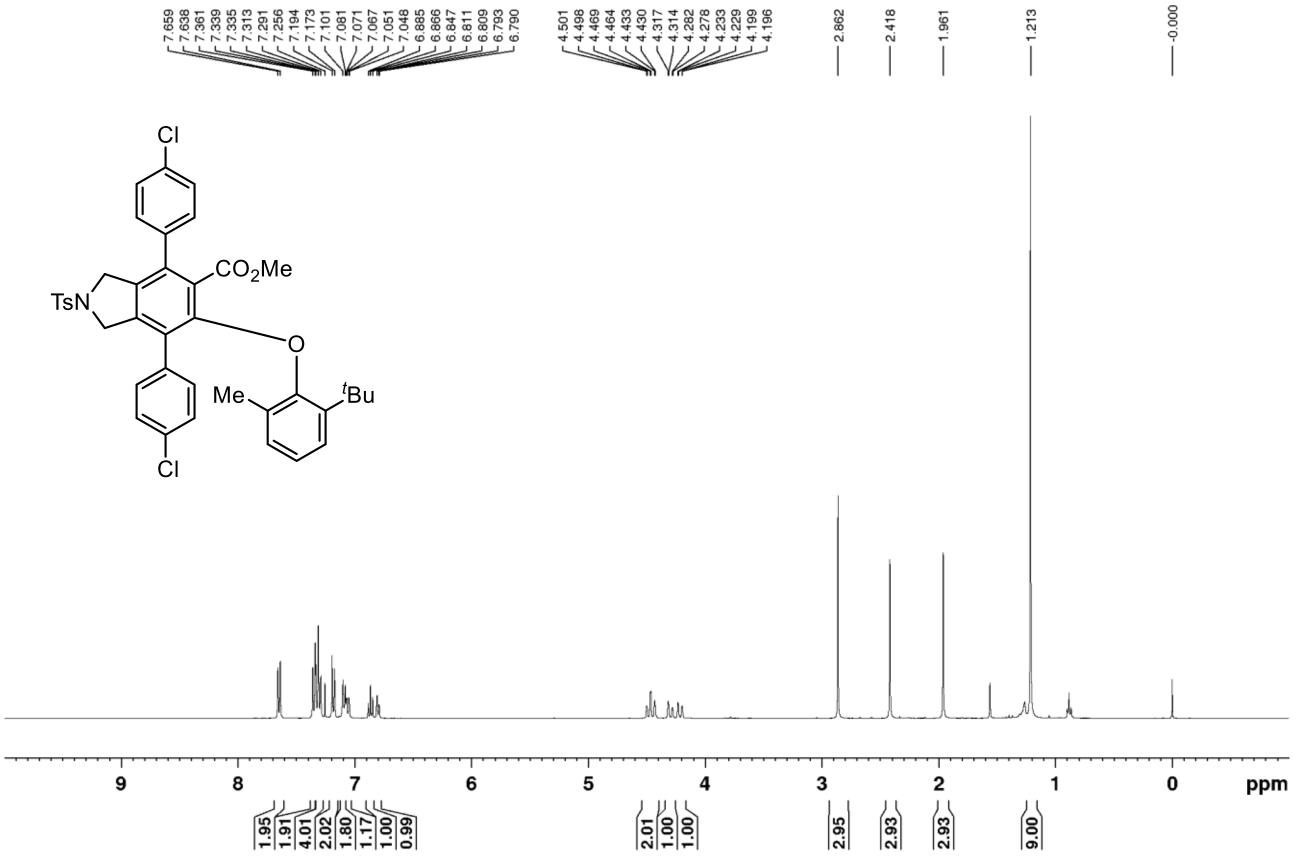

^{13}C NMR ($CDCl_3$, 101 MHz)

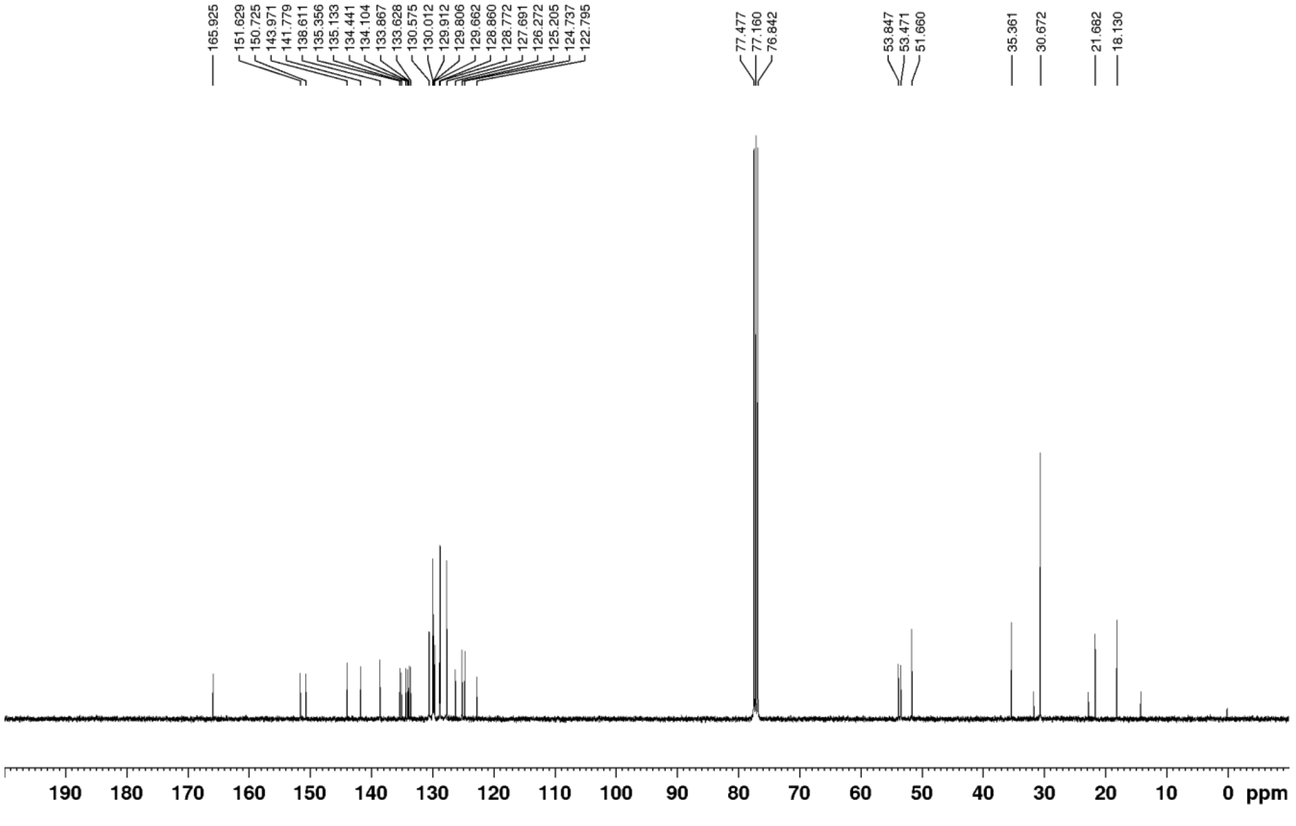

(–)-Methyl 6-(2-(*tert*-butyl)-6-methylphenoxy)-4,7-di(thiophen-3-yl)-2-tosylisoindoline-5-carboxylate [(–)-3ka]

^{1}H NMR ($CDCl_3$, 400 MHz)

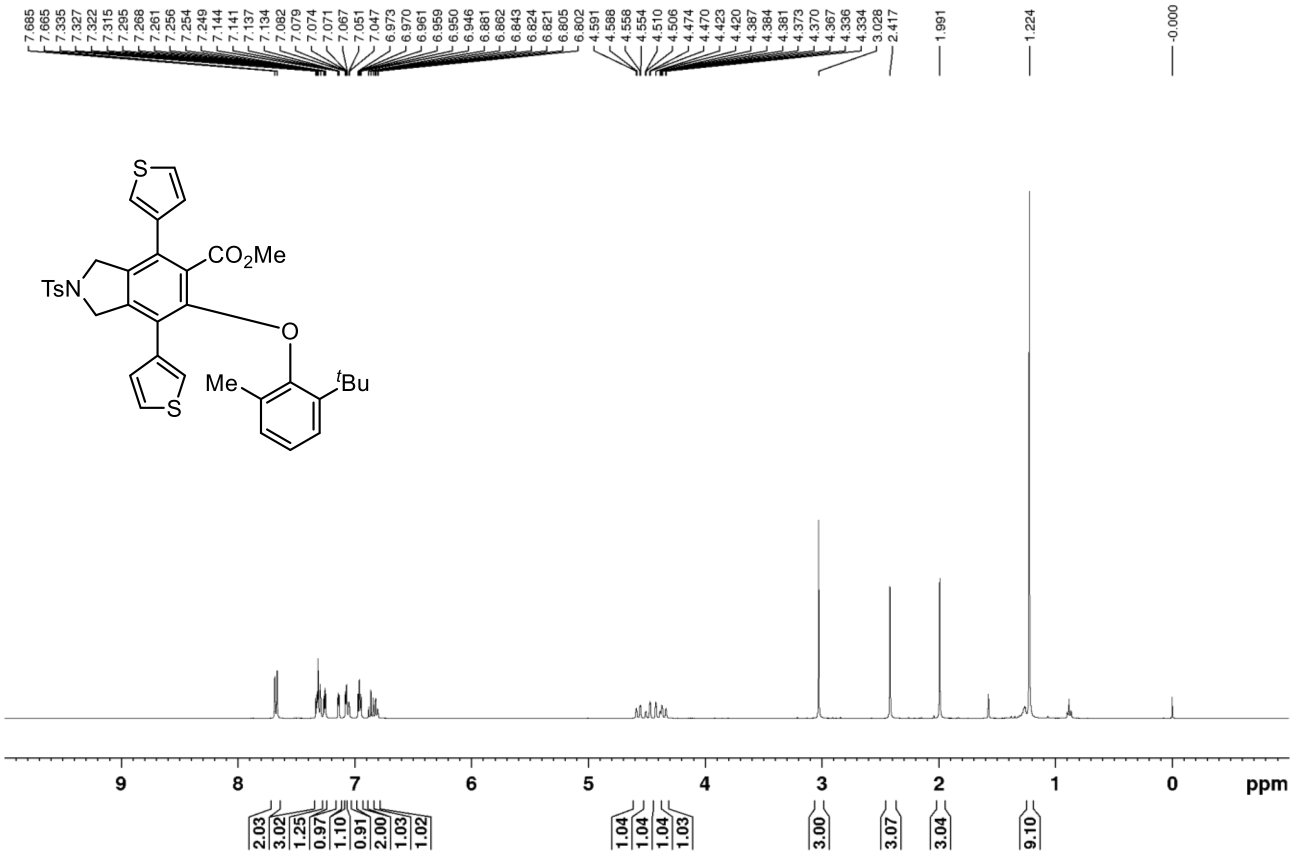

^{13}C NMR ($CDCl_3$, 101 MHz)

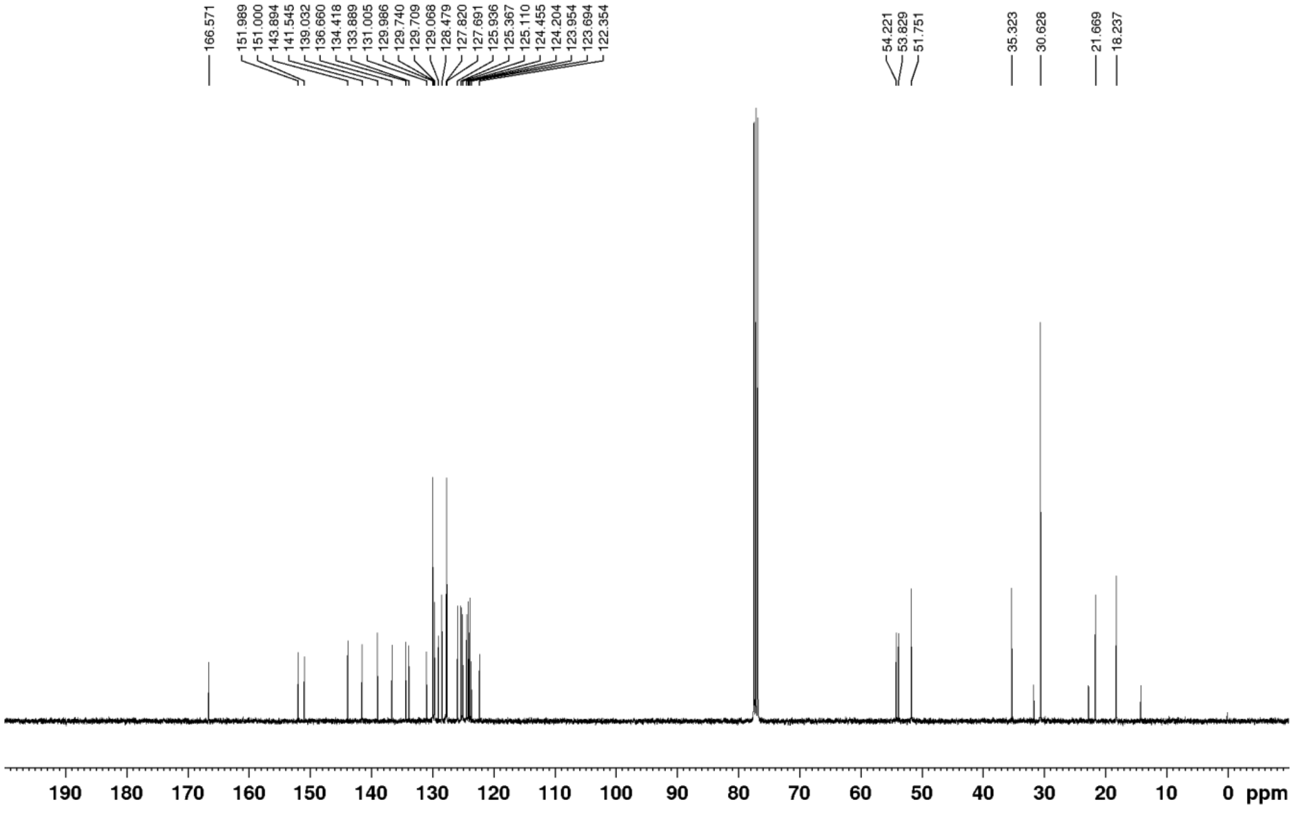

(+)-Trimethyl 6-(2-(*tert*-butyl)-6-methylphenoxy)-2-tosylisoindoline-4,5,7-tricarboxylate [(+)-3la]

^{1}H NMR ($CDCl_3$, 400 MHz)

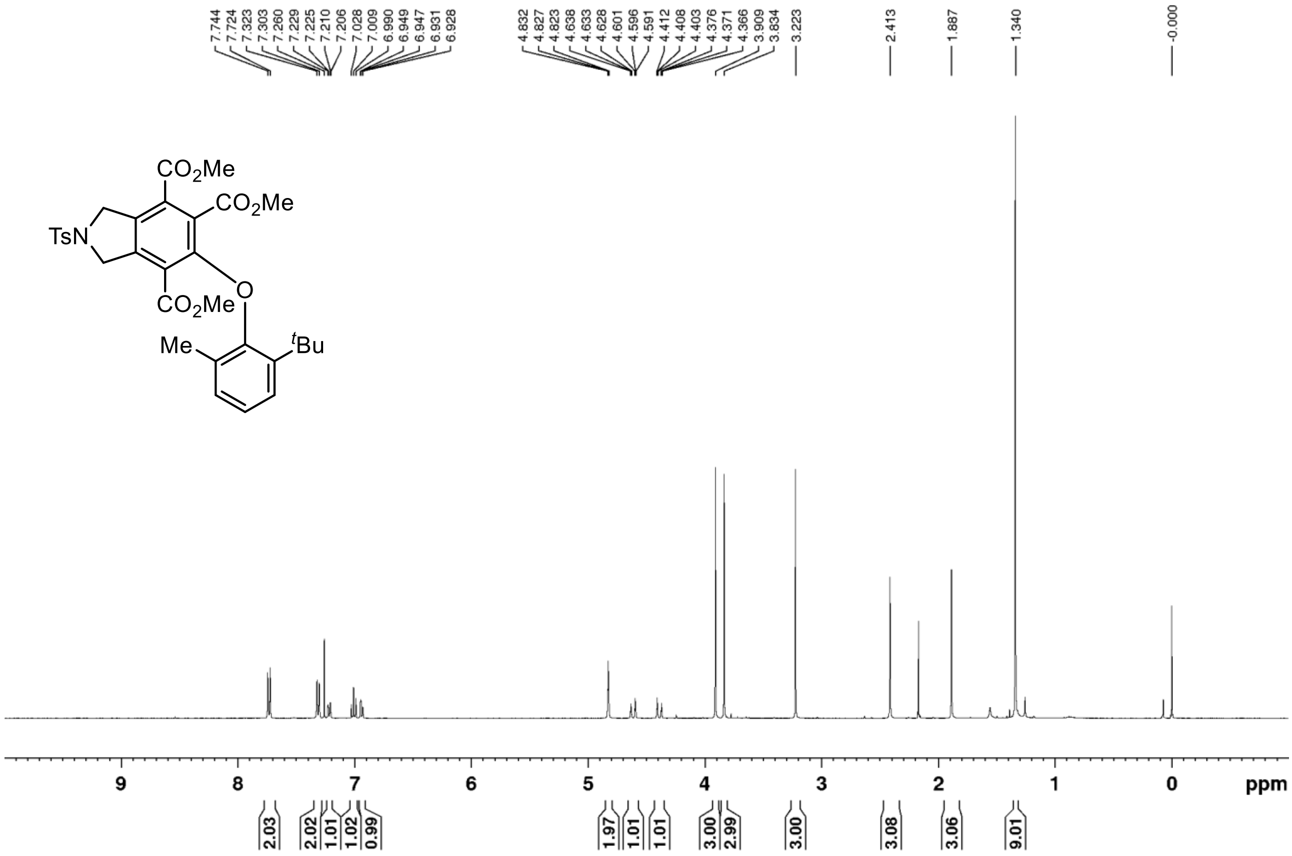

^{13}C NMR ($CDCl_3$, 101 MHz)

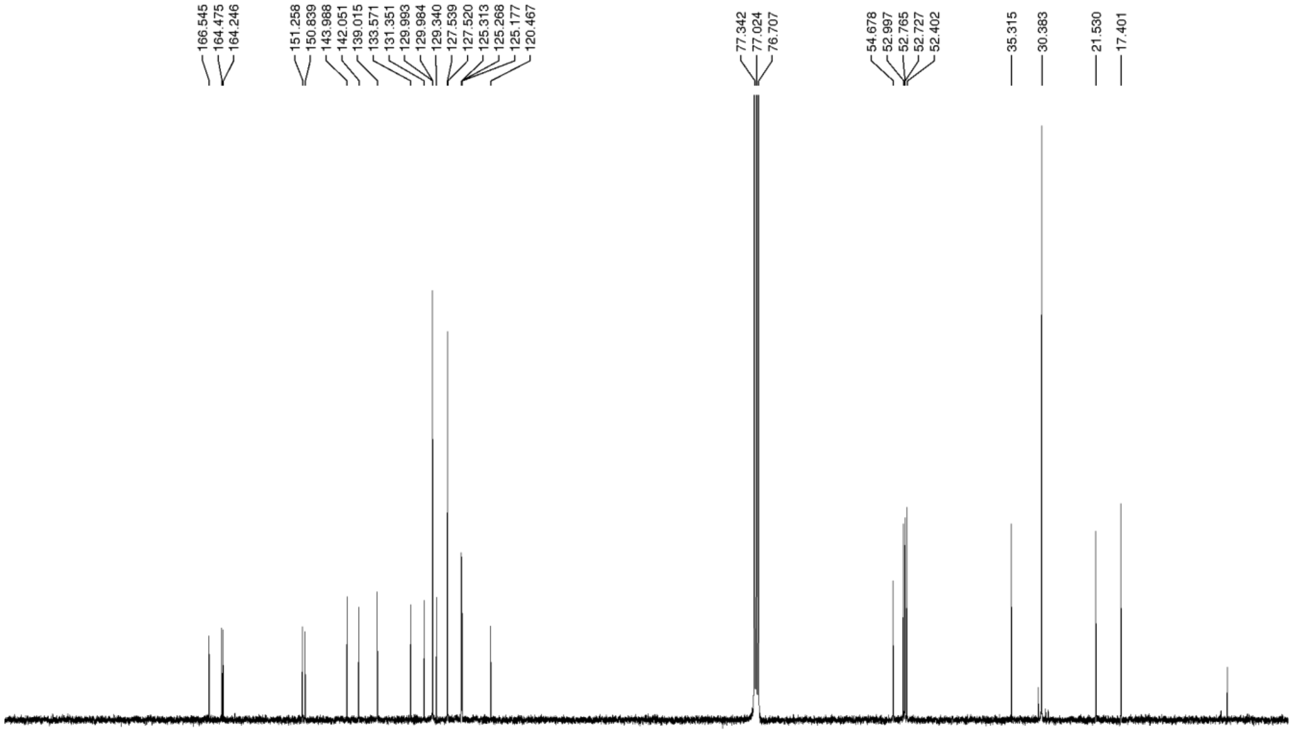

(+)-Methyl 6-(2-(*tert*-butyl)-6-methoxy-4-methylphenoxy)-4,7-dimethyl-2-tosylisoindoline-5-carboxylate [(+)-3hb]

^{1}H NMR (CDCl$_3$, 400 MHz)

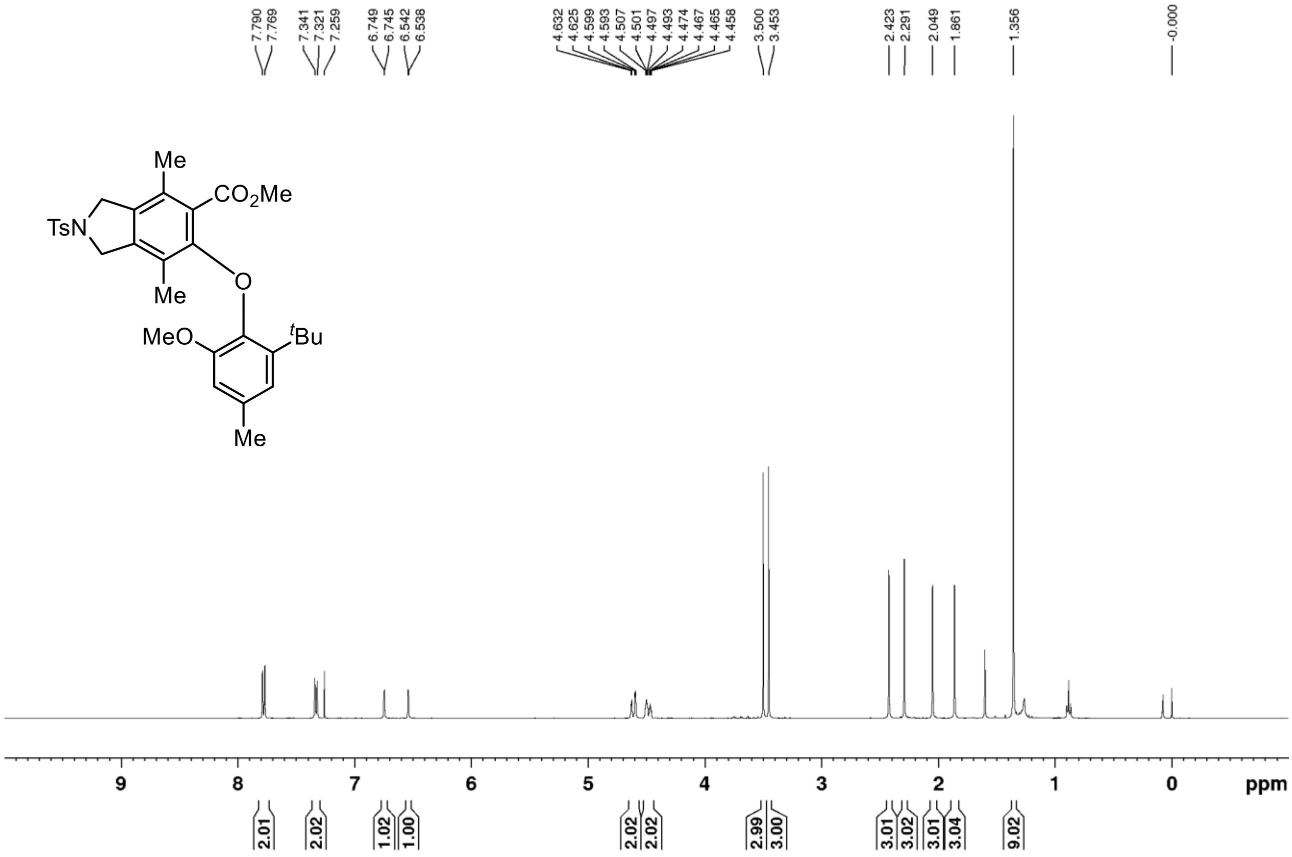

^{13}C NMR (CDCl$_3$, 101 MHz)

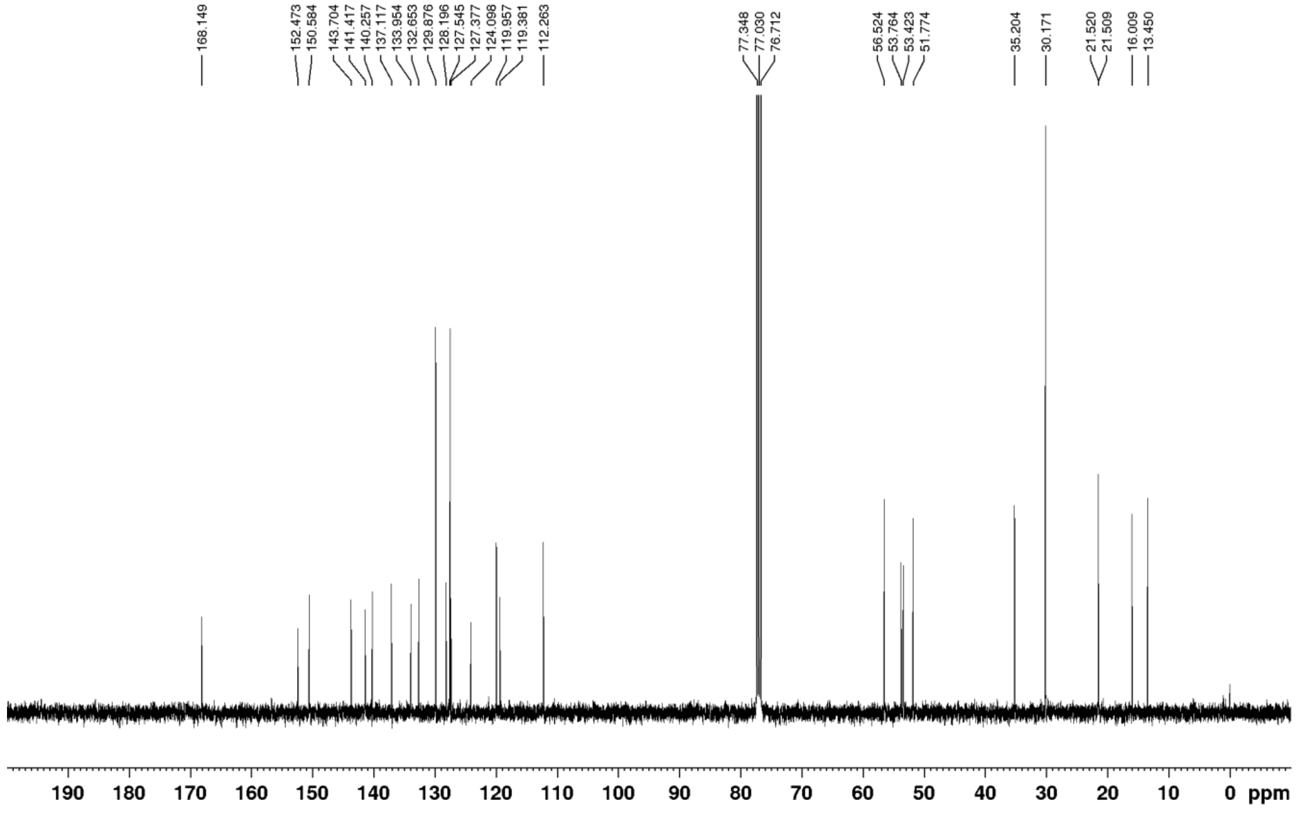

(+)-Dimethyl 6-((3-(*tert*-butyl)-4'-methoxy-5-methyl-[1,1'-biphenyl]-2-yl)oxy)-7-methyl-2-tosylisoindoline-4,5-dicarboxylate [(+)-3aj]

^{1}H NMR ($CDCl_3$, 400 MHz)

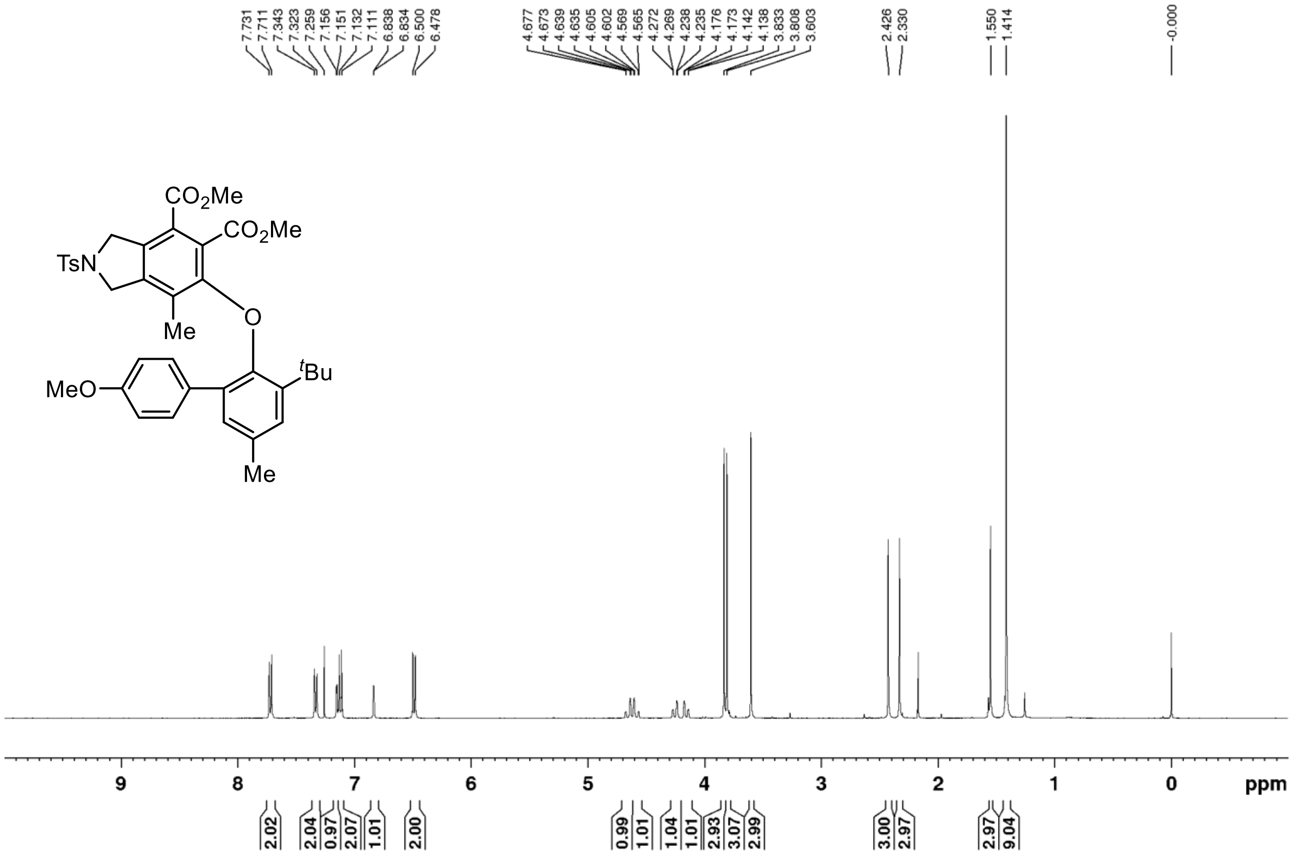

^{13}C NMR ($CDCl_3$, 101 MHz)

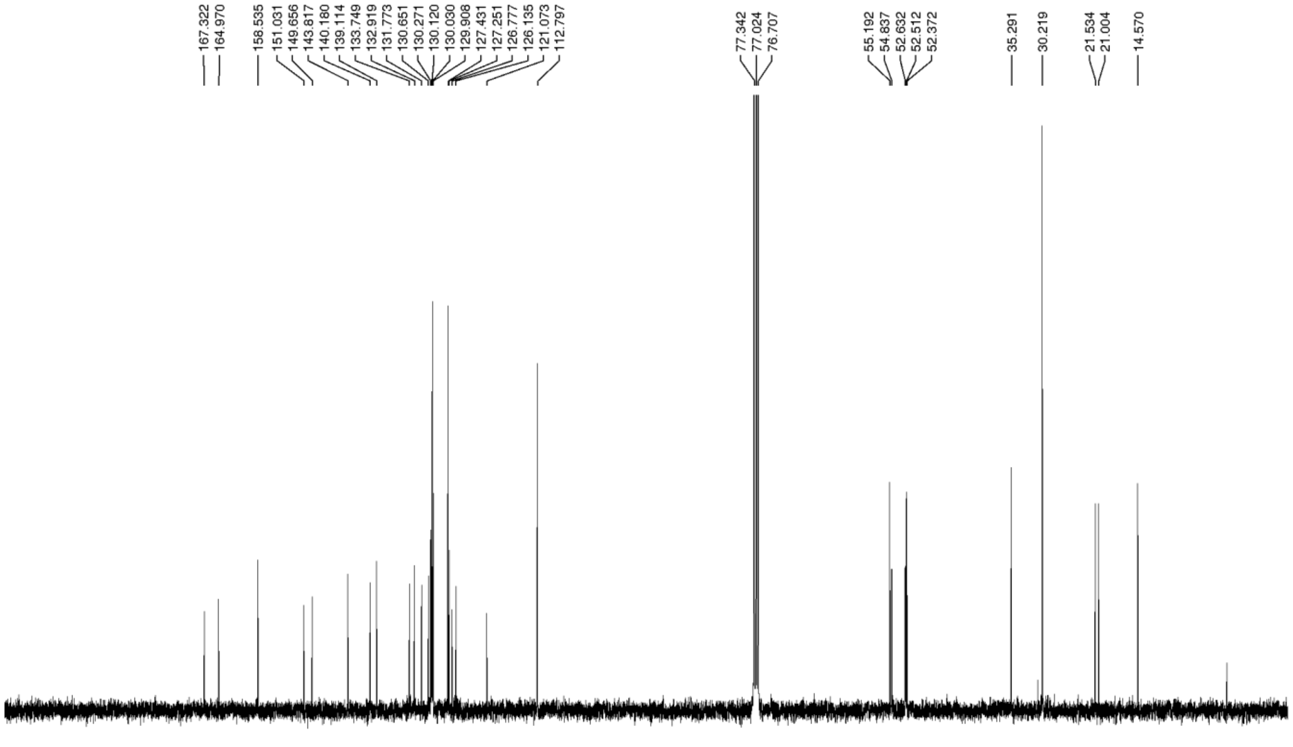

HMBC ($CDCl_3$, 400 MHz)

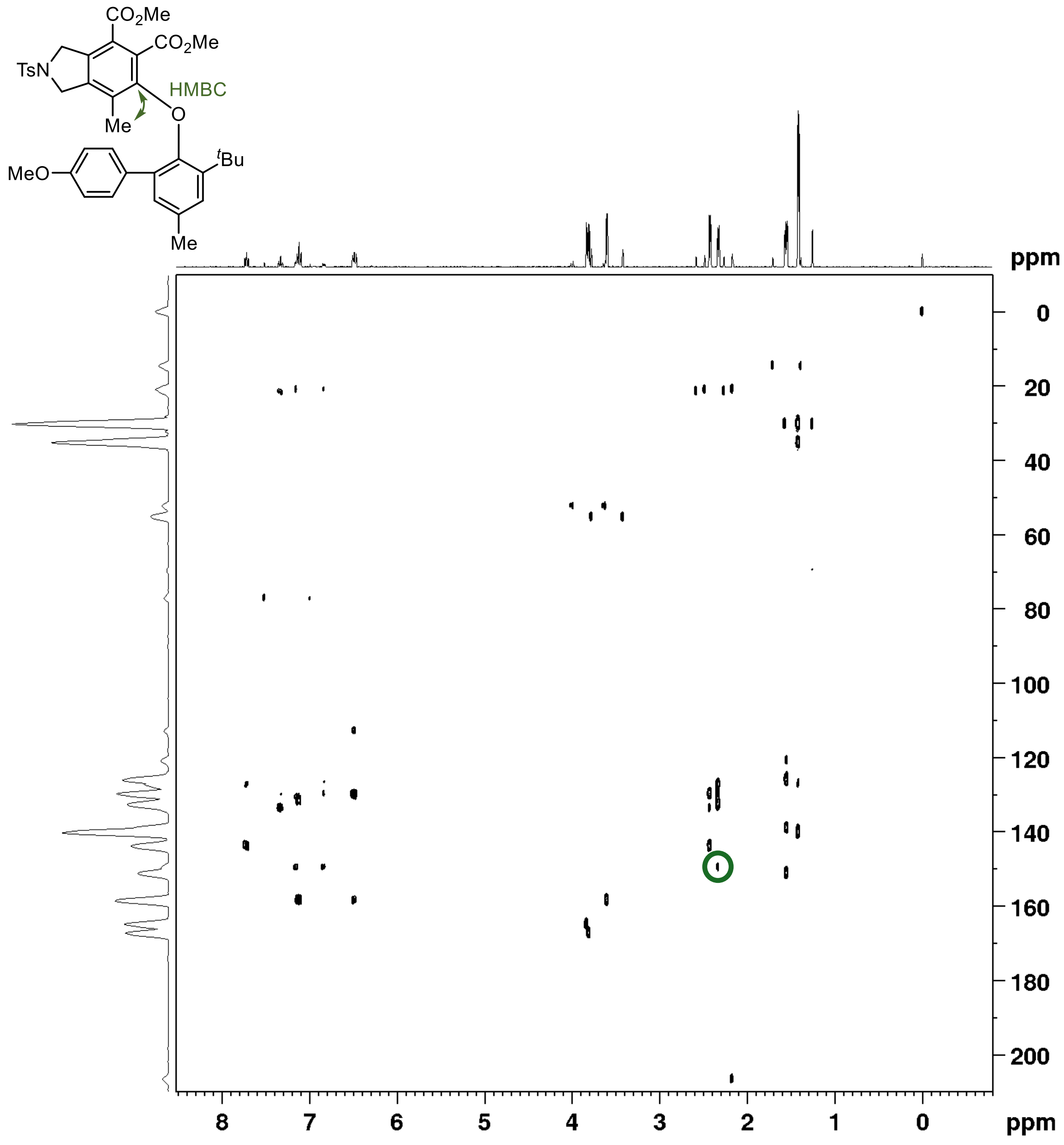

(+)-Dimethyl 6-((3-(*tert*-butyl)-5-methyl-4'-(trifluoromethyl)-[1,1'-biphenyl]-2-yl)oxy)-7-methyl-2-tosylisoindoline-4,5-dicarboxylate [(+)-3ak]

^{1}H NMR ($CDCl_3$, 400 MHz)

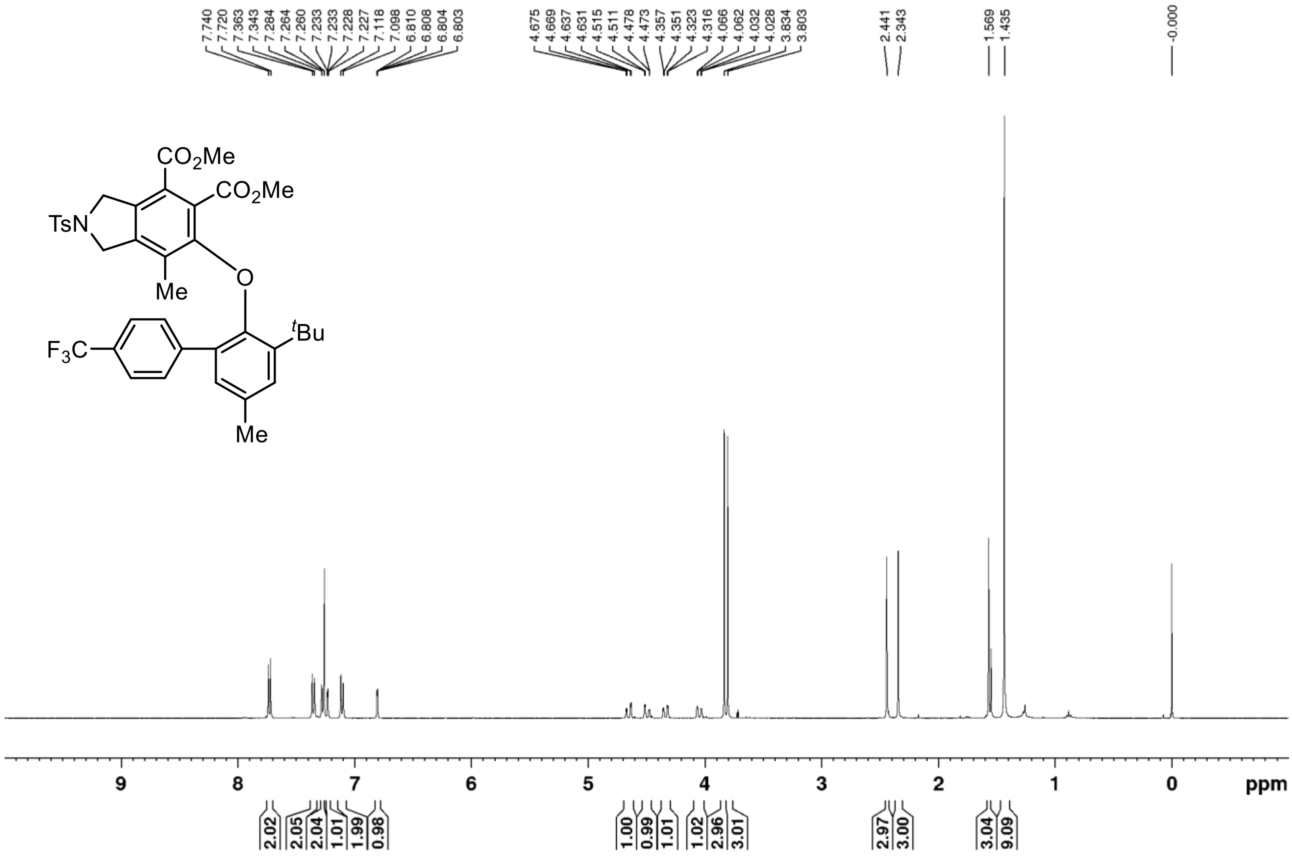

^{13}C NMR ($CDCl_3$, 101 MHz)

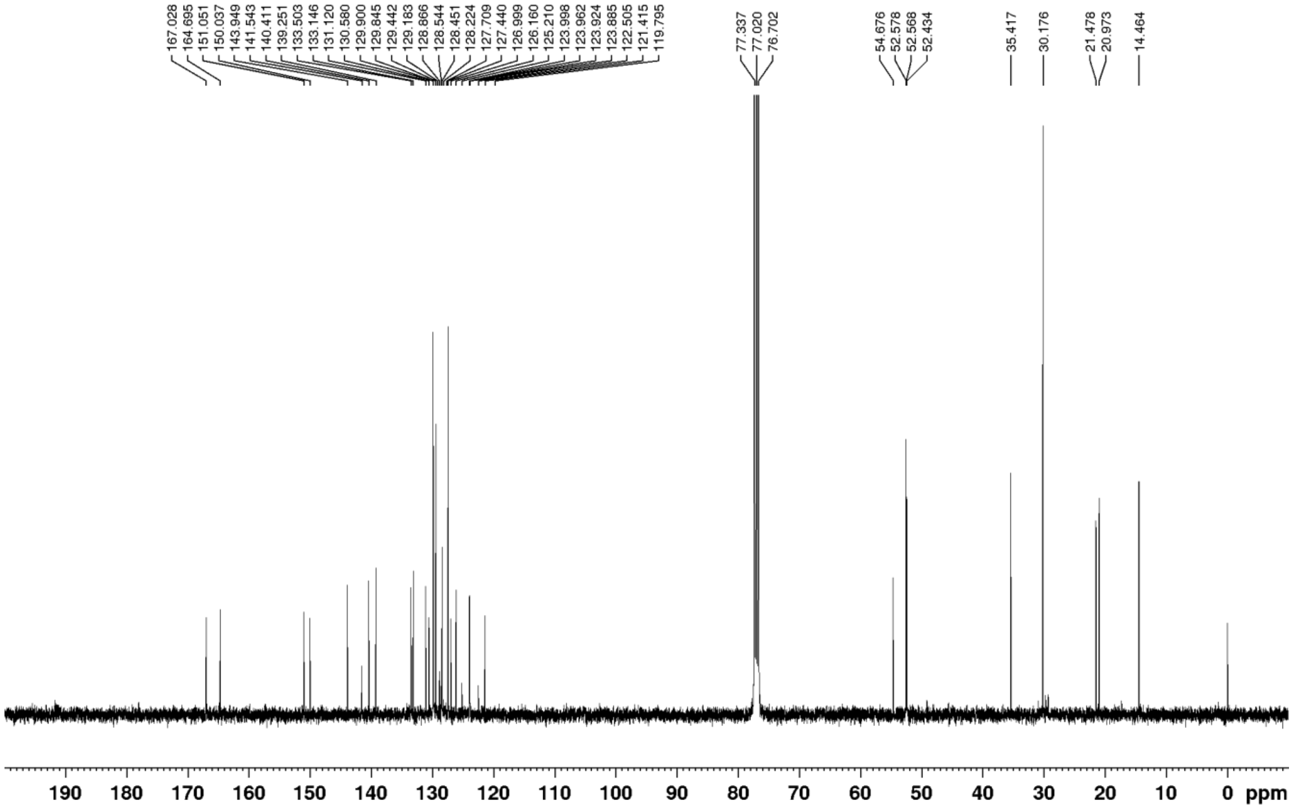

^{19}F NMR ($CDCl_3$, 377 MHz)

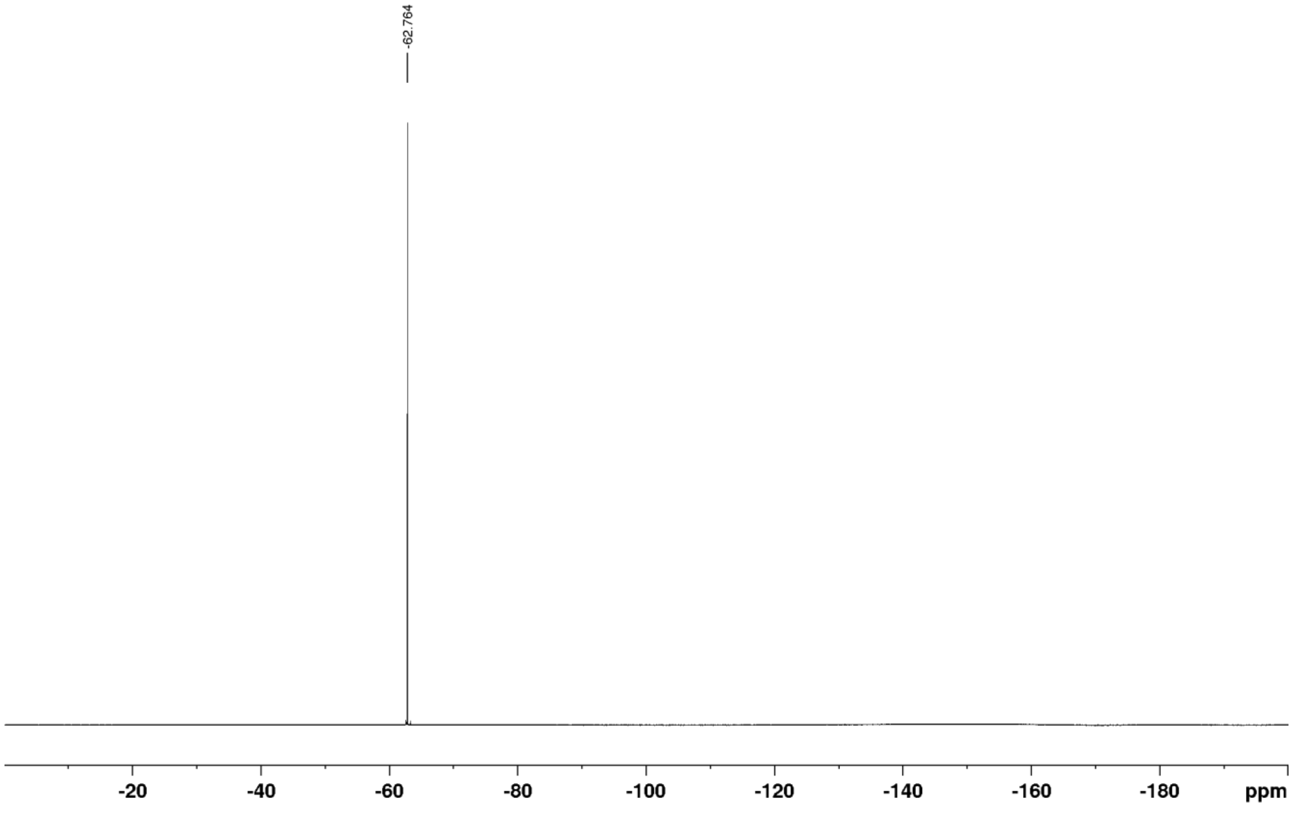

HMBC ($CDCl_3$, 400 MHz)

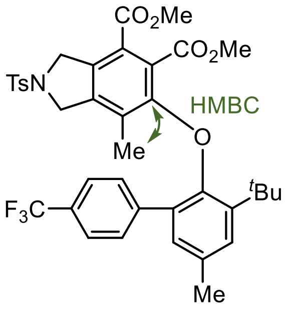

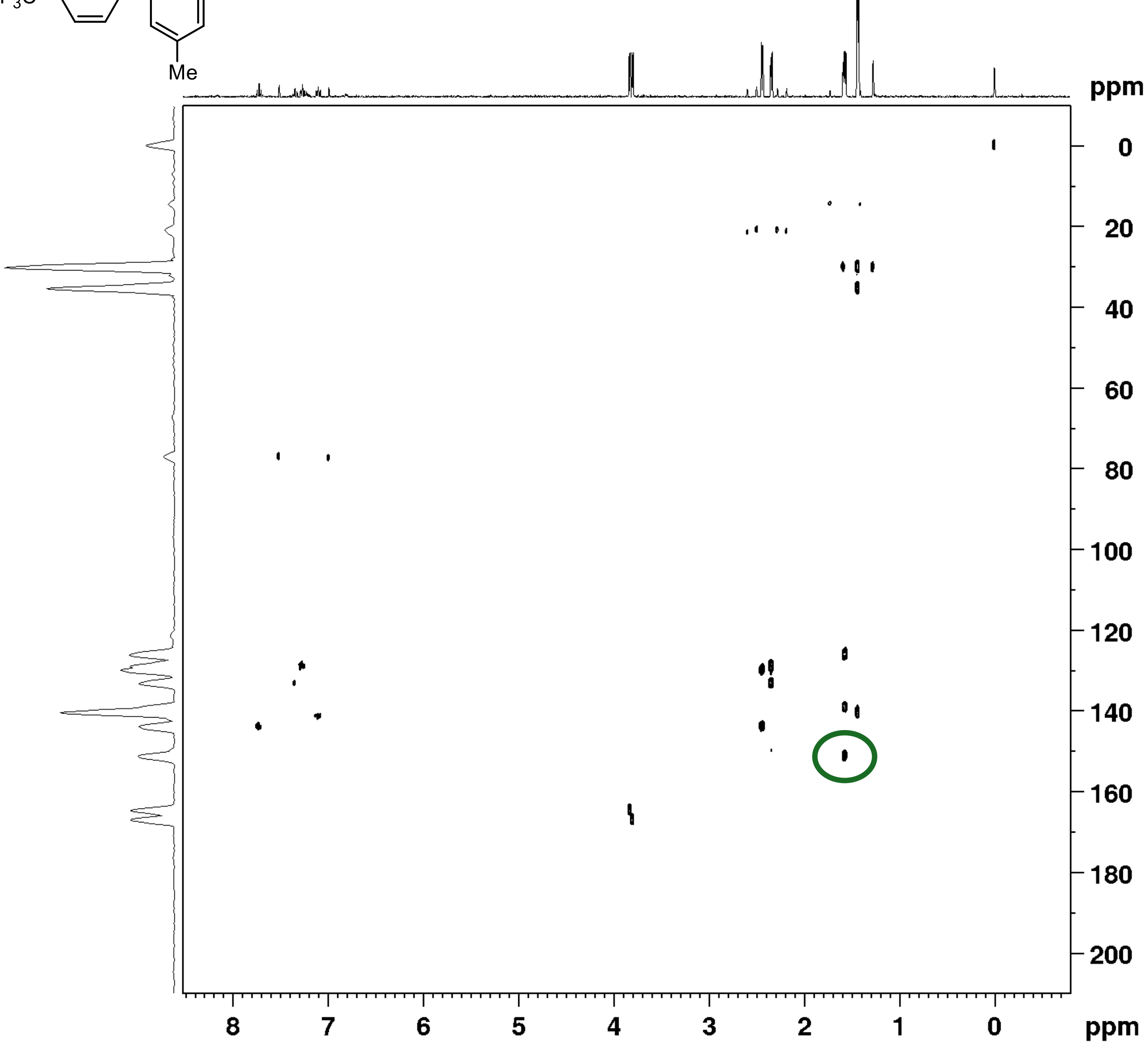

(+)-Dimethyl 6-((3-(*tert*-butyl)-2',5-dimethyl-[1,1'-biphenyl]-2-yl)oxy)-7-methyl-2-tosylisoindoline-4,5-dicarboxylate [(+)-3al]

^{1}H NMR ($CDCl_3$, 400 MHz)

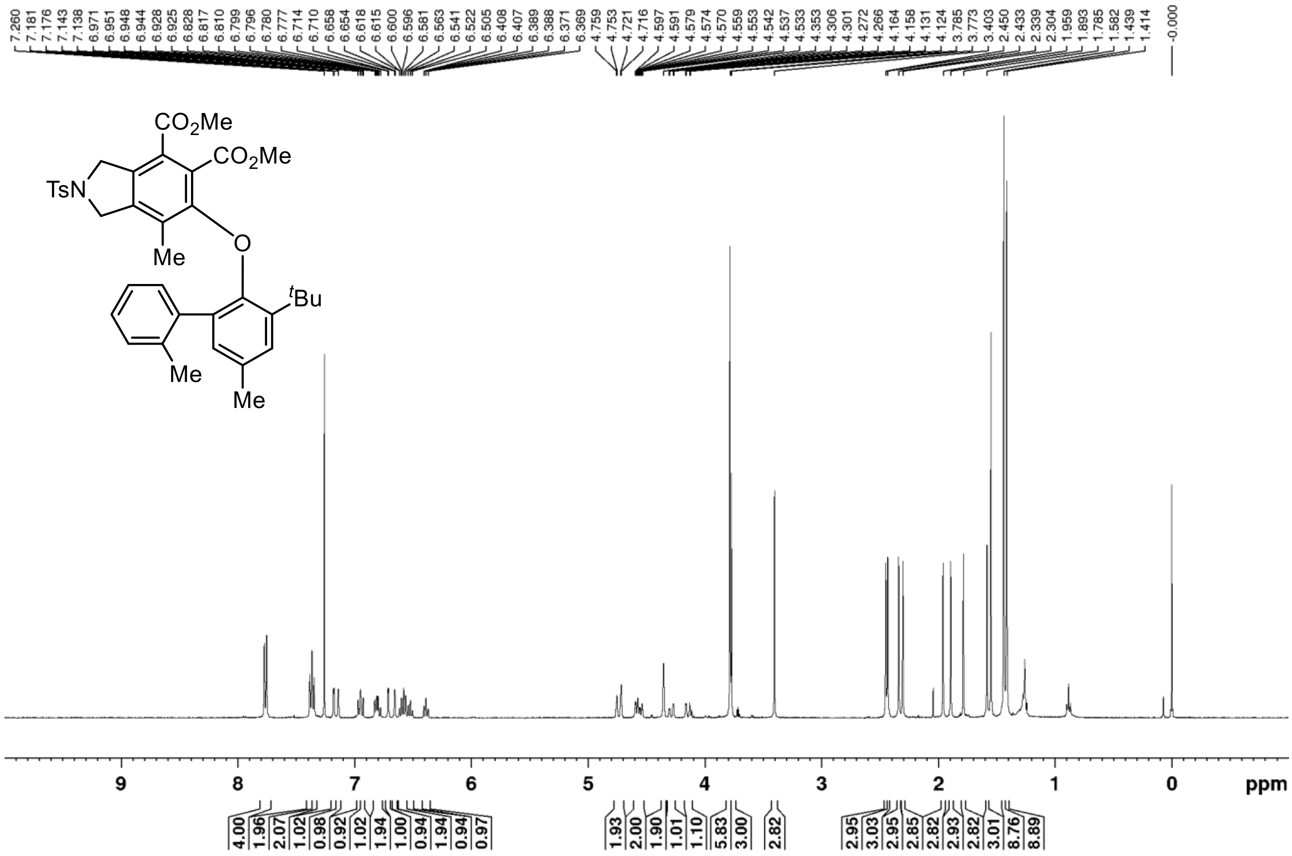

^{13}C NMR ($CDCl_3$, 101 MHz)

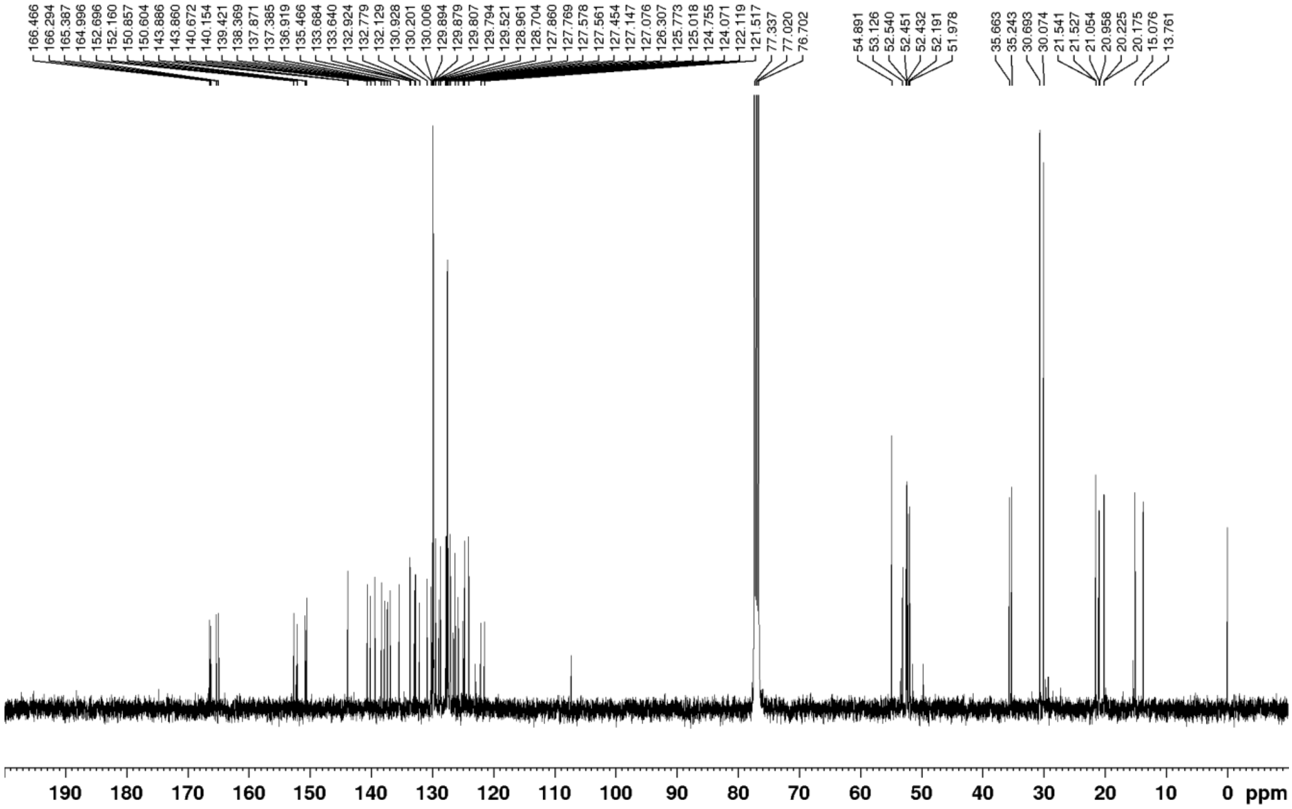

HMBC ($CDCl_3$, 400 MHz)

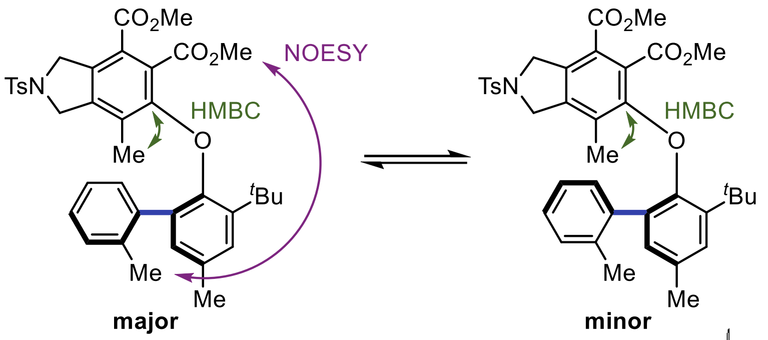

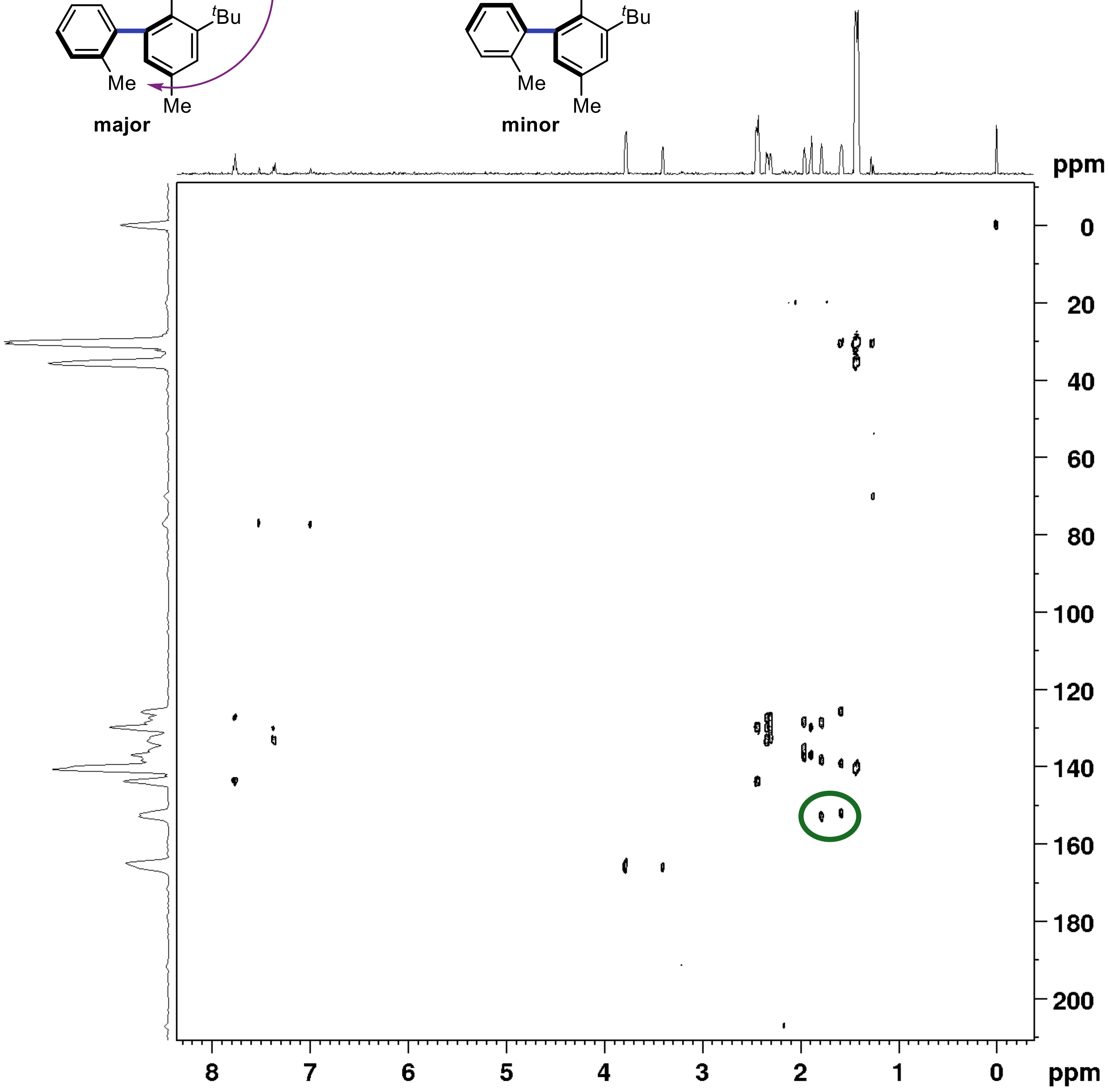

NOESY ($CDCl_3$, 400 MHz)

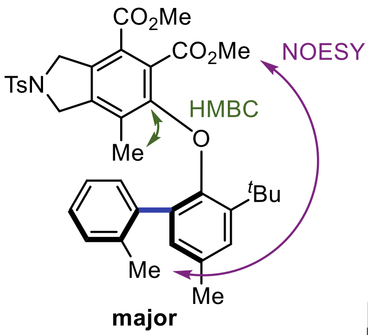

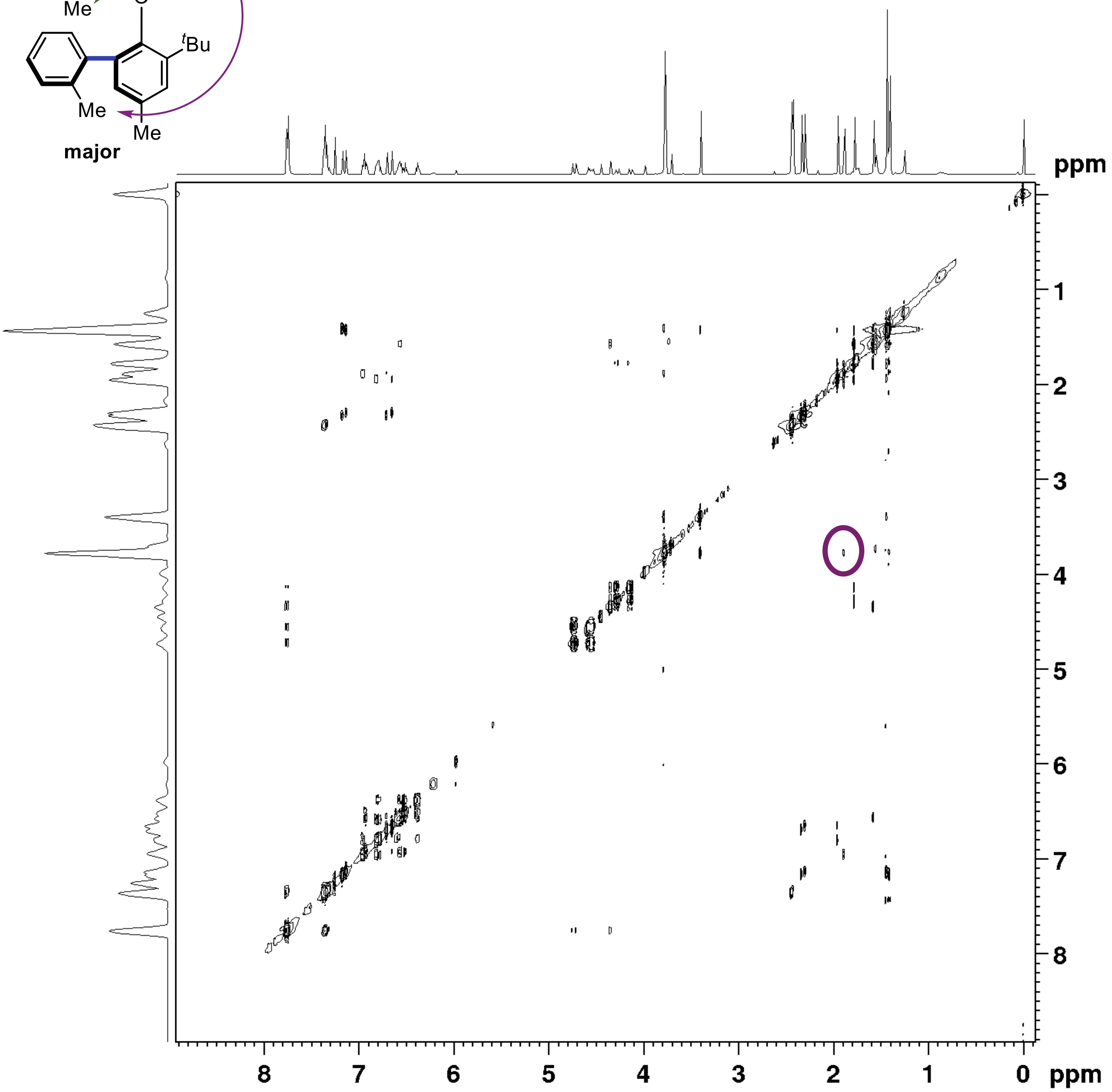

(+)-Methyl 6-((3-(*tert*-butyl)-4',5-dimethyl-[1,1'-biphenyl]-2-yl)oxy)-7-methyl-4-(4-nitrophenyl)-2-tosylisoindoline-5-carboxylate [(+)-3mc]

^{1}H NMR ($CDCl_3$, 400 MHz)

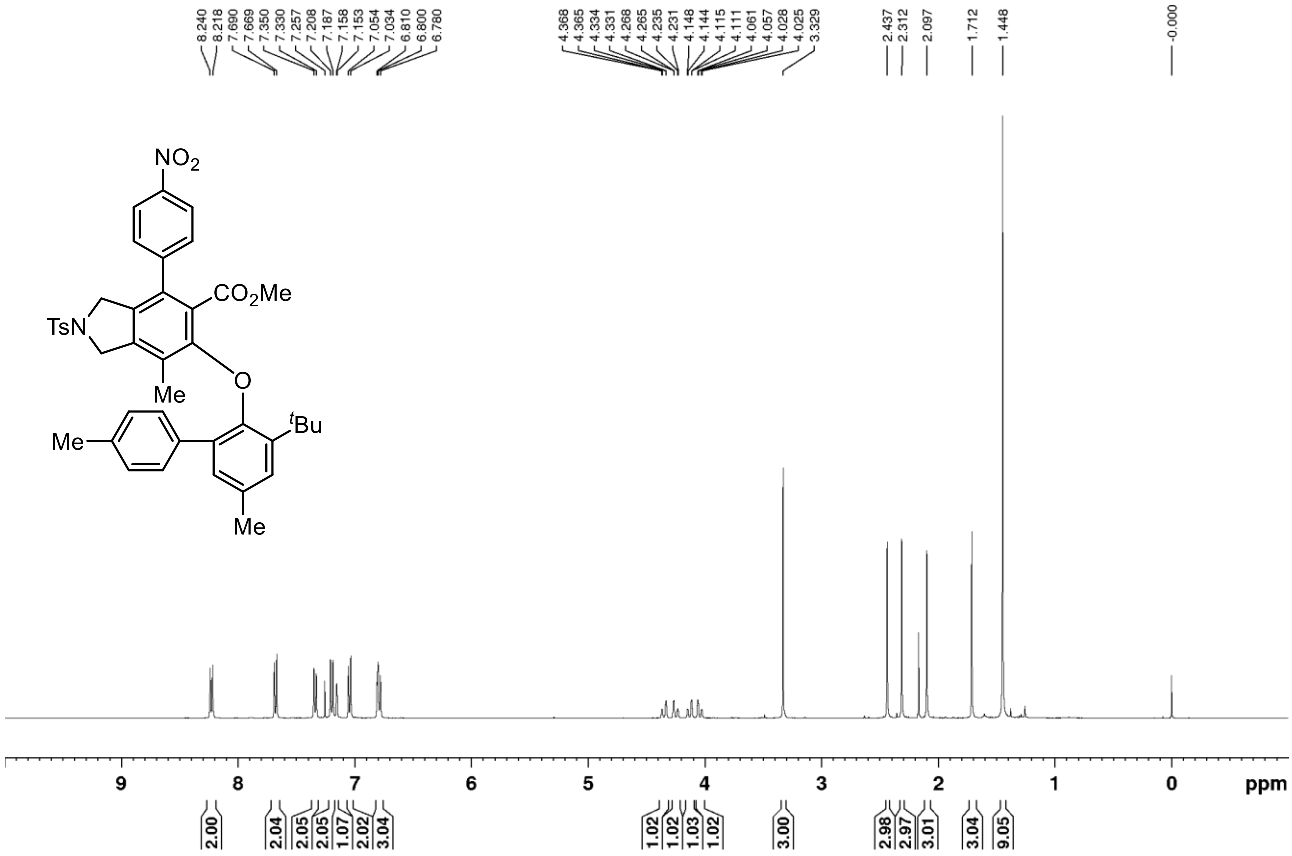

^{13}C NMR ($CDCl_3$, 101 MHz)

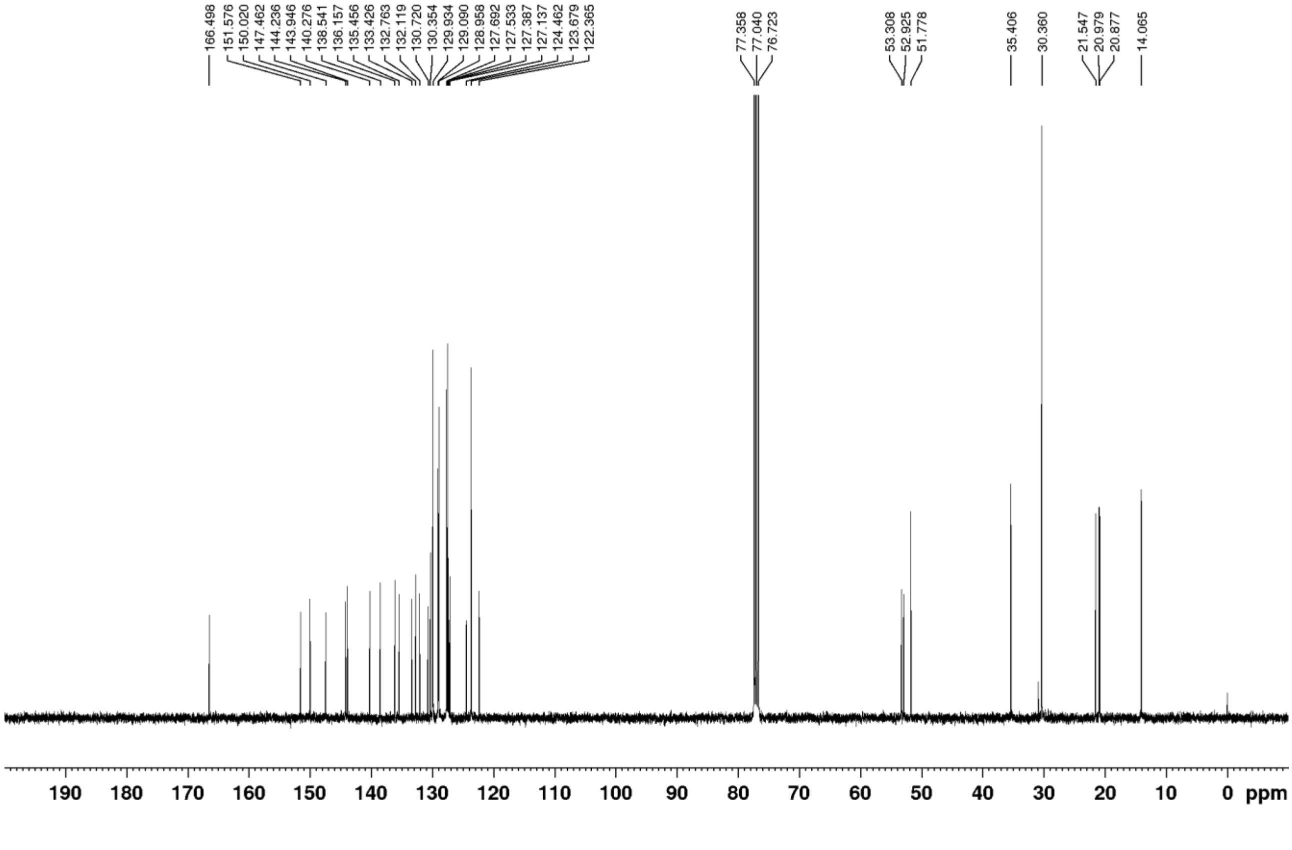

HMBC ($CDCl_3$, 400 MHz)

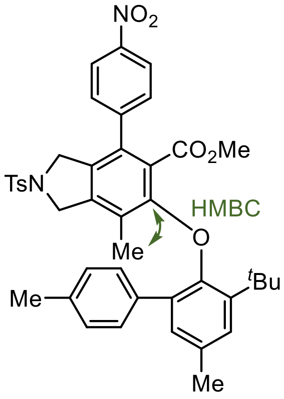

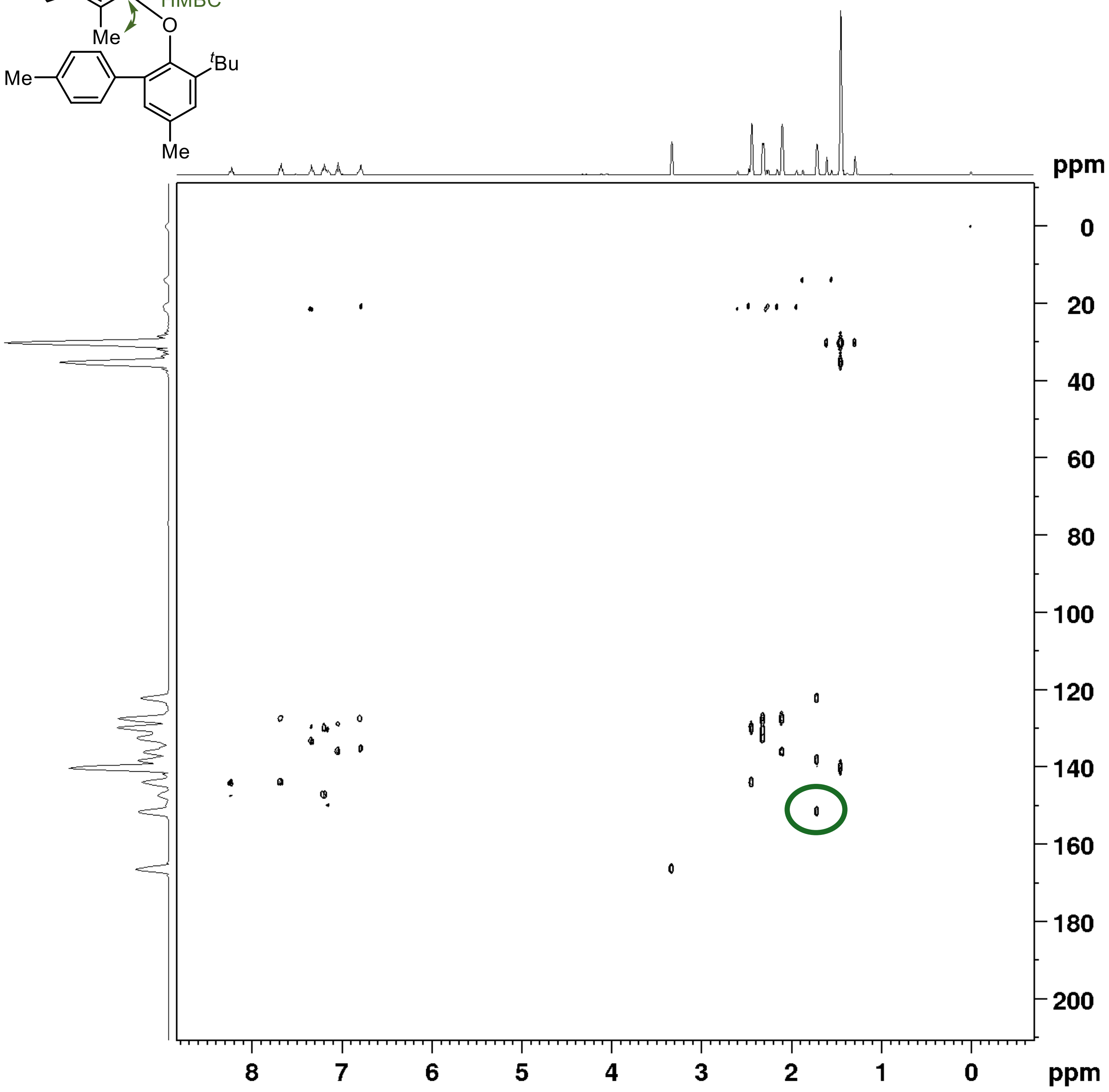

(*S*)-(+)-Methyl 4-(3,5-bis(trifluoromethyl)phenyl)-6-((3-(*tert*-butyl)-4',5-dimethyl-[1,1'-biphenyl]-2-yl)oxy)-7-methyl-2-tosylisoindoline-5-carboxylate [(*S*)-(+)-3gc]

^{1}H NMR ($CDCl_3$, 400 MHz)

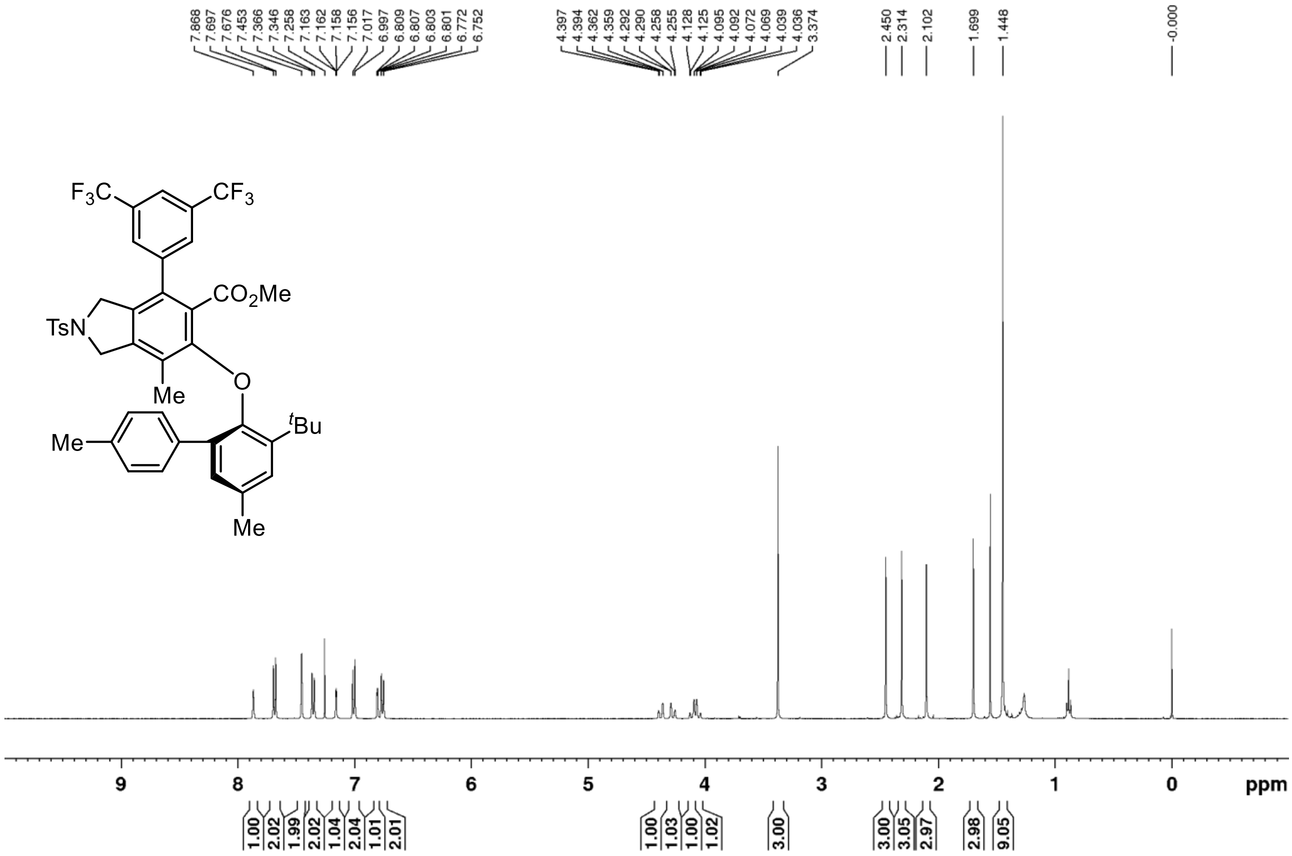

^{13}C NMR ($CDCl_3$, 101 MHz)

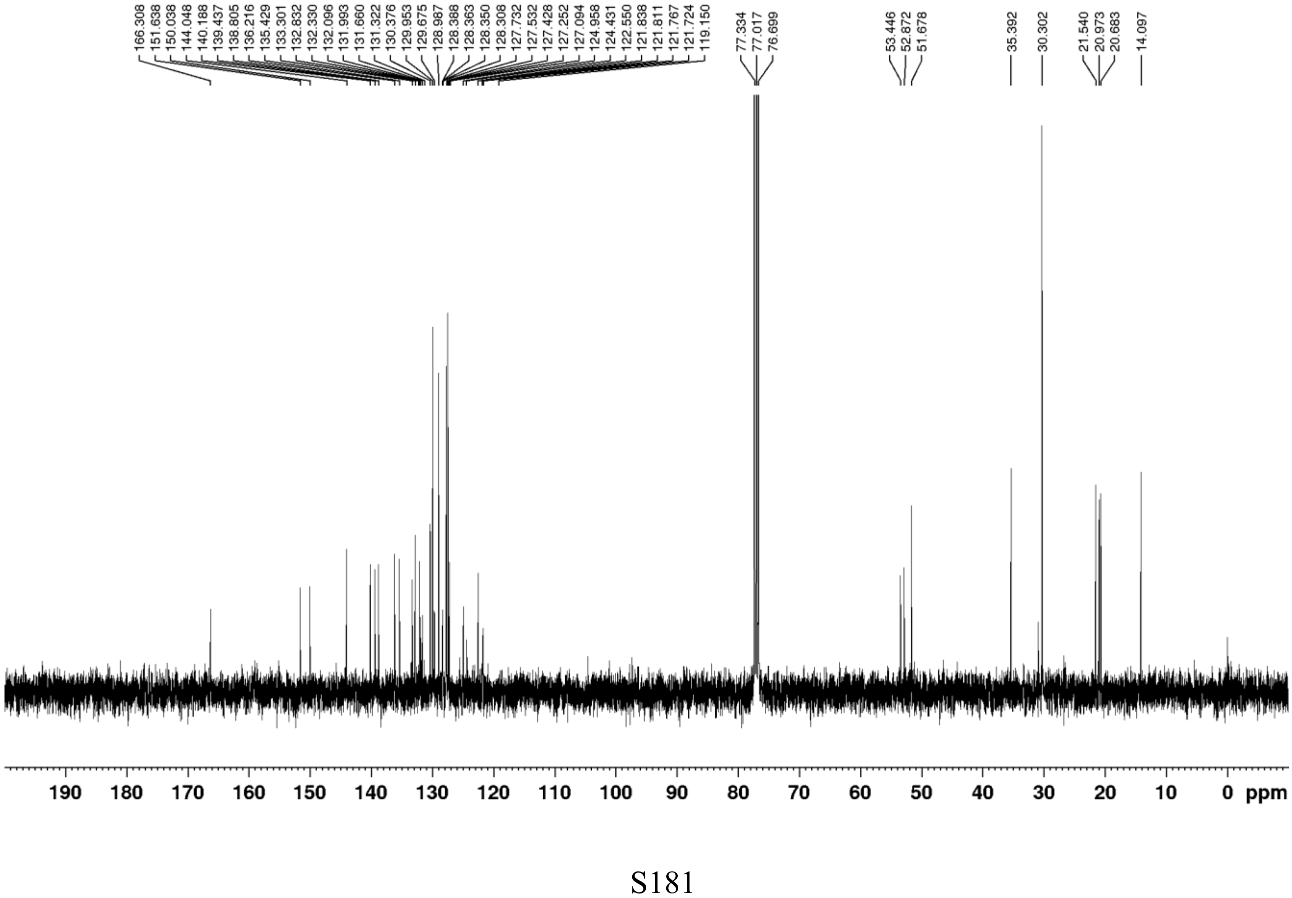

^{19}F NMR ($CDCl_3$, 377 MHz)

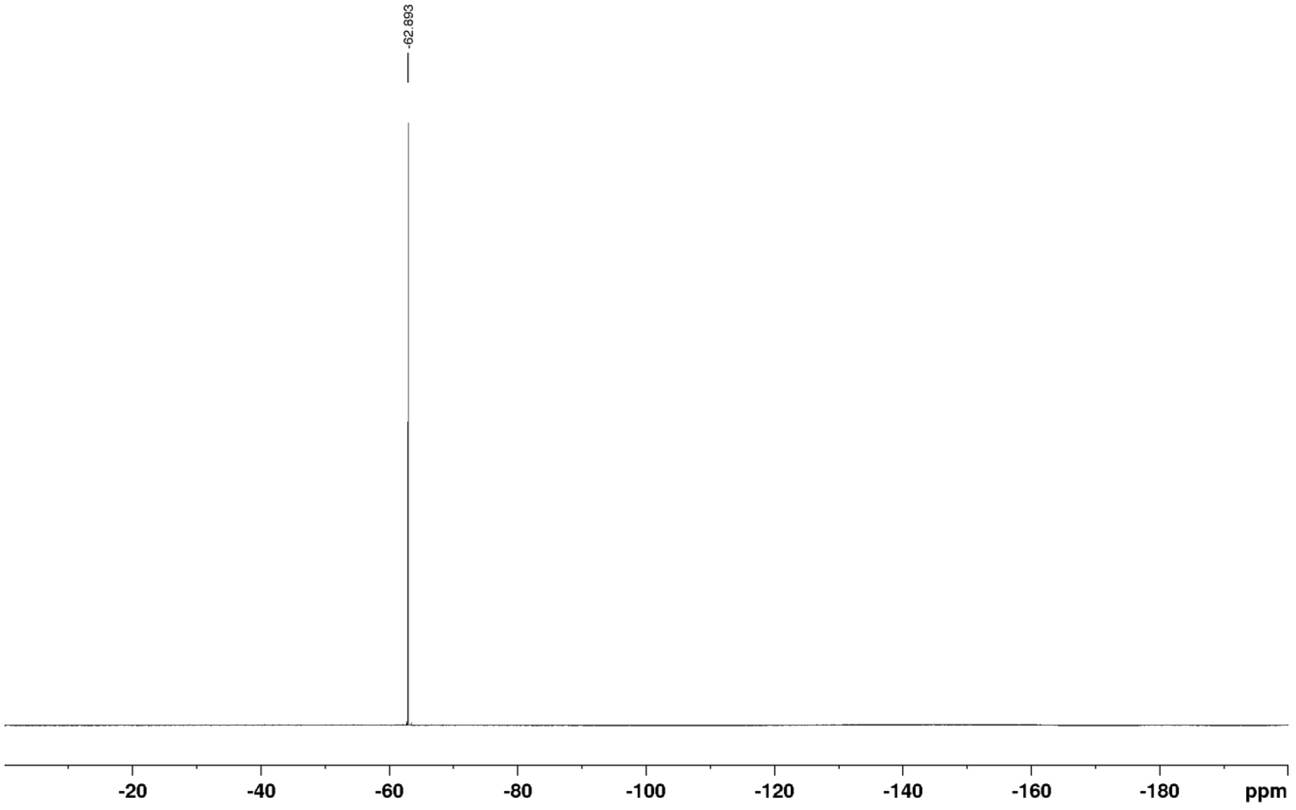

HMBC ($CDCl_3$, 400 MHz)

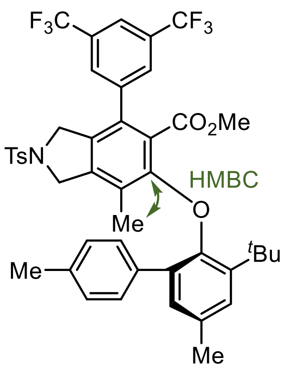

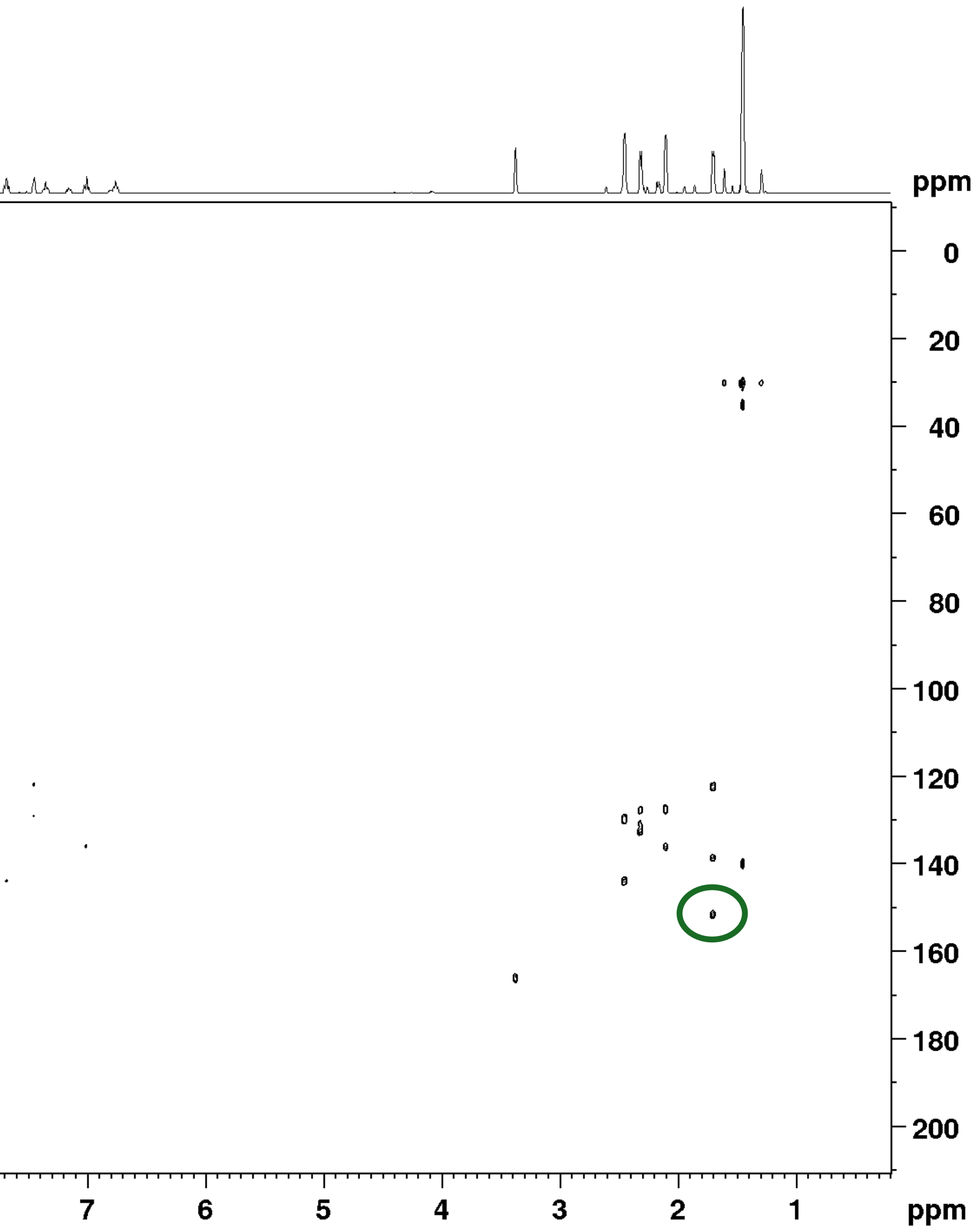

(*S*,*R*)-(–)-Methyl 6-(2-(*tert*-butyl)-6-methylphenoxy)-7-methyl-4-(naphthalen-1-yl)-2-tosylisoindoline-5-carboxylate [(*S*,*R*)-(–)-3na]

^{1}H NMR ($CDCl_3$, 400 MHz)

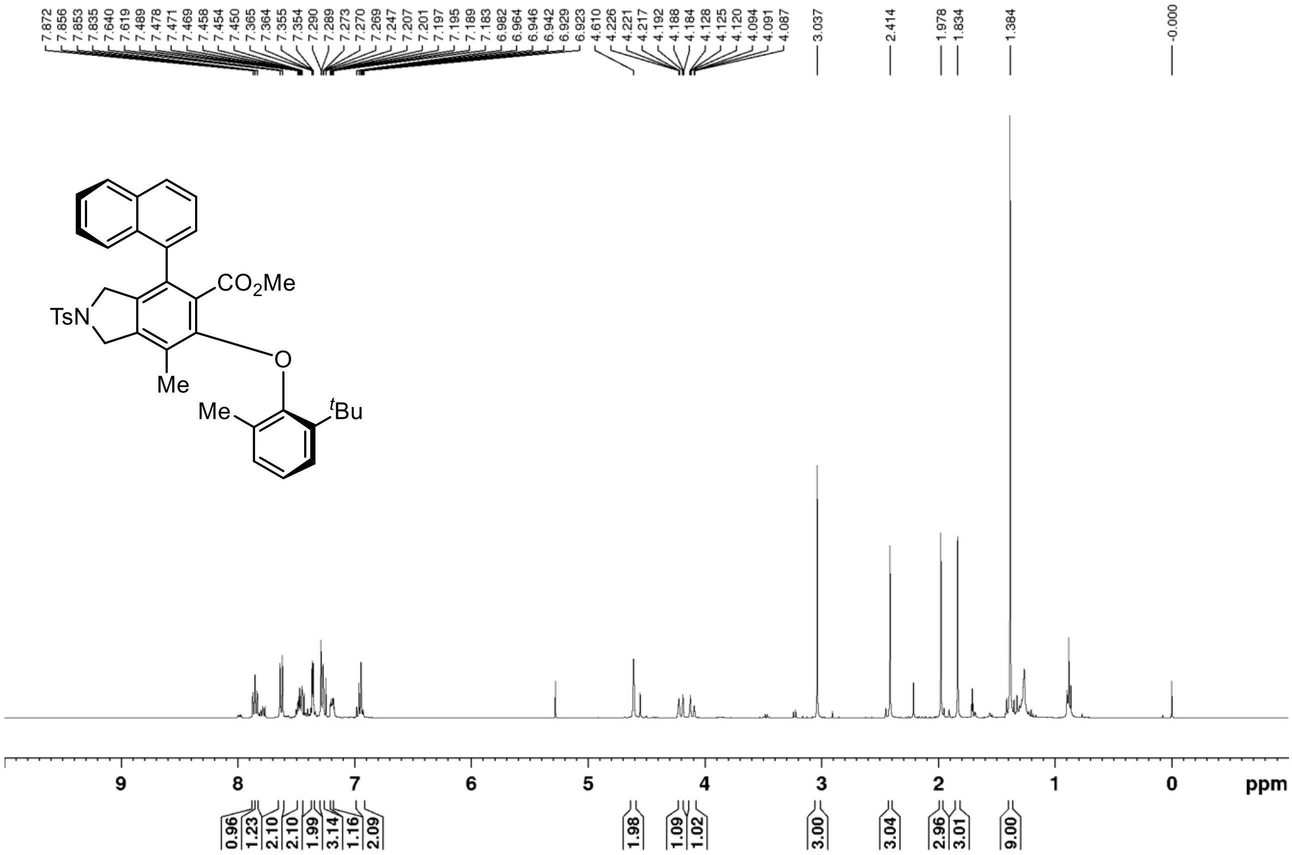

^{13}C NMR ($CDCl_3$, 101 MHz)

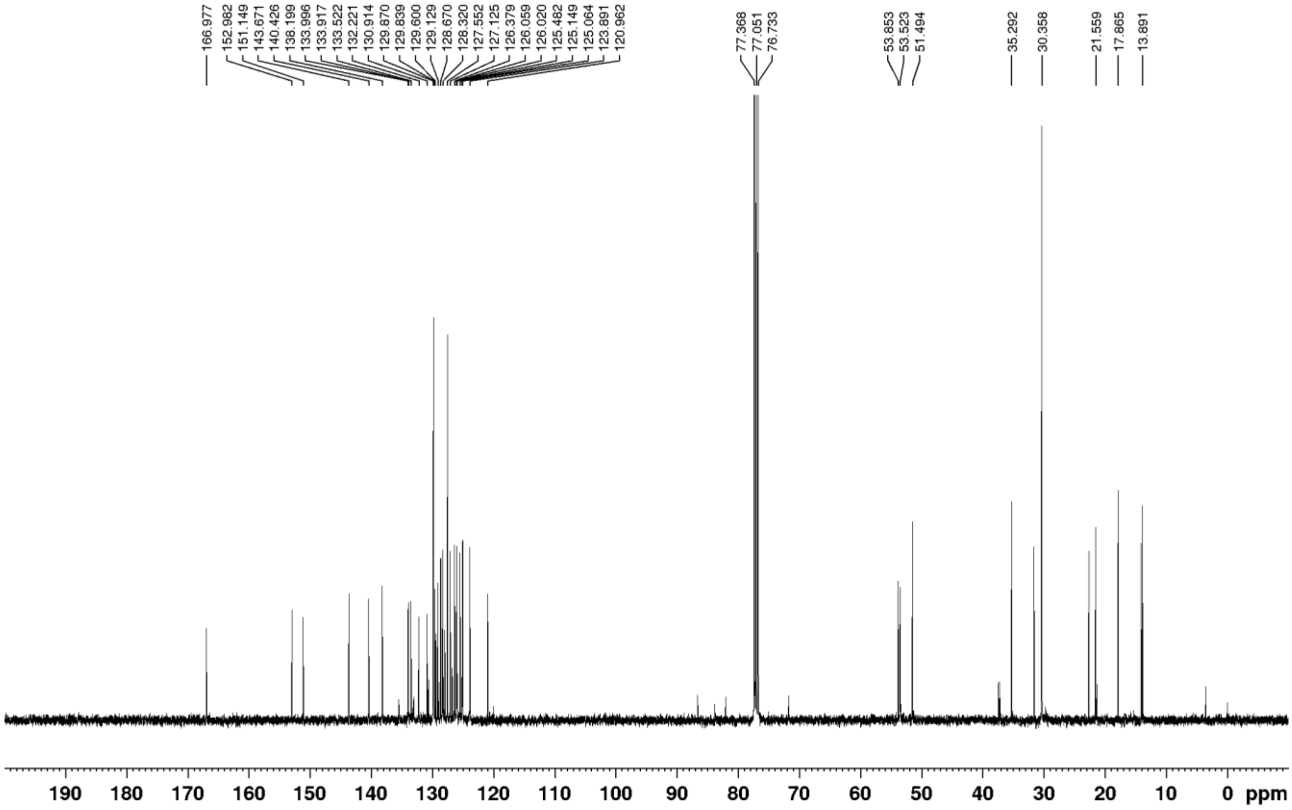

HMBC ($CDCl_3$, 400 MHz)

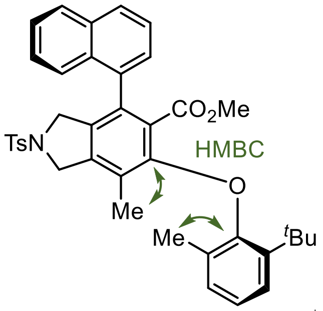

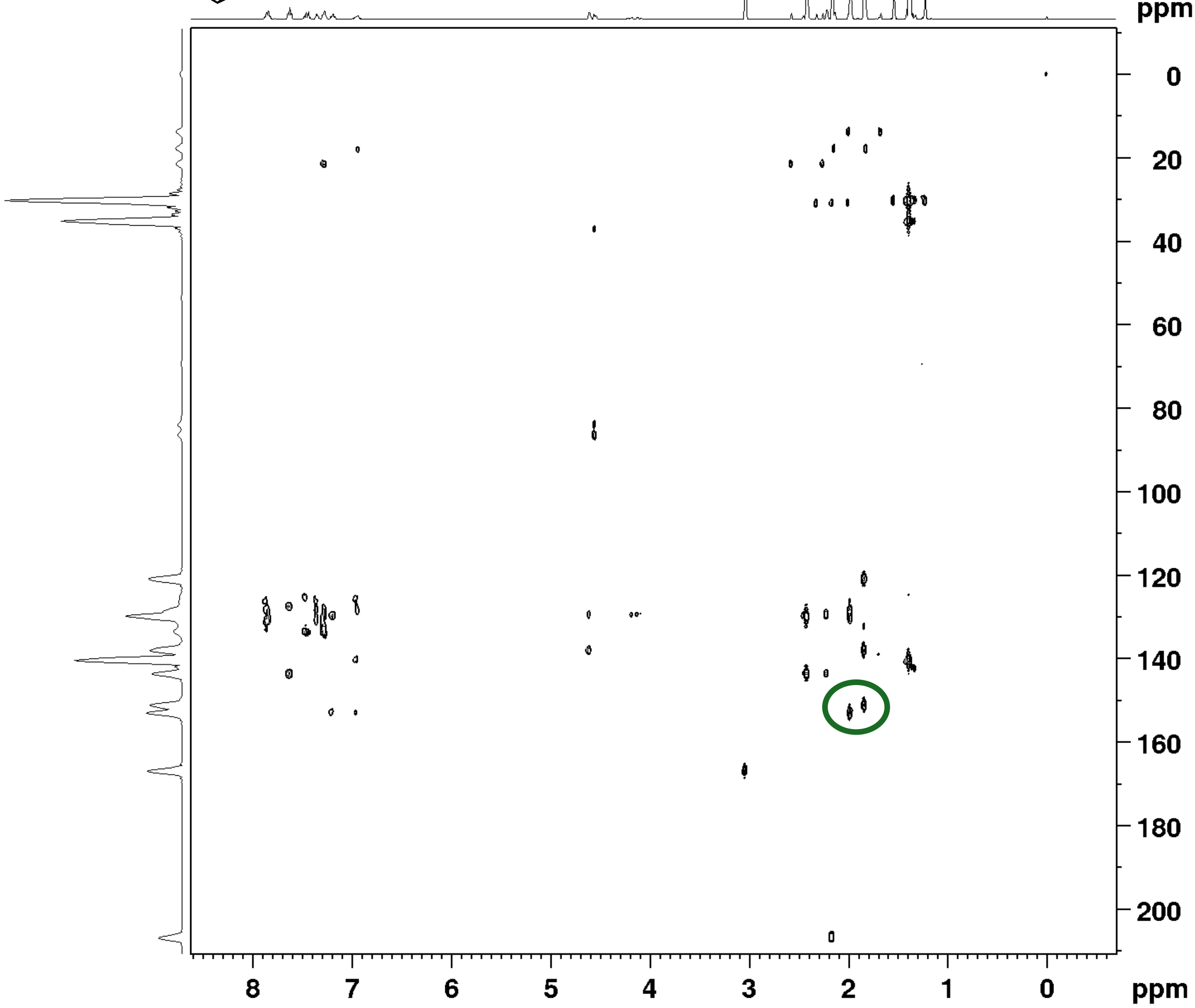

(–)-Methyl 6-(2-(*tert*-butyl)-6-methylphenoxy)-7-methyl-4-(phenanthren-9-yl)-2-tosylisoindoline-5-carboxylate [(–)-3oa]

^{1}H NMR ($CDCl_3$, 400 MHz)

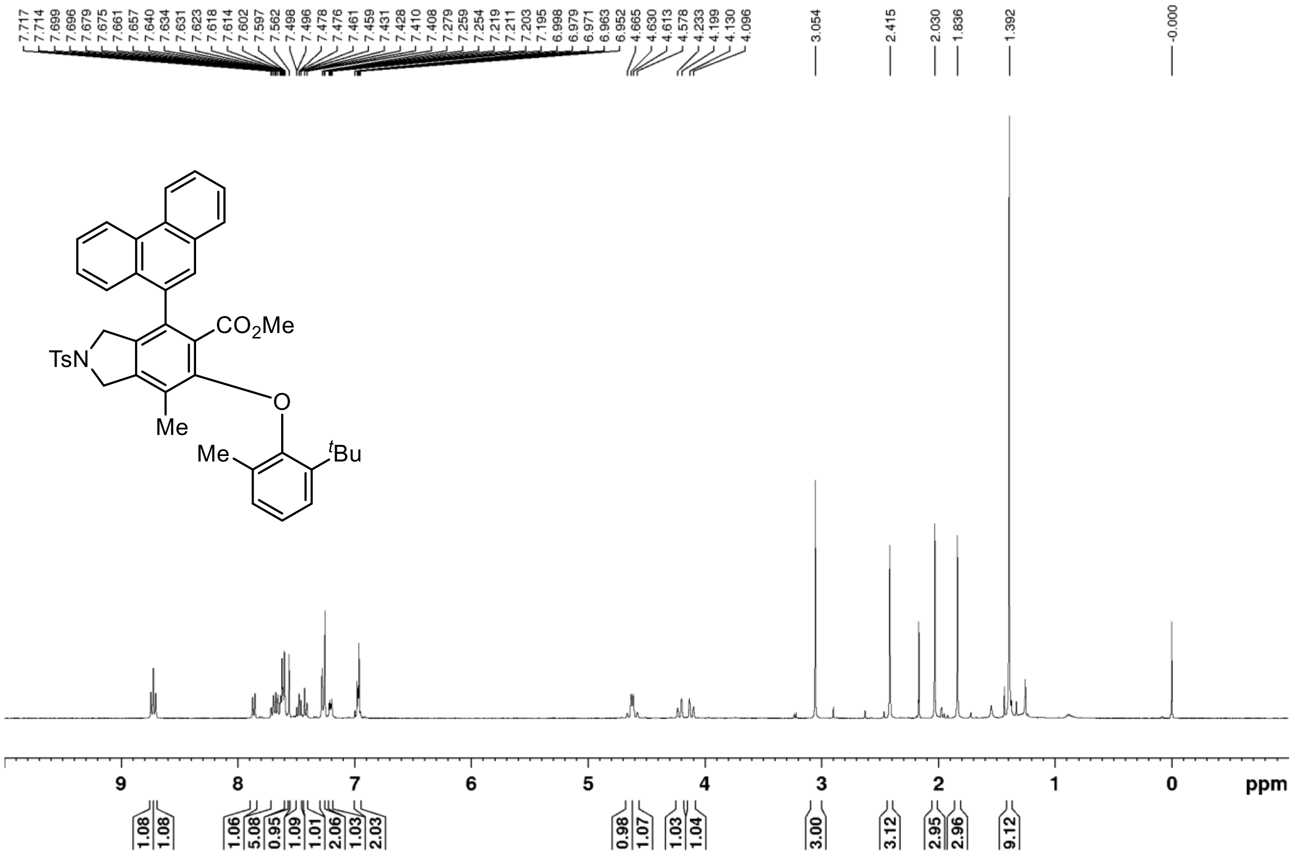

^{13}C NMR ($CDCl_3$, 101 MHz)

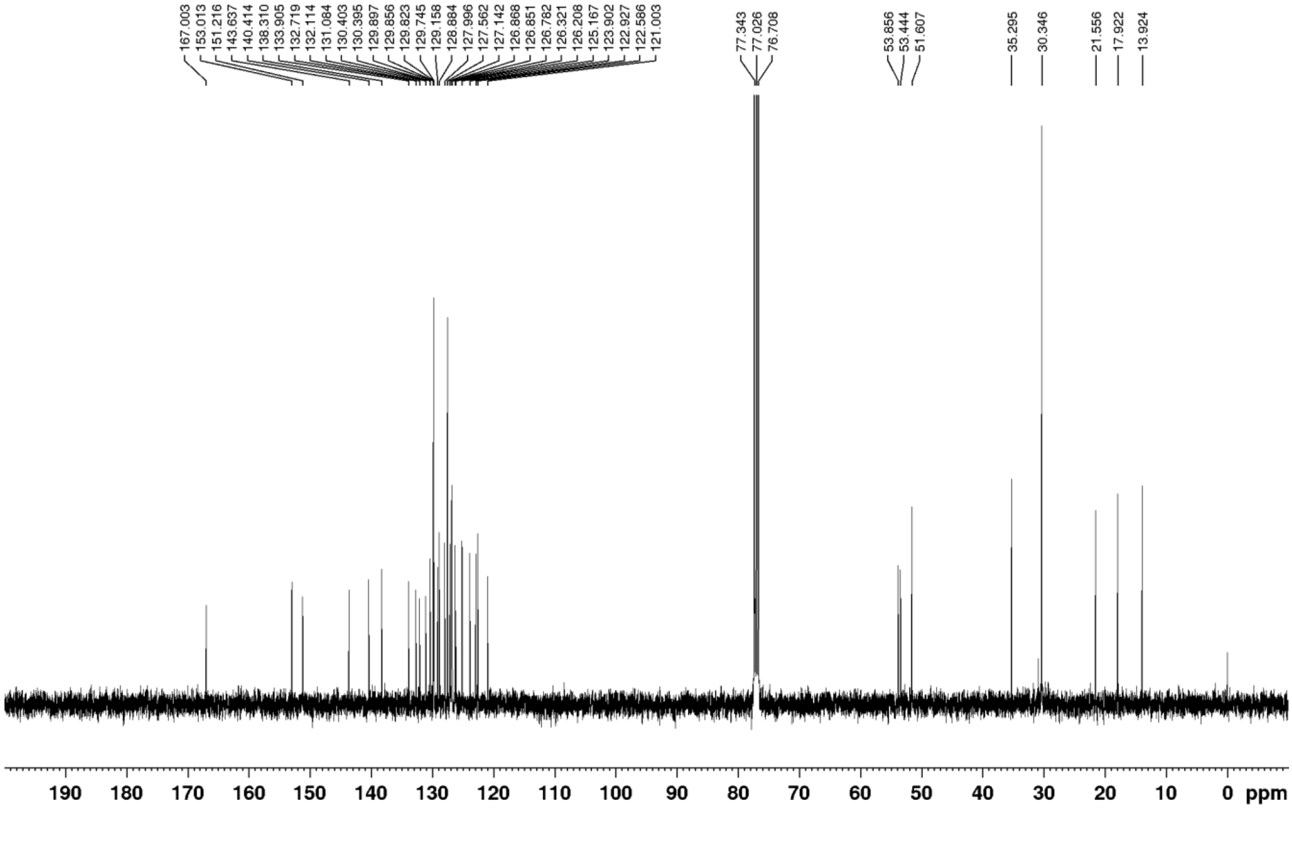

HMBC ($CDCl_3$, 400 MHz)

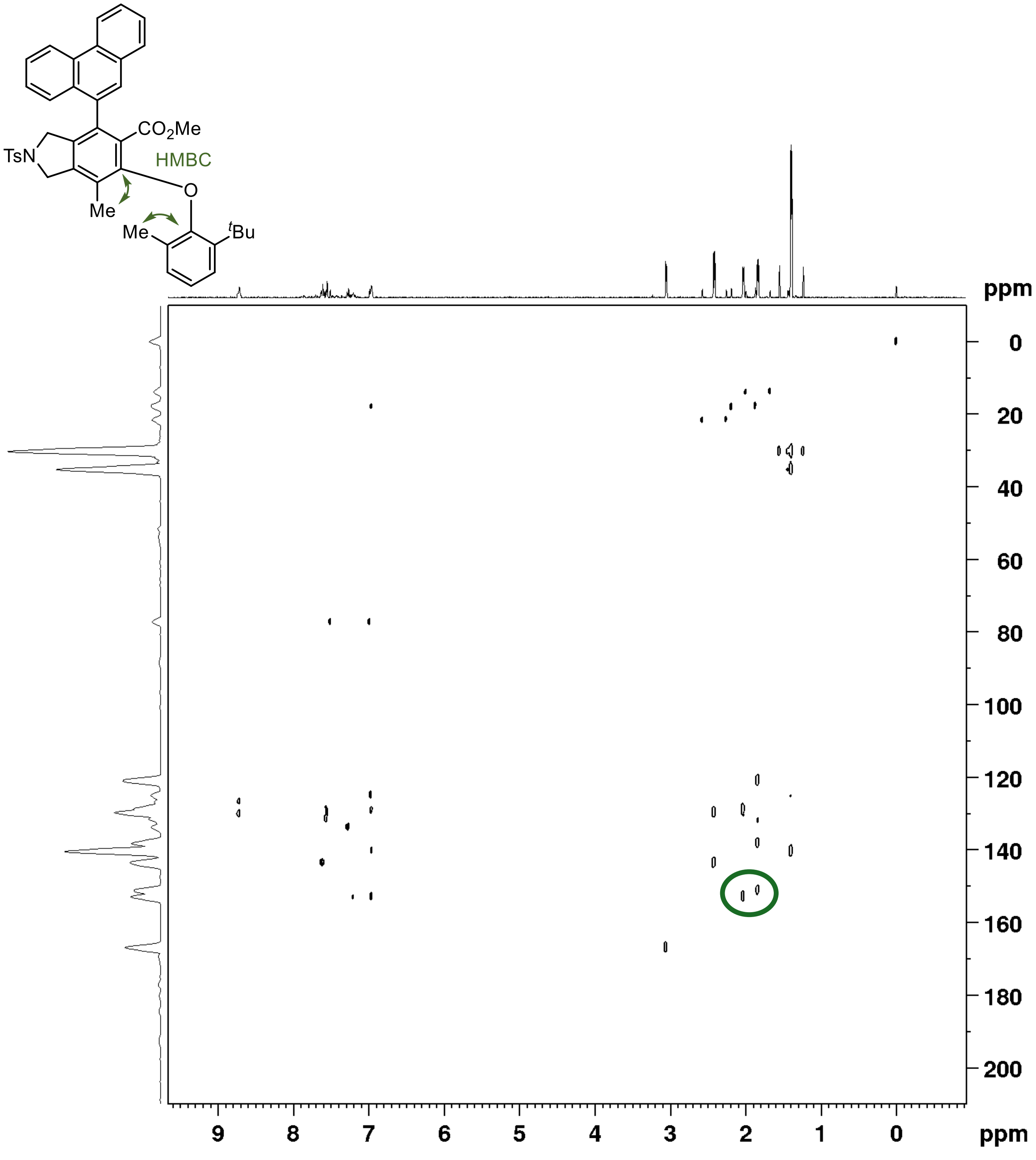

(*S,R*)-(–)-Methyl 6-(2-(*tert*-butyl)-6-methylphenoxy)-7-methyl-4-(pyren-4-yl)-2-tosylisoindoline-5-carboxylate [(*S,R*)-(–)-3pa]

^{1}H NMR ($CDCl_3$, 400 MHz)

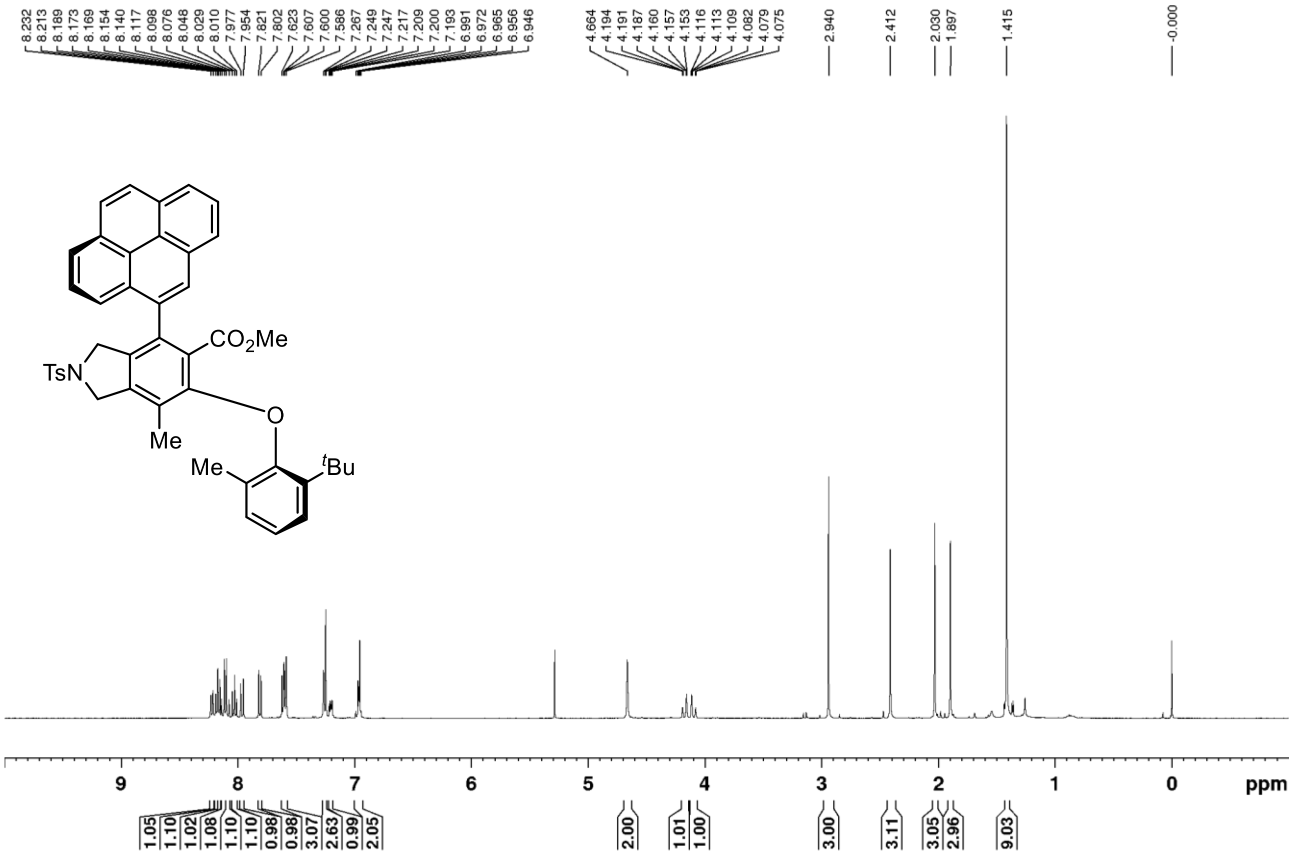

^{13}C NMR ($CDCl_3$, 101 MHz)

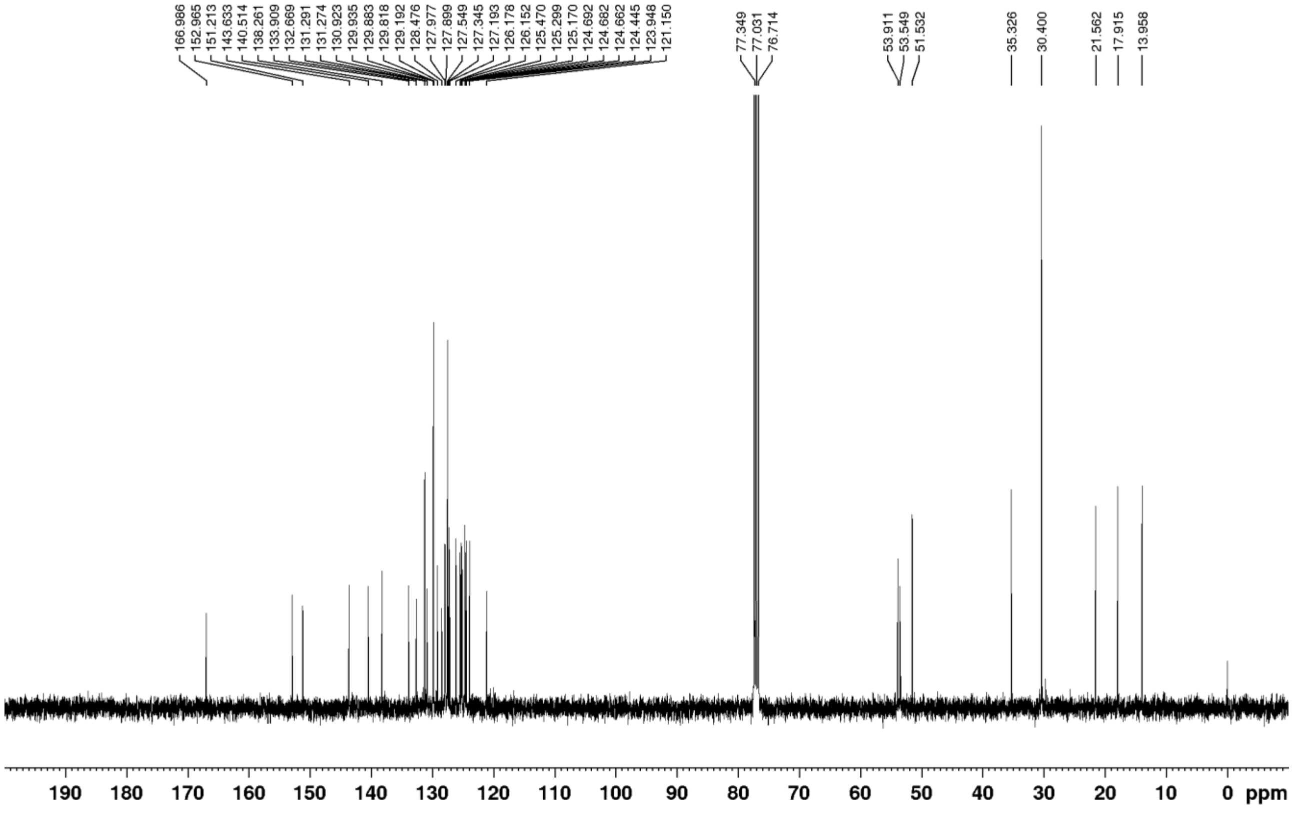

HMBC ($CDCl_3$, 400 MHz)

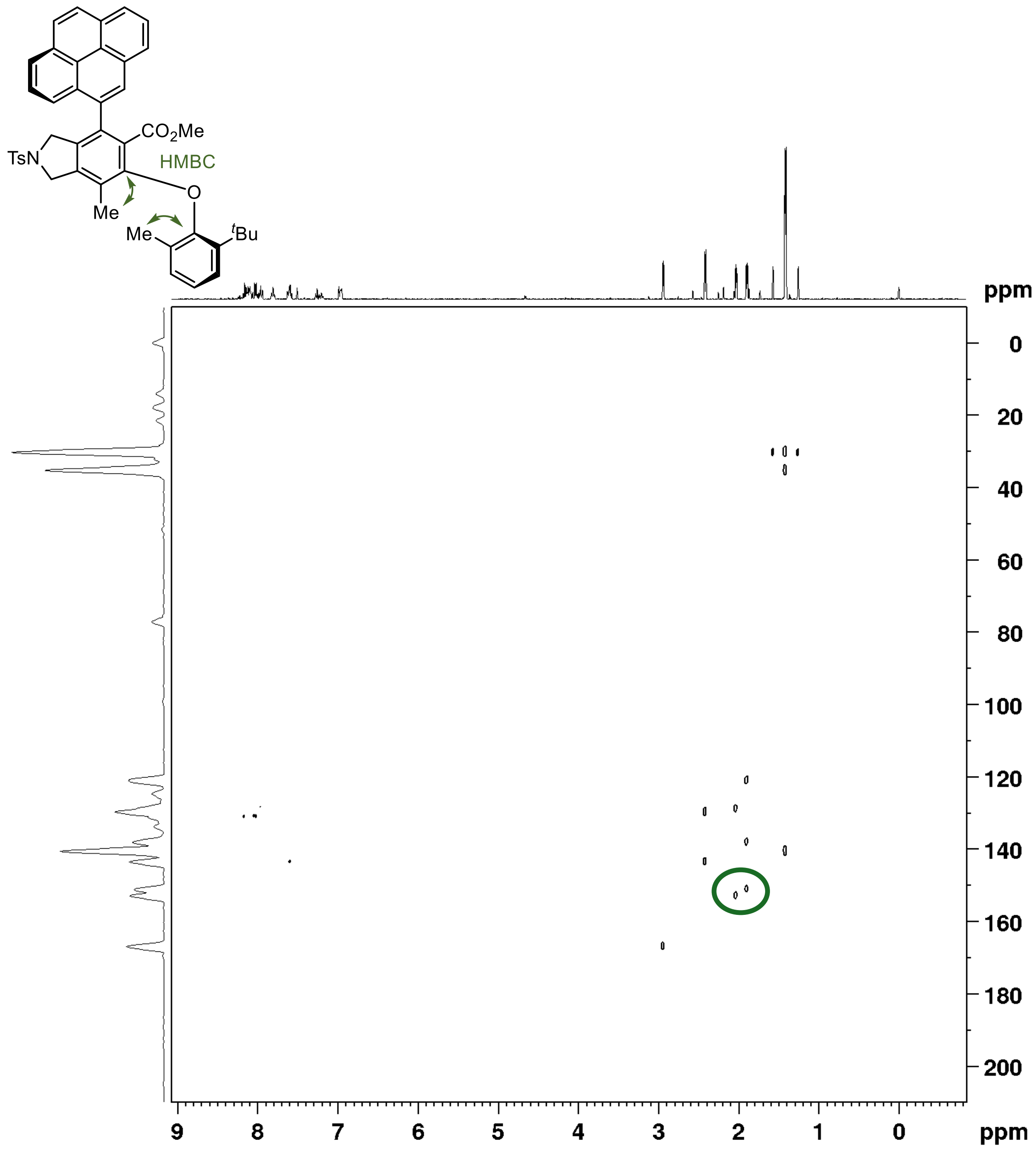

(–)-Methyl 6-((3-(*tert*-butyl)-4',5-dimethyl-[1,1'-biphenyl]-2-yl)oxy)-7-methyl-4-(naphthalen-1-yl)-2-tosylisoindoline-5-carboxylate [(–)-3nc]

^{1}H NMR ($CDCl_3$, 400 MHz)

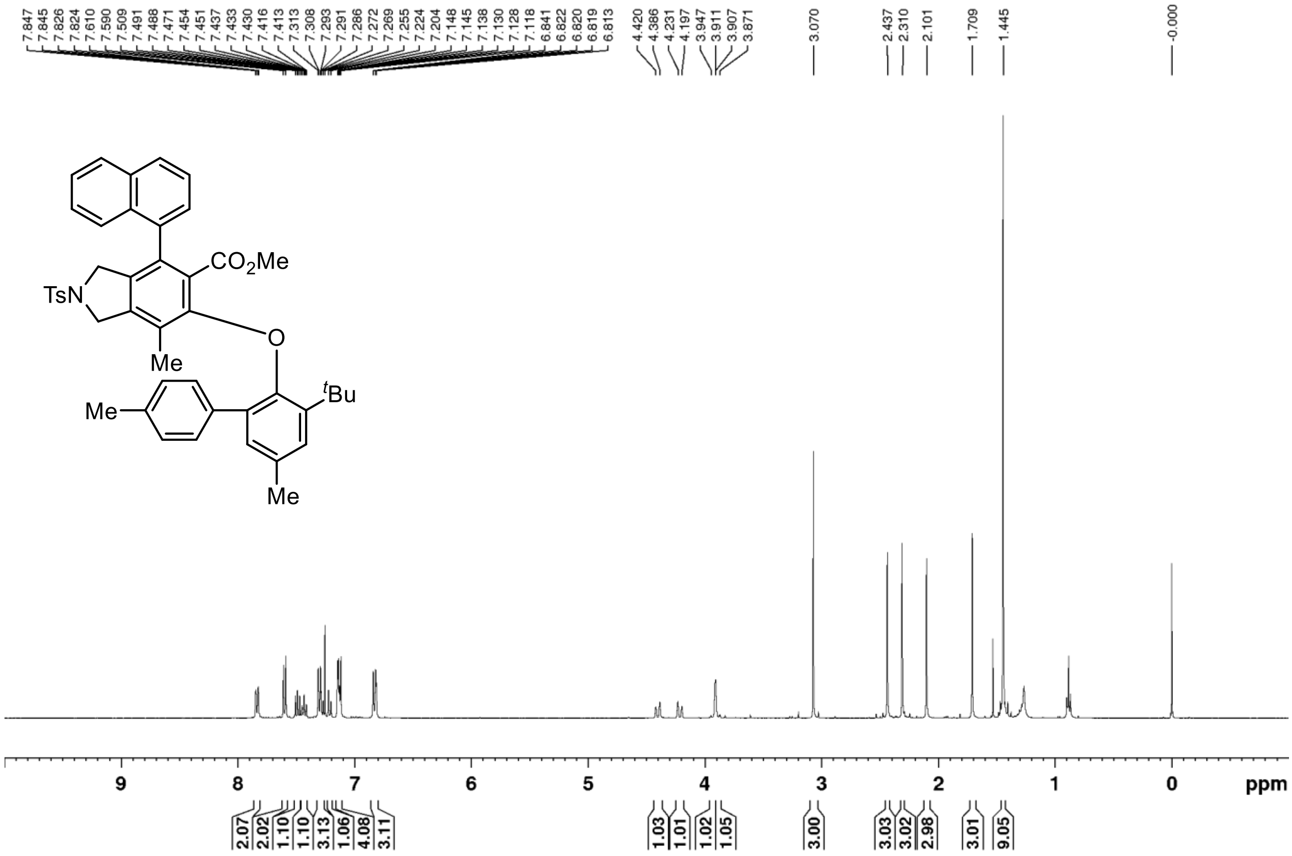

^{13}C NMR ($CDCl_3$, 101 MHz)

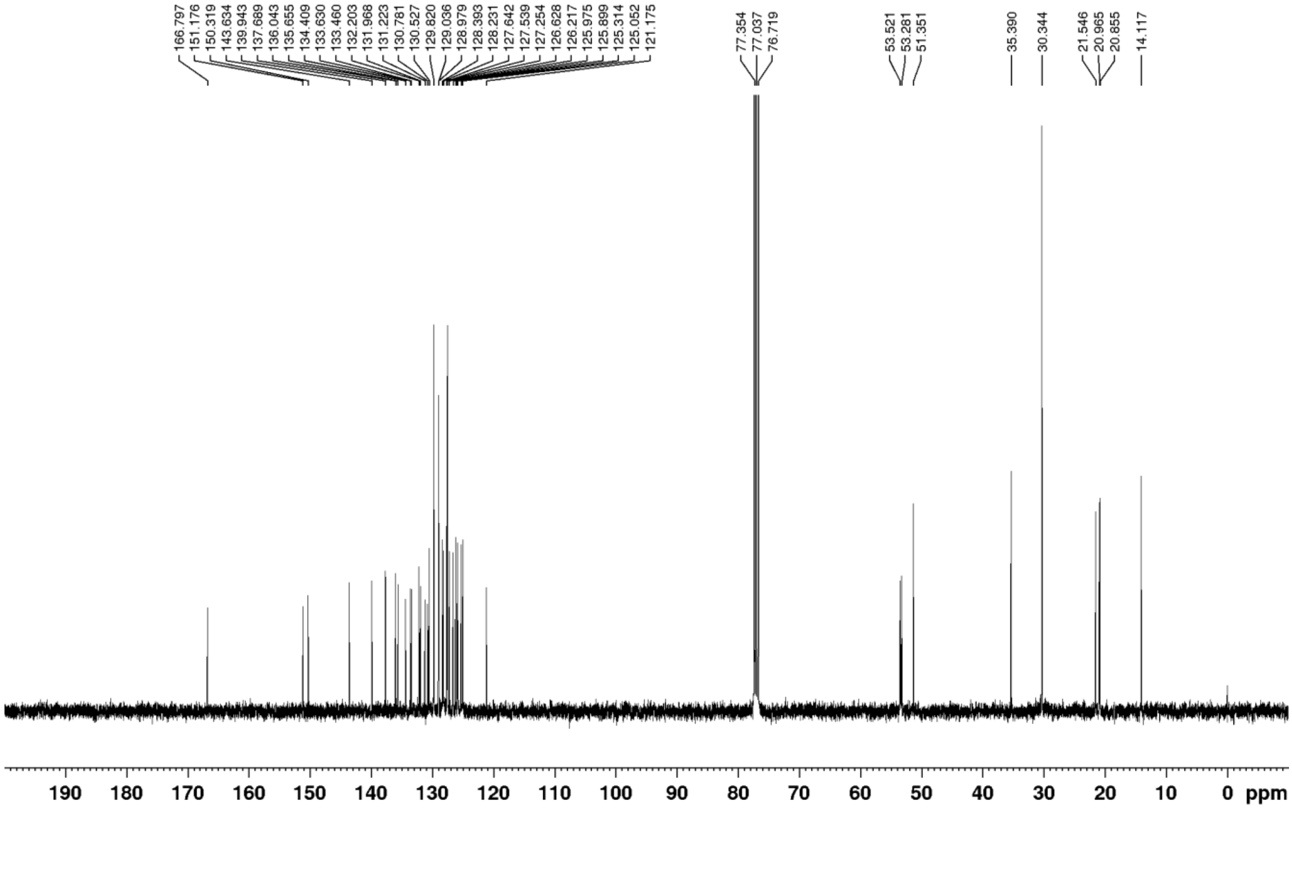

HMBC ($CDCl_3$, 400 MHz)

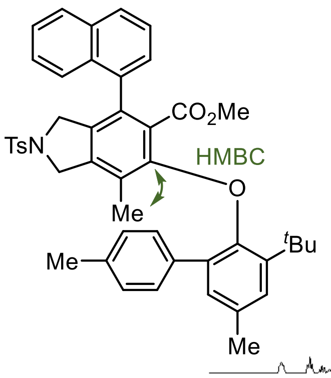

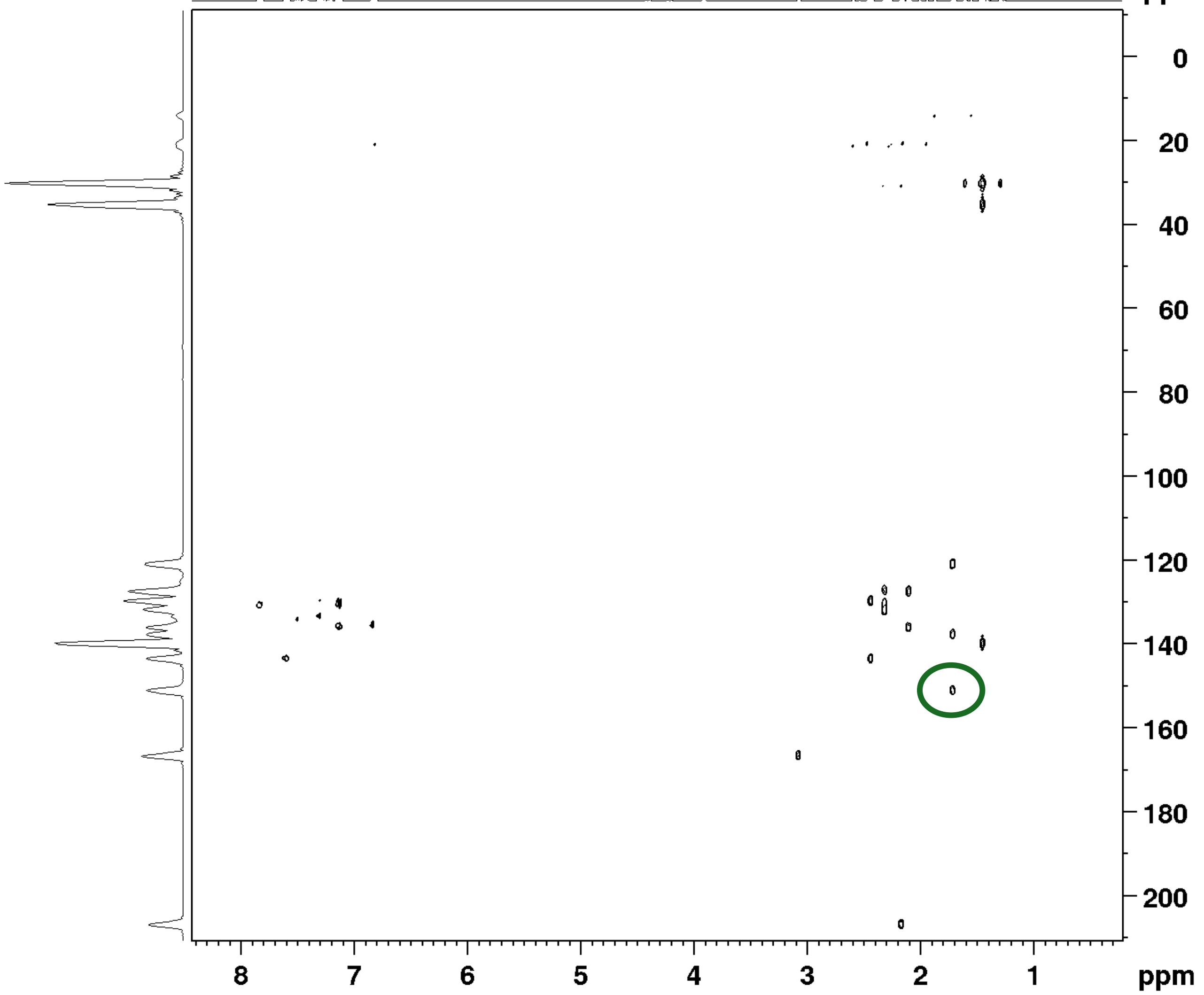

(–)-Methyl 6-((3-(*tert*-butyl)-4',5-dimethyl-[1,1'-biphenyl]-2-yl)oxy)-7-methyl-4-(phenanthren-9-yl)-2-tosylisoindoline-5-carboxylate [(–)-3oc]

^{1}H NMR ($CDCl_3$, 400 MHz)

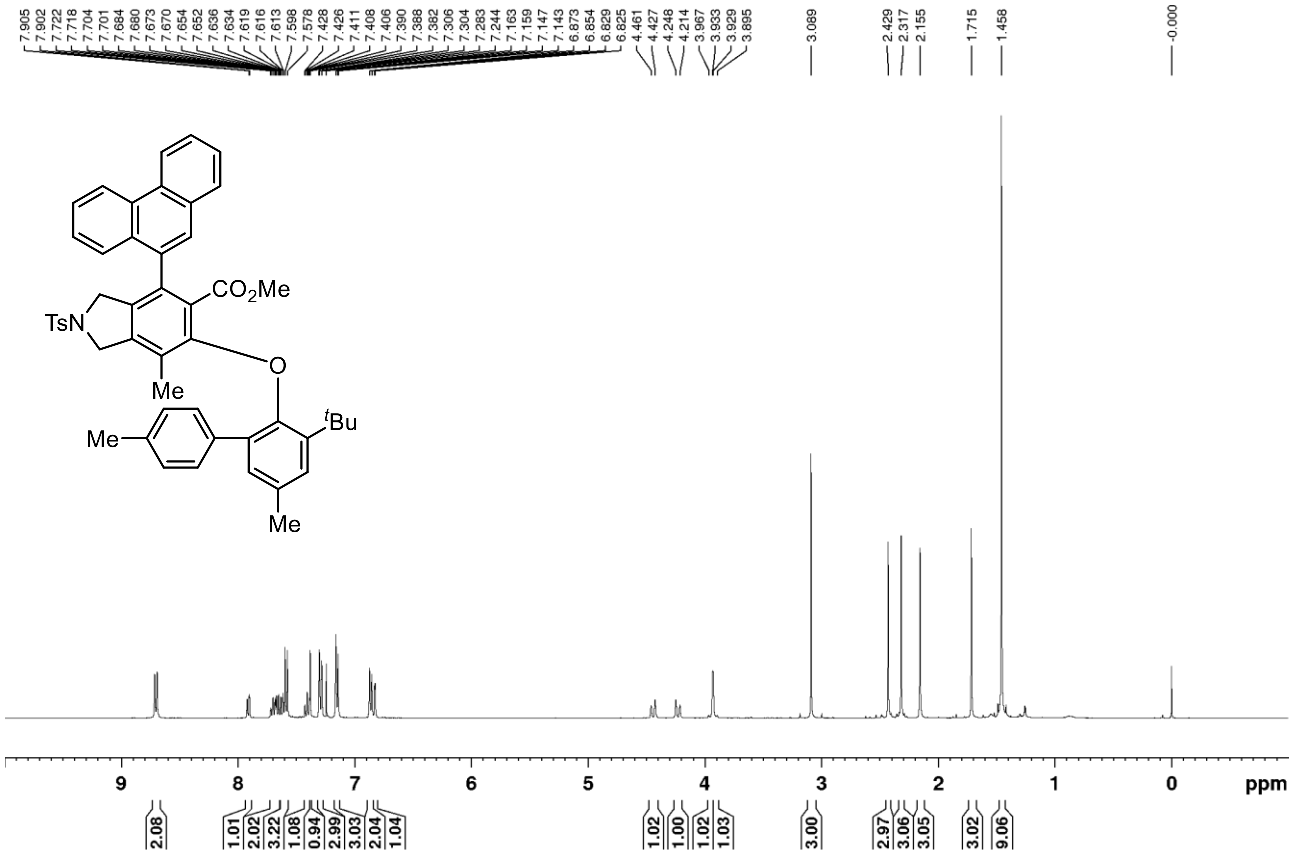

^{13}C NMR ($CDCl_3$, 101 MHz)

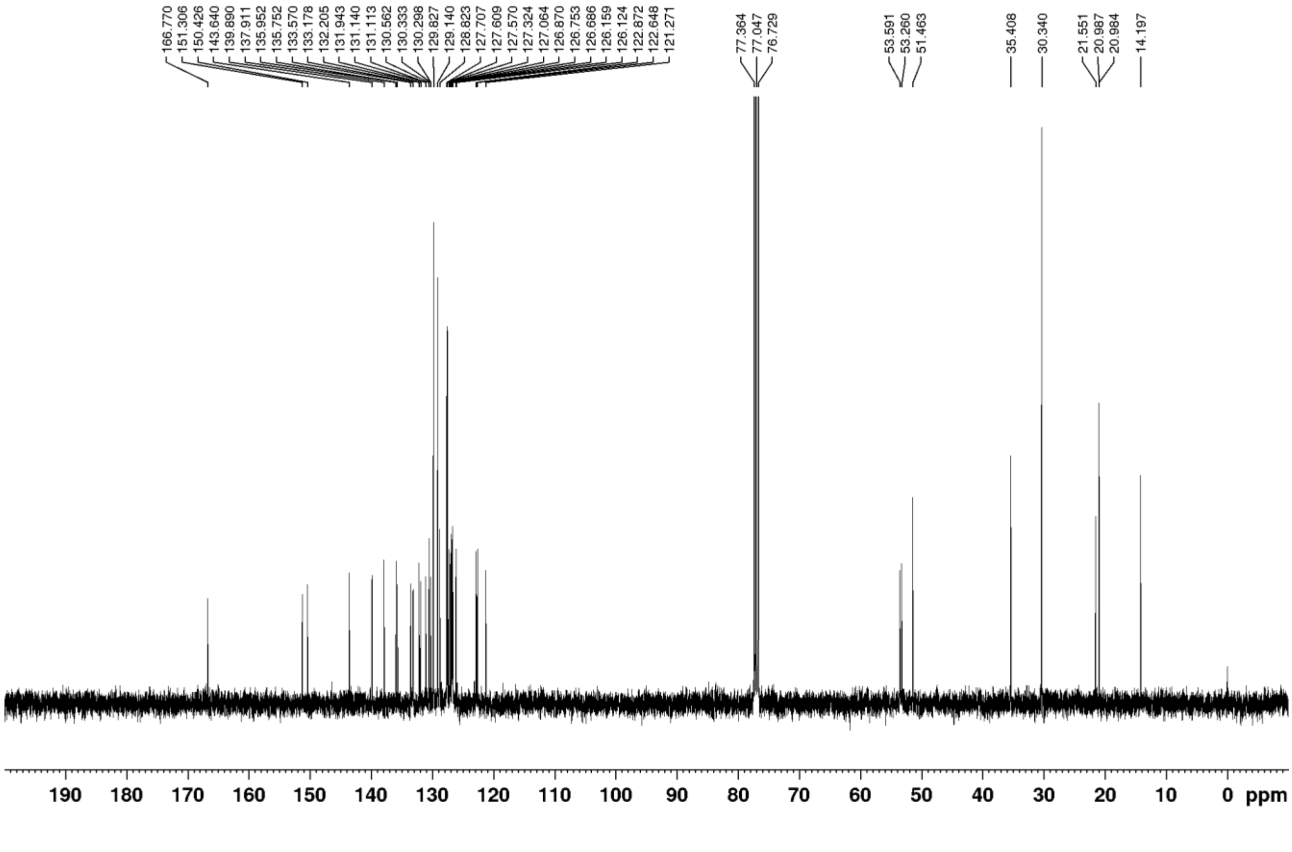

HMBC ($CDCl_3$, 400 MHz)

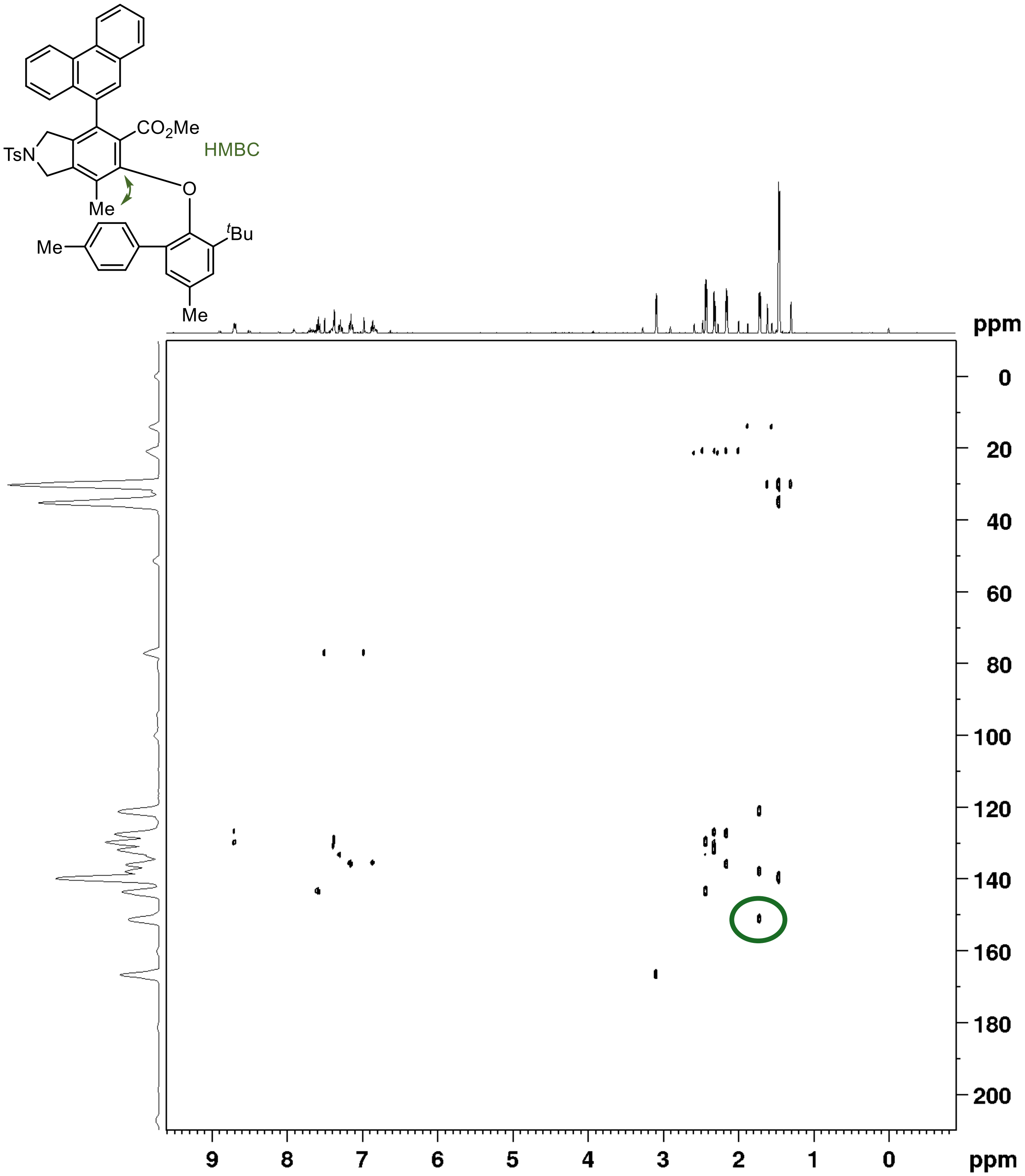

(–)-Methyl 6-((3-(*tert*-butyl)-4',5-dimethyl-[1,1'-biphenyl]-2-yl)oxy)-7-methyl-4-(pyren-4-yl)-2-tosylisoindoline-5-carboxylate [(–)-3pc]

^{1}H NMR ($CDCl_3$, 400 MHz)

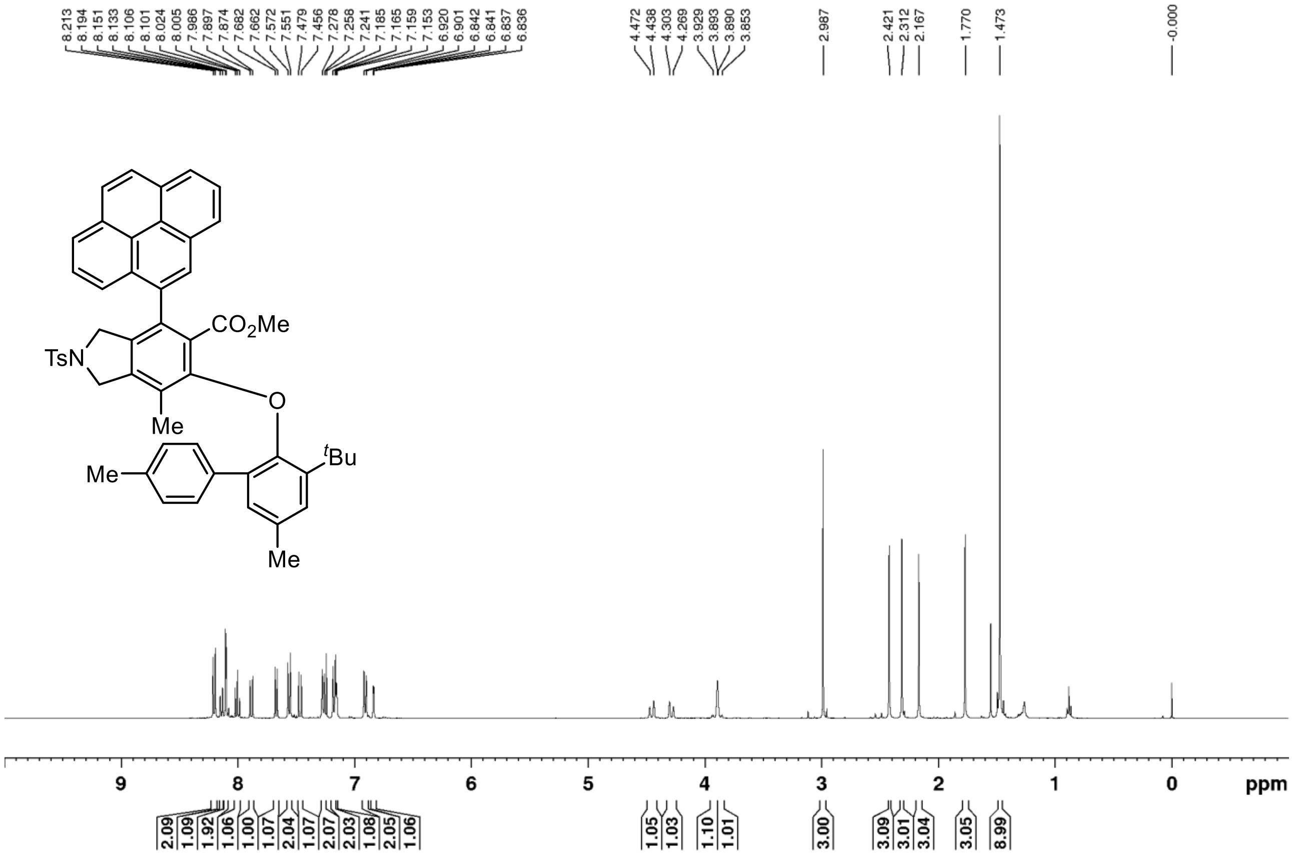

^{13}C NMR ($CDCl_3$, 101 MHz)

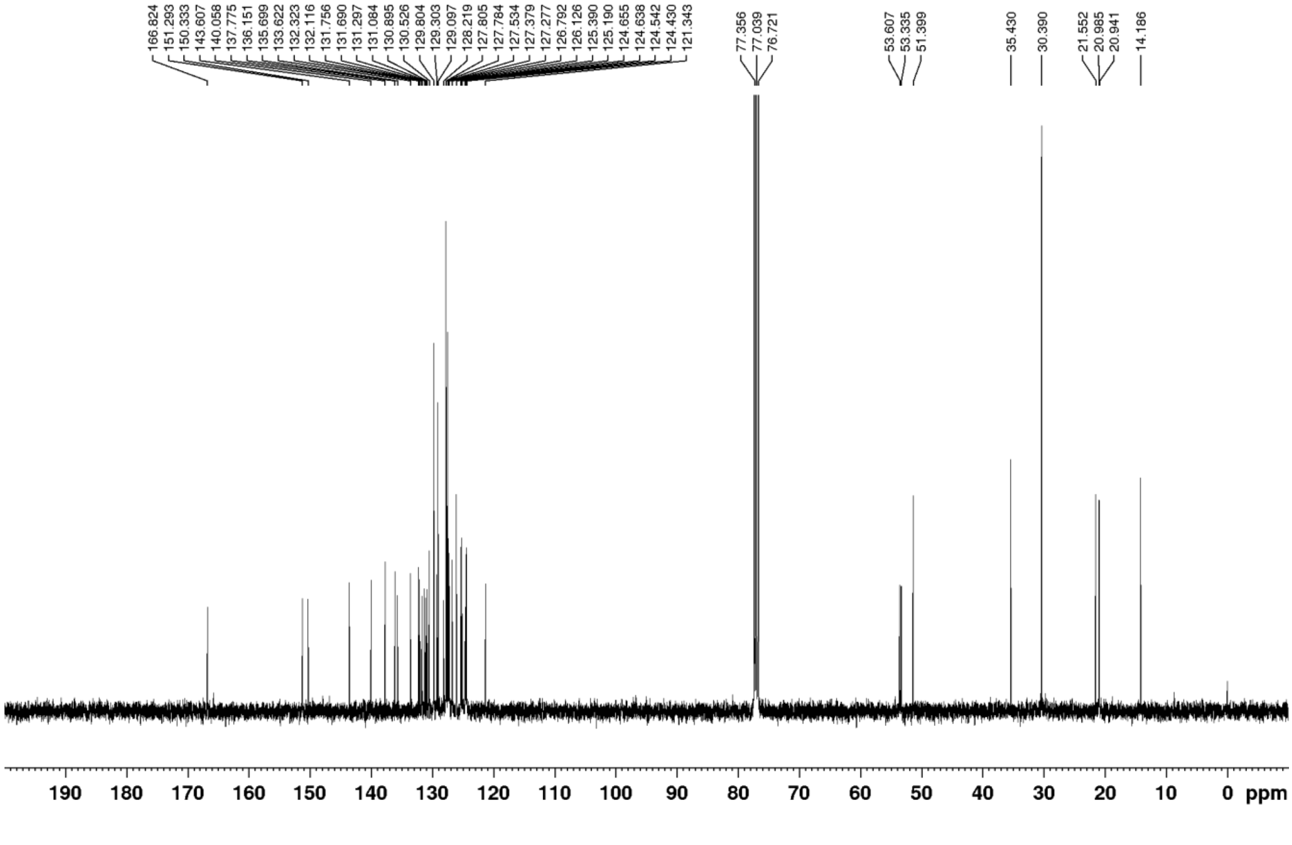

HMBC ($CDCl_3$, 400 MHz)

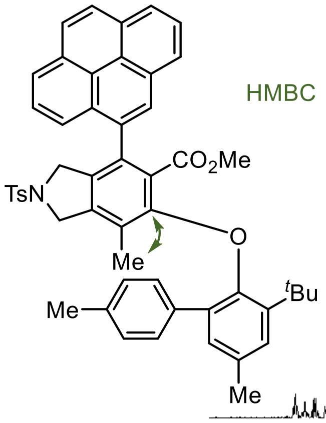

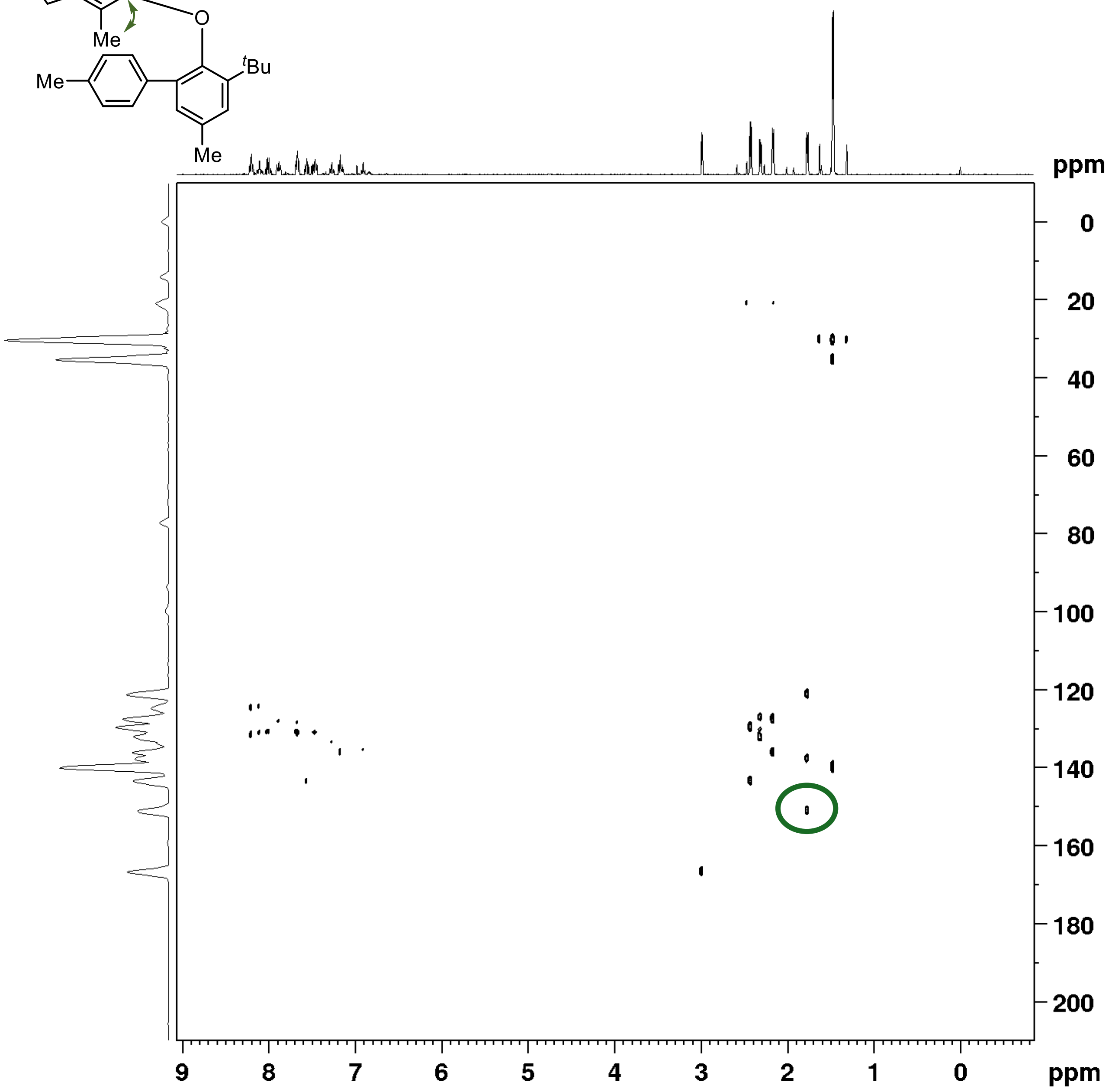

(–)-Methyl 6-(2-(*tert*-butyl)-6-methylphenoxy)-4-(1H-indol-7-yl)-7-methyl-2-tosylisoindoline-5-carboxylate [(–)-3qa]

^{1}H NMR [**3qa**] ($CDCl_3$, 400 MHz)

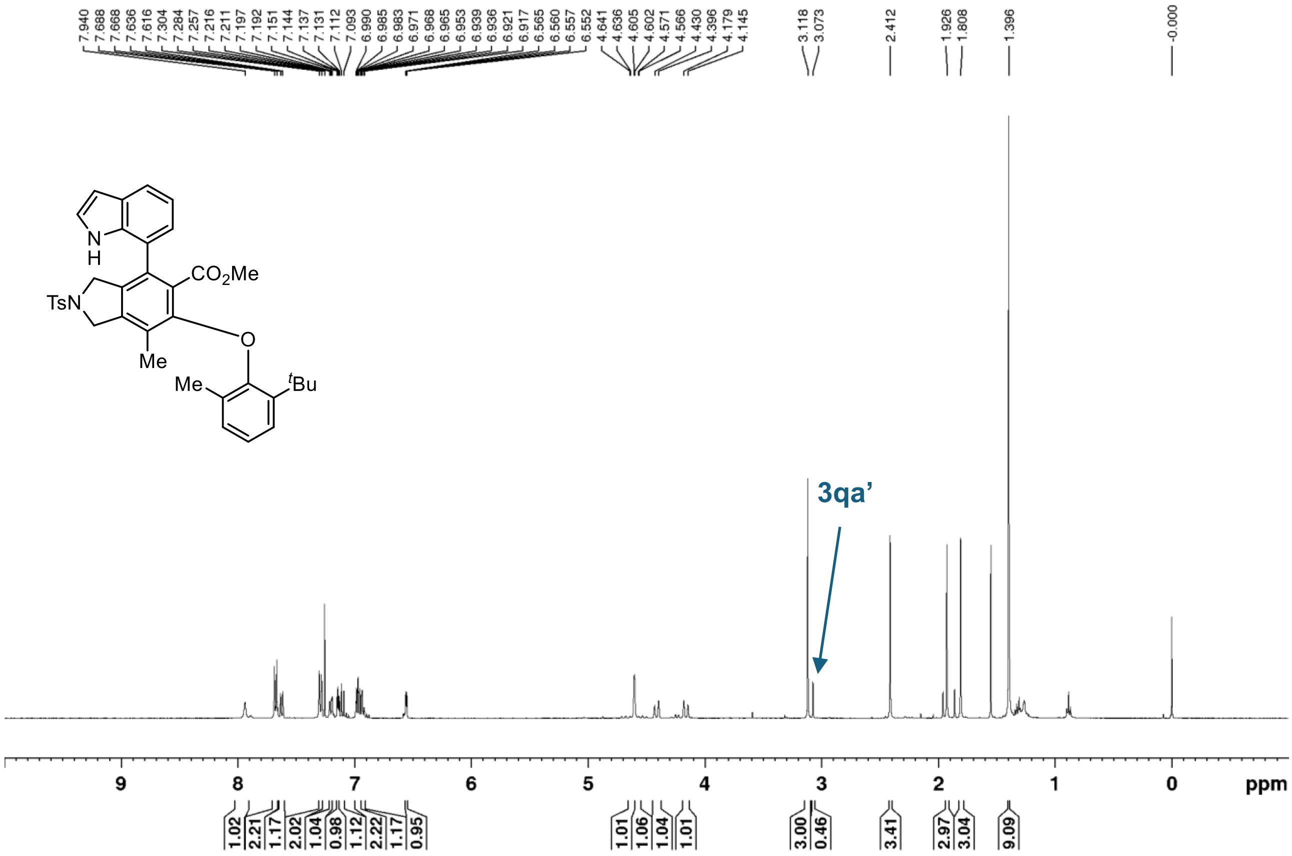

^{1}H NMR [**3qa’**] ($CDCl_3$, 400 MHz)

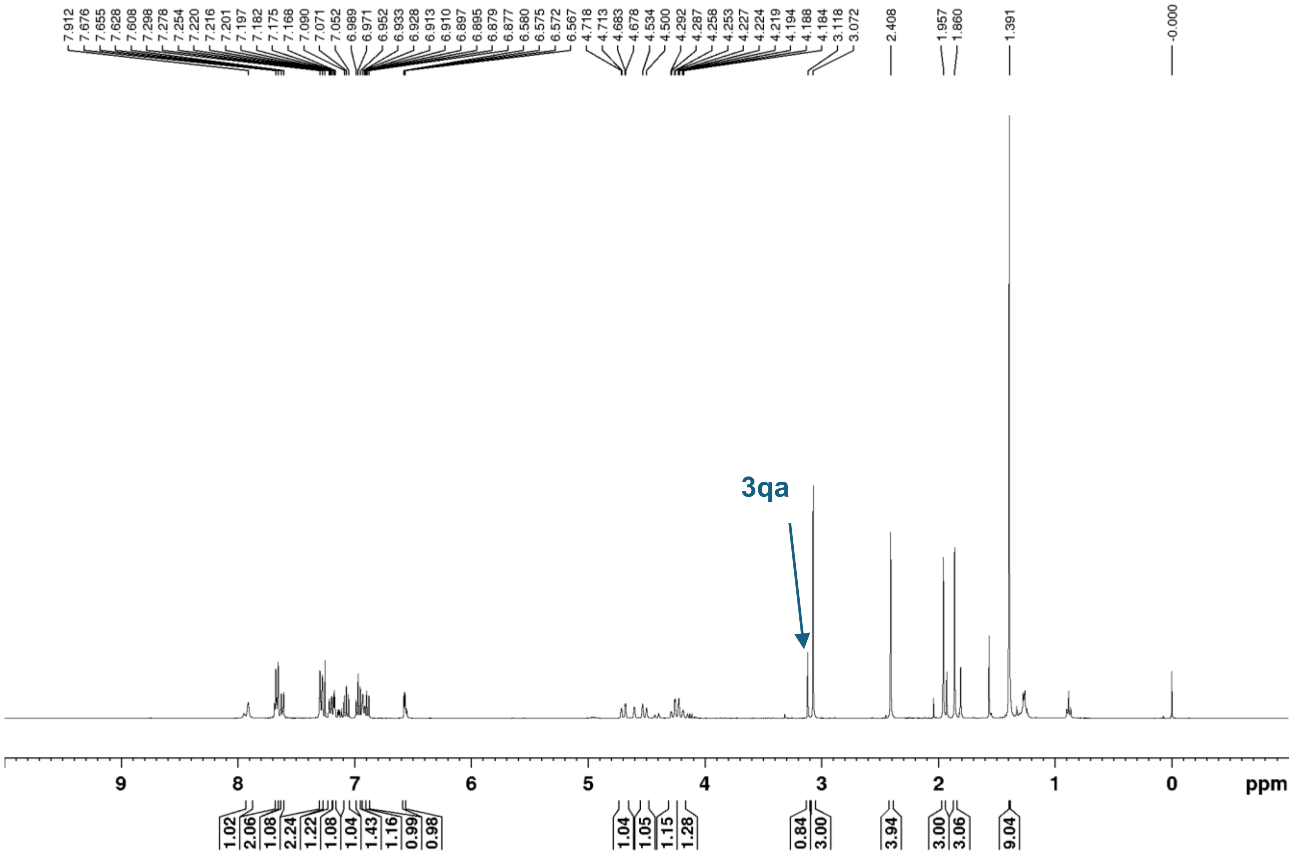

^{13}C NMR ($CDCl_3$, 101 MHz)

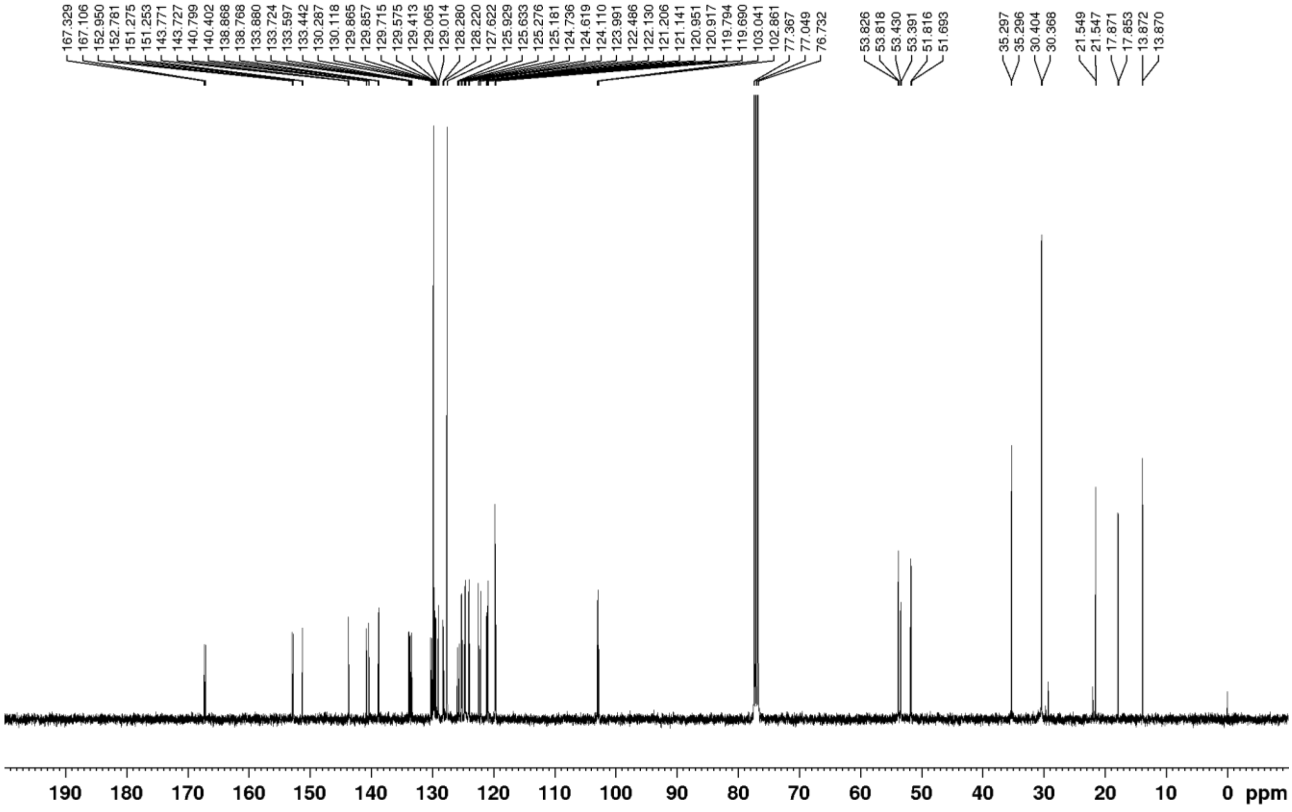

HMBC ($CDCl_3$, 400 MHz)

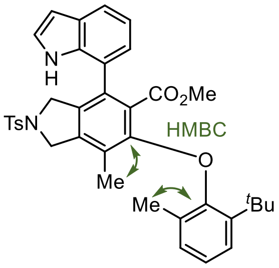

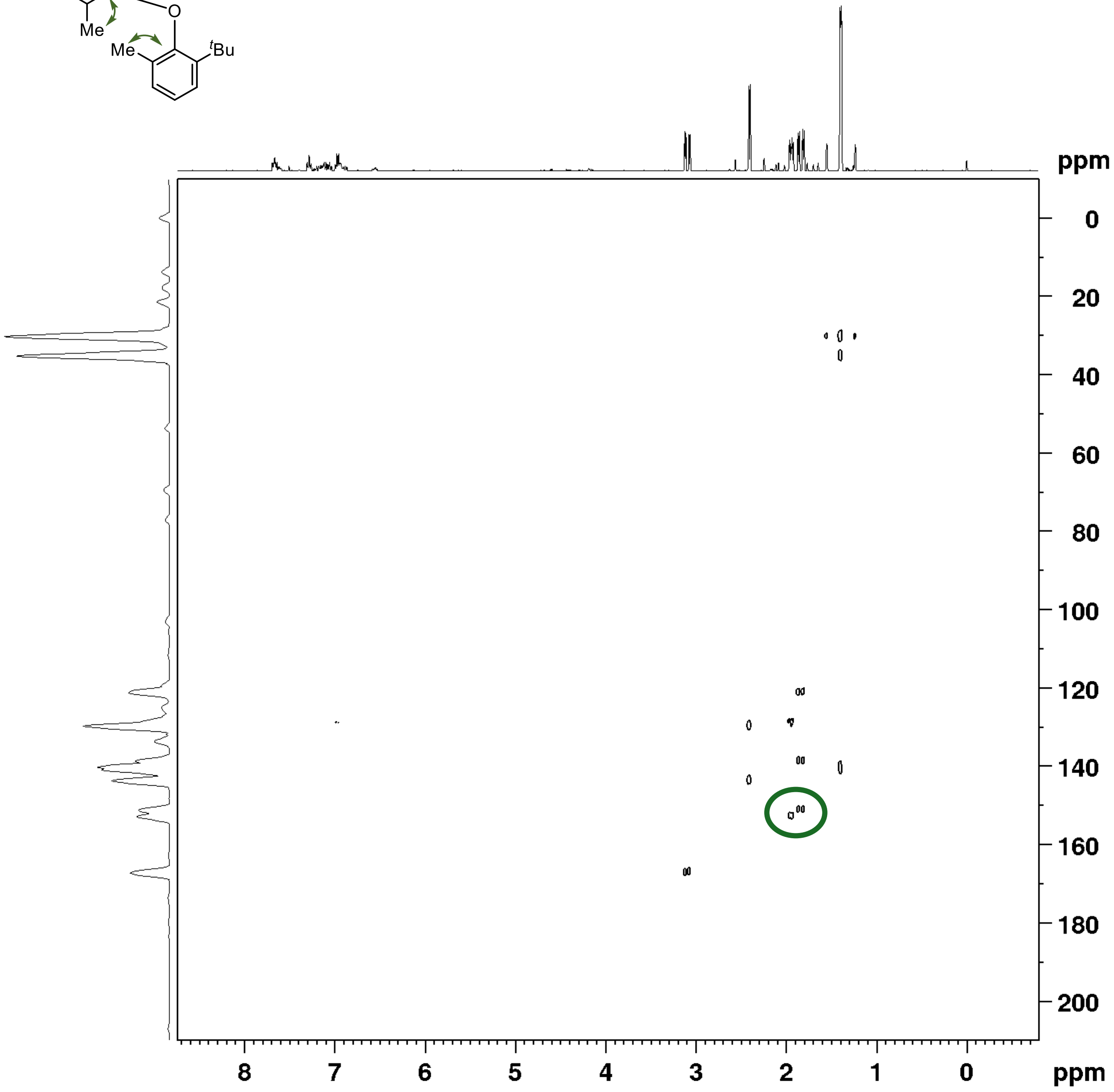

(–)-Methyl 7-([1,1'-biphenyl]-2-yl)-6-(2-(*tert*-butyl)-6-methylphenoxy)-4-methyl-2-tosylisoindoline-5-carboxylate [(–)-3ra]

^{1}H NMR (CDCl$_3$, 400 MHz)

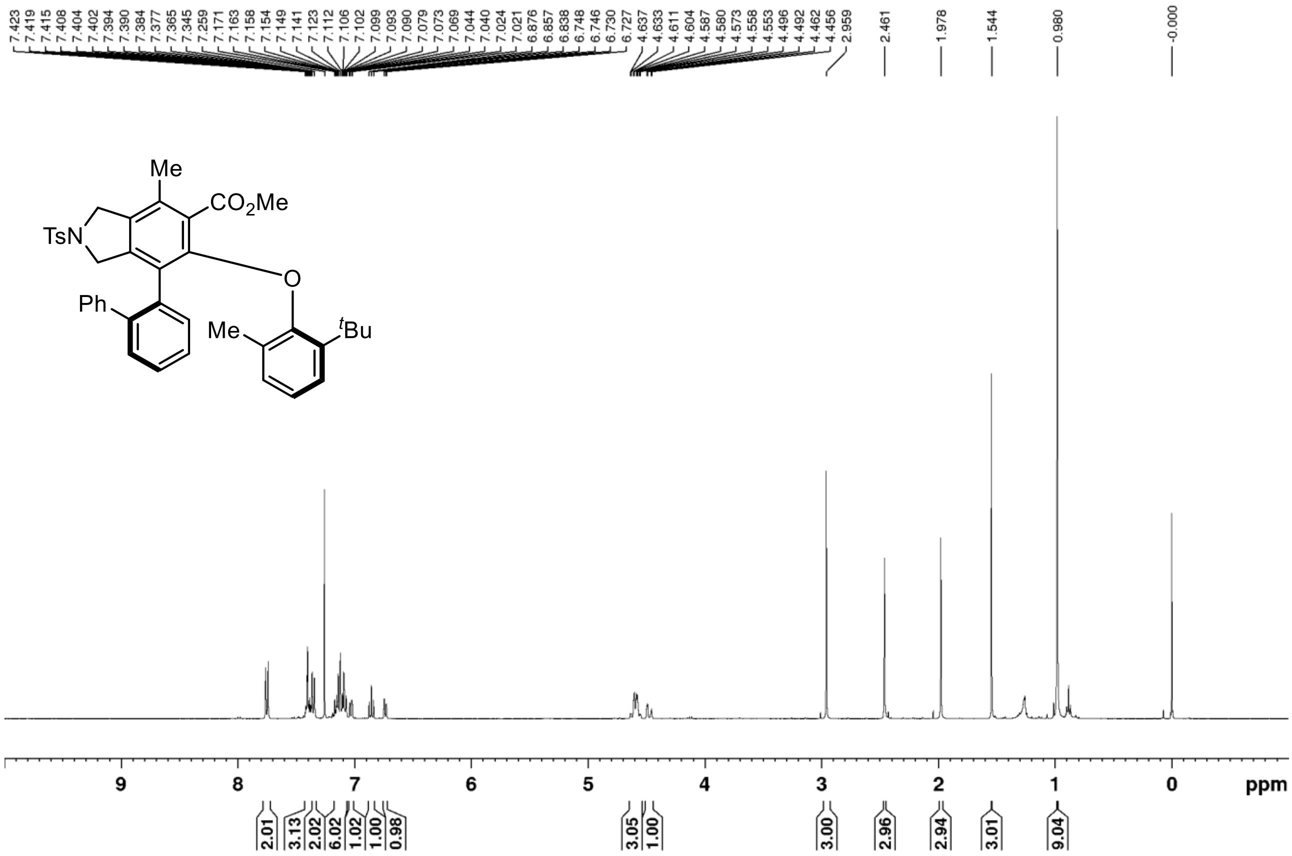

^{13}C NMR (CDCl$_3$, 101 MHz)

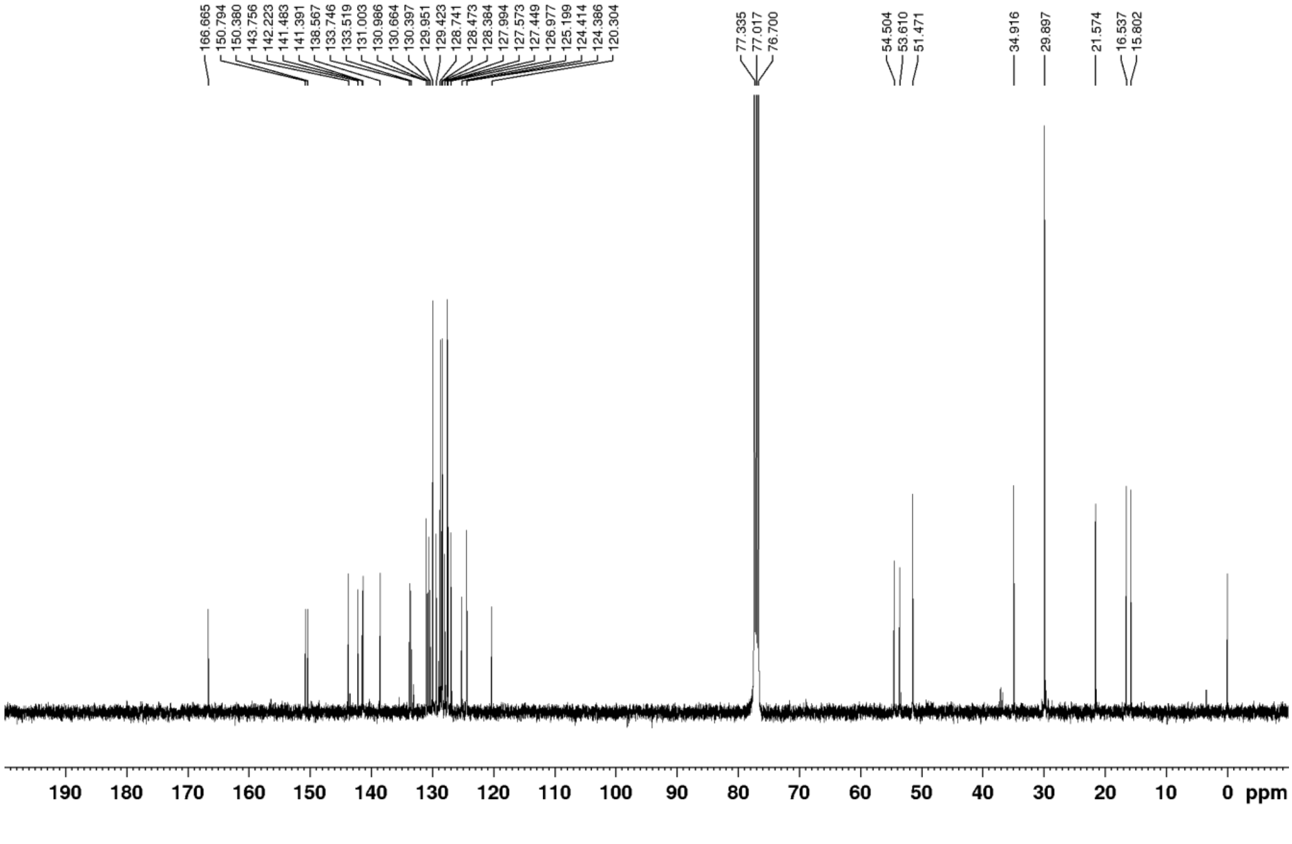

NOESY ($CDCl_3$, 400 MHz)

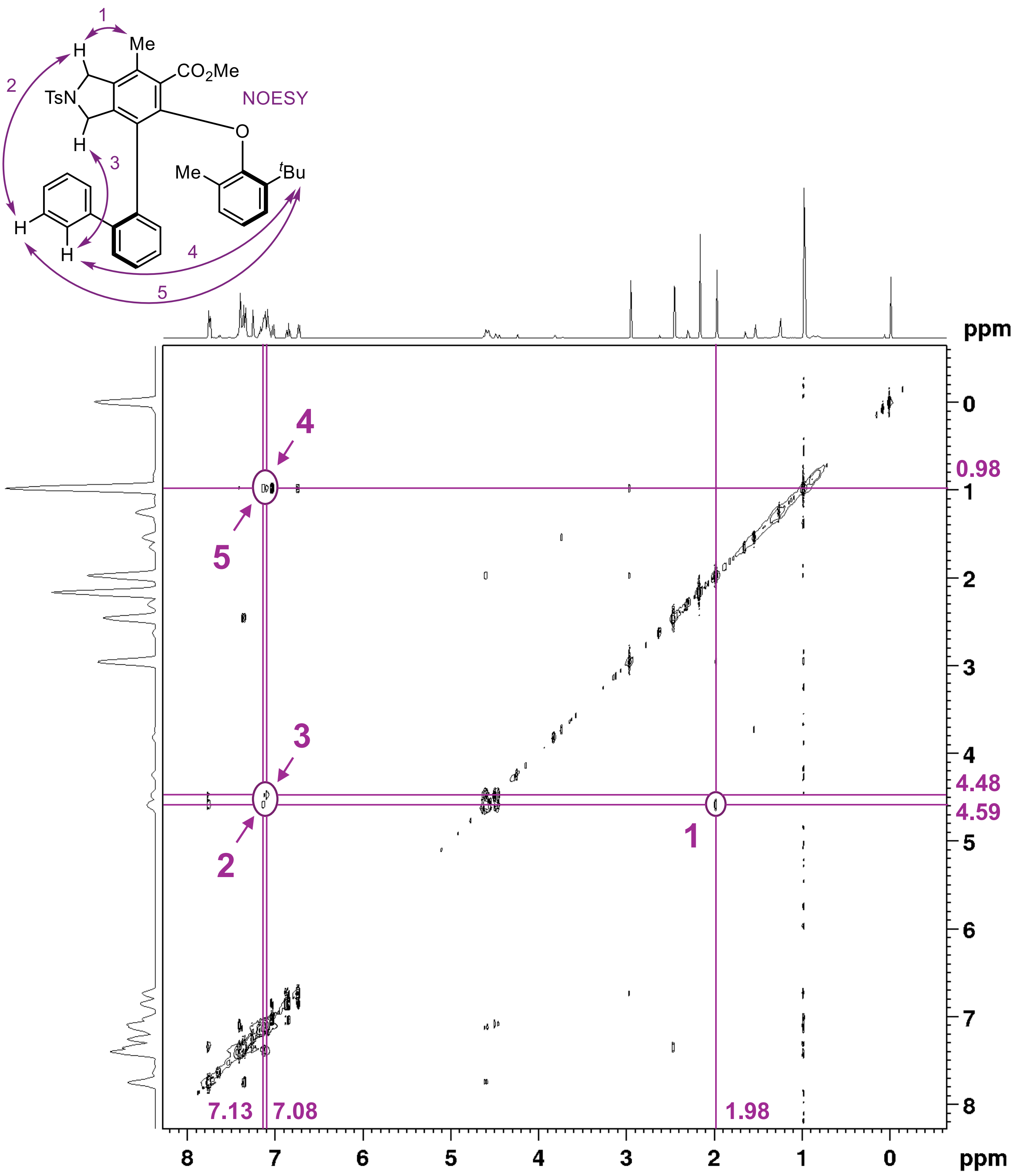

(–)-Methyl 7-(2-aminophenyl)-6-(2-(*tert*-butyl)-6-methylphenoxy)-4-methyl-2-tosylisoindoline-5-carboxylate [(–)-3sa]

^{1}H NMR ($CDCl_3$, 400 MHz)

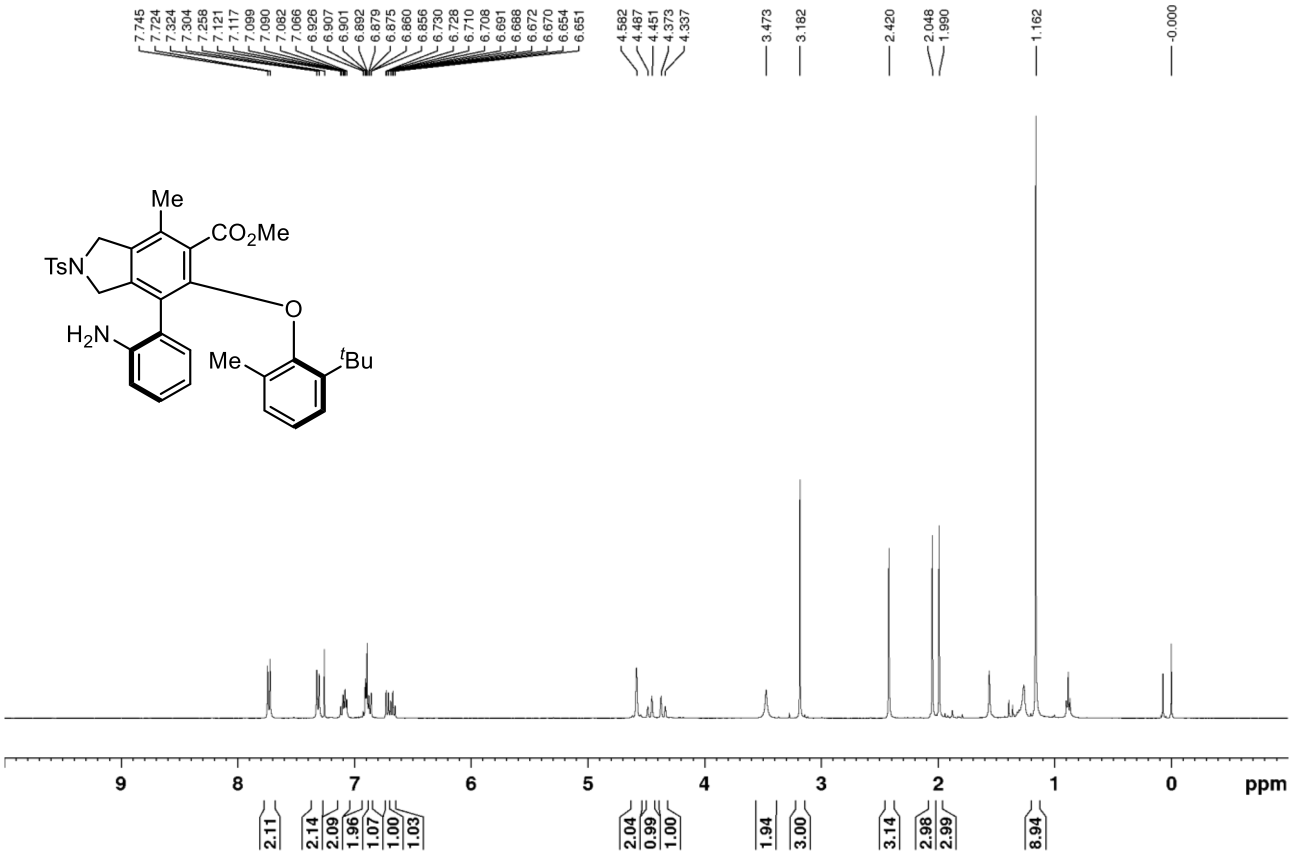

^{13}C NMR ($CDCl_3$, 101 MHz)

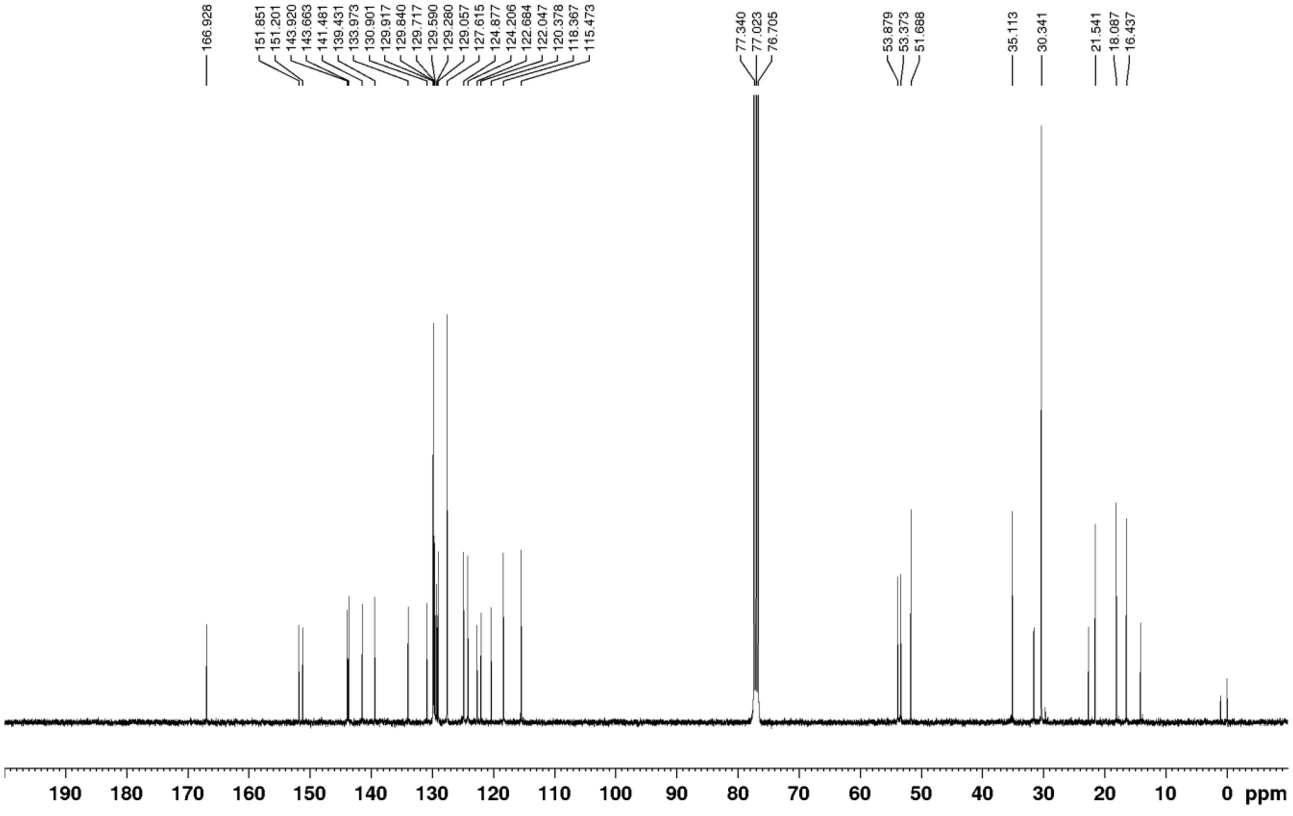

NOESY ($CDCl_3$, 400 MHz)

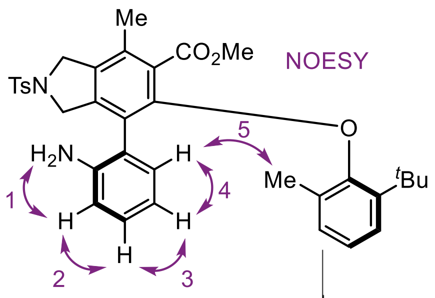

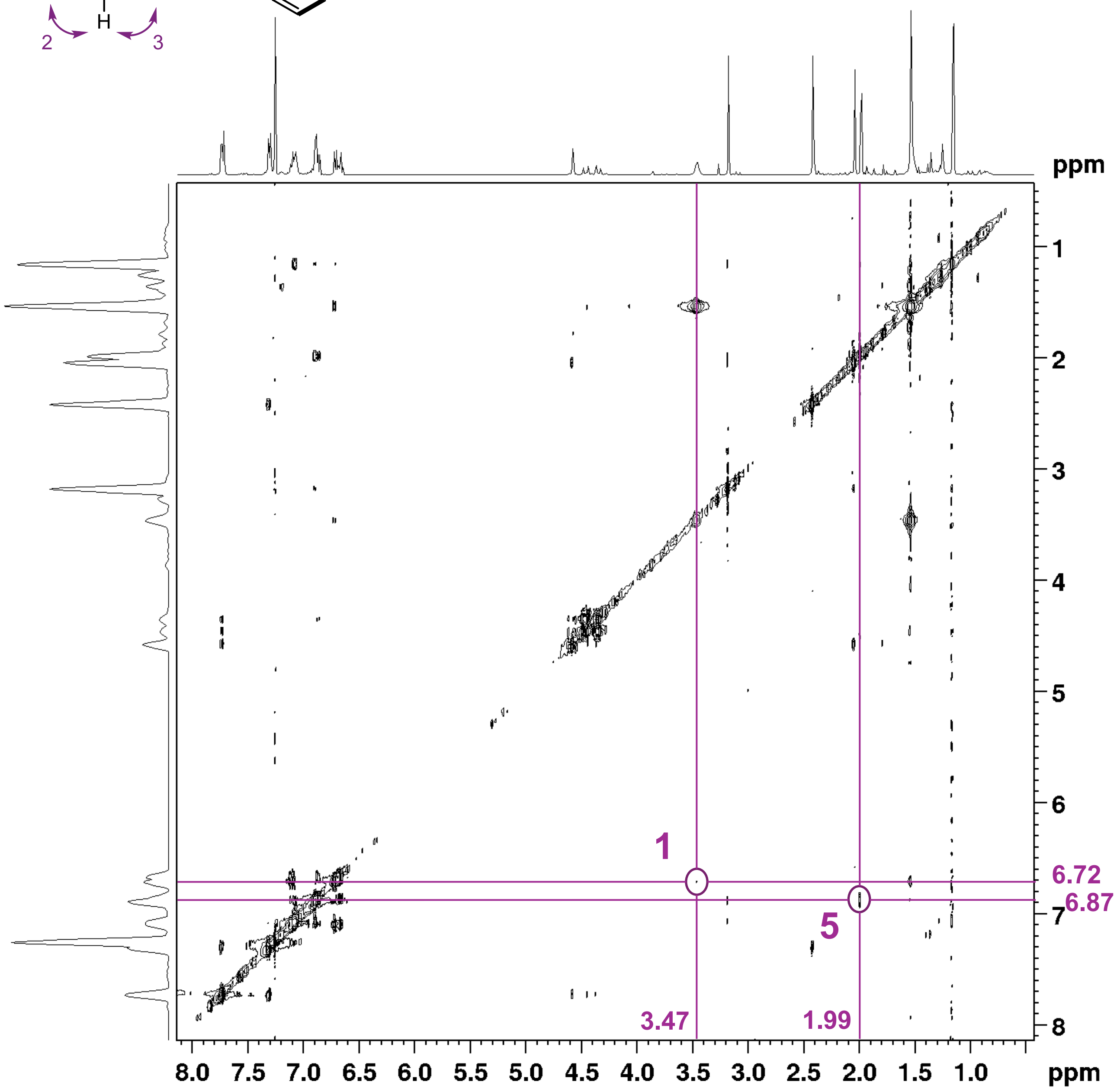

NOESY ($CDCl_3$, 400 MHz)

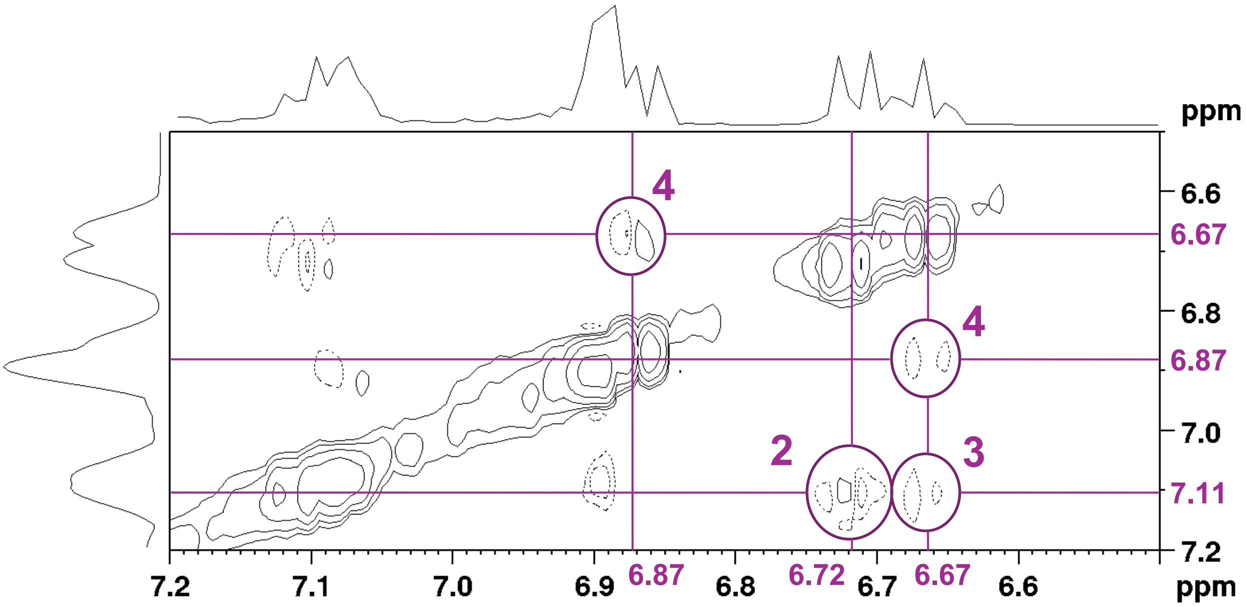

(–)-Methyl 6-(2-((3*r*,5*r*,7*r*)-adamantan-1-yl)-4,6-dimethylphenoxy)-7-(2-aminophenyl)-4-methyl-2-tosylisoindoline-5-carboxylate [(–)-3si]

^{1}H NMR ($CDCl_3$, 400 MHz)

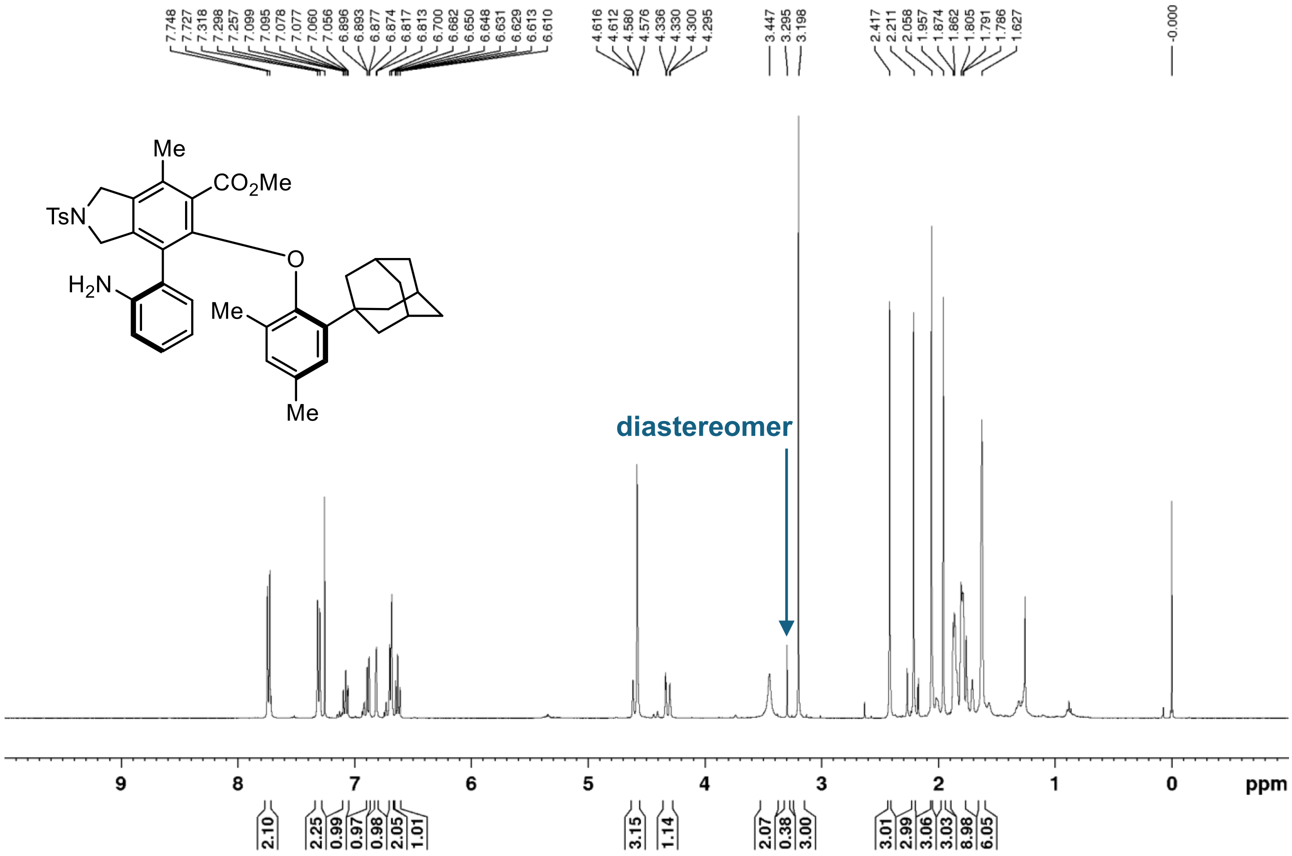

^{13}C NMR ($CDCl_3$, 101 MHz)

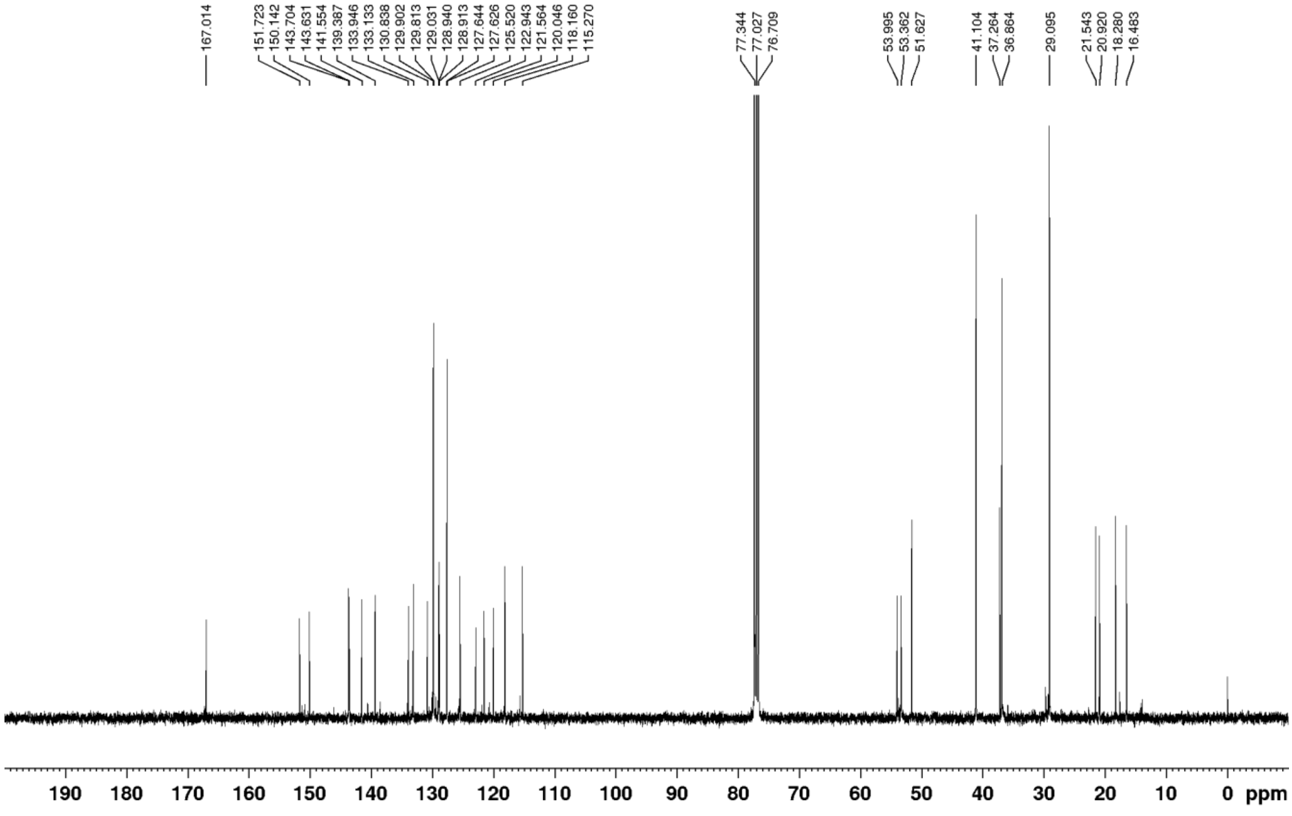

NOESY ($CDCl_3$, 400 MHz)

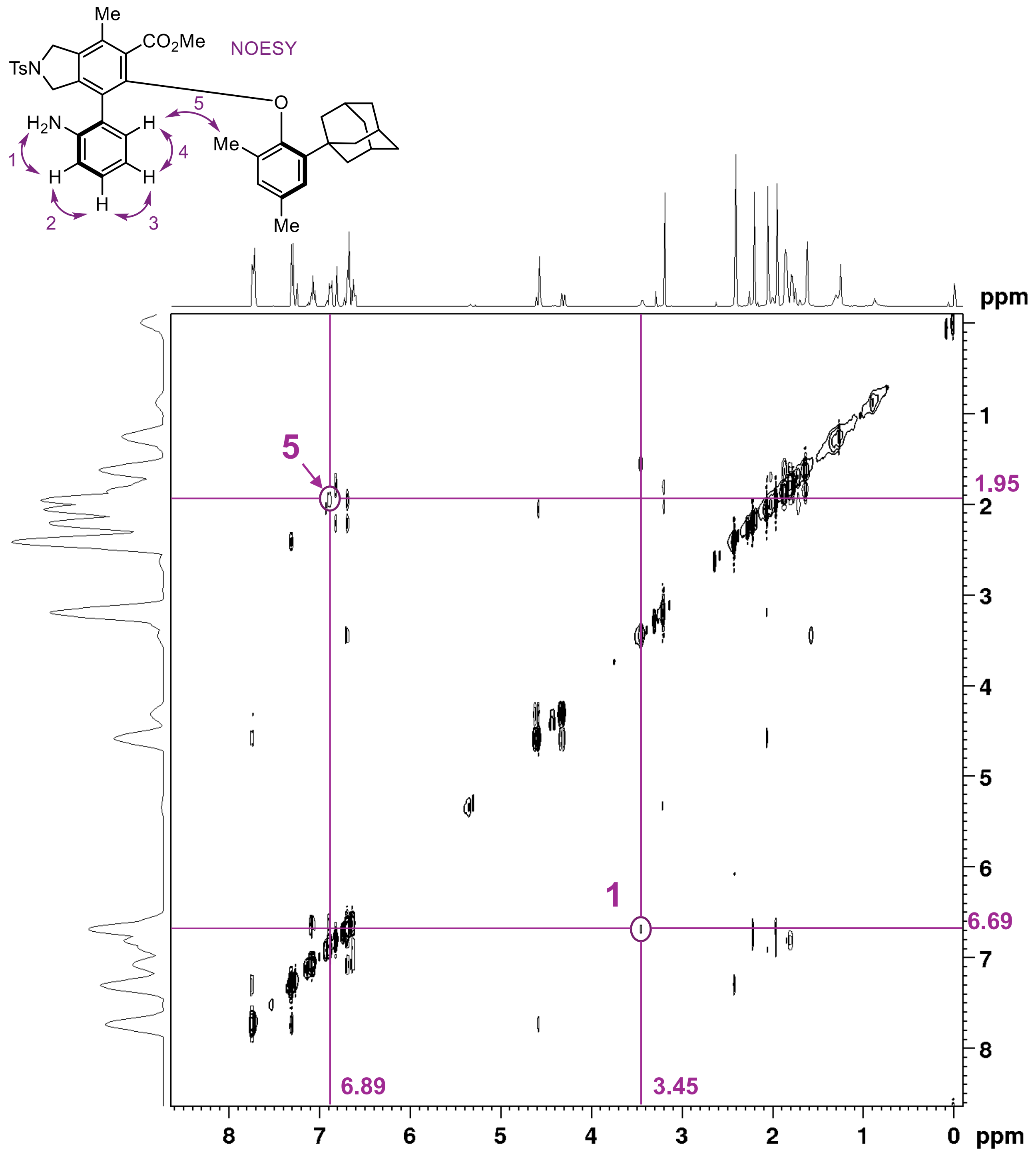

NOESY ($CDCl_3$, 400 MHz)

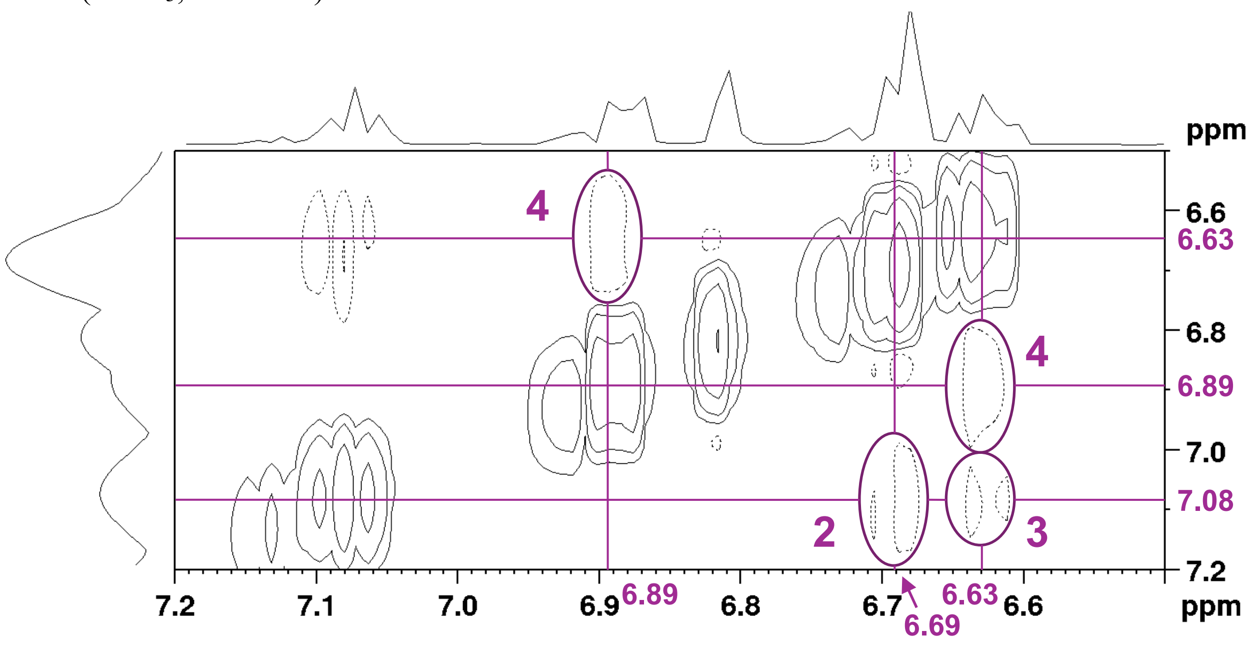

Methyl 6-(2-(*tert*-butyl)-6-methylphenoxy)-7-(2-hydroxyphenyl)-4-methyl-2-tosylisoindoline-5-carboxylate and (3ta) and methyl 6-(2-(*tert*-butyl)-6-methylphenoxy)-4-(2-hydroxyphenyl)-7-methyl-2-tosylisoindoline-5-carboxylate (4ta)

^{1}H NMR [**3ra**] ($CDCl_3$, 400 MHz)

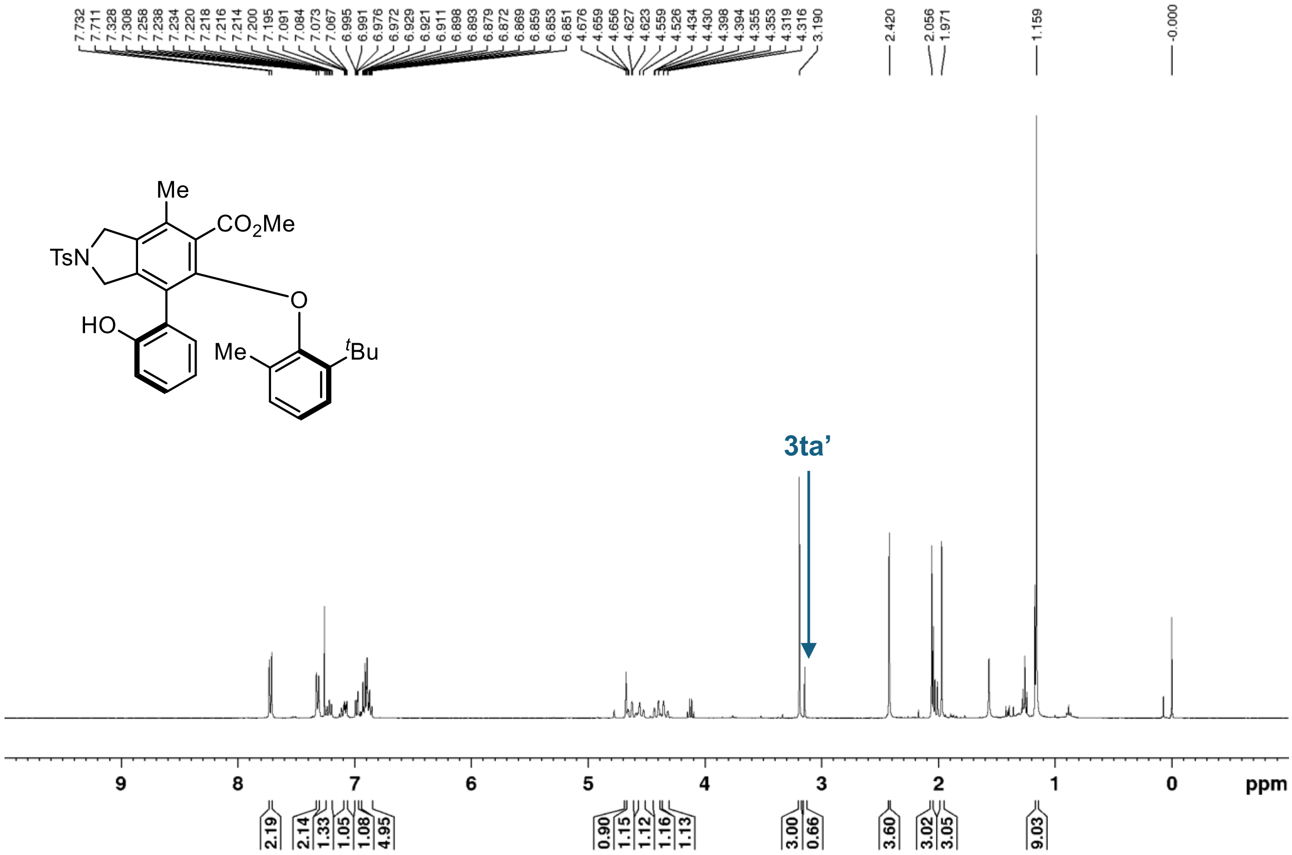

^{1}H NMR [**3ra**] ($CDCl_3$, 400 MHz)

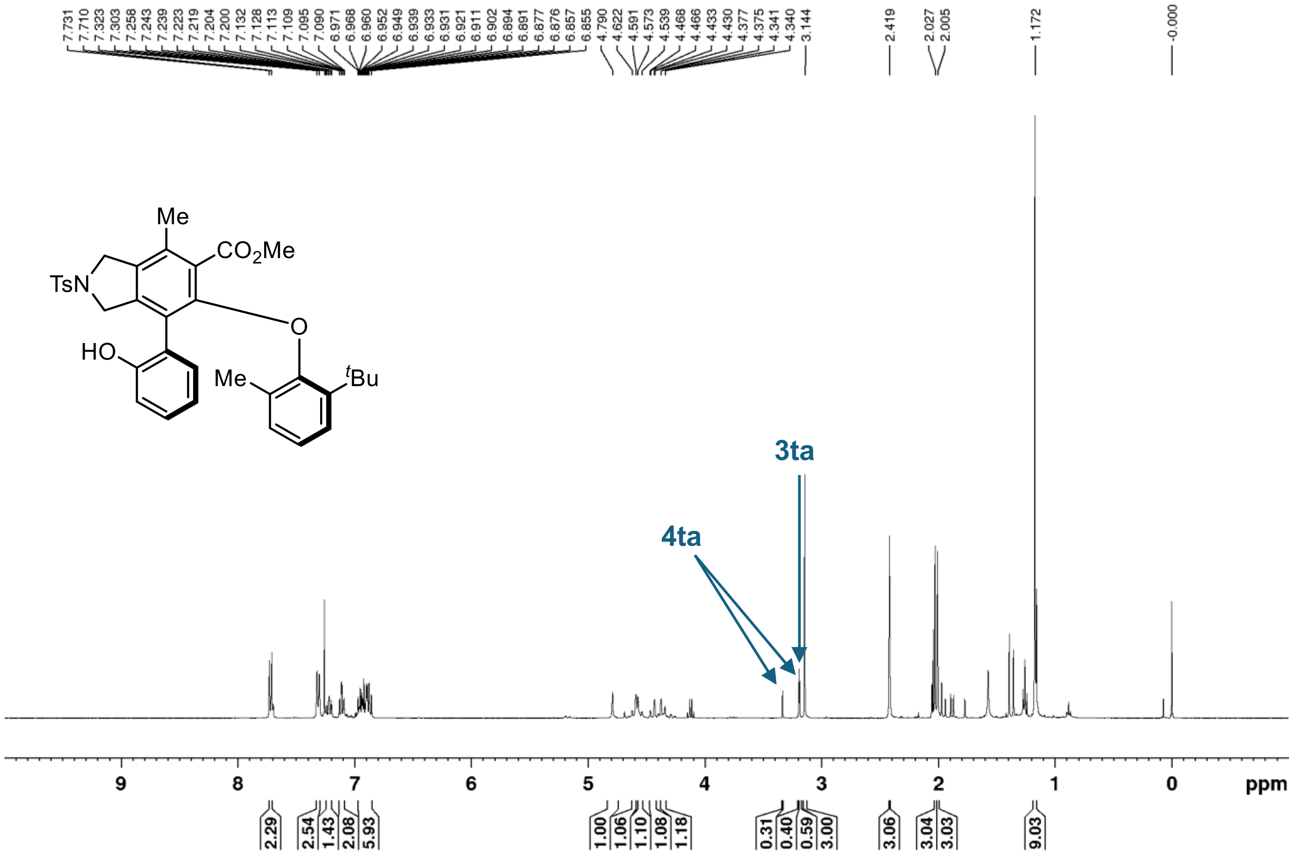

^{13}C NMR ($CDCl_3$, 101 MHz)

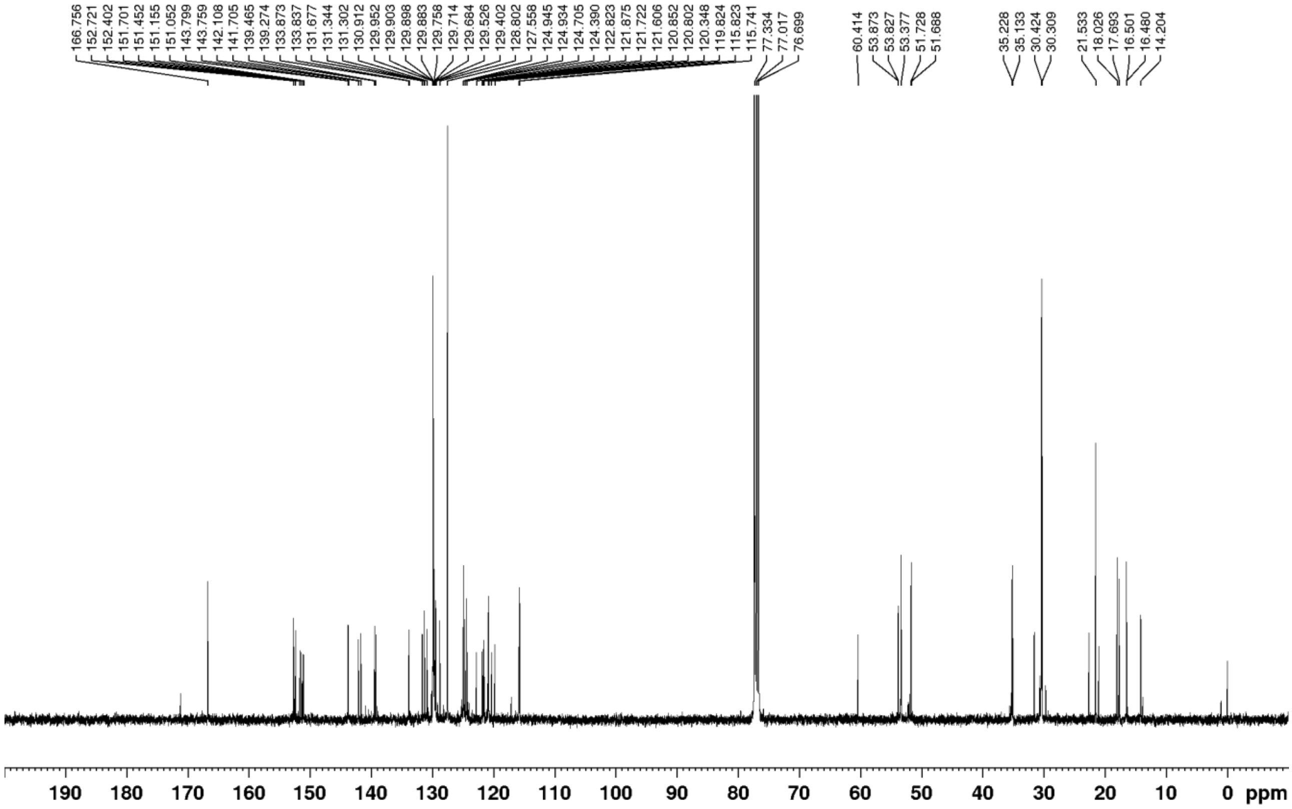

NOESY (CDCl3, 400 MHz)

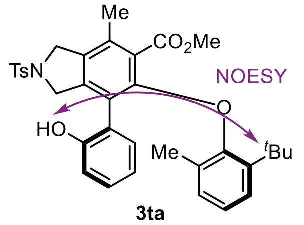

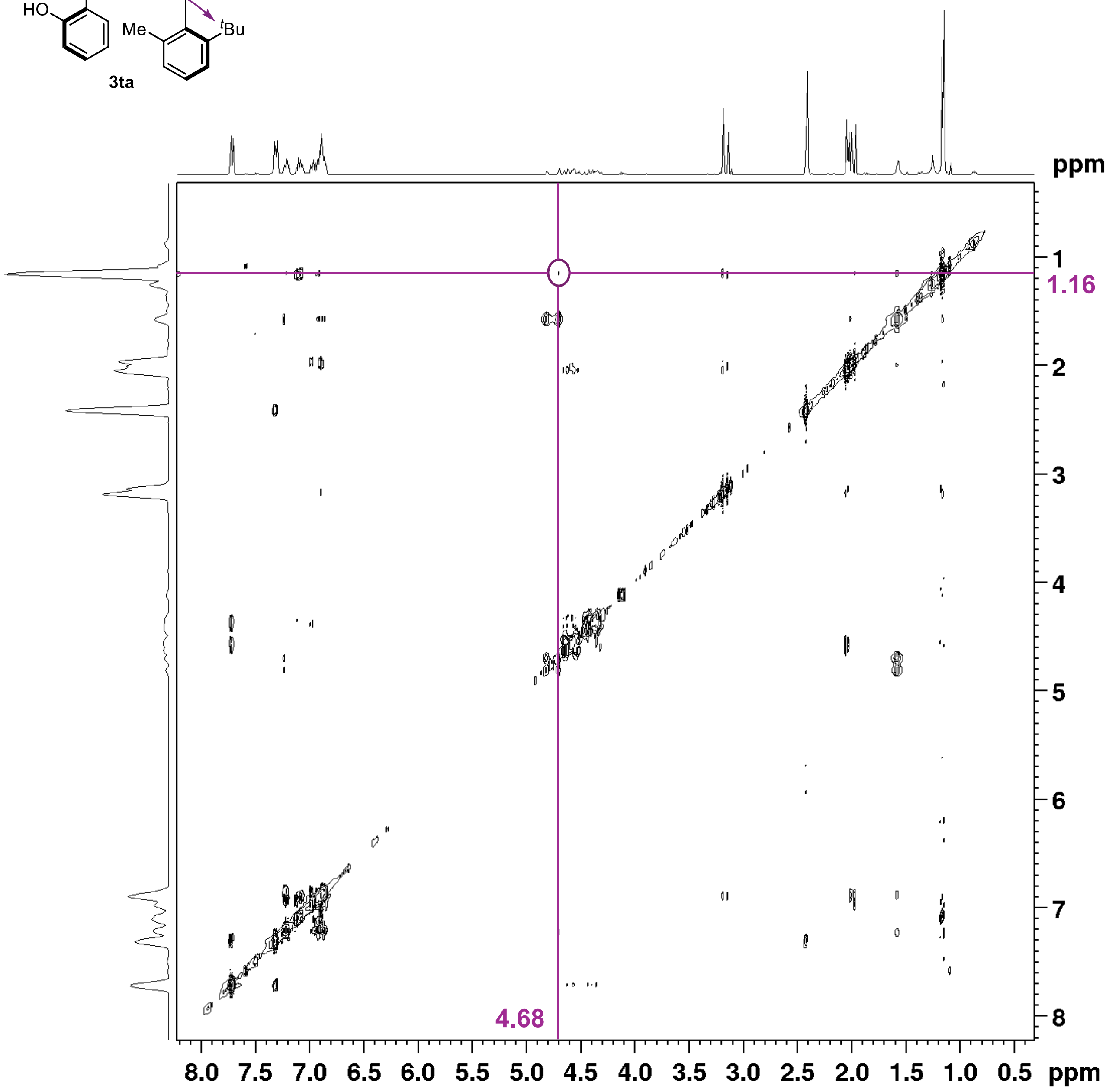

(*R*,*R*)-(–)-Methyl 5-(2-(*tert*-butyl)-6-methoxy-4-methylphenoxy)-6-(dimethylcarbamoyl)-7-methyl-2-tosylisoindoline-4-carboxylate [(*R*,*R*)-(–)-3am]

^{1}H NMR ($CDCl_3$, 400 MHz)

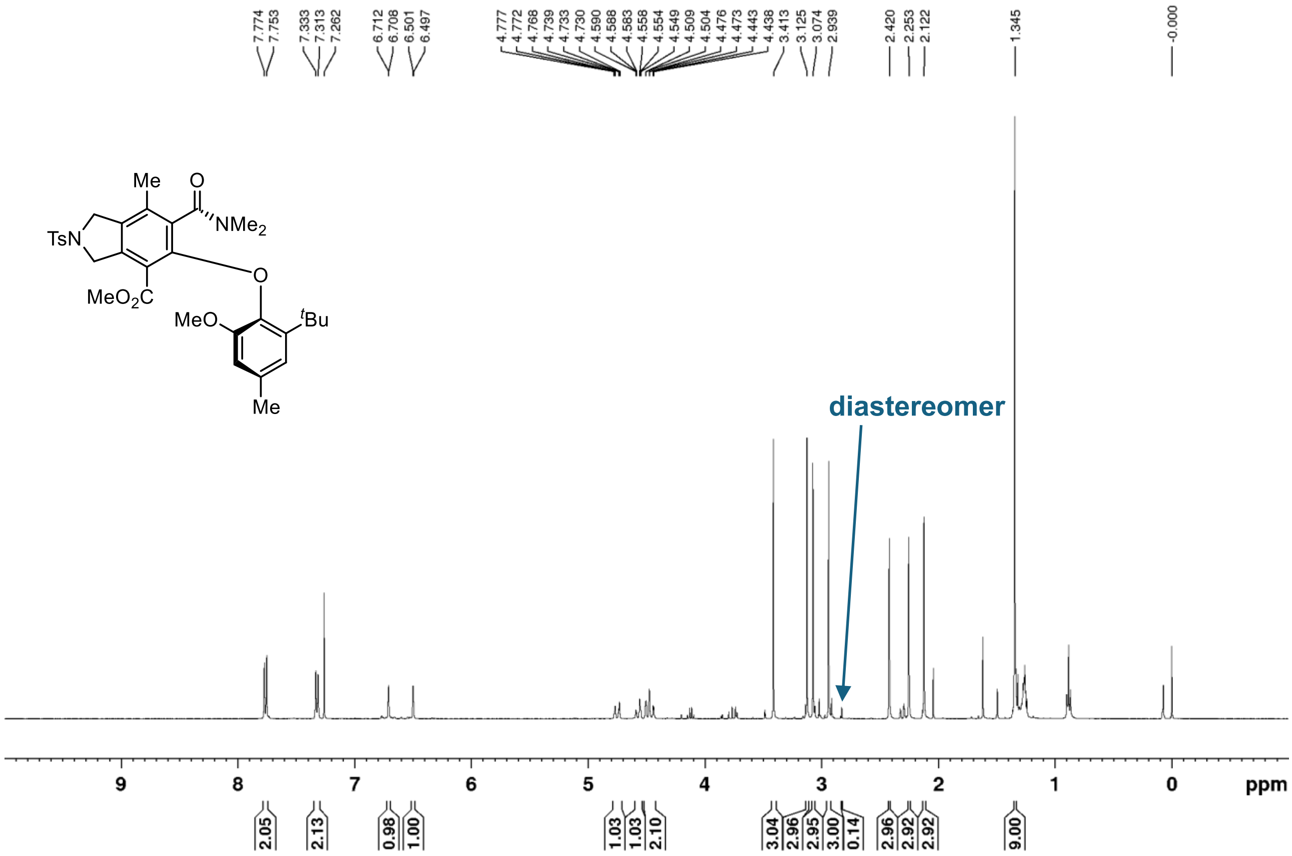

^{13}C NMR ($CDCl_3$, 101 MHz)

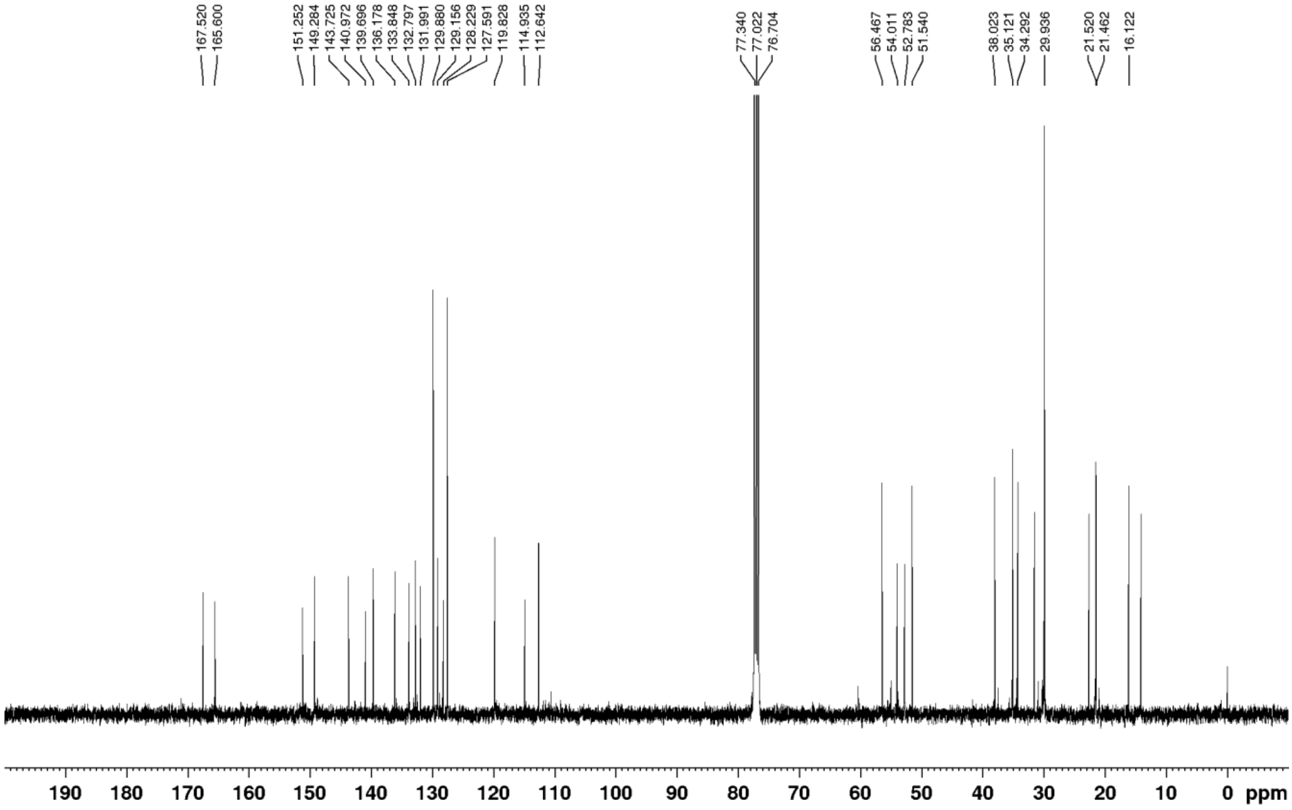

NOESY (CDCl3, 400 MHz)

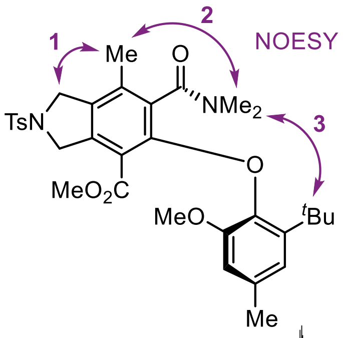

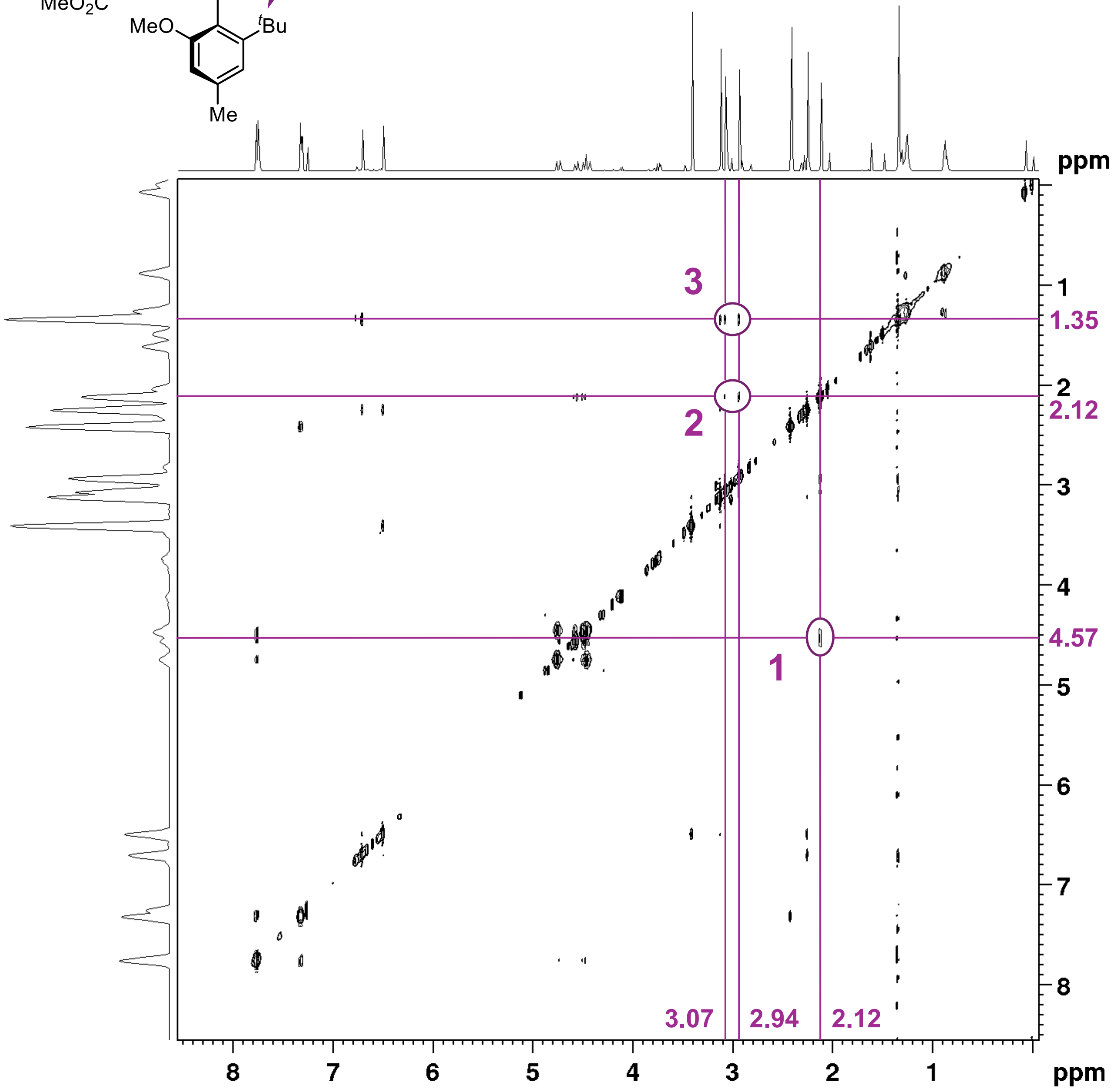

(*R,S*)-(–)- (–)-Methyl 5-(2-(*tert*-butyl)-6-methoxy-4-methylphenoxy)-6-(dimethylcarbamoyl)-7-methyl-2-tosylisoindoline-4-carboxylate [(*R,S*)-(–)-3am']

^{1}H NMR ($CDCl_3$, 400 MHz)

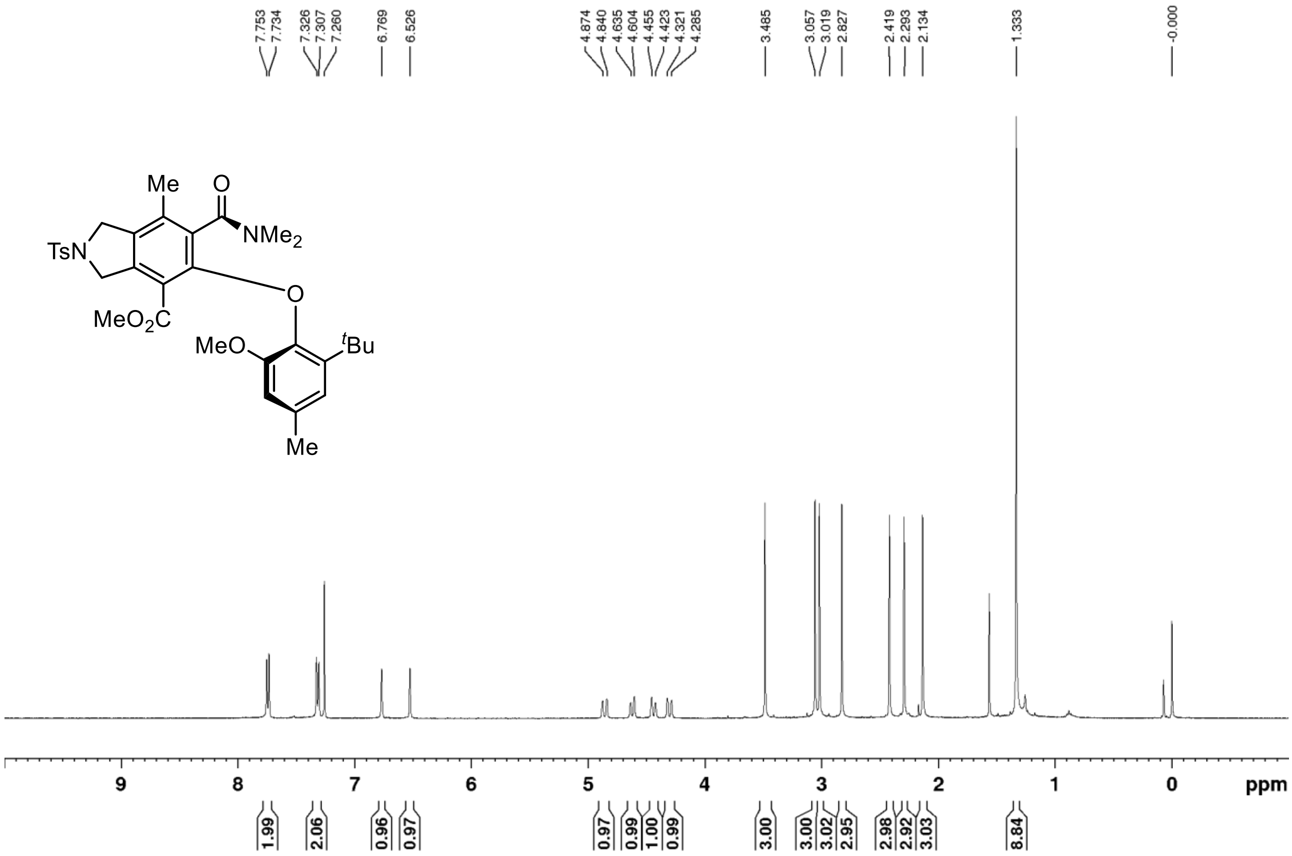

^{13}C NMR ($CDCl_3$, 101 MHz)

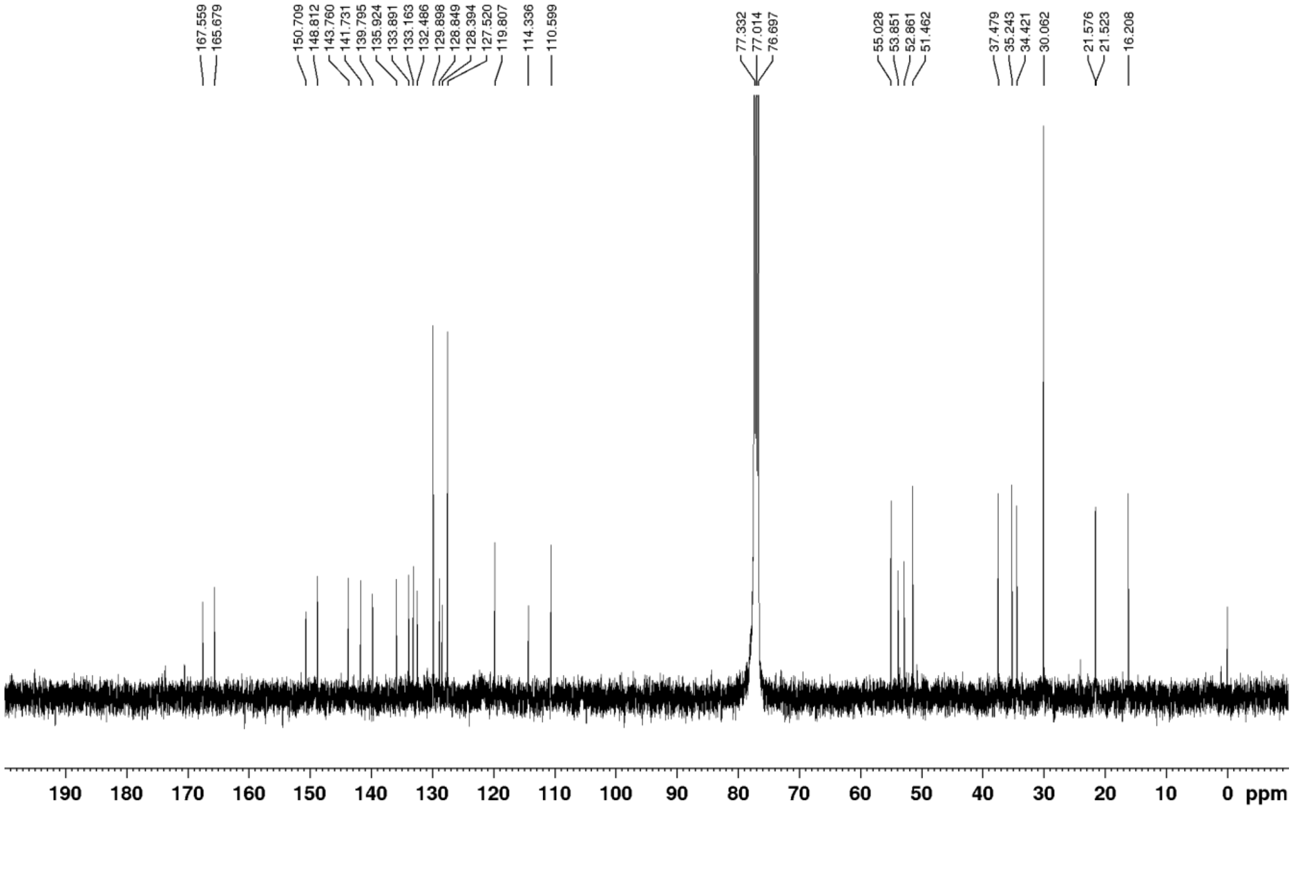

NOESY ($CDCl_3$, 400 MHz)

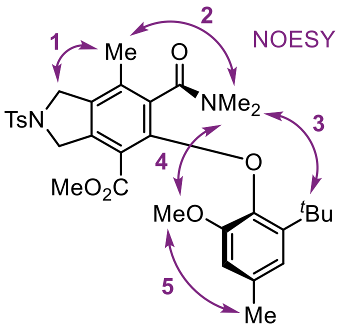

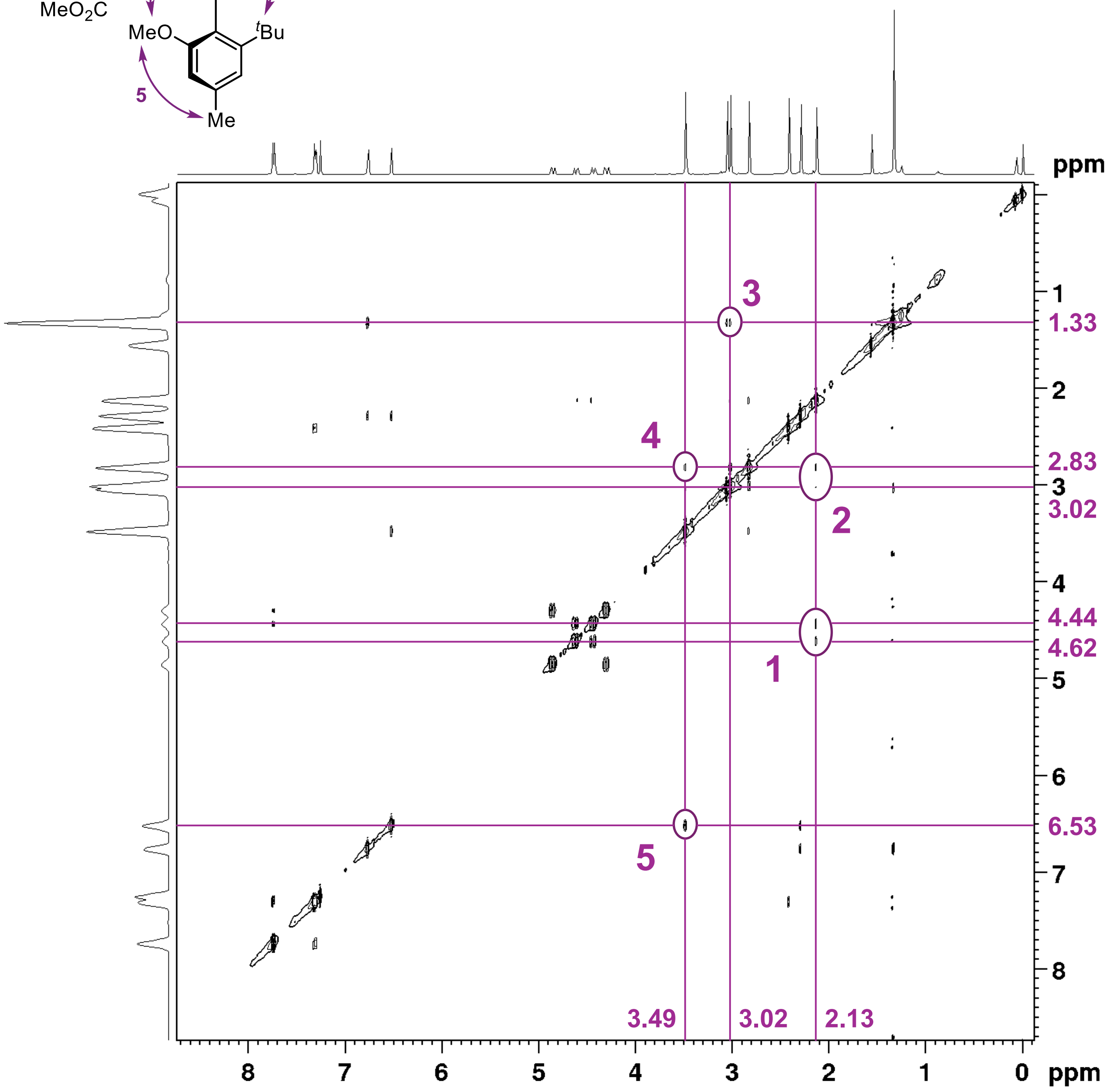

(+)-7-(2-Aminophenyl)-6-(2-(*tert*-butyl)-6-methylphenoxy)-*N*,*N*,4-trimethyl-2-tosylisoindoline-5-carboxamide [(+)-3sn]

^{1}H NMR ($CDCl_3$, 400 MHz)

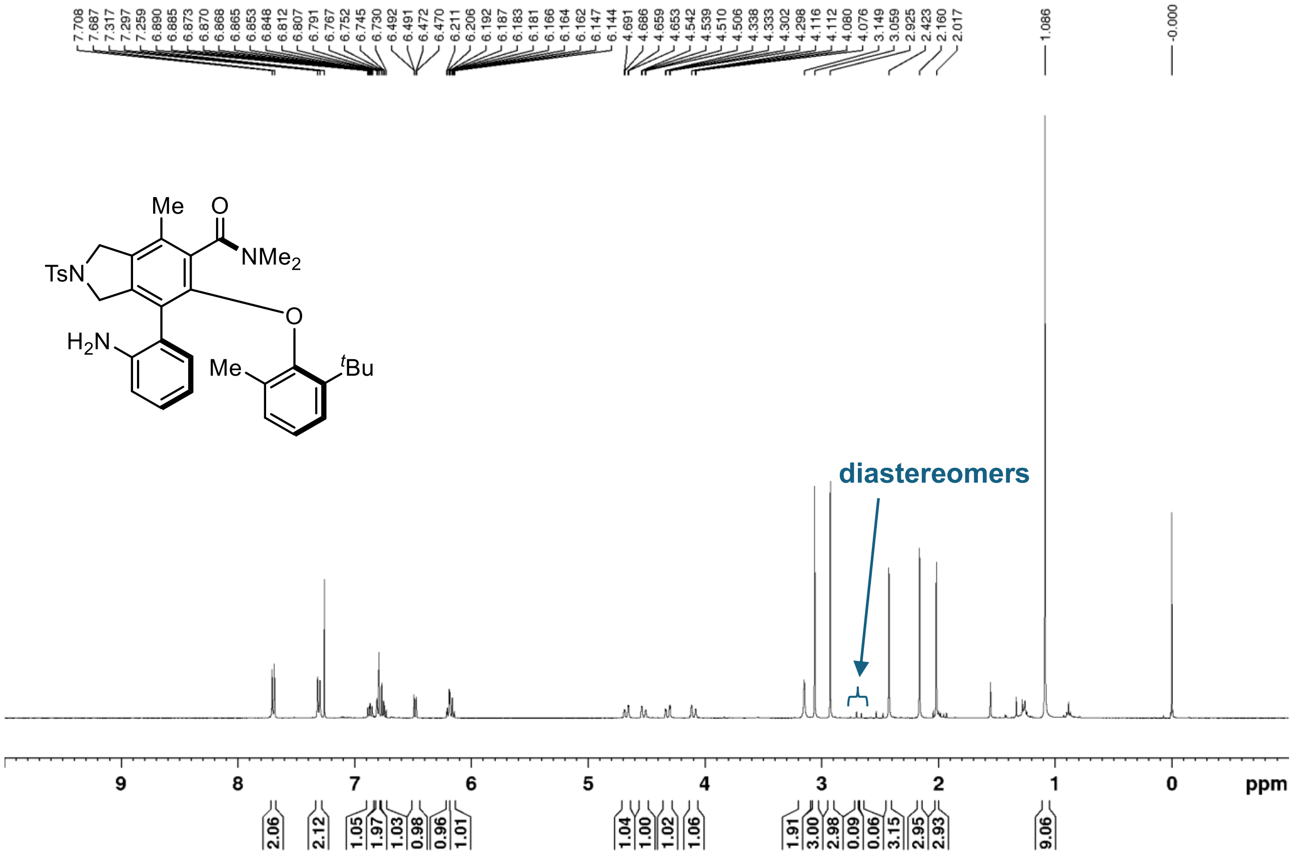

^{13}C NMR ($CDCl_3$, 101 MHz)

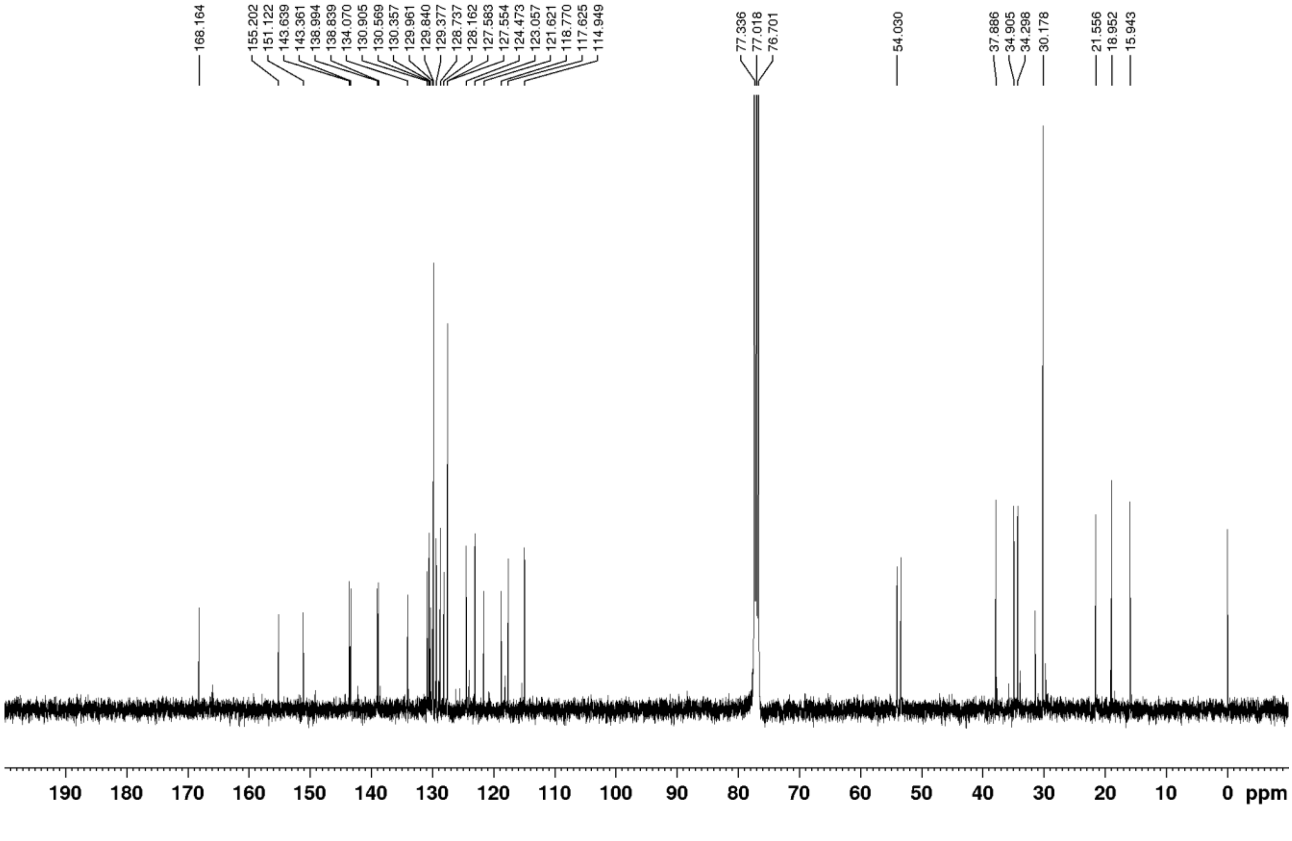

NOESY ($CDCl_3$, 400 MHz)

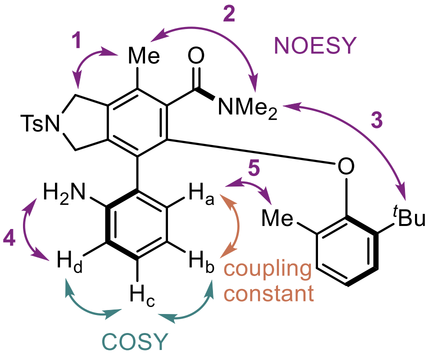

H_a 6.20 ppm (dd, J = 1.9, 7.7 Hz)

H_b 6.16 ppm (ddd, J = 1.0, 6.8, 7.7 Hz)

H_c 6.87 ppm (ddd, J = 2.0, 6.8, 8.0 Hz)

H_d 6.48 ppm (dd, J = 0.6, 8.0 Hz)

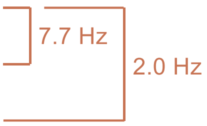

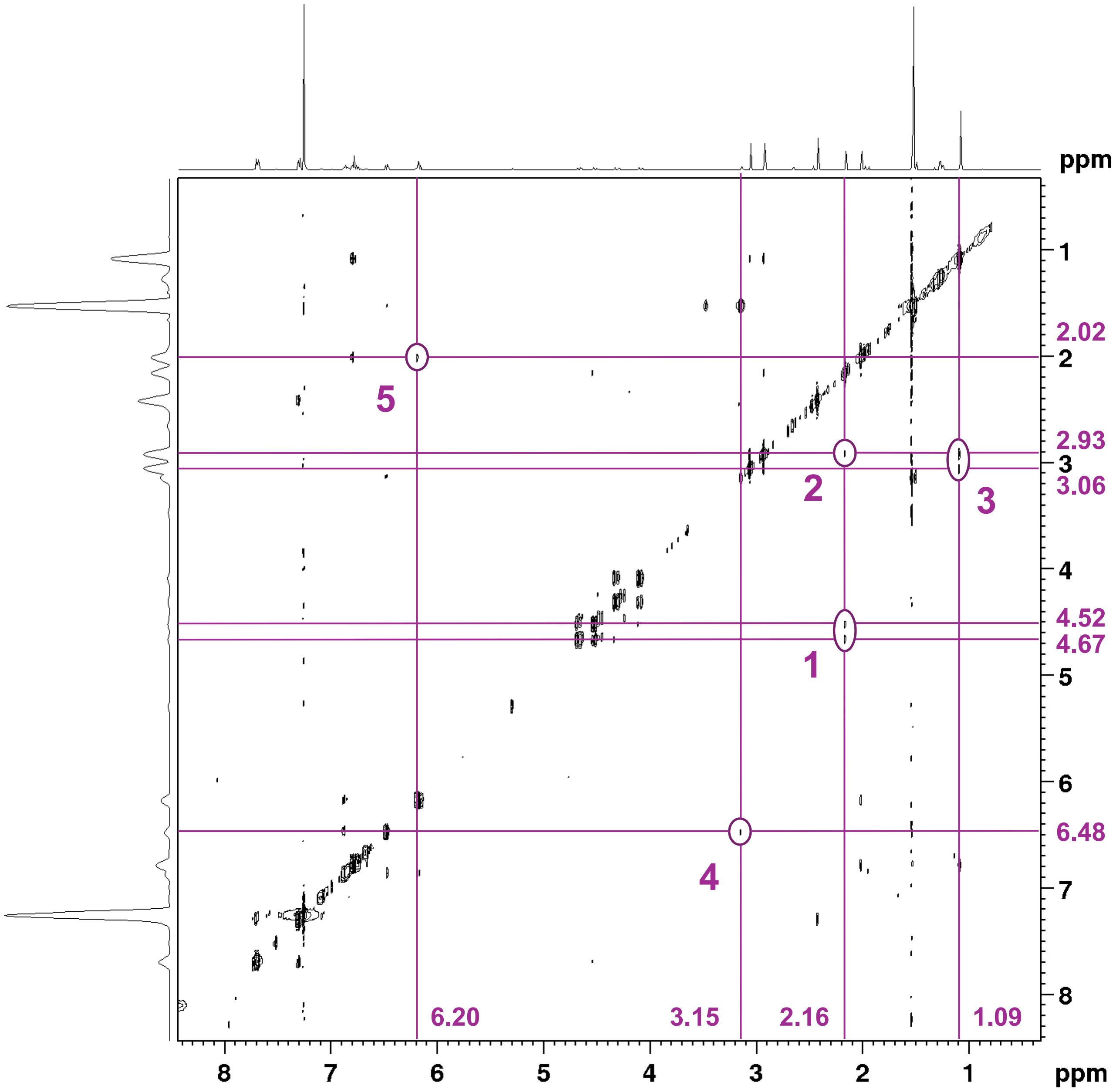

COSY ($CDCl_3$, 400 MHz)

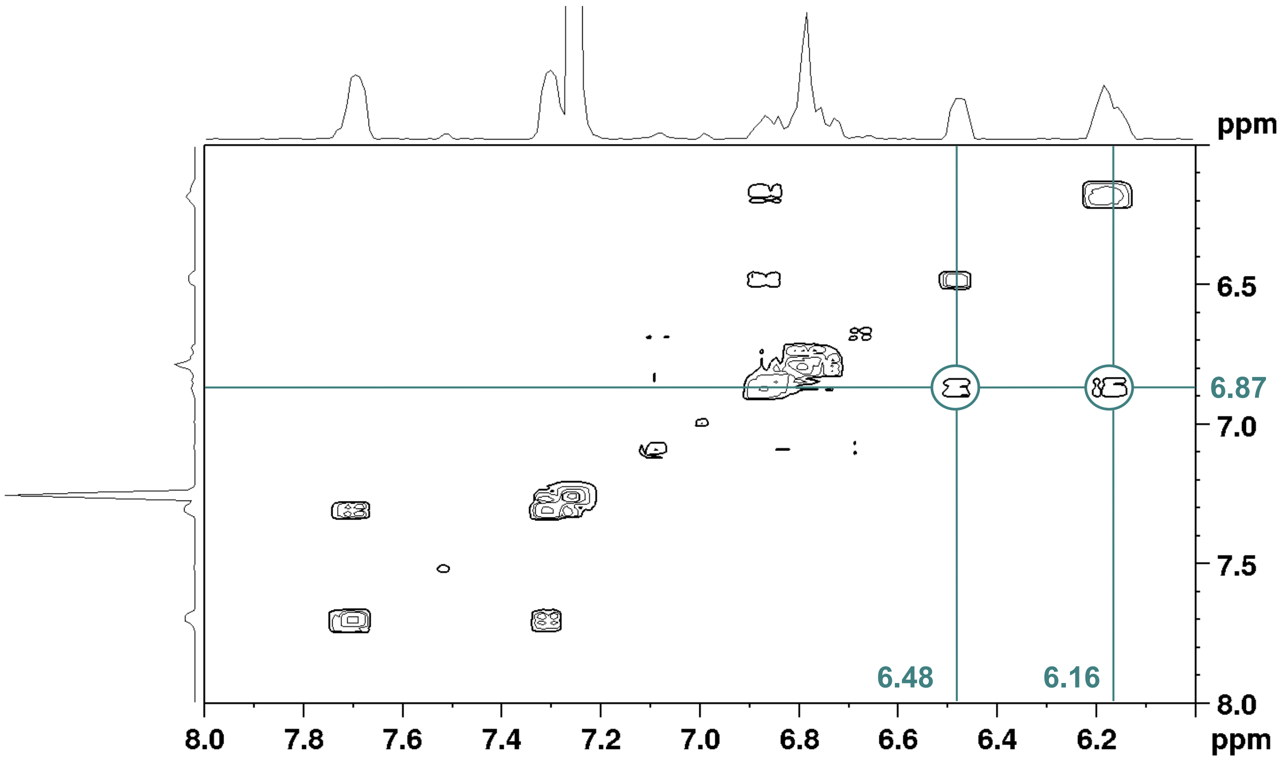

(+)-6-((3-(*tert*-Butyl)-4',5-dimethyl-[1,1'-biphenyl]-2-yl)oxy)-*N*,*N*,4-trimethyl-7-(naphthalen-1-yl)-2-tosylisoindoline-5-carboxamide [(+)-3no]

^{1}H NMR ($CDCl_3$, 400 MHz)

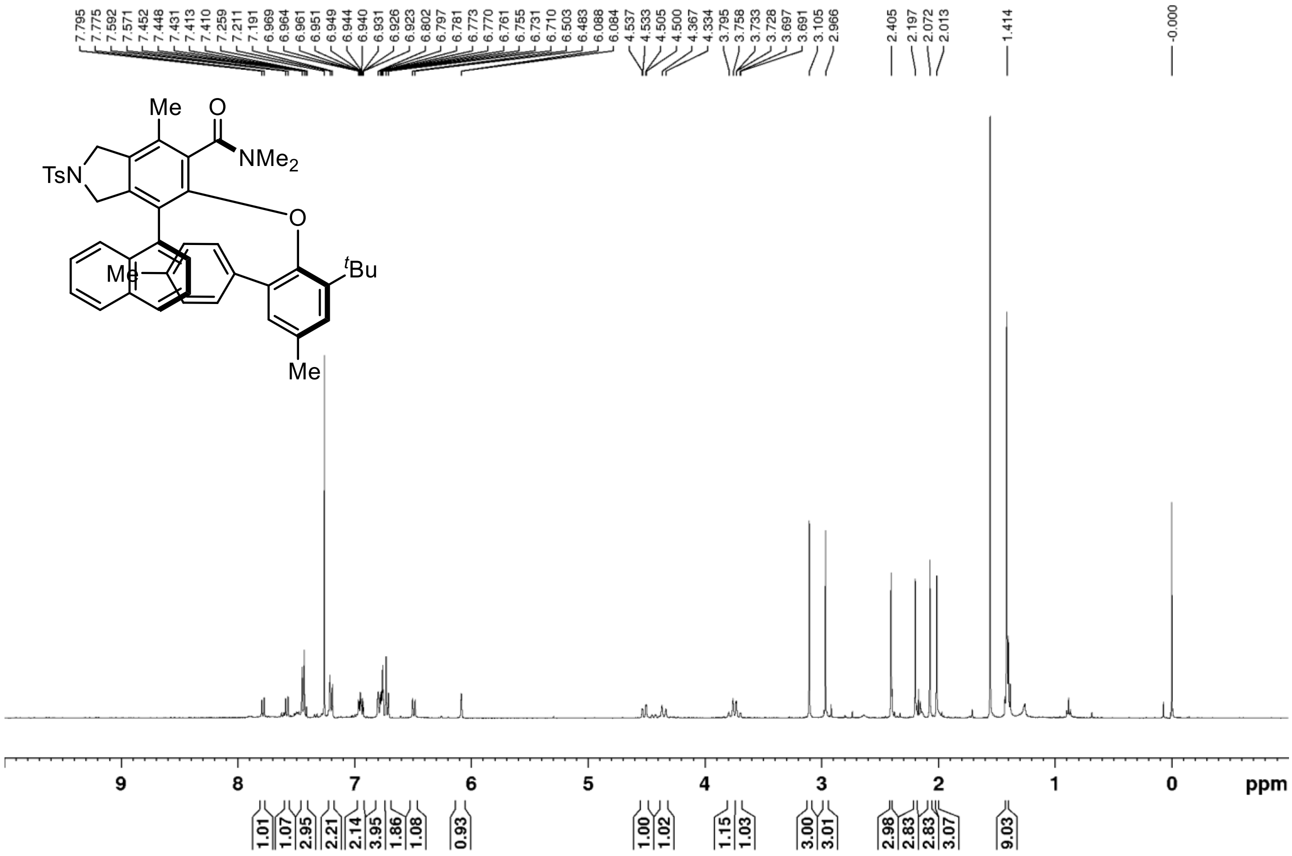

^{13}C NMR ($CDCl_3$, 101 MHz)

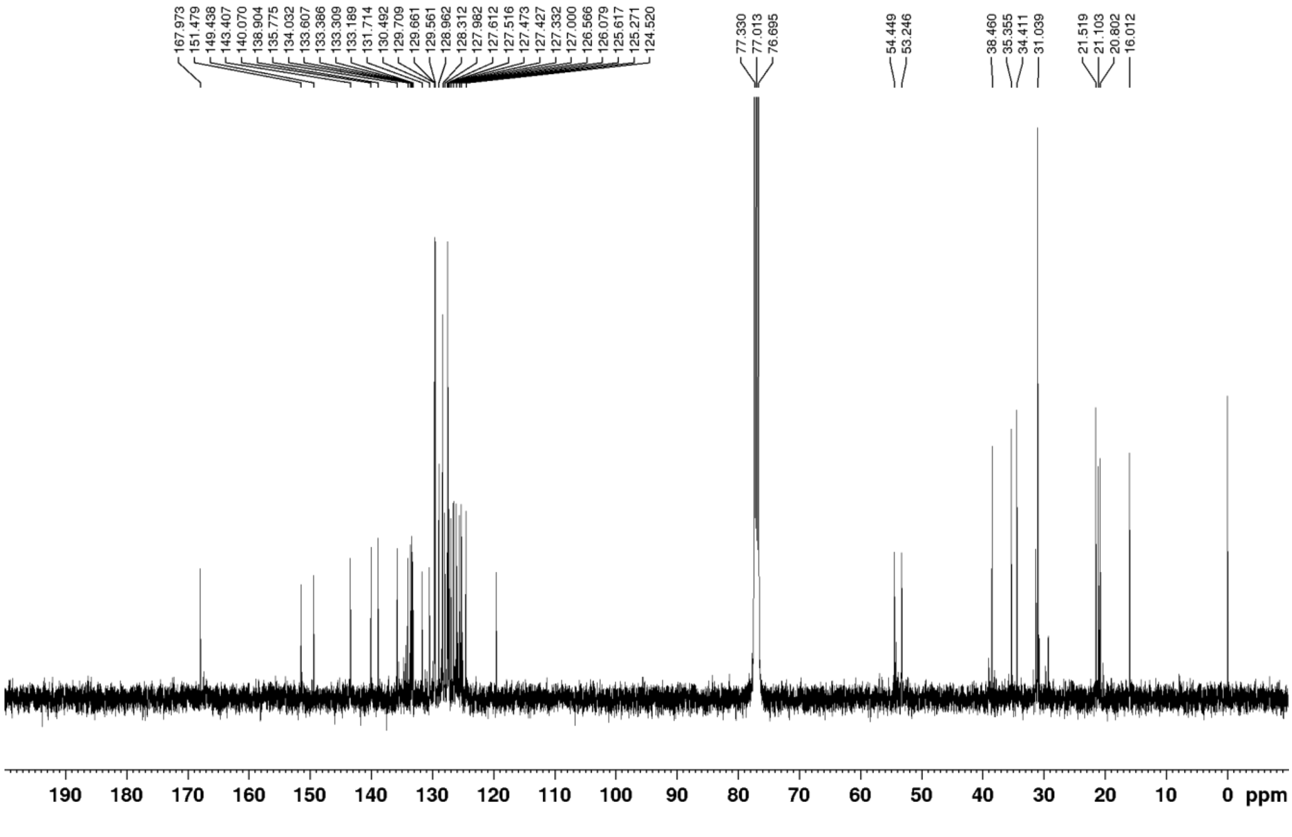

NOESY (CDCl3, 400 MHz)

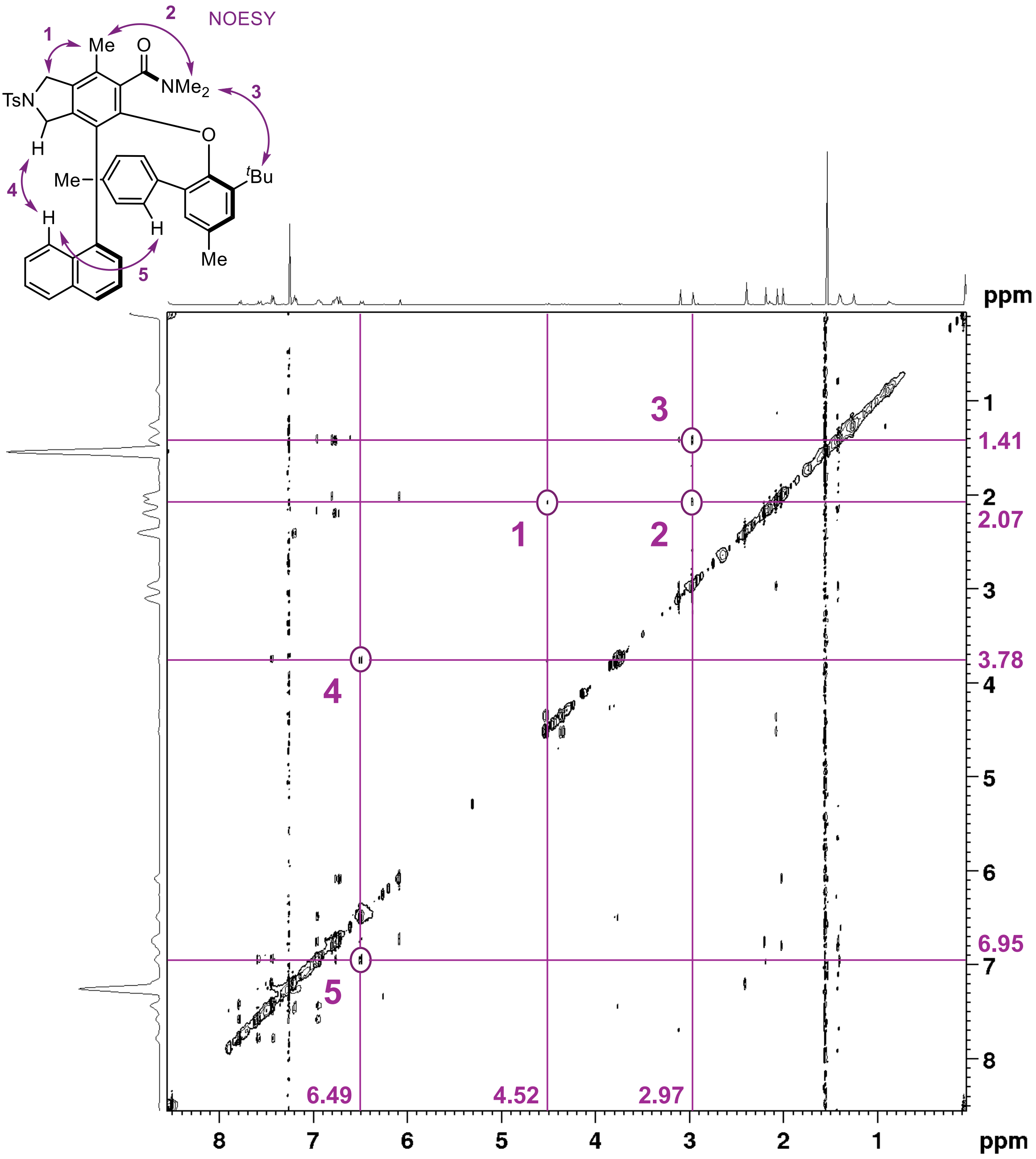

(+)-6-((3-(*tert*-Butyl)-4',5-dimethyl-[1,1'-biphenyl]-2-yl)oxy)-*N*,*N*,7-trimethyl-4-(naphthalen-1-yl)-2-tosylisoindoline-5-carboxamide [(+)-4no]

^{1}H NMR ($CDCl_3$, 400 MHz)

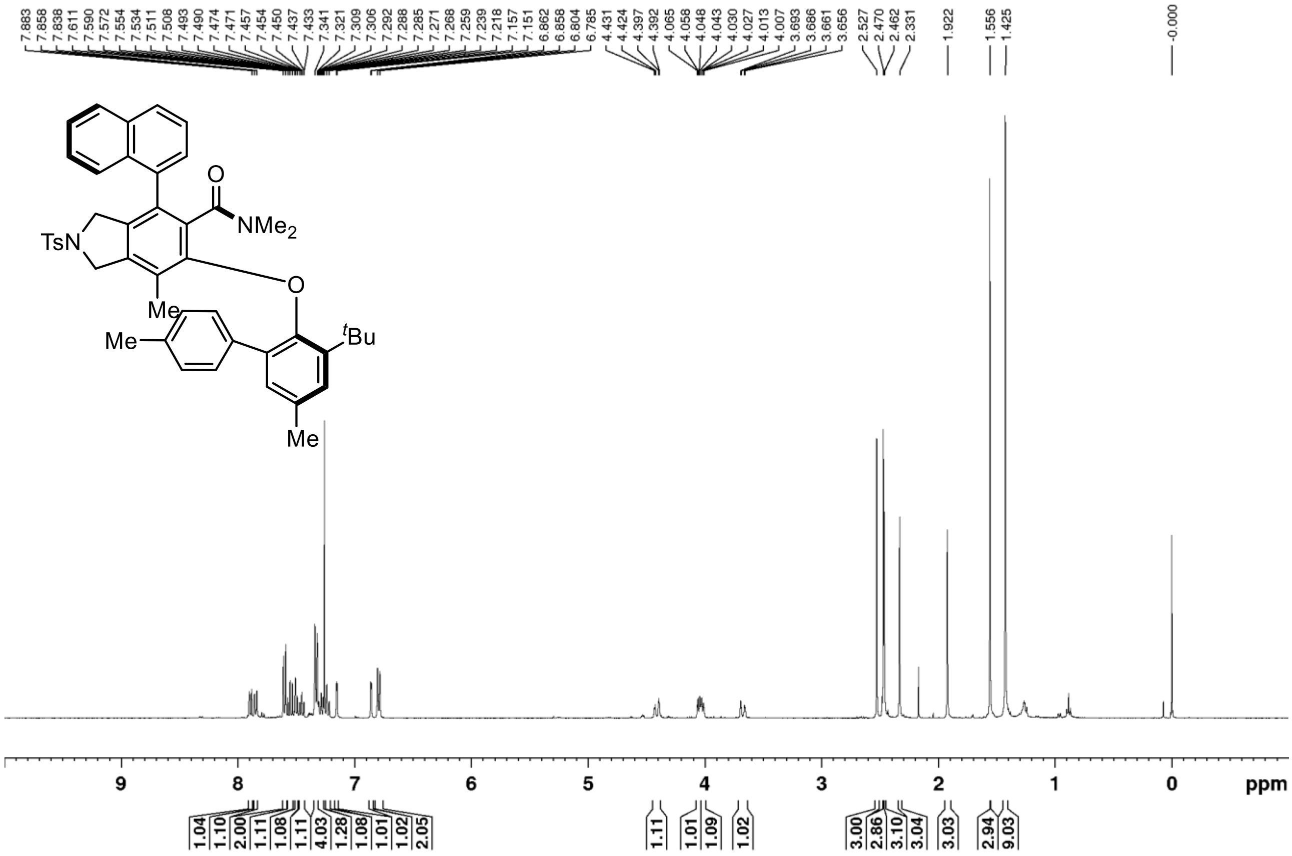

^{13}C NMR ($CDCl_3$, 101 MHz)

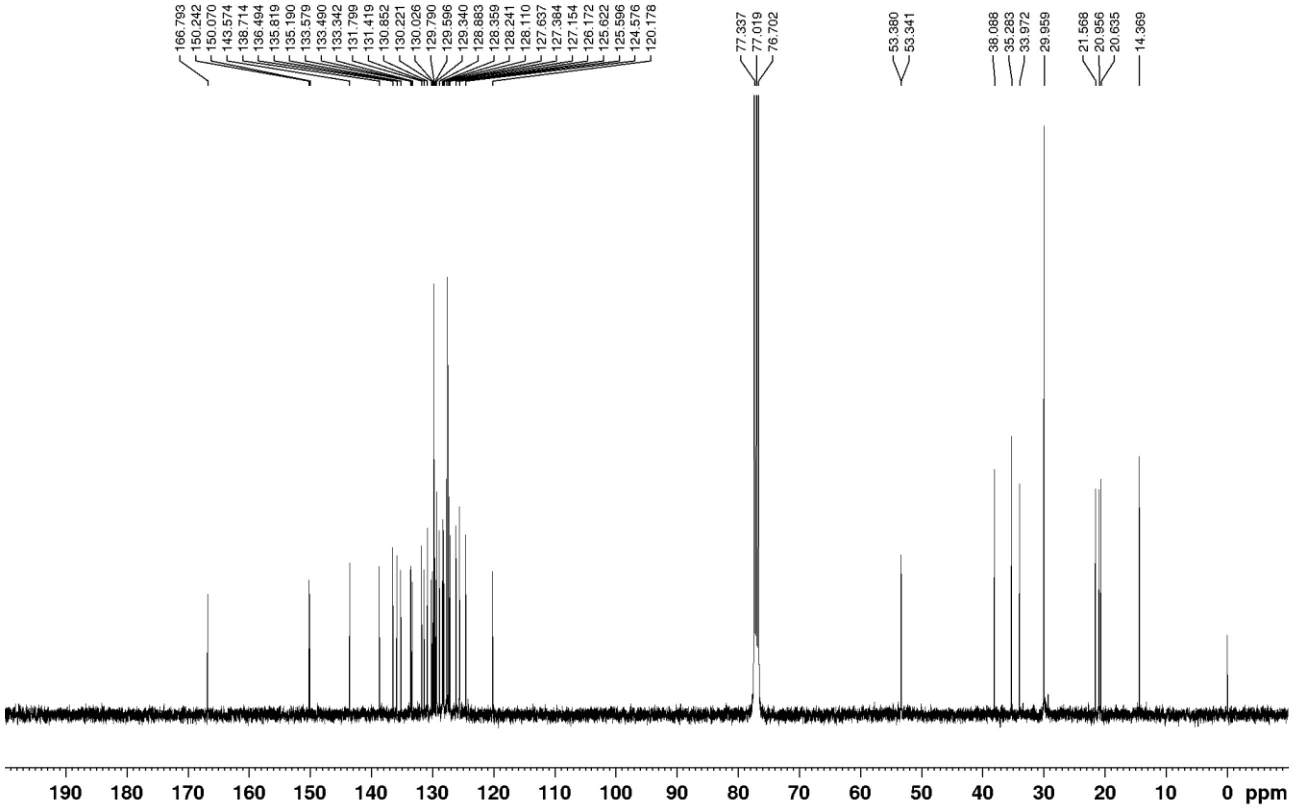

NOESY ($CDCl_3$, 400 MHz)

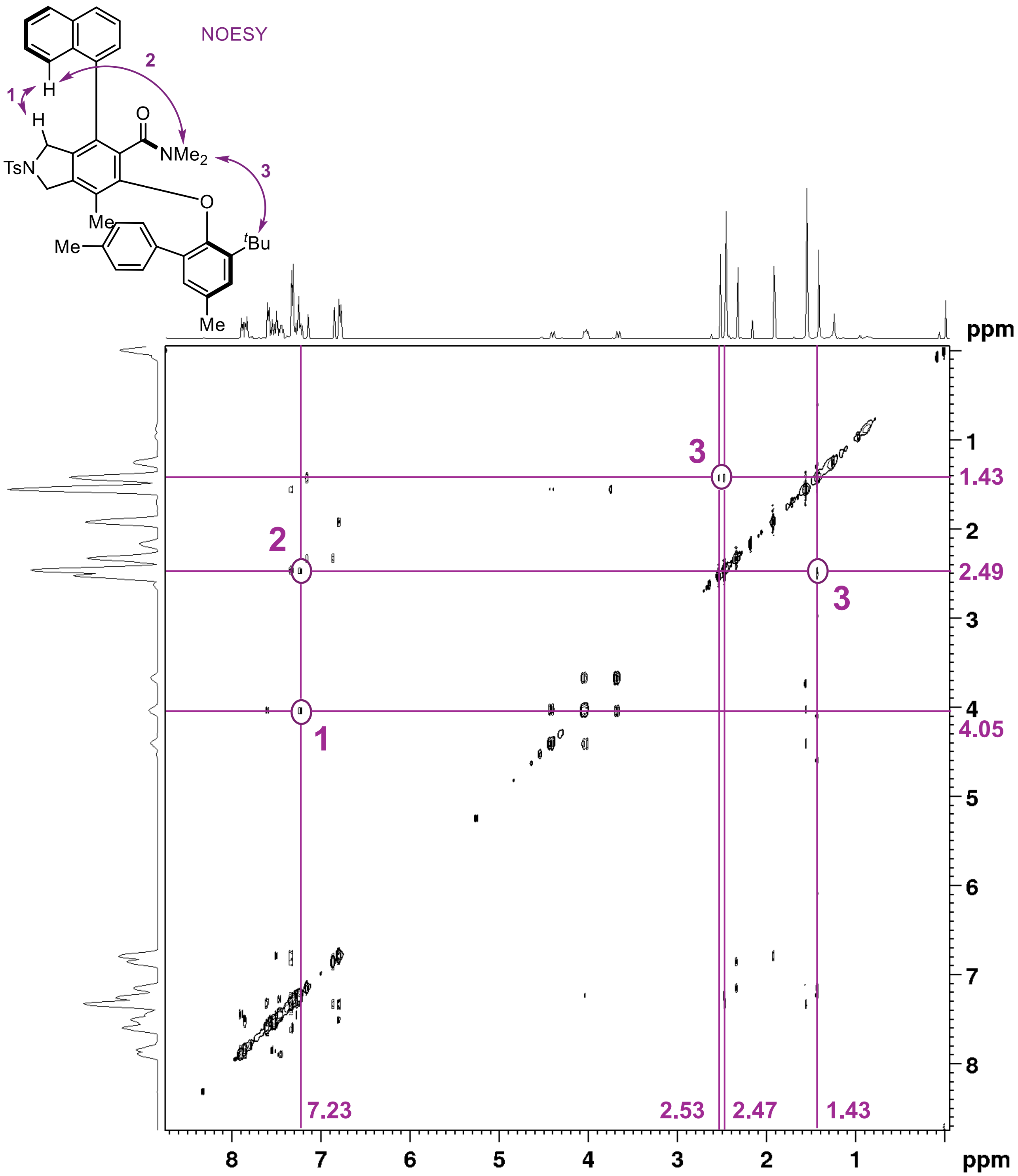

(+)-7-(2-Aminophenyl)-6-(2-(*tert*-butyl)-6-methylphenoxy)-*N*,*N*-dimethyl-4-(naphthalen-1-yl)-2-tosylisoindoline-5-carboxamide [(+)-3un]

^{1}H NMR [**3un**] ($CDCl_3$, 400 MHz)

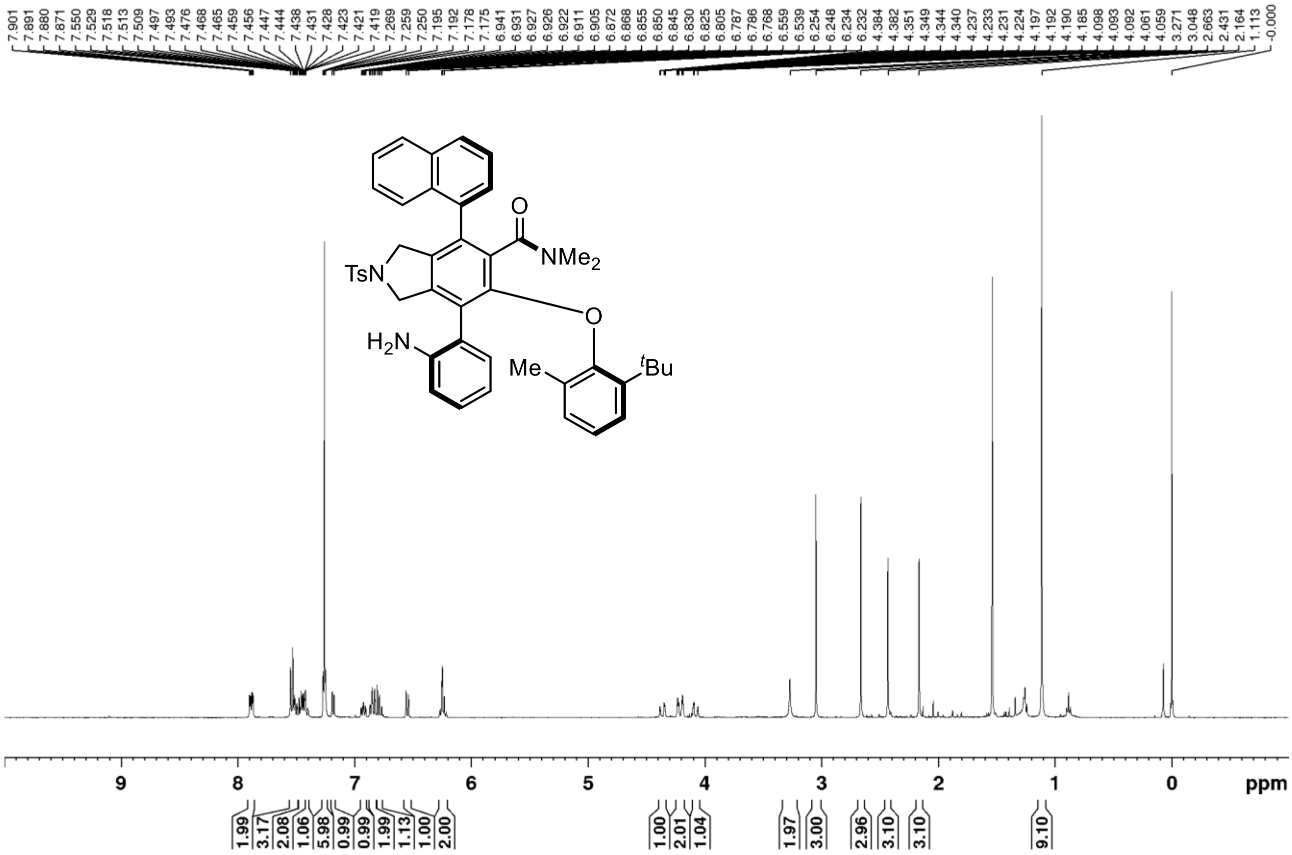

^{1}H NMR [**3un'**] ($CDCl_3$, 400 MHz)

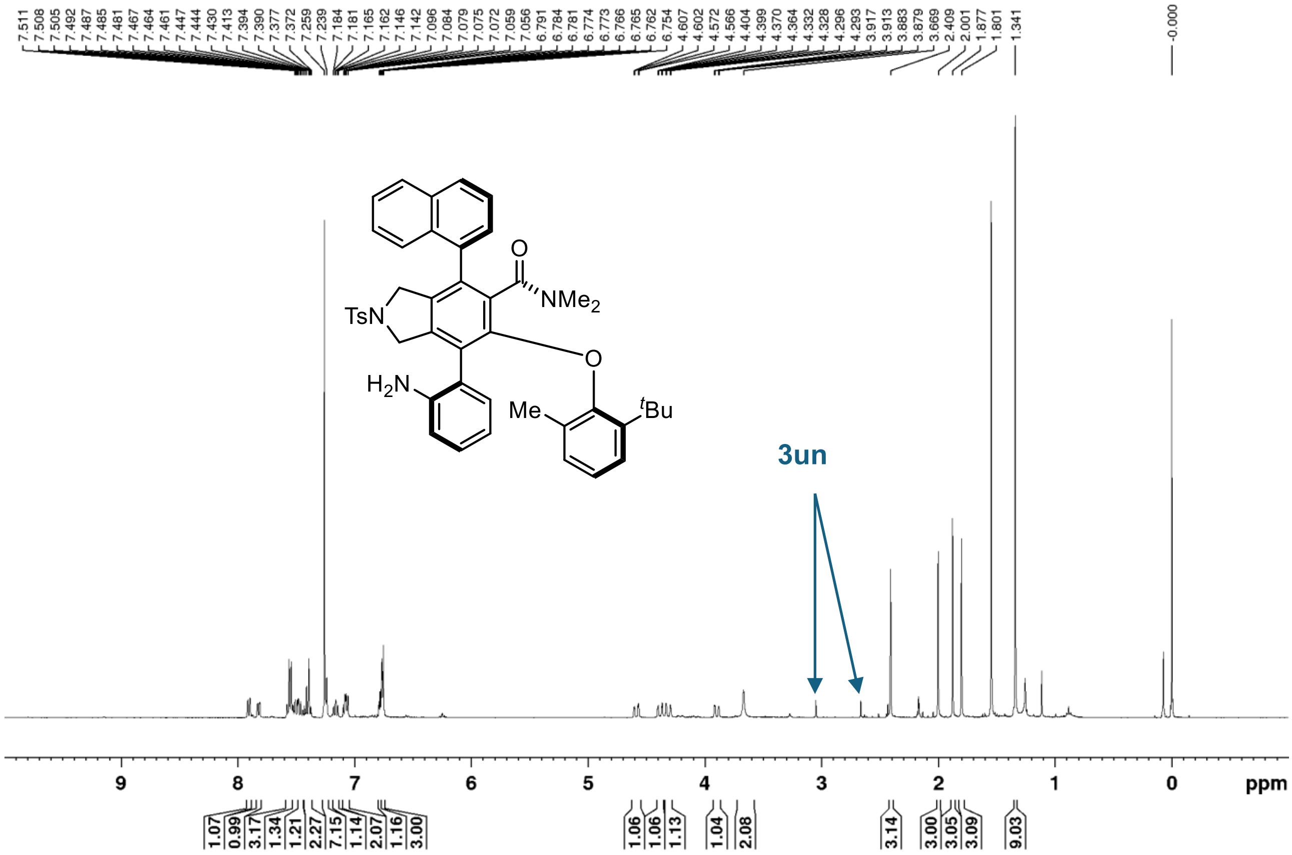

^{13}C NMR ($CDCl_3$, 101 MHz)

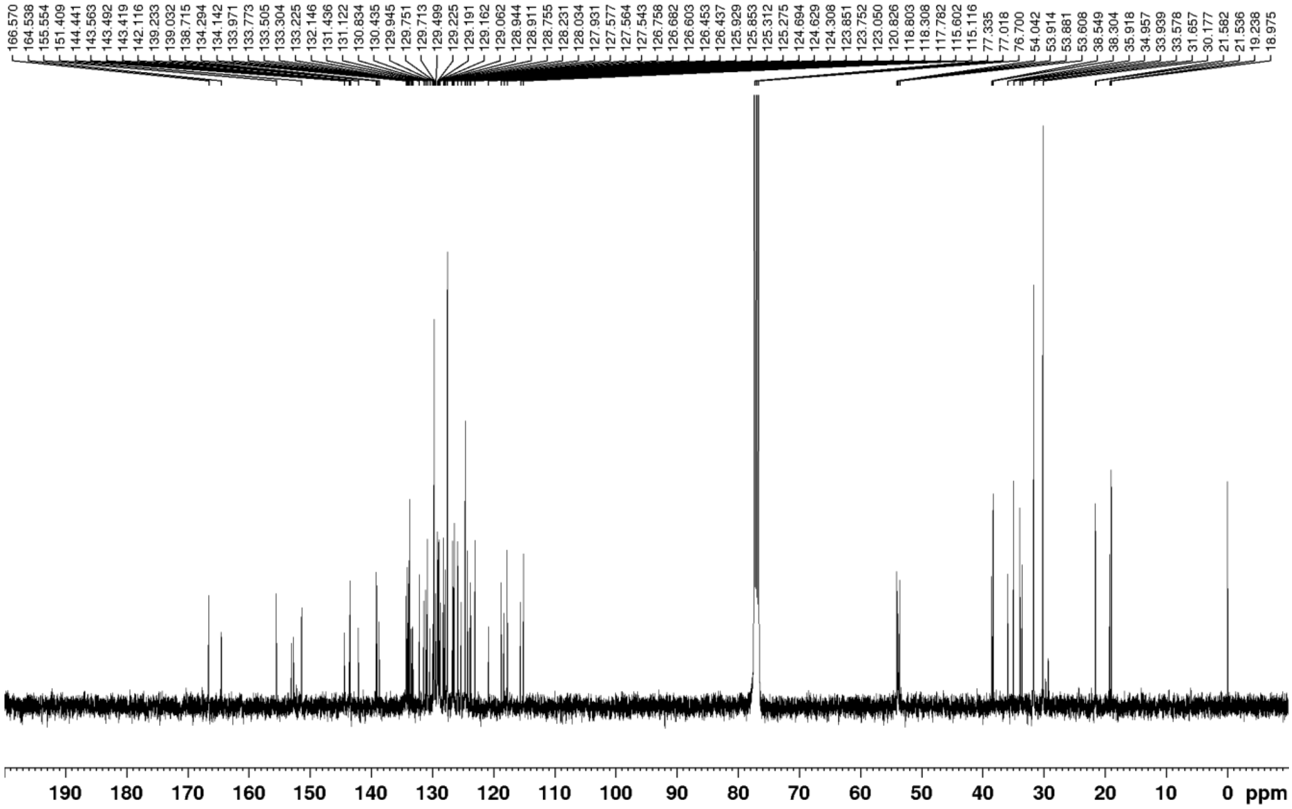

NOESY (CDCl3, 400 MHz)

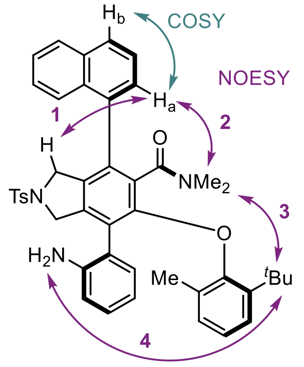

3un

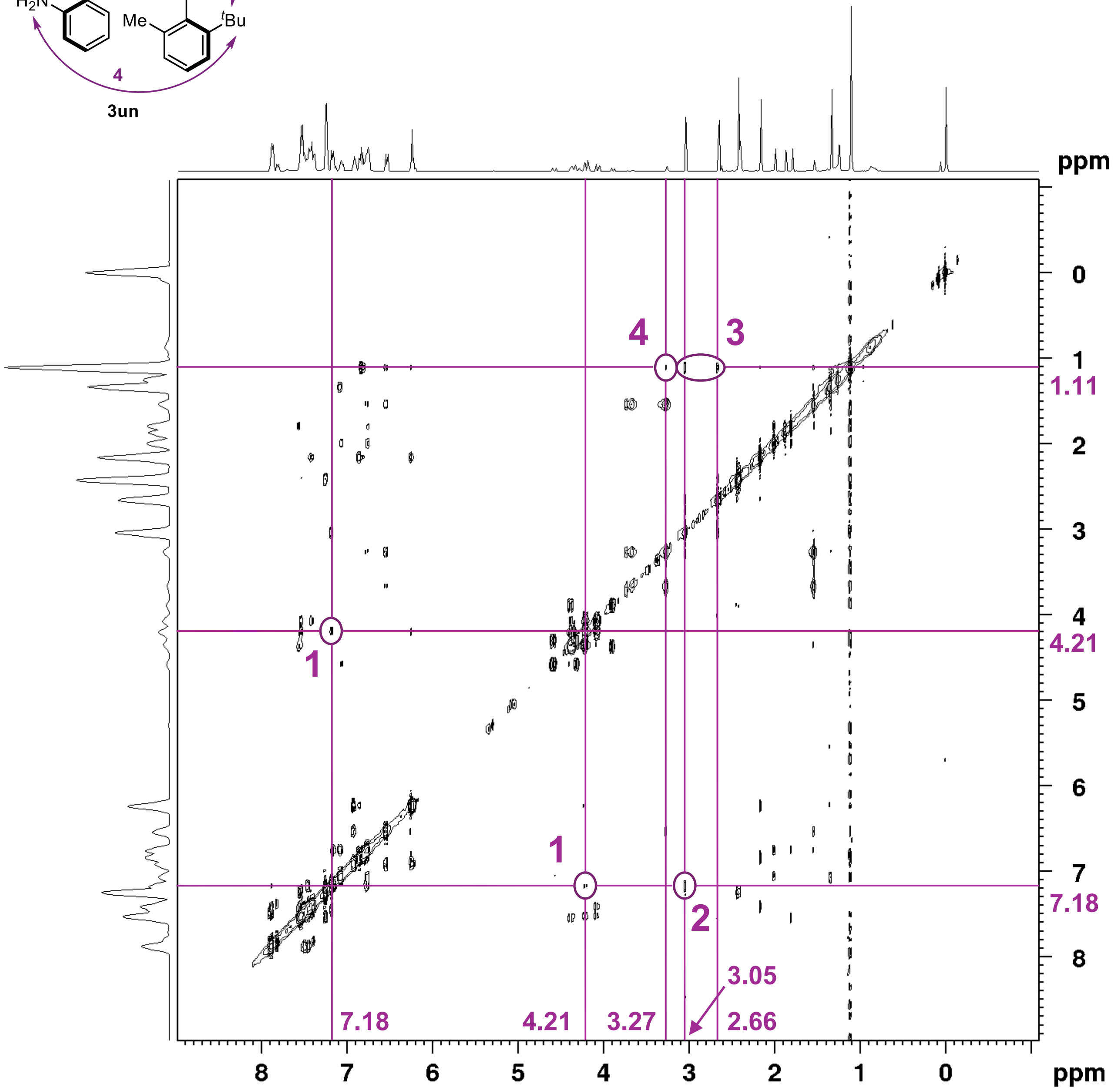

COSY ($CDCl_3$, 400 MHz)

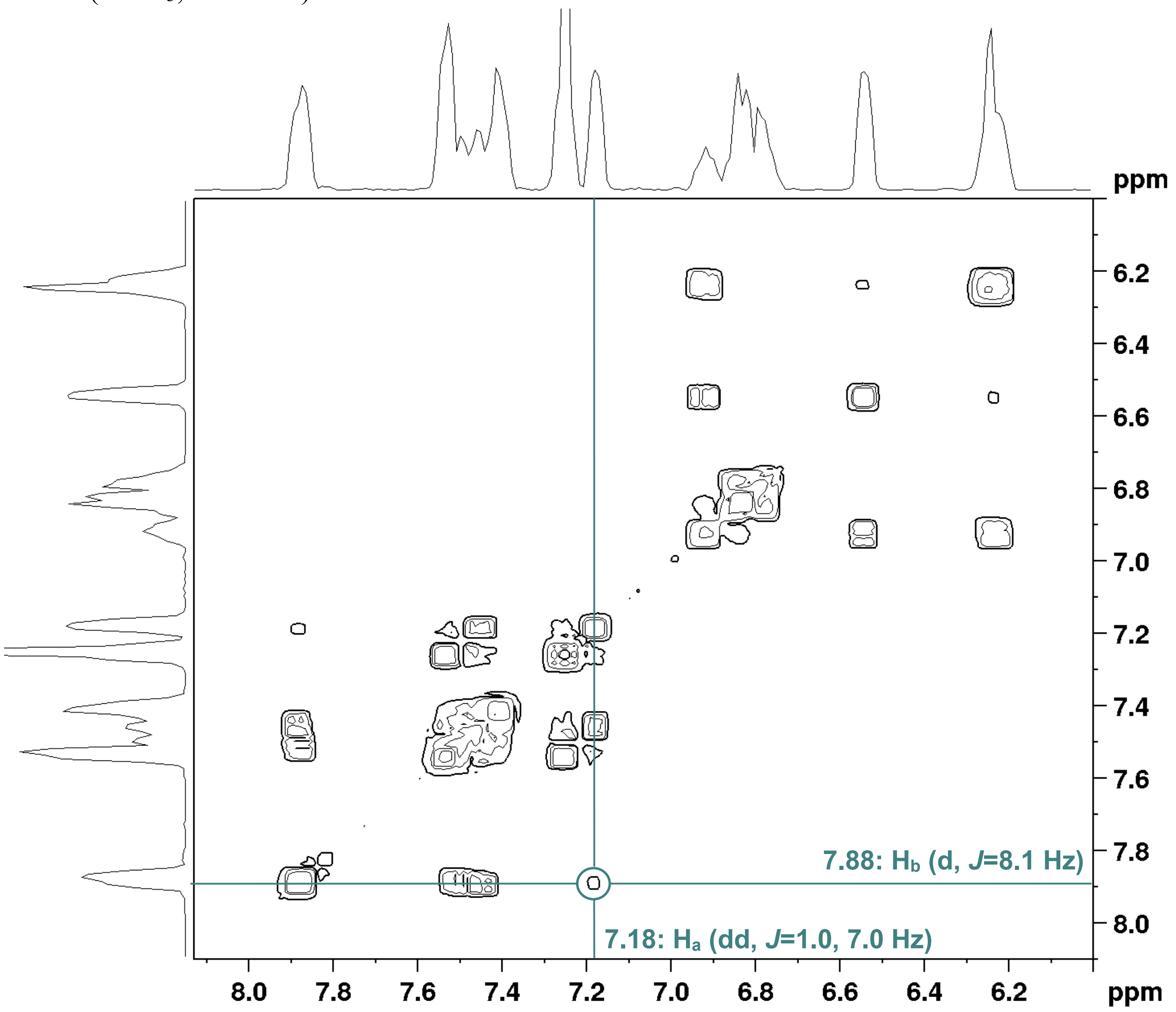

NOESY (CDCl3, 400 MHz)

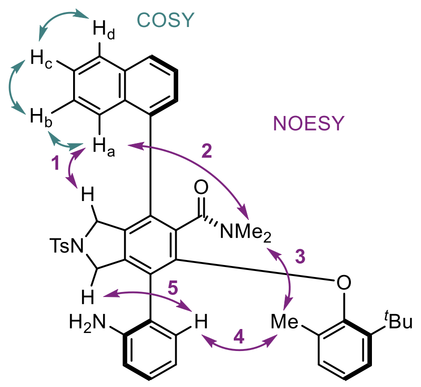

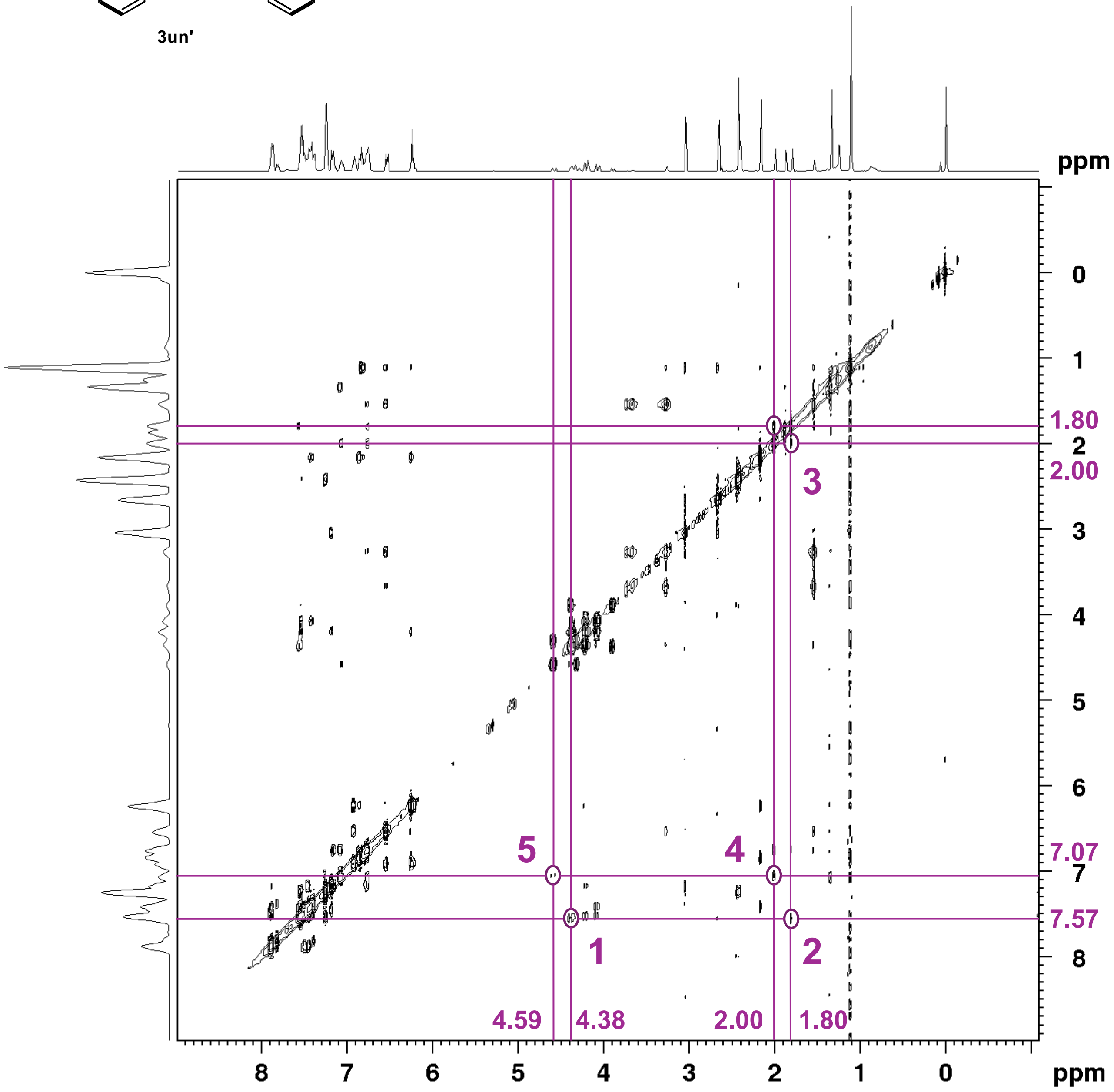

NOESY (CDCl3, 400 MHz)

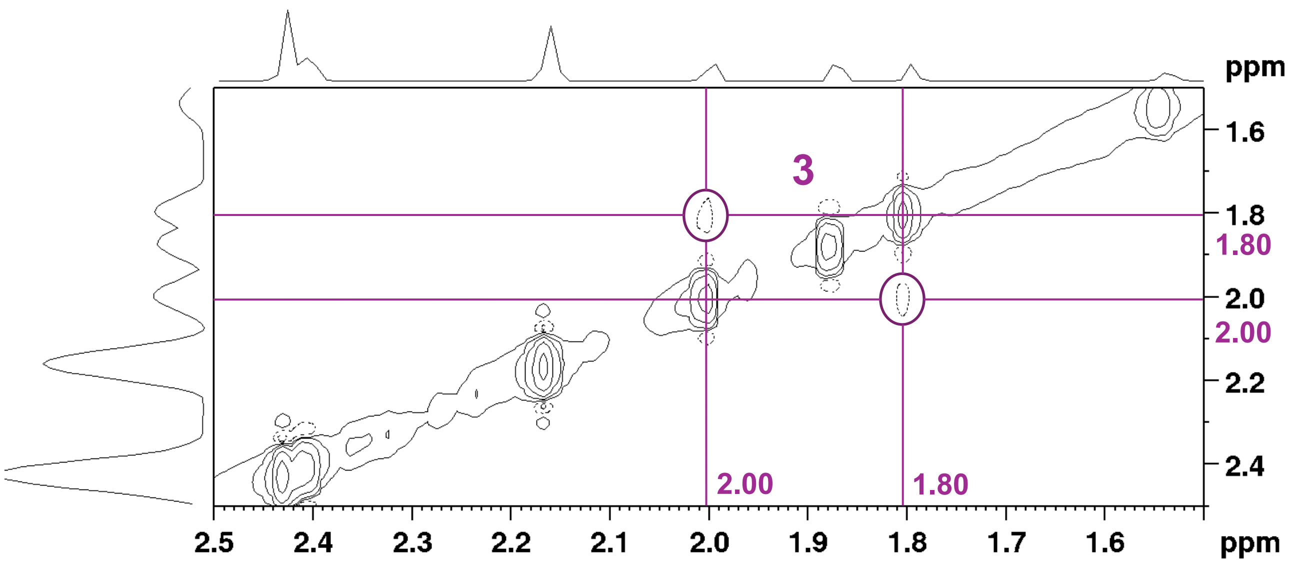

COSY (CDCl3, 400 MHz)

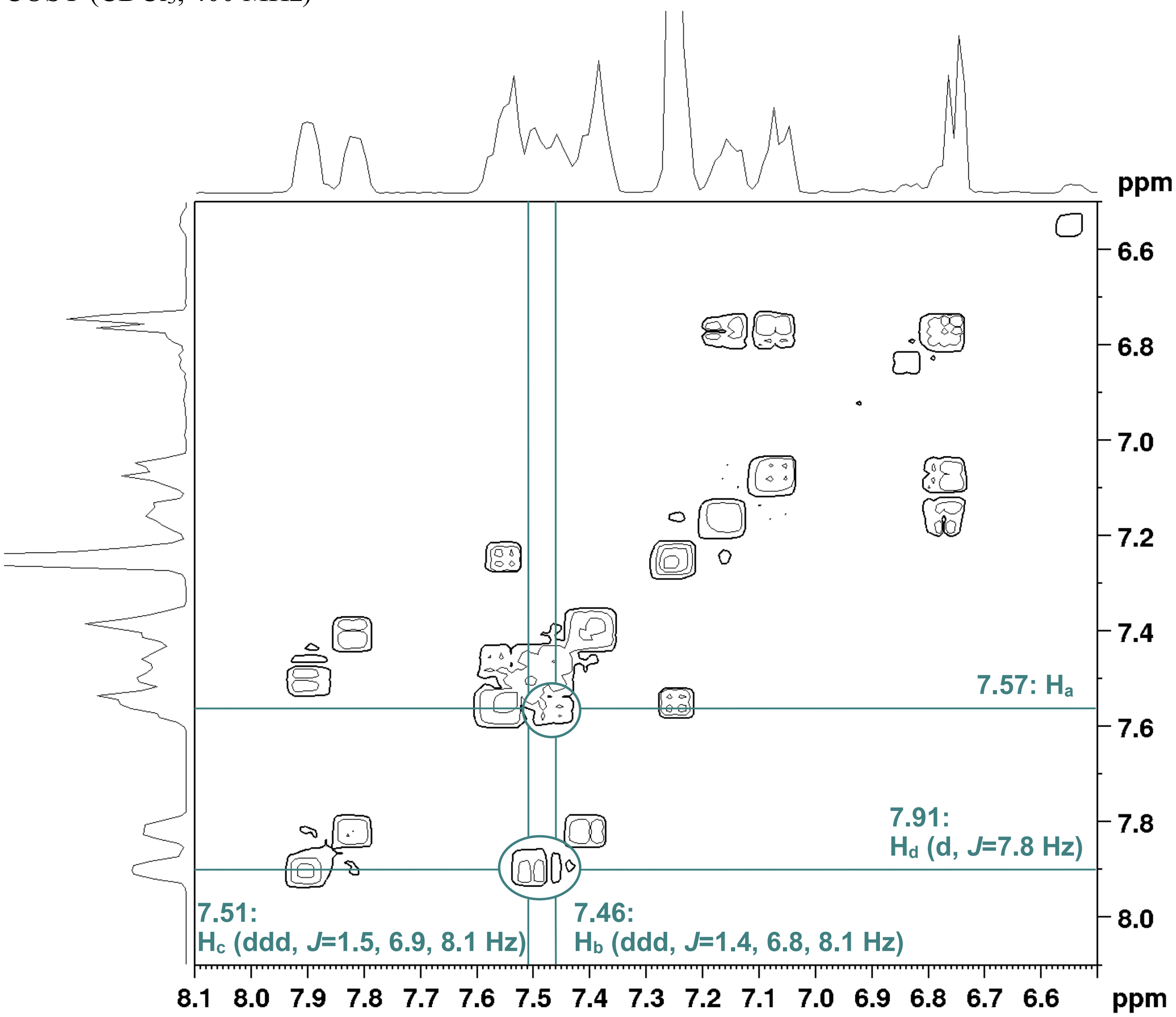

(+)-7-(2-Aminophenyl)-6-(2-(*tert*-butyl)-6-methylphenoxy)-*N*,*N*-dimethyl-4-(naphthalen-1-yl)-2-tosylisoindoline-5-carboxamide [(+)-3un'']

^{1}H NMR ($CDCl_3$, 400 MHz)

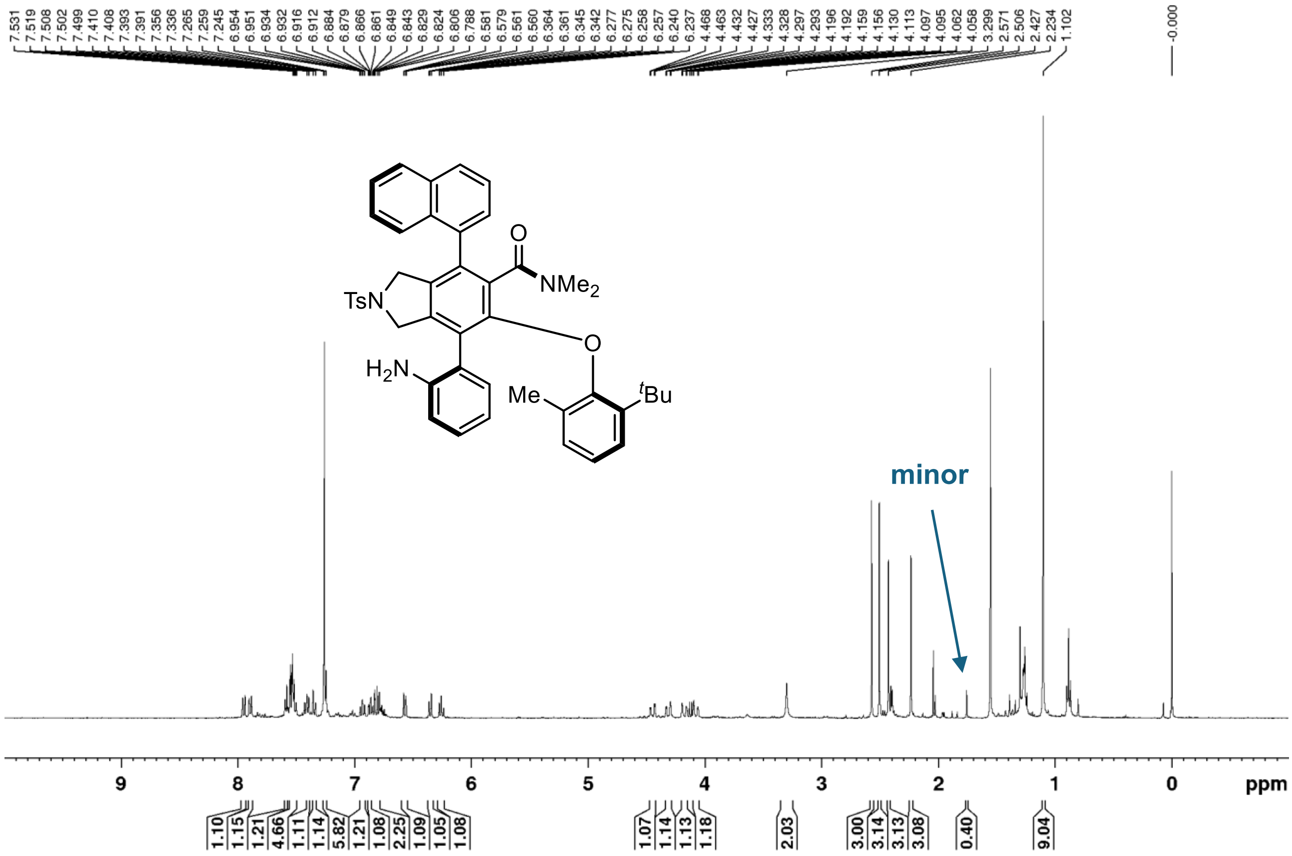

^{13}C NMR ($CDCl_3$, 101 MHz)

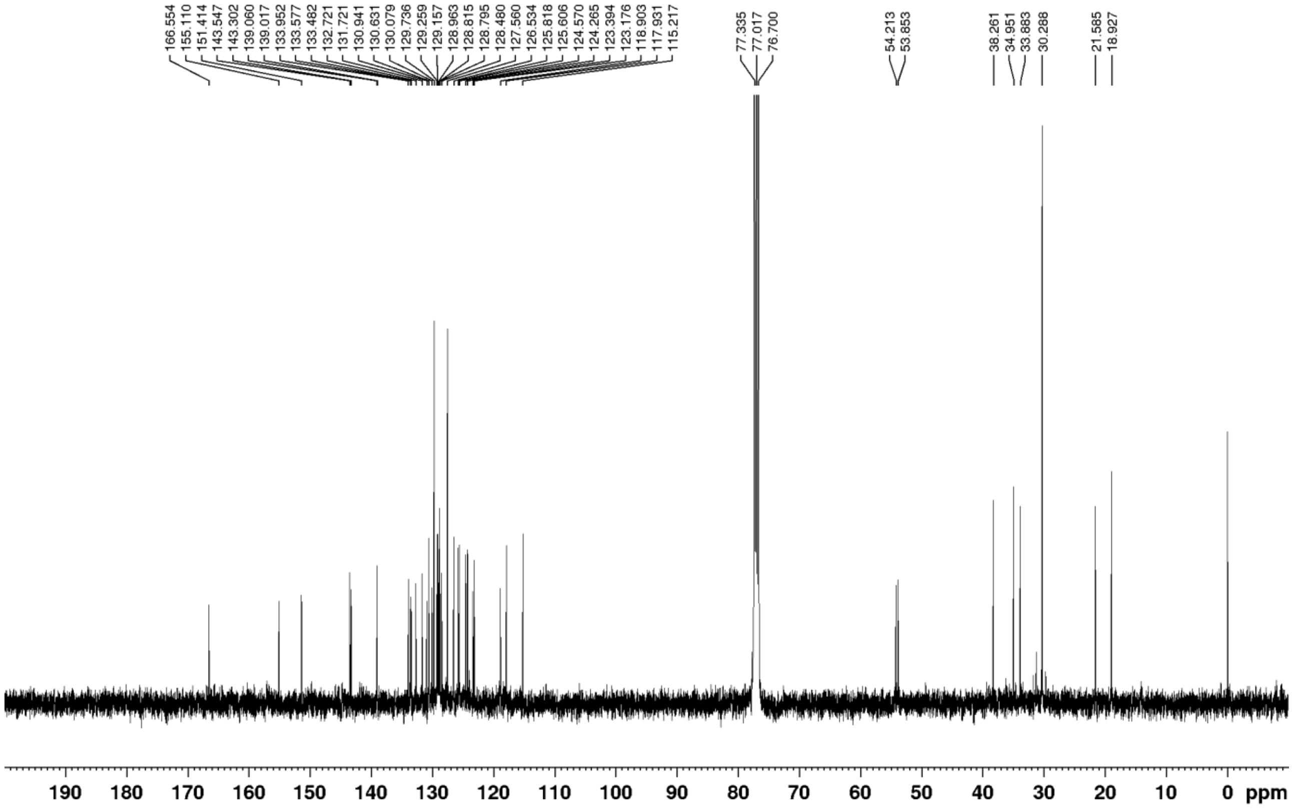

NOESY ($CDCl_3$, 400 MHz)

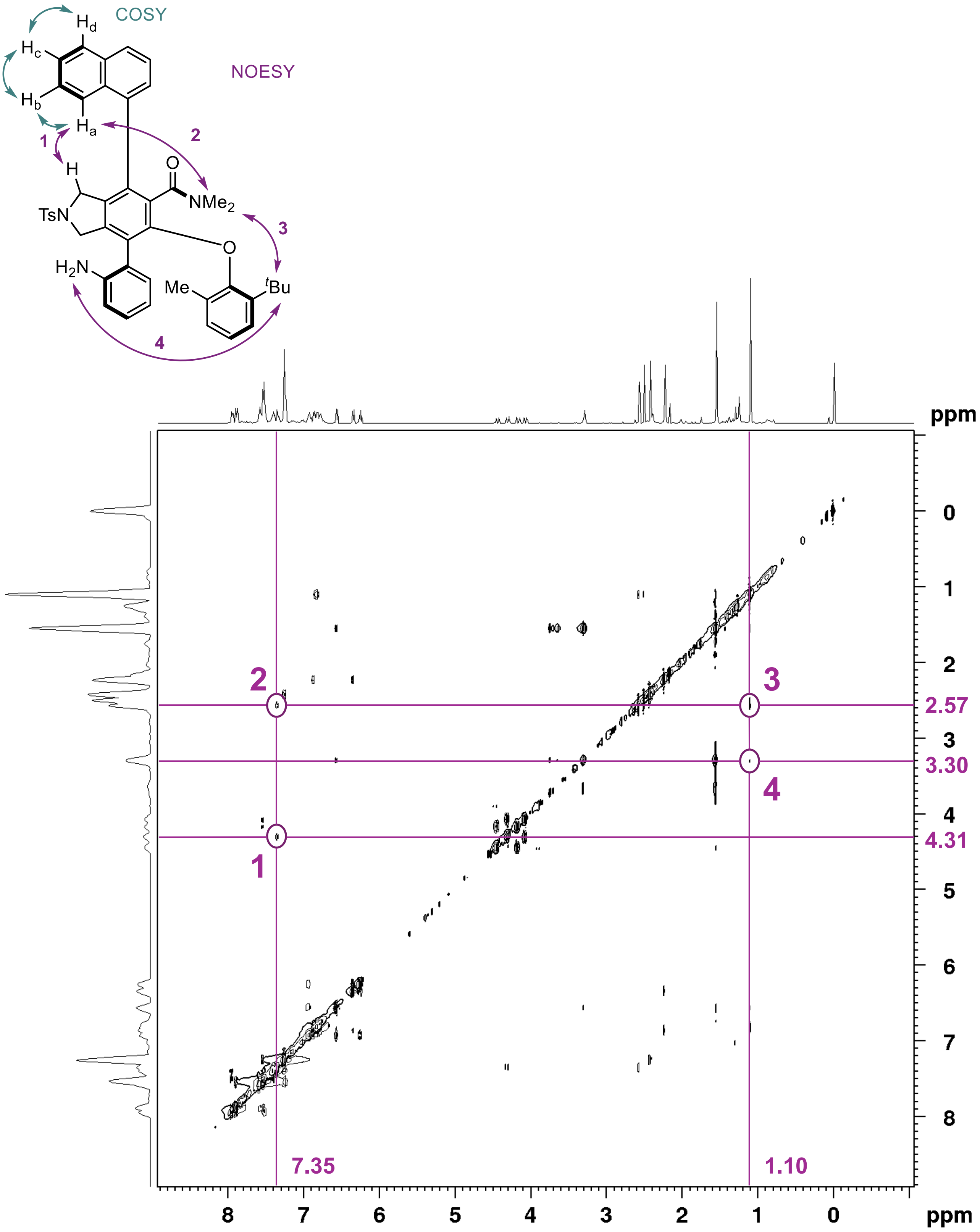

COSY ($CDCl_3$, 400 MHz)

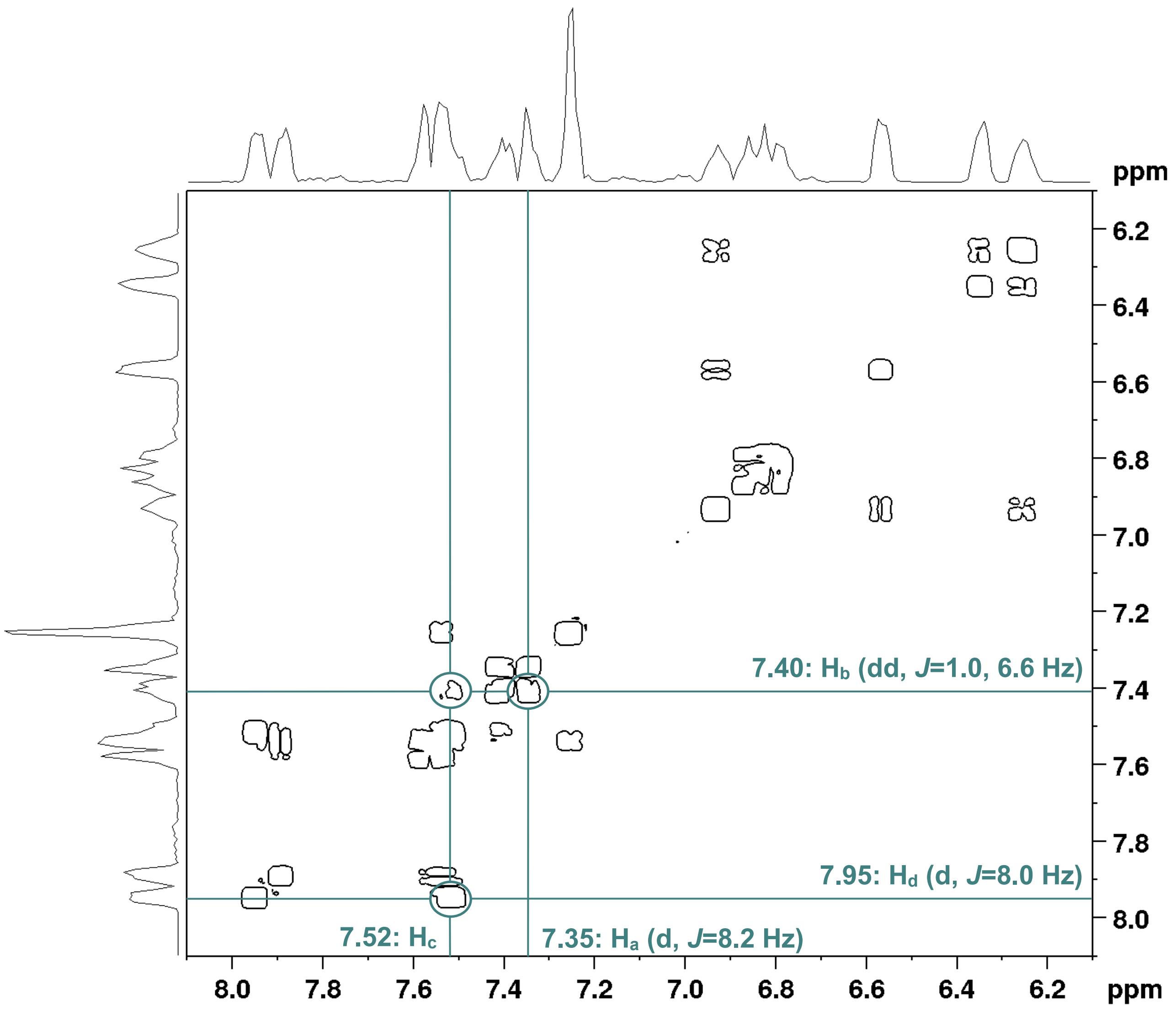

(+)-Methyl 6-(2-(*tert*-butyl)-6-formylphenoxy)-7-(2-hydroxyphenyl)-4-methyl-2-tosylisoindoline-5-carboxylate [(+)-5]

^{1}H NMR ($CDCl_3$, 400 MHz)

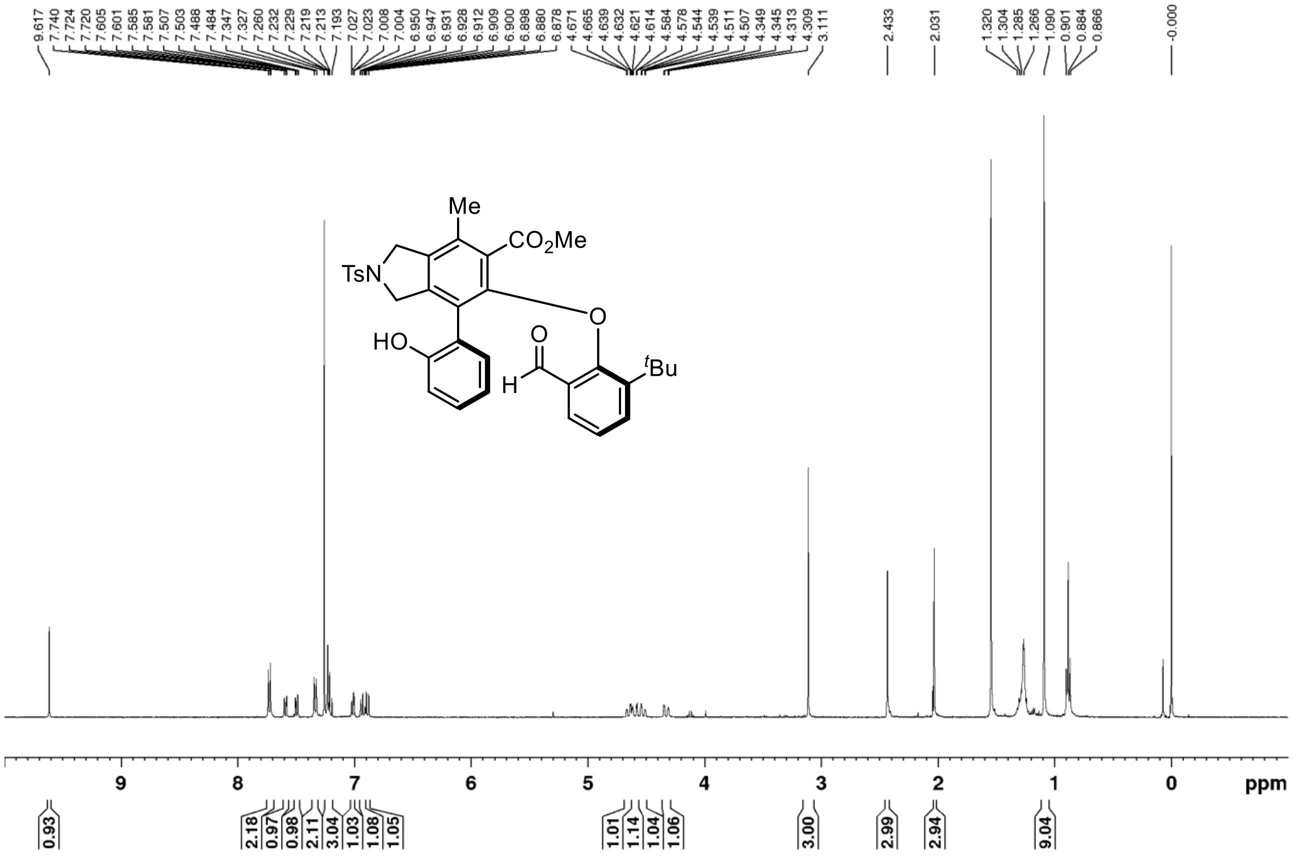

^{13}C NMR ($CDCl_3$, 101 MHz)

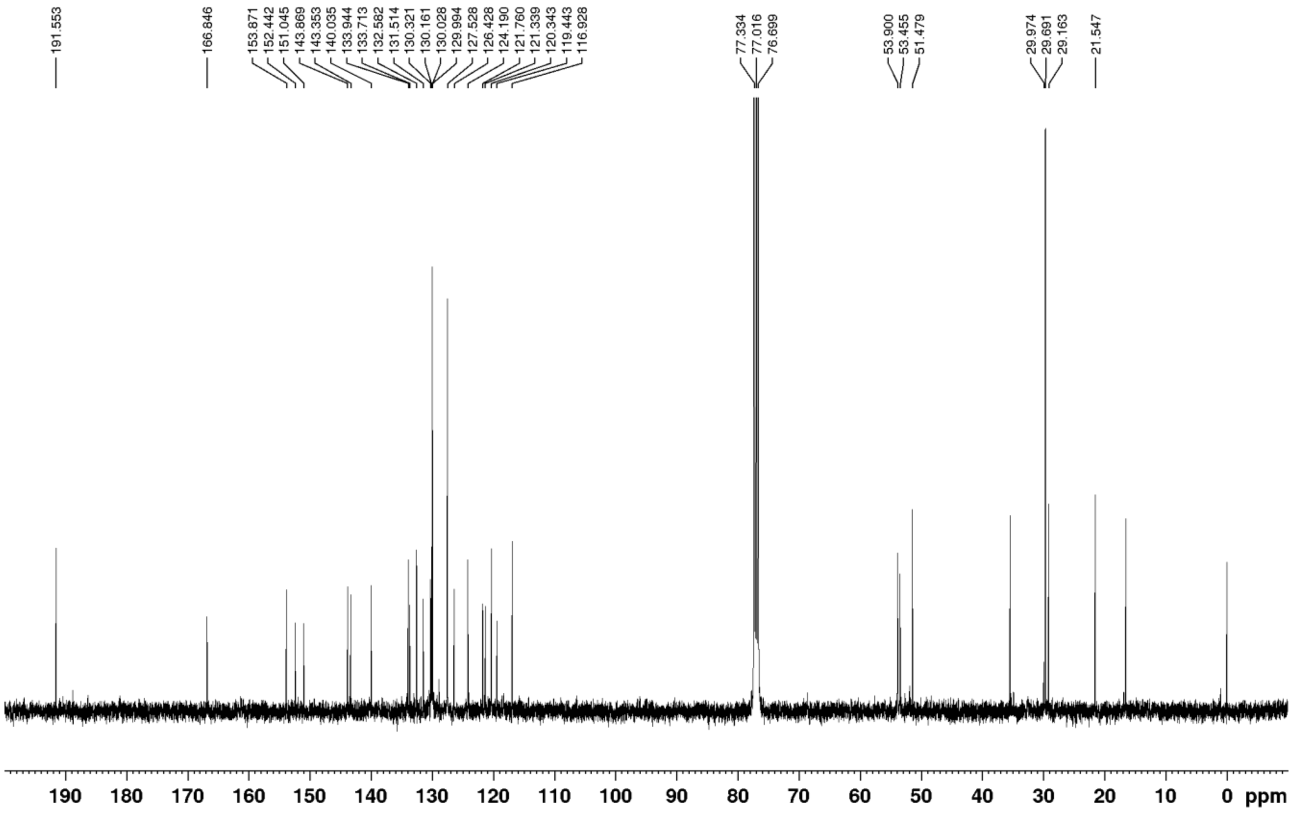

NOESY ($CDCl_3$, 400 MHz)

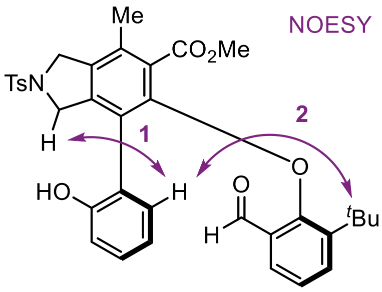

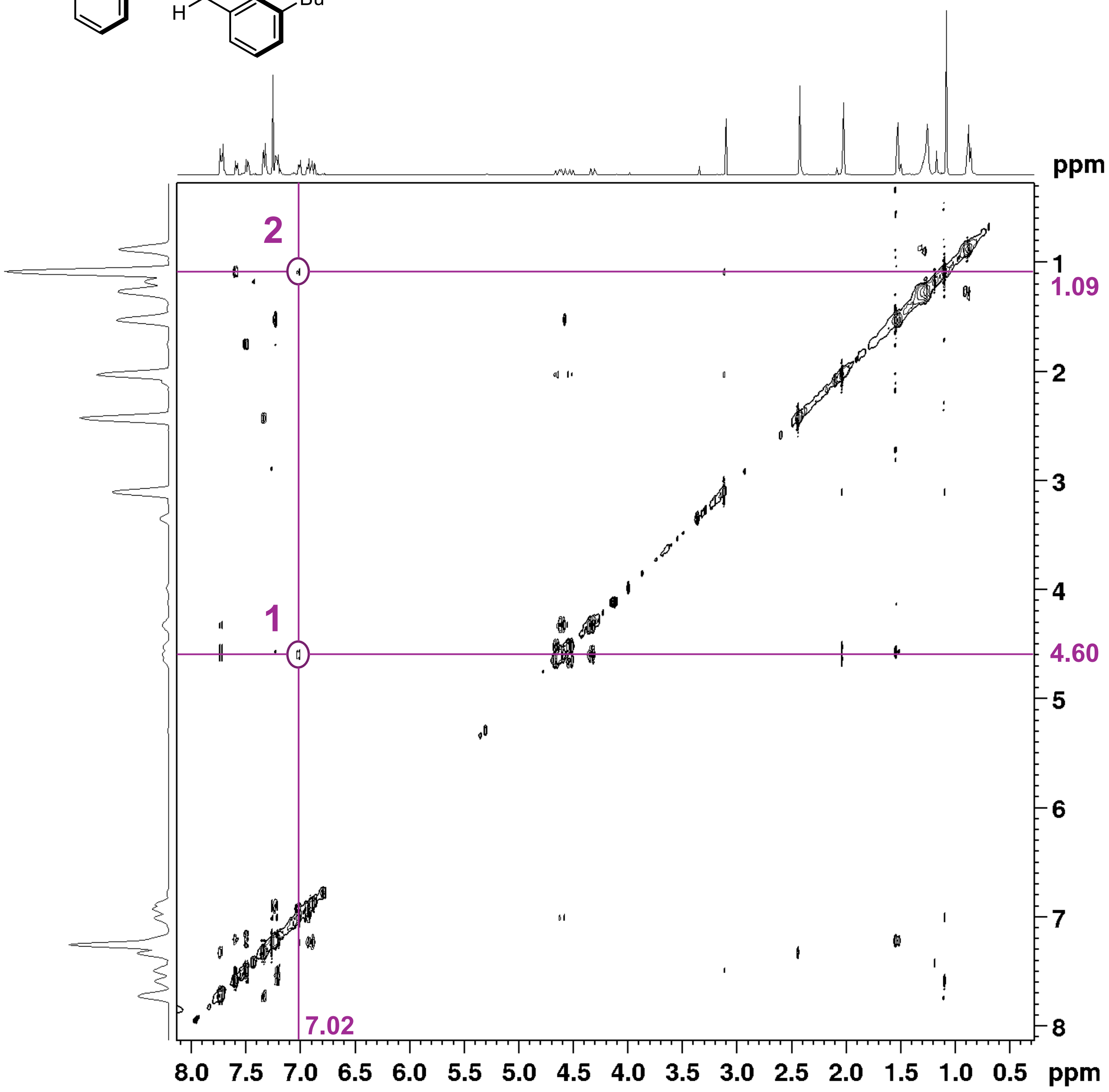

(–)-(7-(2-Aminophenyl)-6-(2-(*tert*-butyl)-6-methylphenoxy)-4-methyl-2-tosylisoindolin-5-yl)methanol [(–)-6]

^{1}H NMR ($CDCl_3$, 400 MHz)

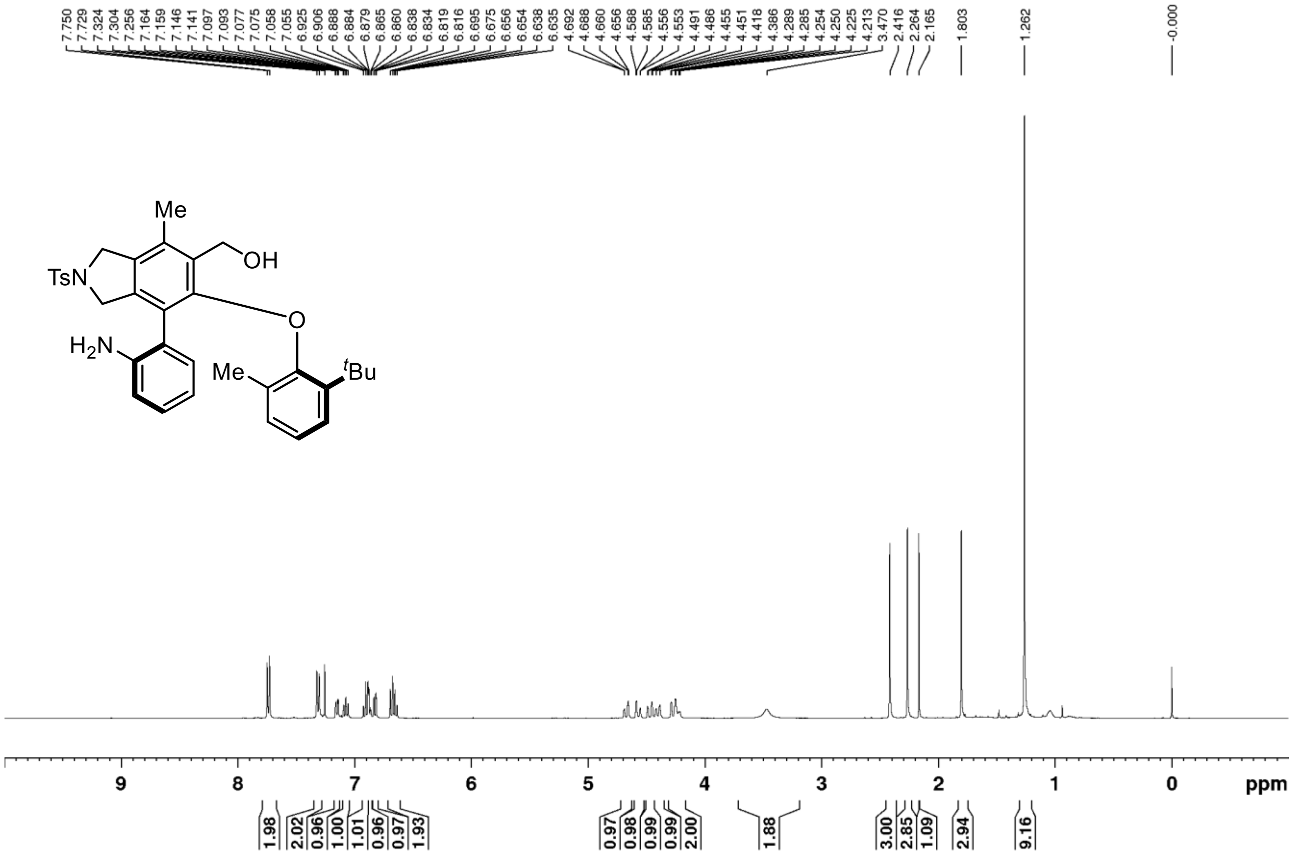

^{13}C NMR ($CDCl_3$, 101 MHz)

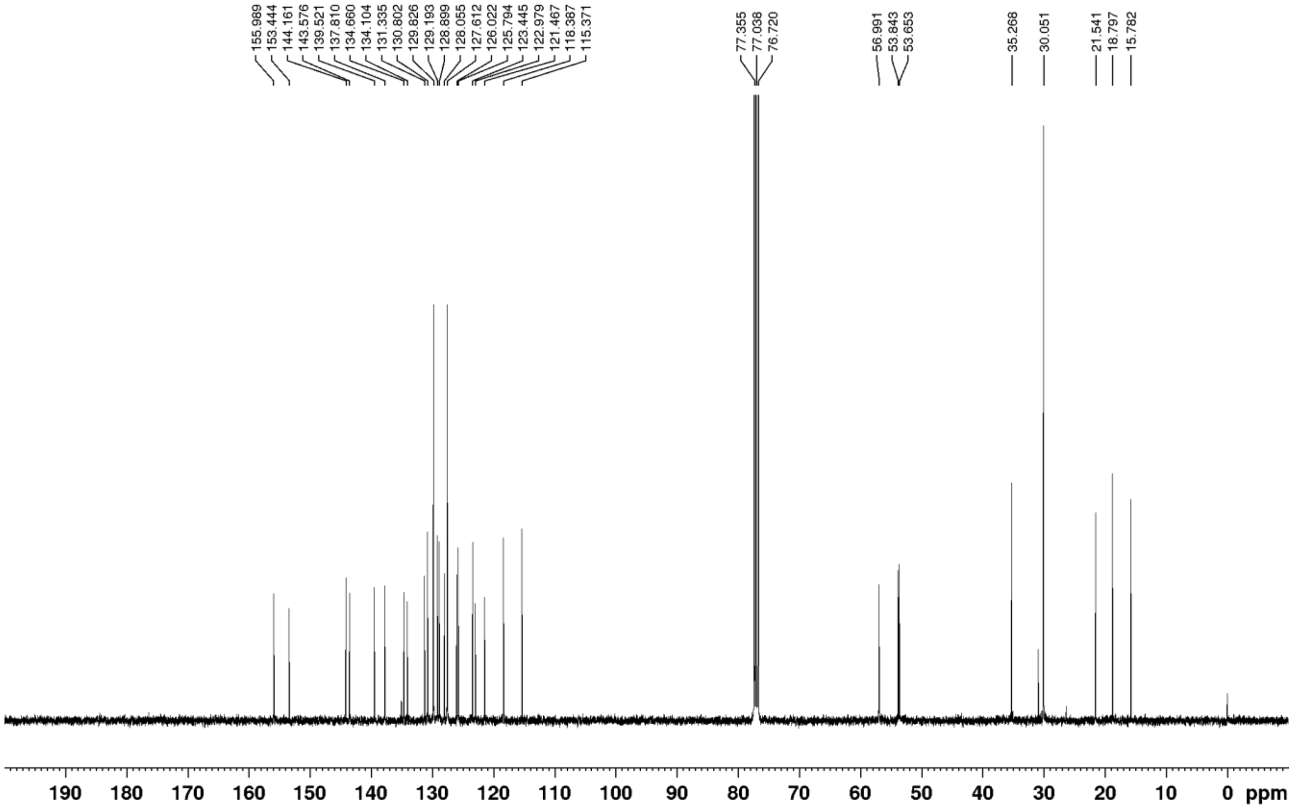

(–)-2-(5-(2-(*tert*-Butyl)-6-methylphenoxy)-7-methyl-6-((p-tolyloxy)methyl)-2-tosylisoindolin-4-yl)aniline [(–)-7]

^{1}H NMR ($CDCl_3$, 400 MHz)

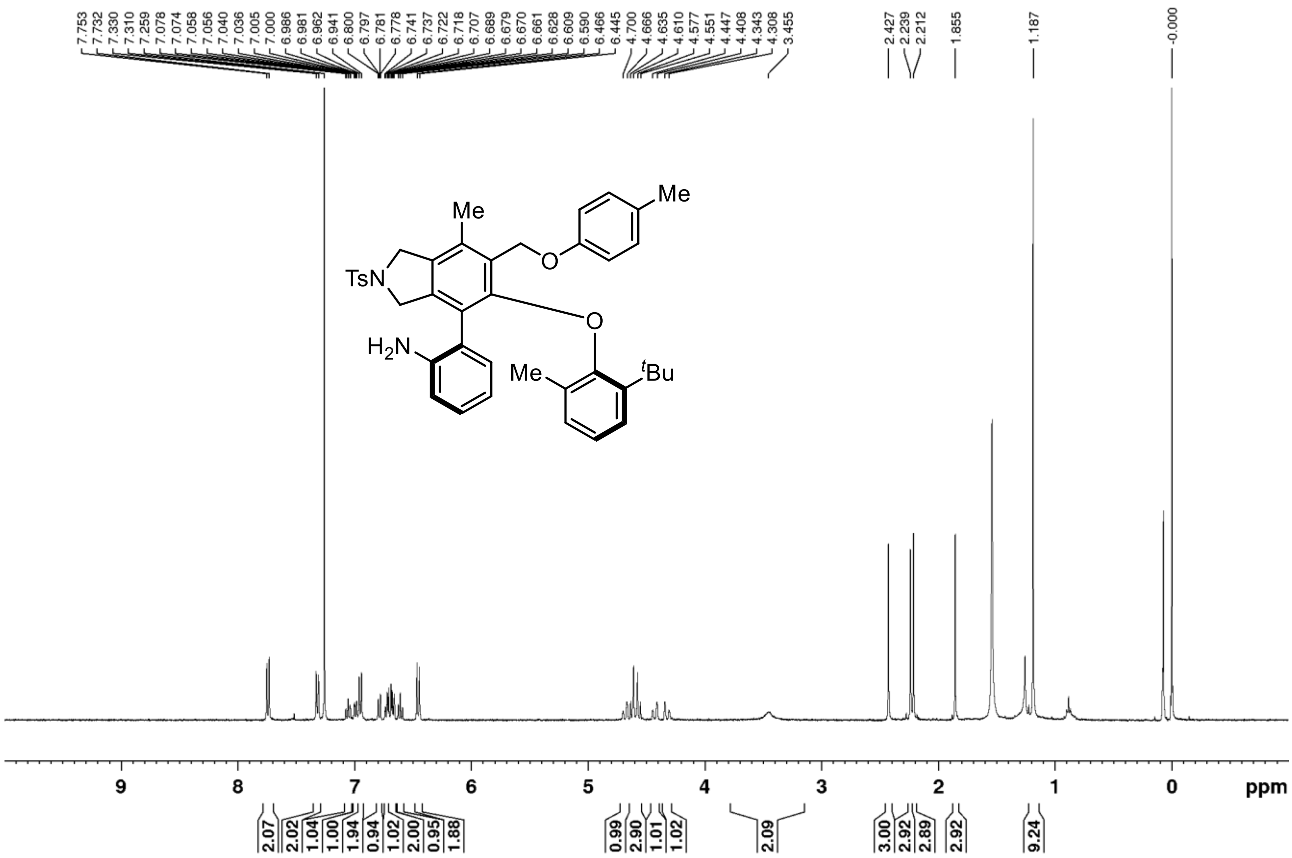

^{13}C NMR ($CDCl_3$, 101 MHz)

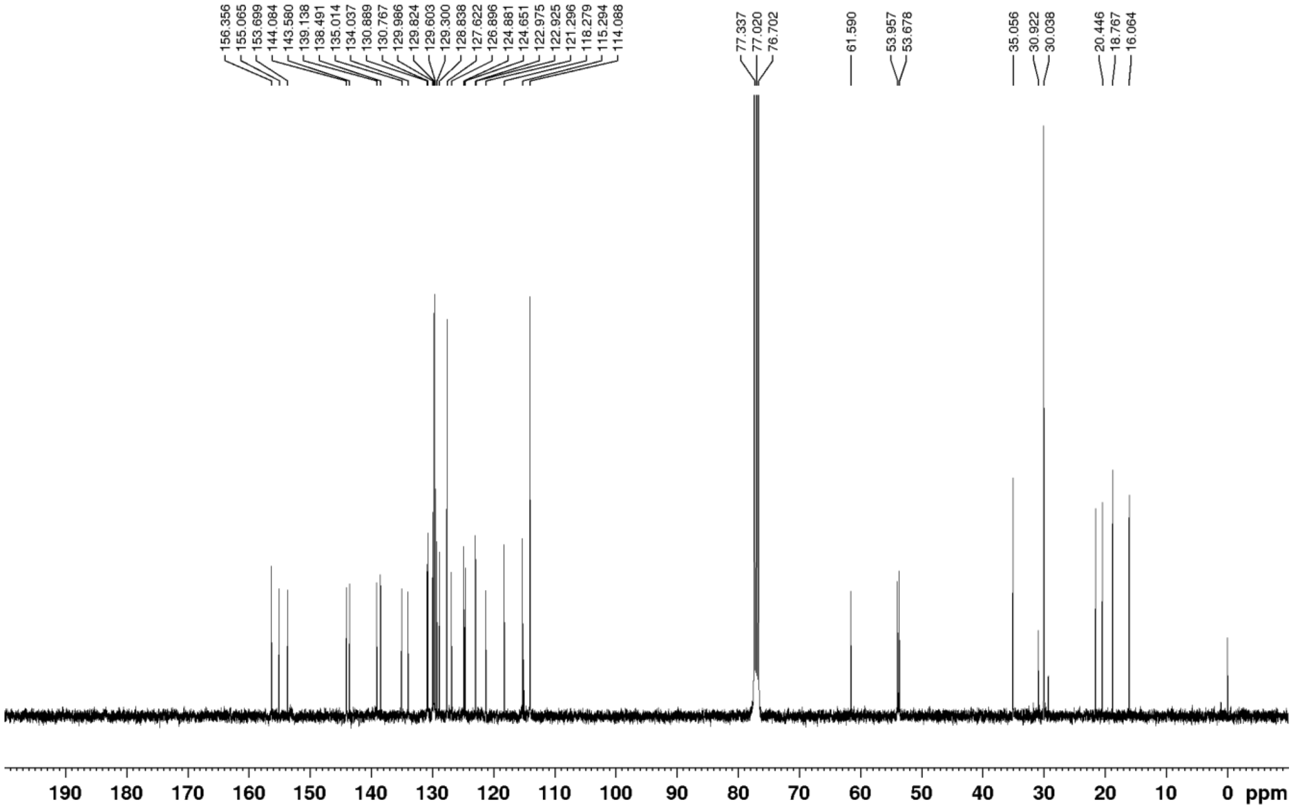

(+)-Methyl 6-(2-(*tert*-butyl)-6-methylphenoxy)-5-(methoxymethyl)-7-methyl-2-tosylisoindoline-4-carboxylate [(+)-3ap]

^{1}H NMR ($CDCl_3$, 400 MHz)

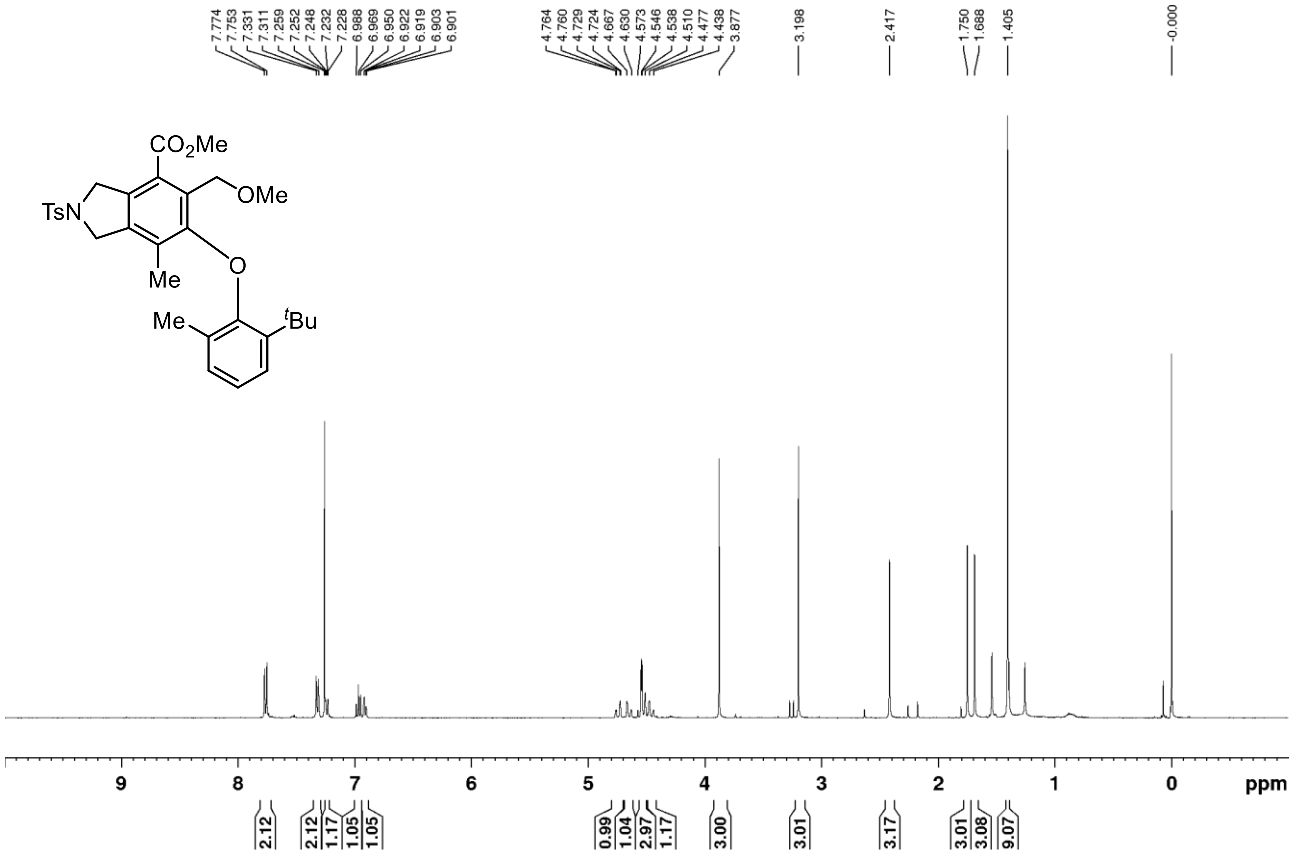

^{13}C NMR ($CDCl_3$, 101 MHz)

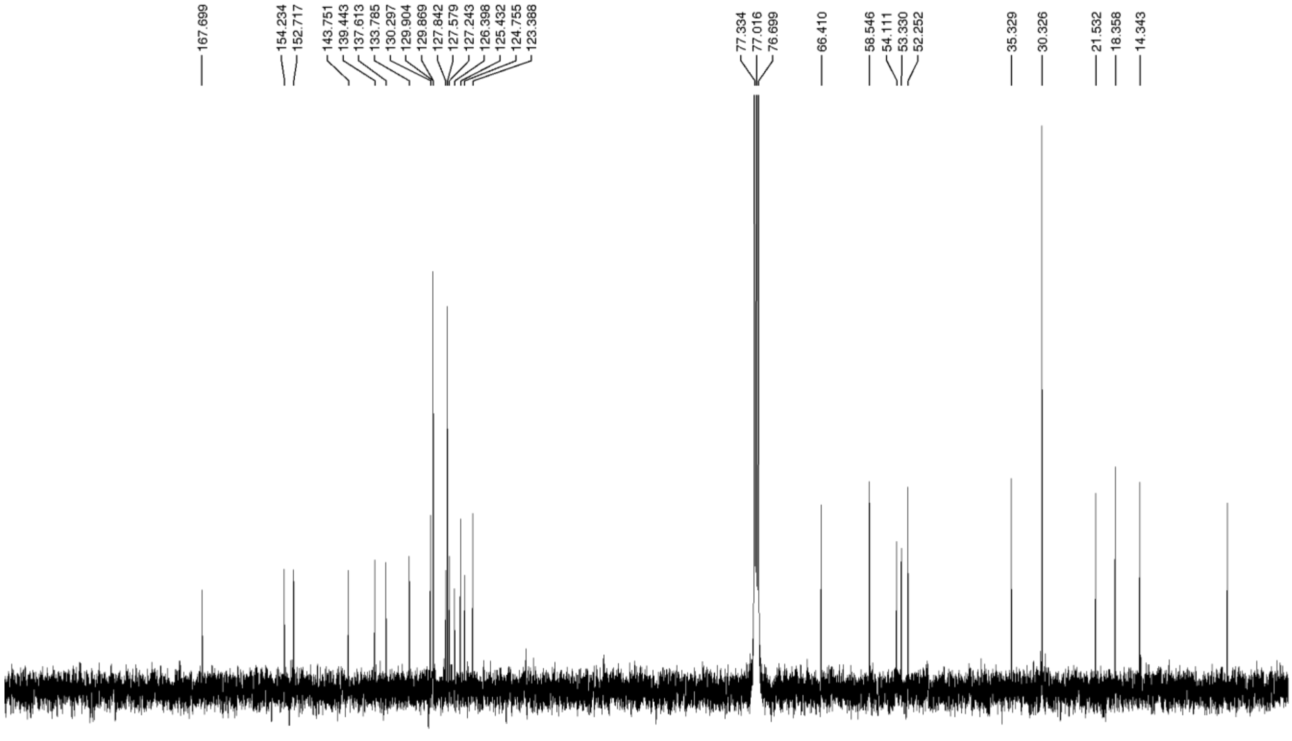

HMBC ($CDCl_3$, 400 MHz)

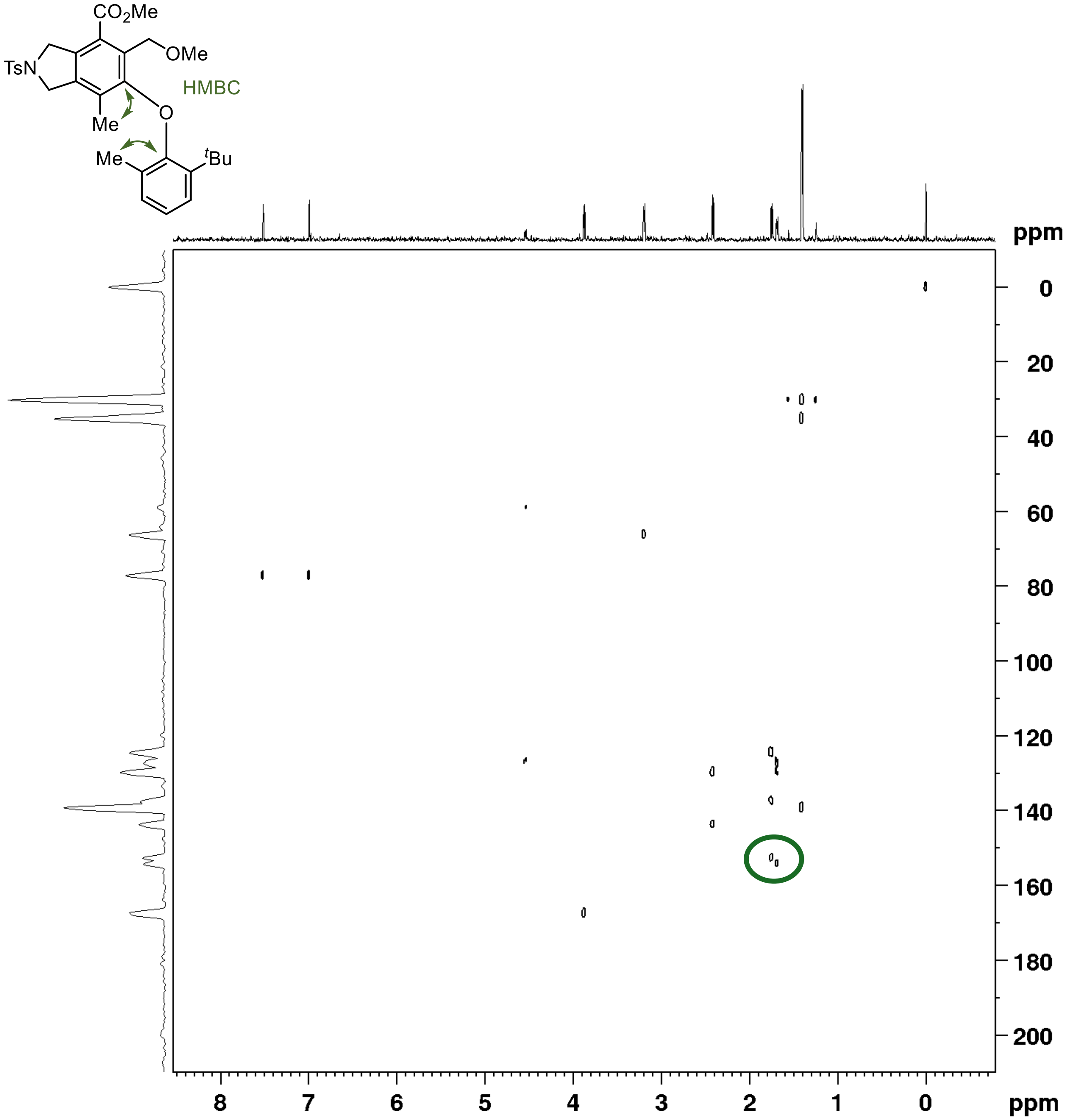

(–)-Methyl 5-(2-(*tert*-butyl)-6-methylphenoxy)-6-(methoxymethyl)-7-methyl-2-tosylisoindoline-4-carboxylate [(–)-4ap]

^{1}H NMR ($CDCl_3$, 400 MHz)

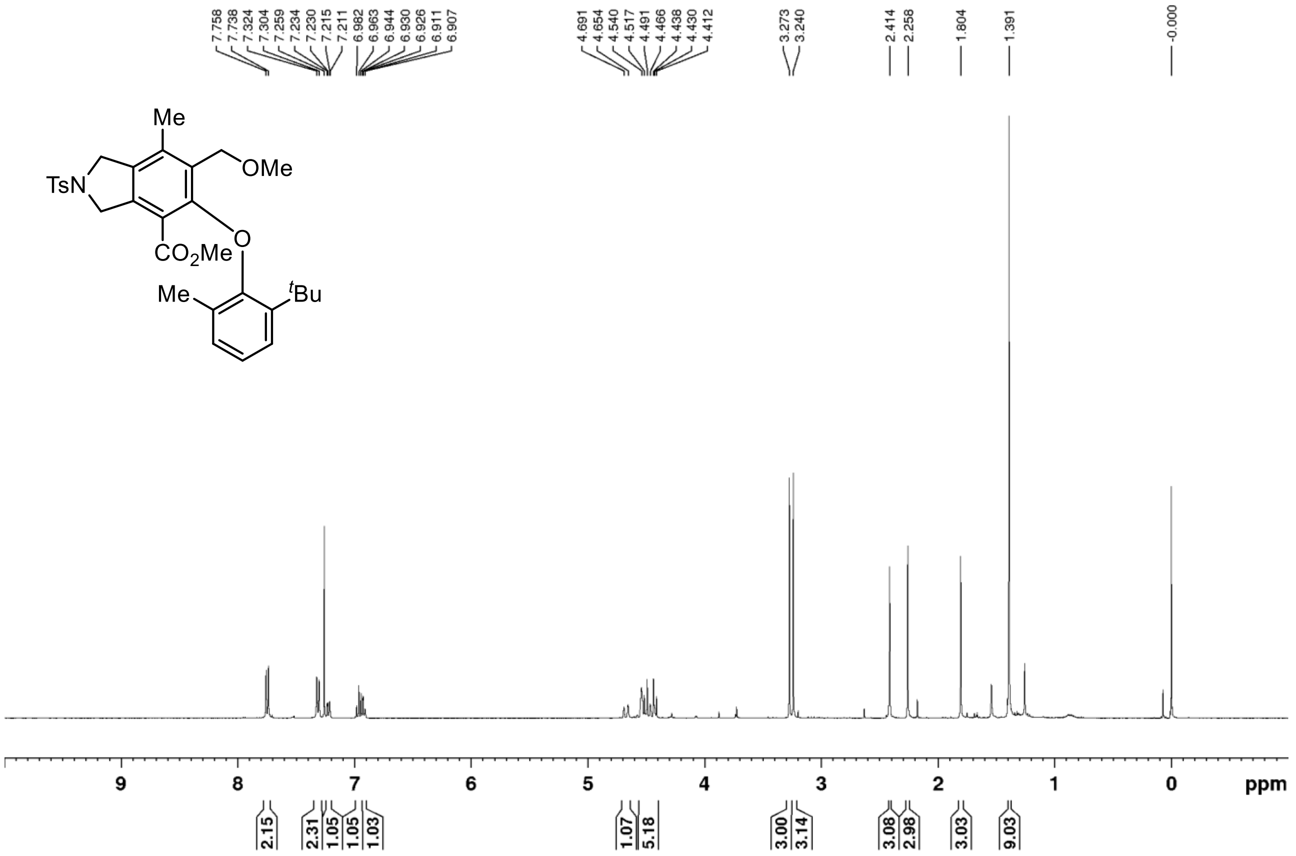

^{13}C NMR ($CDCl_3$, 101 MHz)

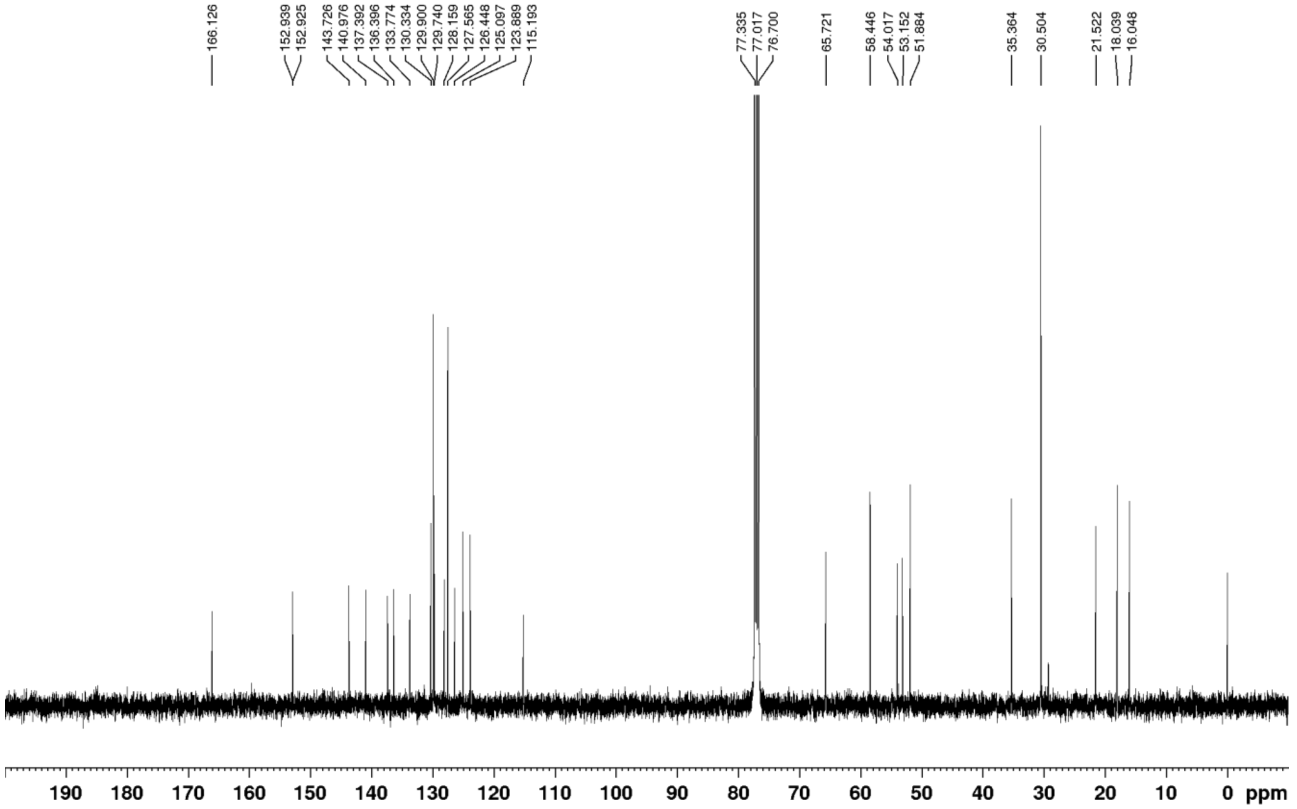

HMBC ($CDCl_3$, 400 MHz)

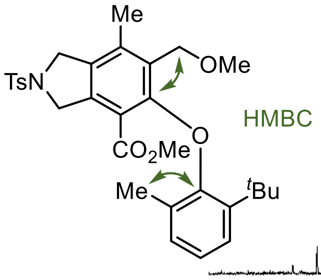

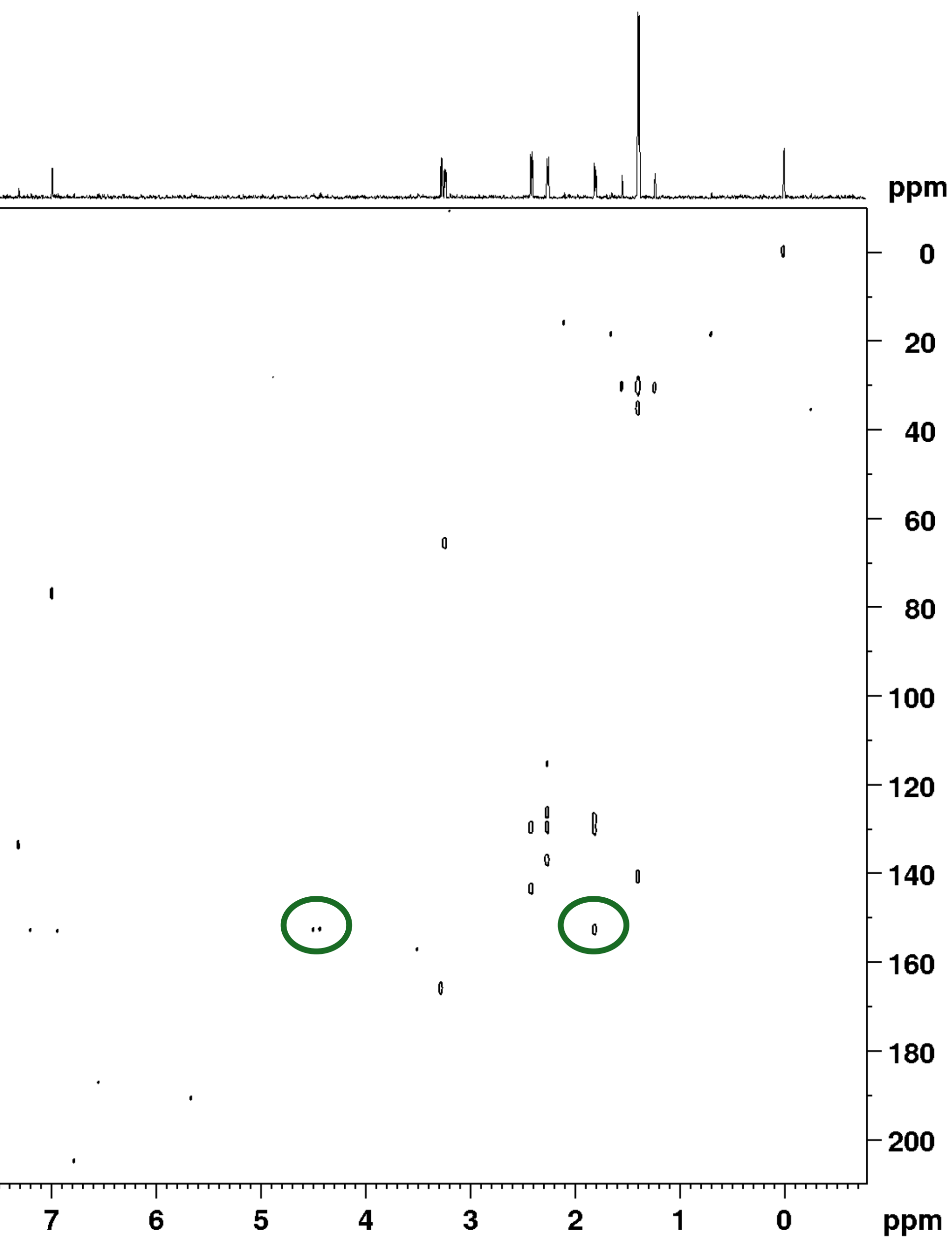

Methyl 6-(2-(*tert*-butyl)-6-methylphenoxy)-5,7-dimethyl-2-tosylisoindoline-4-carboxylate (3aq) and methyl 6-(2-(*tert*-butyl)-6-methylphenoxy)-5,7-dimethyl-2-tosylisoindoline-4-carboxylate (4aq)

^{1}H NMR ($CDCl_3$, 400 MHz)

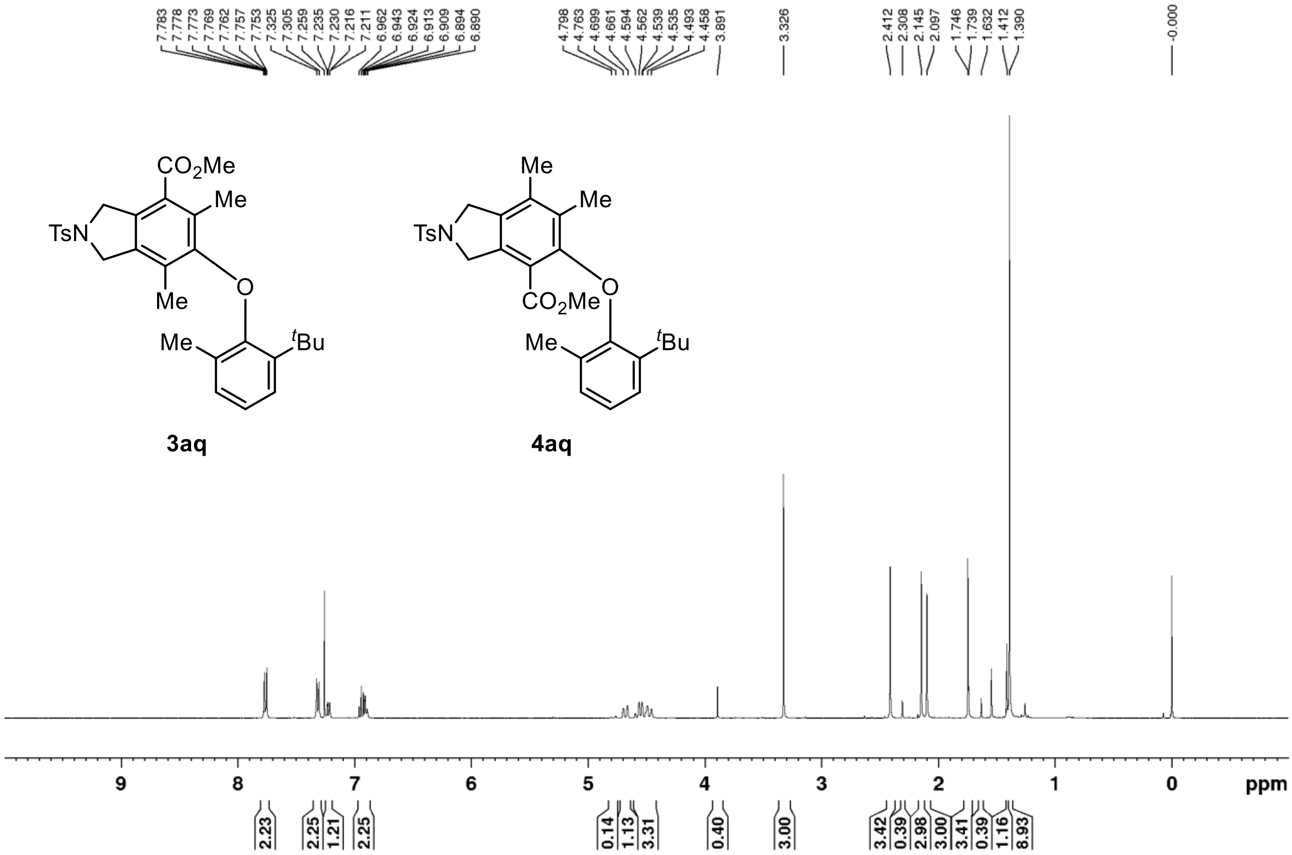

^{13}C NMR ($CDCl_3$, 101 MHz)

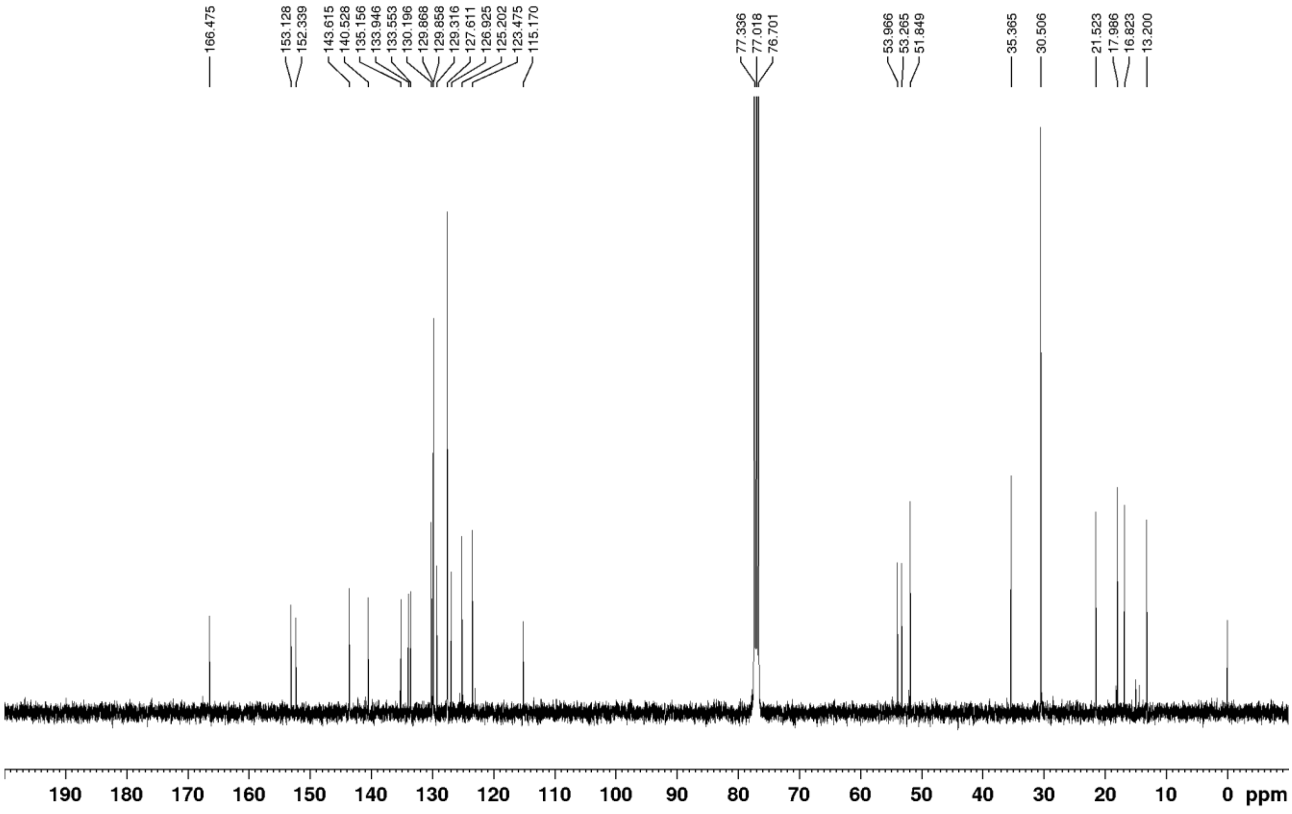

HMBC ($CDCl_3$, 400 MHz)

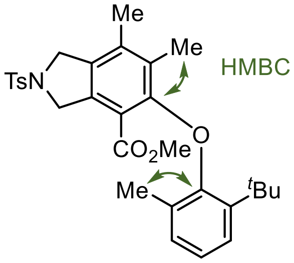

4aq

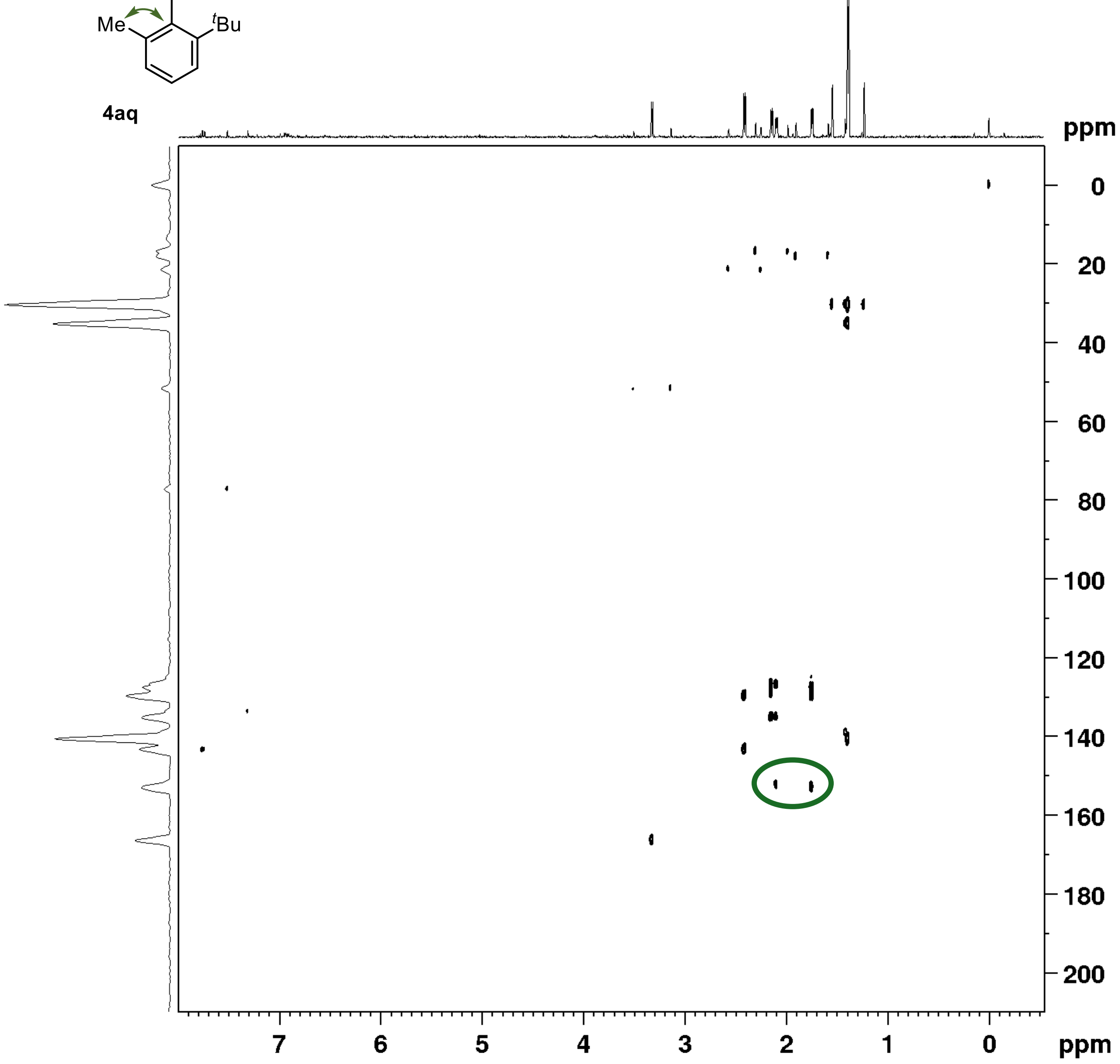

(+)-Dimethyl 6-(2-(*tert*-butyl)-6-methylphenoxy)-7-methyl-1,3-dihydroisobenzofuran-4,5-dicarboxylate [(+)-3ea]

^{1}H NMR ($CDCl_3$, 400 MHz)

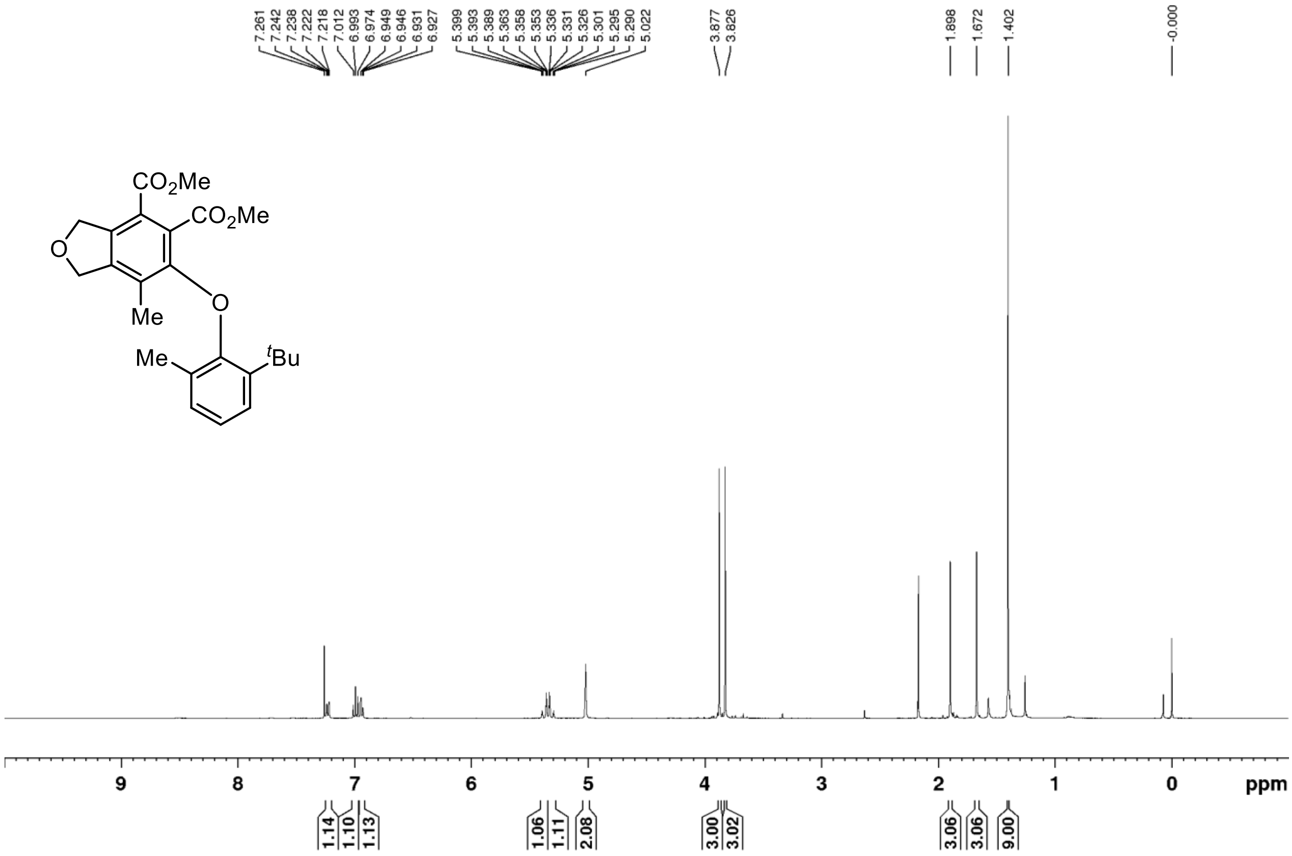

^{13}C NMR ($CDCl_3$, 101 MHz)

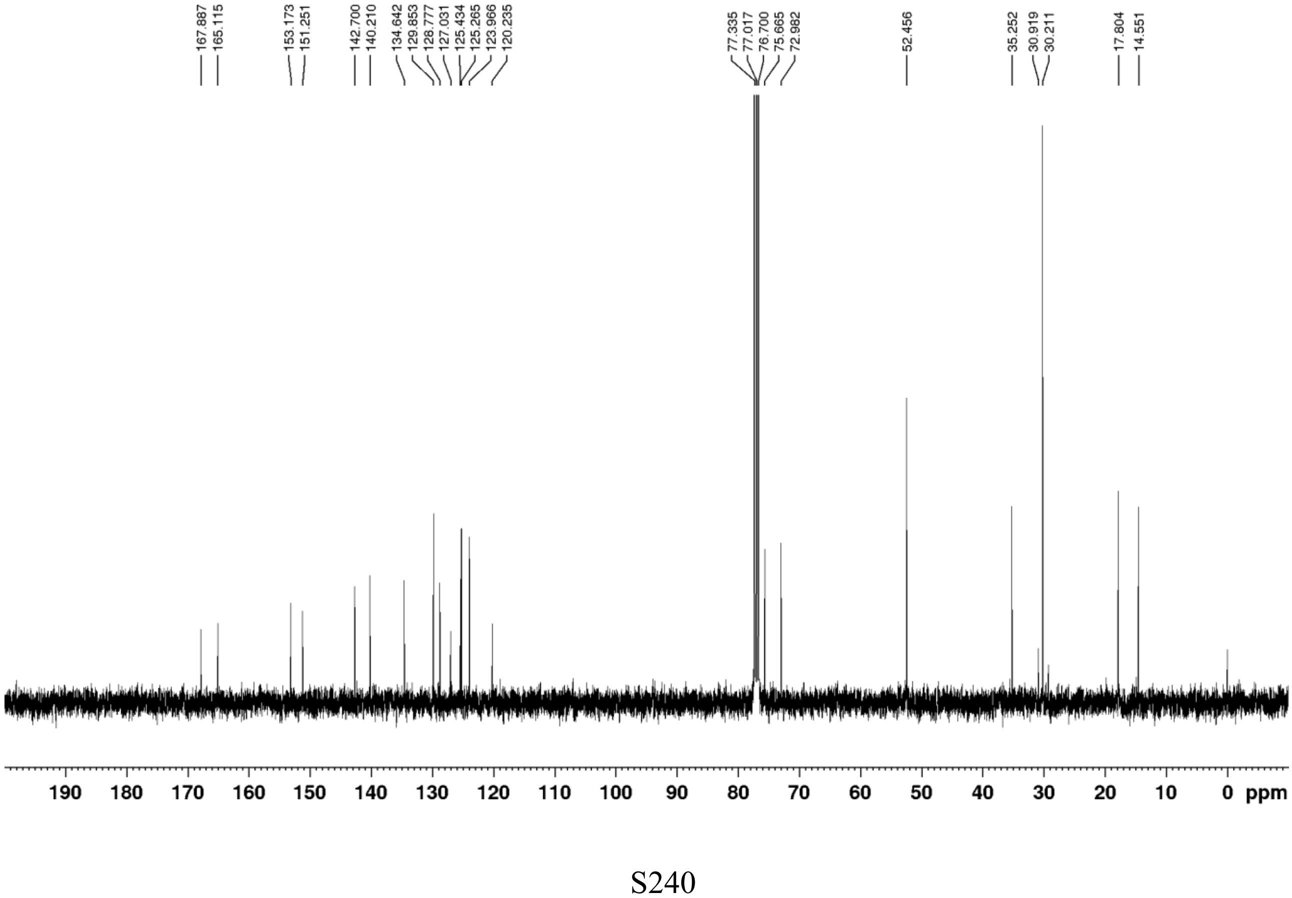

HMBC ($CDCl_3$, 400 MHz)

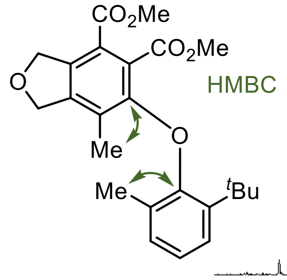

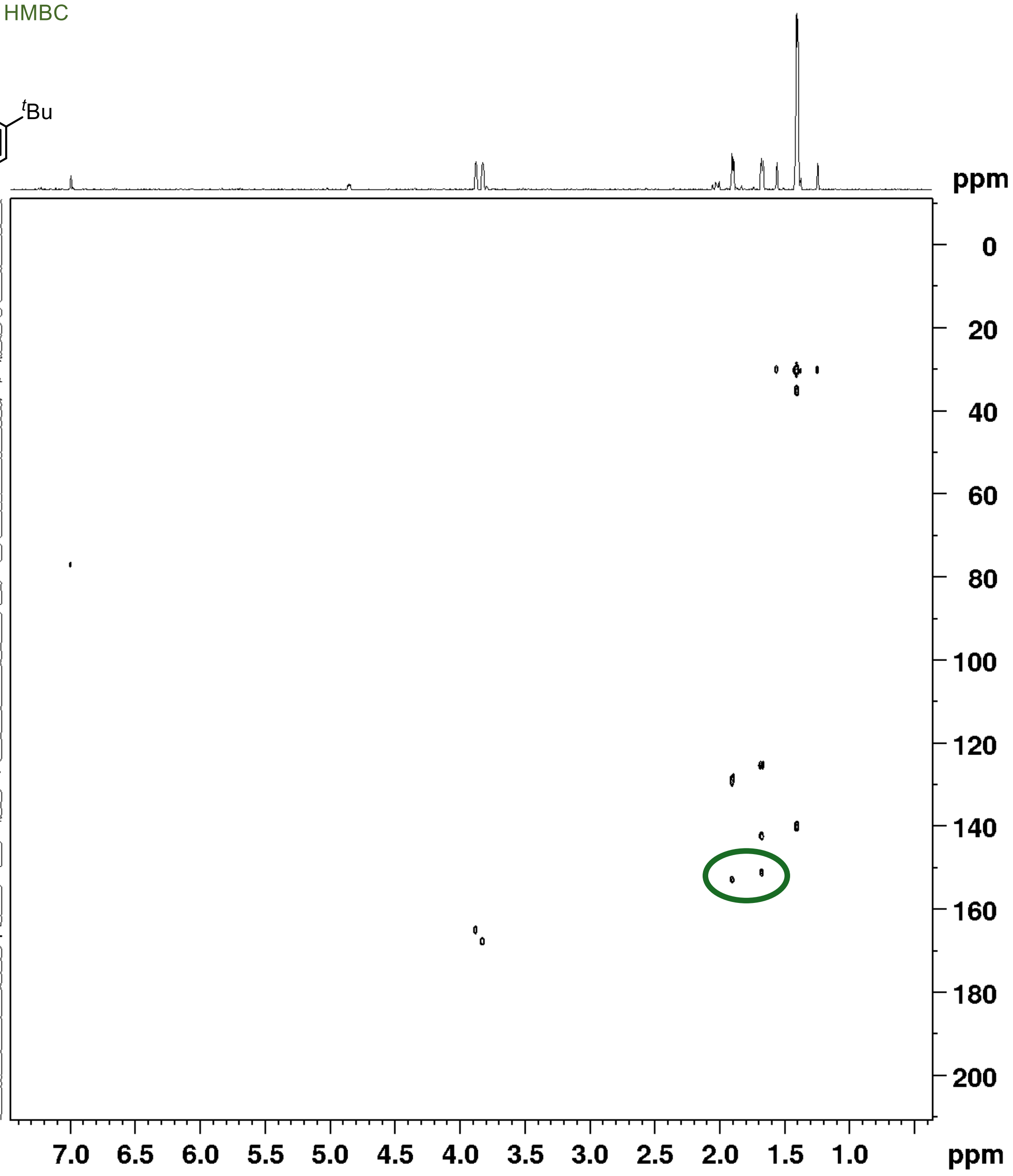

(+)-Dimethyl 5-(2-(*tert*-butyl)-6-methylphenoxy)-7-methyl-1,3-dihydroisobenzofuran-4,6-dicarboxylate [(+)-4ea]

^{1}H NMR ($CDCl_3$, 400 MHz)

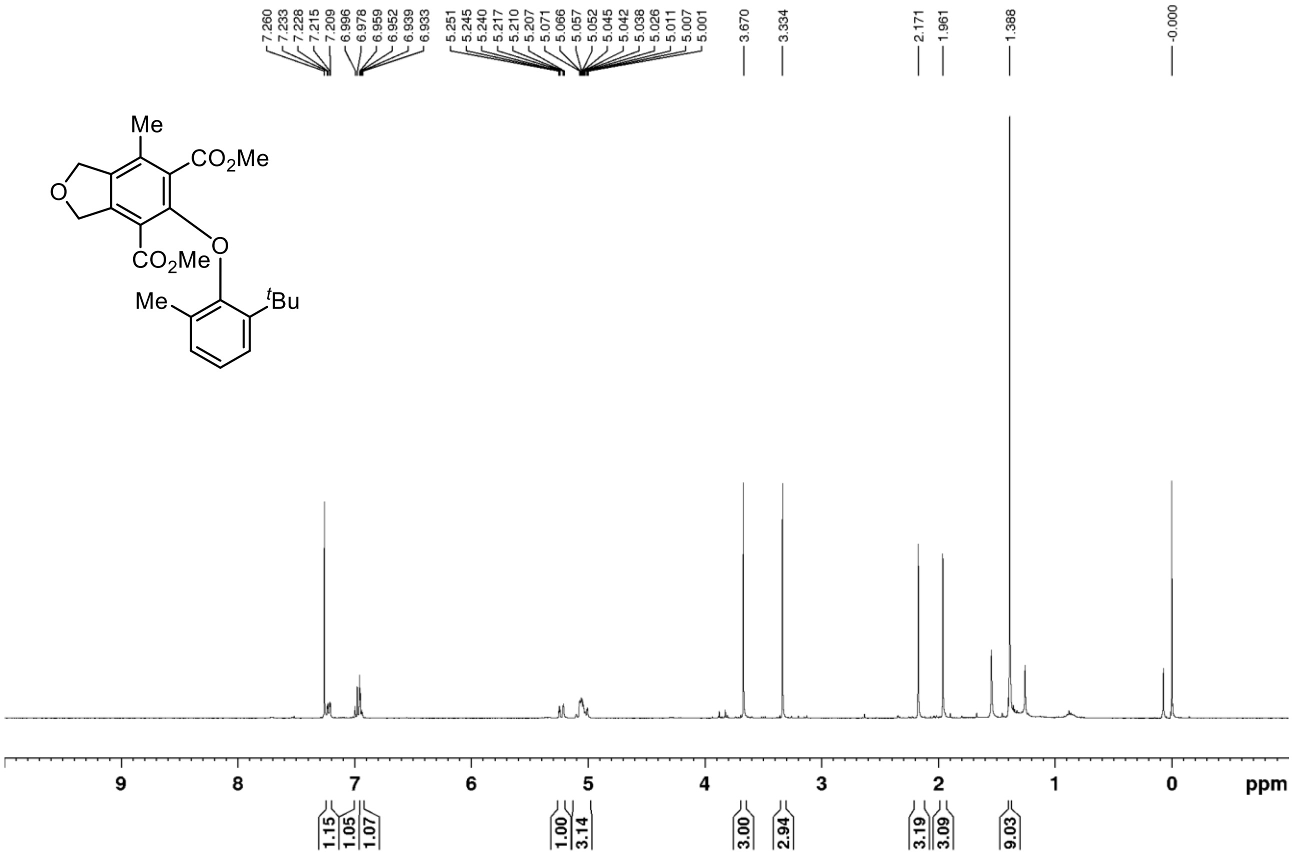

^{13}C NMR ($CDCl_3$, 101 MHz)

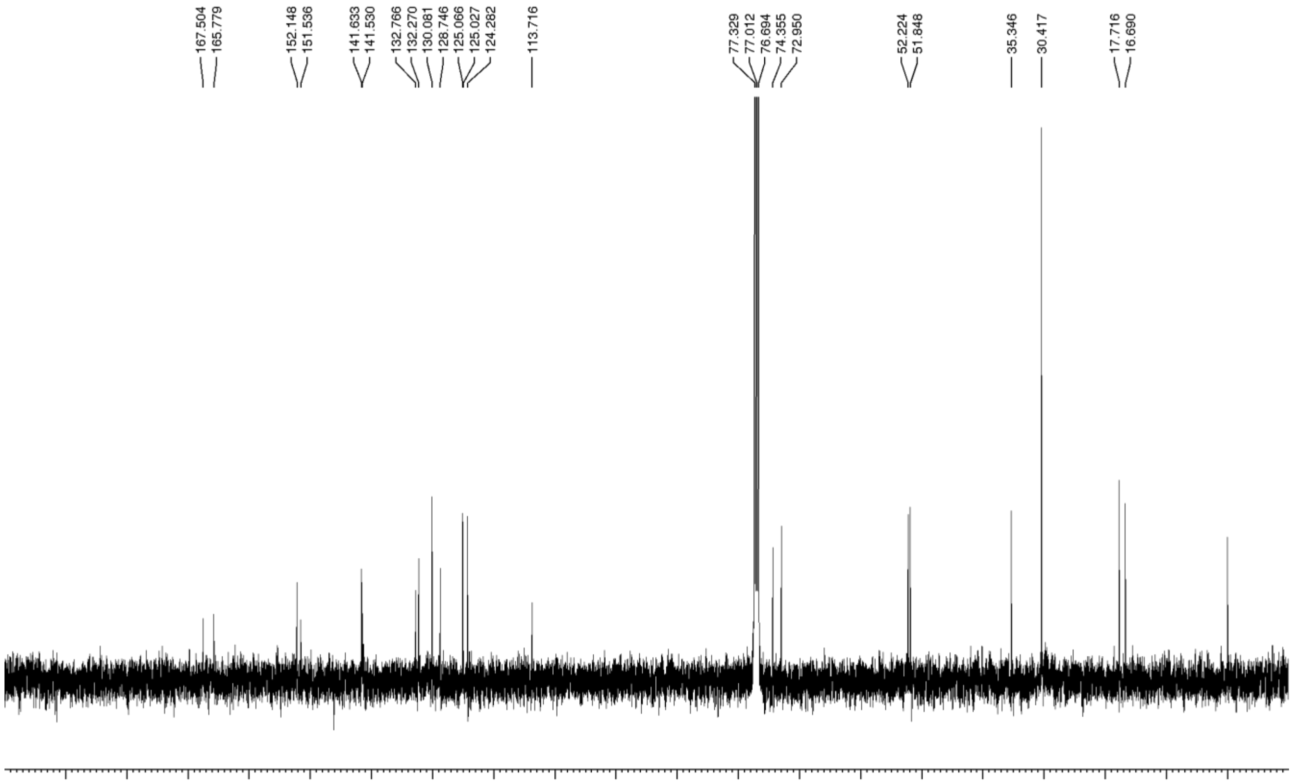

NOESY (CDCl3, 400 MHz)

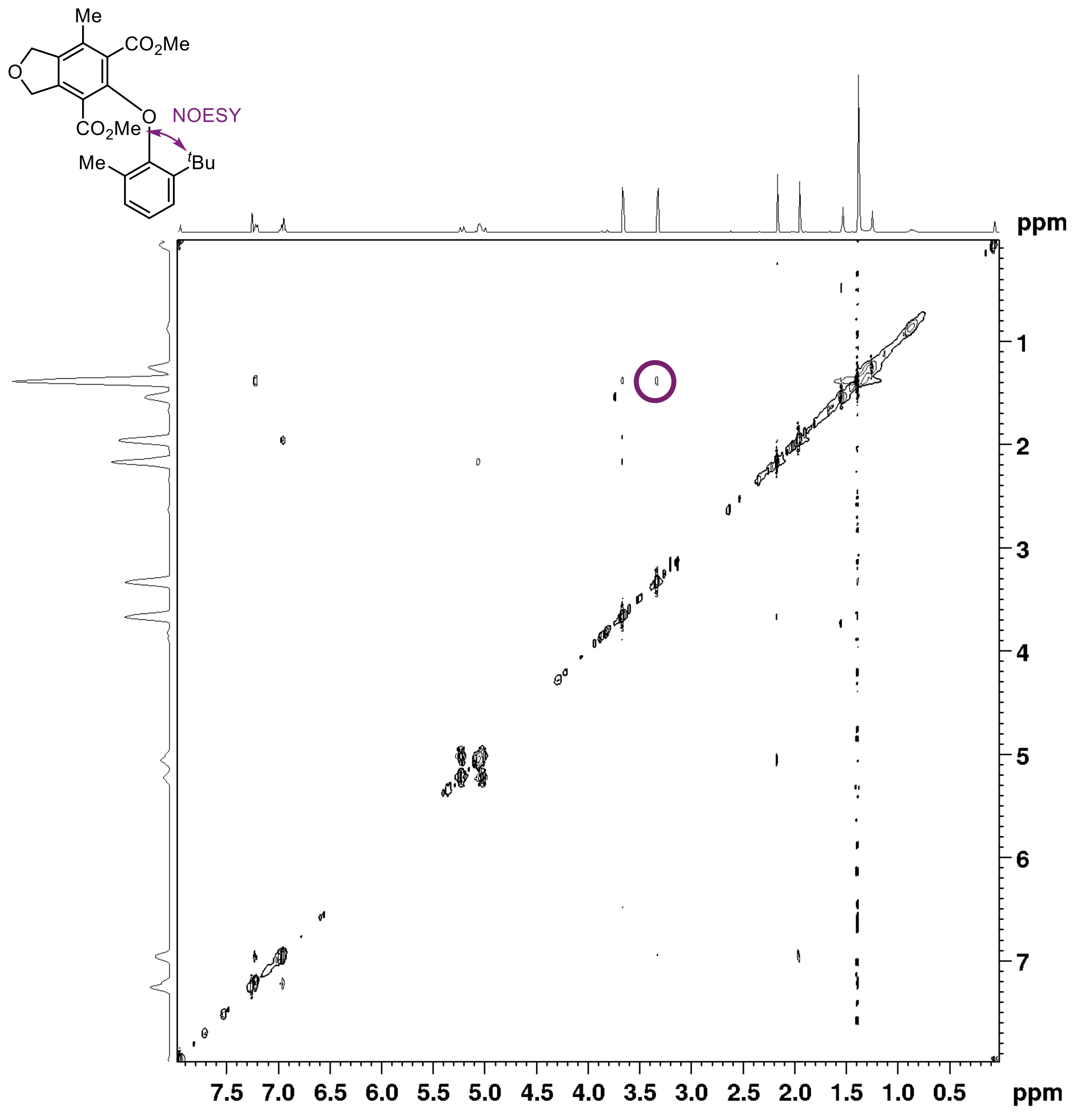

7. Chiral HPLC Charts

The compound number, the name of the chiral column, and the solvent added to *n*-hexane are shown.

3aa

IE-3 / 2-PrOH 20%

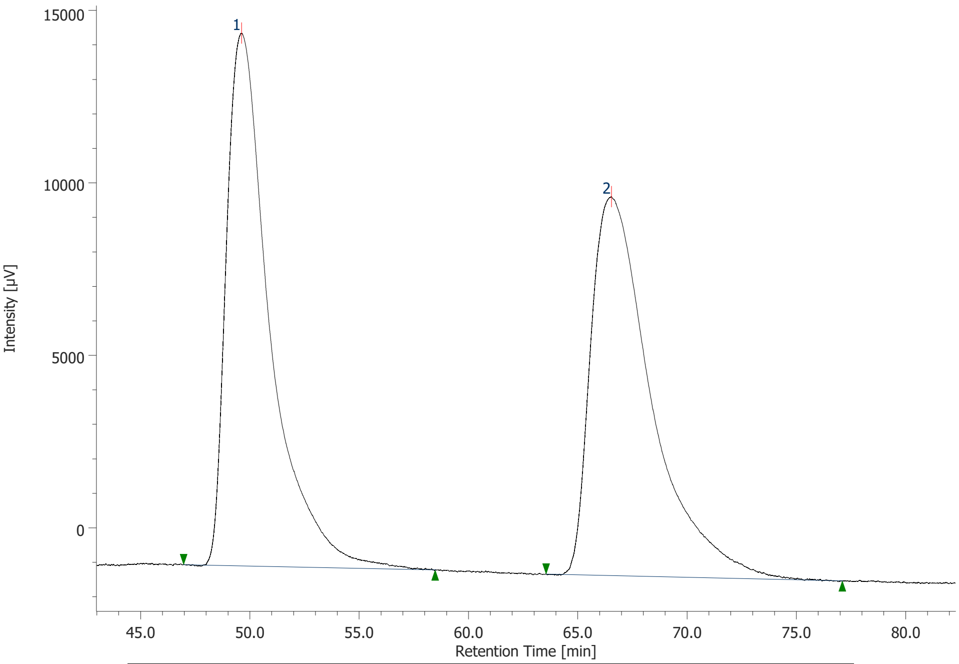

Peak No.	Retention Time (min)	Area (%)
1	49.617	50.262
2	66.533	49.738

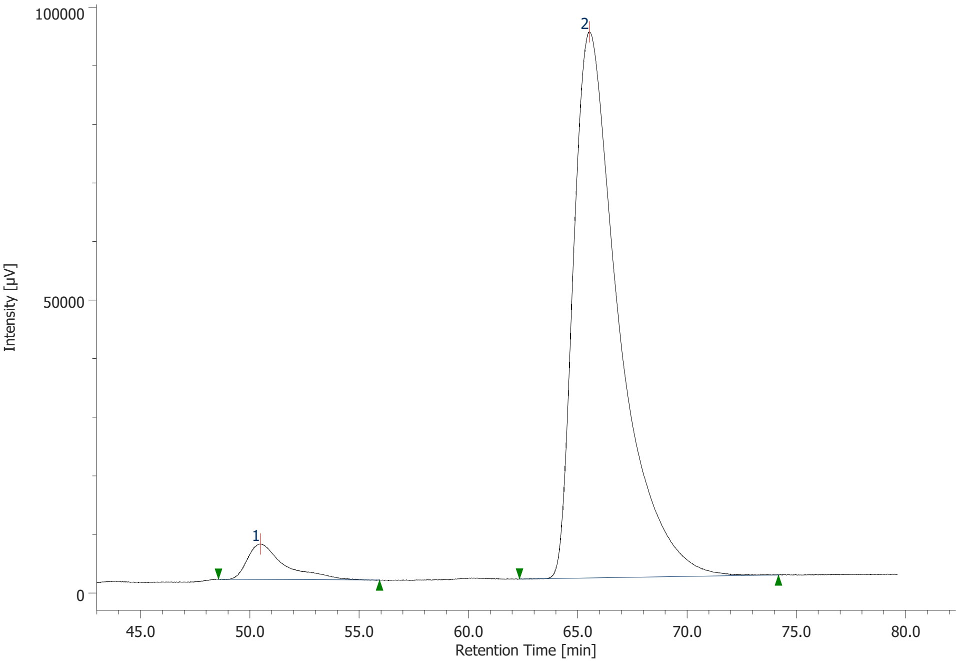

Peak No.	Retention Time (min)	Area (%)
1	50.500	5.156
2	65.542	94.844

4aa

IF-3 / 2-PrOH 20%

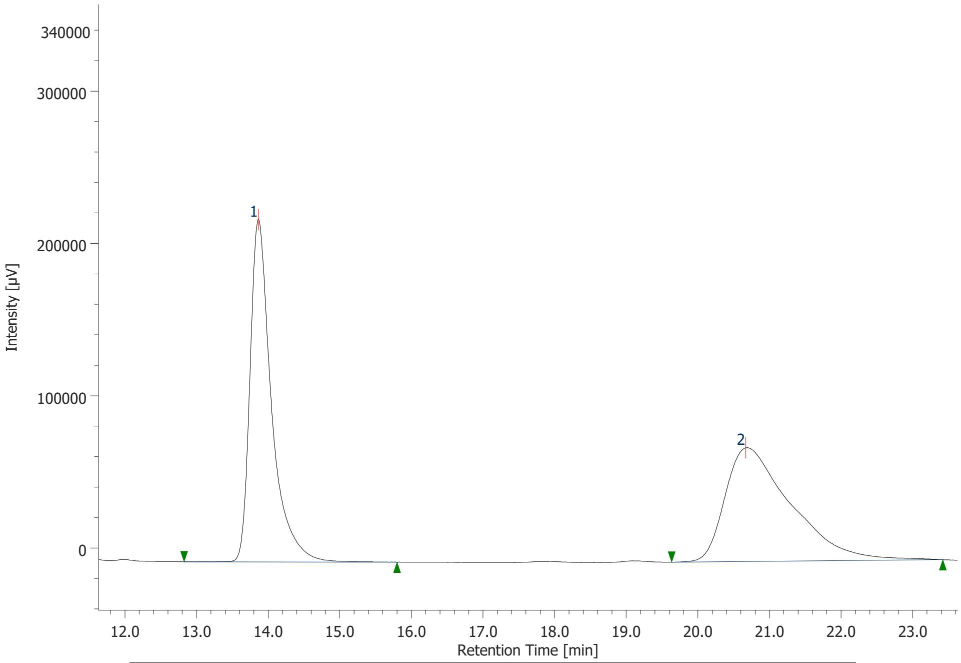

Peak No.	Retention Time (min)	Area (%)
1	13.867	49.685
2	20.667	50.315

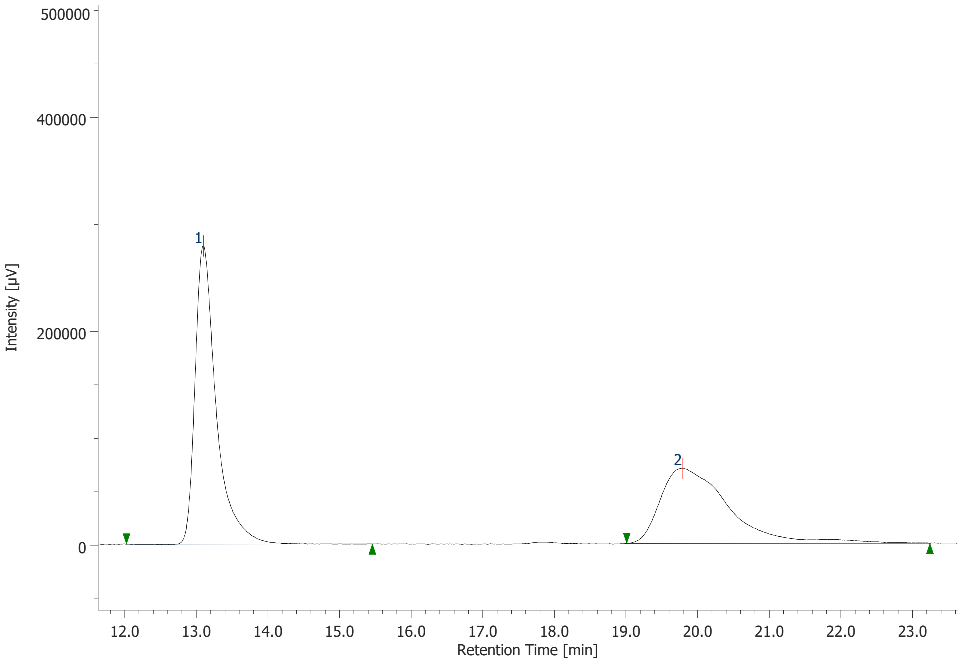

Peak No.	Retention Time (min)	Area (%)
1	13.100	55.219
2	19.792	44.781

3ab

IG-3 / 2-PrOH 15%

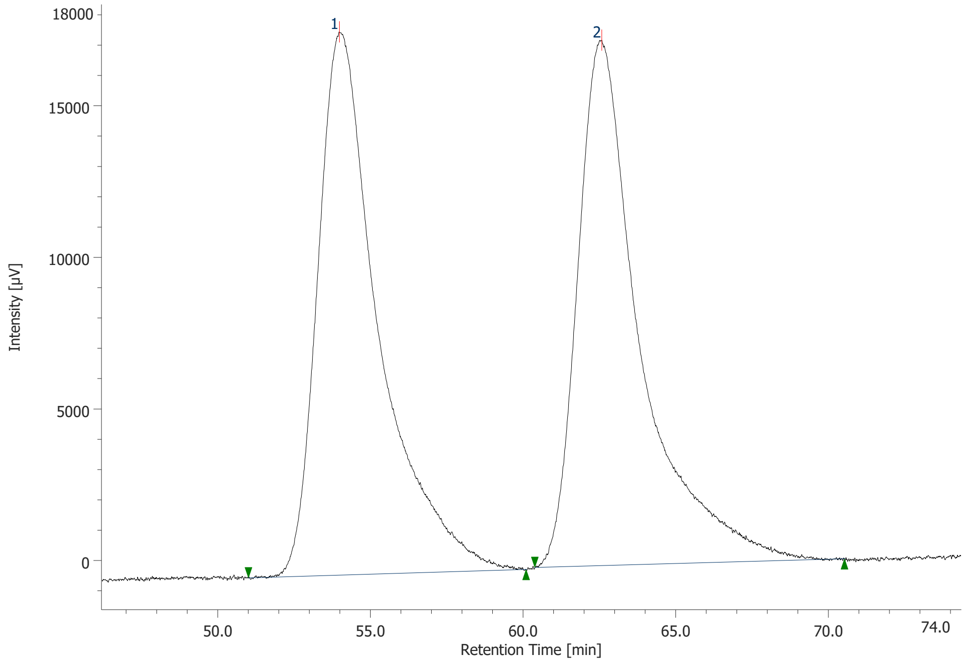

Peak No.	Retention Time (min)	Area (%)
1	53.983	50.171
2	62.575	49.829

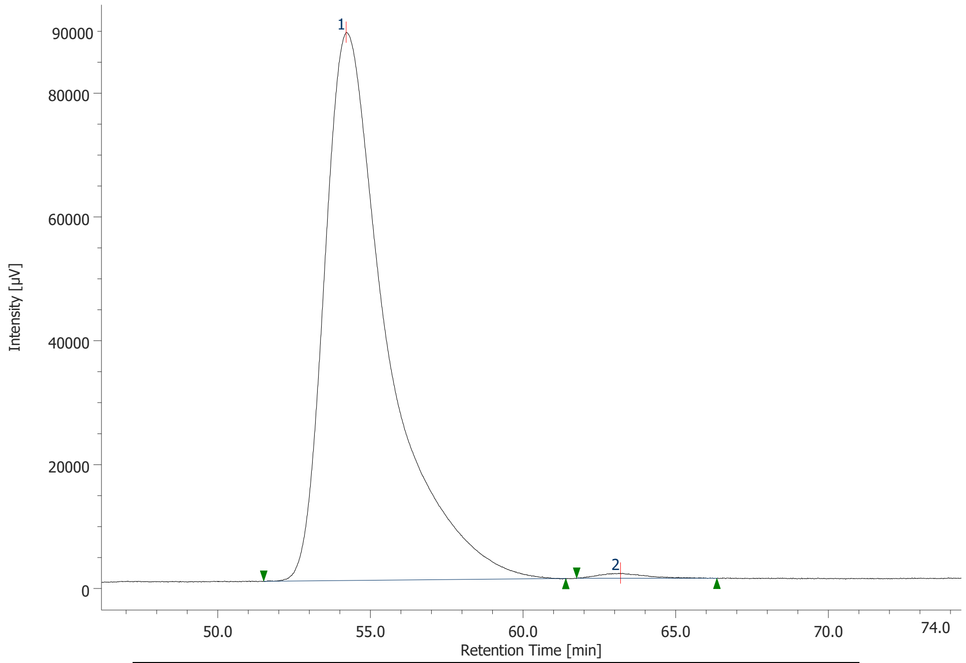

Peak No.	Retention Time (min)	Area (%)
1	54.208	99.329
2	63.192	0.671

4ab

IF-3 / 2-PrOH 15%

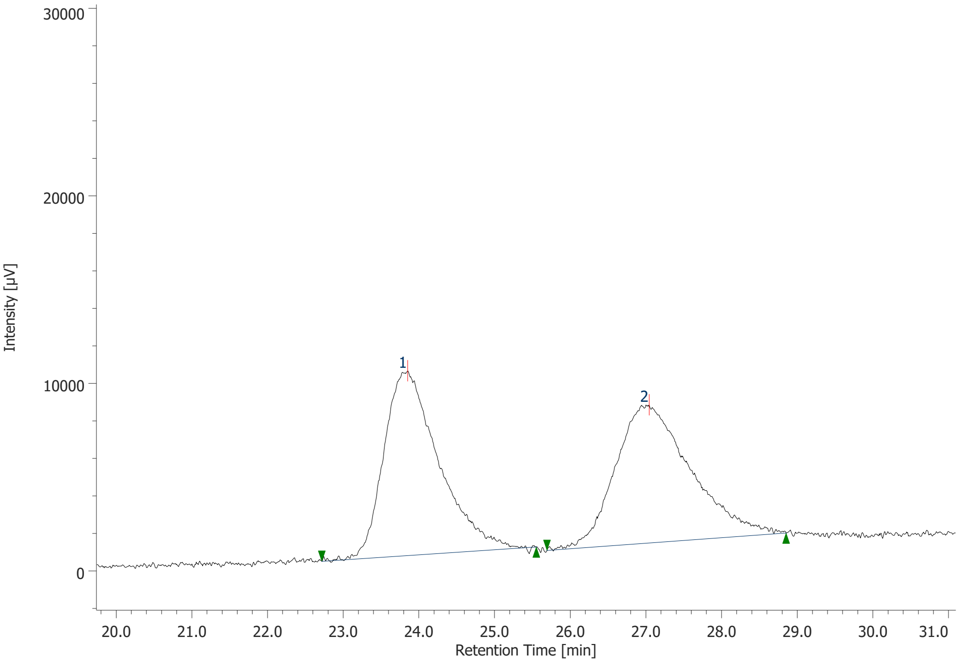

Peak No.	Retention Time (min)	Area (%)
1	23.850	49.407
2	27.042	50.593

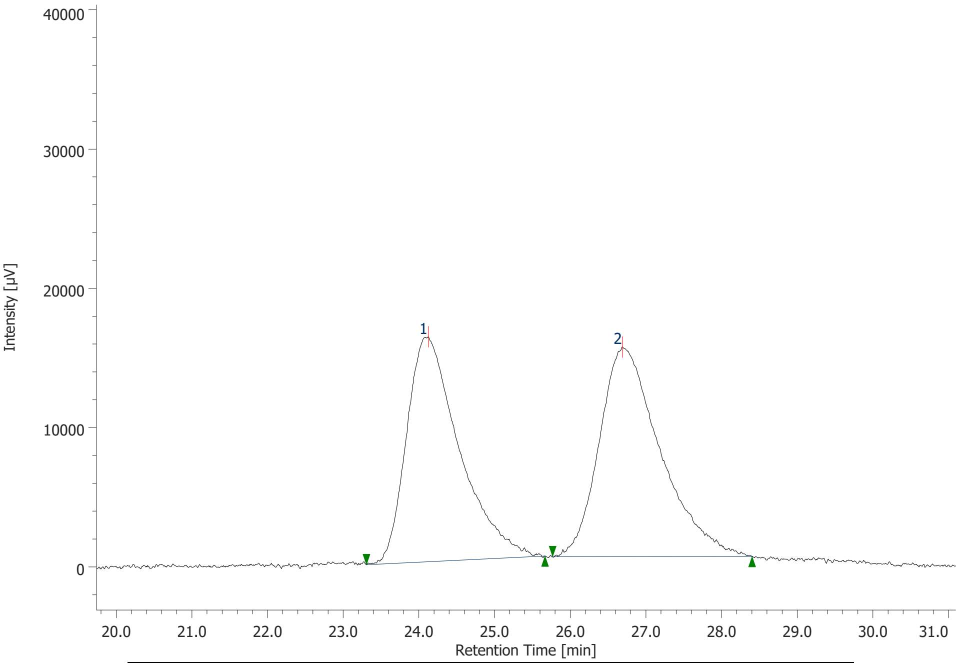

Peak No.	Retention Time (min)	Area (%)
1	24.125	48.104
2	26.692	51.896

3ac

ID-3 / 2-PrOH 20%

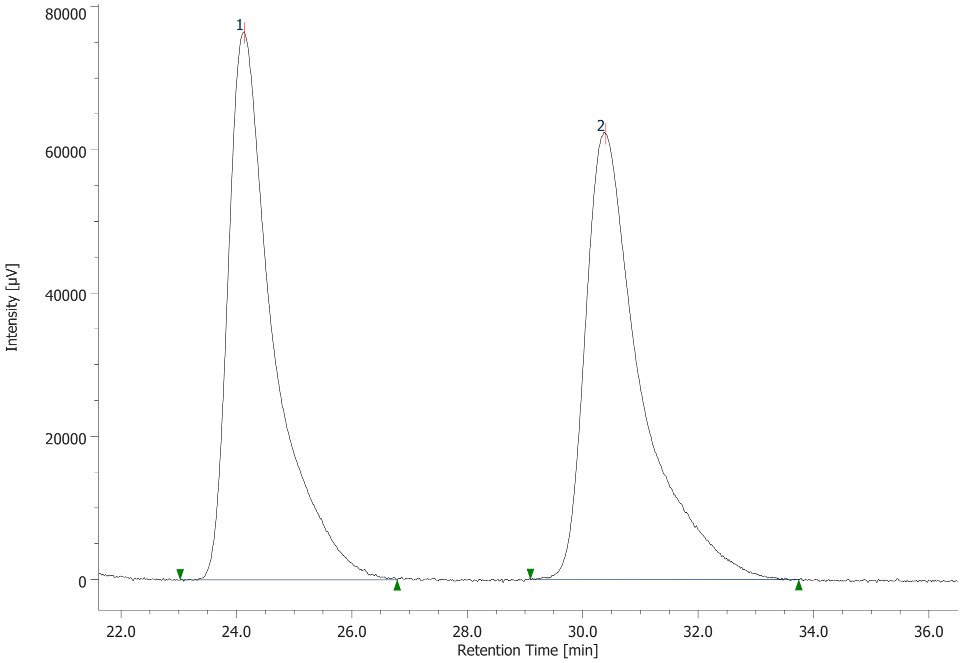

Peak No.	Retention Time (min)	Area (%)
1	24.142	50.016
2	30.400	49.984

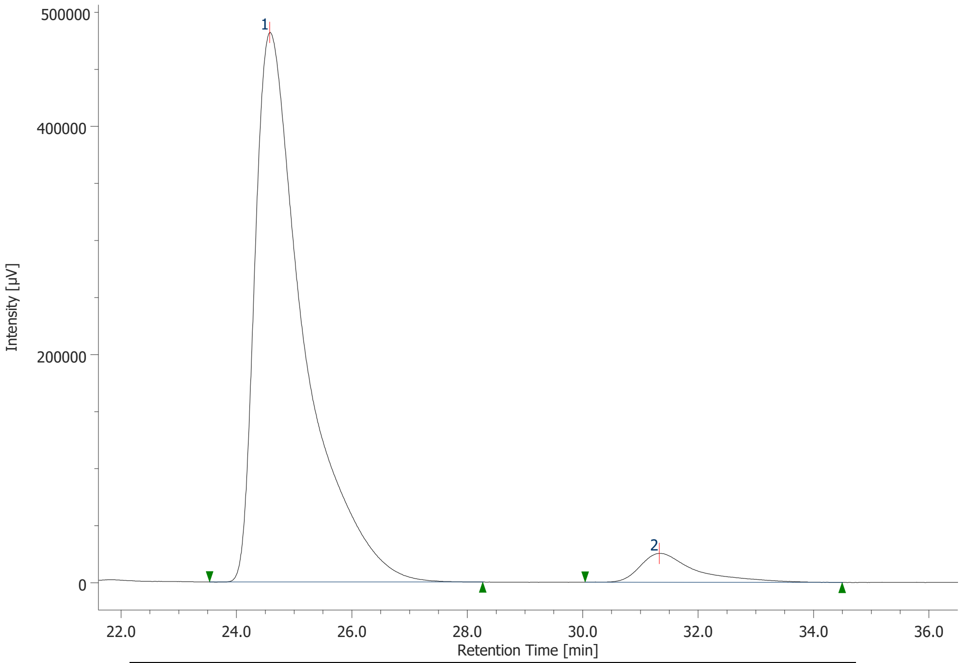

Peak No.	Retention Time (min)	Area (%)
1	24.575	94.395
2	31.325	5.605

4ac

IE-3 / 2-PrOH 20%

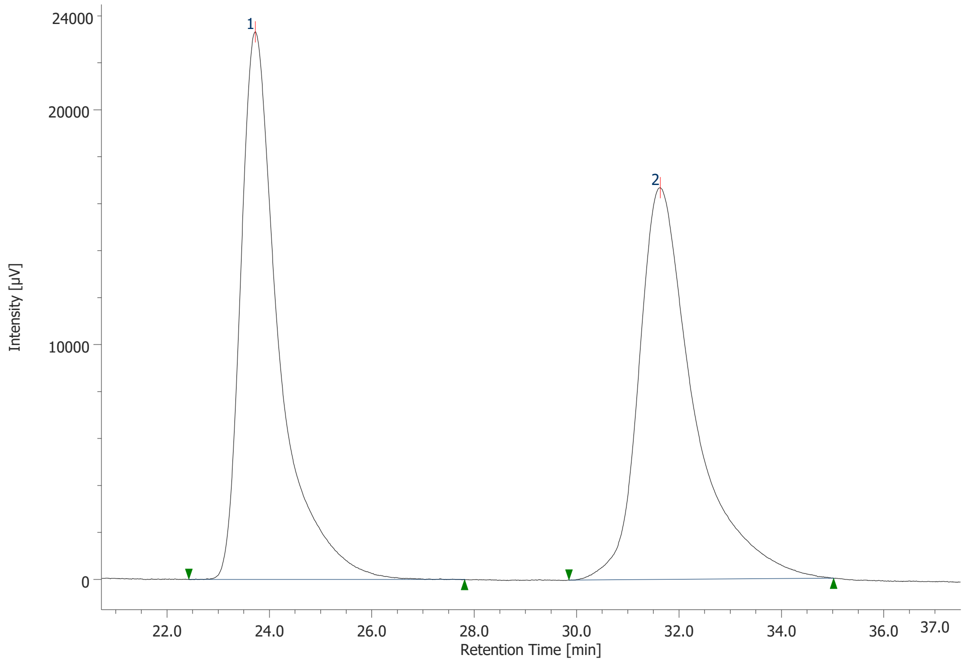

Peak No.	Retention Time (min)	Area (%)
1	23.725	49.309
2	31.633	50.691

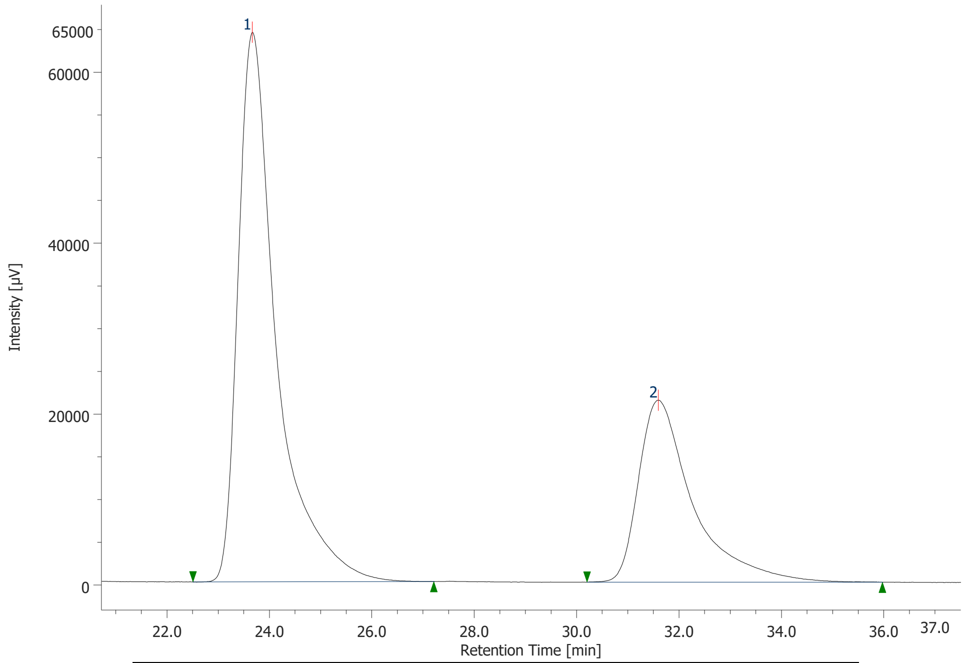

Peak No.	Retention Time (min)	Area (%)
1	23.667	68.107
2	31.592	31.893

3ad

IF-3 / 2-PrOH 10%

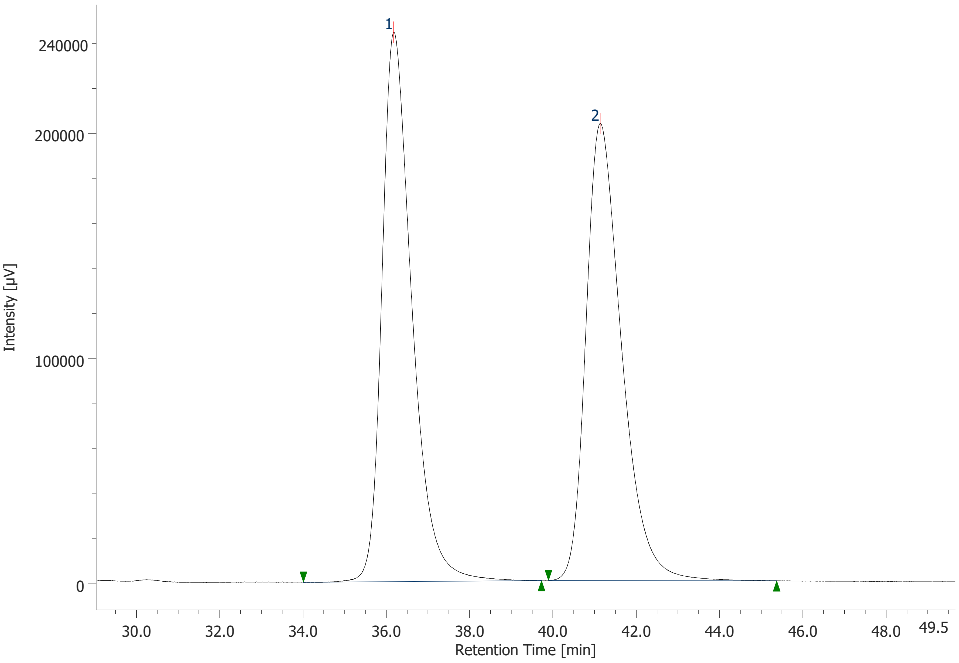

Peak No.	Retention Time (min)	Area (%)
1	36.175	50.310
2	41.133	49.690

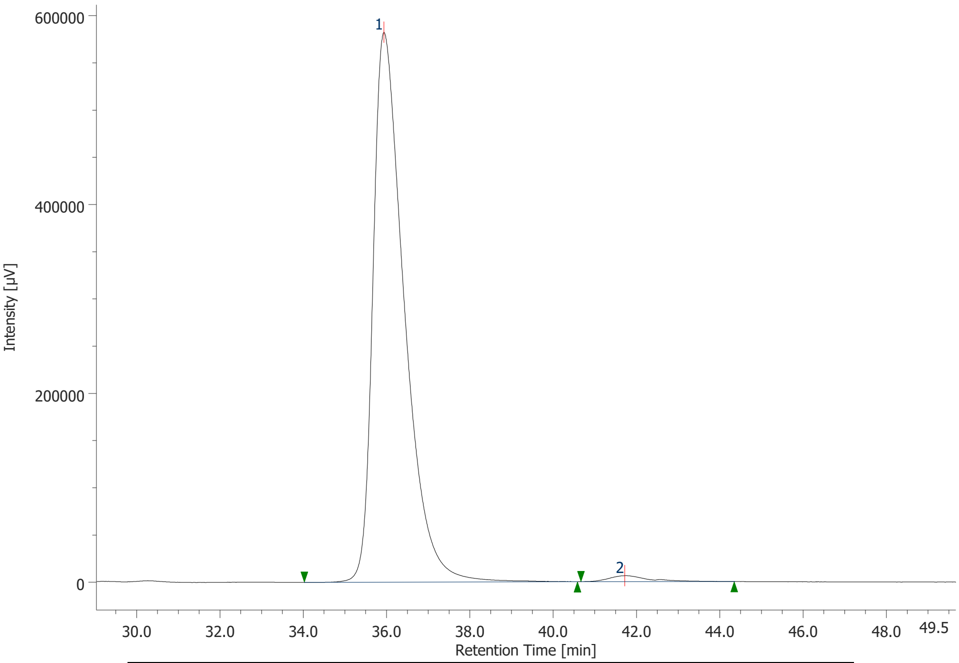

Peak No.	Retention Time (min)	Area (%)
1	35.933	98.648
2	41.717	1.352

3ae

IG-3 / 2-PrOH 15%

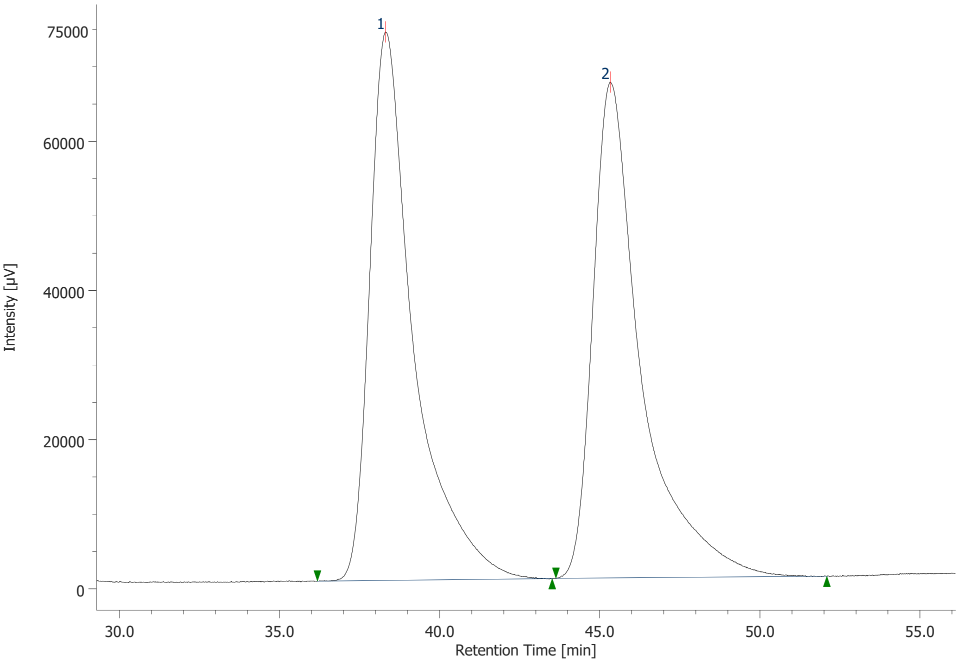

Peak No.	Retention Time (min)	Area (%)
1	38.308	50.221
2	45.325	49.779

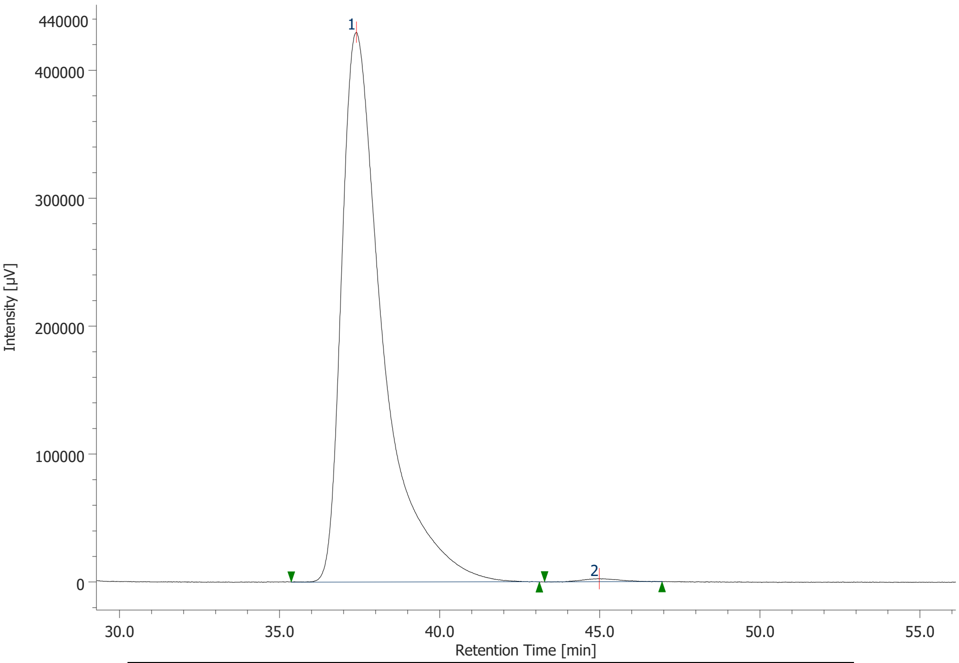

Peak No.	Retention Time (min)	Area (%)
1	37.400	99.484
2	44.983	0.516

3af

IE-3 / 2-PrOH 20%

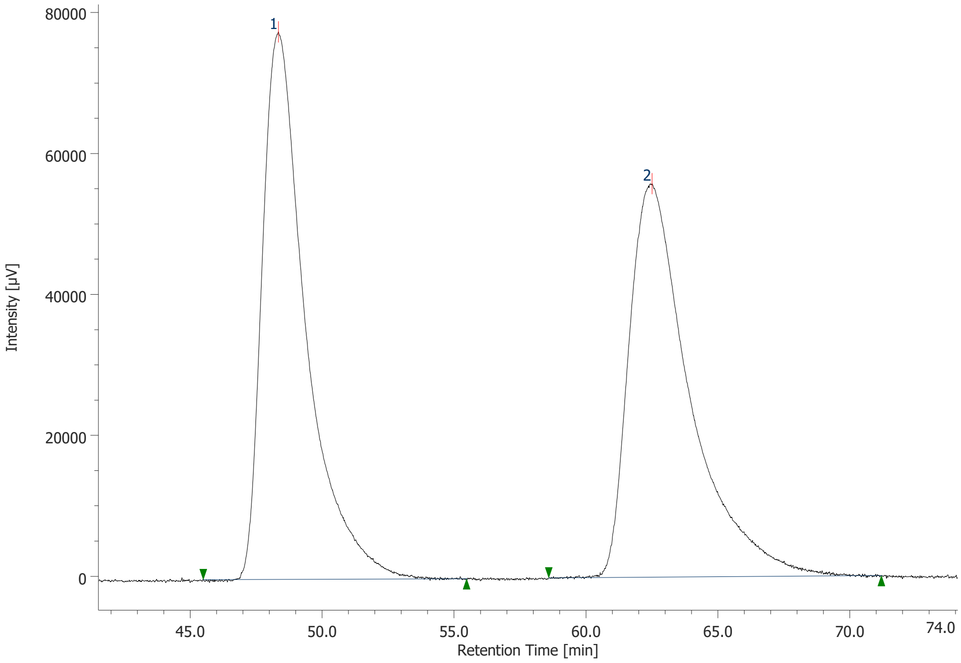

Peak No.	Retention Time (min)	Area (%)
1	48.342	49.882
2	62.500	50.118

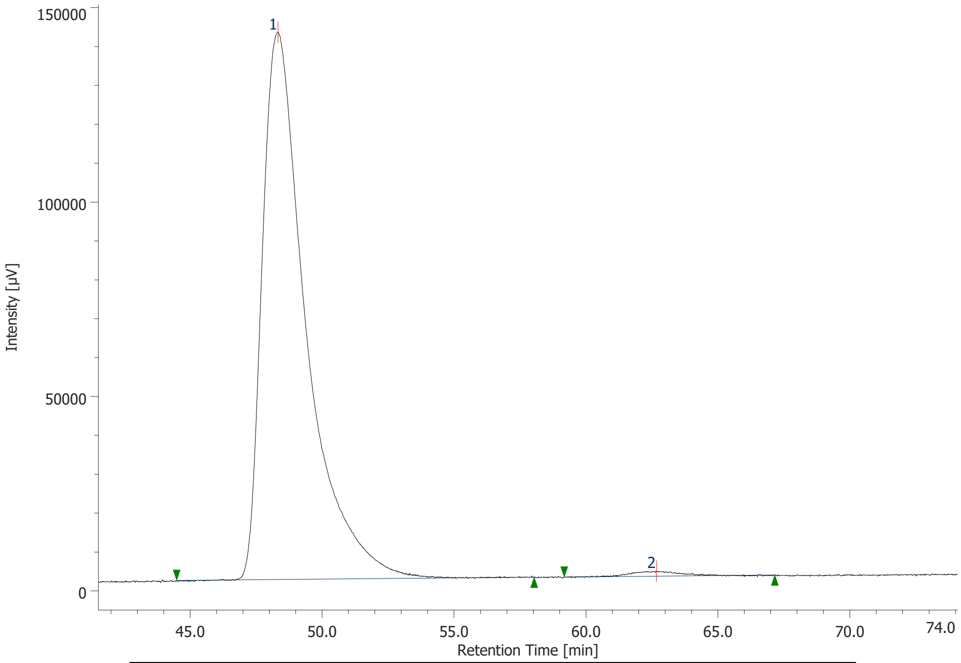

Peak No.	Retention Time (min)	Area (%)
1	48.325	98.982
2	62.667	1.018

3ag

IF-3 / 2-PrOH 20%

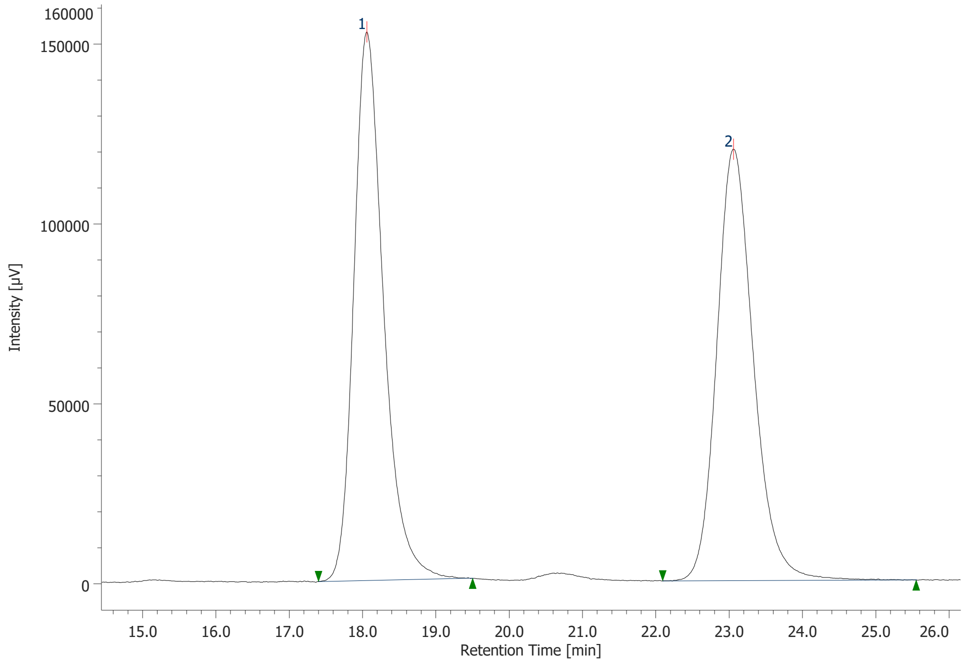

Peak No.	Retention Time (min)	Area (%)
1	18.058	50.769
2	23.058	49.231

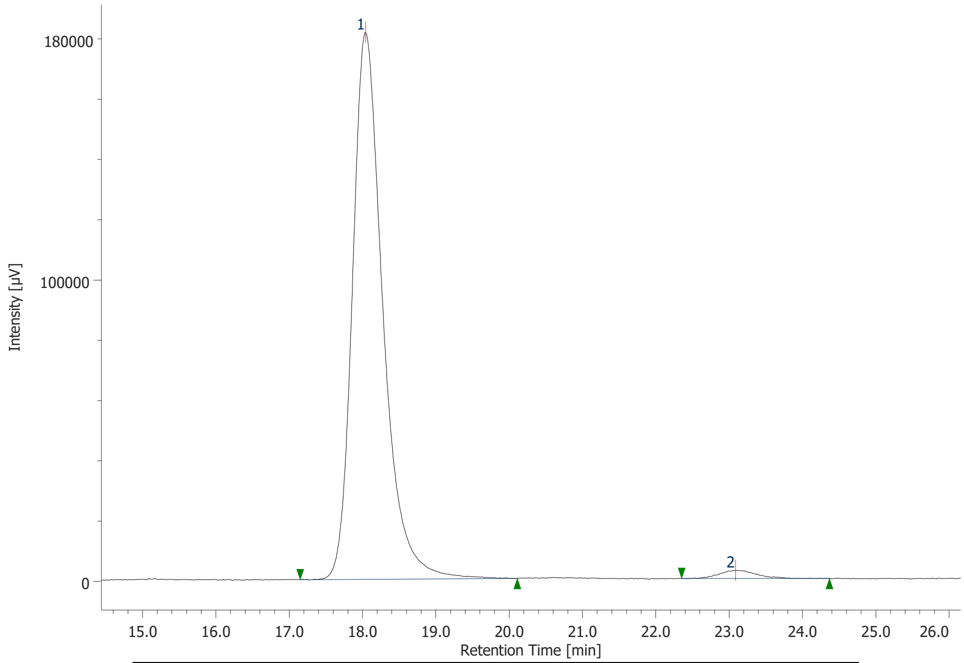

Peak No.	Retention Time (min)	Area (%)
1	18.042	98.181
2	23.083	1.819

3ah

IF-3 / 2-PrOH 20%

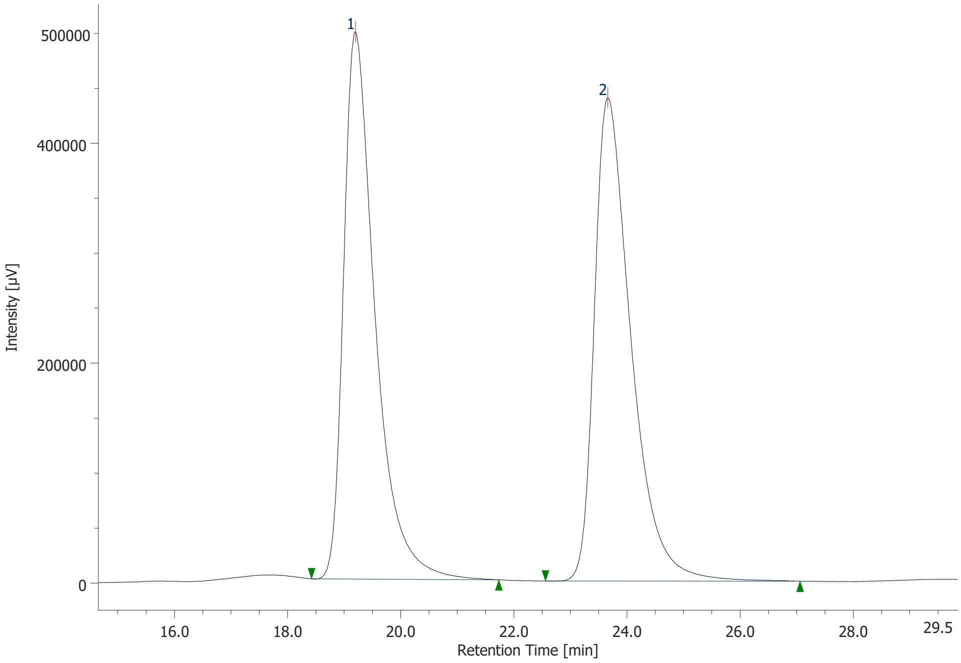

Peak No.	Retention Time (min)	Area (%)
1	19.200	49.329
2	23.658	50.671

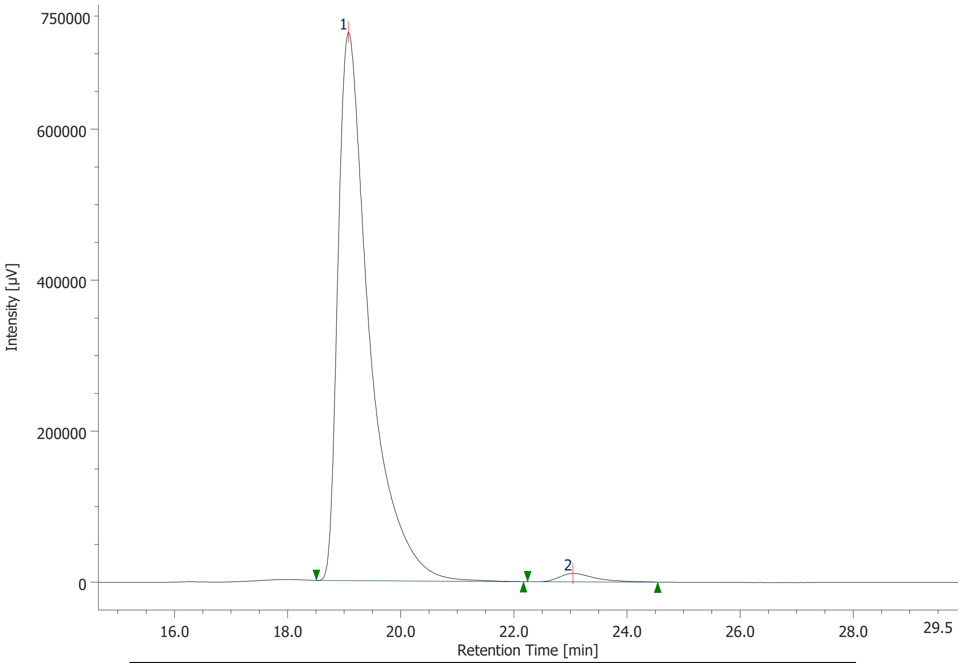

Peak No.	Retention Time (min)	Area (%)
1	19.075	98.295
2	23.042	1.705

3bb

ID-3 / 2-PrOH 20%

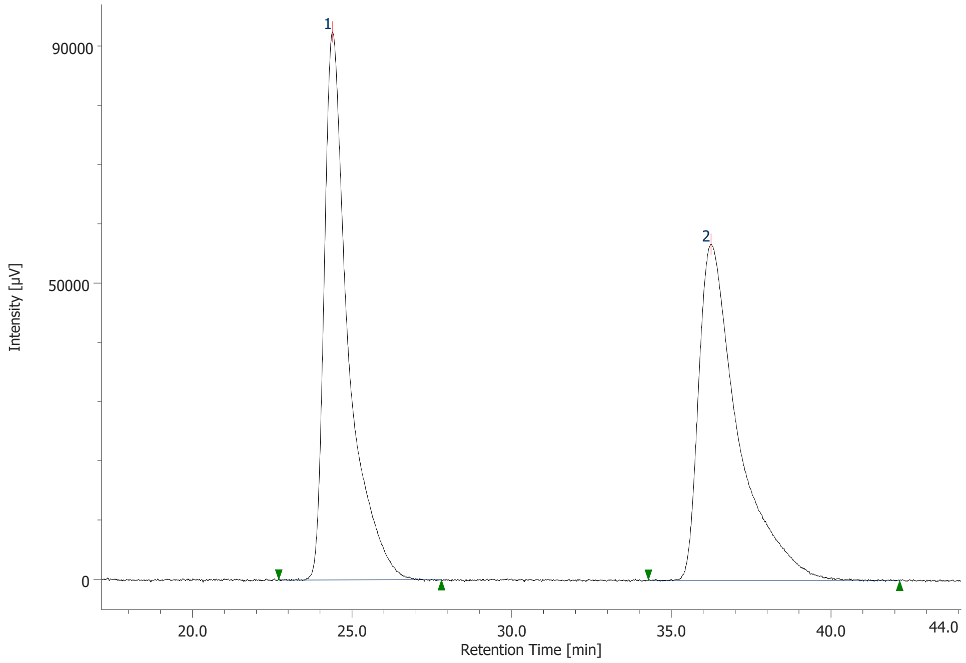

Peak No.	Retention Time (min)	Area (%)
1	24.392	50.040
2	36.242	49.960

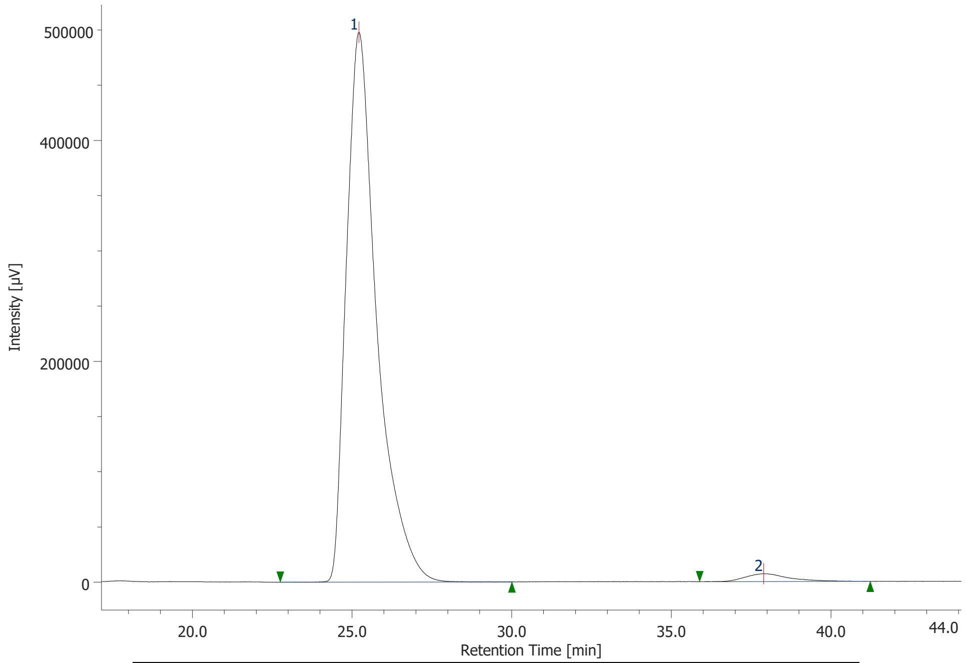

Peak No.	Retention Time (min)	Area (%)
1	25.217	97.986
2	37.883	2.014

3cb

ID-3 / 2-PrOH 20%

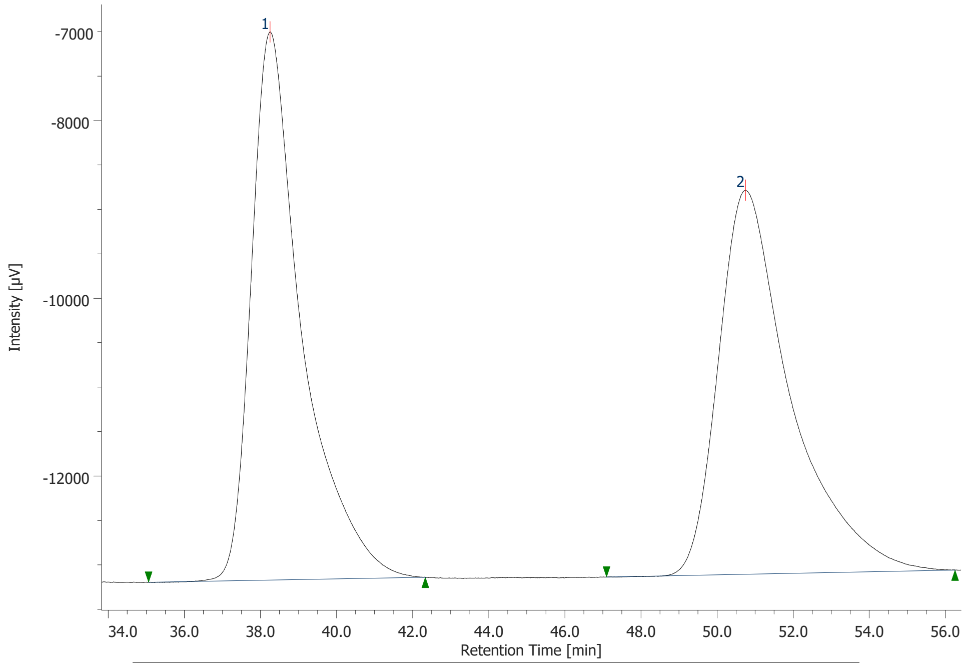

Peak No.	Retention Time (min)	Area (%)
1	38.250	50.167
2	50.742	49.833

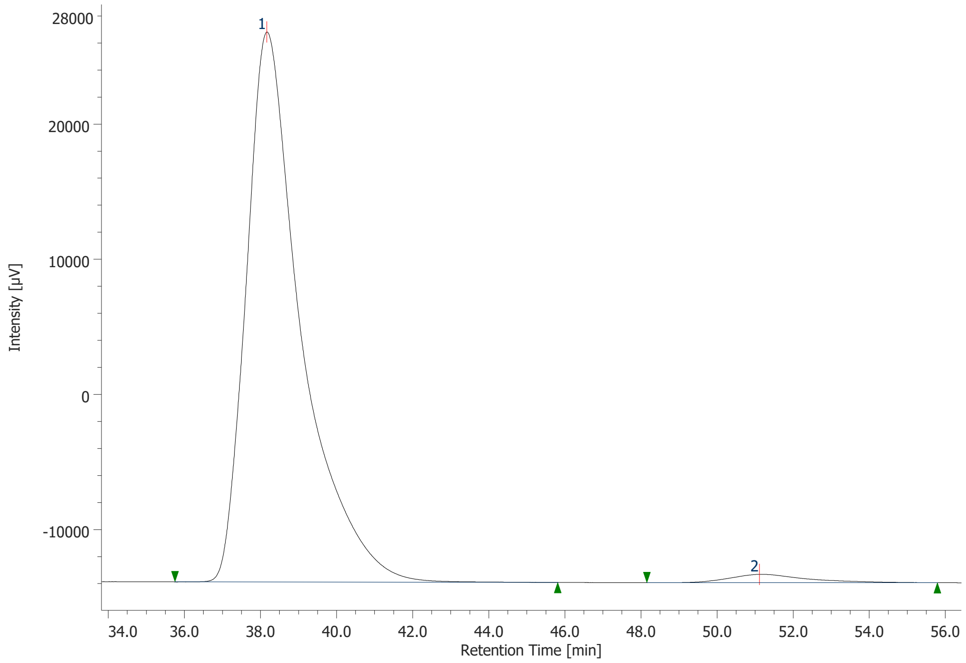

Peak No.	Retention Time (min)	Area (%)
1	38.167	97.919
2	51.108	2.081

3db

IG-3 / 2-PrOH 20%

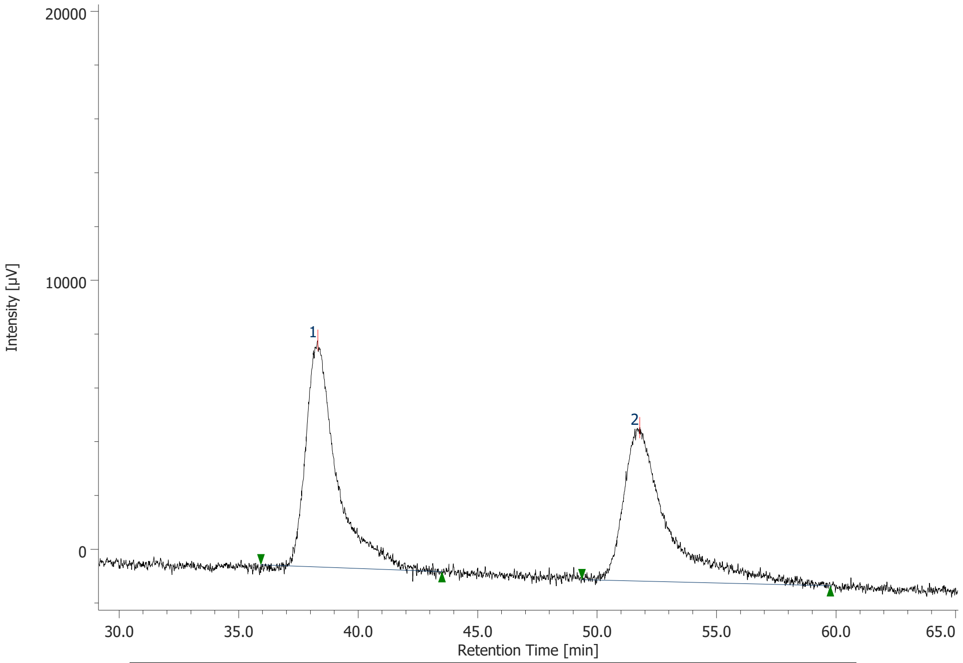

Peak No.	Retention Time (min)	Area (%)
1	38.308	50.492
2	51.783	49.508

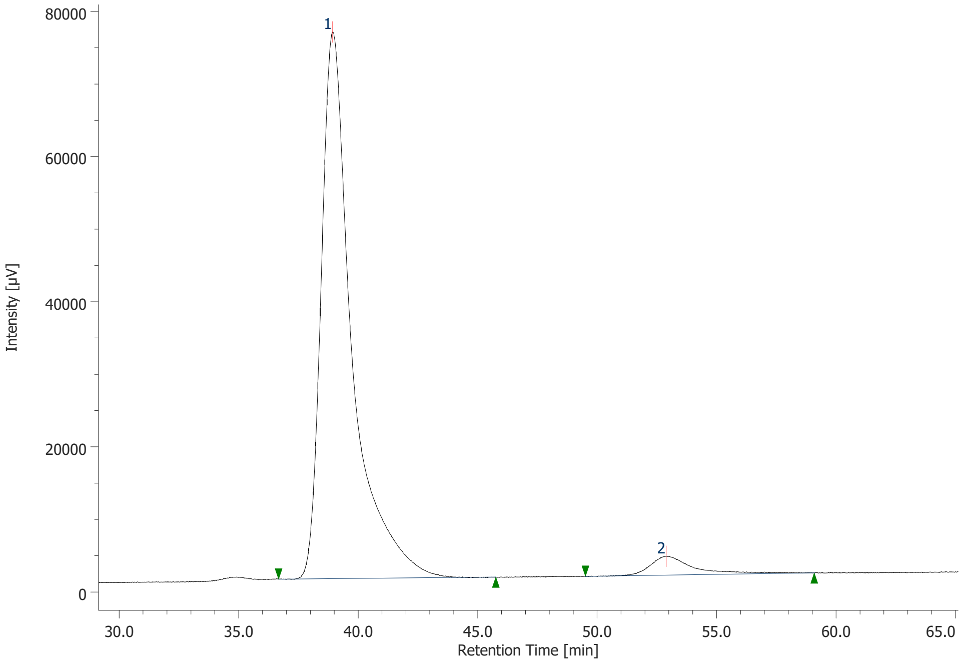

Peak No.	Retention Time (min)	Area (%)
1	38.933	95.307
2	52.883	4.693

3eb

IE-3 / 2-PrOH 20%

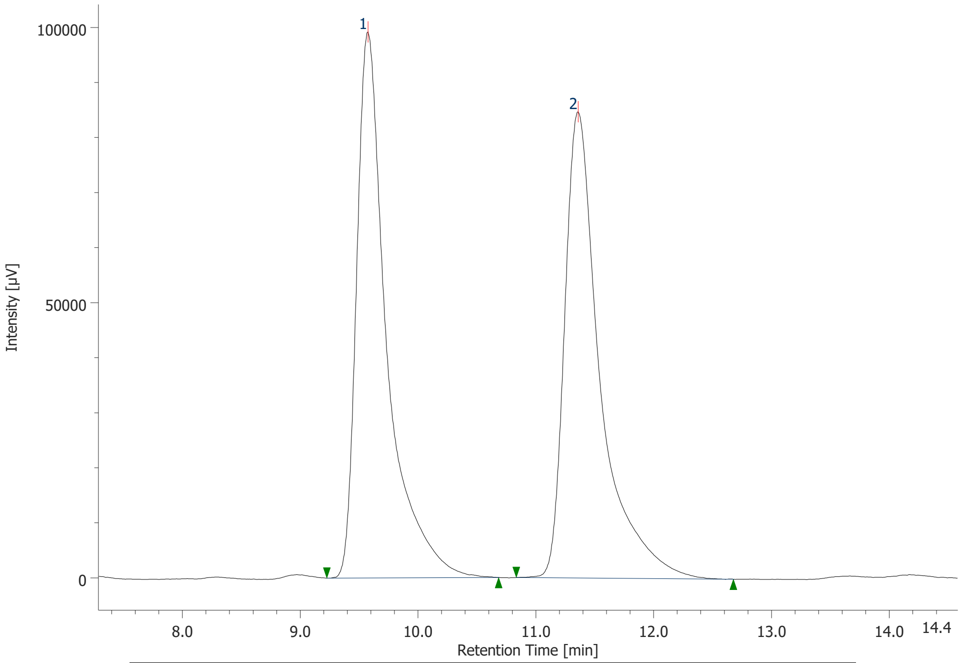

Peak No.	Retention Time (min)	Area (%)
1	9.575	49.994
2	11.358	50.006

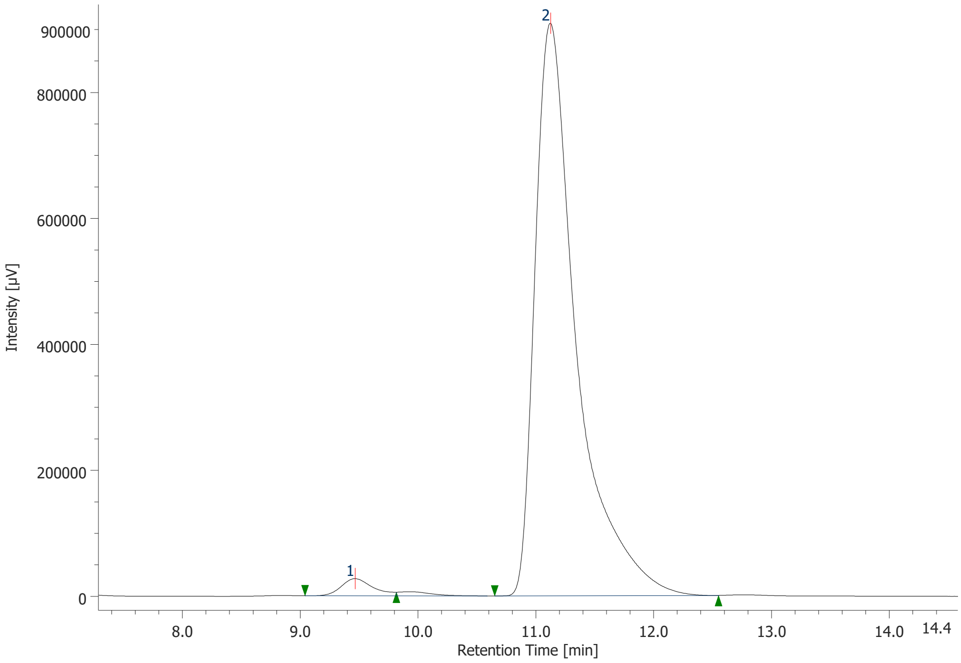

Peak No.	Retention Time (min)	Area (%)
1	9.467	2.256
2	11.125	97.744

4eb

IG-3 / 2-PrOH 20%

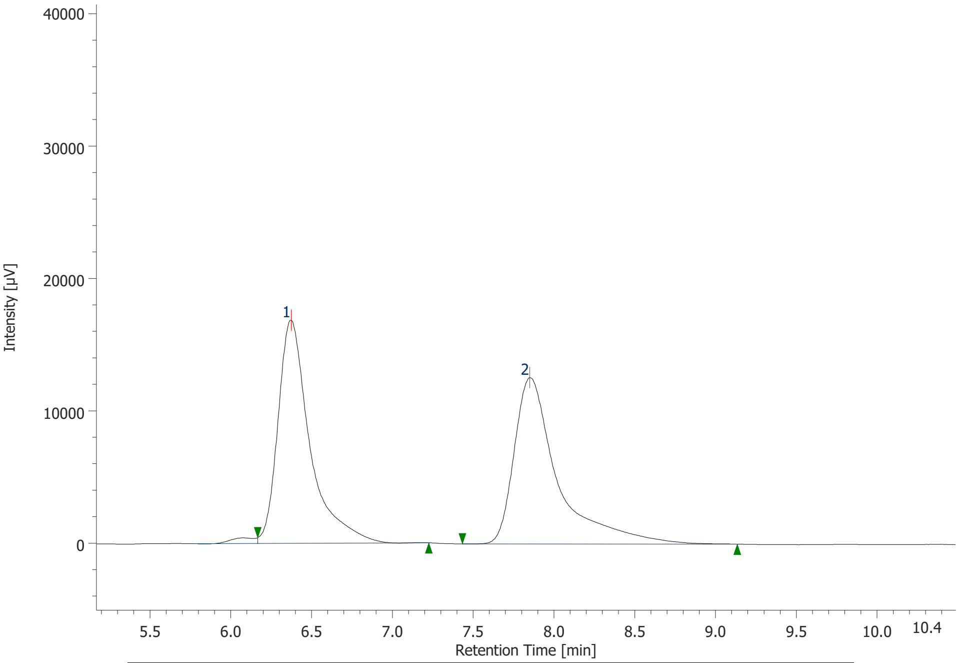

Peak No.	Retention Time (min)	Area (%)
1	6.375	49.734
2	7.850	50.266

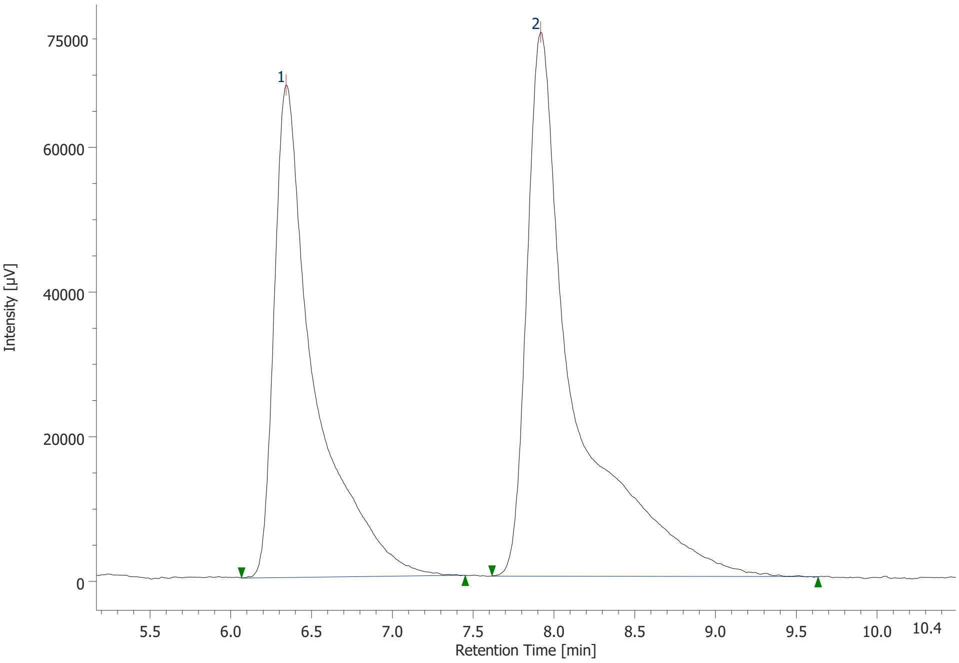

Peak No.	Retention Time (min)	Area (%)
1	6.342	43.267
2	7.917	56.733

3fb

ID-3 / 2-PrOH 8%

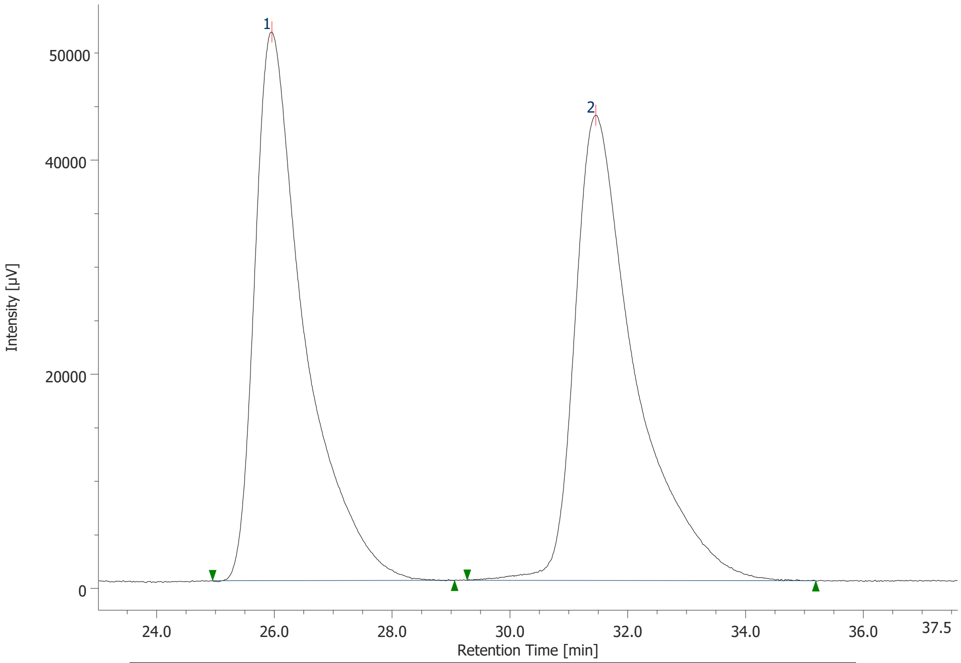

Peak No.	Retention Time (min)	Area (%)
1	25.958	49.254
2	31.458	50.746

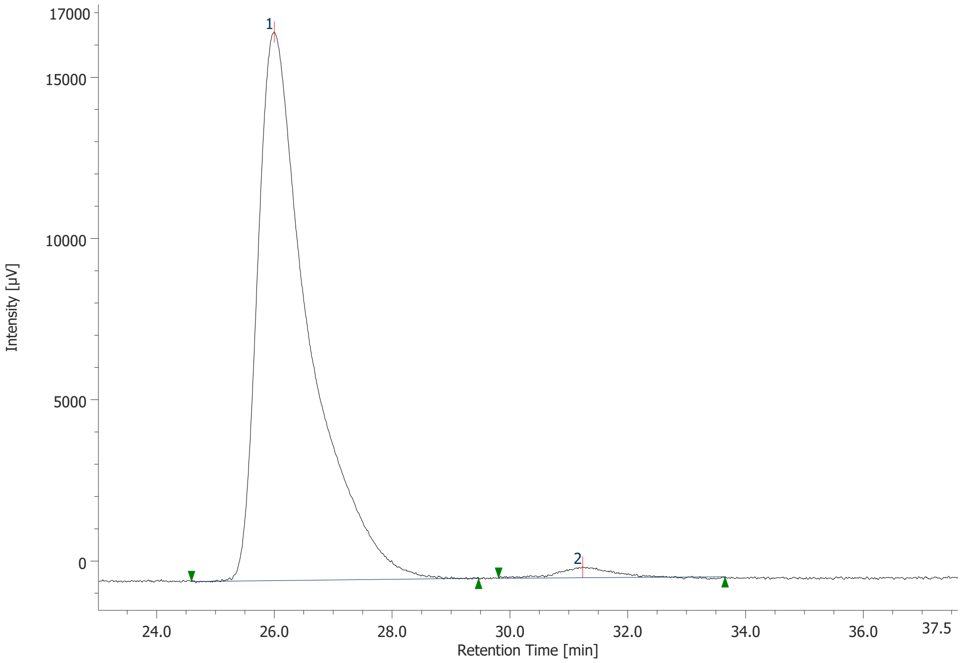

Peak No.	Retention Time (min)	Area (%)
1	26.000	98.004
2	31.233	1.996

3ai

IF-3 / 2-PrOH 20%

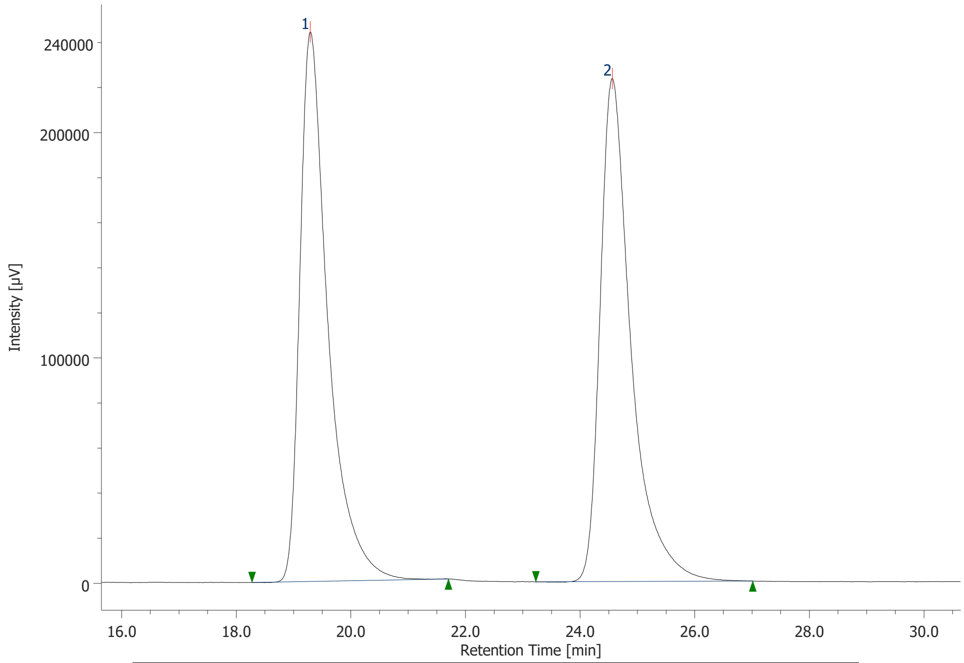

Peak No.	Retention Time (min)	Area (%)
1	19.292	49.866
2	24.558	50.134

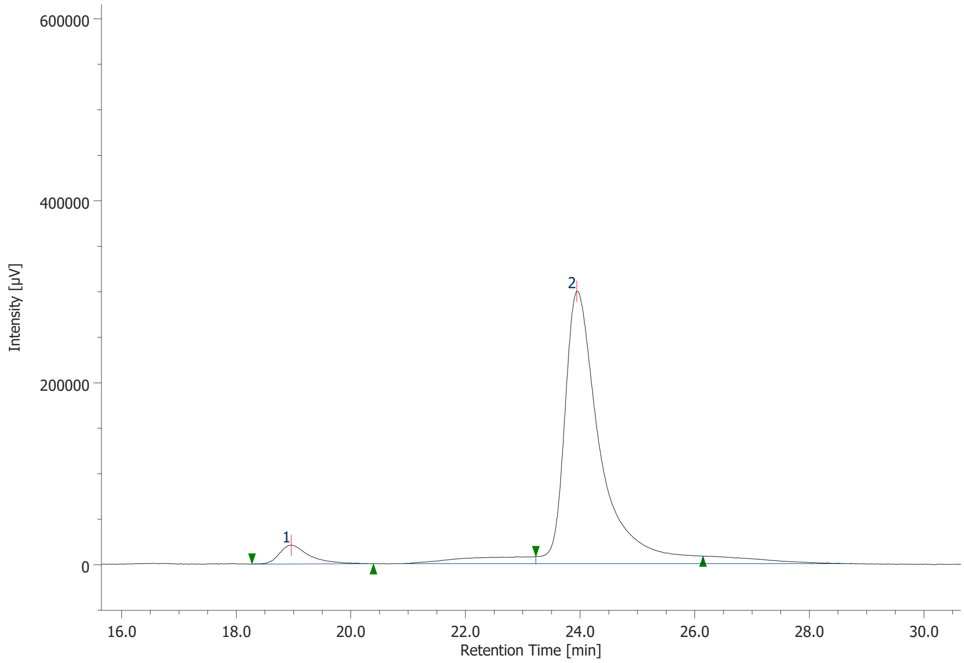

Peak No.	Retention Time (min)	Area (%)
1	18.958	5.581
2	23.942	94.419

4ai

IF-3 / 2-PrOH 20%

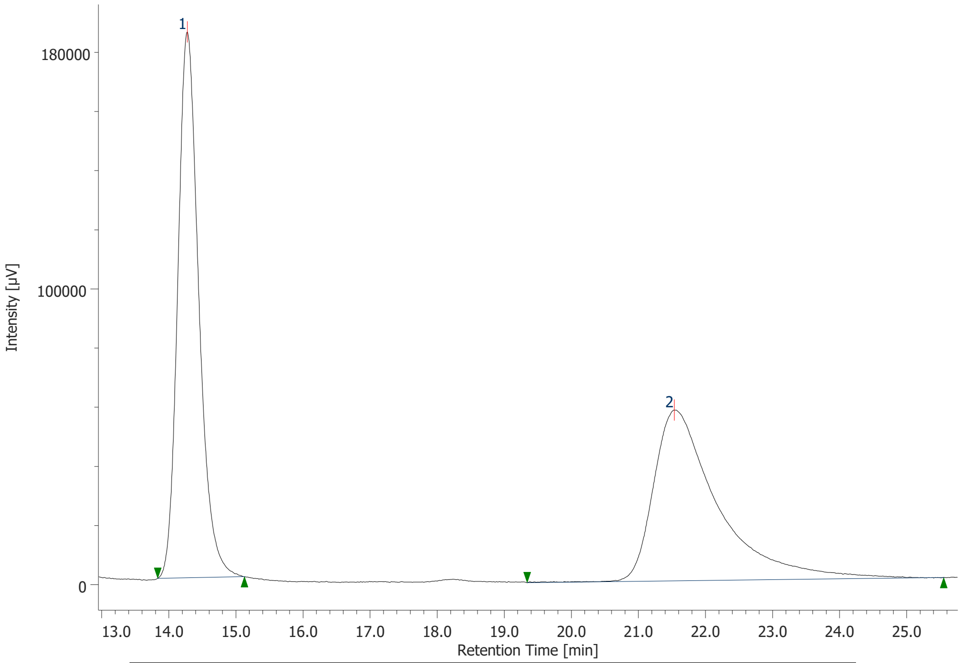

Peak No.	Retention Time (min)	Area (%)
1	14.275	51.087
2	21.533	48.913

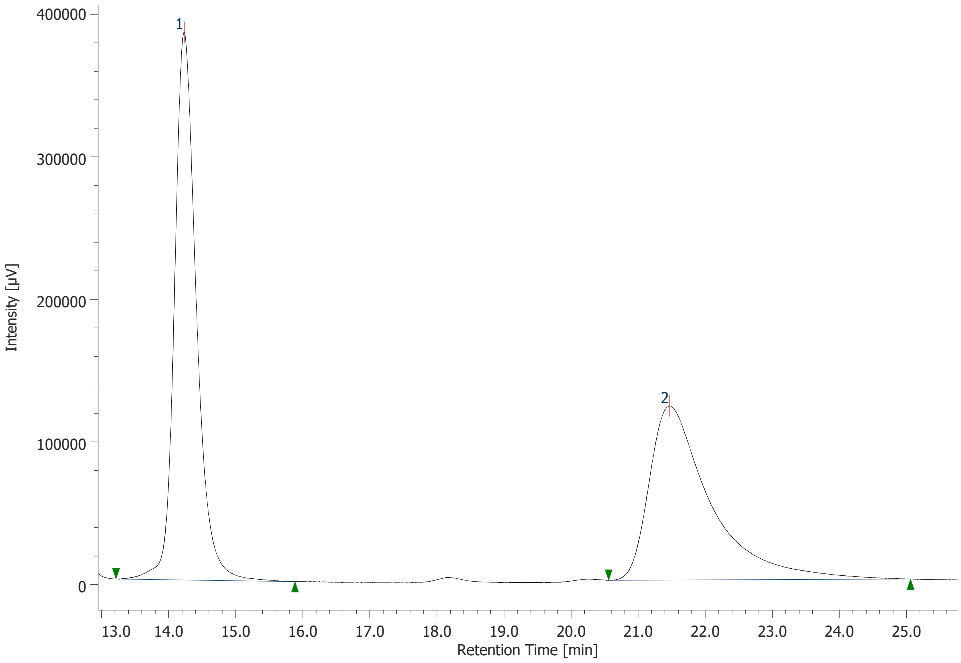

Peak No.	Retention Time (min)	Area (%)
1	14.233	53.036
2	21.467	46.964

3ga

IF-3 / 2-PrOH 0.5%

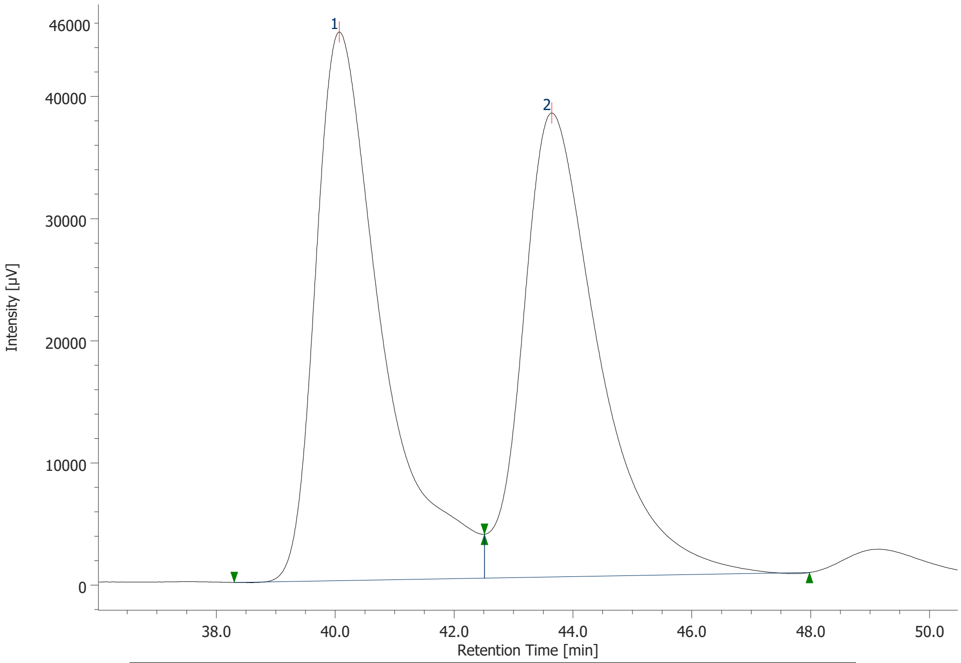

Peak No.	Retention Time (min)	Area (%)
1	40.067	50.780
2	43.642	49.220

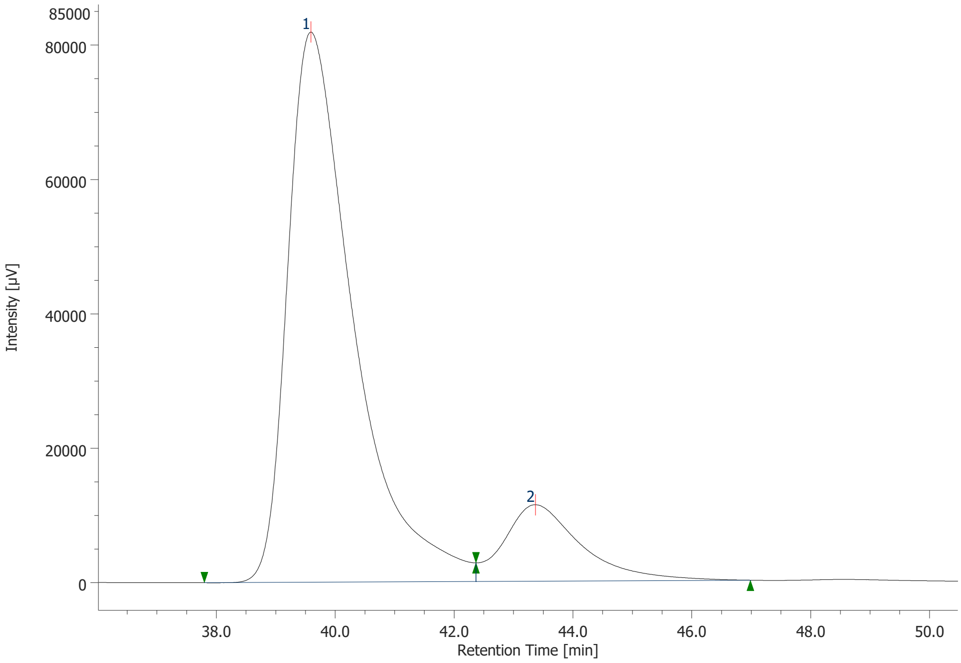

Peak No.	Retention Time (min)	Area (%)
1	39.592	86.063
2	43.367	13.937

3ha

IG-3 / 2-PrOH 5%

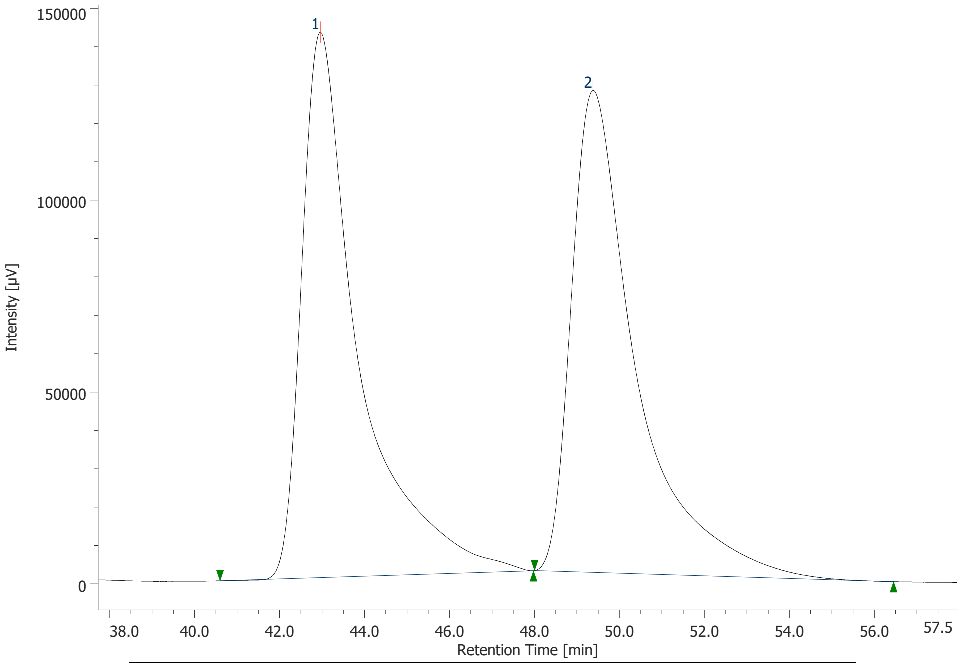

Peak No.	Retention Time (min)	Area (%)
1	42.958	50.036
2	49.375	49.964

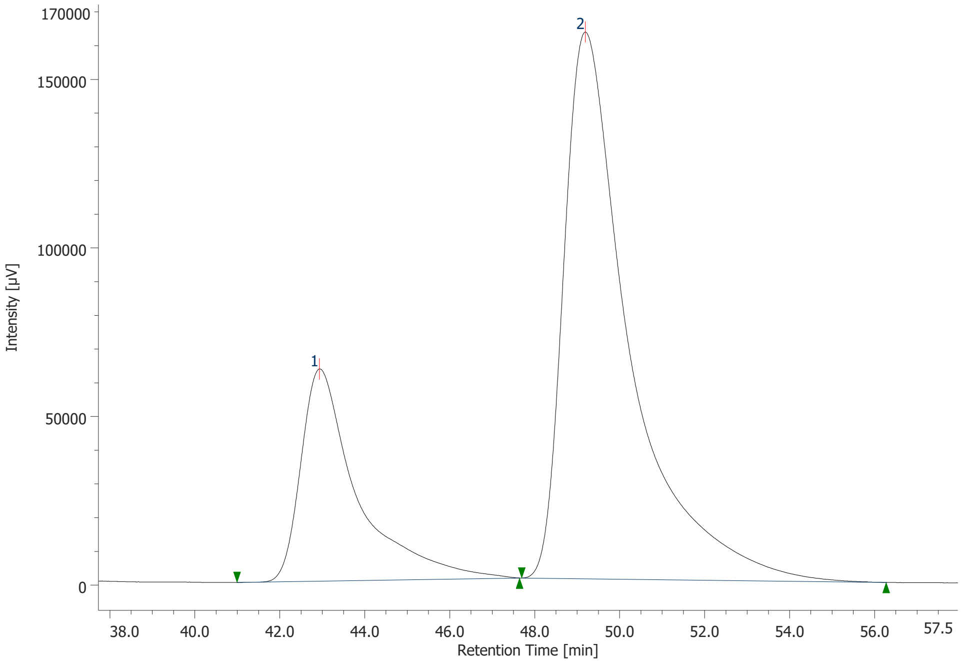

Peak No.	Retention Time (min)	Area (%)
1	42.933	25.004
2	49.192	74.996

3ia

ID-3 / 2-PrOH 5%

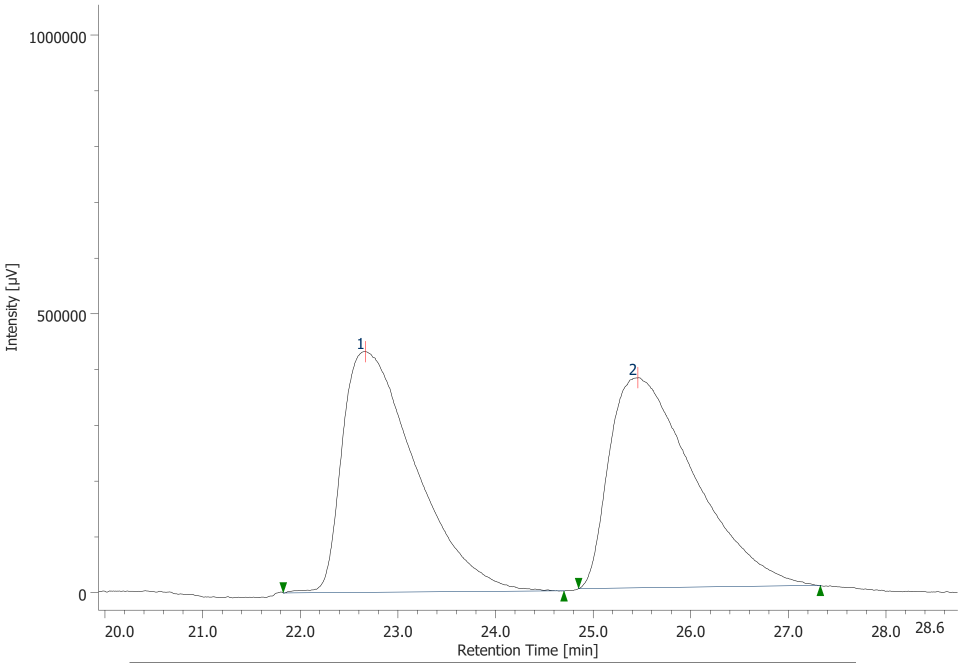

Peak No.	Retention Time (min)	Area (%)
1	22.667	49.637
2	25.458	50.363

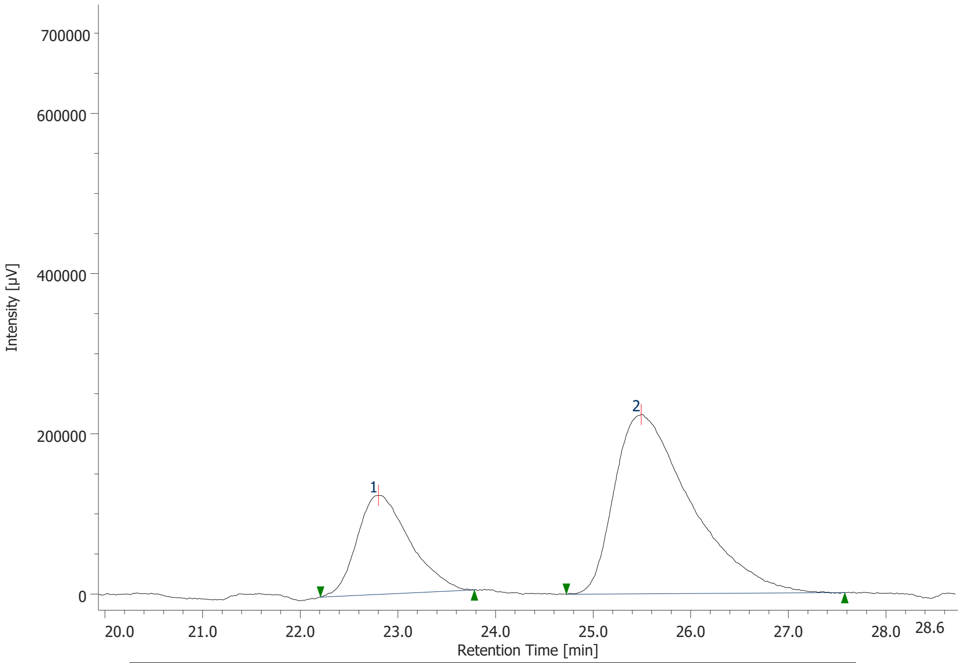

Peak No.	Retention Time (min)	Area (%)
1	22.800	27.544
2	25.492	72.456

3ja

IE-3 / 2-PrOH 2%

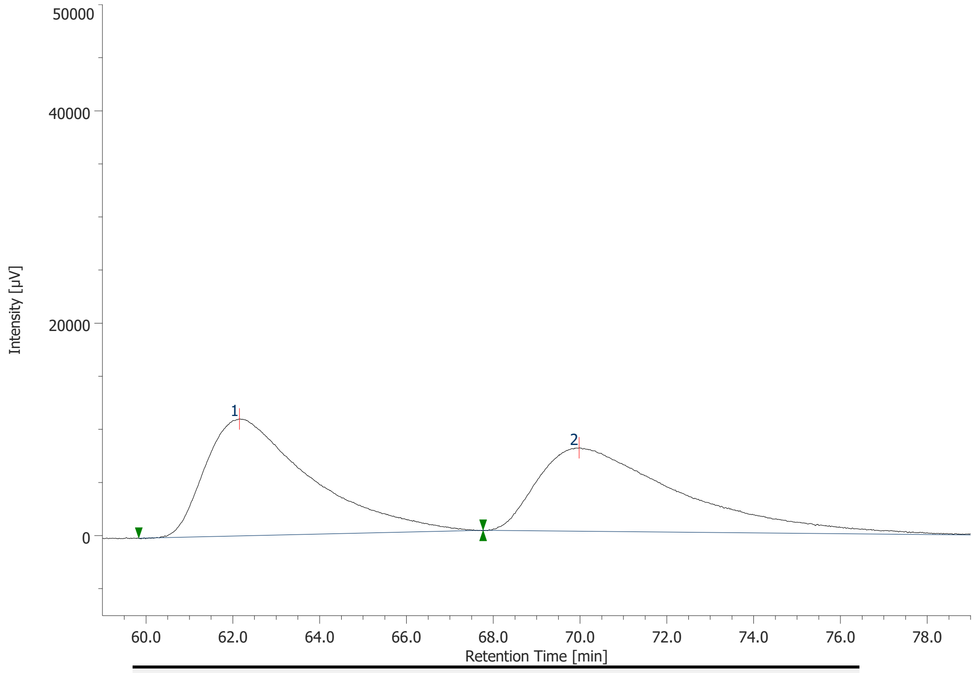

Peak No.	Retention Time (min)	Area (%)
1	62.150	50.779
2	69.975	49.221

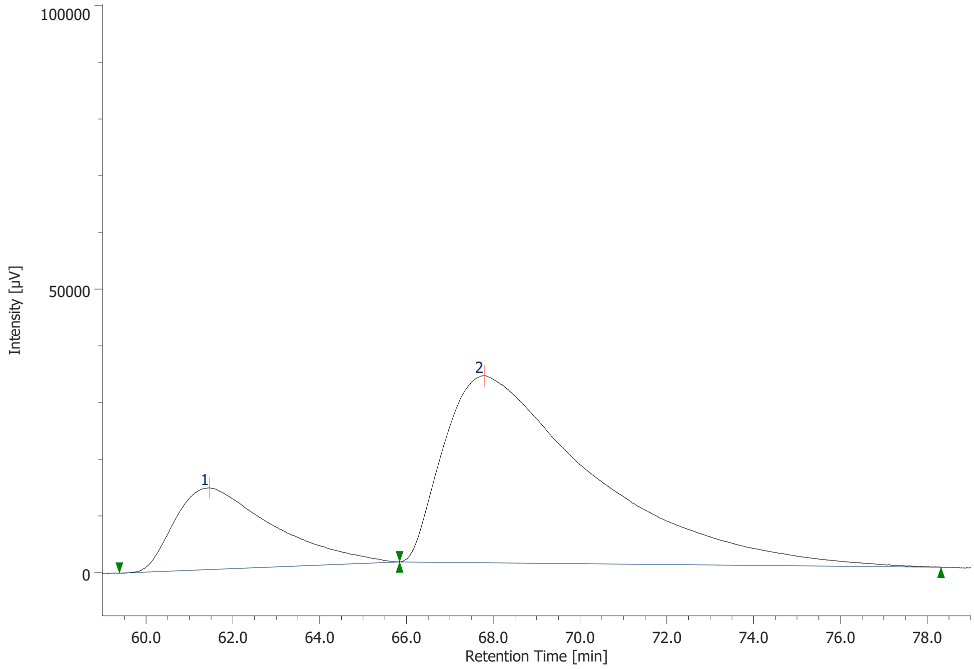

Peak No.	Retention Time (min)	Area (%)
1	61.467	22.355
2	67.792	77.645

3ka

IG-3 / 2-PrOH 5%

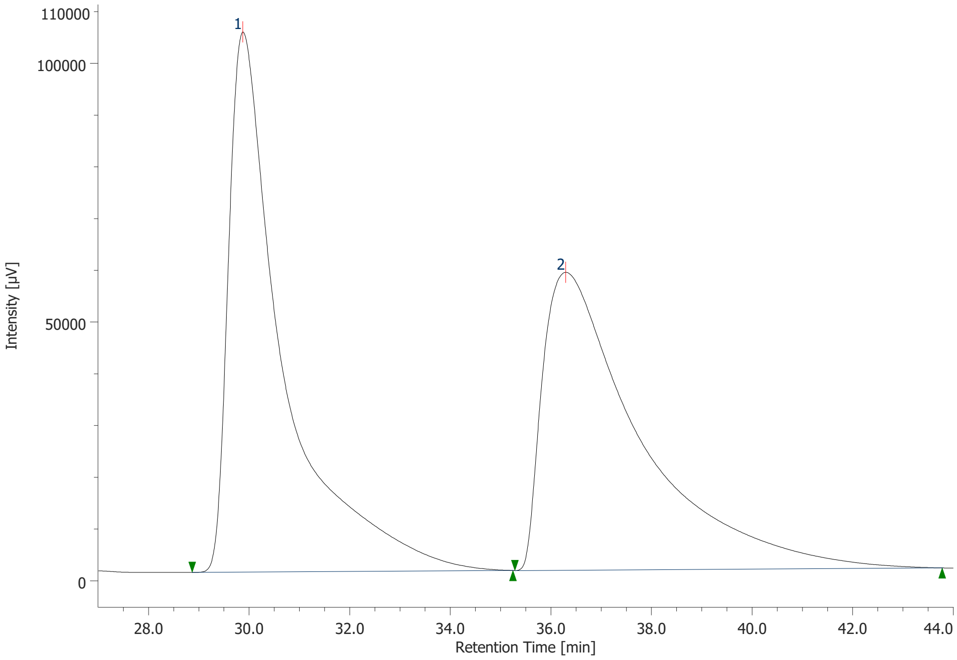

Peak No.	Retention Time (min)	Area (%)
1	29.875	50.428
2	36.282	49.572

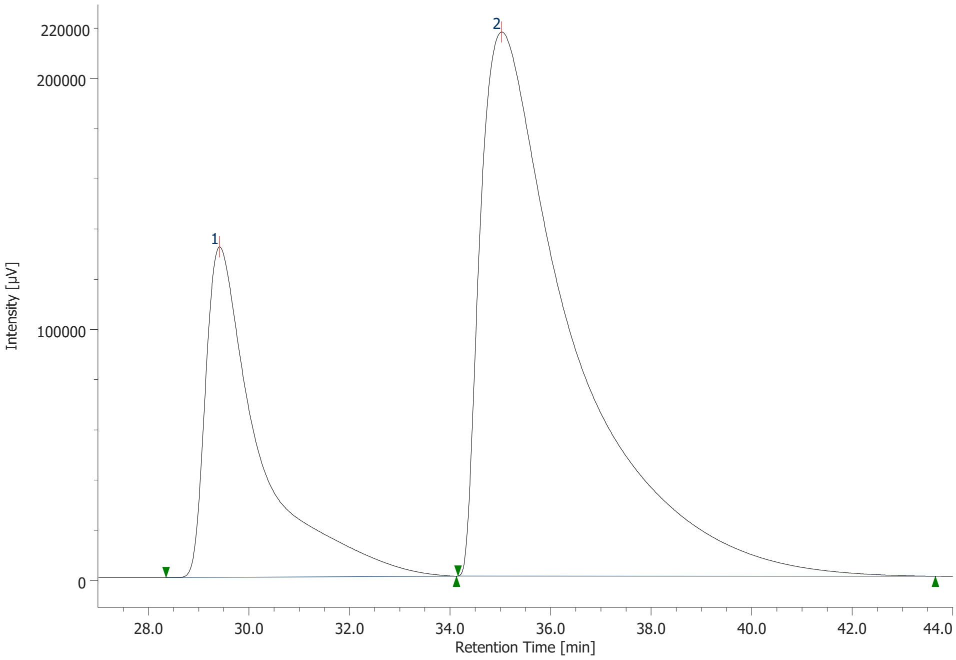

Peak No.	Retention Time (min)	Area (%)
1	29.417	26.116
2	35.025	73.884

3la

IF-3 / 2-PrOH 15%

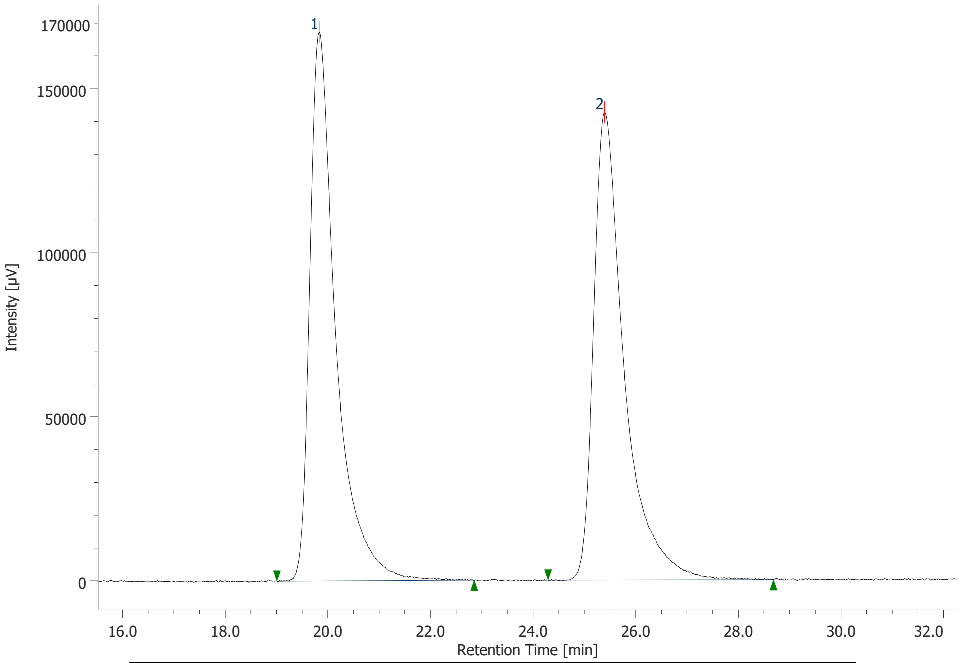

Peak No.	Retention Time (min)	Area (%)
1	19.833	49.983
2	25.392	50.017

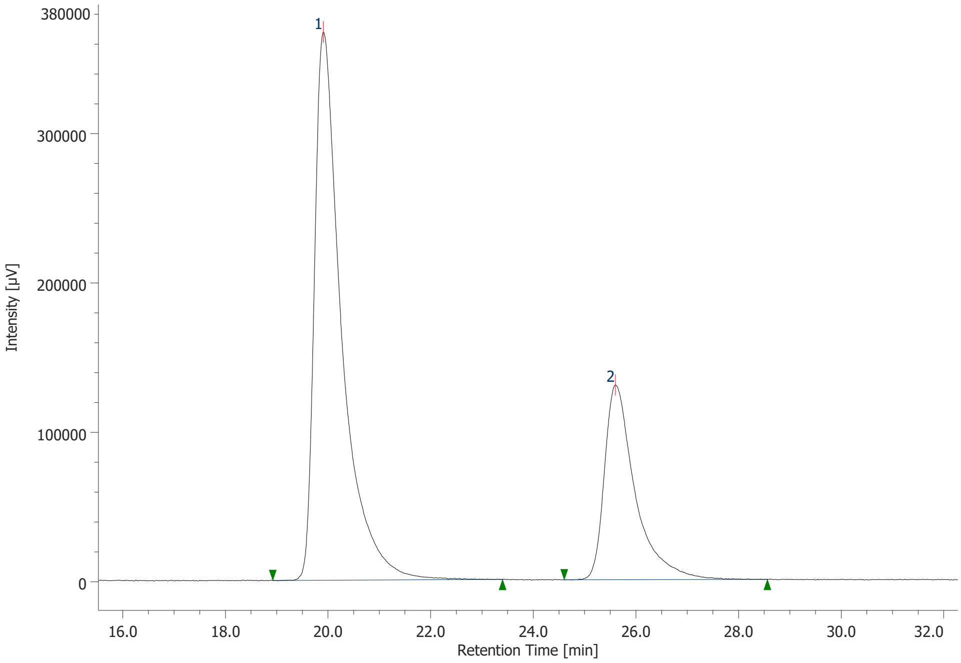

Peak No.	Retention Time (min)	Area (%)
1	19.908	71.430
2	25.600	28.570

3hb

IE-3 / 2-PrOH 20%

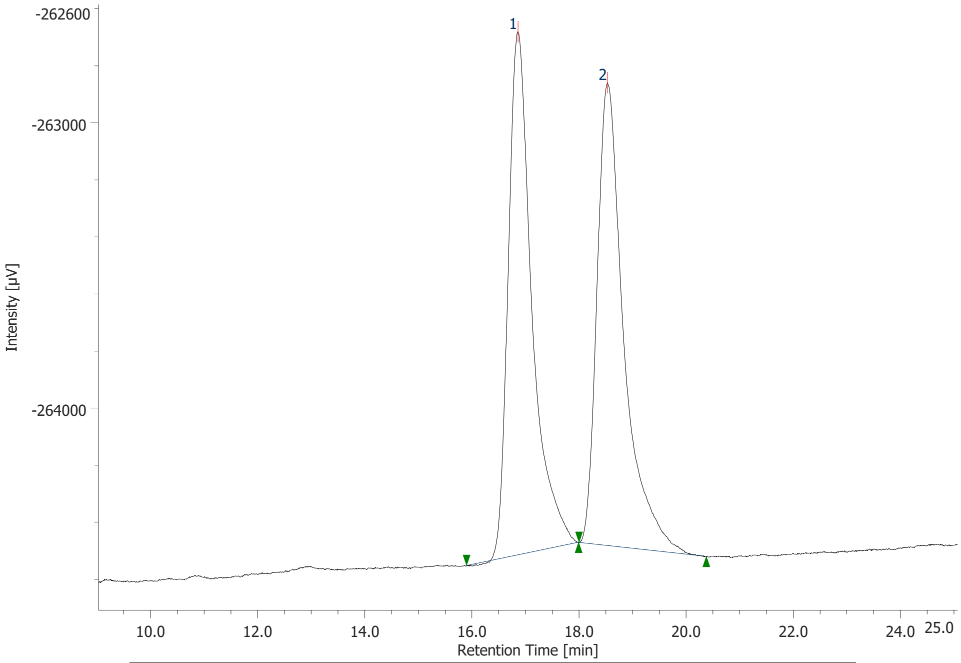

Peak No.	Retention Time (min)	Area (%)
1	16.858	50.058
2	18.533	49.942

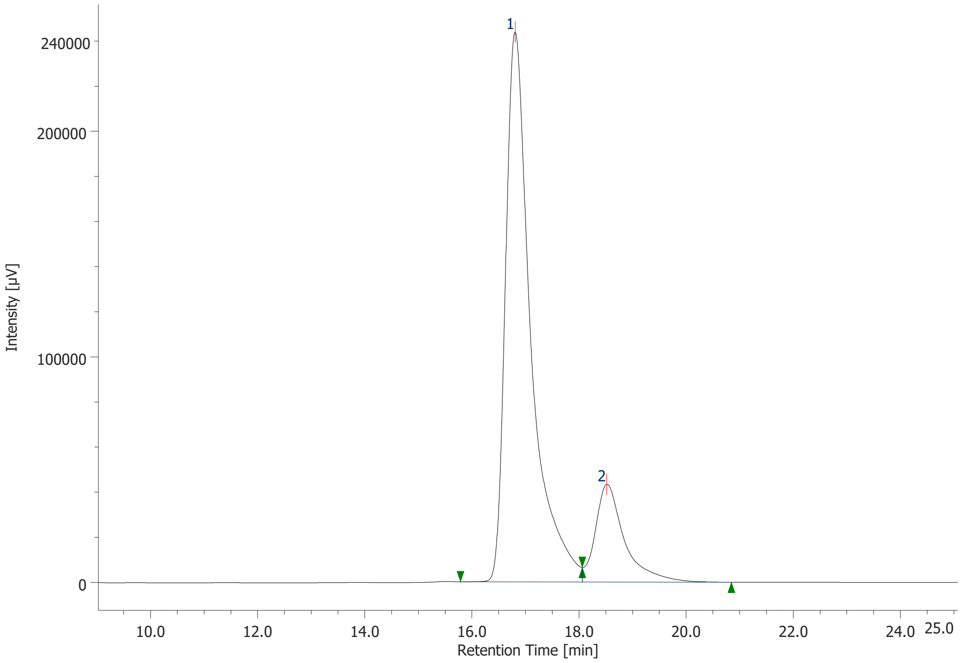

Peak No.	Retention Time (min)	Area (%)
1	16.808	83.079
2	18.517	16.921

3aj

IF-3 / 2-PrOH 20%

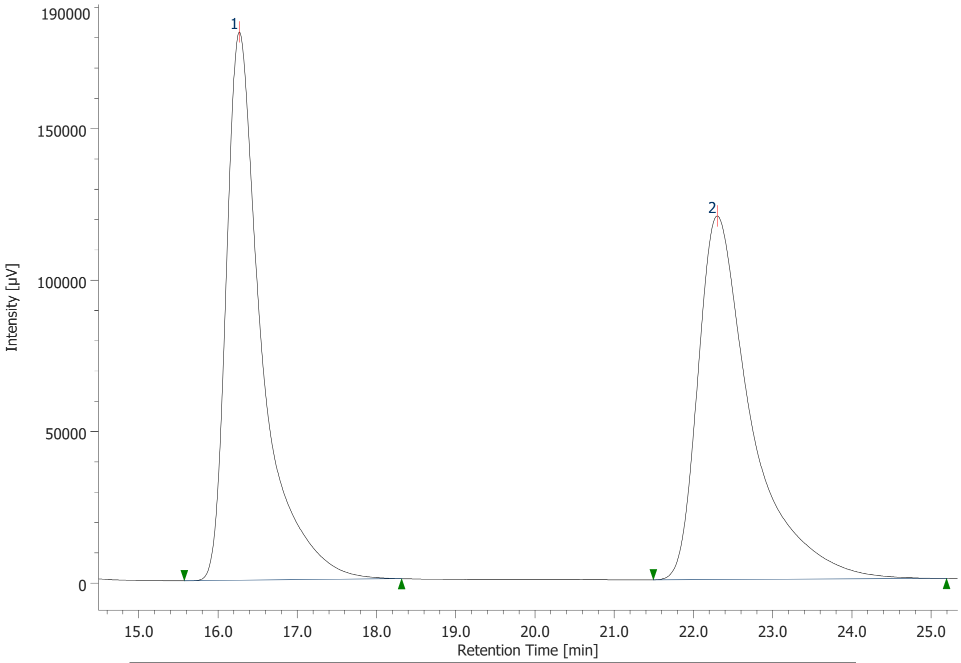

Peak No.	Retention Time (min)	Area (%)
1	16.267	50.029
2	22.300	49.971

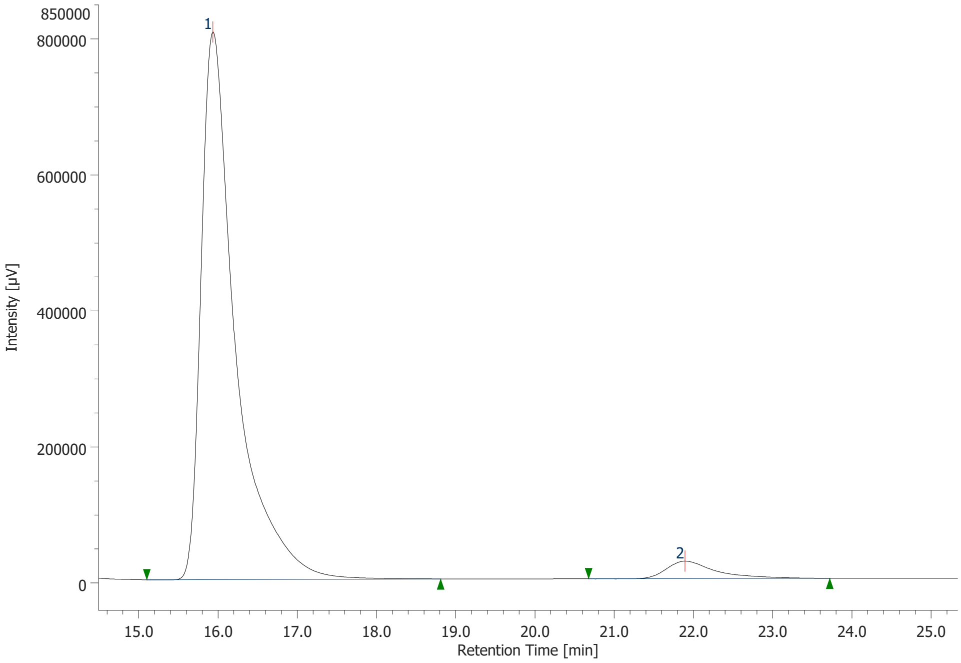

Peak No.	Retention Time (min)	Area (%)
1	15.933	95.654
2	21.892	4.346

3ak

IF-3 / 2-PrOH 20%

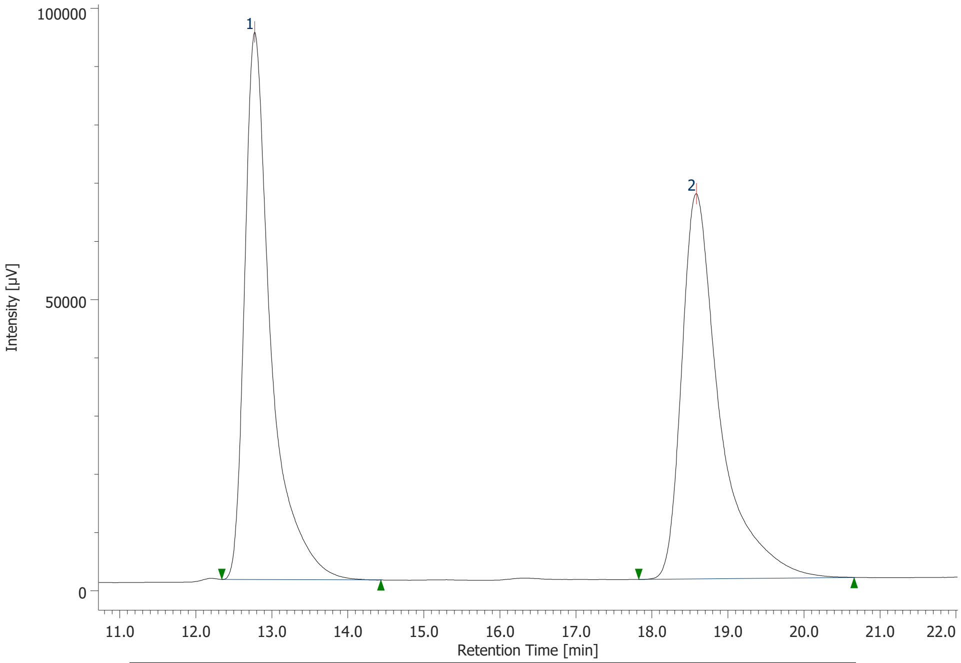

Peak No.	Retention Time (min)	Area (%)
1	12.775	49.622
2	18.583	50.378

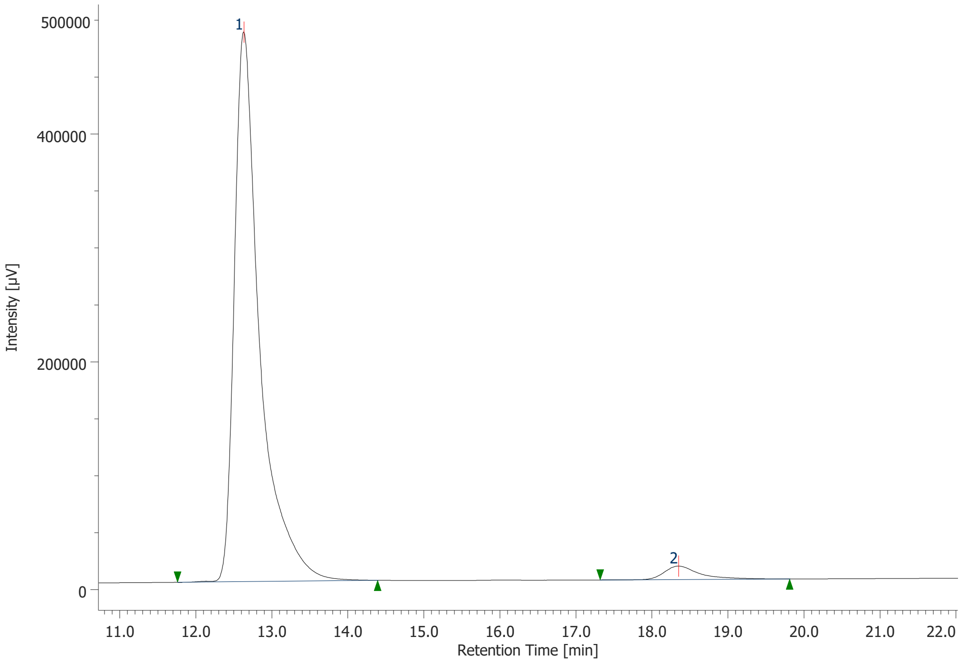

Peak No.	Retention Time (min)	Area (%)
1	12.633	96.720
2	18.350	3.280

3al

IG-3 / 2-PrOH 20%

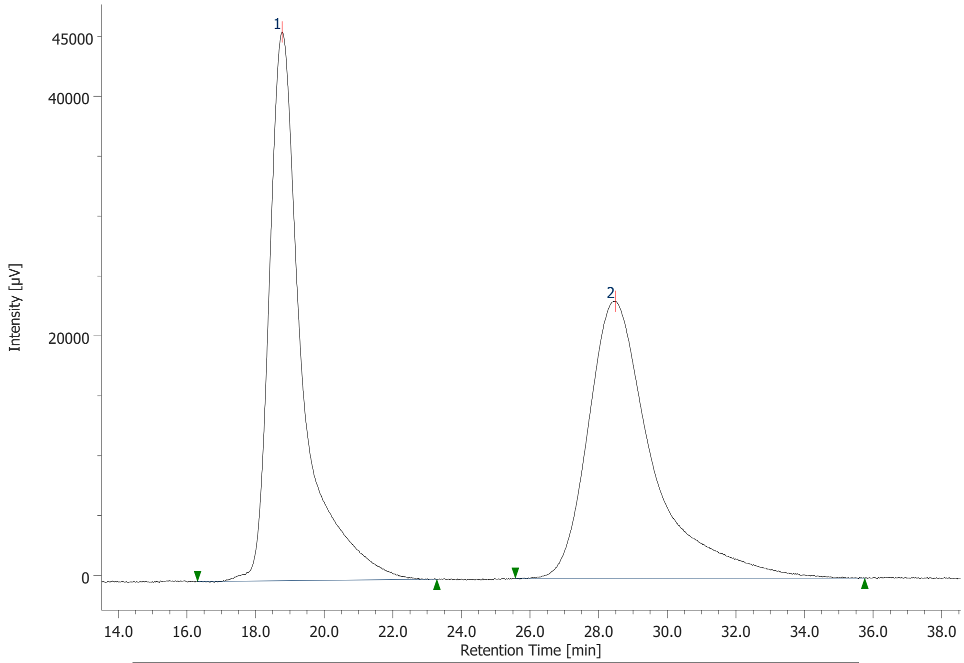

Peak No.	Retention Time (min)	Area (%)
1	18.775	50.203
2	28.492	49.797

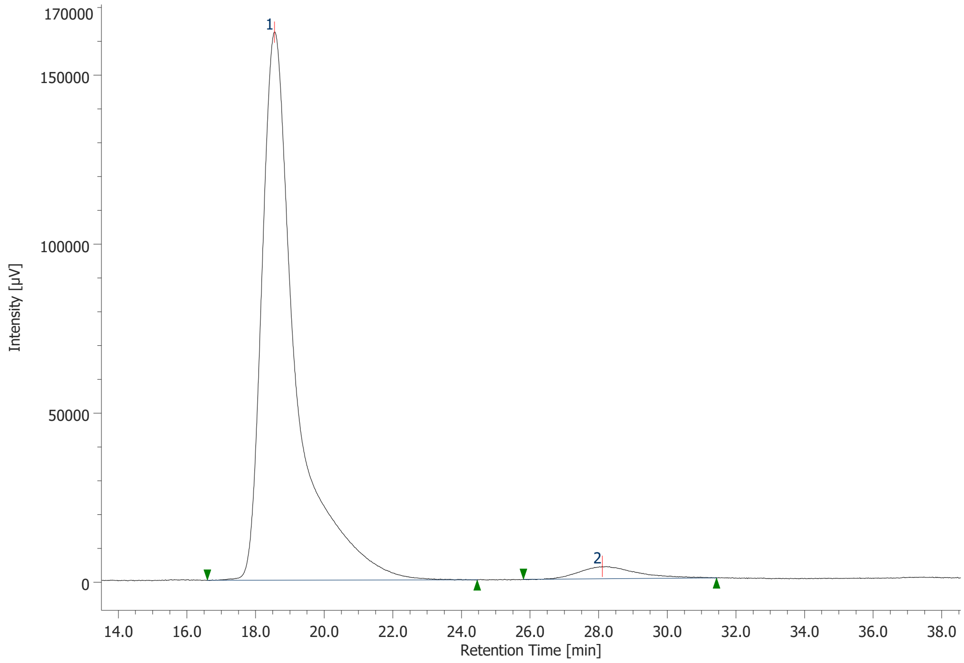

Peak No.	Retention Time (min)	Area (%)
1	18.550	96.384
2	28.100	3.616

3mc

IE-3 / 2-PrOH 20%

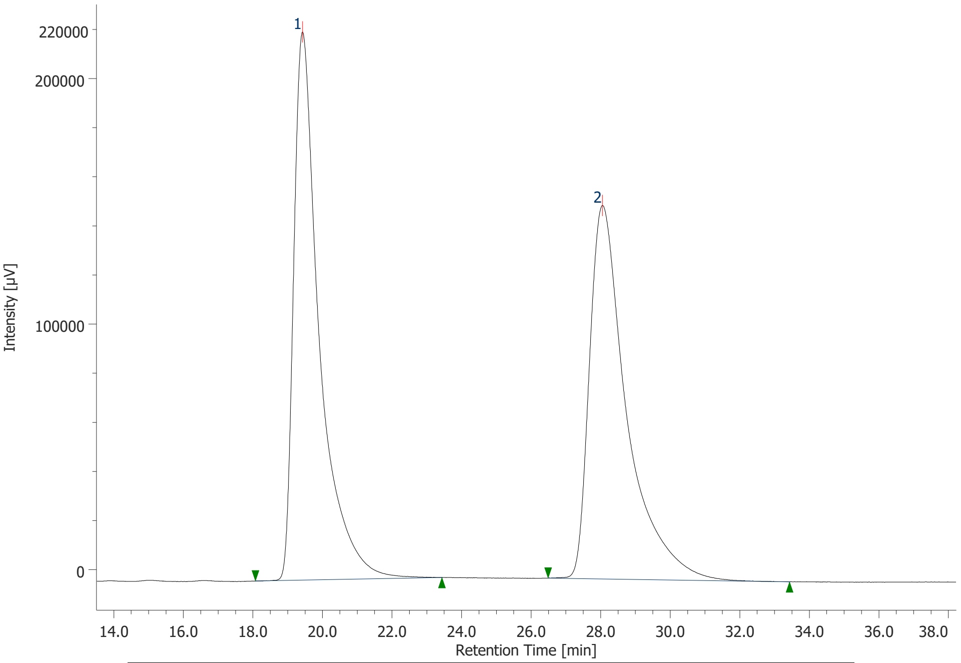

Peak No.	Retention Time (min)	Area (%)
1	19.425	50.353
2	28.050	49.647

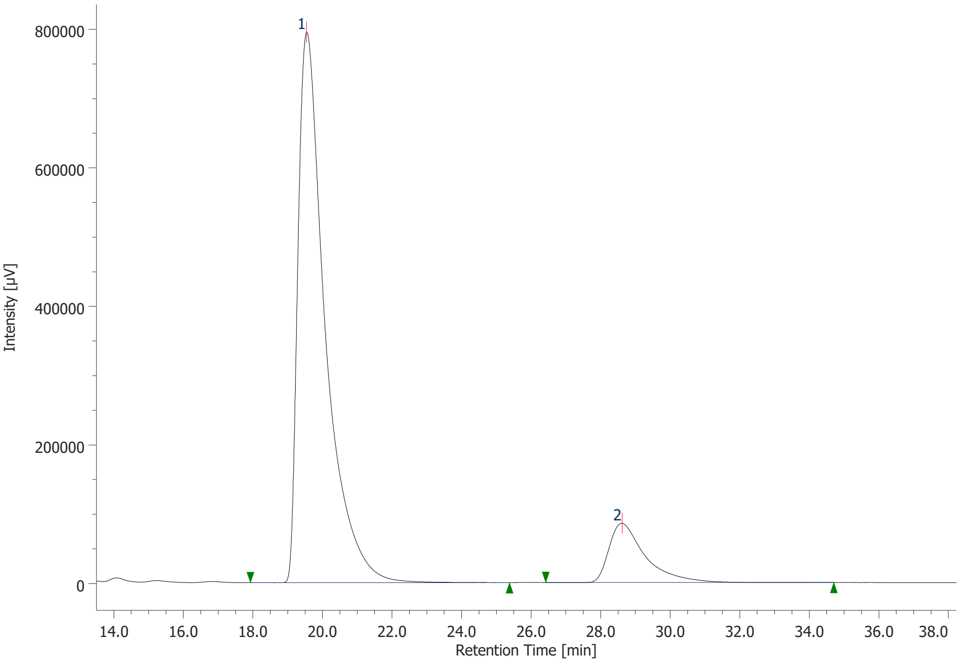

Peak No.	Retention Time (min)	Area (%)
1	19.542	87.537
2	28.617	12.463

3gc

IB / 2-PrOH 1%

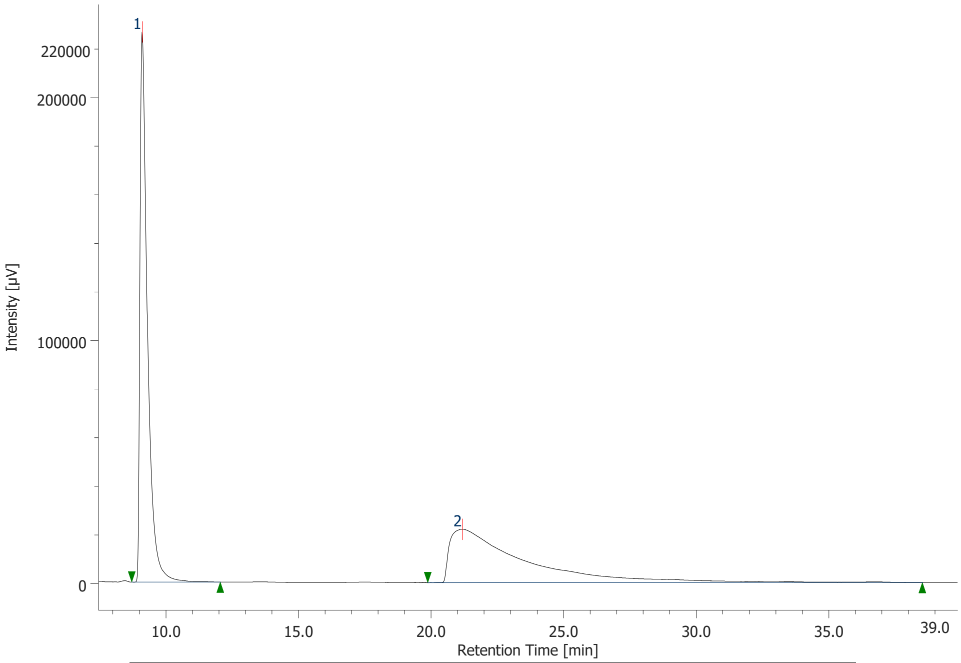

Peak No.	Retention Time (min)	Area (%)
1	9.108	51.023
2	21.175	48.977

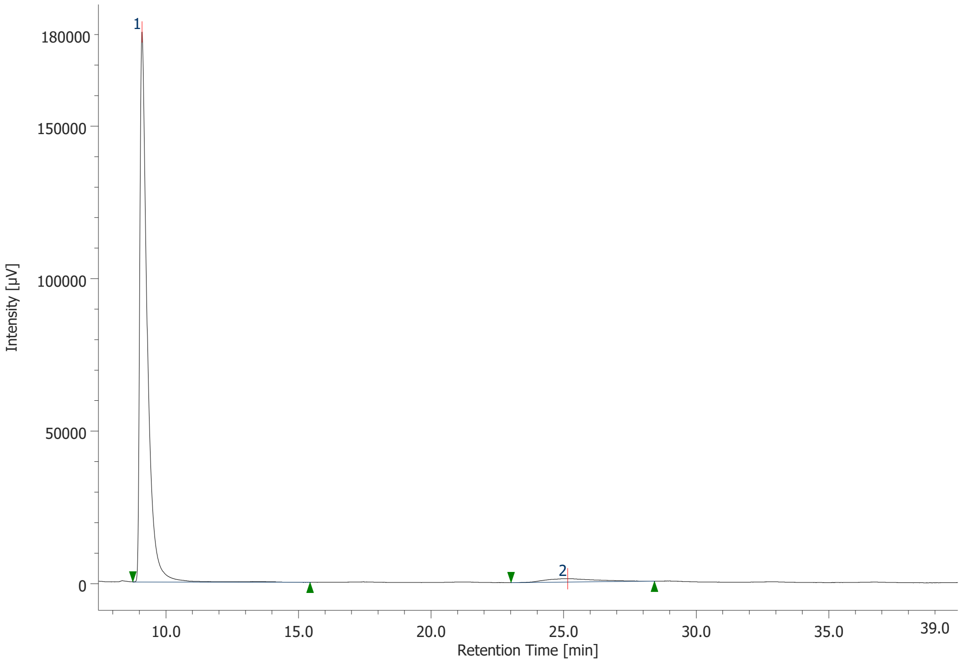

Peak No.	Retention Time (min)	Area (%)
1	9.100	96.057
2	25.150	3.943

3na

IA / 2-PrOH 10%

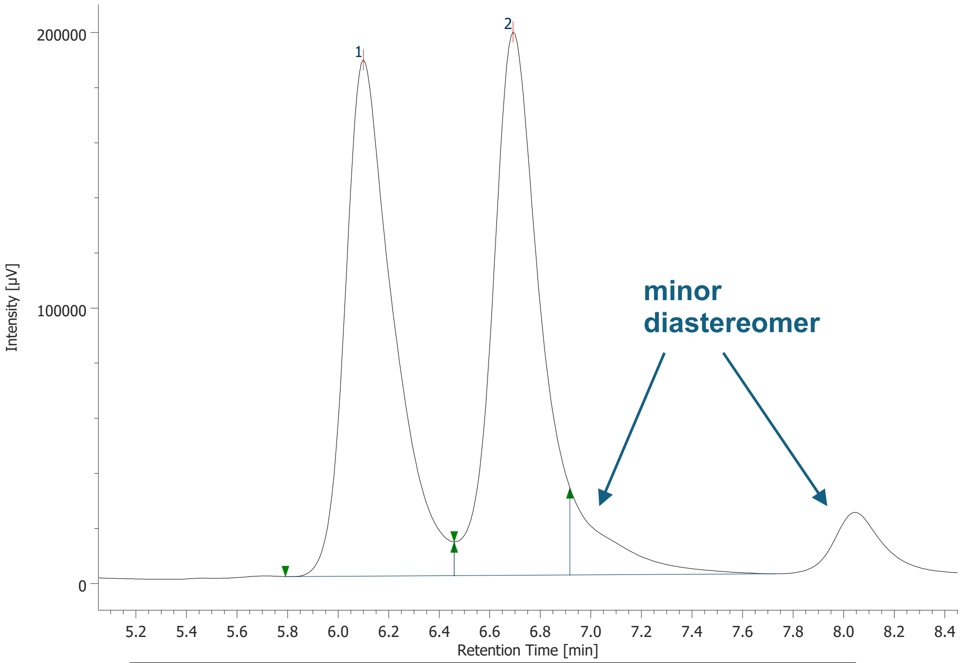

Peak No.	Retention Time (min)	Area (%)
1	6.100	49.908
2	6.692	50.092

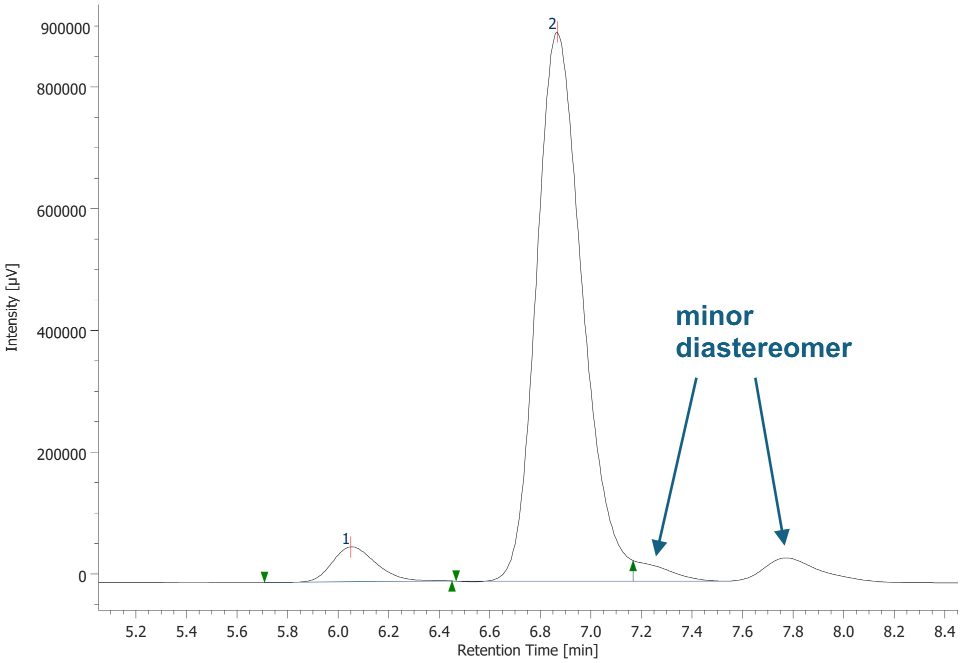

Peak No.	Retention Time (min)	Area (%)
1	6.050	5.733
2	6.867	94.267

3oa

IG-3 / 2-PrOH 8%

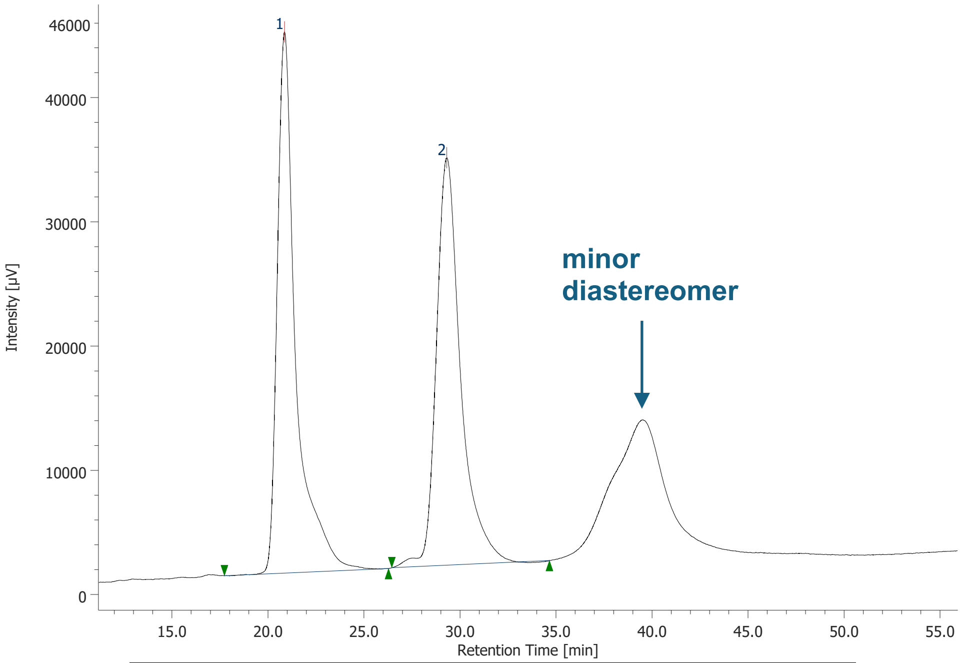

Peak No.	Retention Time (min)	Area (%)
1	20.858	50.399
2	29.308	49.601

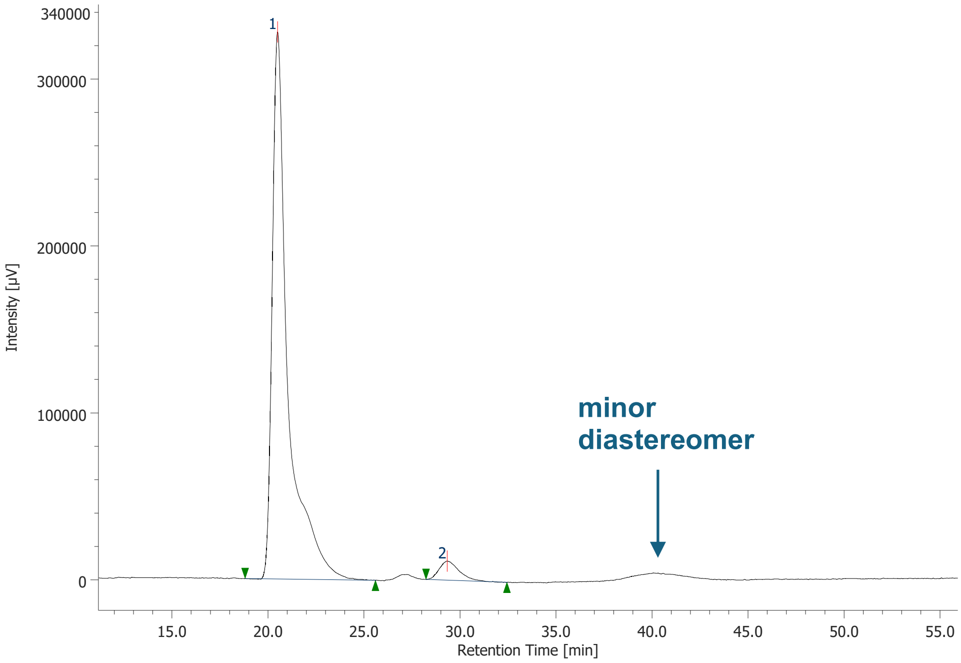

Peak No.	Retention Time (min)	Area (%)
1	20.500	95.733
2	29.333	4.267

3pa

IF-3 / 2-PrOH 20%

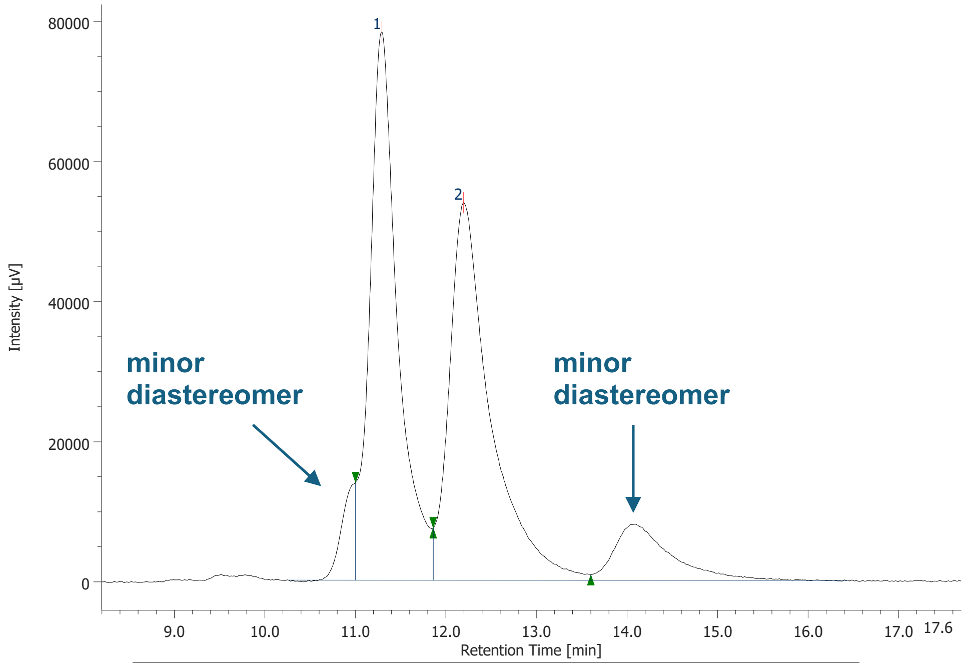

Peak No.	Retention Time (min)	Area (%)
1	11.292	49.940
2	12.192	50.060

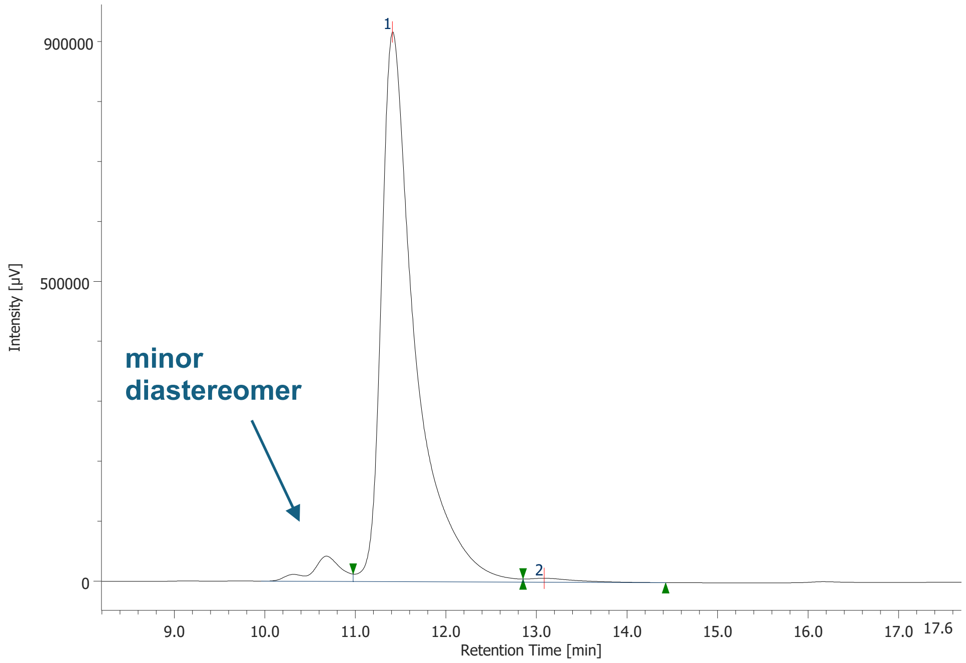

Peak No.	Retention Time (min)	Area (%)
1	11.408	98.994
2	13.083	1.006

3nc

IG-3 / 2-PrOH 20%

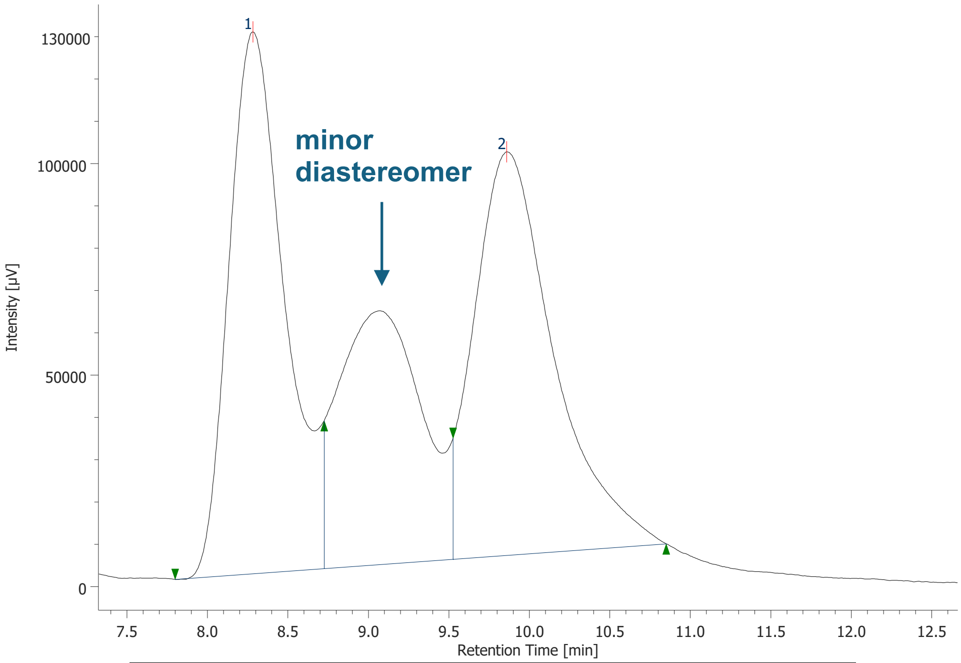

Peak No.	Retention Time (min)	Area (%)
1	8.283	48.638
2	9.858	51.362

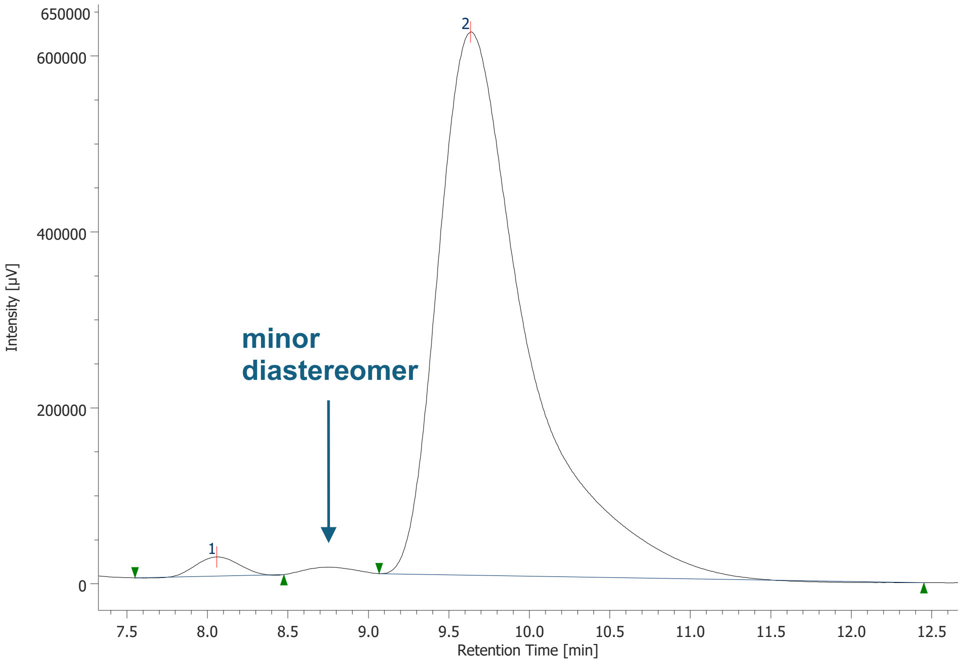

Peak No.	Retention Time (min)	Area (%)
1	8.058	1.660
2	9.633	98.340

3oc

IE-3 / 2-PrOH 40%

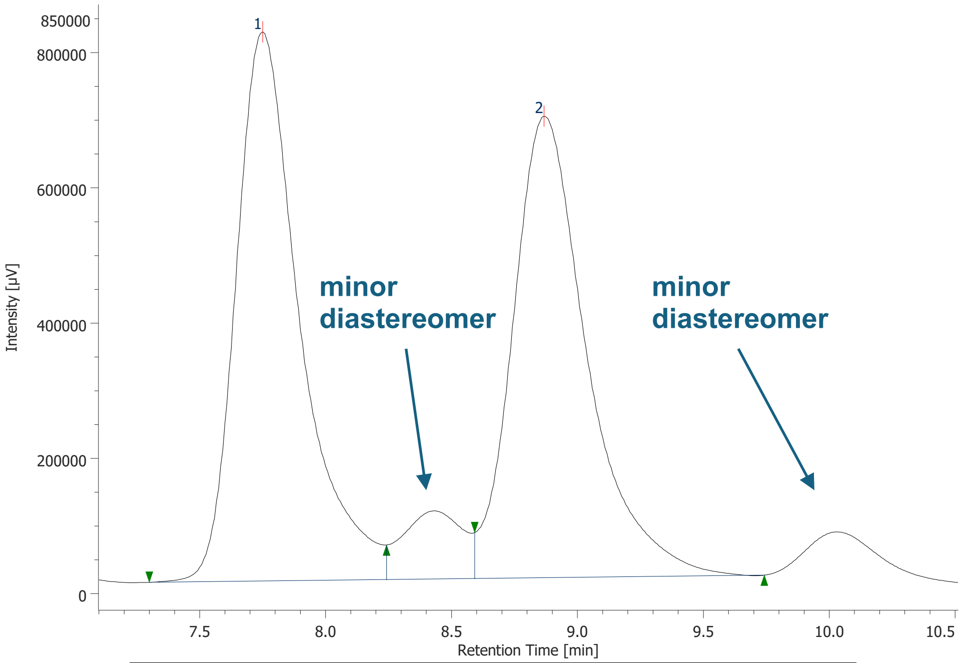

Peak No.	Retention Time (min)	Area (%)
1	7.750	50.620
2	8.867	49.380

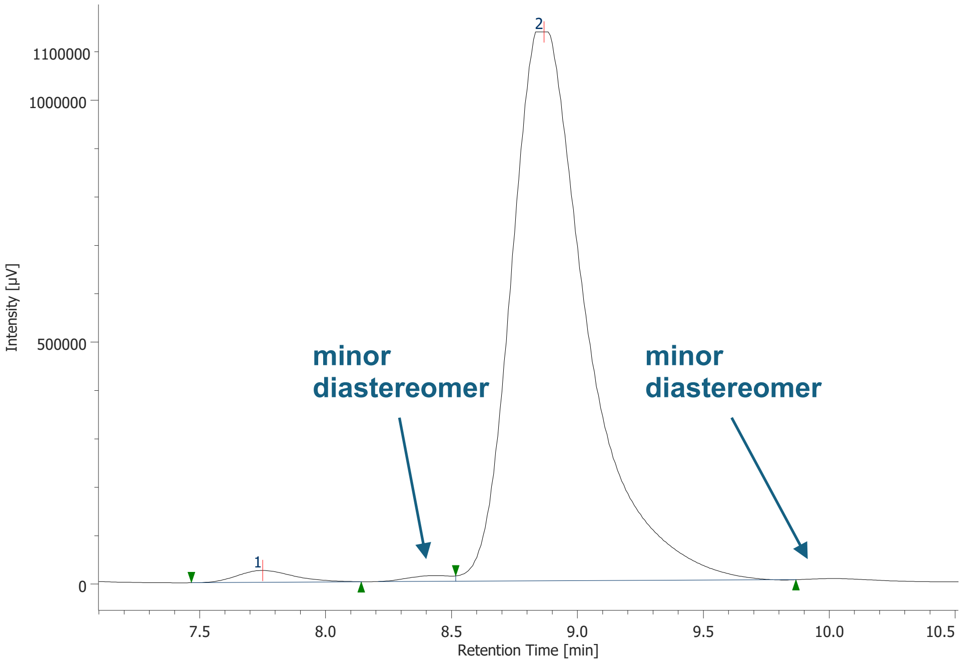

Peak No.	Retention Time (min)	Area (%)
1	7.750	1.571
2	8.867	98.429

3pc

IF-3 / 2-PrOH 3%

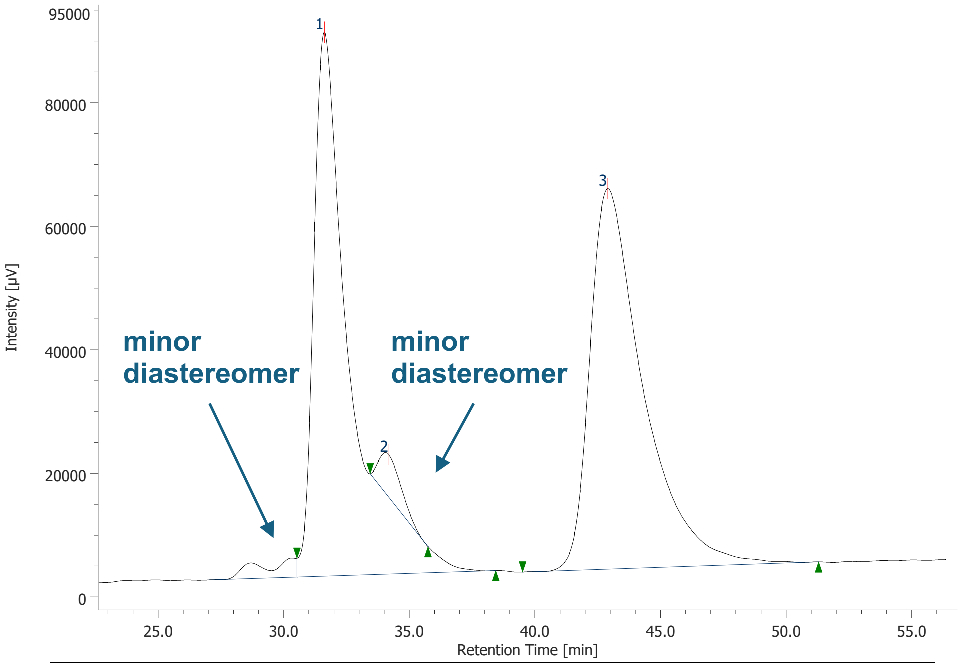

Peak No.	Retention Time (min)	Area (%)
1	31.617	48.428
2	34.183	2.310 (minor diastereomer)
3	42.900	49.262

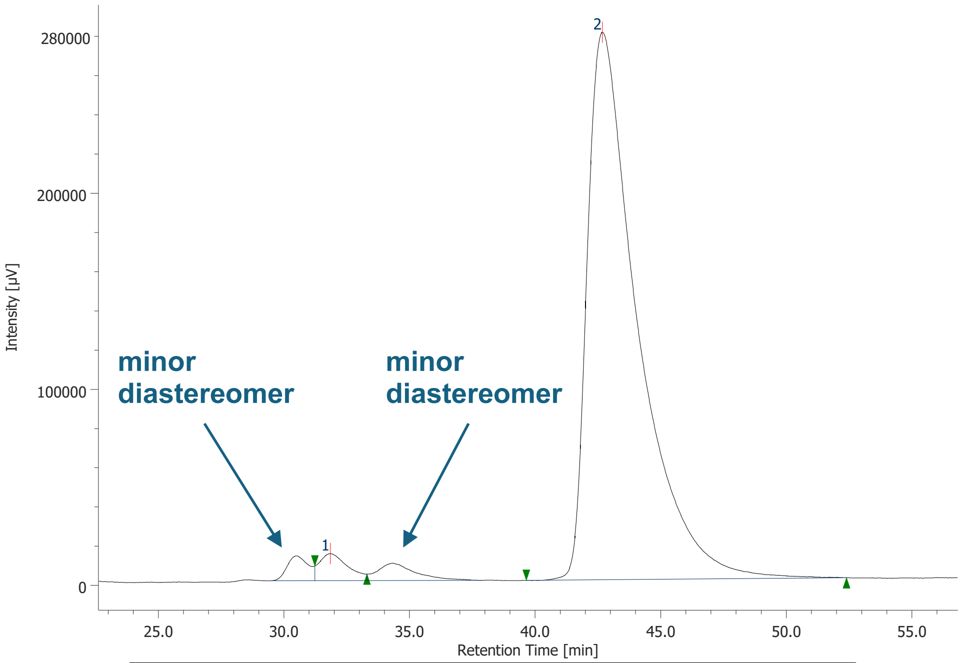

Peak No.	Retention Time (min)	Area (%)
1	31.842	2.701
2	42.675	97.299

3qa

IE-3 / 2-PrOH 15%

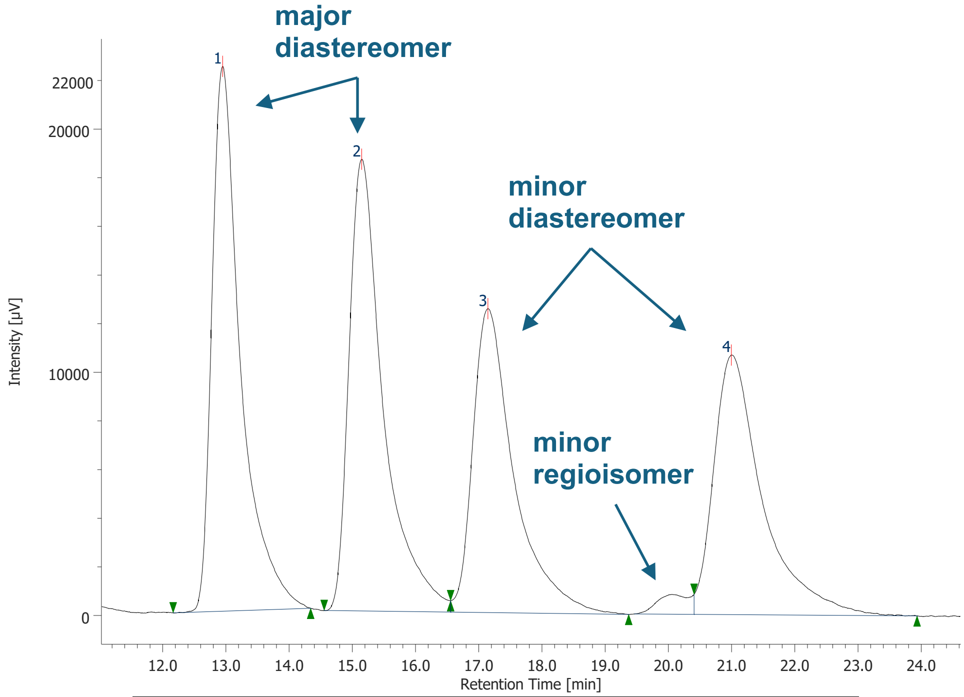

Peak No.	Retention Time (min)	Area (%)
1	12.947	27.806
2	15.147	27.586
3	17.143	22.186
4	20.997	22.422

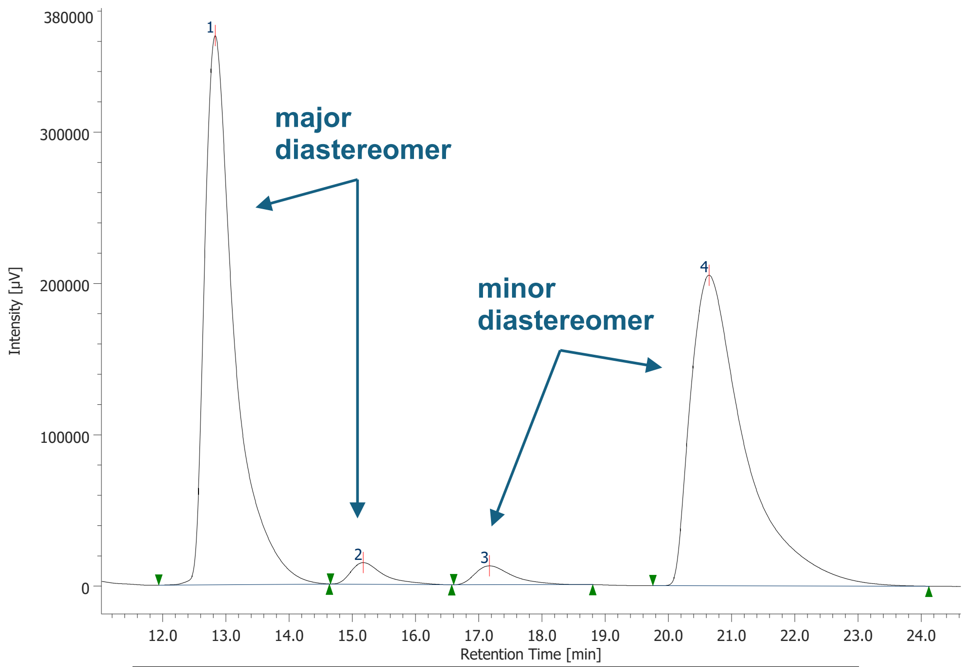

Peak No.	Retention Time (min)	Area (%)
1	12.830	47.420
2	15.170	2.038
3	17.167	2.048
4	20.643	48.493

3ra

IF-3 / 2-PrOH 20%

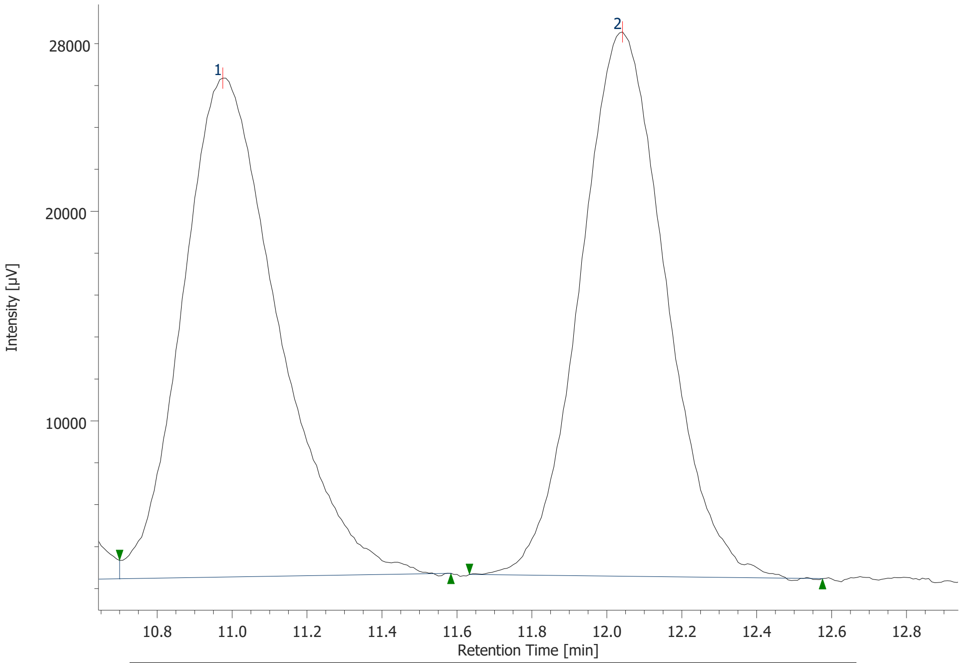

Peak No.	Retention Time (min)	Area (%)
1	10.975	50.679
2	12.042	49.321

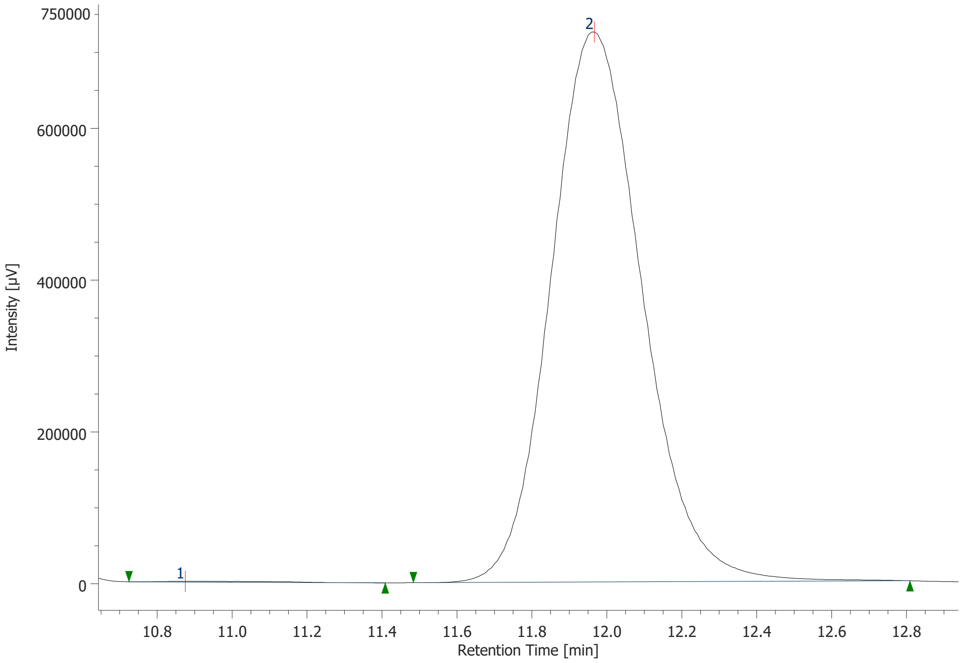

Peak No.	Retention Time (min)	Area (%)
1	10.875	0.172
2	11.967	99.828

3sa

IG-3 / 2-PrOH 8%

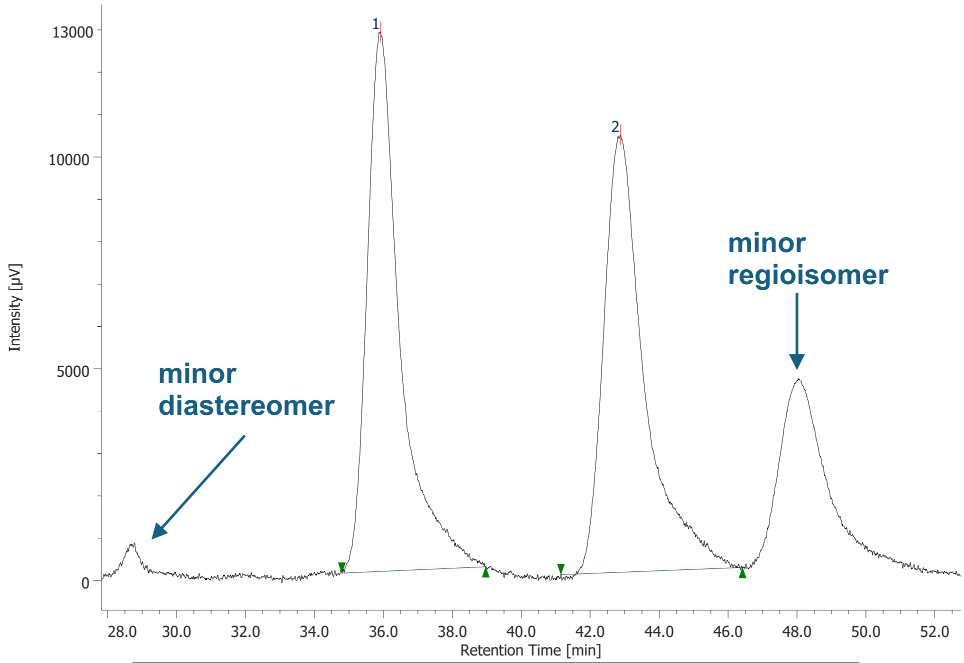

Peak No.	Retention Time (min)	Area (%)
1	35.917	50.080
2	42.875	49.920

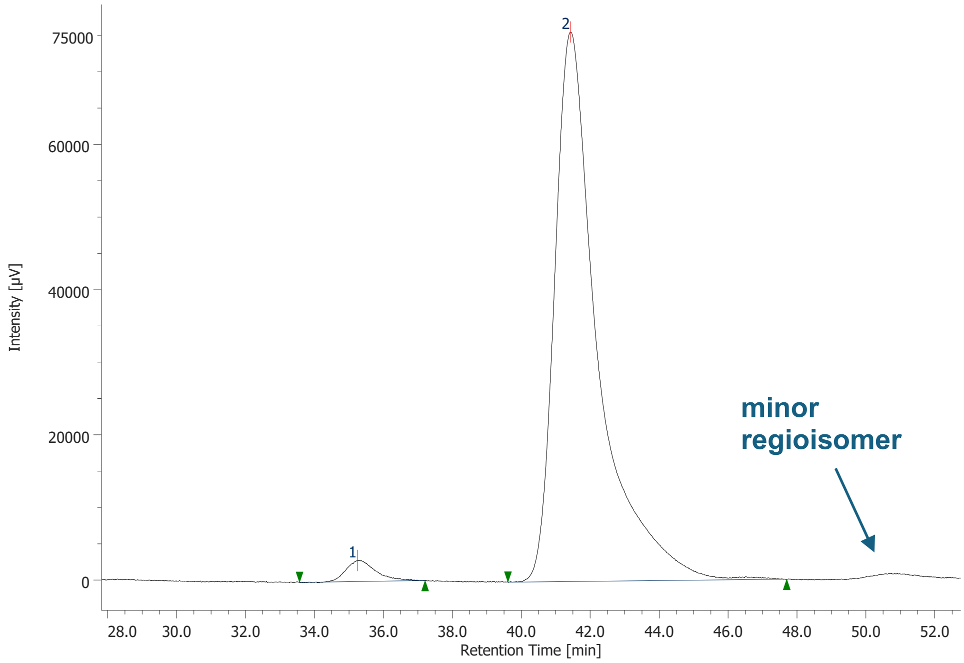

Peak No.	Retention Time (min)	Area (%)
1	35.250	2.579
2	41.433	97.421

3si

ID-3+ IE-3 / 2-PrOH 5%

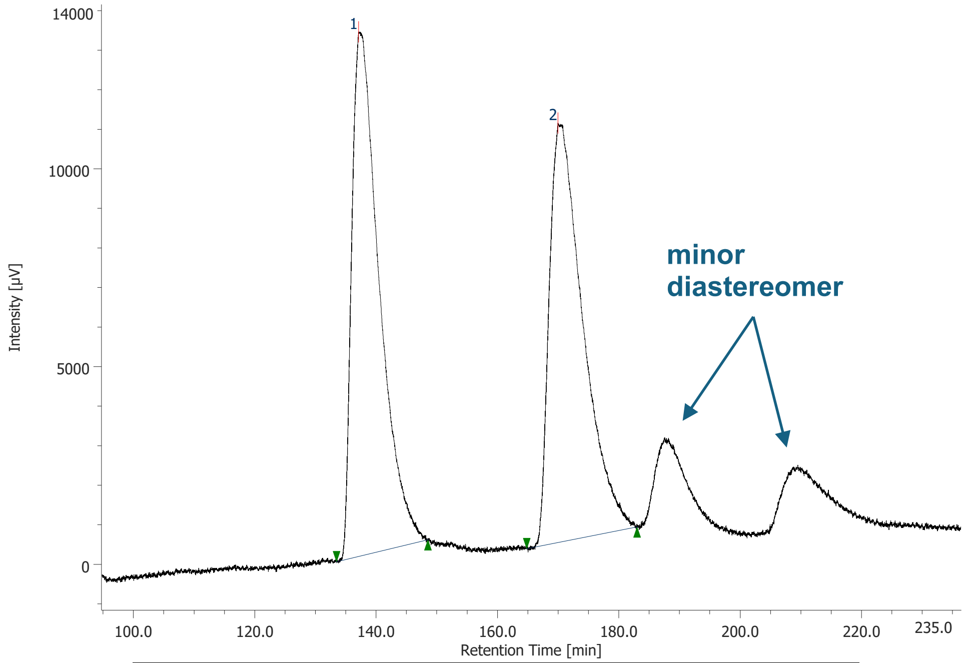

Peak No.	Retention Time (min)	Area (%)
1	137.100	50.643
2	169.967	49.357

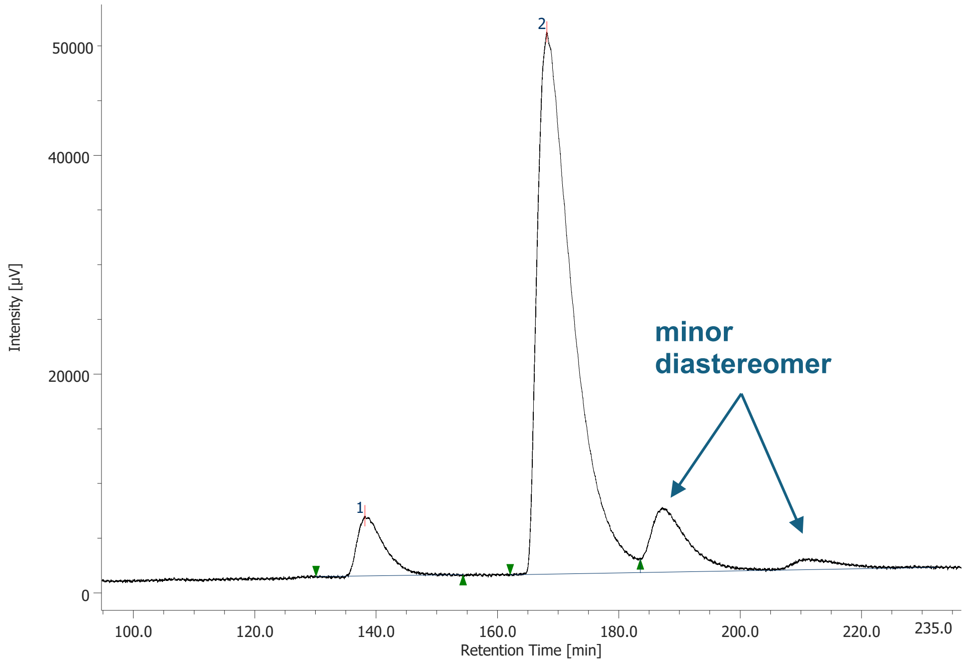

Peak No.	Retention Time (min)	Area (%)
1	138.133	7.693
2	168.100	92.307

3ta

IG-3 / 2-PrOH 3%

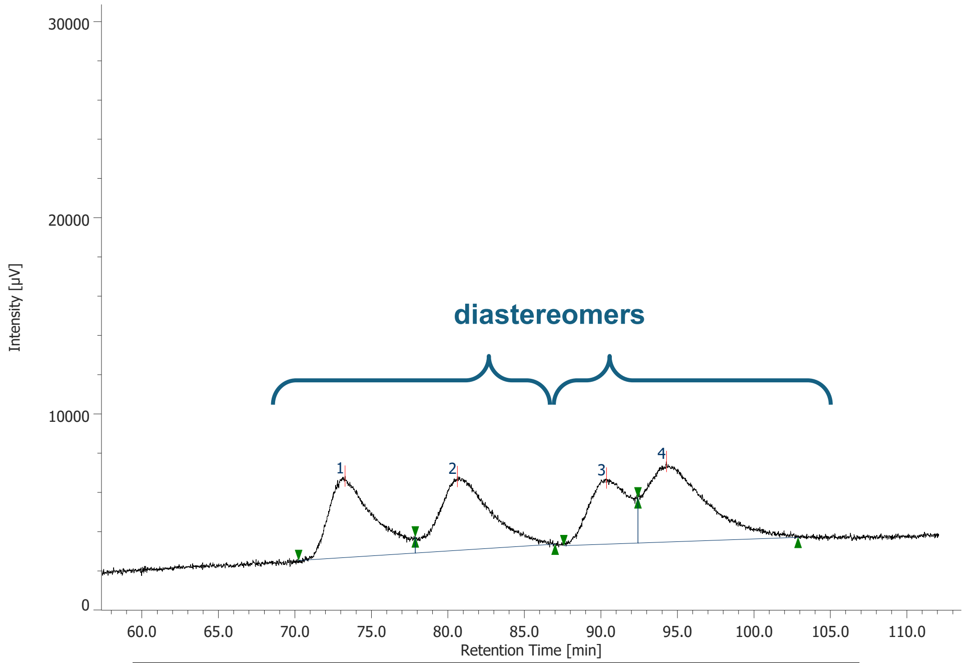

Peak No.	Retention Time (min)	Area (%)
1	73.275	24.550
2	80.617	25.678
3	90.358	17.859
4	94.275	31.913

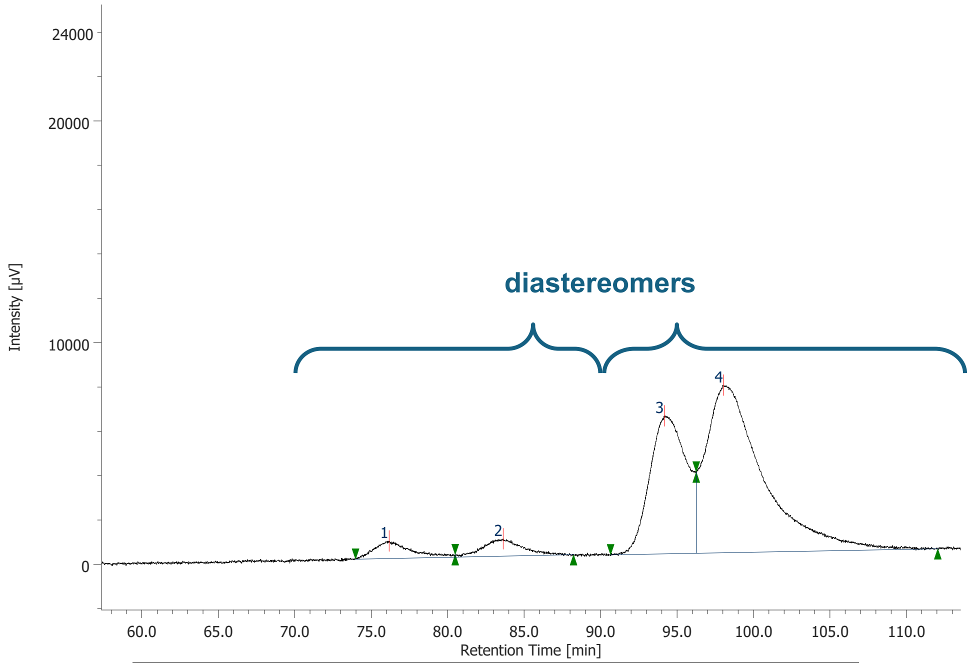

Peak No.	Retention Time (min)	Area (%)
1	76.167	3.875
2	83.625	4.156
3	94.167	31.768
4	98.042	60.201

3am

IF-3 / 2-PrOH 20%

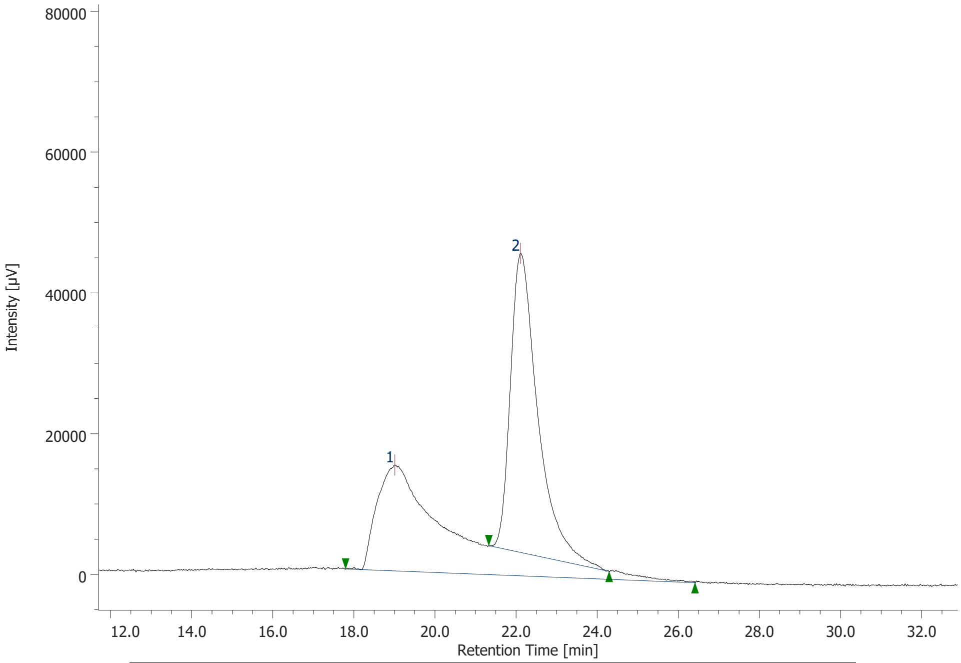

Peak No.	Retention Time (min)	Area (%)
1	19.008	50.745
2	22.108	49.255

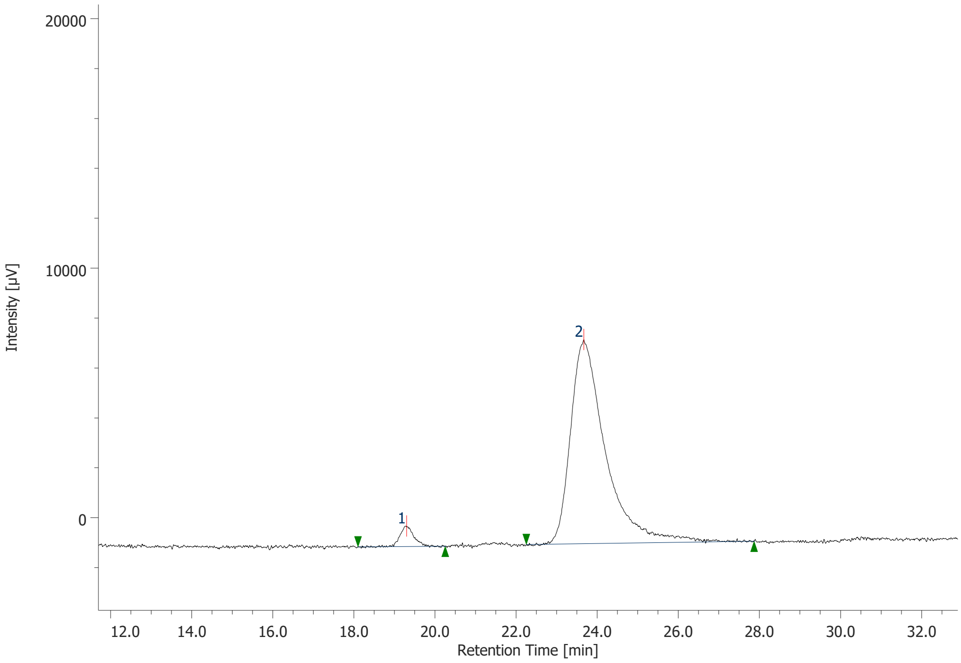

Peak No.	Retention Time (min)	Area (%)
1	19.300	3.982
2	23.667	96.018

3am’ (epimerized from **3am**)
IF-3 / 2-PrOH 20%

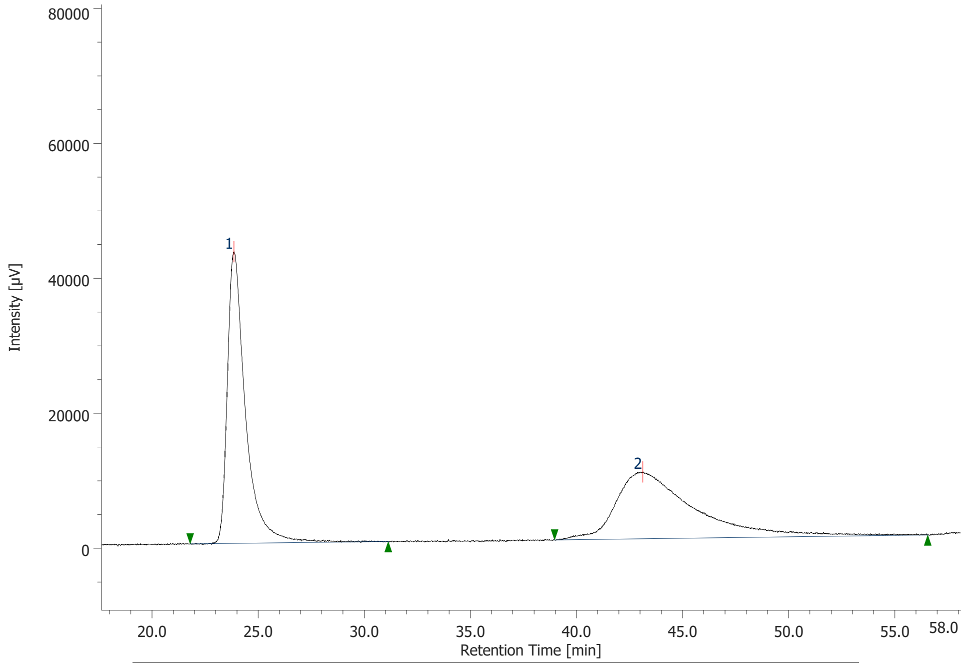

Peak No.	Retention Time (min)	Area (%)
1	23.858	50.255
2	43.125	49.745

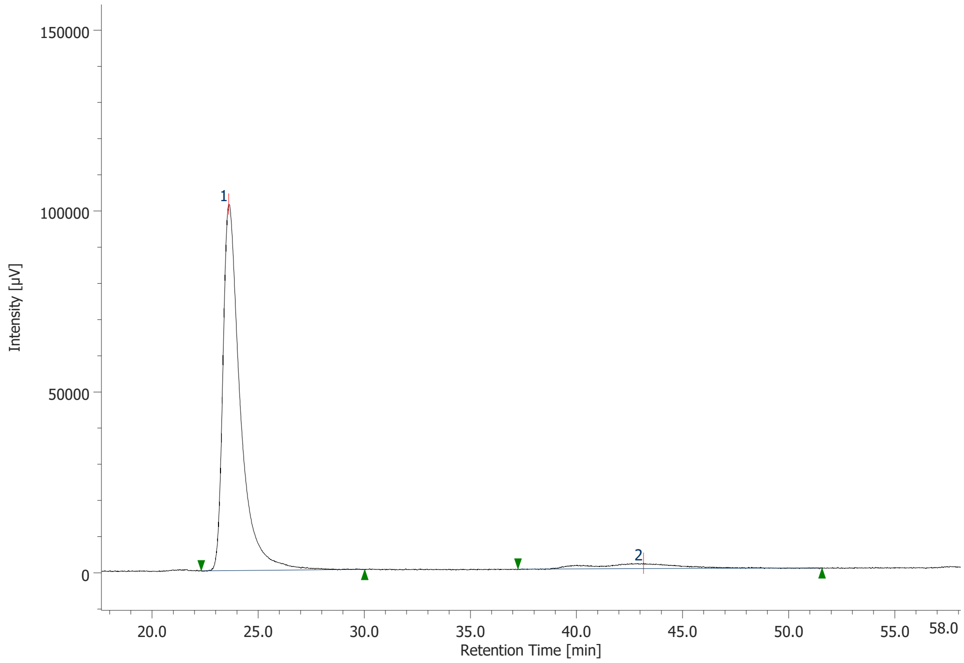

Peak No.	Retention Time (min)	Area (%)
1	23.617	93.687
2	43.158	6.313

3sn

IF-3 / 2-PrOH 40%

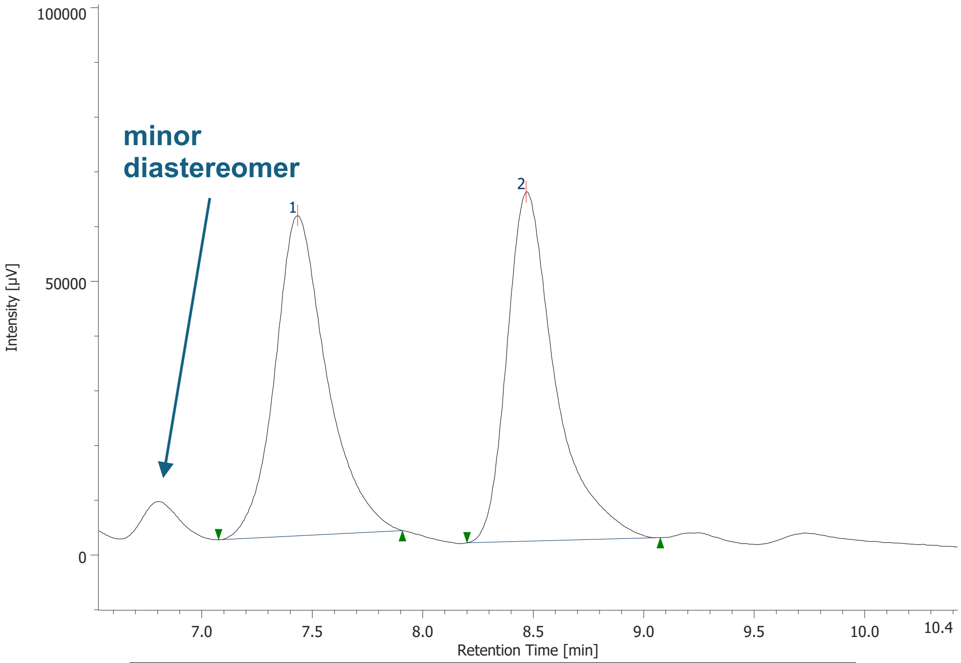

Peak No.	Retention Time (min)	Area (%)
1	7.433	49.641
2	8.467	50.359

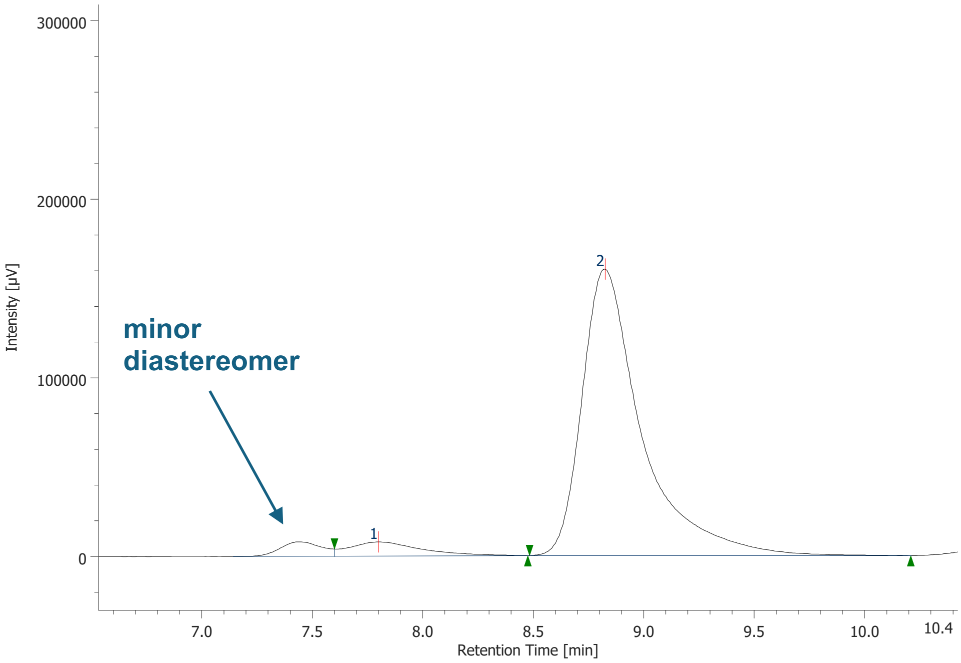

Peak No.	Retention Time (min)	Area (%)
1	7.800	5.554
2	8.825	94.446

3no

ID-3 / 2-PrOH 30%

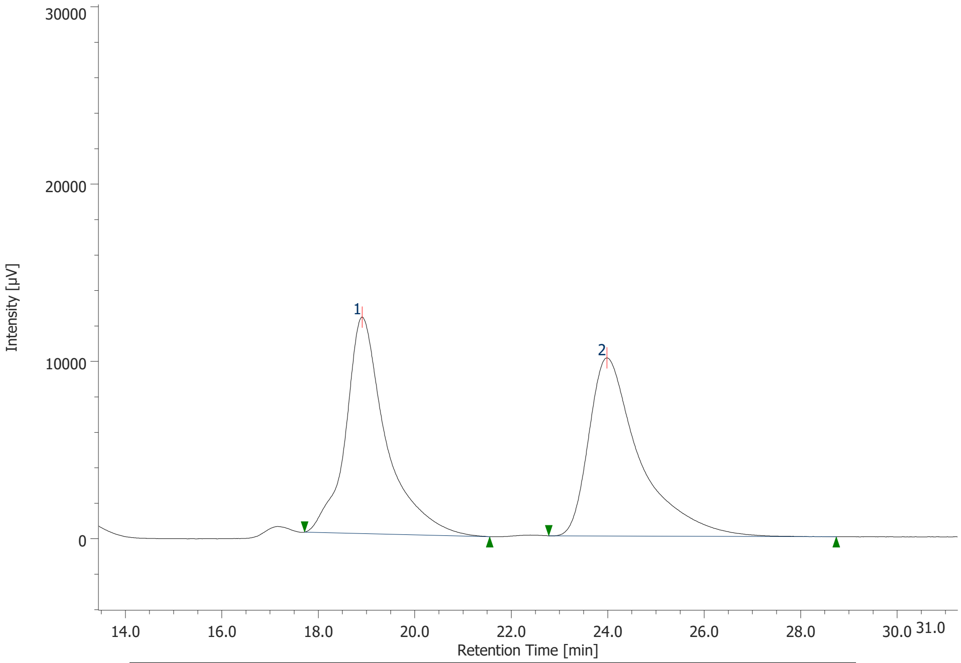

Peak No.	Retention Time (min)	Area (%)
1	18.908	49.118
2	23.983	50.882

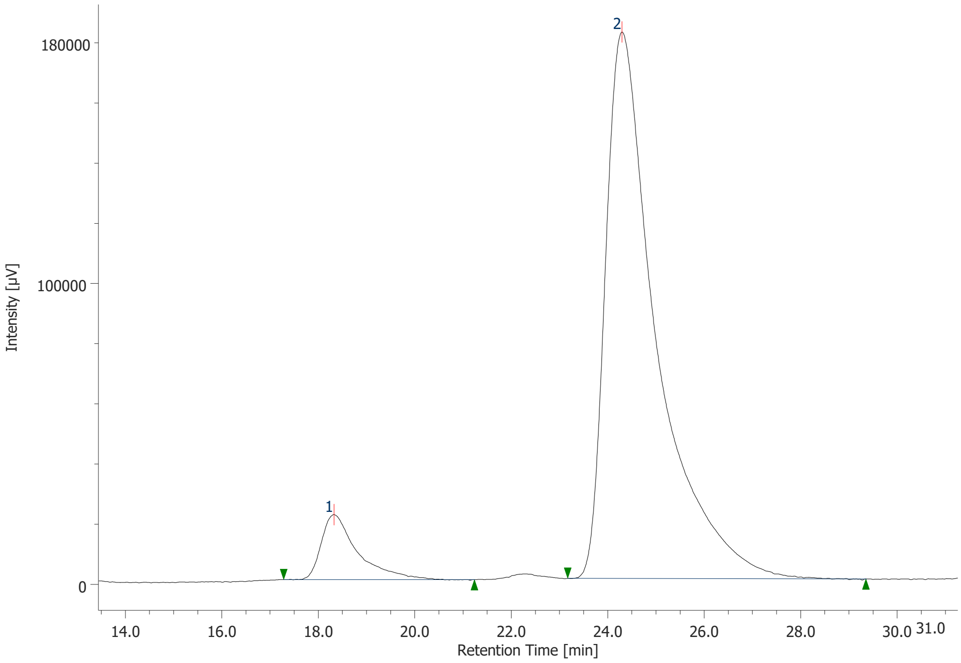

Peak No.	Retention Time (min)	Area (%)
1	18.325	7.771
2	24.292	92.229

4no

IG-3 / 2-PrOH 30%

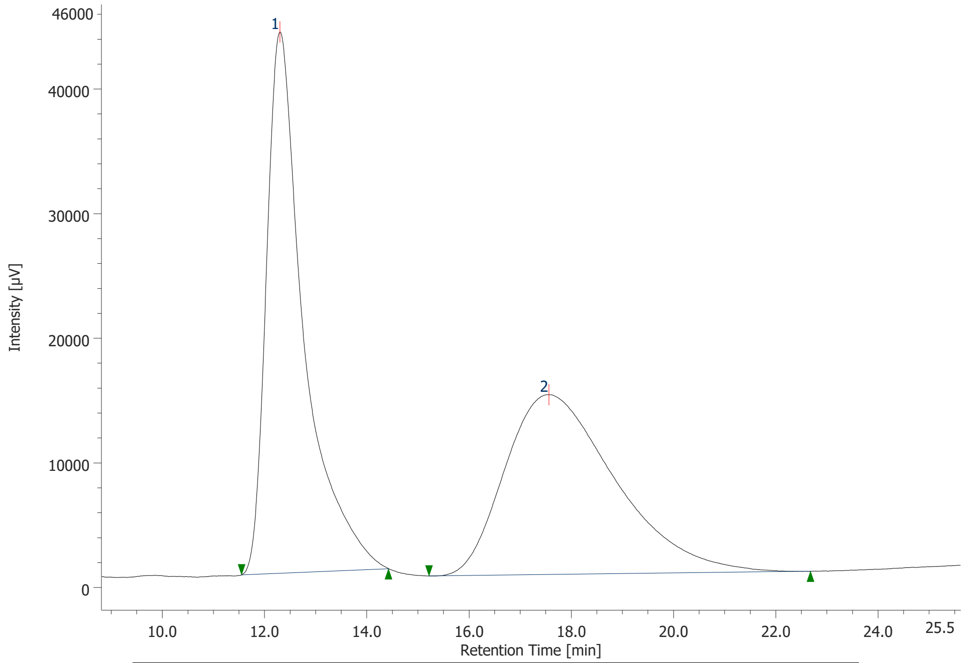

Peak No.	Retention Time (min)	Area (%)
1	12.300	50.637
2	17.558	49.363

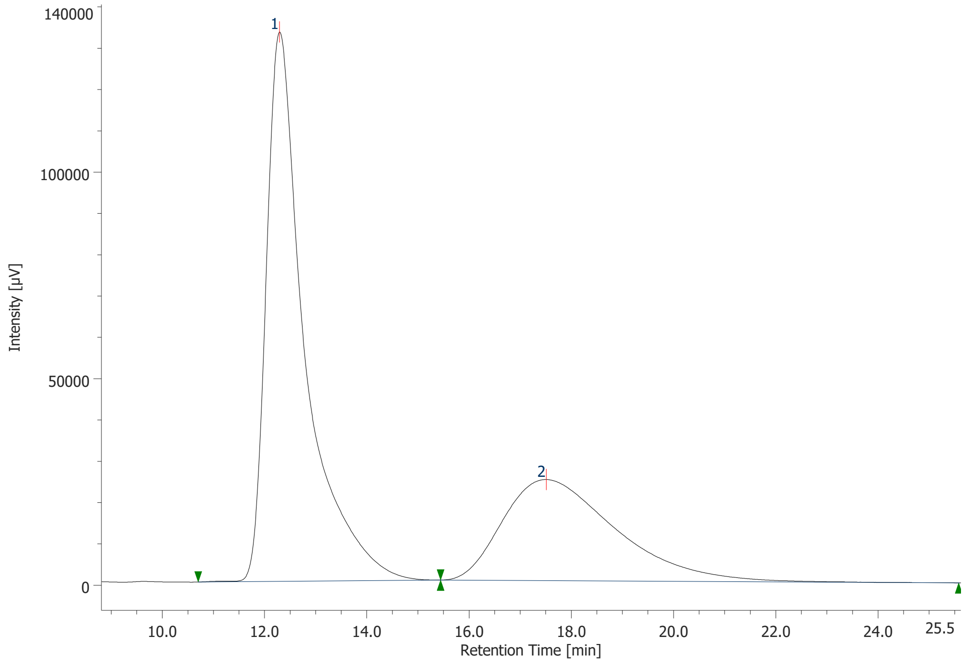

Peak No.	Retention Time (min)	Area (%)
1	12.292	65.086
2	17.508	34.914

3un

IG-3 / 2-PrOH 10%

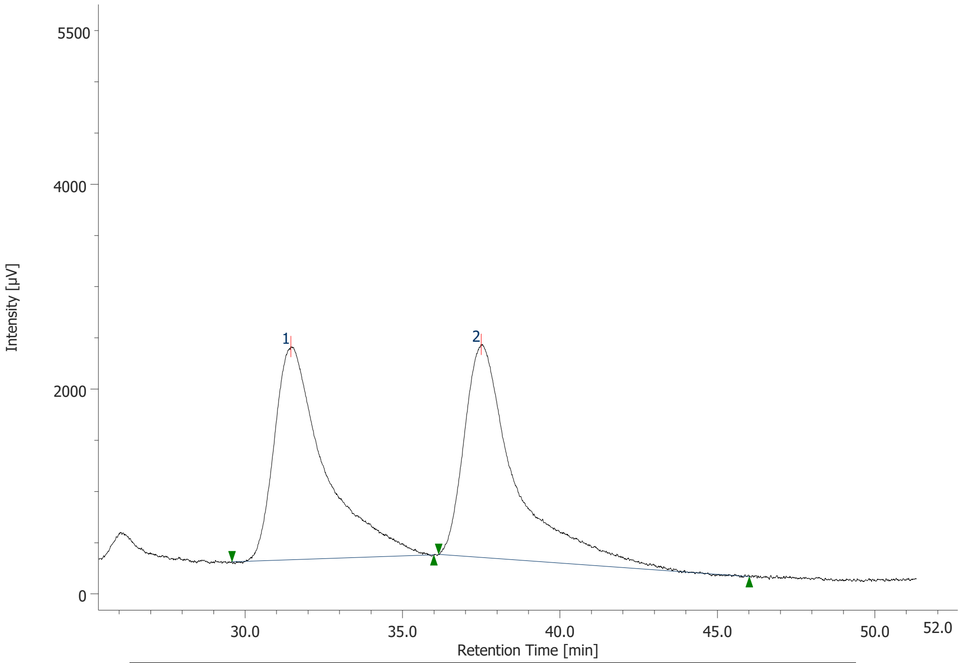

Peak No.	Retention Time (min)	Area (%)
1	31.450	49.547
2	37.492	50.453

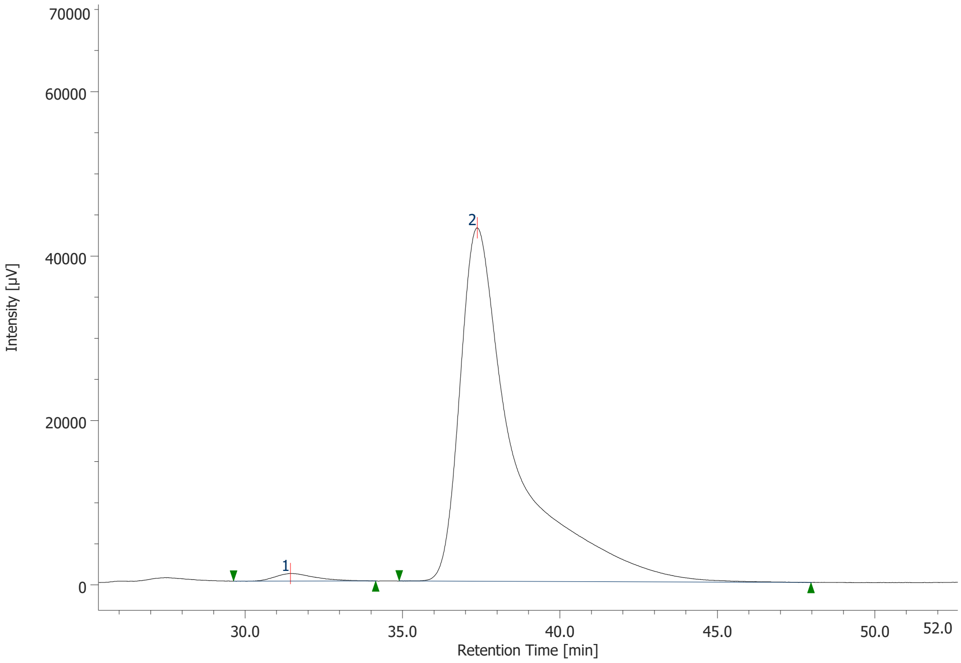

Peak No.	Retention Time (min)	Area (%)
1	31.442	1.511
2	37.367	98.489

4un

IF-3 / 2-PrOH 10%

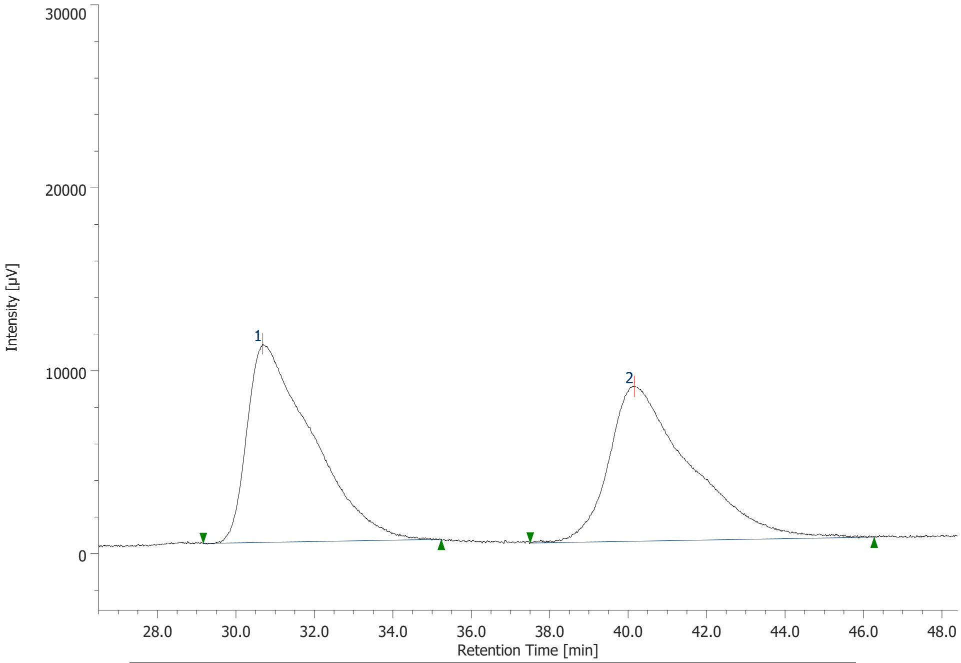

Peak No.	Retention Time (min)	Area (%)
1	30.683	50.634
2	40.158	49.366

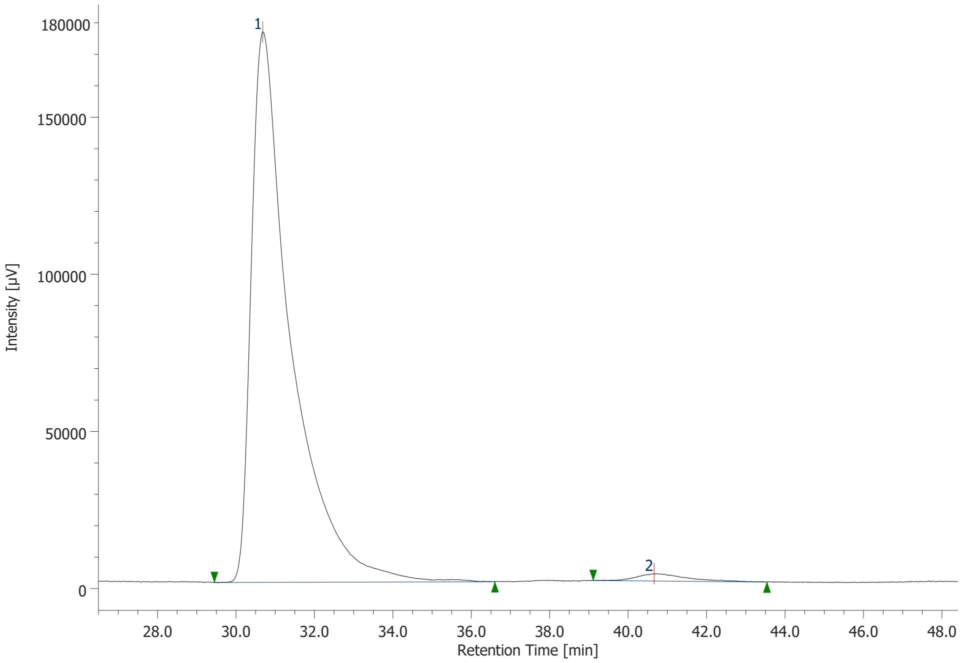

Peak No.	Retention Time (min)	Area (%)
1	30.683	98.346
2	40.658	1.654

5

IF-3 / 2-PrOH 15%

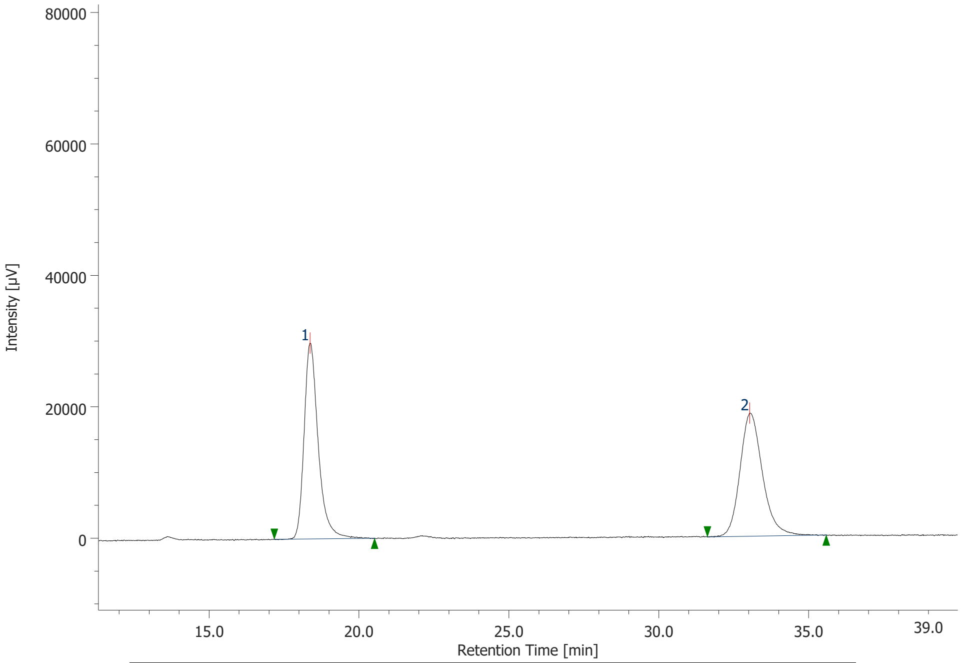

Peak No.	Retention Time (min)	Area (%)
1	18.367	50.113
2	33.033	49.887

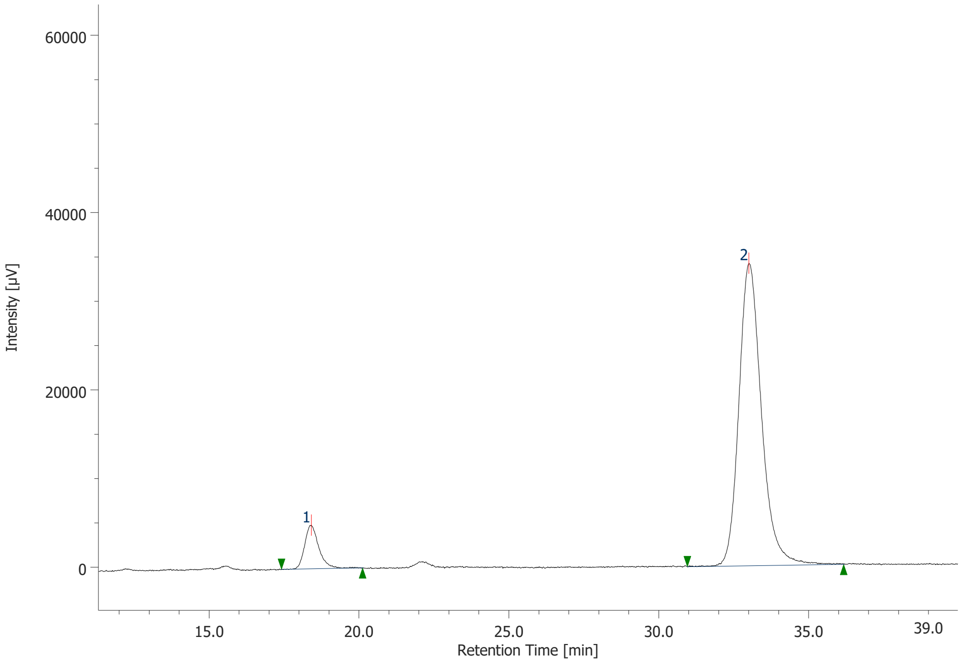

Peak No.	Retention Time (min)	Area (%)
1	18.408	8.245
2	33.000	91.755

6

IG-3 / 2-PrOH 20%

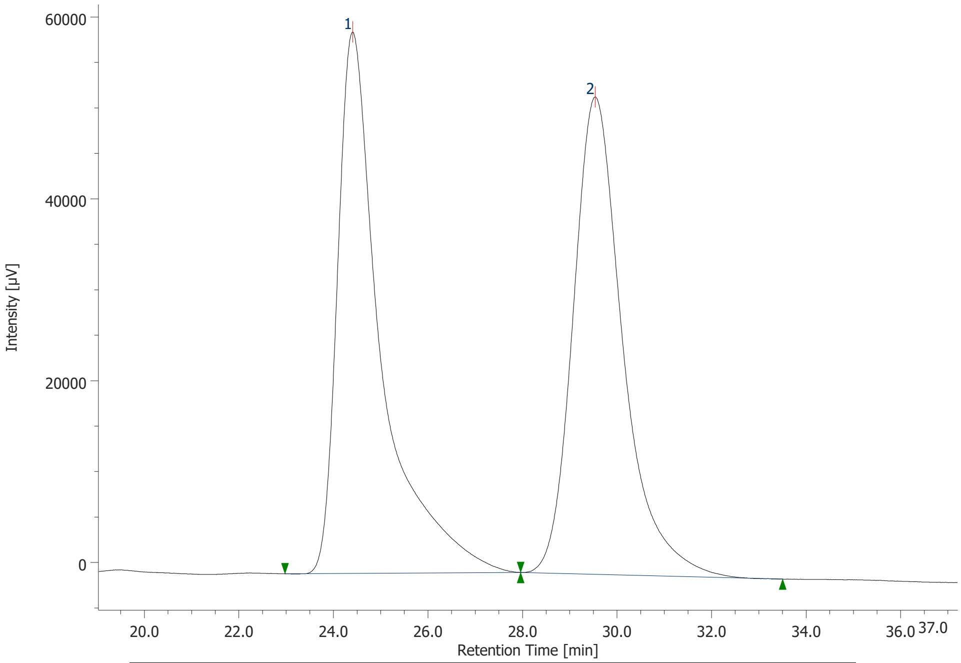

Peak No.	Retention Time (min)	Area (%)
1	24.408	49.943
2	29.533	50.057

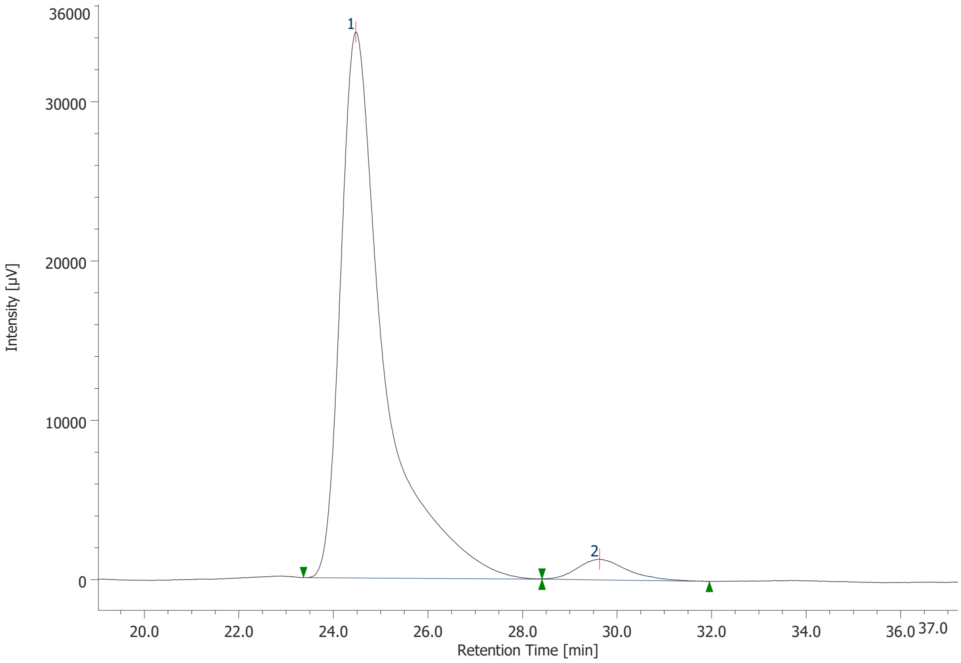

Peak No.	Retention Time (min)	Area (%)
1	24.475	95.903
2	29.625	4.097

7

IF-3 / 2-PrOH 8%

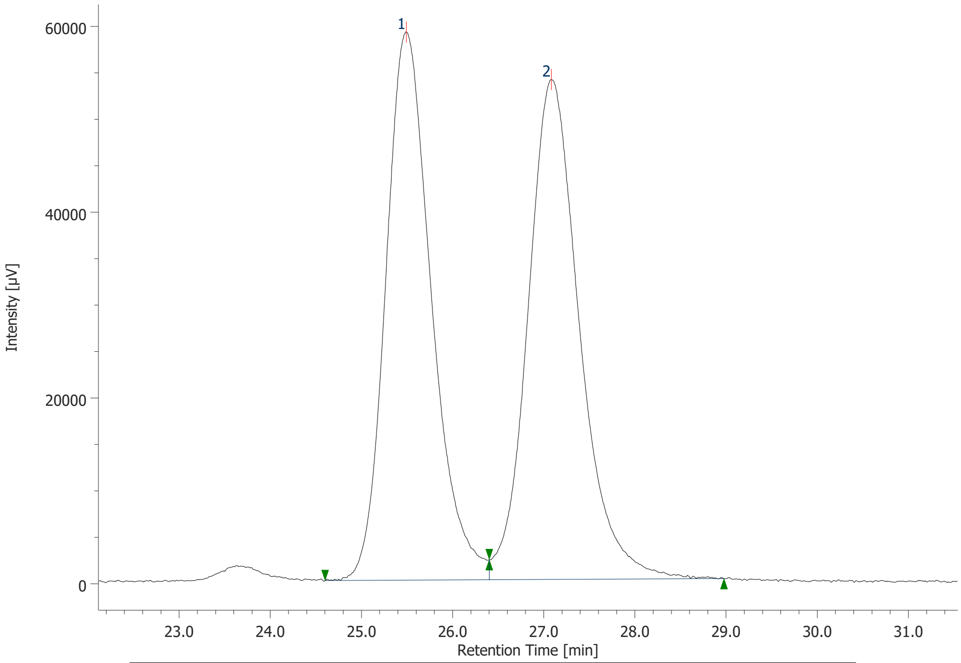

Peak No.	Retention Time (min)	Area (%)
1	25.492	49.306
2	27.083	50.694

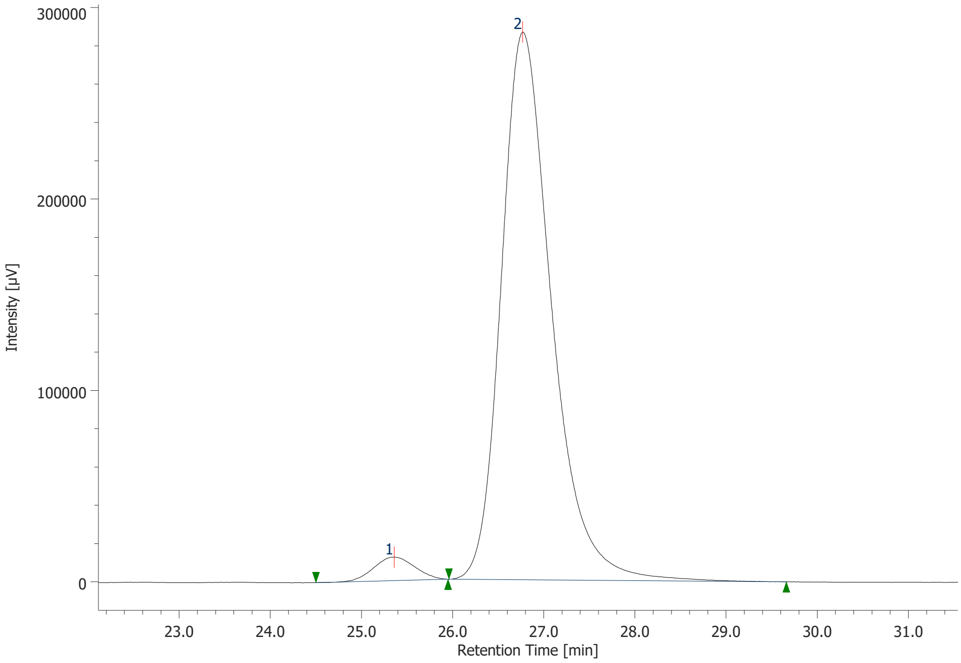

Peak No.	Retention Time (min)	Area (%)
1	25.358	3.352
2	26.767	96.648

3ap

IE-3 / 2-PrOH 30%

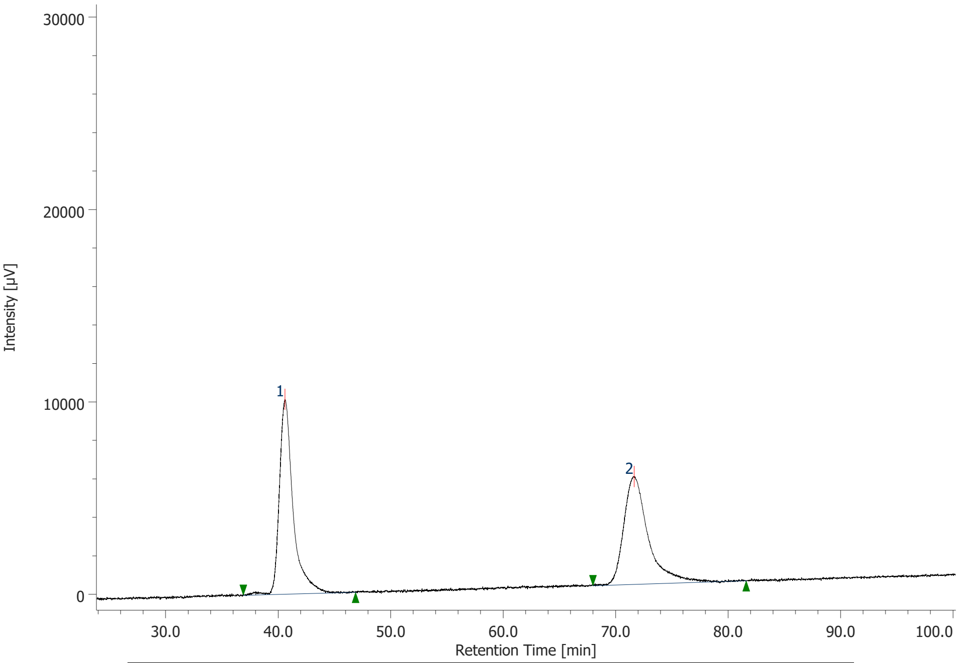

Peak No.	Retention Time (min)	Area (%)
1	40.600	50.343
2	71.642	49.657

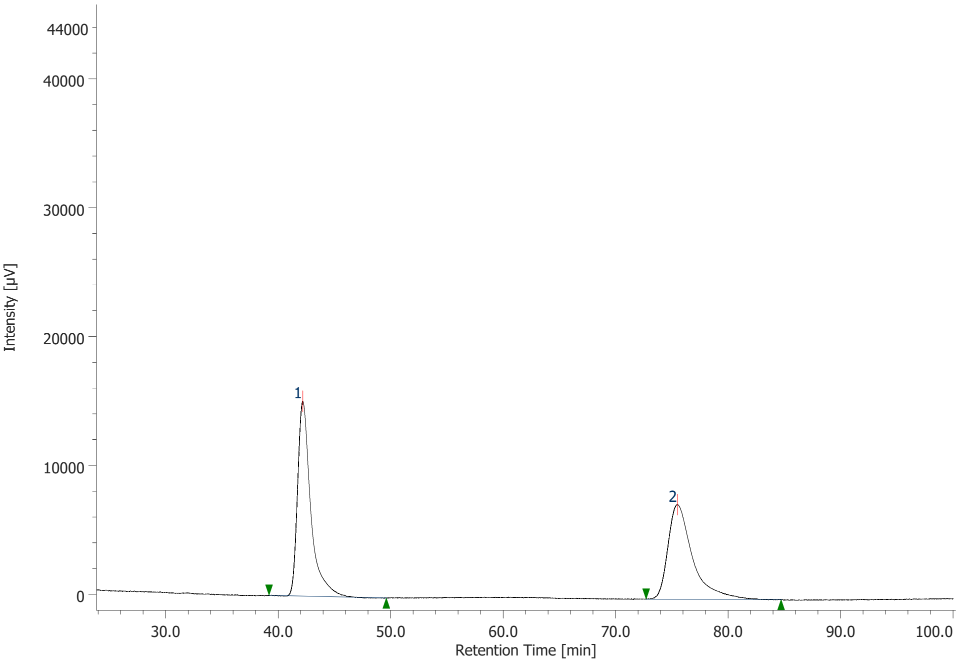

Peak No.	Retention Time (min)	Area (%)
1	42.197	52.522
2	75.510	47.478

4ap

IE-3 / 2-PrOH 20%

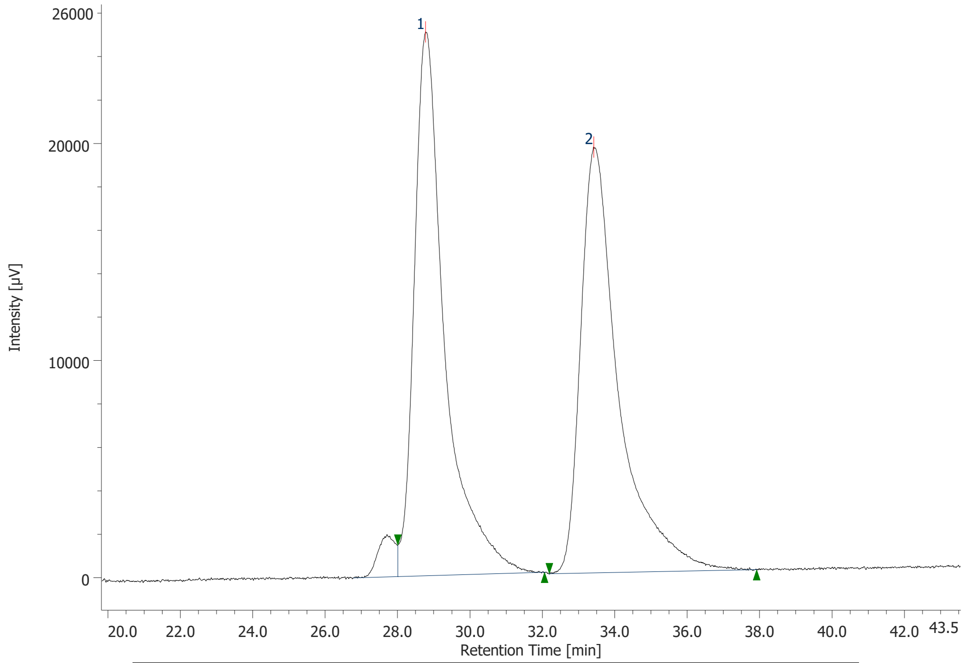

Peak No.	Retention Time (min)	Area (%)
1	28.775	50.598
2	33.417	49.402

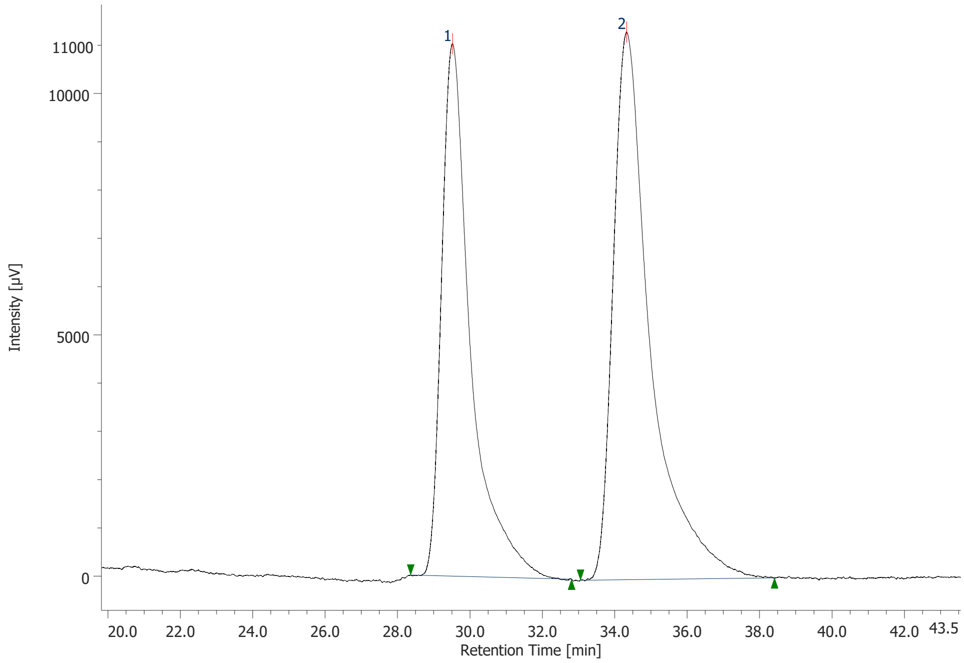

Peak No.	Retention Time (min)	Area (%)
1	29.517	43.129
2	34.327	56.871

3aq+4aq

IG-3 / 2-PrOH 50%

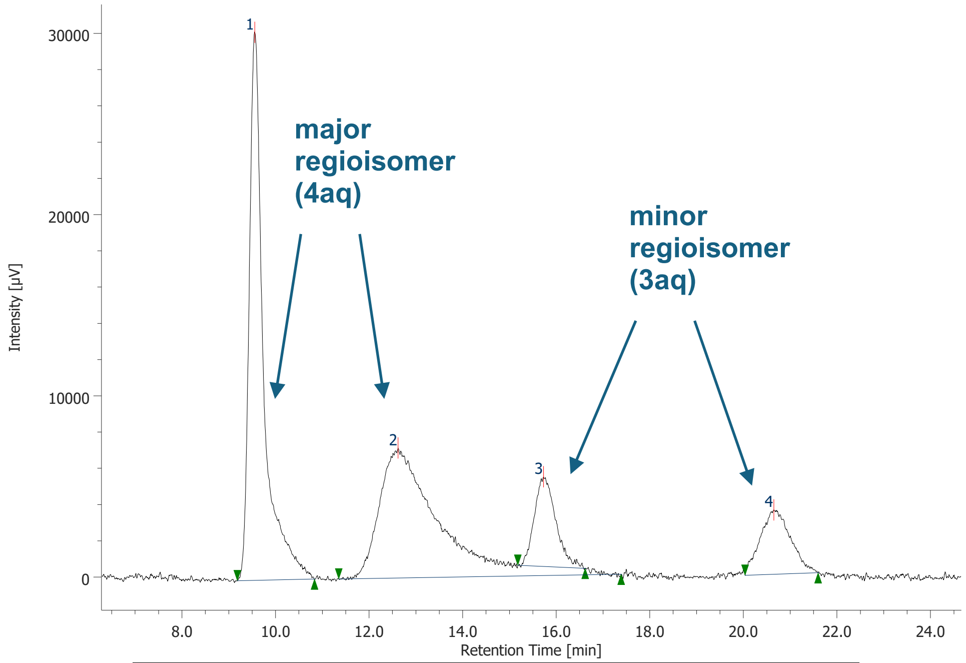

Peak No.	Retention Time (min)	Area (%)
1	9.558	40.265
2	12.617	39.999
3	15.725	9.811
4	20.650	9.925

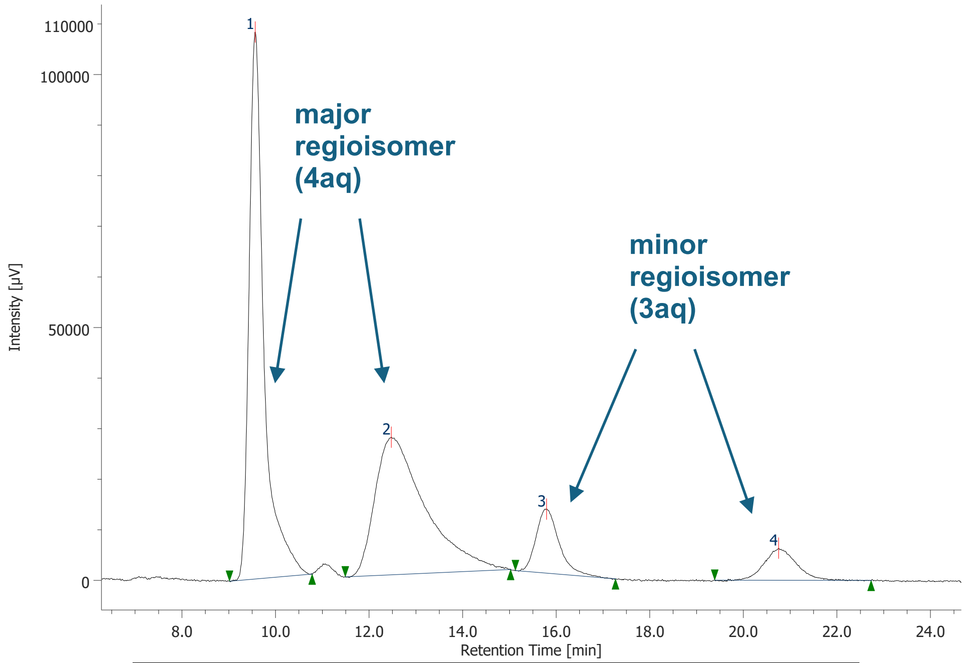

Peak No.	Retention Time (min)	Area (%)
1	9.567	46.446
2	12.475	39.398
3	15.792	8.372
4	20.750	5.784

3ea

IF-3 / 2-PrOH 5%

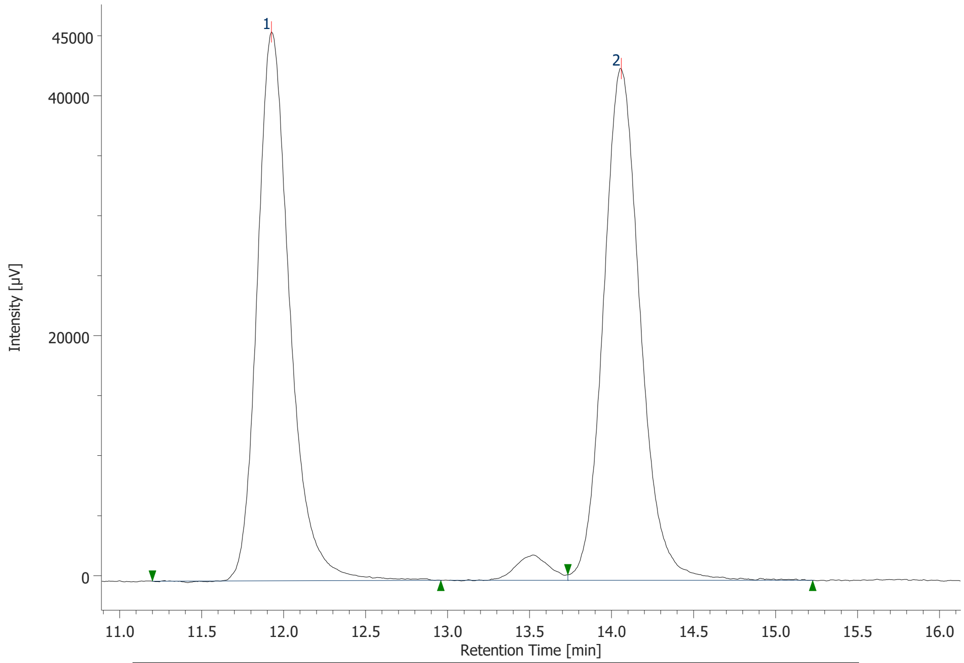

Peak No.	Retention Time (min)	Area (%)
1	11.925	48.698
2	14.058	51.302

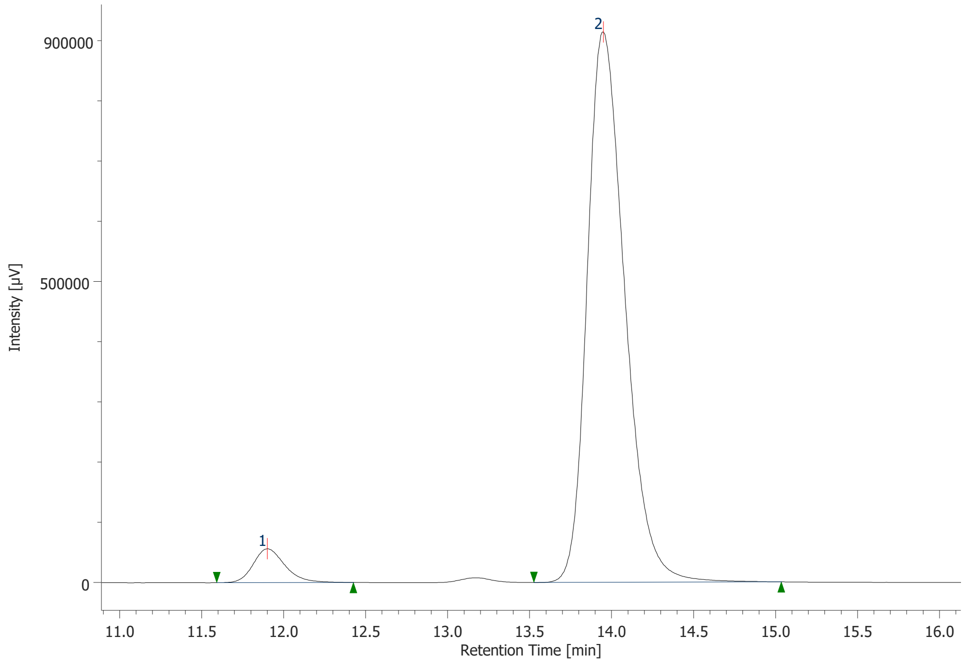

Peak No.	Retention Time (min)	Area (%)
1	11.900	4.925
2	13.950	95.075

4ea

IG-3 / 2-PrOH 5%

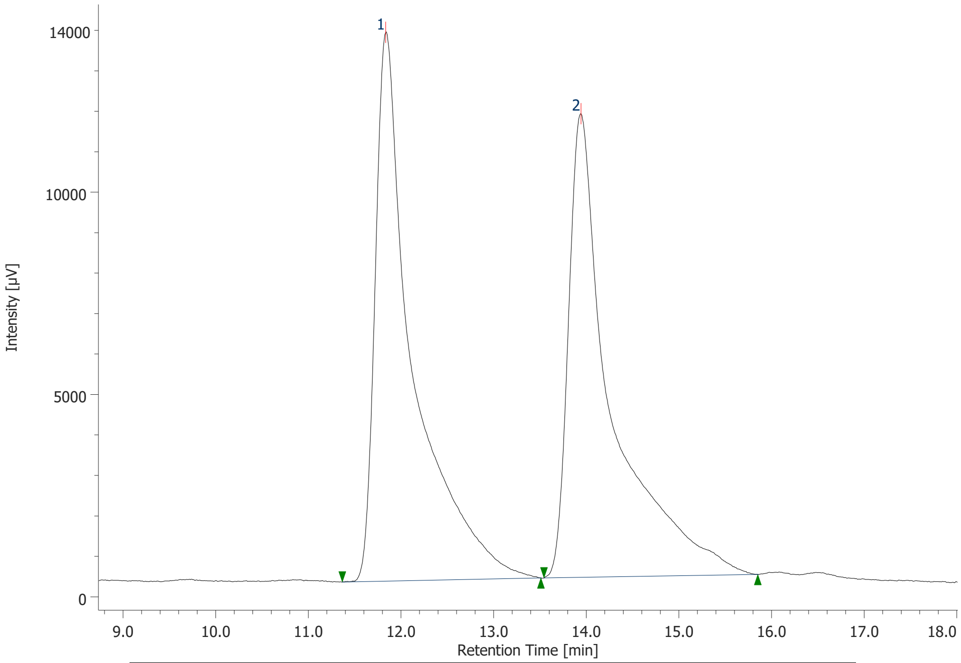

Peak No.	Retention Time (min)	Area (%)
1	11.833	50.905
2	13.942	49.095

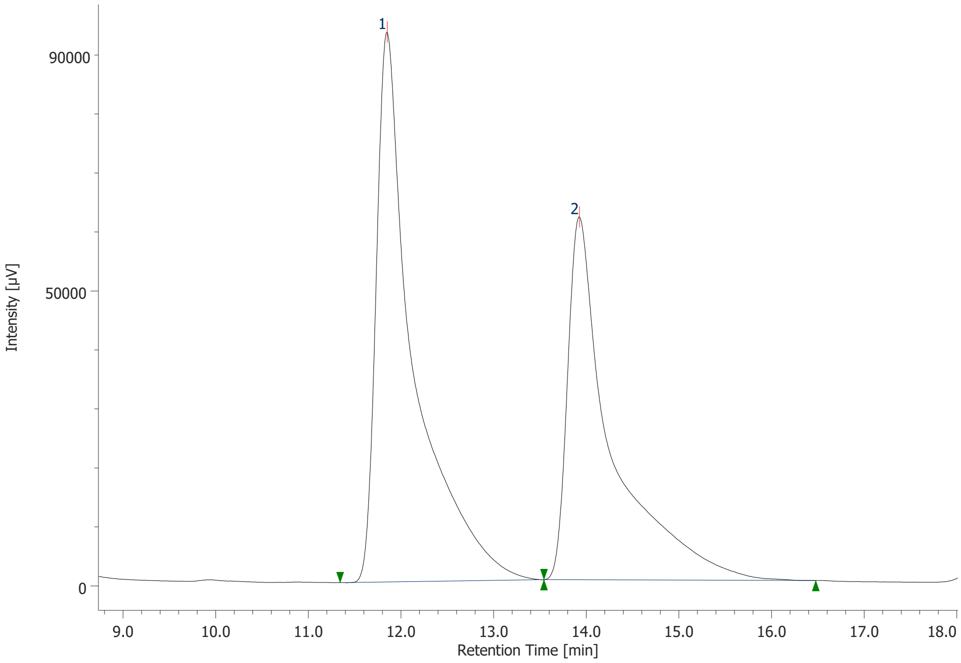

Peak No.	Retention Time (min)	Area (%)
1	11.850	56.419
2	13.925	43.581
